# Supplementary material for: Causes and consequences of child growth faltering in low-resource settings
Source: Nature. 2023 Sep 13;621(7979):568–76. doi: 10.1038/s41586-023-06501-x (PMC10511328; doi:10.1038/s41586-023-06501-x)
Supplement: Supplementary file 1 — This file contains Supplementary Notes 1–7 [file 41586_2023_6501_MOESM1_ESM.pdf]

nature portfolio

https://doi.org/10.1038/s41586-023-06501-x

## Supplementary information

# Causes and consequences of child growth faltering in low-resource settings

In the format provided by the authors and unedited

## Supplement Information for:

**Causes and consequences of child growth faltering in low- and middle-income countries**

Andrew Mertens,[1]* Jade Benjamin-Chung,[2,3,4] John M Colford Jr,[1] Jeremy Coyle[1], Mark J van der Laan,[1] Alan E Hubbard,[1] Sonali Rosete,[1] Ivana Malenica,[1] Nima Hejazi,[1] Oleg Sofrygin,[1] Wilson Cai,[1] Haodong Li,[1] Anna Nguyen,[1] Nolan N Pokpongkiat,[1] Stephanie Djajadi,[1] Anmol Seth,[1] Esther Jung,[1] Esther O Chung,[1] Wendy Jilek,[1] Vishak Subramoney,[5] Ryan Hafen,[6] Jonas Häggström,[7] Thea Norman,[8] Kenneth H Brown,[9] Parul Christian,[10] Benjamin F. Arnold,[11] and members of the *ki* Child Growth Consortium

[1] Division of Epidemiology & Biostatistics, University of California, Berkeley, 2121 Berkeley Way Rm 5302 Berkeley, CA 94720-7360

[2] Department of Epidemiology & Population Health, Stanford University, Stanford University, 300 Pasteur Dr., Stanford, CA 94305

[3] Division of Epidemiology & Biostatistics, University of California, Berkeley, 2121 Berkeley Way Rm 5302 Berkeley, CA 94720-7360

[4] Chan Zuckerberg Biohub, San Francisco, CA 94158

[5] DVPL Tech

[6] Hafen Consulting, LLC, West Richland WA, 99353

[7] Cytel Inc, 1050 Winter St Suite 2700 Waltham, MA 02451, USA

[8] Quantitative Sciences, Bill & Melinda Gates Foundation, 500 5th Ave N, Seattle, WA 98109

[9] Department of Nutrition, University of California, Davis, 3135 Meyer Hall, Davis, CA 95616-5270

[10] Center for Human Nutrition, Department of International Health, Johns Hopkins Bloomberg School of Public Health, Baltimore, MD 21205

[11] Francis I. Proctor Foundation and Department of Ophthalmology, University of California, San Francisco, 490 Illinois St, San Francisco, CA 94158

**Corresponding authors:**

Andrew Mertens, PhD, amertens@berkeley.edu, Research Data Analyst, Division of Epidemiology & Biostatistics, University of California, Berkeley, 2121 Berkeley Way Rm 5302 Berkeley, CA 94720-7360

Benjamin F. Arnold, PhD, ben.arnold@ucsf.edu, Associate Professor, Francis I. Proctor Foundation, University of California, San Francisco, 490 Illinois St, San Francisco, CA 94158

# Table of Contents

Supplementary Note 1. Directed Acyclic Diagrams (DAGs) .......... 2
1.1 Overview .......... 2
1.2 Simplified example DAG: .......... 3
1.3 DAG used: Association between maternal weight and child growth faltering .......... 4
1.4 Table of Adjustment Covariates .......... 4
Supplementary Note 2. Complete-Case Sensitivity Analyses .......... 9
2.1 Heatmap of covariate missingness by study .......... 9
2.2 Sensitivity analysis to complete case versus median/mode imputed analyses .......... 9
Plots comparing population intervention effect estimates (the estimates presented in Figures 2 and 3) compared to estimates only using complete-case data (excluding any observations missing covariate information). .......... 9
Supplementary Note 3. Sensitivity analysis using fixed effects models .......... 11
3.1 Overview .......... 11
3.2 Primary manuscript figures recreated with estimates pooled using fixed effects .......... 12
Supplementary Note 4. Comparison of Adjusted and Unadjusted Population Intervention Effects .......... 17
Supplementary Note 5. Spline plots: all exposures .......... 17
Supplementary Note 6. Sensitivity to dropping PROBIT trial .......... 77
6.1 Overview .......... 77
6.2 Comparison of attributable differences estimated with and without the PROBIT trial .......... 78
Supplementary Note 7 Average treatment effect and relative risk plots .......... 78

# Supplementary Note 1. Directed Acyclic Diagrams (DAGs)

## 1.1 Overview

For each exposure, we used the directed acyclic graph (DAG) framework to identify potential confounders from the broader set of exposures used in the analysis. We did not adjust for characteristics that were assumed to be intermediate on the causal path between any exposure and the outcome, because while controlling for mediators may help adjust for unmeasured confounders in some conditions, it can also lead to collider bias. As DAG's were hand-drawn and are complex for the large number of exposures, we show an example DAG here and then list all potential considered for each exposure in a table below.

The DAG below shows the set of adjusted-for exposures and unadjusted-for exposures for the estimated association between maternal weight and child growth faltering. Grey variables are on the causal pathway and are not adjusted for, while blue variables were considered potential confounders and adjusted for. Note collinear variables like maternal BMI are shown as on the causal pathway in this DAG, which may not be biologically accurate, but they were not adjusted for so are included with the potential mediators.

Given the exposures measured in each study, we attempted to maximally adjust for confounding without adjusting for mediators on the casual pathway, which would remove part of the effect of the exposure on the outcome. Confounders were not measured in every cohort, so there could be residual confounding in cohort-specific estimates, and actual DAGs will look different across cohort-specific estimates for each study.

## 1.2 Simplified example DAG:

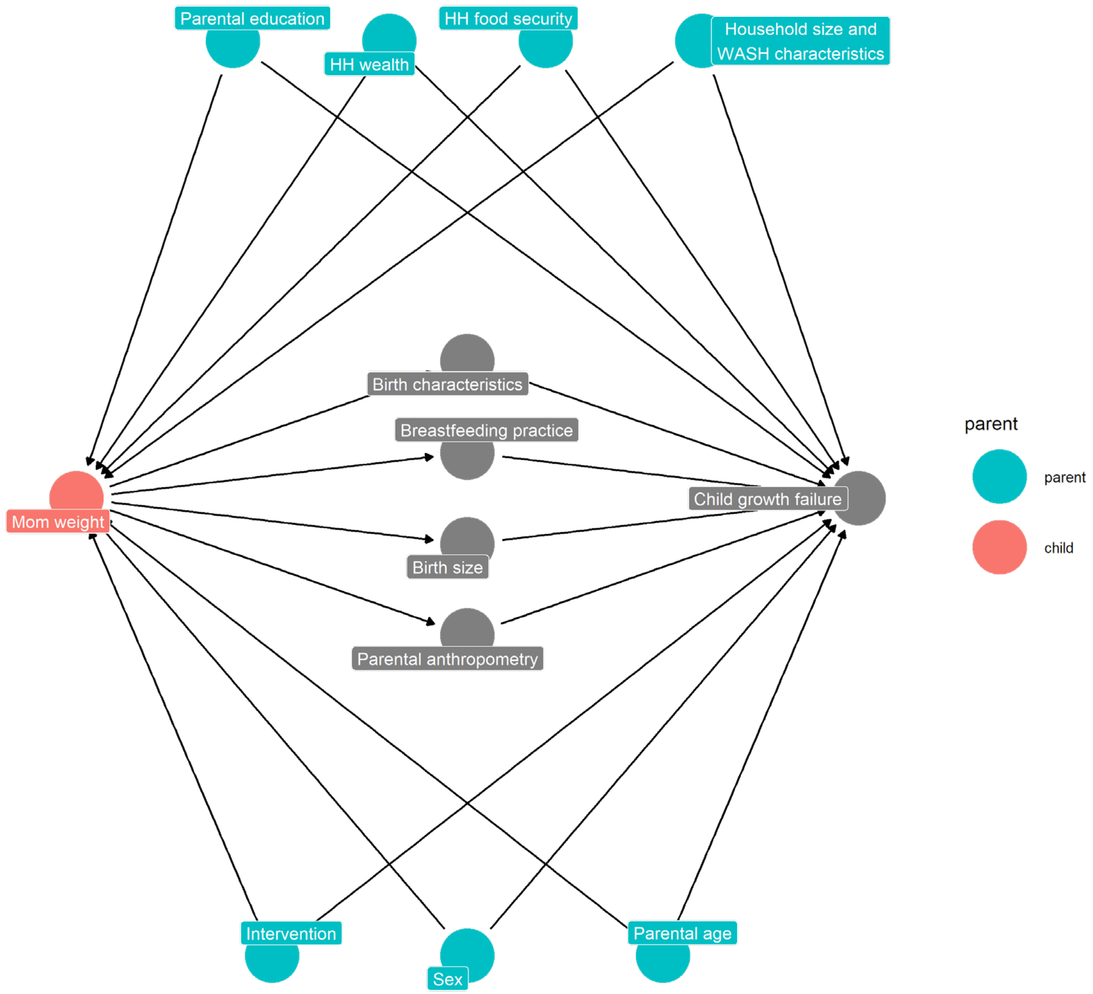

## 1.3 DAG used: Association between maternal weight and child growth faltering

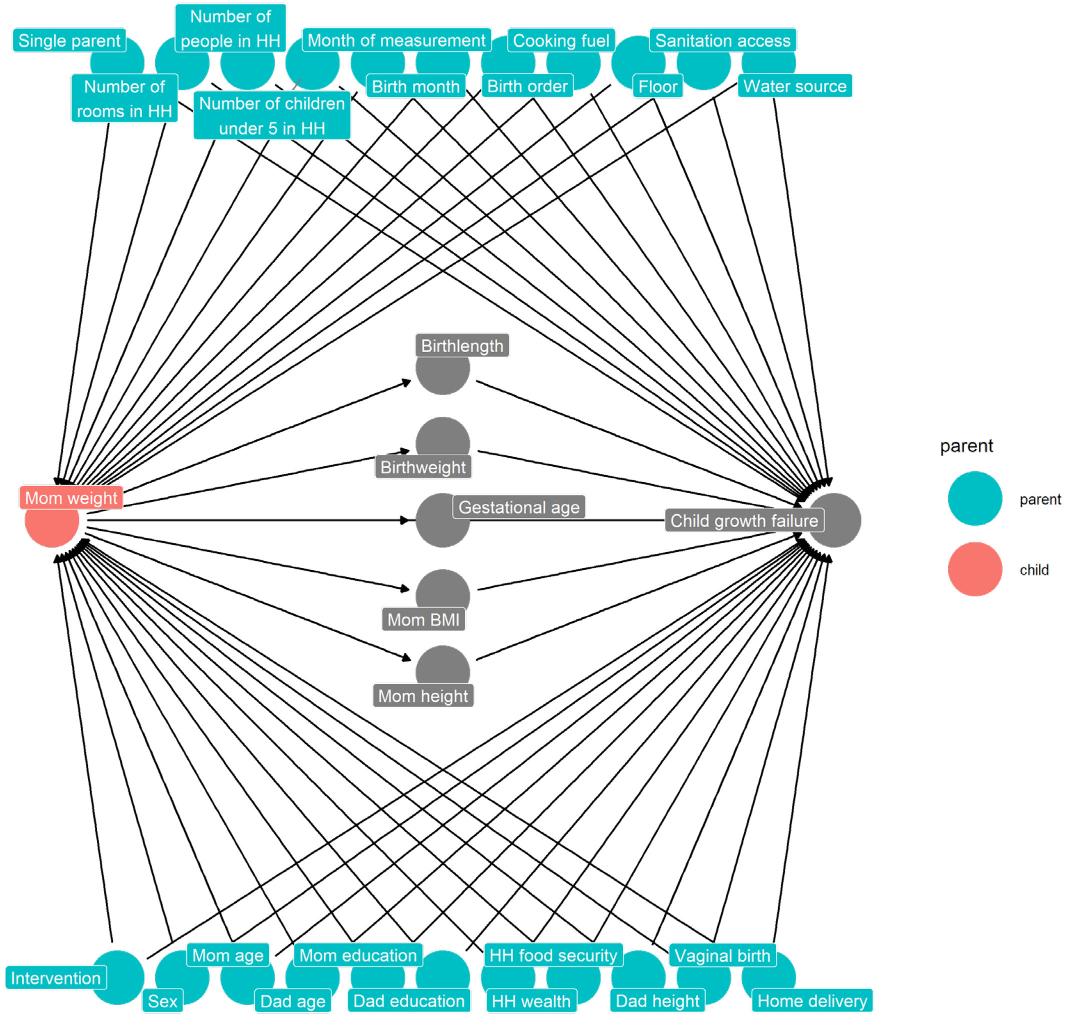


## 1.4 Table of Adjustment Covariates

| Exposure | Potential adjustment covariates |
|---|---|
| # of children <5 in HH | Treatment arm , Mother's age , Father's age , Mother's education , Father's education , HH wealth , HH food security , Mother's height , Mother's weight , BMI , Father's height , Single parent , # of rooms in HH , Birth order , Treats drinking water , Clean cooking fuel usage, Improved floor , Improved sanitation , Safe water source |
| # of people in HH | Treatment arm , Mother's age , Father's age , Mother's education , Father's education , HH wealth , HH food security , Mother's height , Mother's weight , BMI , Father's height , Single parent , # of rooms in HH , Birth order , Treats drinking water , Clean cooking fuel usage, Improved floor , Improved sanitation , Safe water source |

| Exposure | Potential adjustment covariates |
|---|---|
| # of rooms in HH | Treatment arm , Child sex , Mother's age , Father's age , Mother's education , Father's education , HH wealth , HH food security , Mother's height , Mother's weight , BMI , Father's height , Single parent , # of people in HH , # of children <5 in HH , Birth order , Treats drinking water , Clean cooking fuel usage, Improved floor , Improved sanitation , Safe water source |
| Any wasting <6 mo. | Treatment arm , Child sex , Mother's age , Father's age , Mother's education , Father's education , HH wealth , HH food security , Mother's height , Mother's weight , BMI , Father's height , Vaginal birth , Child delivered at home , Single parent , # of rooms in HH , # of people in HH , # of children <5 in HH , Month of measurement , Month of Birth , Birth order , Treats drinking water , Clean cooking fuel usage, Improved floor , Improved sanitation , Safe water source |
| Birth length (cm) | Treatment arm , Child sex , Mother's age , Father's age , Mother's education , Father's education , HH wealth , HH food security , Mother's height , Mother's weight , BMI , Father's height , Vaginal birth , Child delivered at home , Single parent , # of rooms in HH , # of people in HH , # of children <5 in HH , Month of Birth , Birth order , Treats drinking water , Clean cooking fuel usage, Improved floor , Improved sanitation , Safe water source |
| Birth order | Treatment arm , Mother's age , Father's age , Mother's education , Father's education , HH wealth , HH food security , Mother's height , Mother's weight , BMI , Father's height , Vaginal birth , Child delivered at home , Single parent , # of rooms in HH , Treats drinking water , Clean cooking fuel usage, Improved floor , Improved sanitation , Safe water source |
| Birthweight (kg) | Treatment arm , Child sex , Mother's age , Father's age , Mother's education , Father's education , HH wealth , HH food security , Mother's height , Mother's weight , BMI , Father's height , Vaginal birth , Child delivered at home , Single parent , # of rooms in HH , # of people in HH , # of children <5 in HH , Month of Birth , Birth order , Treats drinking water , Clean cooking fuel usage, Improved floor , Improved sanitation , Safe water source |
| Breastfed hour after birth | Treatment arm , Child sex , Mother's age , Father's age , Mother's education , Father's education , HH wealth , HH food security , Mother's height , Mother's weight , BMI , Father's height , Vaginal birth , Child delivered at home , Gestational age at birth, Birthweight (kg) , Birth length (cm) , Single parent , # of rooms in HH , # of people in HH , # of children <5 in HH , Month of Birth , Birth order , Treats drinking water , Clean cooking fuel usage, Improved floor , Improved sanitation , Safe water source |
| Child delivered at home | Treatment arm , Child sex , Mother's age , Father's age , Mother's education , Father's education , HH wealth , HH food security , Mother's height , Mother's weight , BMI , Father's height , Single parent , # of rooms in HH , # of people in HH , # of children <5 in HH , Month of Birth , Birth order , Treats |

| Exposure | Potential adjustment covariates |
|---|---|
| | drinking water , Clean cooking fuel usage, Improved floor , Improved sanitation , Safe water source |
| Clean cooking fuel usage | Treatment arm , Child sex , Mother's age , Father's age , Mother's education , Father's education , HH wealth , HH food security , Mother's height , Mother's weight , BMI , Father's height , Single parent , # of rooms in HH , # of people in HH , # of children <5 in HH, Birth order , Treats drinking water , Improved floor , Improved sanitation , Safe water source |
| Diarrhea <24 mo. (% days) | Treatment arm , Child sex , Mother's age , Father's age , Mother's education , Father's education , HH wealth , HH food security , Mother's height , Mother's weight , BMI , Father's height , Vaginal birth , Child delivered at home , Gestational age at birth, Birthweight (kg) , Birth length (cm) , Single parent , # of rooms in HH , # of people in HH , # of children <5 in HH , Month of measurement , Month of Birth , Birth order , Treats drinking water , Clean cooking fuel usage, Improved floor , Improved sanitation , Safe water source |
| Diarrhea <6 mo. (% days) | Treatment arm , Child sex , Mother's age , Father's age , Mother's education , Father's education , HH wealth , HH food security , Mother's height , Mother's weight , BMI , Father's height , Vaginal birth , Child delivered at home , Gestational age at birth, Birthweight (kg) , Birth length (cm) , Single parent , # of rooms in HH , # of people in HH , # of children <5 in HH , Month of measurement , Month of Birth , Birth order , Treats drinking water , Clean cooking fuel usage, Improved floor , Improved sanitation , Safe water source |
| Excl/Pred breastfed <6mo. | Treatment arm , Child sex , Mother's age , Father's age , Mother's education , Father's education , HH wealth , HH food security , Mother's height , Mother's weight , BMI , Father's height , Vaginal birth , Child delivered at home , Gestational age at birth, Birthweight (kg) , Birth length (cm) , Single parent , # of rooms in HH , # of people in HH , # of children <5 in HH , Month of measurement , Month of Birth , Birth order , Treats drinking water , Clean cooking fuel usage, Improved floor , Improved sanitation , Safe water source |
| Father's age | Treatment arm , Mother's age , Mother's education , Father's education , HH wealth , HH food security , Mother's height , Mother's weight , BMI , Father's height , Single parent , # of rooms in HH , # of people in HH , # of children <5 in HH , Month of Birth , Treats drinking water , Clean cooking fuel usage, Improved floor , Improved sanitation , Safe water source |
| Father's education | Treatment arm , Mother's age , Father's age , Mother's education , HH wealth , Mother's height , Mother's weight , BMI , Father's height , Gestational age at birth, Birthweight (kg) , Birth length (cm) , Single parent , # of rooms in HH , # of people in HH , # of children <5 in HH , Treats drinking water , Clean cooking fuel usage, Improved floor , Improved sanitation , Safe water source |
| Father's height | Treatment arm , Mother's age , Father's age , Mother's education , Father's education , HH wealth , HH food security , Mother's height , Mother's weight |

| Exposure | Potential adjustment covariates |
|---|---|
| | , BMI , Single parent , # of rooms in HH , Treats drinking water , Clean cooking fuel usage, Improved floor , Improved sanitation , Safe water source |
| Gestational age at birth | Treatment arm , Child sex , Mother's age , Father's age , Mother's education , Father's education , HH wealth , HH food security , Mother's height , Mother's weight , BMI , Father's height , Single parent , # of rooms in HH , # of people in HH , # of children <5 in HH , Month of Birth , Birth order , Treats drinking water , Clean cooking fuel usage, Improved floor , Improved sanitation , Safe water source |
| HH food security | Treatment arm , Child sex , Mother's age , Father's age , Mother's education , Father's education , HH wealth , Vaginal birth , Child delivered at home , Single parent , # of rooms in HH , # of people in HH , # of children <5 in HH , Month of Birth , Birth order , Treats drinking water , Clean cooking fuel usage, Improved floor , Improved sanitation , Safe water source |
| HH wealth | Treatment arm , Child sex , Mother's age , Father's age , Mother's education , Father's education , Gestational age at birth, Birthweight (kg) , Birth length (cm) , Single parent , # of people in HH , # of children <5 in HH , Birth order |
| Improved floor | Treatment arm , Child sex , Mother's age , Father's age , Mother's education , Father's education , HH wealth , HH food security , Mother's height , Mother's weight , BMI , Father's height , Single parent , # of rooms in HH , # of people in HH , # of children <5 in HH , Birth order , Treats drinking water , Clean cooking fuel usage, Improved sanitation , Safe water source |
| Improved sanitation | Treatment arm , Child sex , Mother's age , Father's age , Mother's education , Father's education , HH wealth , HH food security , Mother's height , Mother's weight , BMI , Father's height , Single parent , # of rooms in HH , # of people in HH , # of children <5 in HH , Birth order , Treats drinking water , Clean cooking fuel usage, Improved floor , Safe water source |
| Mother's age | Treatment arm , Father's age , Mother's education , Father's education , HH wealth , HH food security , Mother's height , Mother's weight , BMI , Father's height , Single parent , # of rooms in HH , # of people in HH , # of children <5 in HH , Treats drinking water , Clean cooking fuel usage, Improved floor , Improved sanitation , Safe water source |
| Mother's BMI | Treatment arm , Mother's age , Father's age , Mother's education , Father's education , HH wealth , HH food security , Father's height , Single parent , # of rooms in HH , # of people in HH , # of children <5 in HH , Month of Birth , Birth order , Treats drinking water , Clean cooking fuel usage, Improved floor , Improved sanitation , Safe water source |
| Mother's education | Treatment arm , Mother's age , Father's age , Father's education , HH wealth , Mother's height , Mother's weight , BMI , Father's height , Gestational age at birth, Birthweight (kg) , Birth length (cm) , Single parent , # of rooms in HH |

| Exposure | Potential adjustment covariates |
|---|---|
| | , # of people in HH , # of children <5 in HH , Treats drinking water , Clean cooking fuel usage, Improved floor , Improved sanitation , Safe water source |
| Mother's height | Treatment arm , Mother's age , Father's age , Mother's education , Father's education , HH wealth , HH food security , Father's height , Single parent , # of rooms in HH , Treats drinking water , Clean cooking fuel usage, Improved floor , Improved sanitation , Safe water source |
| Mother's weight | Treatment arm , Mother's age , Father's age , Mother's education , Father's education , HH wealth , HH food security , Father's height , Single parent , # of rooms in HH , # of people in HH , # of children <5 in HH , Month of Birth , Birth order , Treats drinking water , Clean cooking fuel usage, Improved floor , Improved sanitation , Safe water source |
| Persistent wasting <6 mo. | Treatment arm , Child sex , Mother's age , Father's age , Mother's education , Father's education , HH wealth , HH food security , Mother's height , Mother's weight , BMI , Father's height , Vaginal birth , Child delivered at home , Single parent , # of rooms in HH , # of people in HH , # of children <5 in HH , Month of measurement , Month of Birth , Birth order , Treats drinking water , Clean cooking fuel usage, Improved floor , Improved sanitation , Safe water source |
| Safe water source | Treatment arm , Child sex , Mother's age , Father's age , Mother's education , Father's education , HH wealth , HH food security , Mother's height , Mother's weight , BMI , Father's height , Single parent , # of rooms in HH , # of people in HH , # of children <5 in HH , Birth order , Treats drinking water , Clean cooking fuel usage, Improved floor , Improved sanitation |
| Single parent | Treatment arm , Mother's age , Father's age , Mother's education , Father's education , HH wealth , HH food security , Mother's height , Mother's weight , BMI , Father's height , # of rooms in HH , # of people in HH , # of children <5 in HH , Treats drinking water , Clean cooking fuel usage, Improved floor , Improved sanitation , Safe water source |
| Small for gestational age | Treatment arm , Child sex , Mother's age , Father's age , Mother's education , Father's education , HH wealth , HH food security , Mother's height , Mother's weight , BMI , Father's height , Single parent , # of rooms in HH , # of people in HH , # of children <5 in HH , Month of Birth , Birth order , Treats drinking water , Clean cooking fuel usage, Improved floor , Improved sanitation , Safe water source |
| Treats drinking water | Treatment arm , Child sex , Mother's age , Father's age , Mother's education , Father's education , HH wealth , HH food security , Mother's height , Mother's weight , BMI , Father's height , Single parent , # of rooms in HH , # of people in HH , # of children <5 in HH , Month of Birth , Birth order , Clean cooking fuel usage, Improved floor , Improved sanitation , Safe water source |

# Supplementary Note 2. Complete-Case Sensitivity Analyses

## 2.1 Heatmap of covariate missingness by study

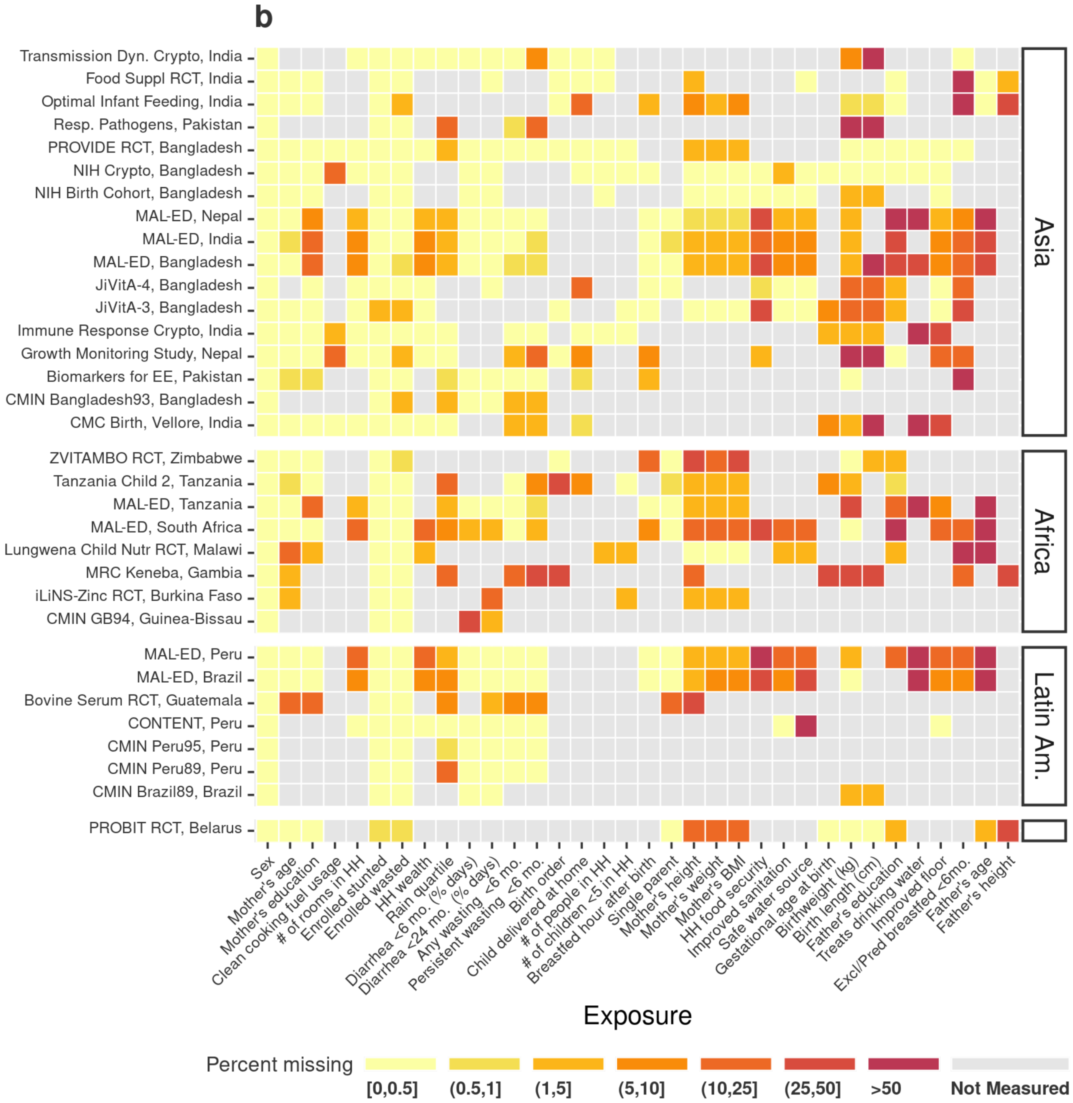


## 2.2 Sensitivity analysis to complete case versus median/mode imputed analyses

Plots comparing population intervention effect estimates (the estimates presented in Figures 2 and 3) compared to estimates only using complete-case data (excluding any observations missing covariate information).

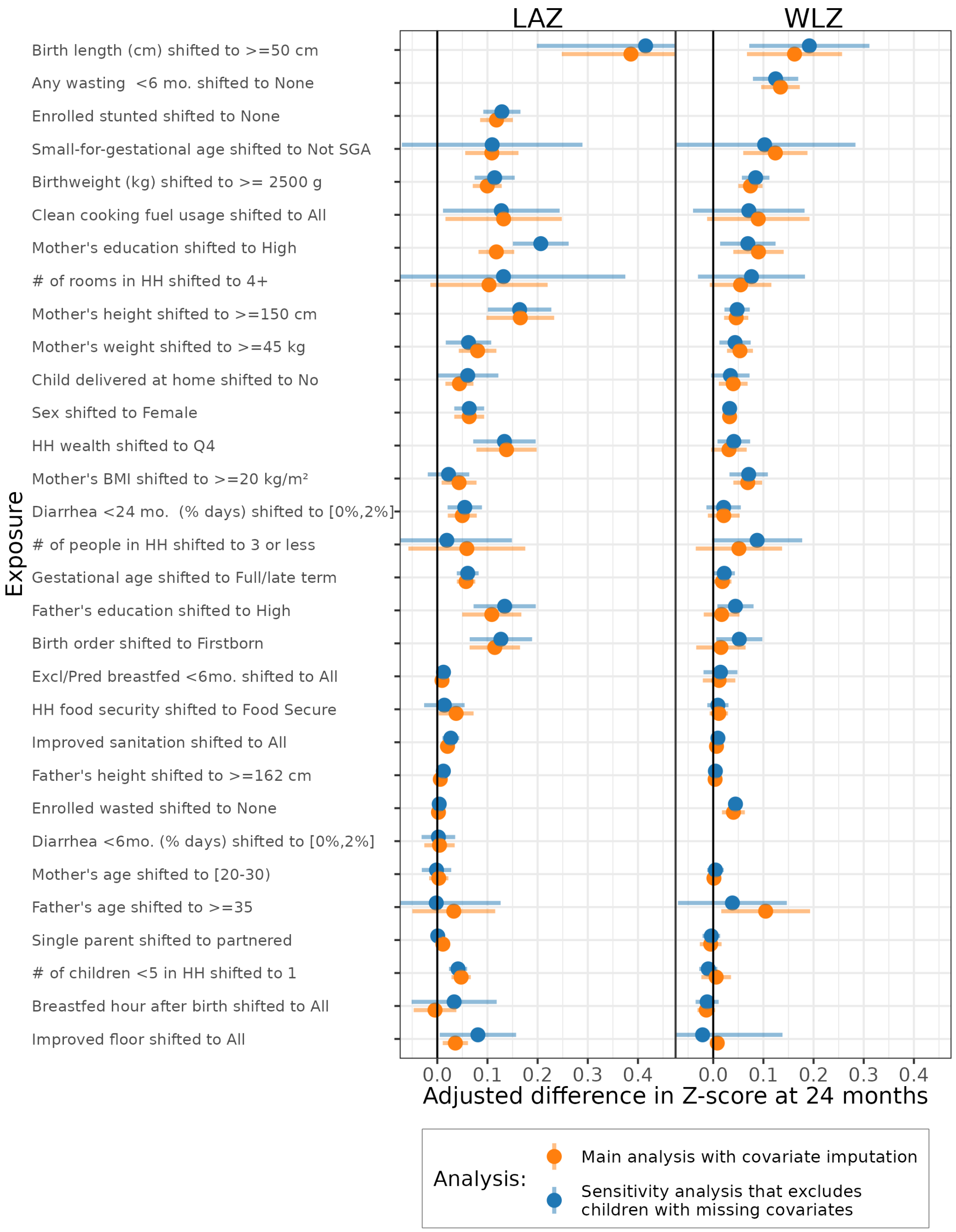


The median absolute difference in pooled Z-scores was 0.009 and the mean absolute difference was 0.022.

# Supplementary Note 3. Sensitivity analysis using fixed effects models

## 3.1 Overview

In the primary manuscript, we compared estimates pooled across studies using random effects models, which are more conservative in the presence of heterogeneity across studies. Here, we present estimates pooled using fixed effects. Inferences about estimates from fixed effects models are restricted to only the included studies, while random effects estimates assume included studies are sampled from a distribution of hypothetical studies. Fixed effect estimates had narrow confidence intervals, and therefore more risk factors had statistically significant PIEs. The fixed effect estimates are weighted towards results from larger studies, so are more heavily influenced by the large Jivita-3, Probit, and ZVITAMBO trials. But in general, the key exposures identified from the random-effects estimates also had the strongest associations when pooled using fixed-effect estimates.

## 3.2 Primary manuscript figures recreated with estimates pooled using fixed effects

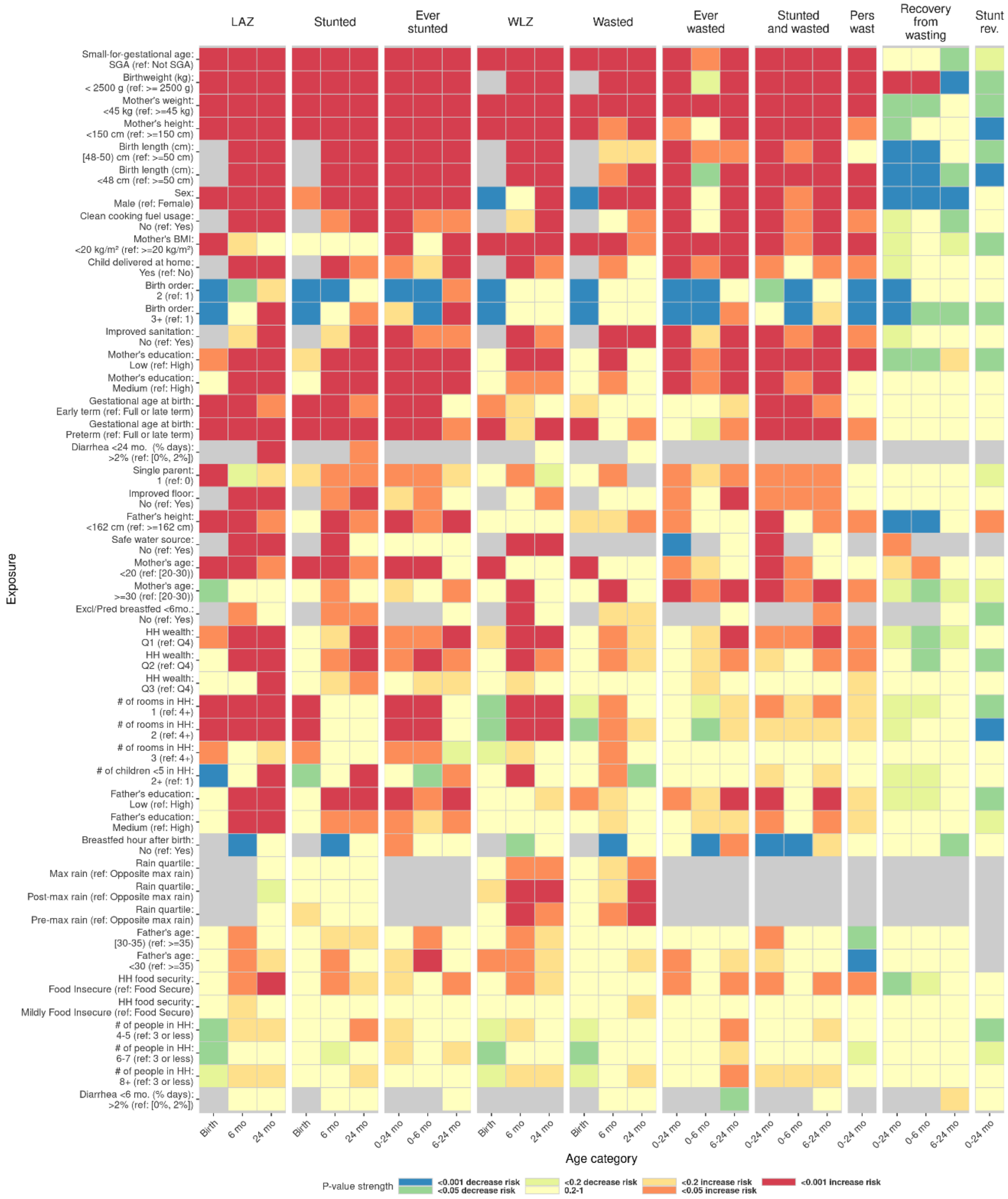


**Heatmap of significance and direction across exposure-outcome combinations of associations estimated using fixed effects.**

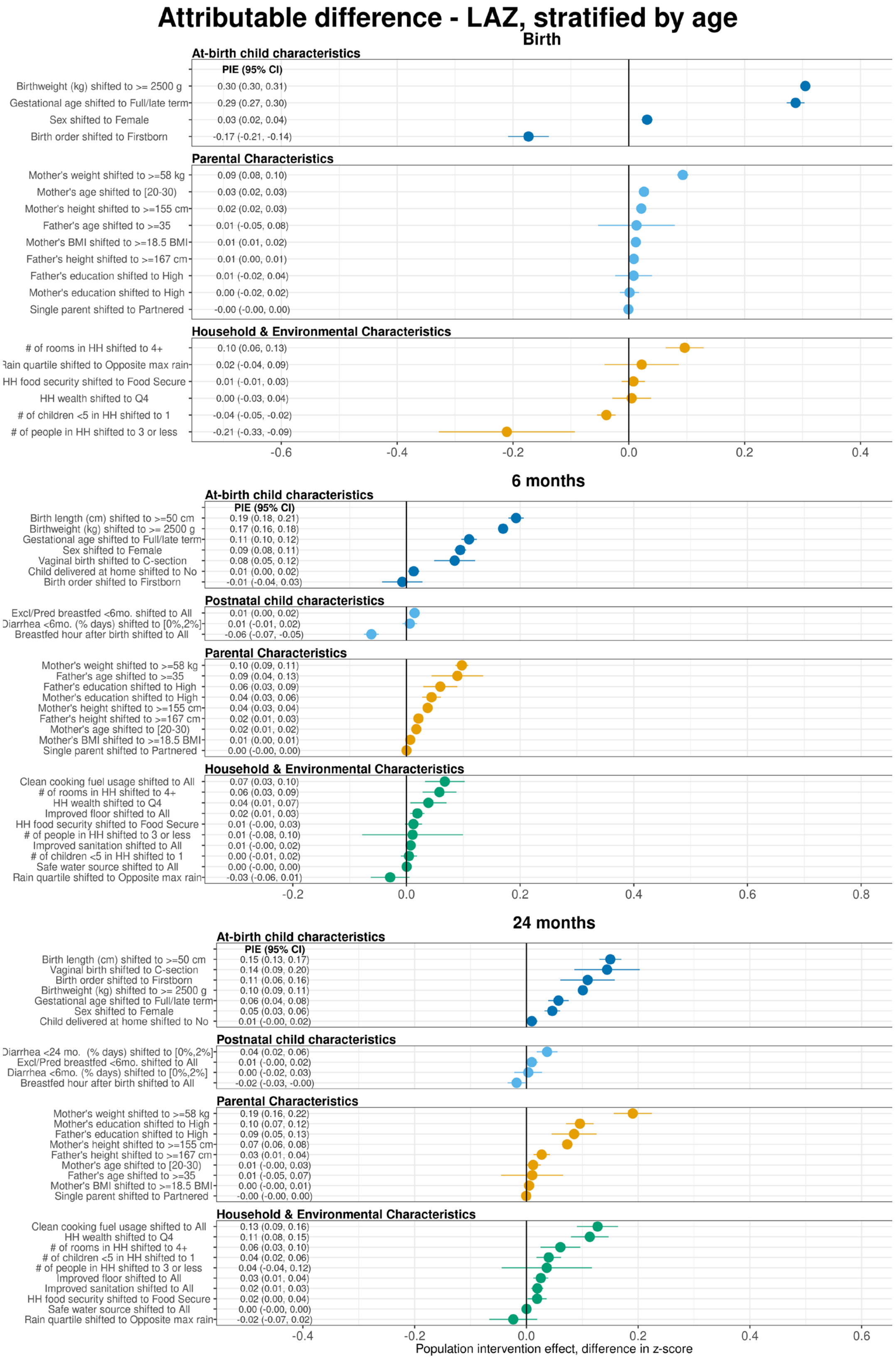


**Age-stratified population attributable differences in length-for-age Z-scores estimated using fixed effects.**

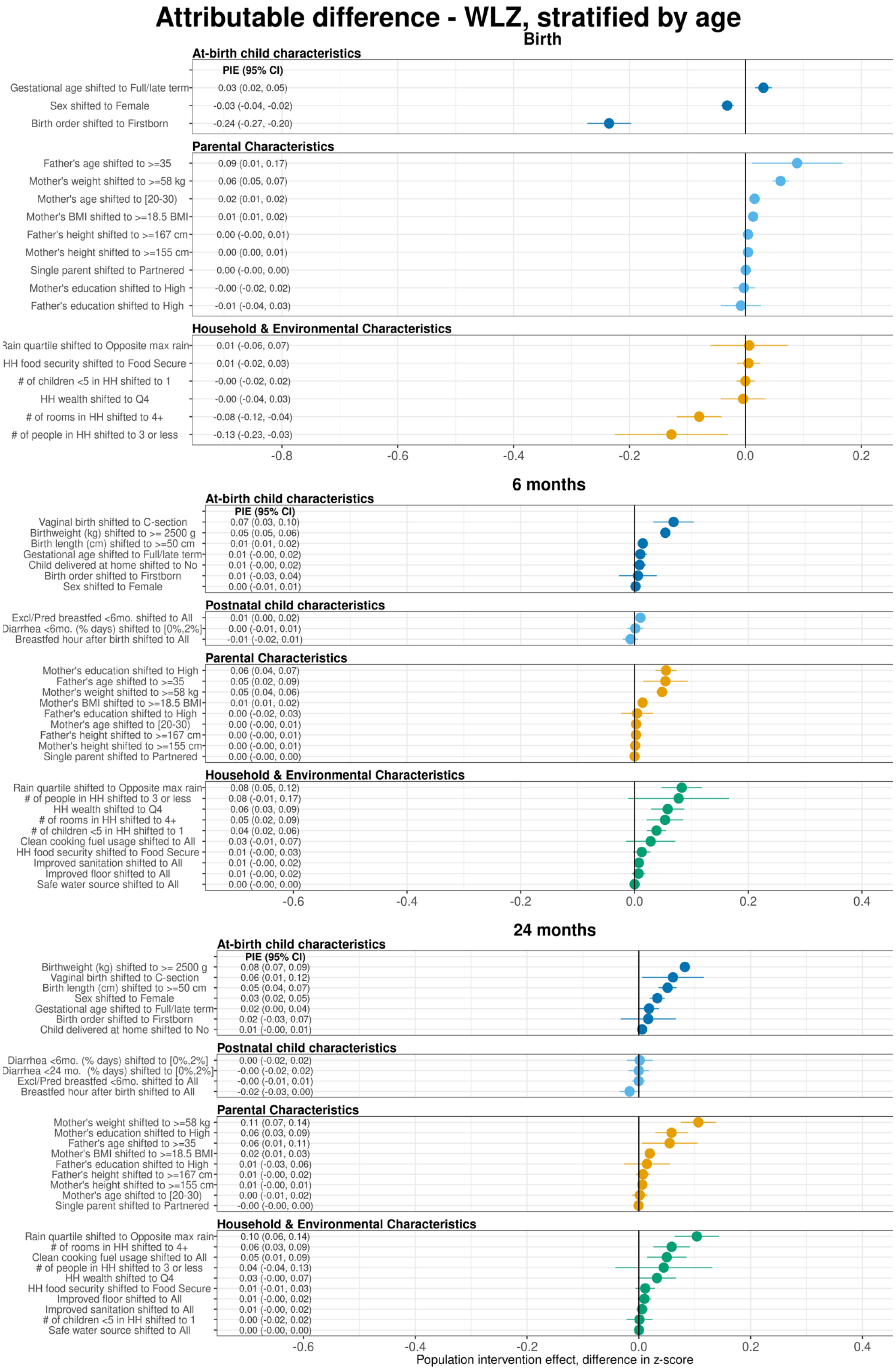


**Age-stratified population attributable differences in weight-for-length Z-scores estimated using fixed effects.**

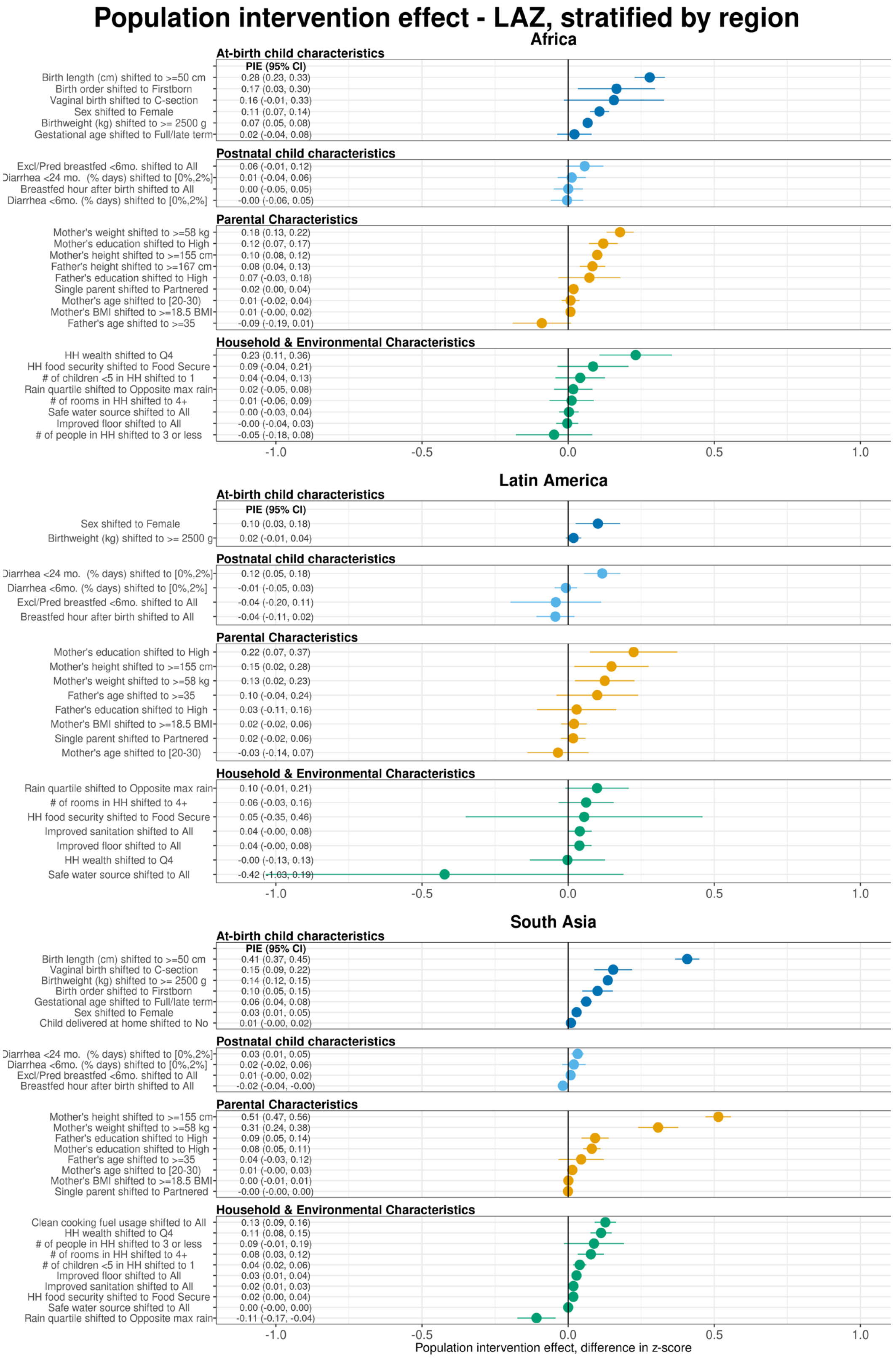


**Region-stratified population attributable differences in length-for-age Z-scores estimated using fixed effects.**

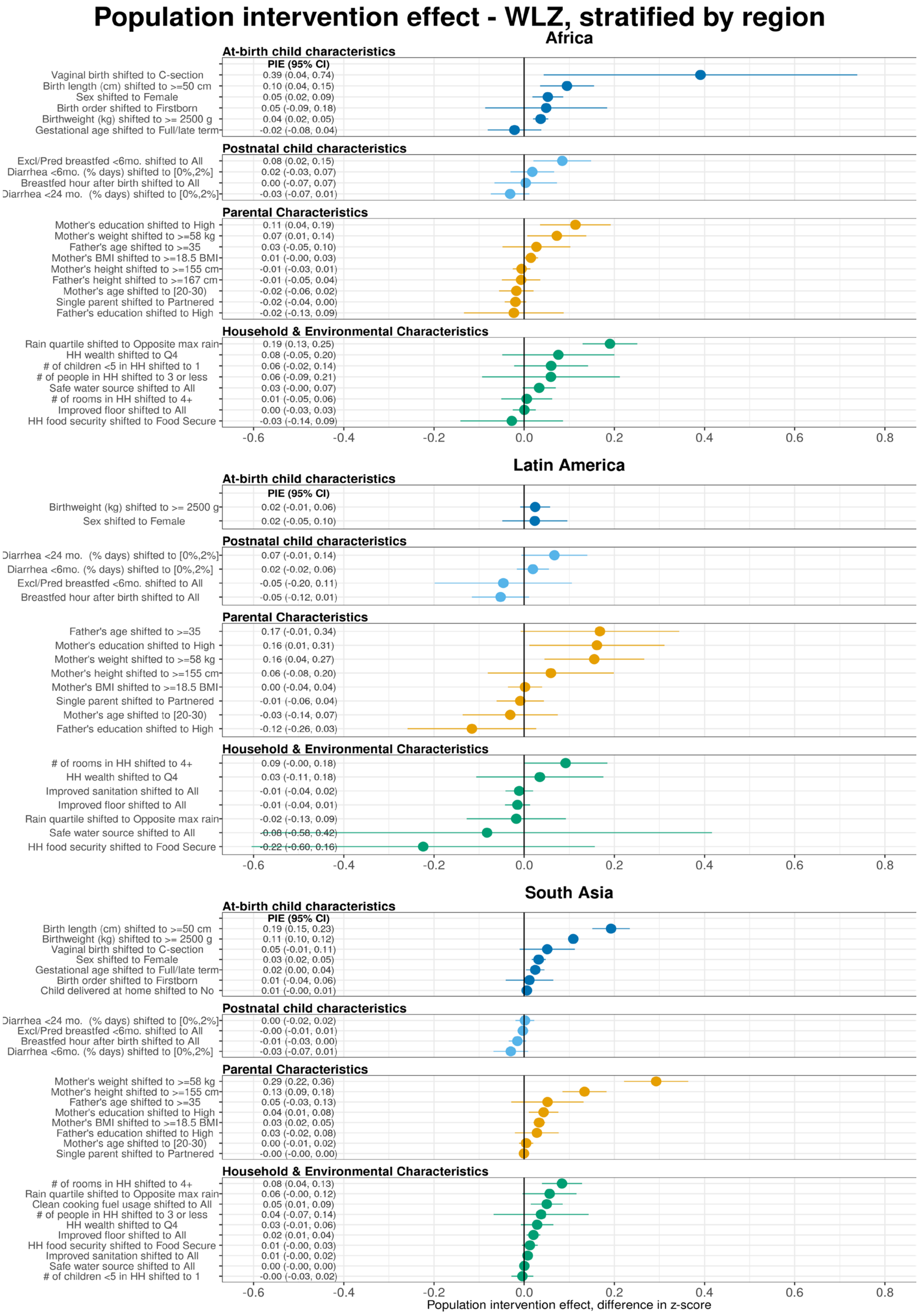


**Region-stratified population attributable differences in weight-for-length Z-scores estimated using fixed effects.**

## Supplementary Note 4. Comparison of Adjusted and Unadjusted Population Intervention Effects

Plots comparing covariate-adjusted population intervention effect estimates (the estimates presented in Figures 2 and 3) compared to estimates not adjusted for potential confounders laid out in Table 1.4.

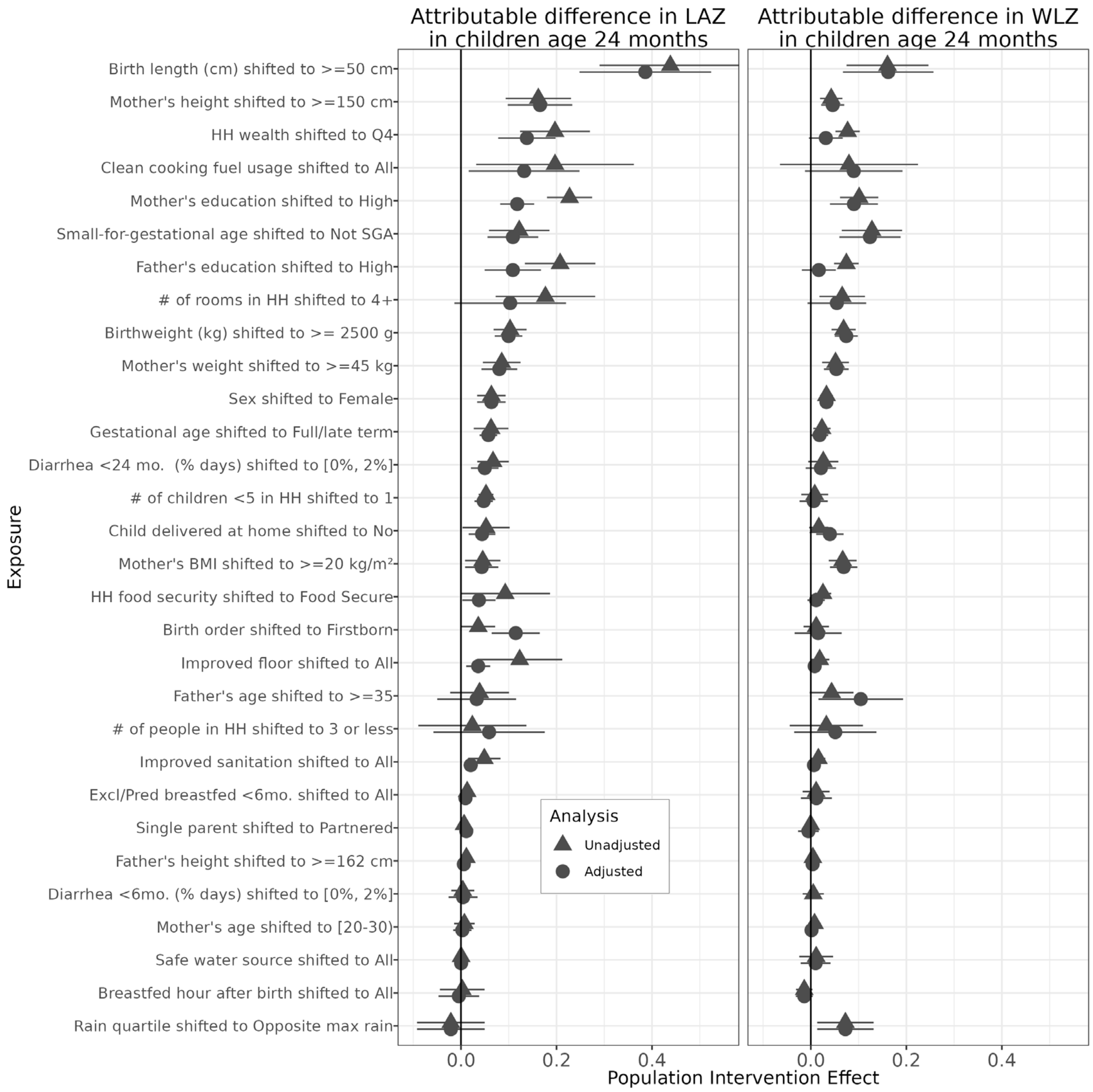


## Supplementary Note 5. Spline plots: all exposures

Spline plots of HAZ and WHZ over the first two years of children's lives, stratified by levels of risk factors. Plots were constructed using the same methods as for Figures 4a and 4b for all risk factors (details in the Methods section).

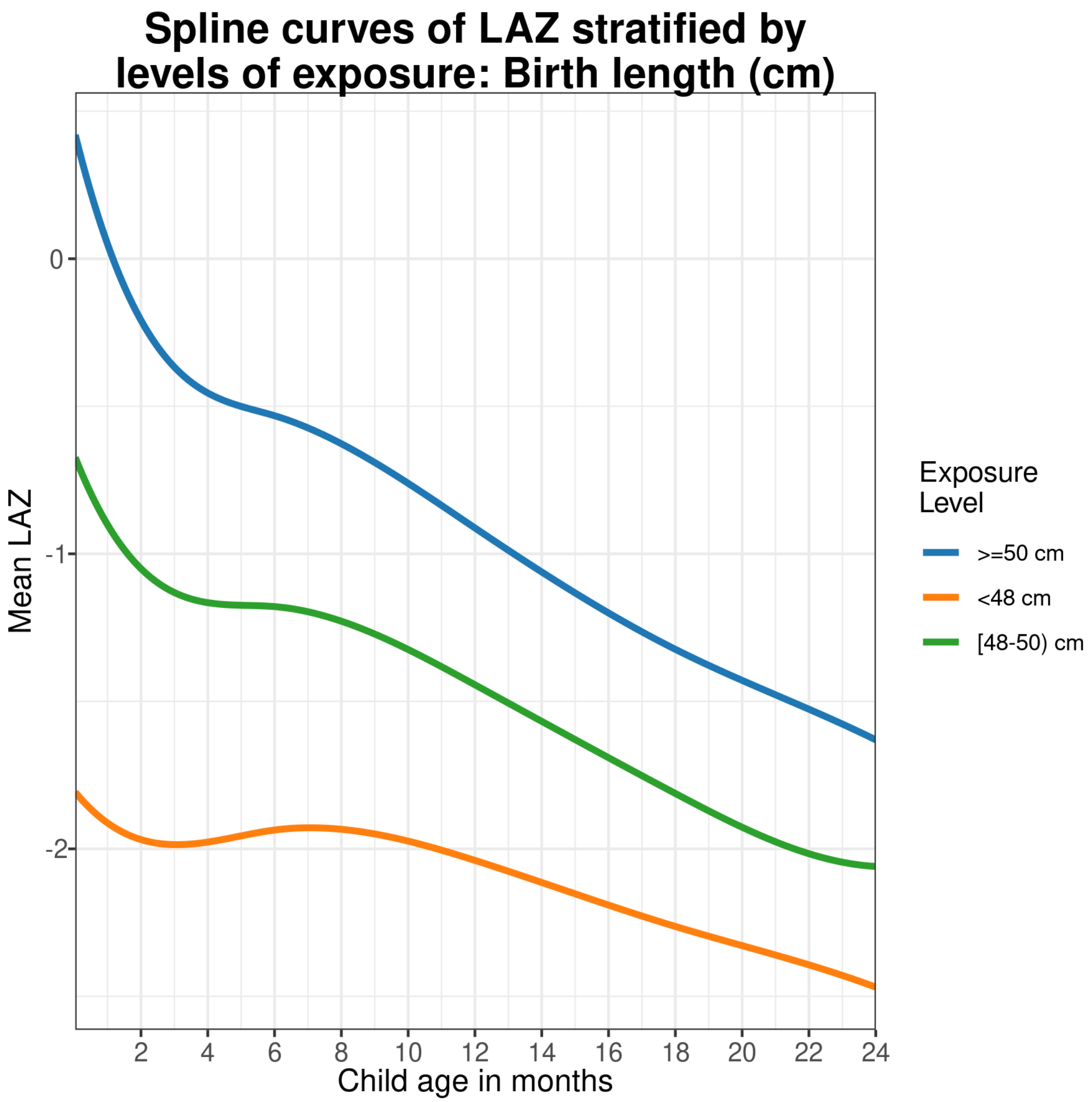

Spline curves of LAZ stratified by
levels of exposure: Birth length (cm)
0
-1
-2
Mean LAZ
2
4
6
8
10
12
14
16
18
20
22
24
Child age in months
Exposure
Level
>=50 cm
<48 cm
[48-50) cm

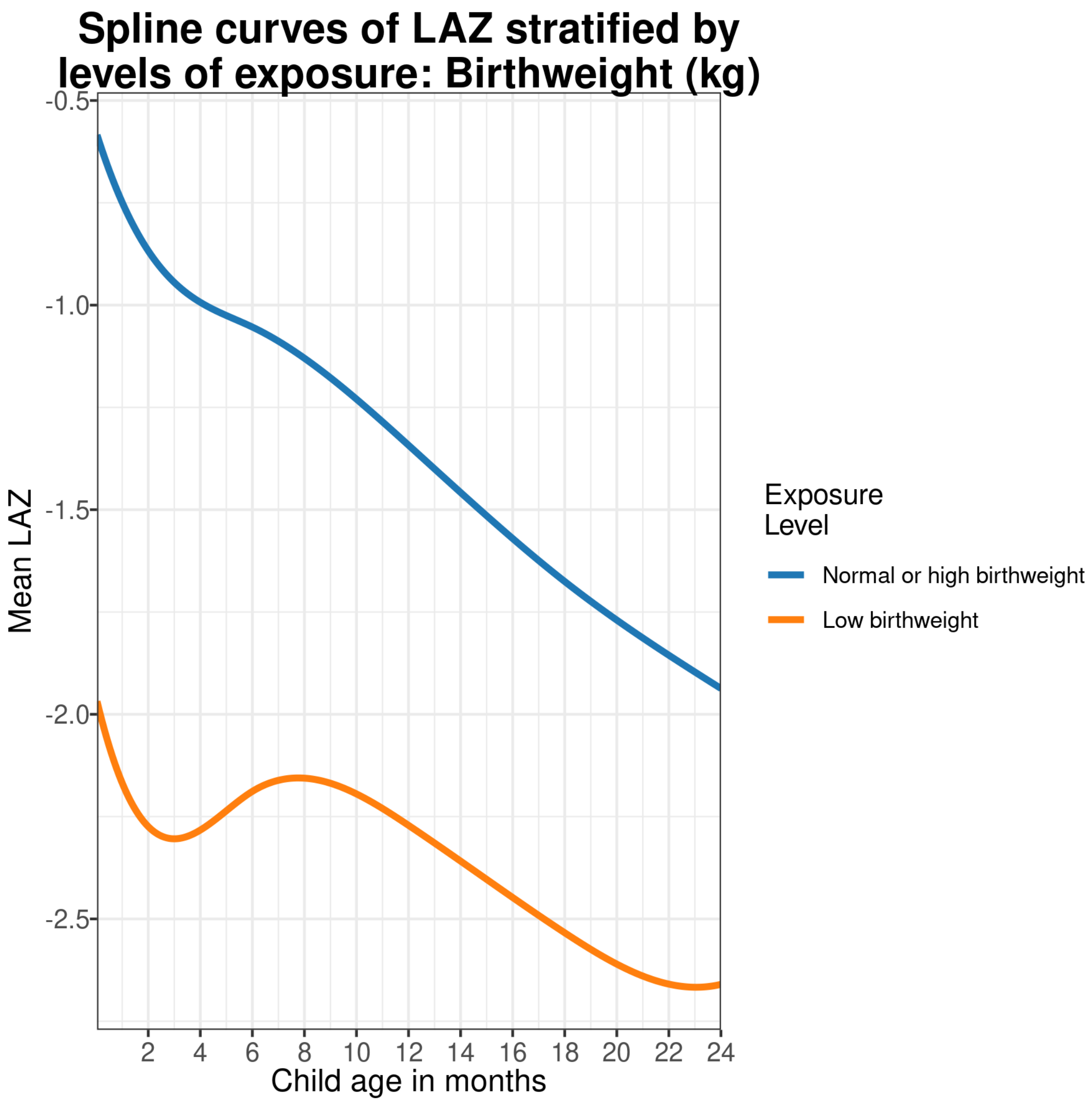

Spline curves of LAZ stratified by
levels of exposure: Birthweight (kg)
-0.5
-1.0
-1.5
-2.0
-2.5
Mean LAZ
2
4
6
8
10
12
14
16
18
20
22
24
Child age in months
Exposure
Level
Normal or high birthweight
Low birthweight

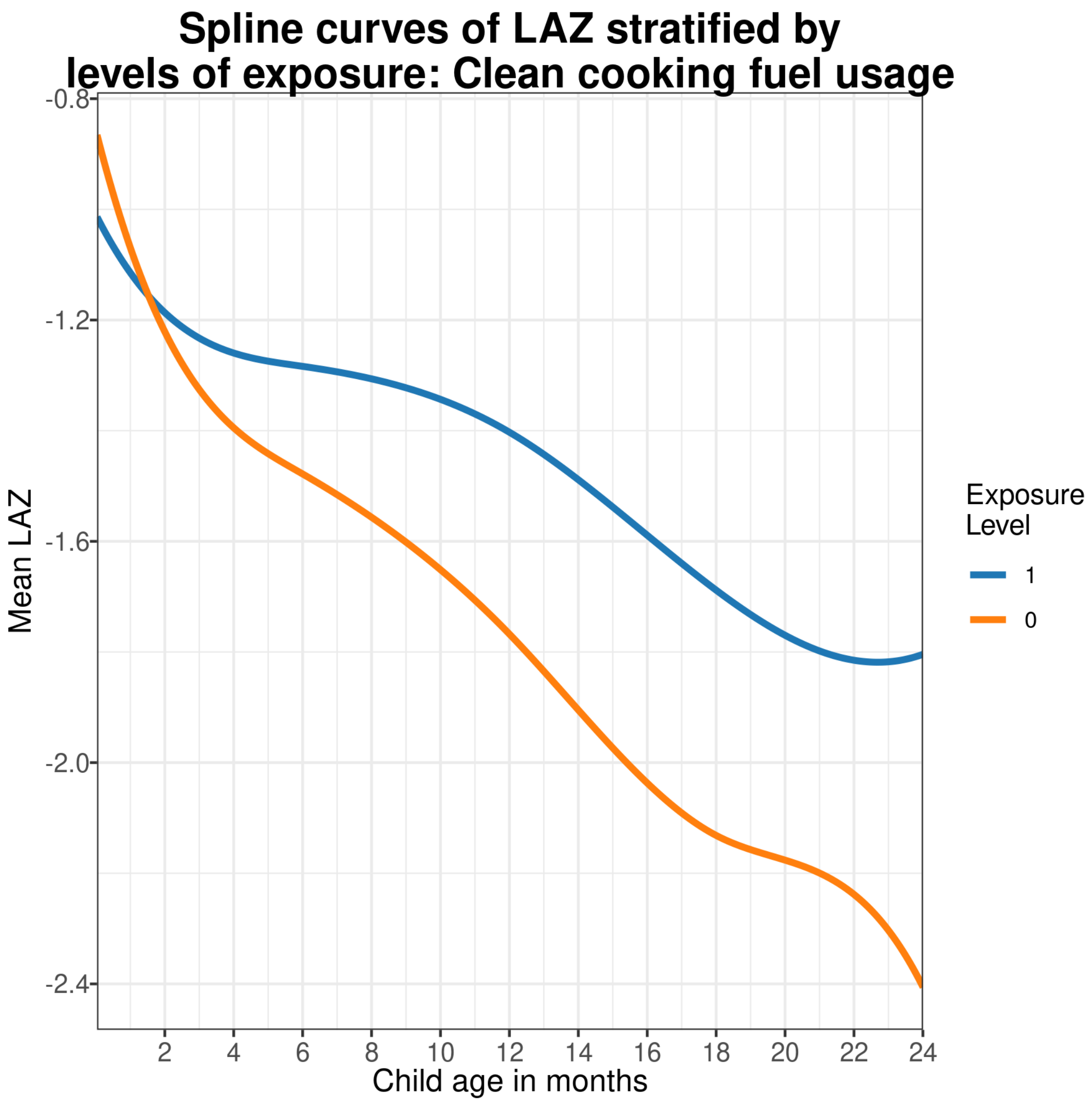

Spline curves of LAZ stratified by
levels of exposure: Clean cooking fuel usage
Mean LAZ
-0.8
-1.2
-1.6
-2.0
-2.4
2
4
6
8
10
12
14
16
18
20
22
24
Child age in months
Exposure Level
1
0

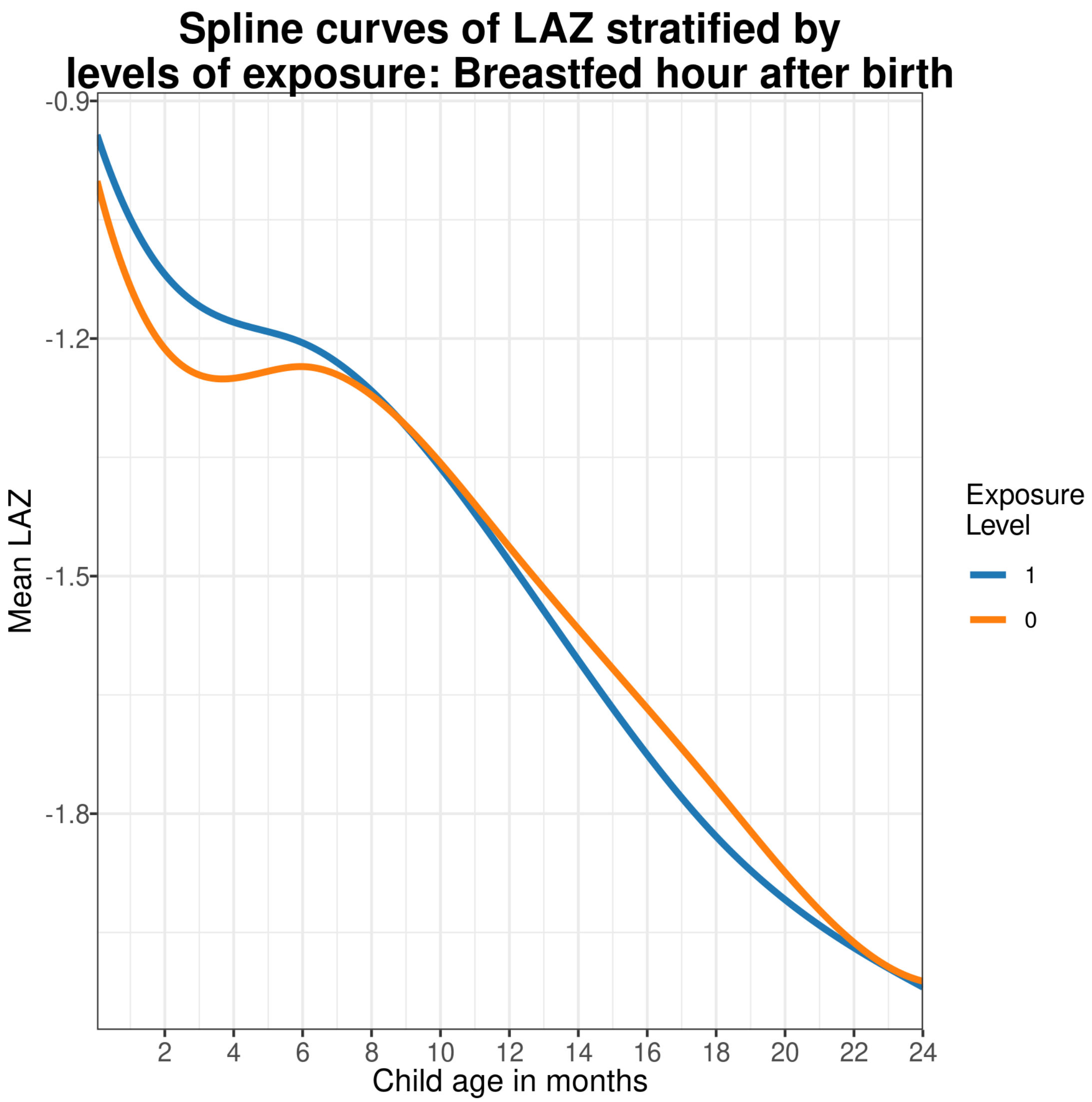
Spline curves of LAZ stratified by
levels of exposure: Breastfed hour after birth
-0.9
-1.2
-1.5
-1.8
Mean LAZ
2
4
6
8
10
12
14
16
18
20
22
24
Child age in months
Exposure
Level
1
0

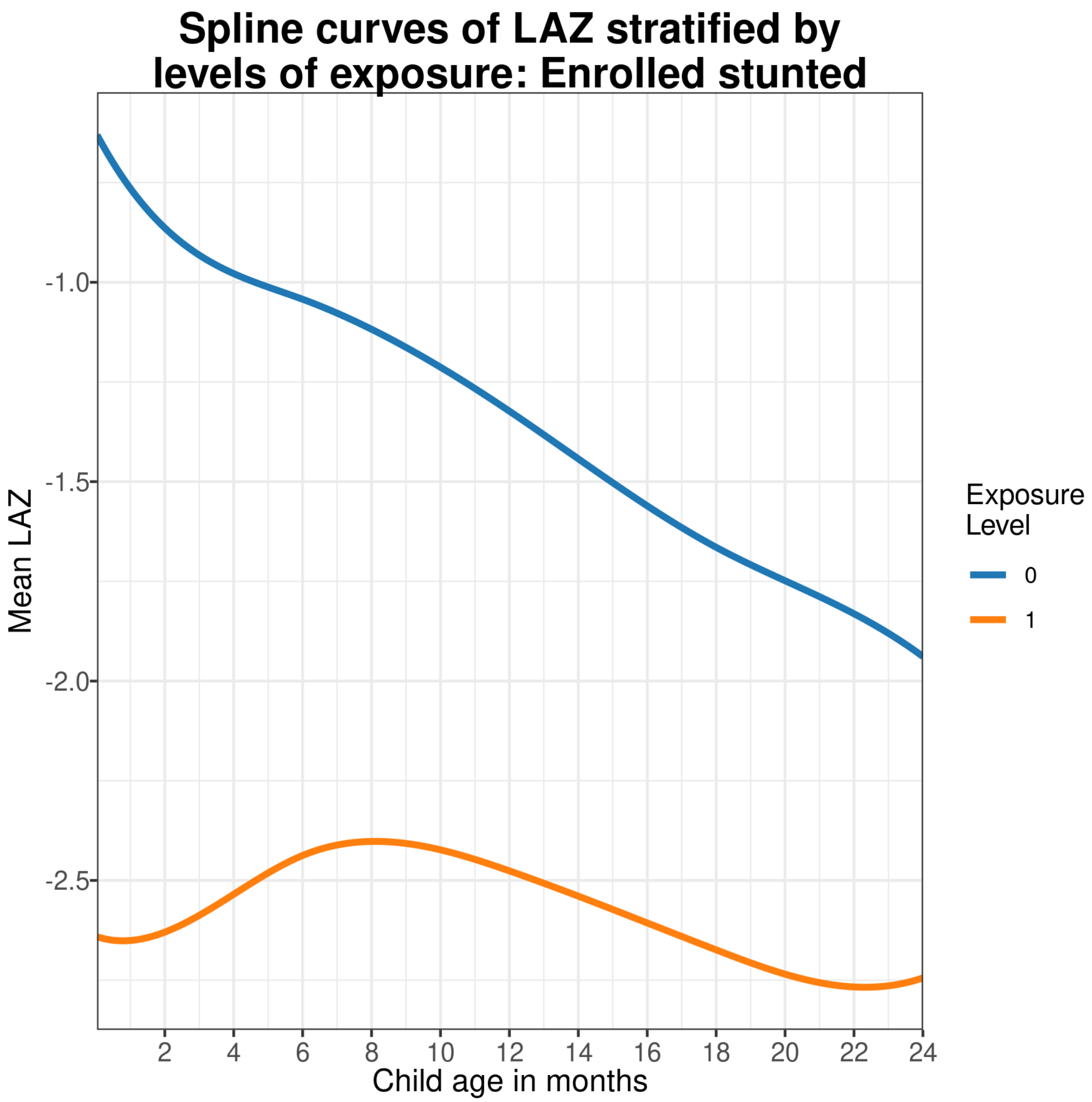
Spline curves of LAZ stratified by
levels of exposure: Enrolled stunted
Mean LAZ
-1.0
-1.5
-2.0
-2.5
2
4
6
8
10
12
14
16
18
20
22
24
Child age in months
Exposure
Level
0
1

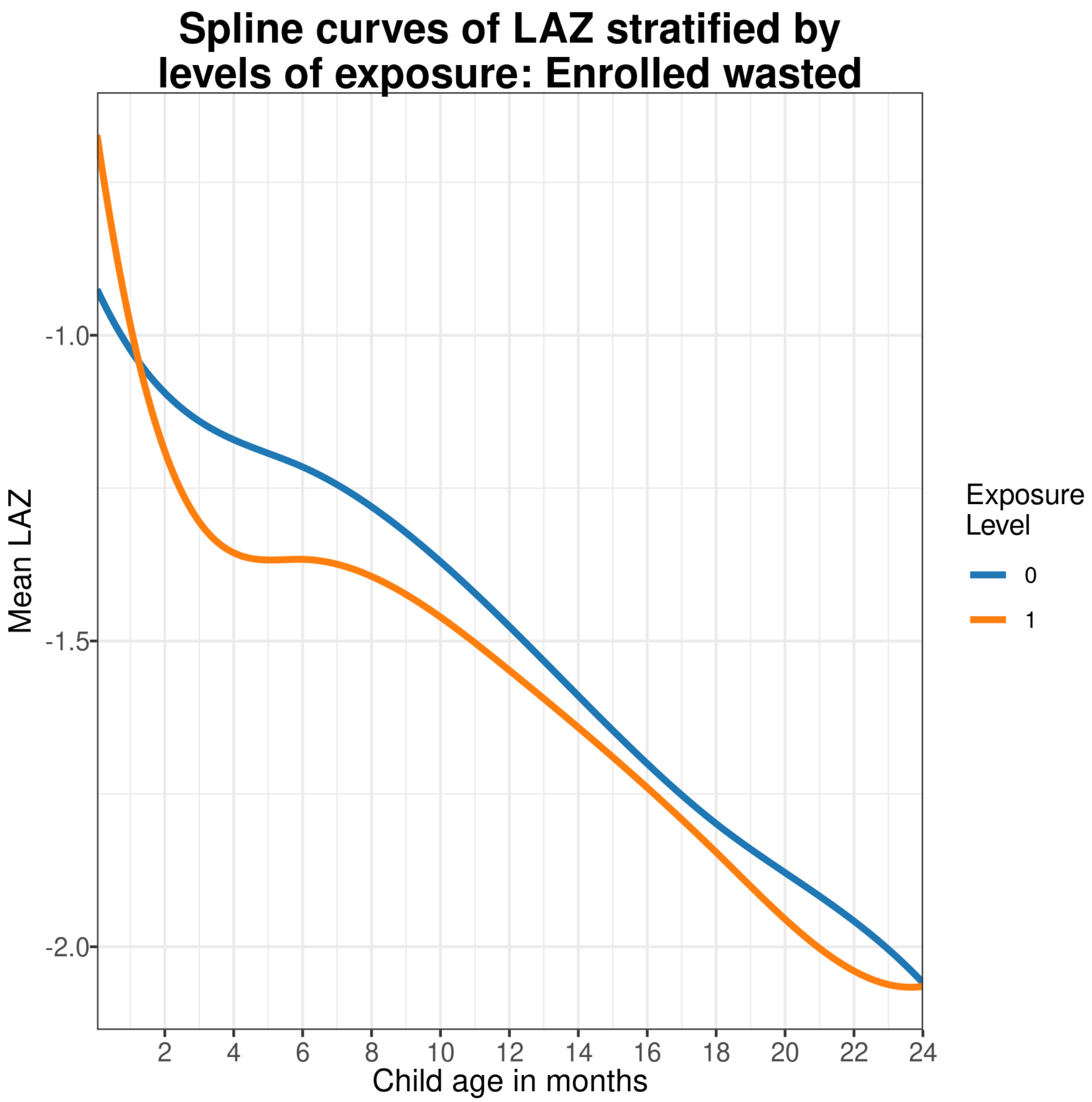

Spline curves of LAZ stratified by levels of exposure: Enrolled wasted
Mean LAZ
-1.0
-1.5
-2.0
2
4
6
8
10
12
14
16
18
20
22
24
Child age in months
Exposure Level
0
1

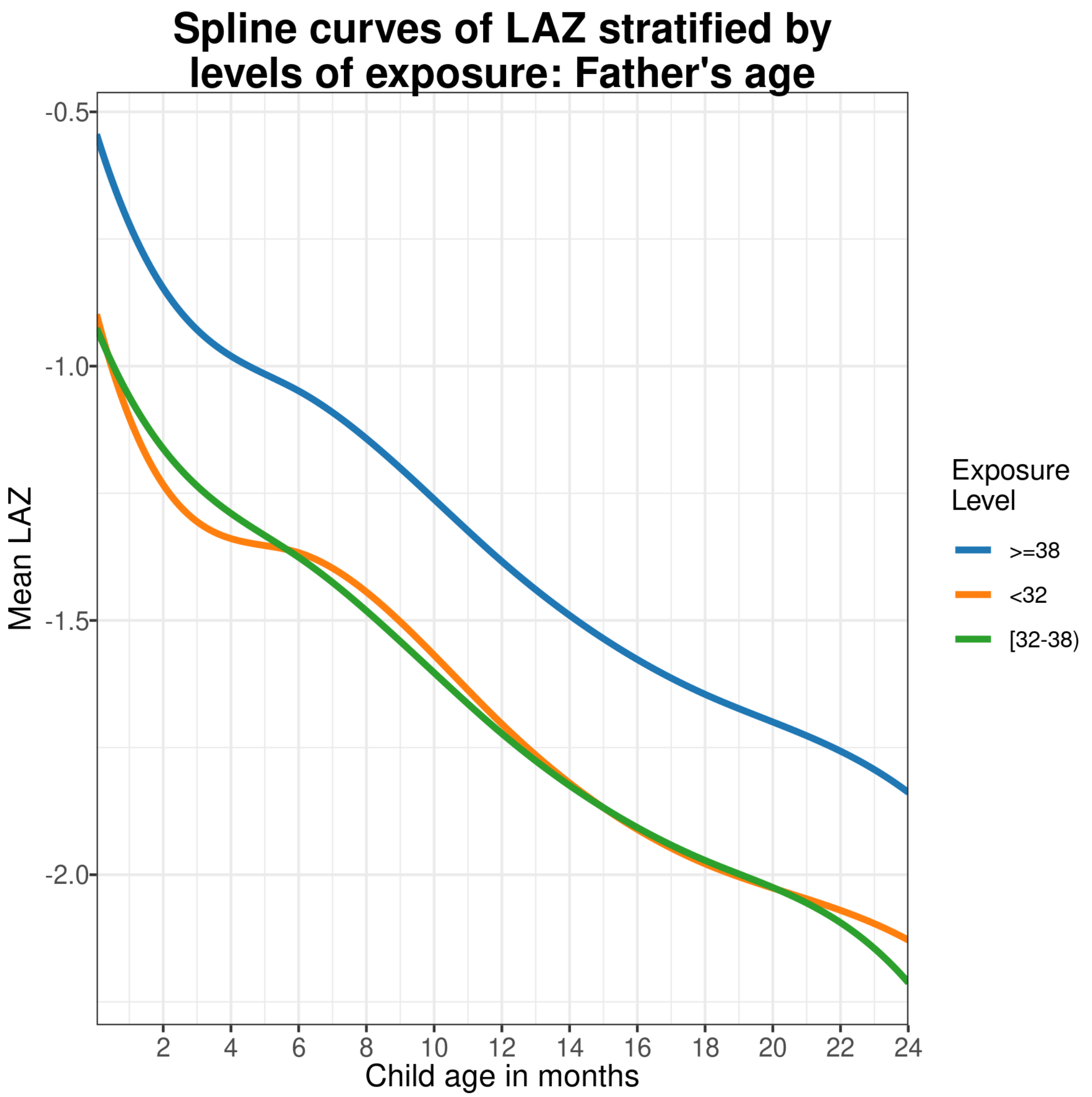
Spline curves of LAZ stratified by
levels of exposure: Father's age
Mean LAZ
-0.5
-1.0
-1.5
-2.0
2
4
6
8
10
12
14
16
18
20
22
24
Child age in months
Exposure
Level
>=38
<32
[32-38)

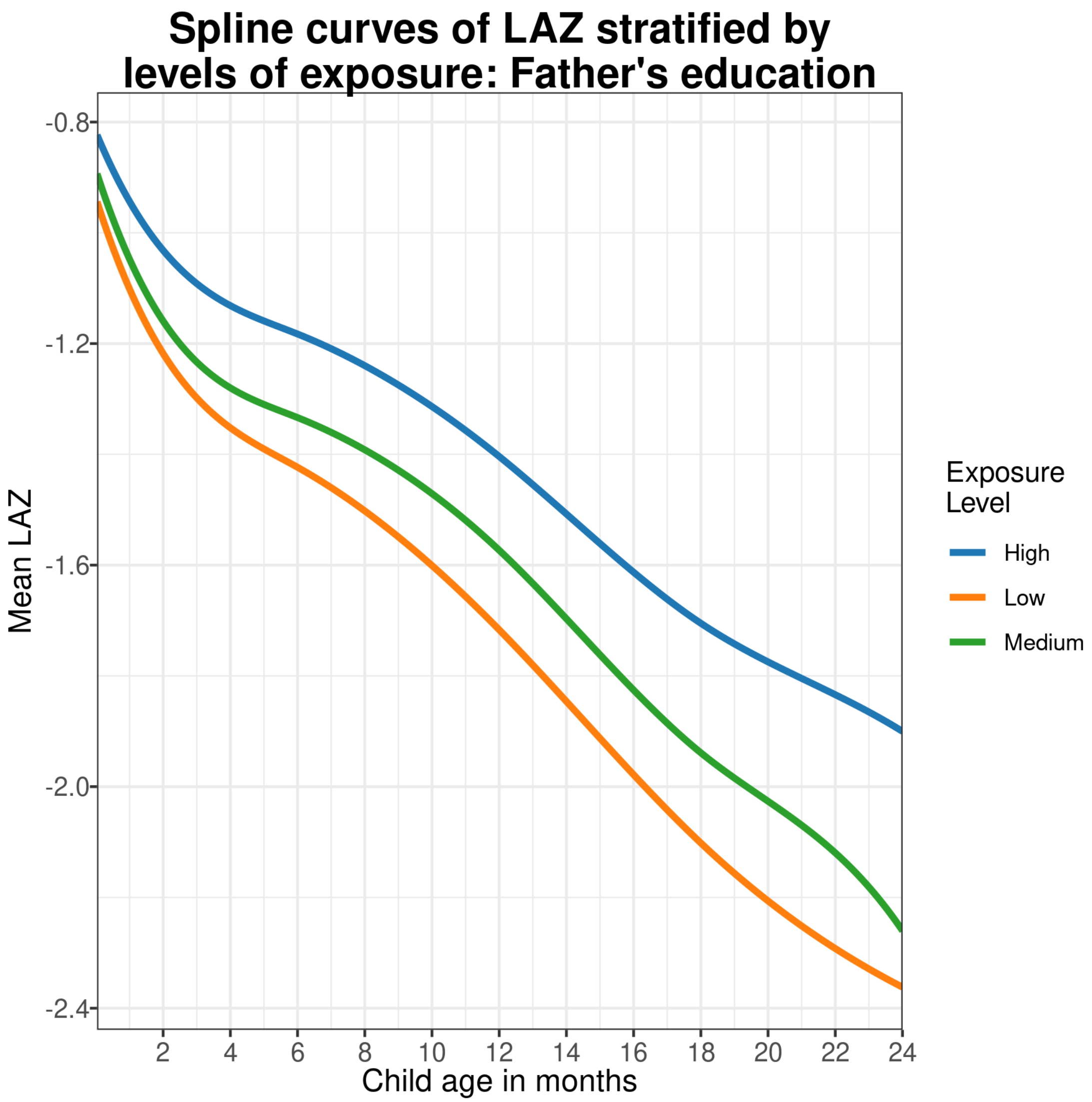
Spline curves of LAZ stratified by levels of exposure: Father's education
Mean LAZ
-0.8
-1.2
-1.6
-2.0
-2.4
2
4
6
8
10
12
14
16
18
20
22
24
Child age in months
Exposure Level
High
Low
Medium

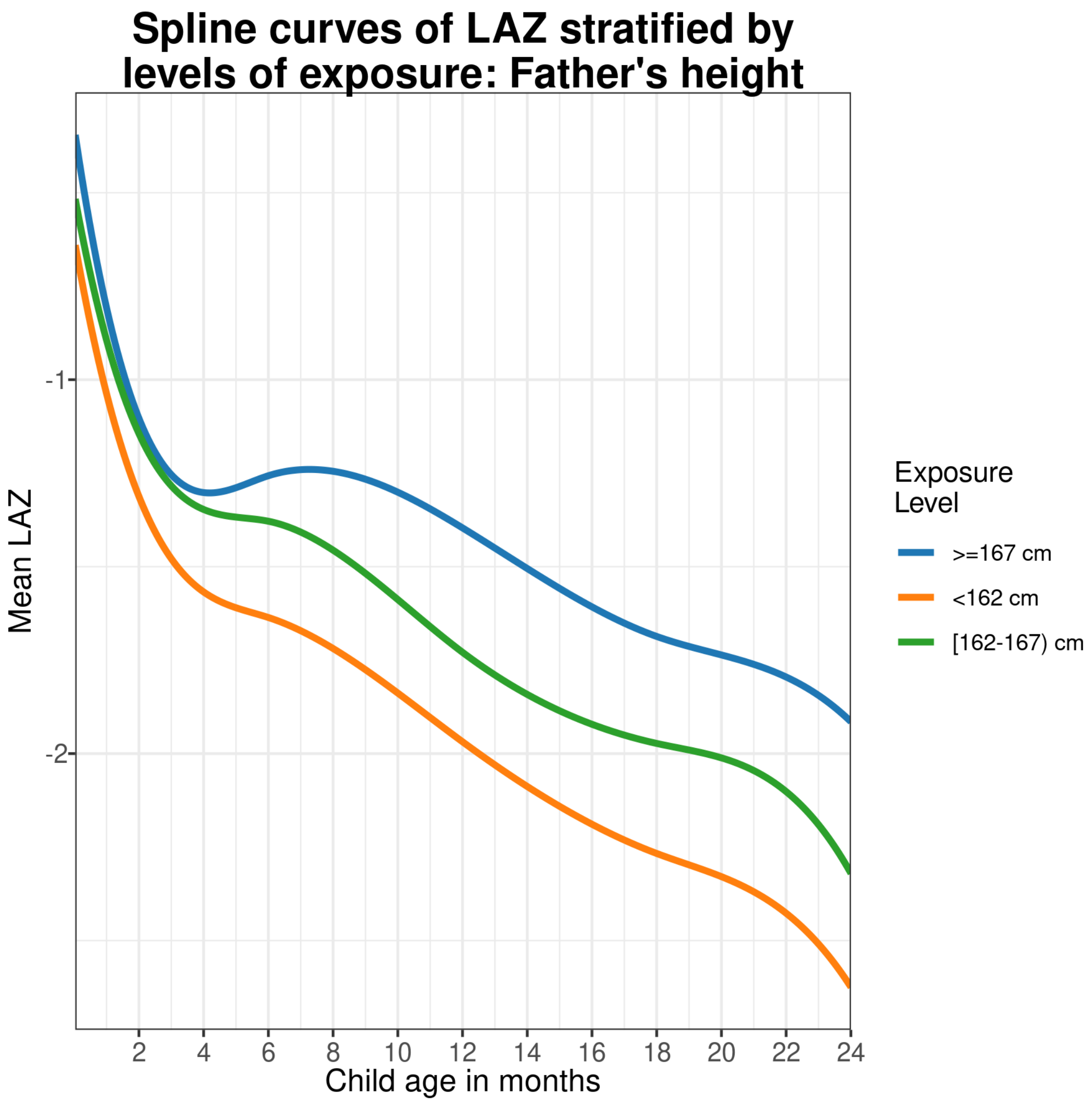
Spline curves of LAZ stratified by
levels of exposure: Father's height
Mean LAZ
-1
-2
2
4
6
8
10
12
14
16
18
20
22
24
Child age in months
Exposure
Level
>=167 cm
<162 cm
[162-167) cm

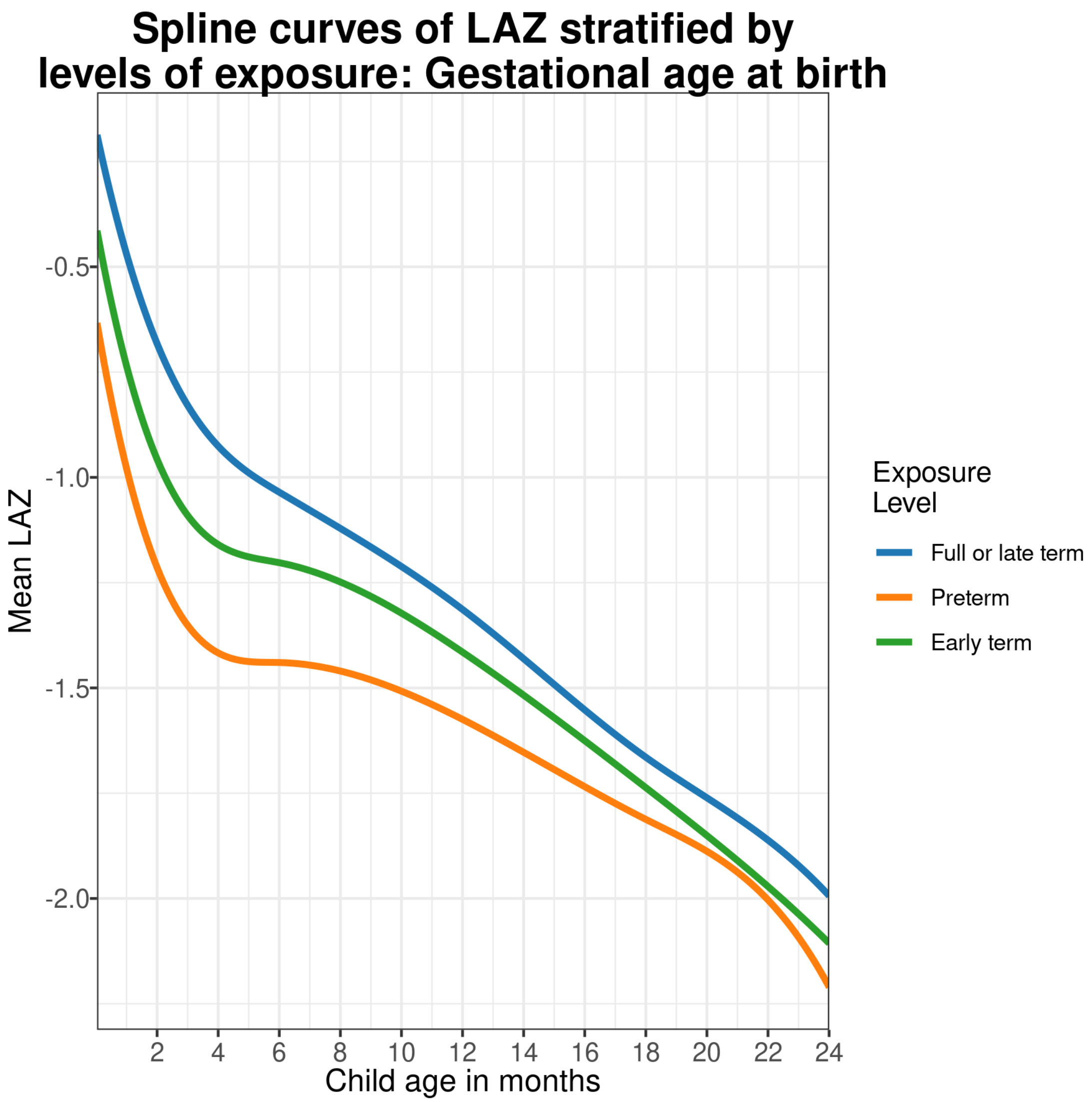

Spline curves of LAZ stratified by
levels of exposure: Gestational age at birth
Mean LAZ
-0.5
-1.0
-1.5
-2.0
2
4
6
8
10
12
14
16
18
20
22
24
Child age in months
Exposure
Level
Full or late term
Preterm
Early term

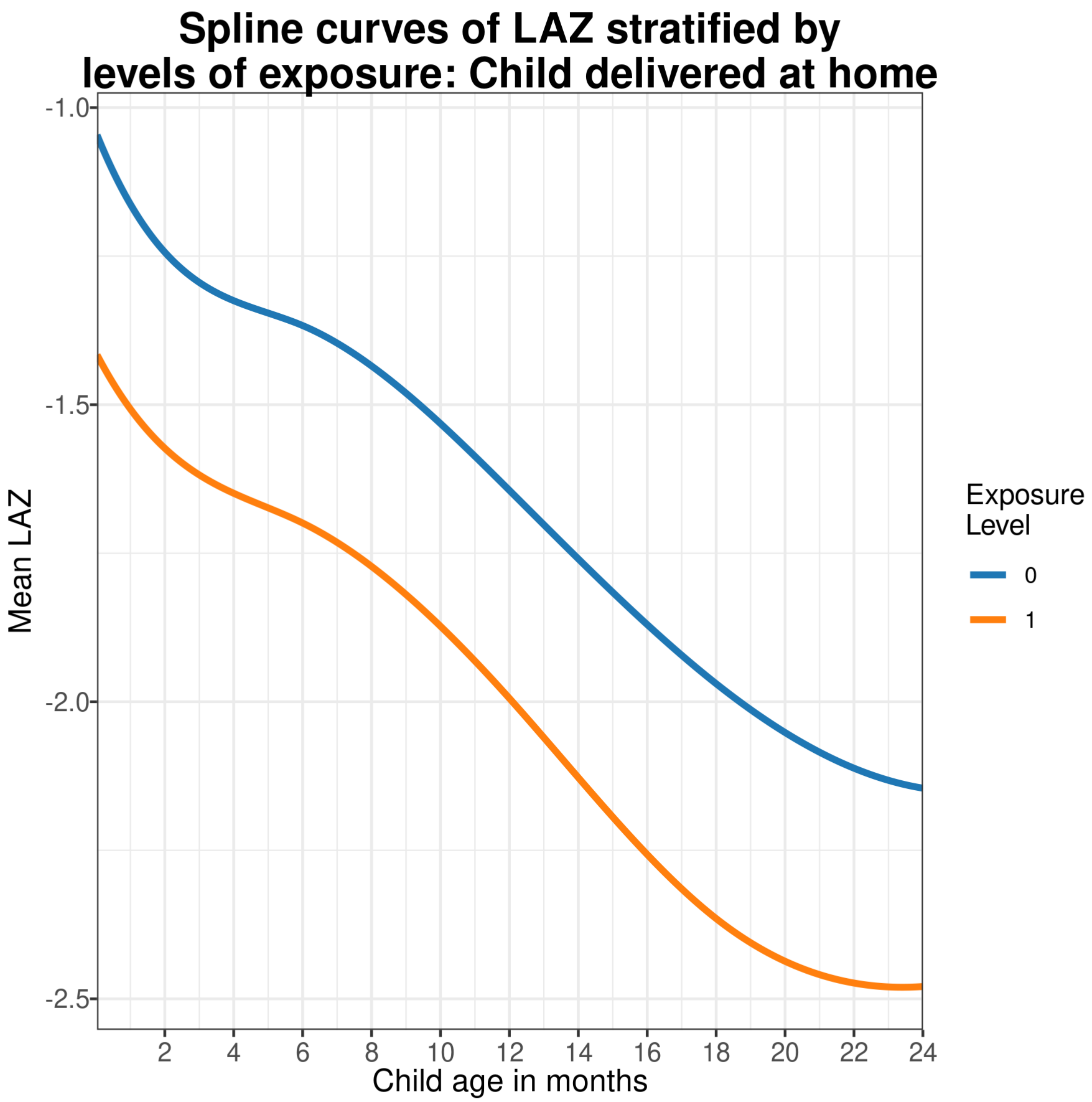

Spline curves of LAZ stratified by
levels of exposure: Child delivered at home
Mean LAZ
-1.0
-1.5
-2.0
-2.5
2
4
6
8
10
12
14
16
18
20
22
24
Child age in months
Exposure Level
0
1

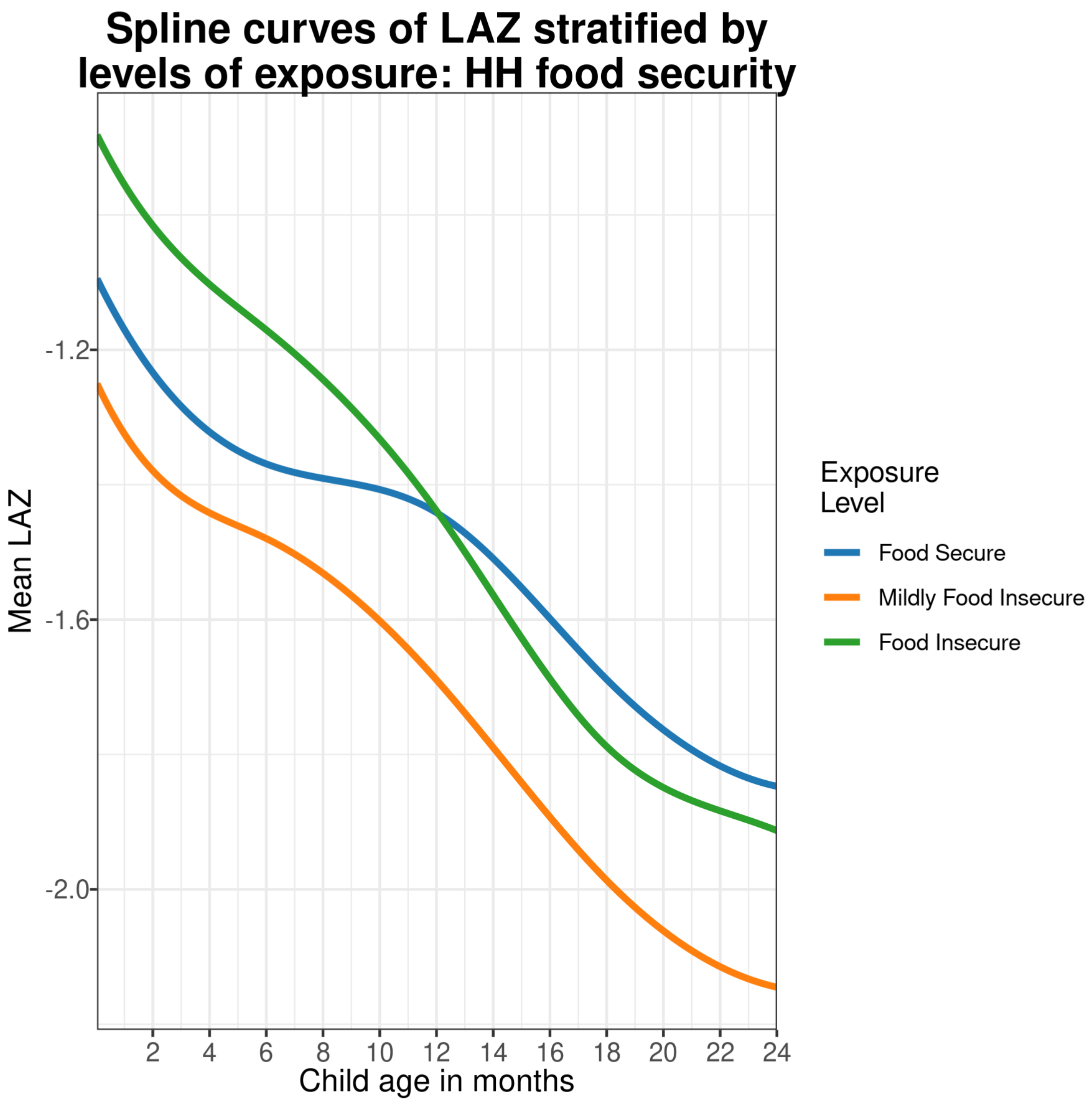
Spline curves of LAZ stratified by
levels of exposure: HH food security
Mean LAZ
-1.2
-1.6
-2.0
2
4
6
8
10
12
14
16
18
20
22
24
Child age in months
Exposure
Level
Food Secure
Mildly Food Insecure
Food Insecure

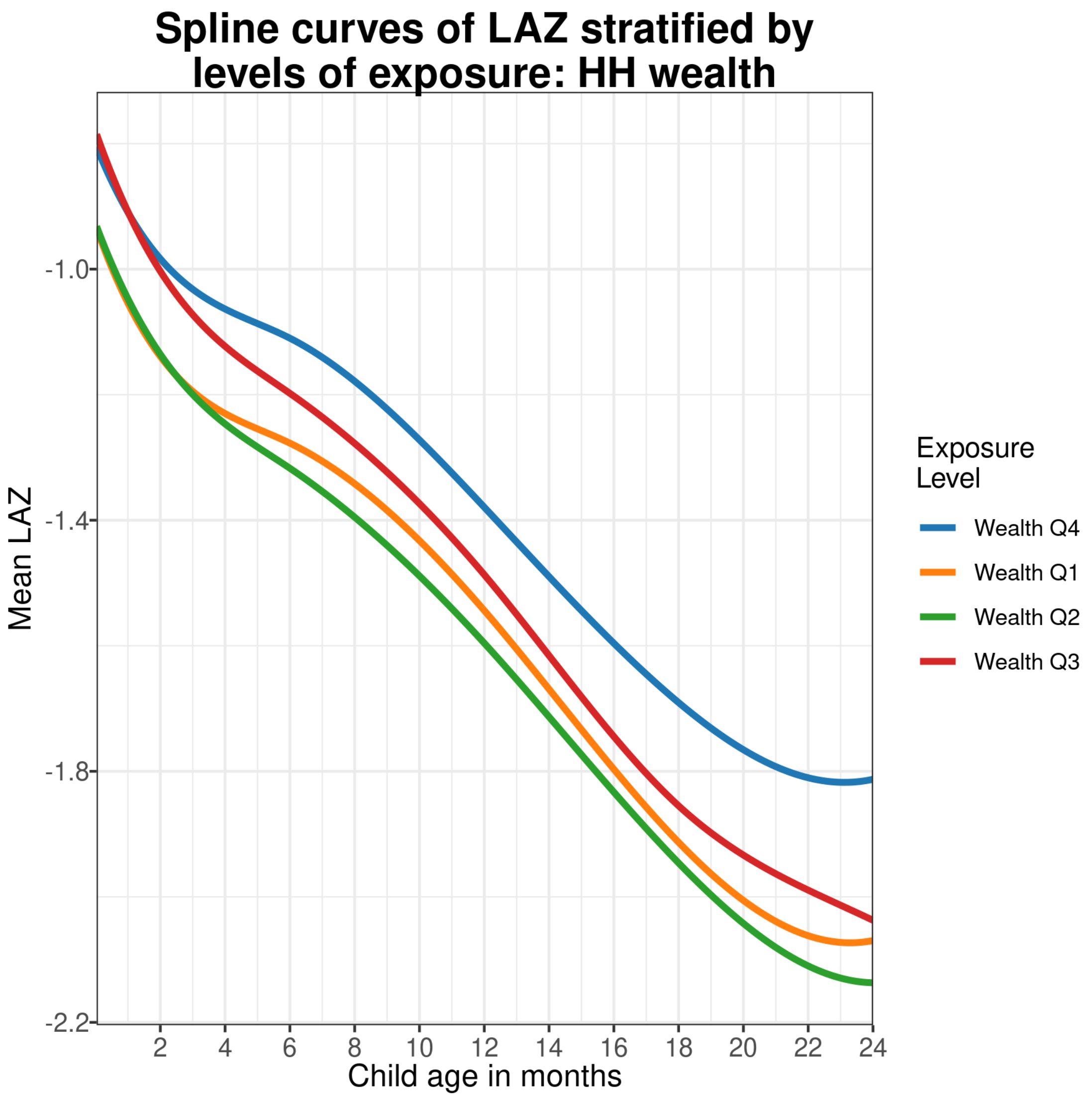
Spline curves of LAZ stratified by levels of exposure: HH wealth
Mean LAZ
-1.0
-1.4
-1.8
-2.2
2
4
6
8
10
12
14
16
18
20
22
24
Child age in months
Exposure Level
Wealth Q4
Wealth Q1
Wealth Q2
Wealth Q3

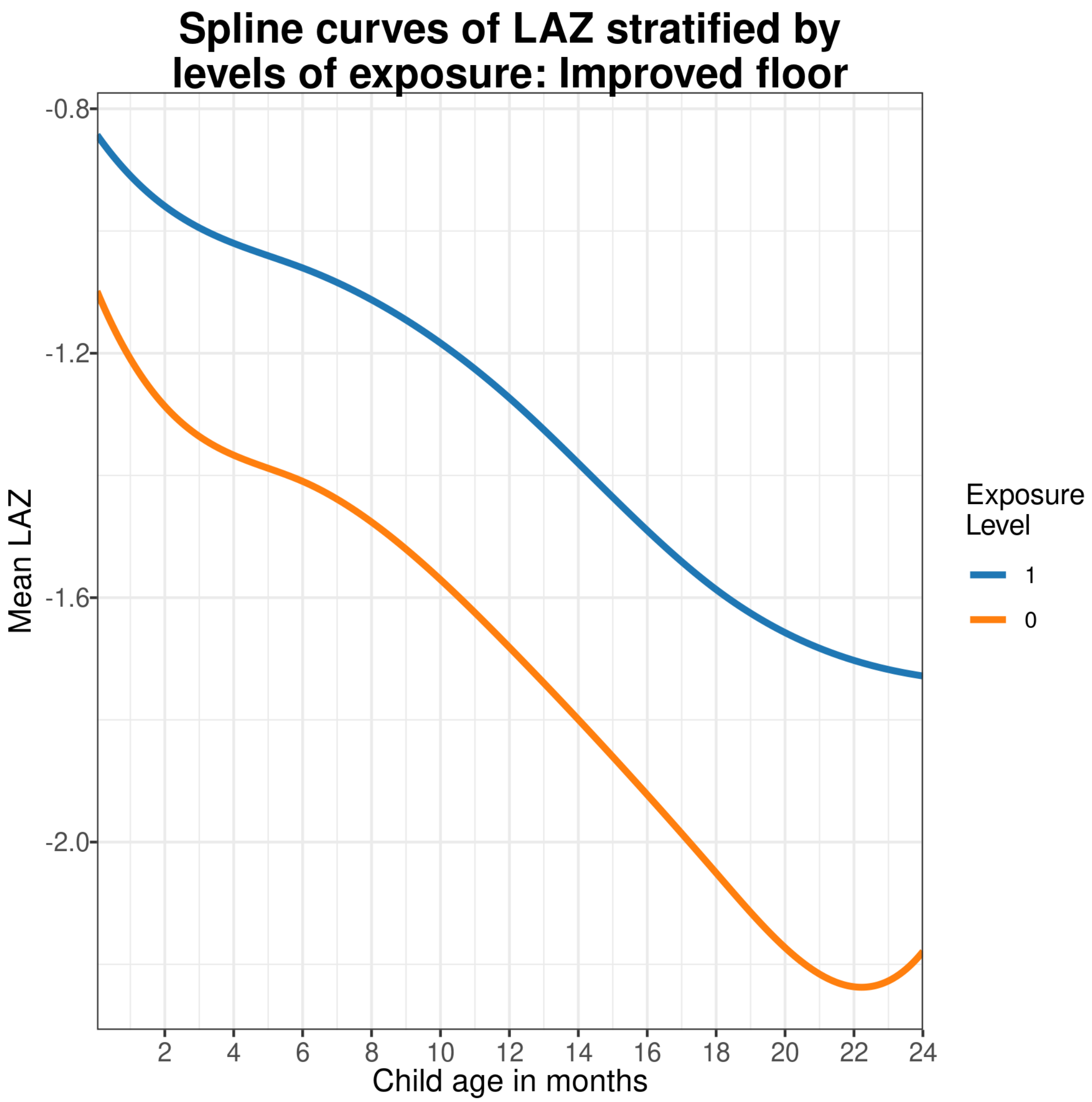

Spline curves of LAZ stratified by levels of exposure: Improved floor
-0.8
-1.2
-1.6
-2.0
Mean LAZ
2
4
6
8
10
12
14
16
18
20
22
24
Child age in months
Exposure Level
1
0

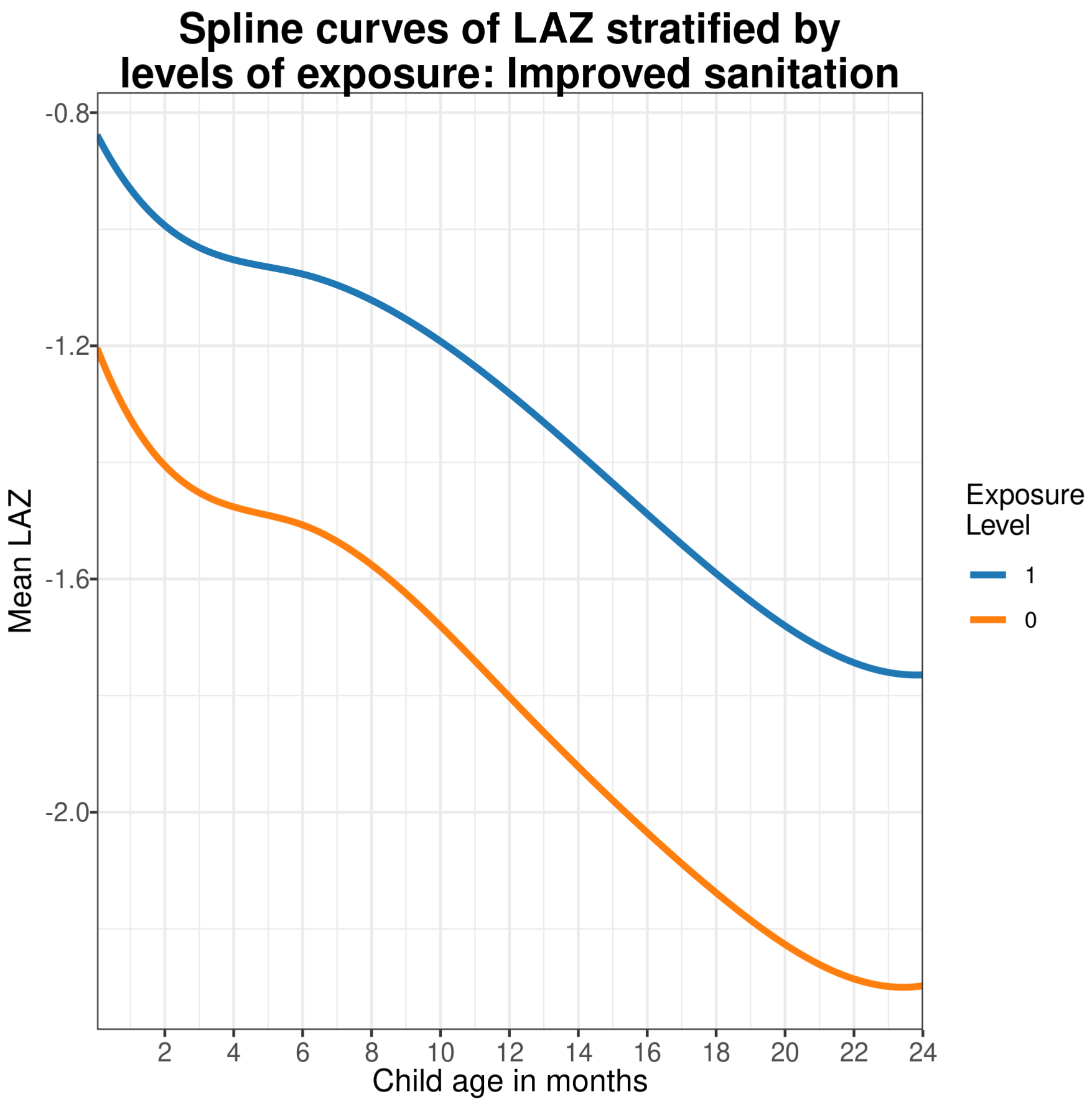

Spline curves of LAZ stratified by
levels of exposure: Improved sanitation
Mean LAZ
-0.8
-1.2
-1.6
-2.0
2
4
6
8
10
12
14
16
18
20
22
24
Child age in months
Exposure
Level
1
0

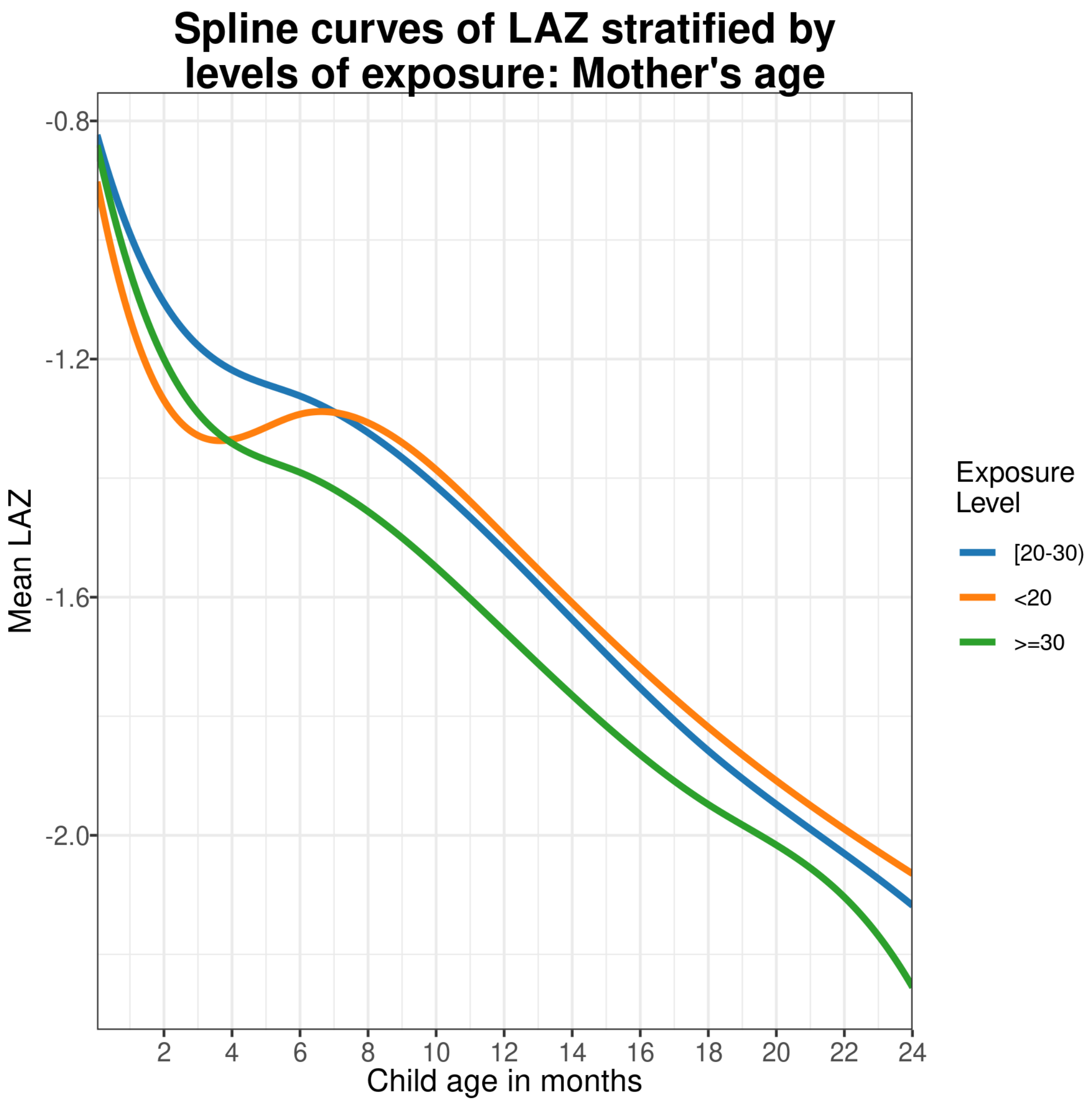

Spline curves of LAZ stratified by levels of exposure: Mother's age
Mean LAZ
-0.8
-1.2
-1.6
-2.0
Child age in months
2
4
6
8
10
12
14
16
18
20
22
24
Exposure Level
[20-30)
<20
>=30

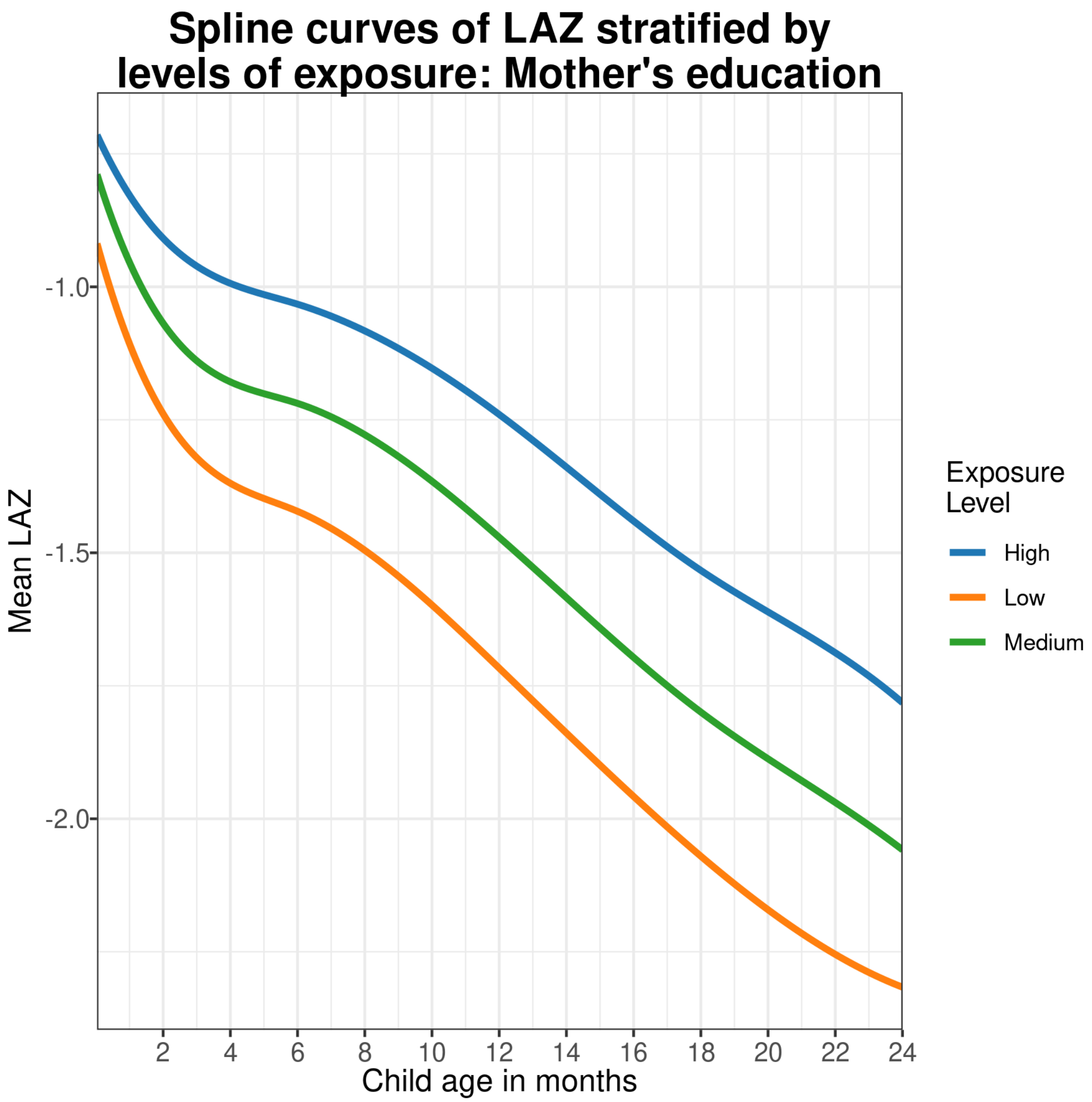

Spline curves of LAZ stratified by
levels of exposure: Mother's education
Mean LAZ
-1.0
-1.5
-2.0
2
4
6
8
10
12
14
16
18
20
22
24
Child age in months
Exposure
Level
High
Low
Medium

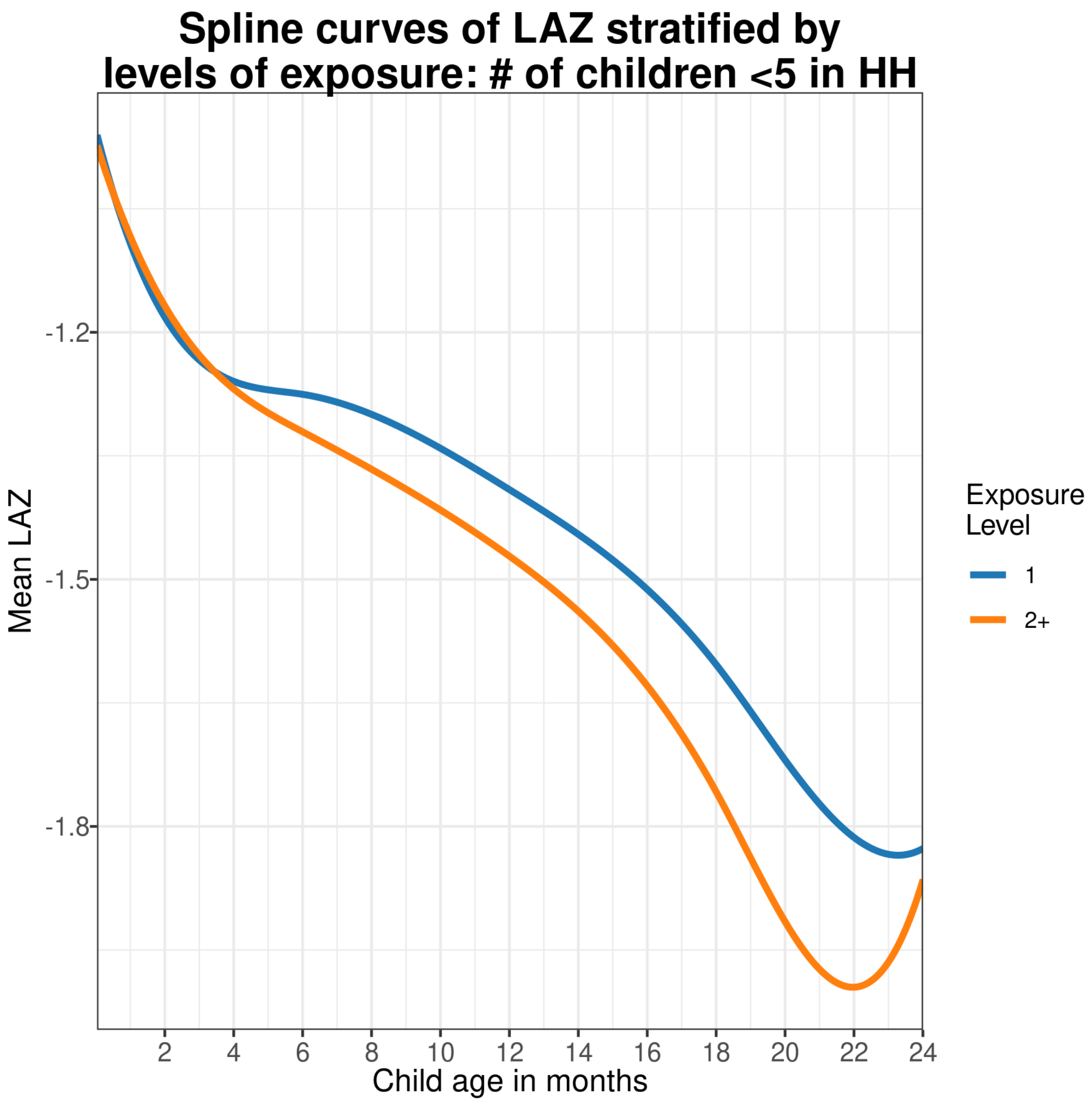
Spline curves of LAZ stratified by
levels of exposure: # of children <5 in HH
Mean LAZ
-1.2
-1.5
-1.8
2
4
6
8
10
12
14
16
18
20
22
24
Child age in months
Exposure
Level
1
2+

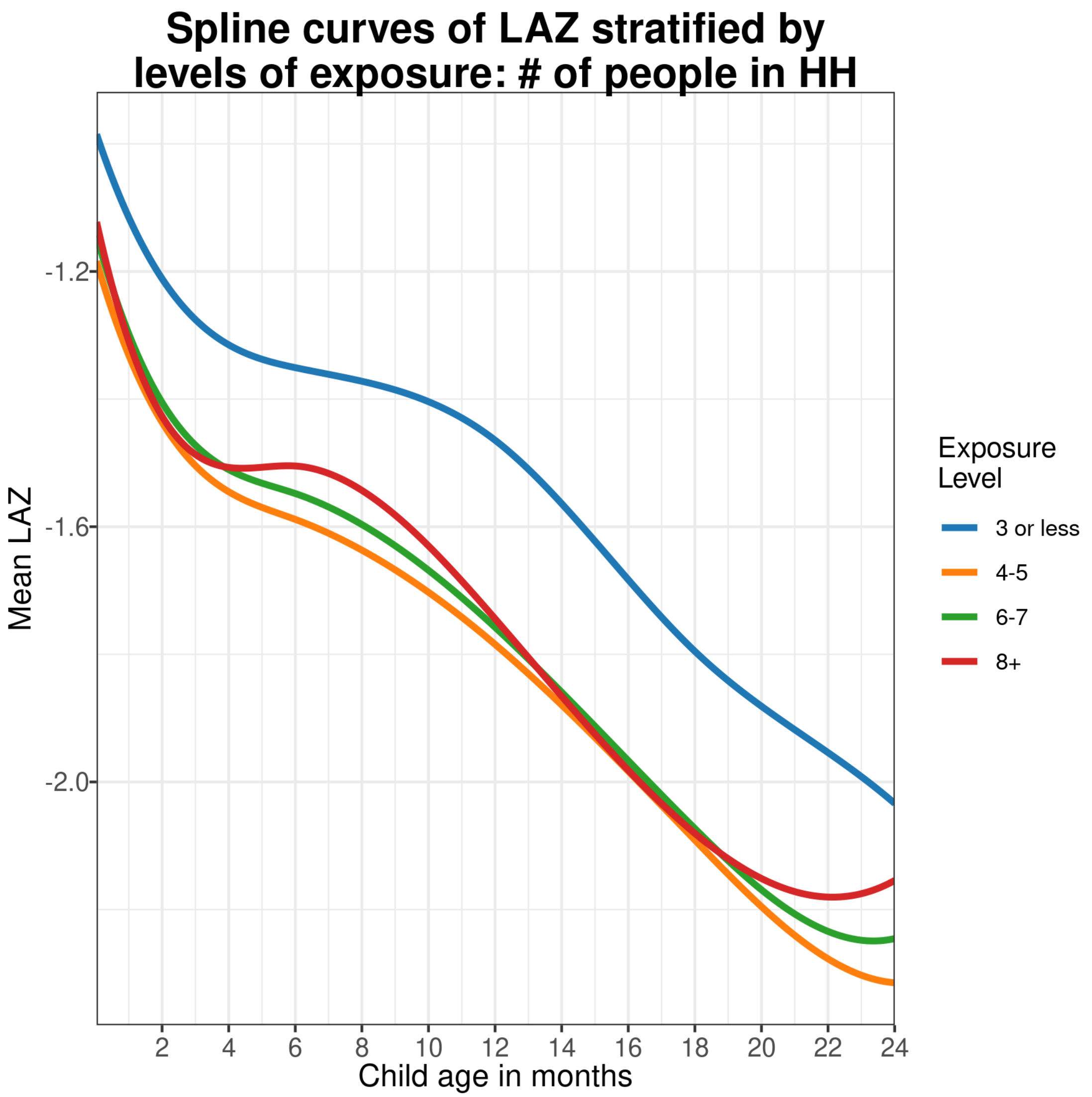
Spline curves of LAZ stratified by
levels of exposure: # of people in HH
Mean LAZ
-1.2
-1.6
-2.0
2
4
6
8
10
12
14
16
18
20
22
24
Child age in months
Exposure
Level
3 or less
4-5
6-7
8+

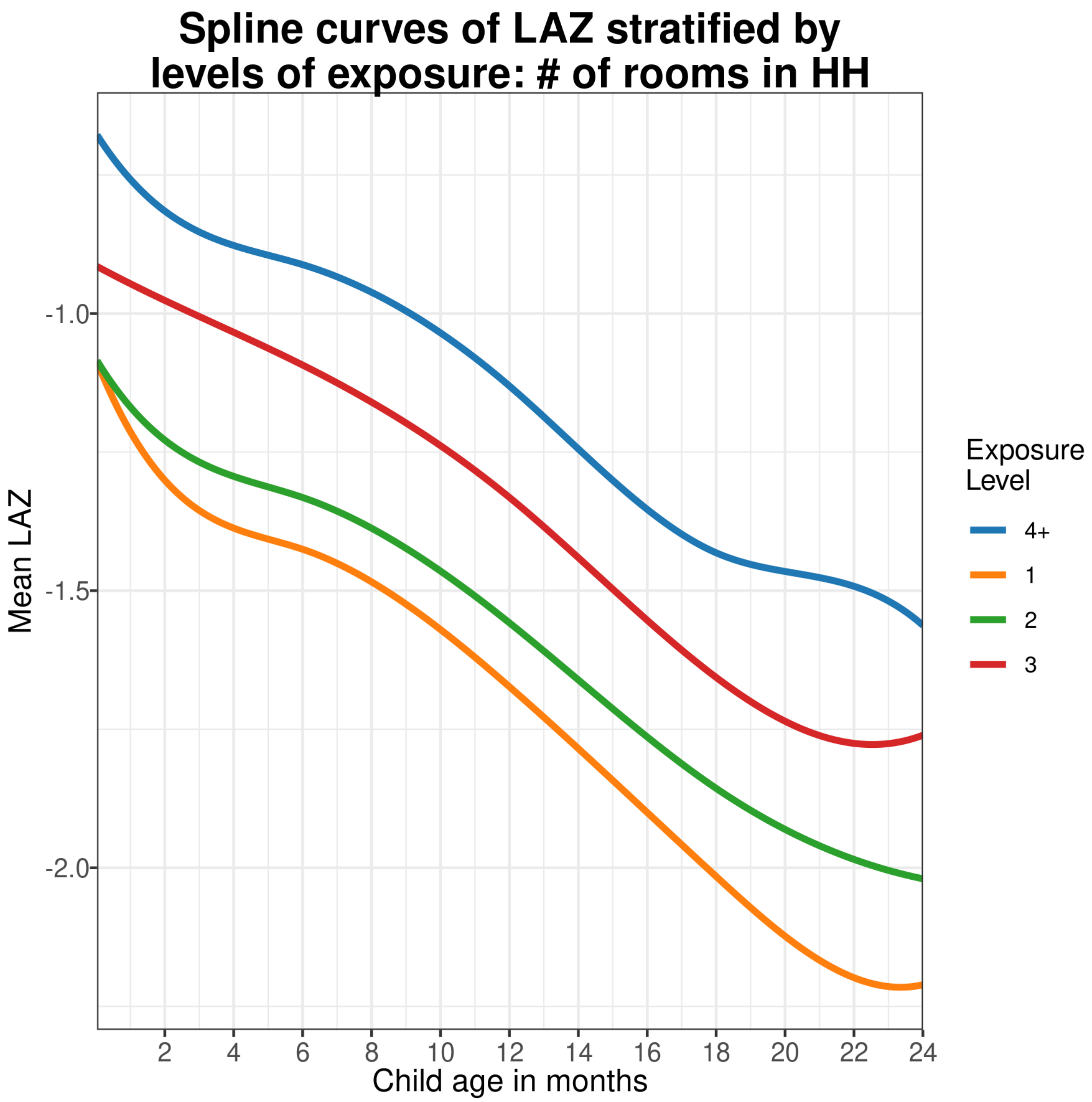
Spline curves of LAZ stratified by
levels of exposure: # of rooms in HH
Mean LAZ
-1.0
-1.5
-2.0
2
4
6
8
10
12
14
16
18
20
22
24
Child age in months
Exposure
Level
4+
1
2
3

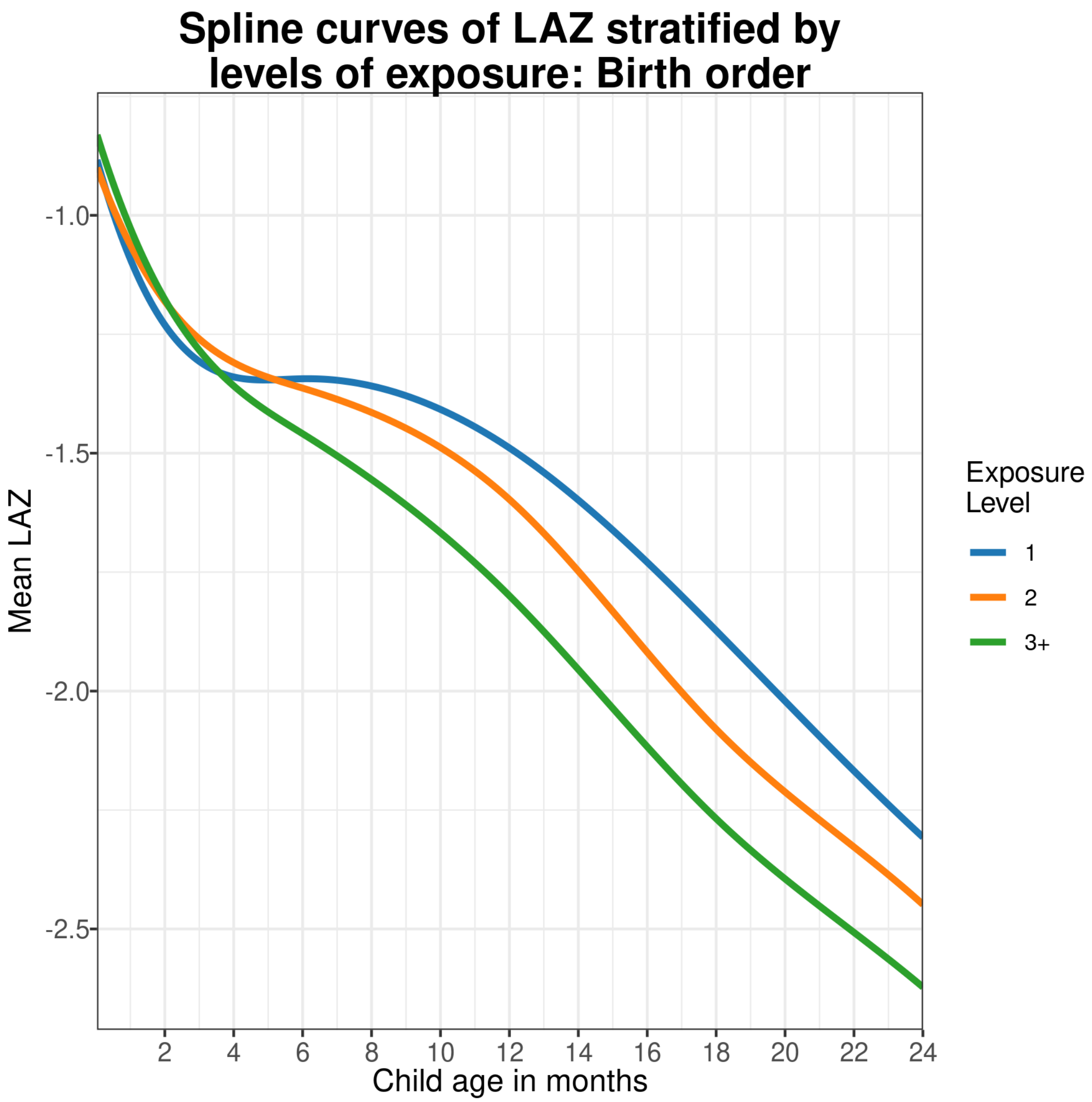
Spline curves of LAZ stratified by
levels of exposure: Birth order
Mean LAZ
-1.0
-1.5
-2.0
-2.5
2
4
6
8
10
12
14
16
18
20
22
24
Child age in months
Exposure
Level
1
2
3+

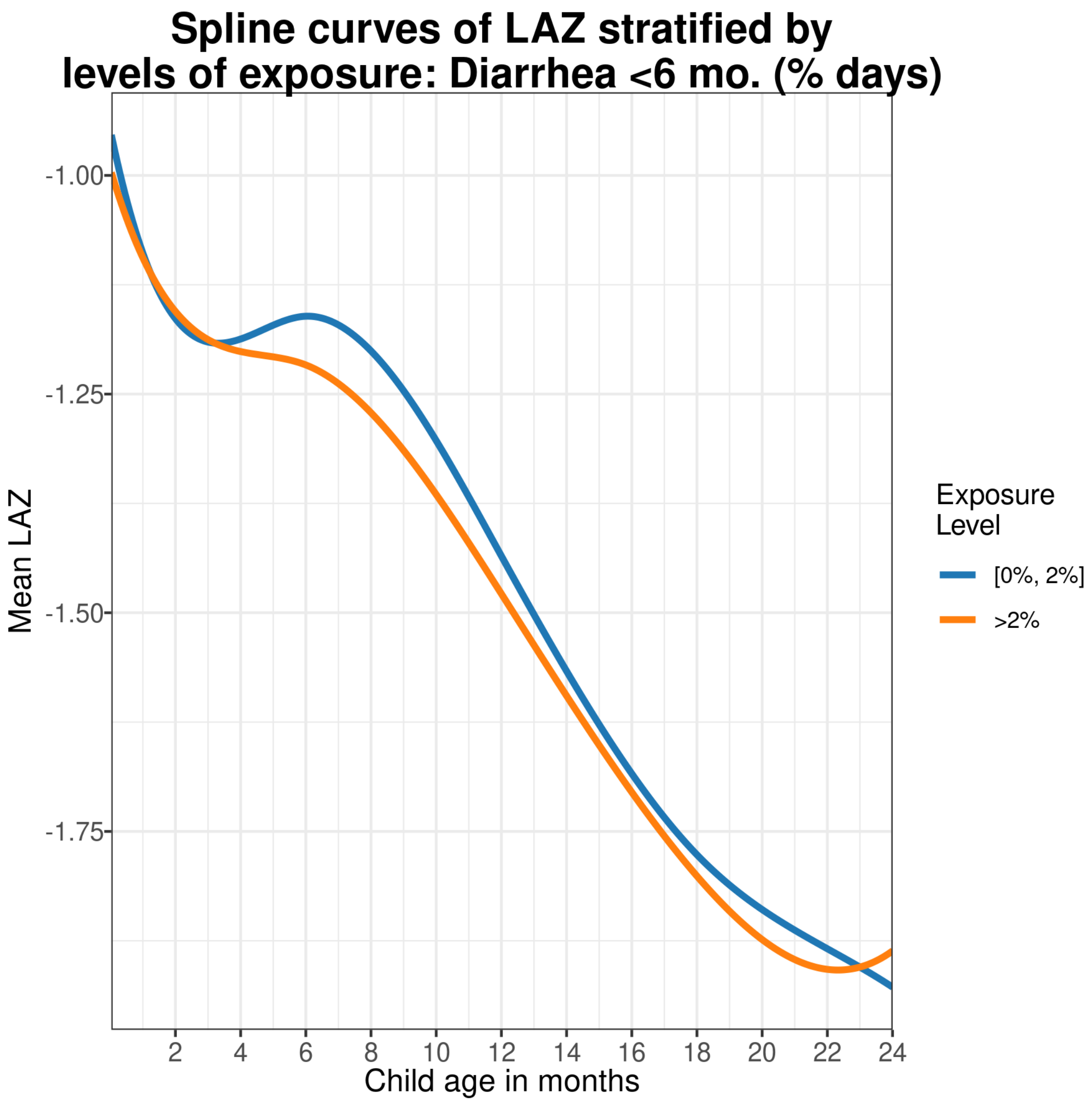
Spline curves of LAZ stratified by
levels of exposure: Diarrhea <6 mo. (% days)
Mean LAZ
-1.00
-1.25
-1.50
-1.75
2
4
6
8
10
12
14
16
18
20
22
24
Child age in months
Exposure
Level
[0%, 2%]
>2%

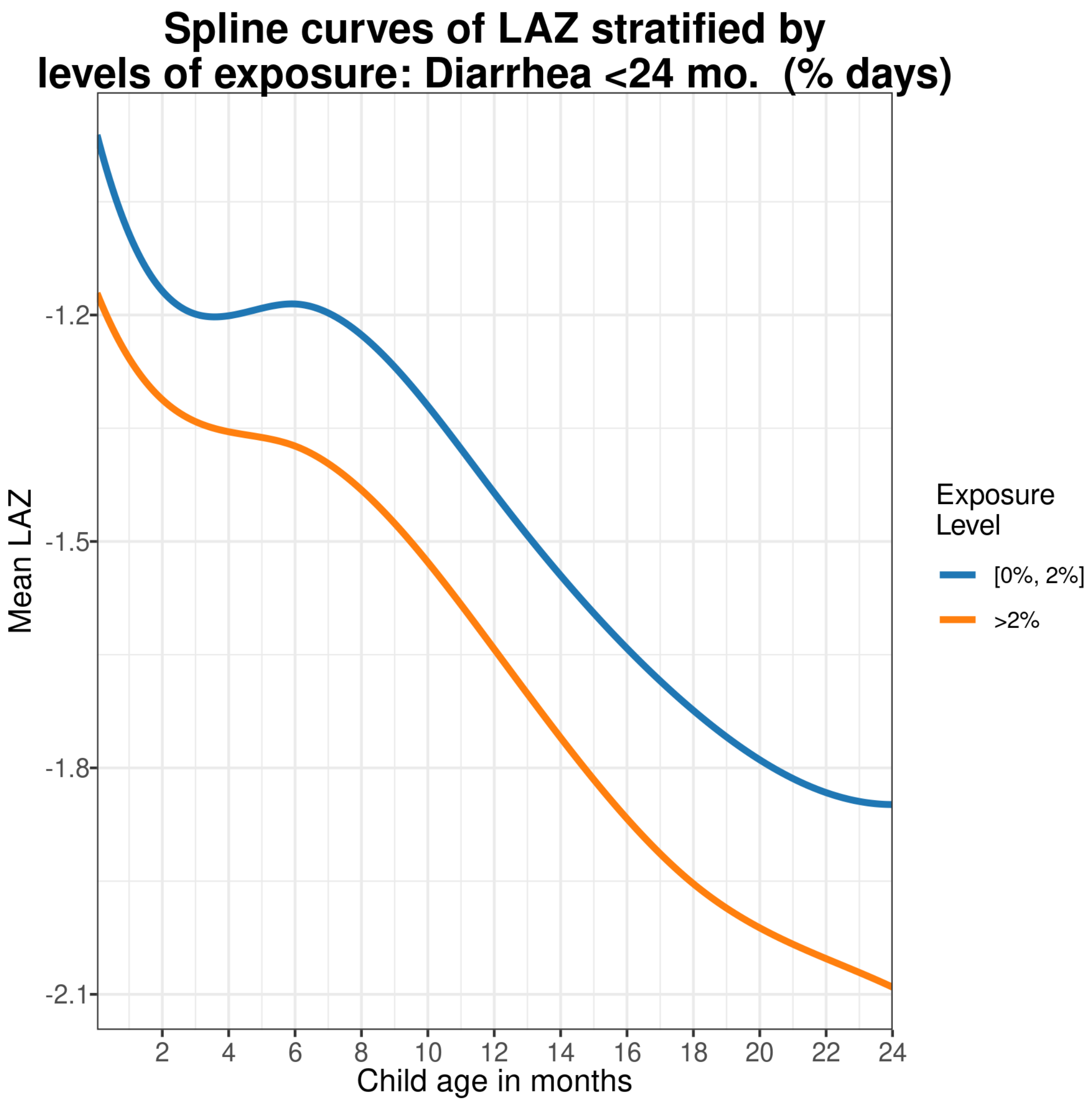
Spline curves of LAZ stratified by
levels of exposure: Diarrhea <24 mo. (% days)
Mean LAZ
-1.2
-1.5
-1.8
-2.1
2
4
6
8
10
12
14
16
18
20
22
24
Child age in months
Exposure
Level
[0%, 2%]
>2%

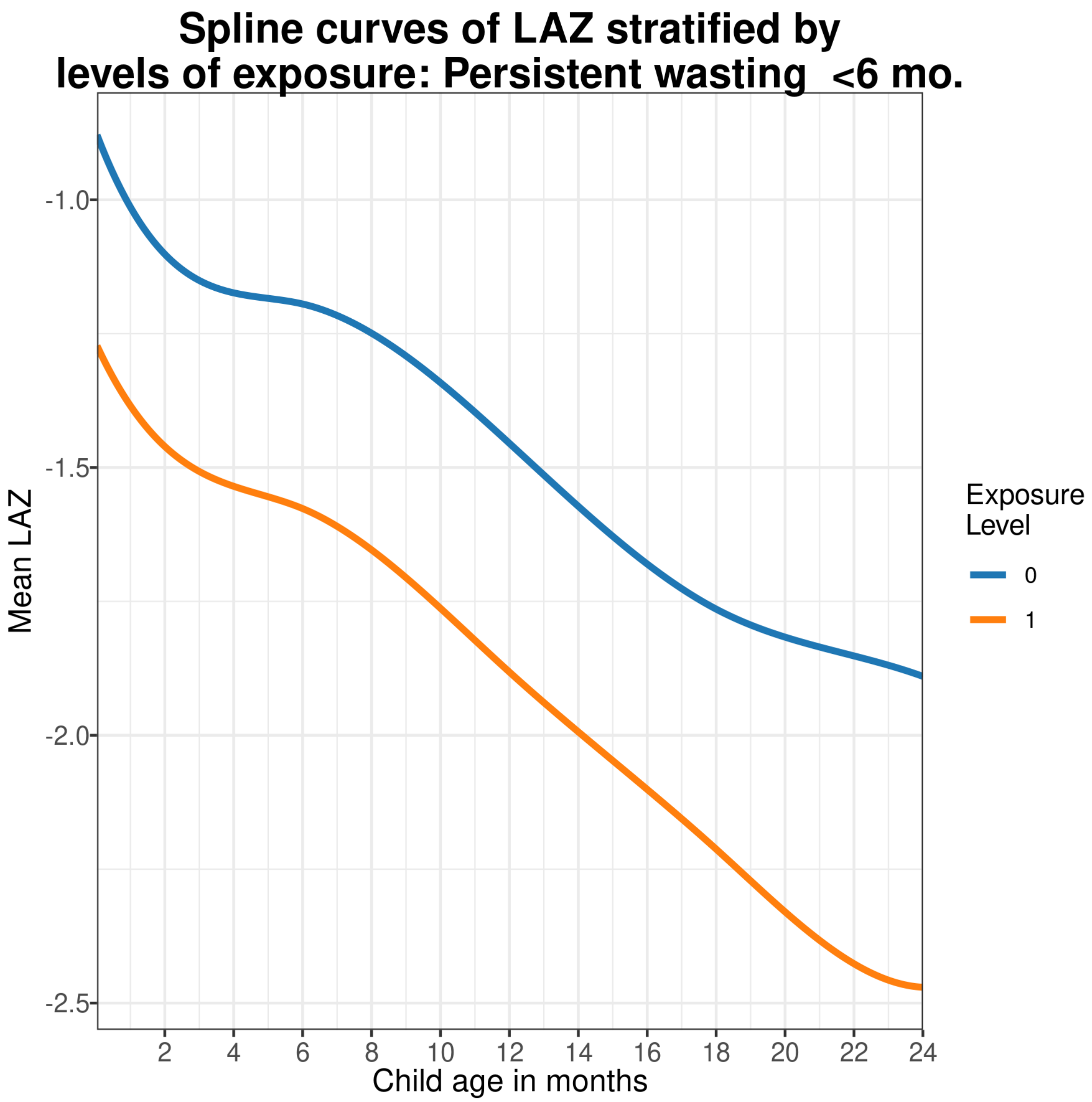

Spline curves of LAZ stratified by
levels of exposure: Persistent wasting <6 mo.
Mean LAZ
-1.0
-1.5
-2.0
-2.5
2
4
6
8
10
12
14
16
18
20
22
24
Child age in months
Exposure
Level
0
1

Spline curves of LAZ stratified by levels of exposure: Excl/Pred breastfed <6mo.

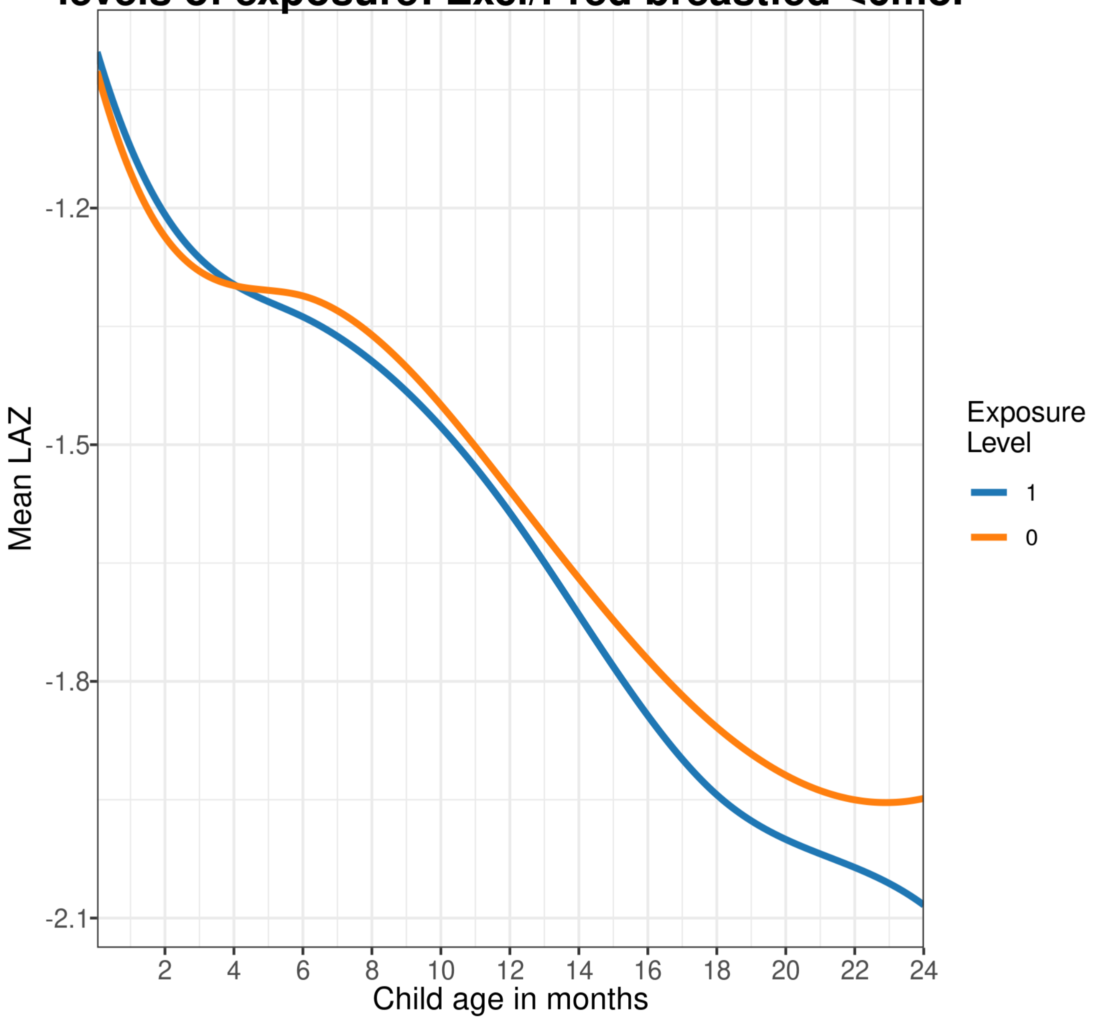

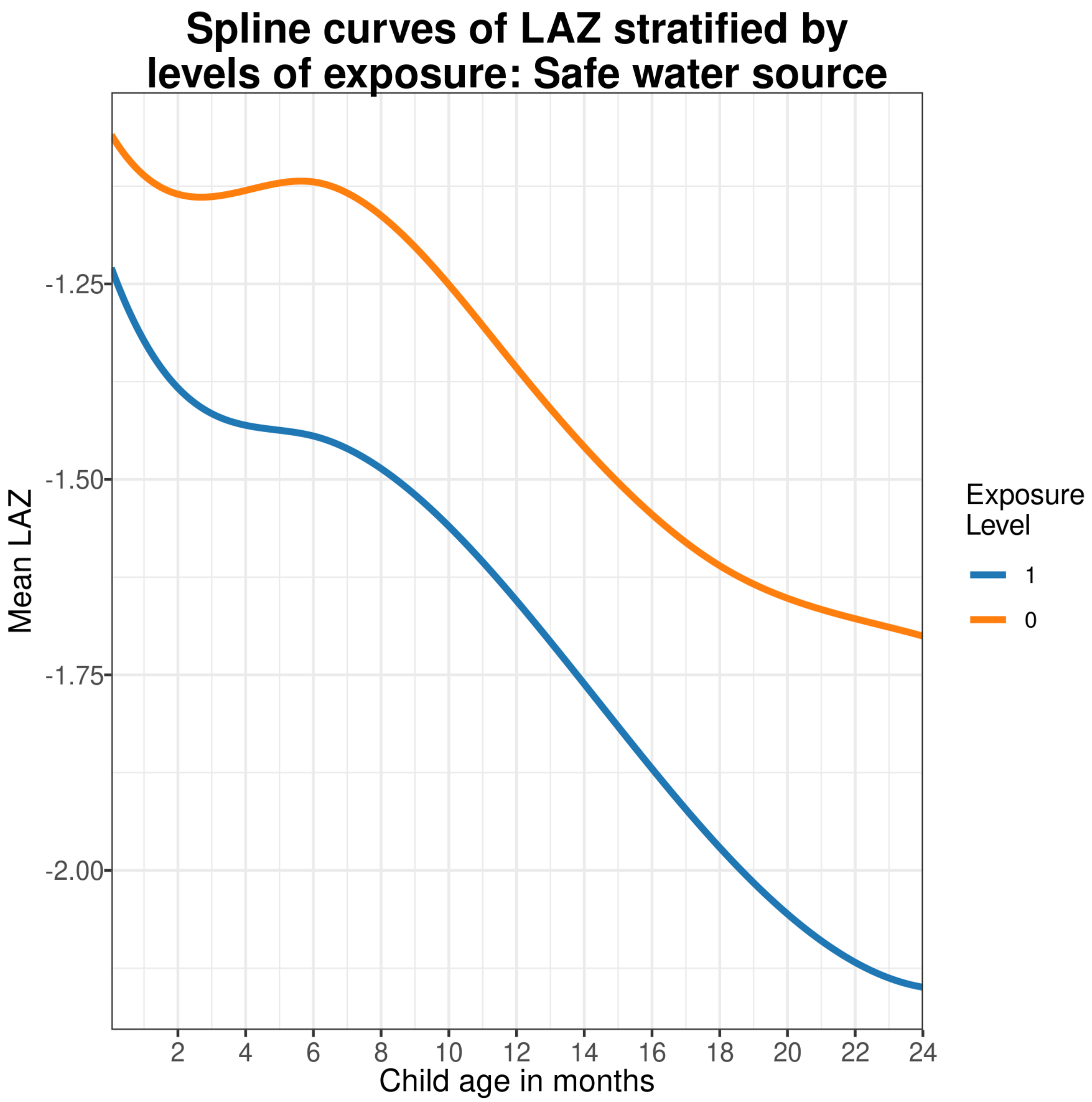
Spline curves of LAZ stratified by
levels of exposure: Safe water source
Mean LAZ
-1.25
-1.50
-1.75
-2.00
2
4
6
8
10
12
14
16
18
20
22
24
Child age in months
Exposure
Level
1
0

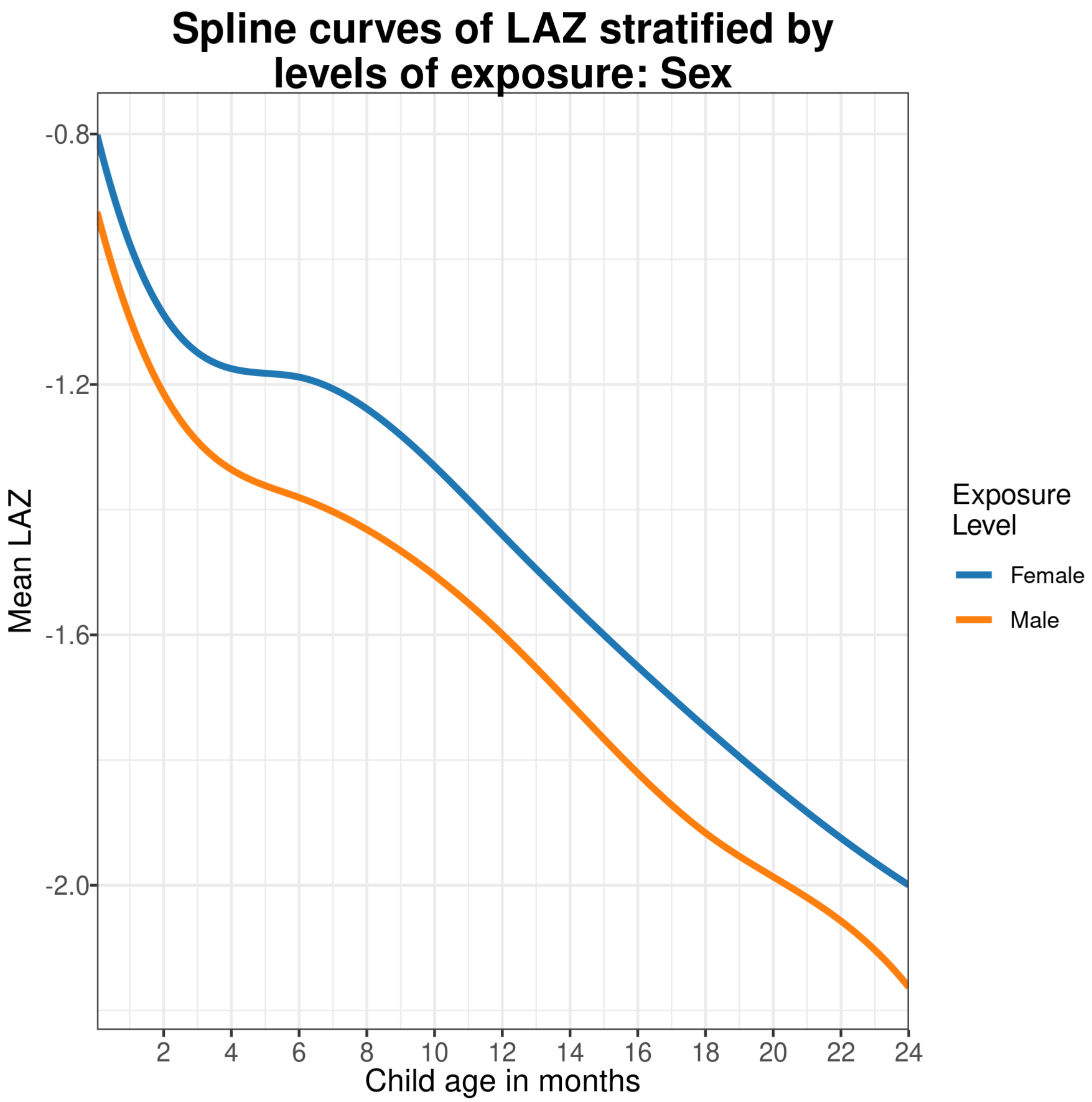

Spline curves of LAZ stratified by levels of exposure: Sex
Mean LAZ
-0.8
-1.2
-1.6
-2.0
2
4
6
8
10
12
14
16
18
20
22
24
Child age in months
Exposure Level
Female
Male

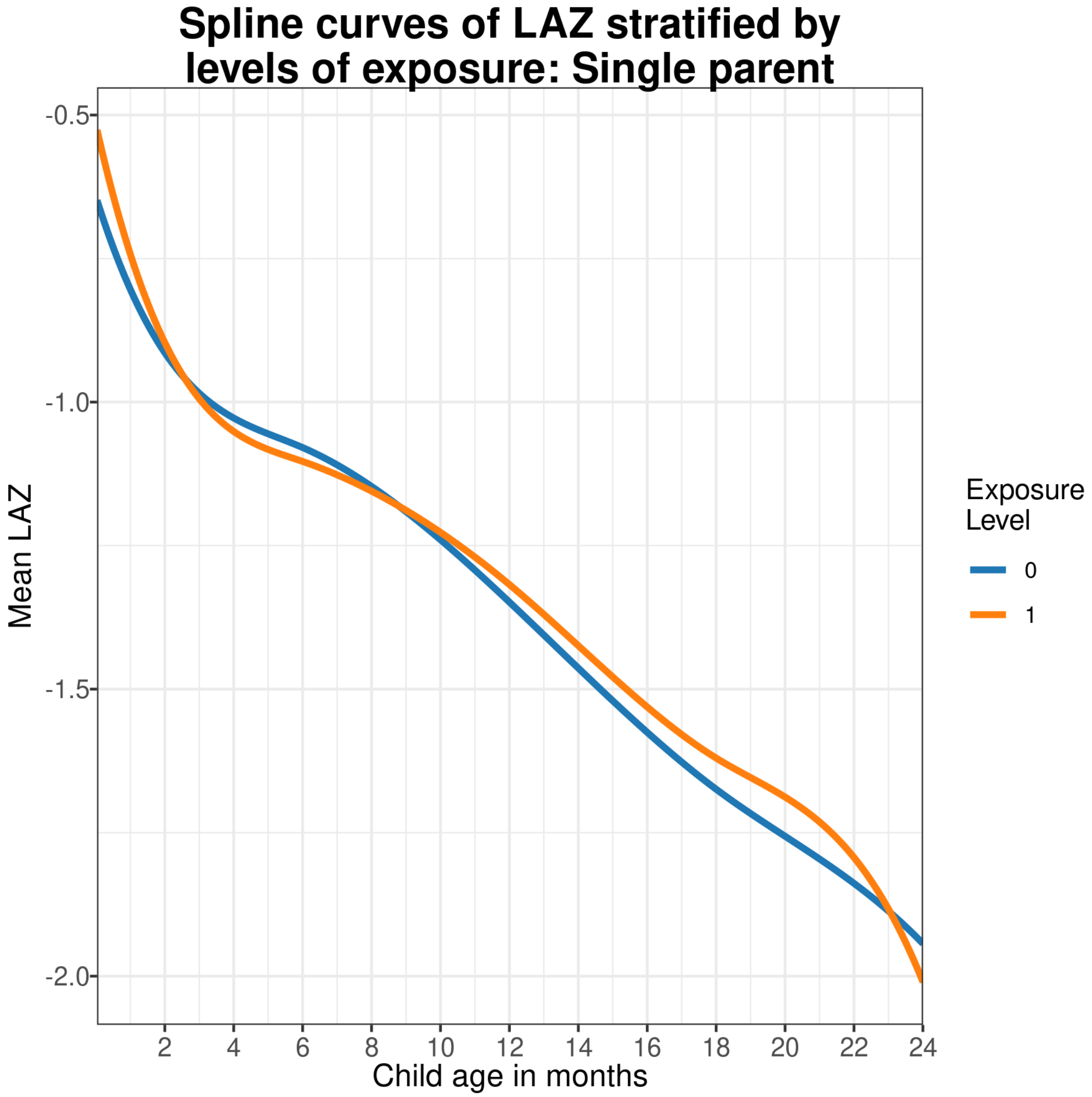
Spline curves of LAZ stratified by
levels of exposure: Single parent
Mean LAZ
-0.5
-1.0
-1.5
-2.0
2
4
6
8
10
12
14
16
18
20
22
24
Child age in months
Exposure
Level
0
1

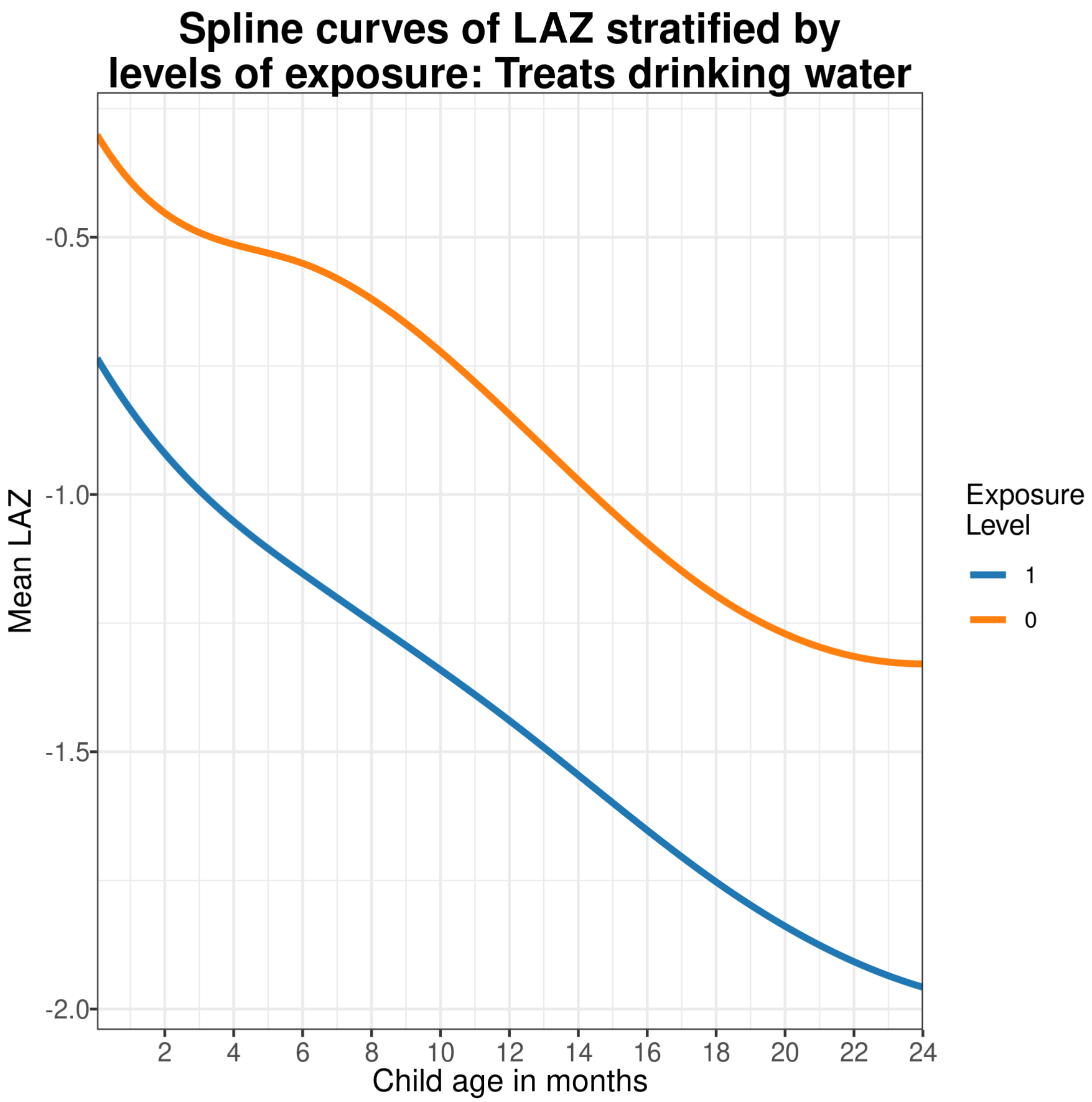
Spline curves of LAZ stratified by
levels of exposure: Treats drinking water
Mean LAZ
-0.5
-1.0
-1.5
-2.0
2
4
6
8
10
12
14
16
18
20
22
24
Child age in months
Exposure
Level
1
0

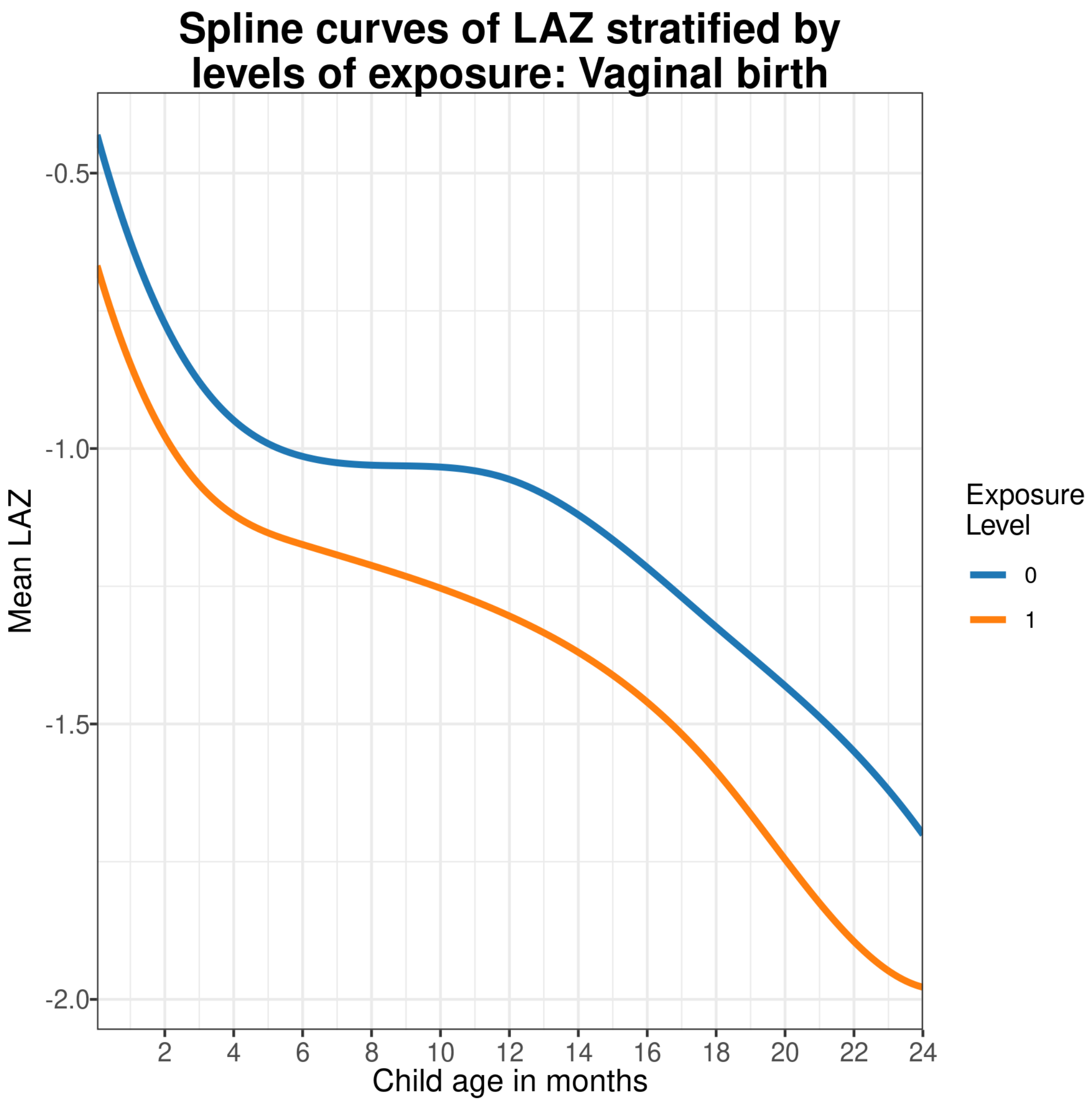
Spline curves of LAZ stratified by levels of exposure: Vaginal birth
Mean LAZ
-0.5
-1.0
-1.5
-2.0
2
4
6
8
10
12
14
16
18
20
22
24
Child age in months
Exposure Level
0
1

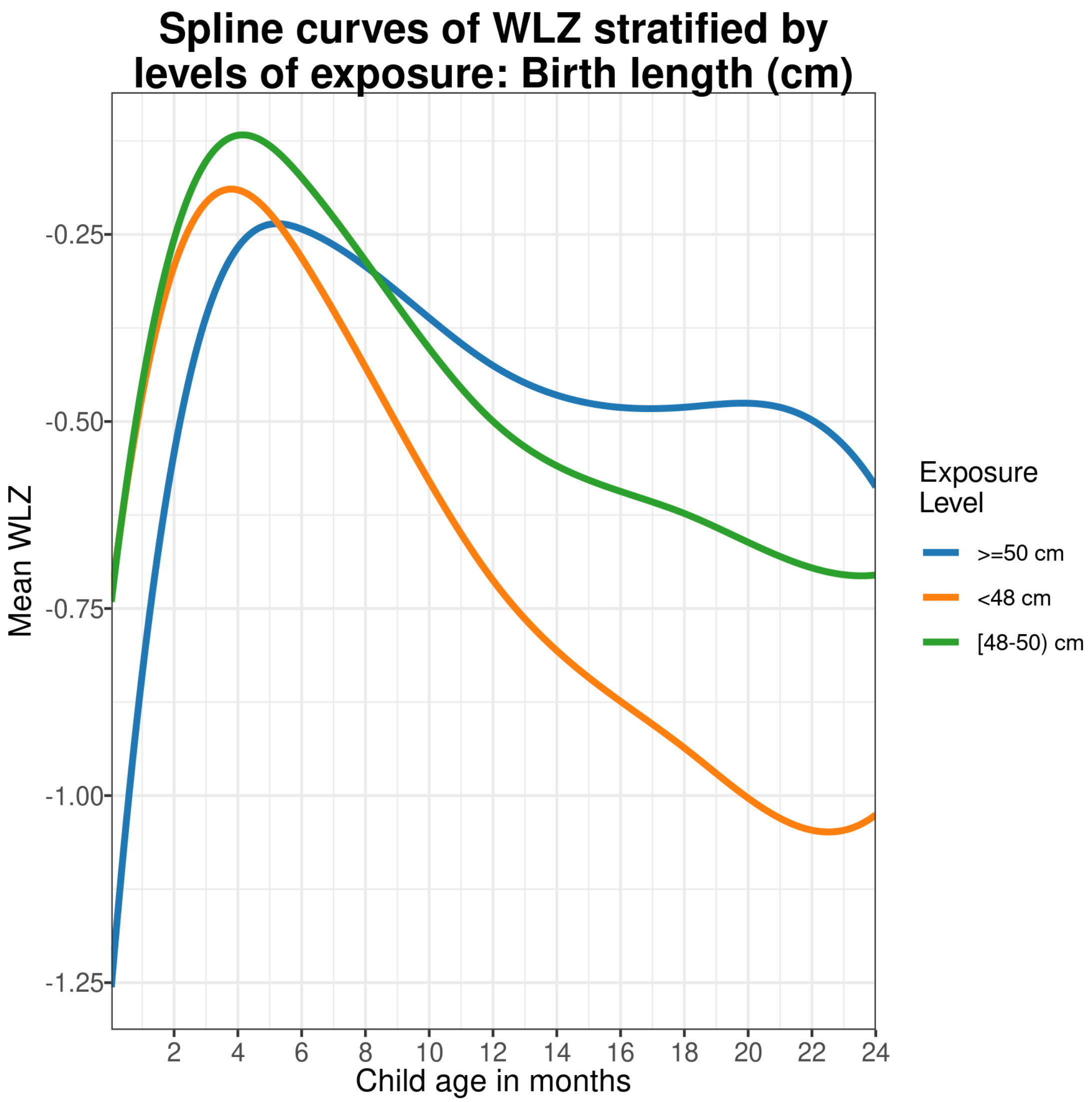
Spline curves of WLZ stratified by
levels of exposure: Birth length (cm)
Mean WLZ
-0.25
-0.50
-0.75
-1.00
-1.25
2
4
6
8
10
12
14
16
18
20
22
24
Child age in months
Exposure
Level
>=50 cm
<48 cm
[48-50) cm

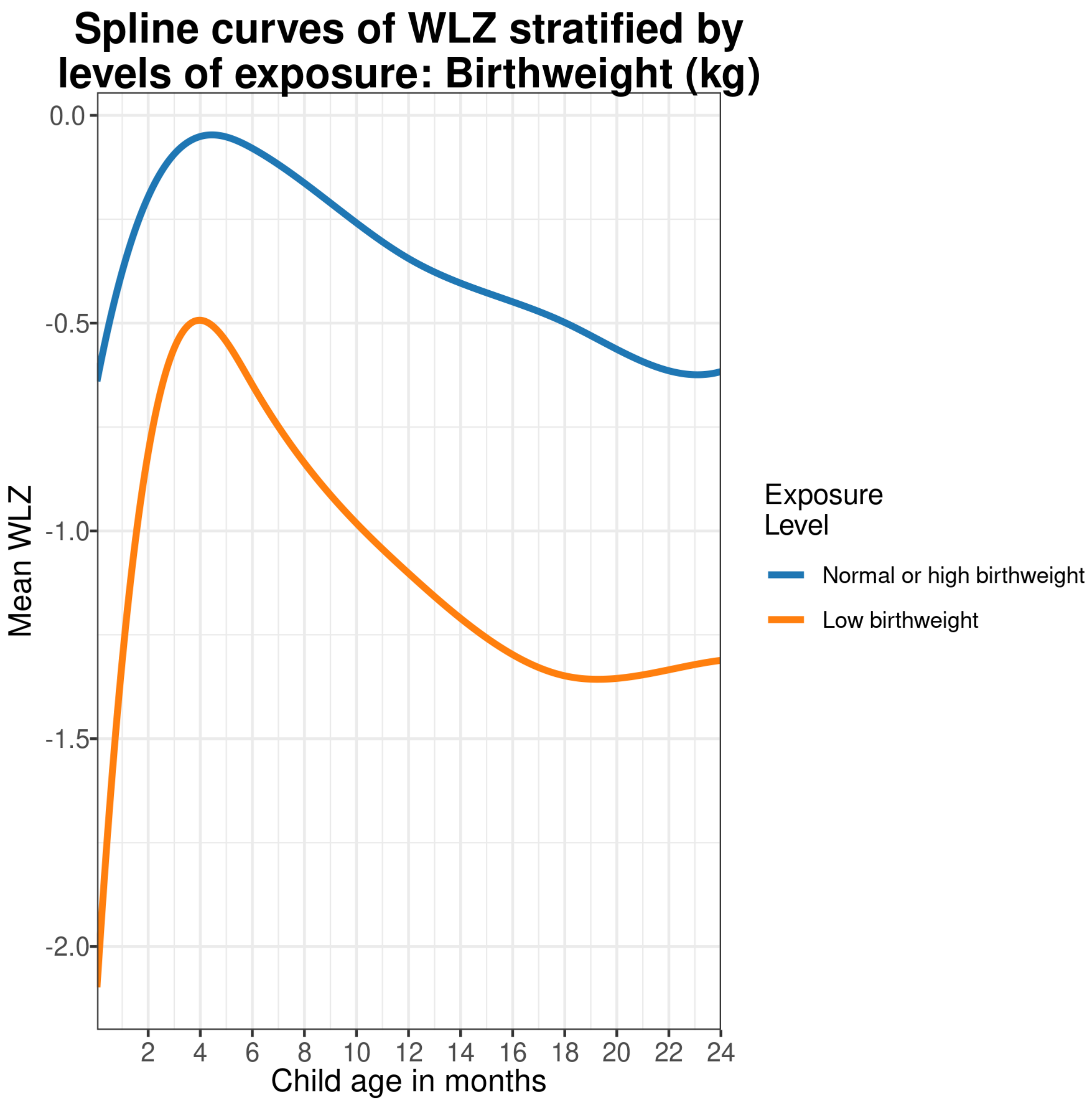

Spline curves of WLZ stratified by levels of exposure: Birthweight (kg)
0.0
-0.5
-1.0
-1.5
-2.0
Mean WLZ
2
4
6
8
10
12
14
16
18
20
22
24
Child age in months
Exposure Level
Normal or high birthweight
Low birthweight

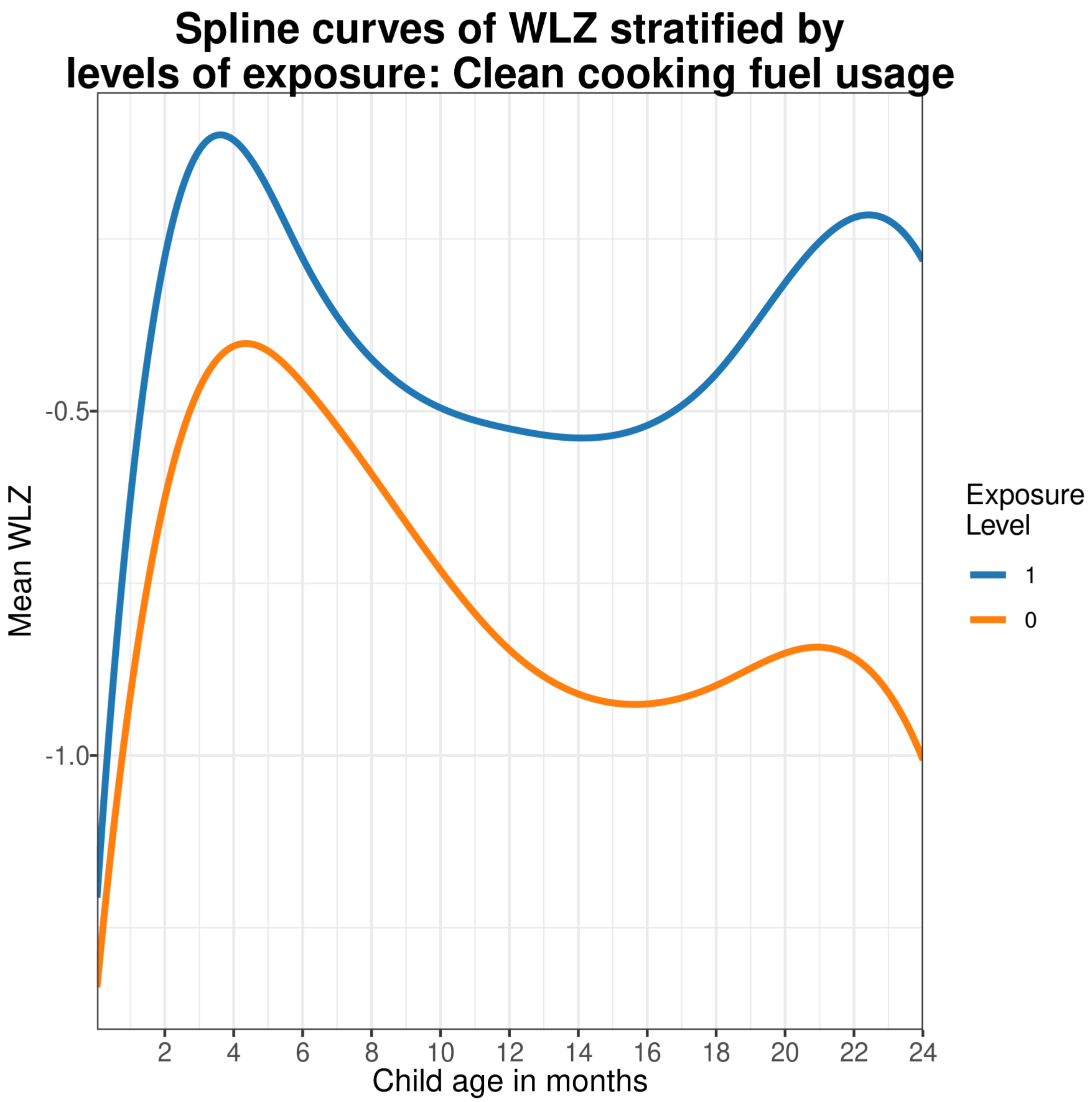
Spline curves of WLZ stratified by
levels of exposure: Clean cooking fuel usage
Mean WLZ
-0.5
-1.0
2
4
6
8
10
12
14
16
18
20
22
24
Child age in months
Exposure
Level
1
0

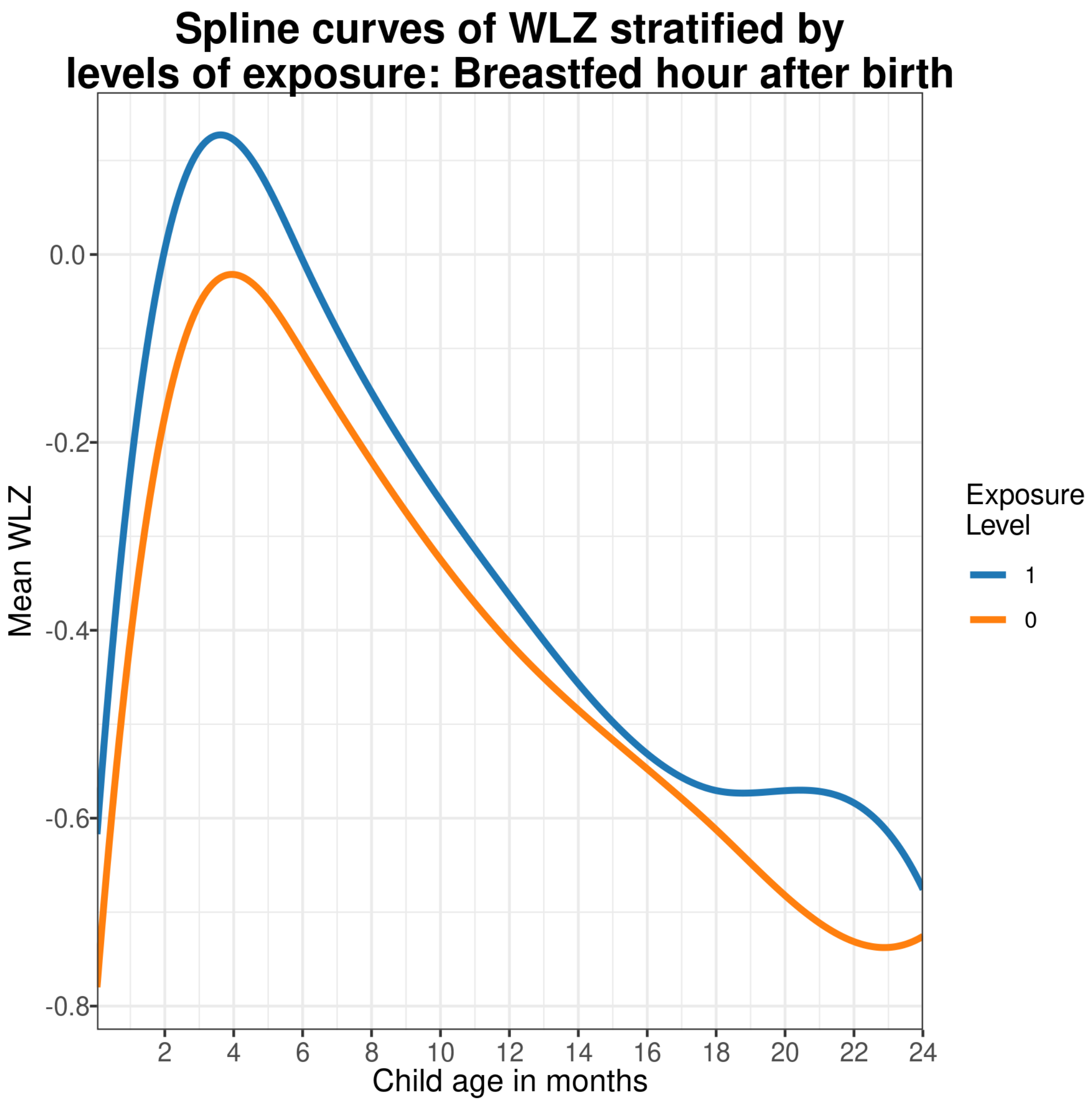
Spline curves of WLZ stratified by
levels of exposure: Breastfed hour after birth
Mean WLZ
0.0
-0.2
-0.4
-0.6
-0.8
2
4
6
8
10
12
14
16
18
20
22
24
Child age in months
Exposure
Level
1
0

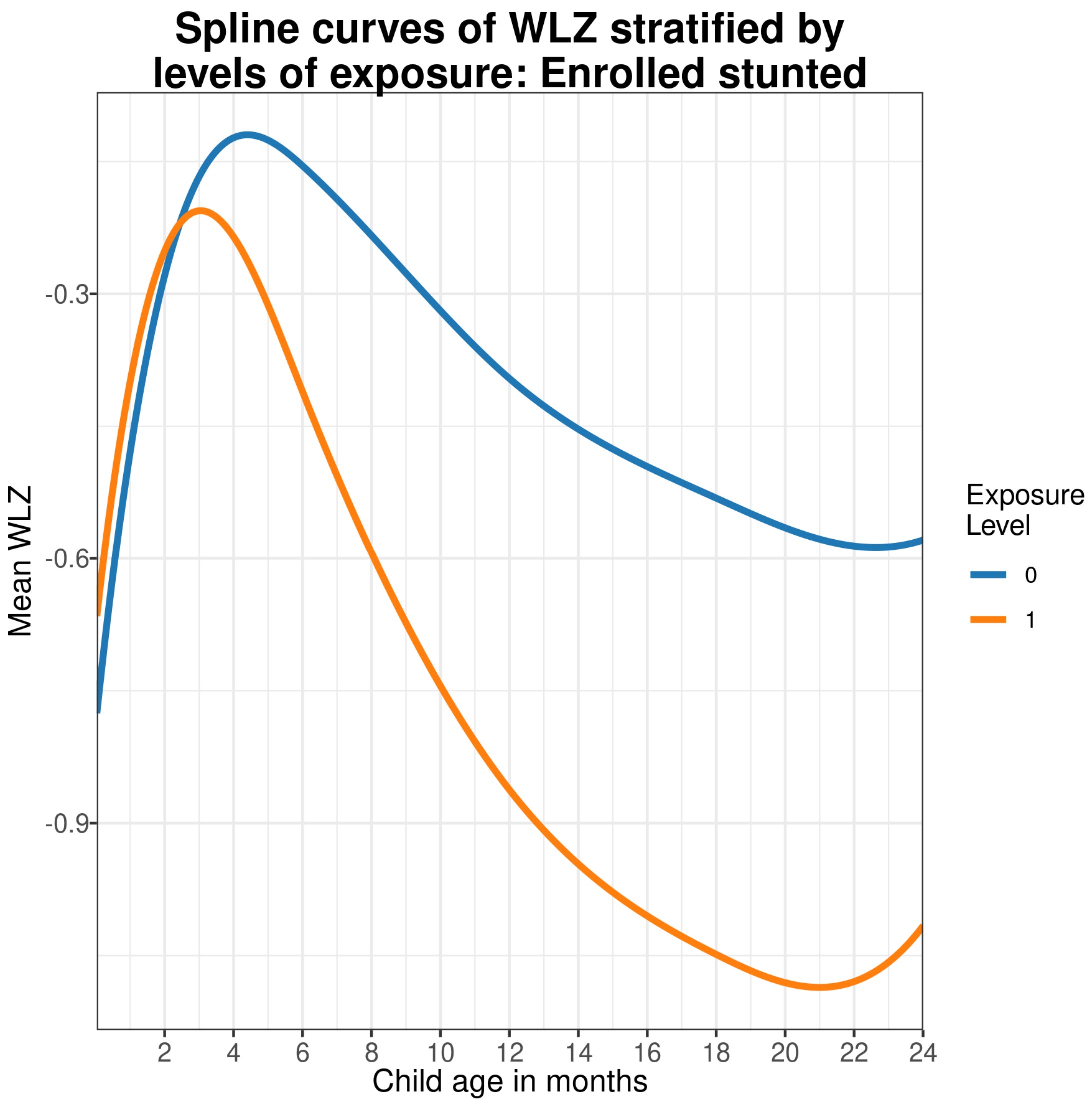

Spline curves of WLZ stratified by
levels of exposure: Enrolled stunted
Mean WLZ
-0.3
-0.6
-0.9
2
4
6
8
10
12
14
16
18
20
22
24
Child age in months
Exposure
Level
0
1

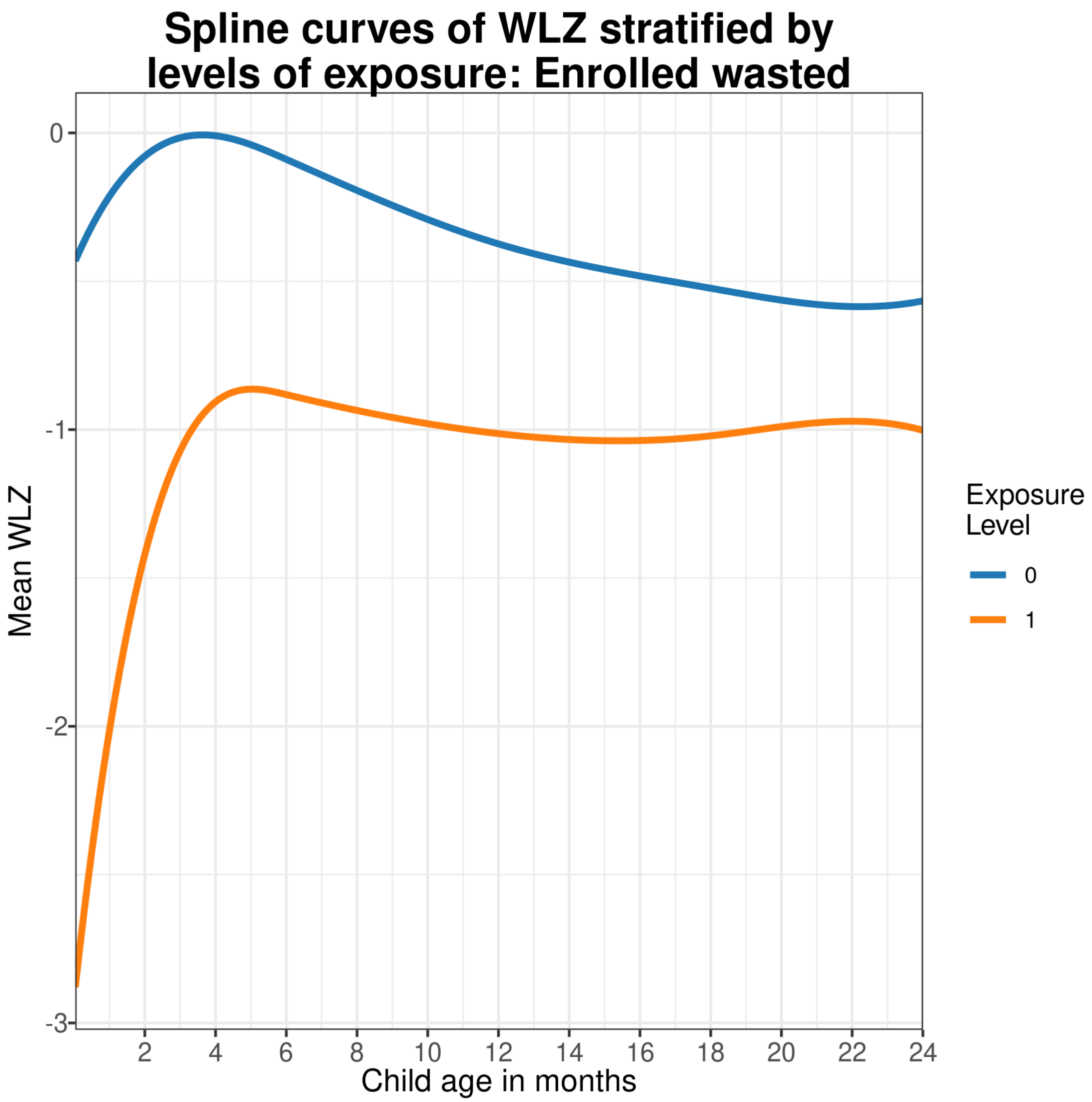

Spline curves of WLZ stratified by levels of exposure: Enrolled wasted
0
-1
-2
-3
Mean WLZ
2
4
6
8
10
12
14
16
18
20
22
24
Child age in months
Exposure Level
0
1

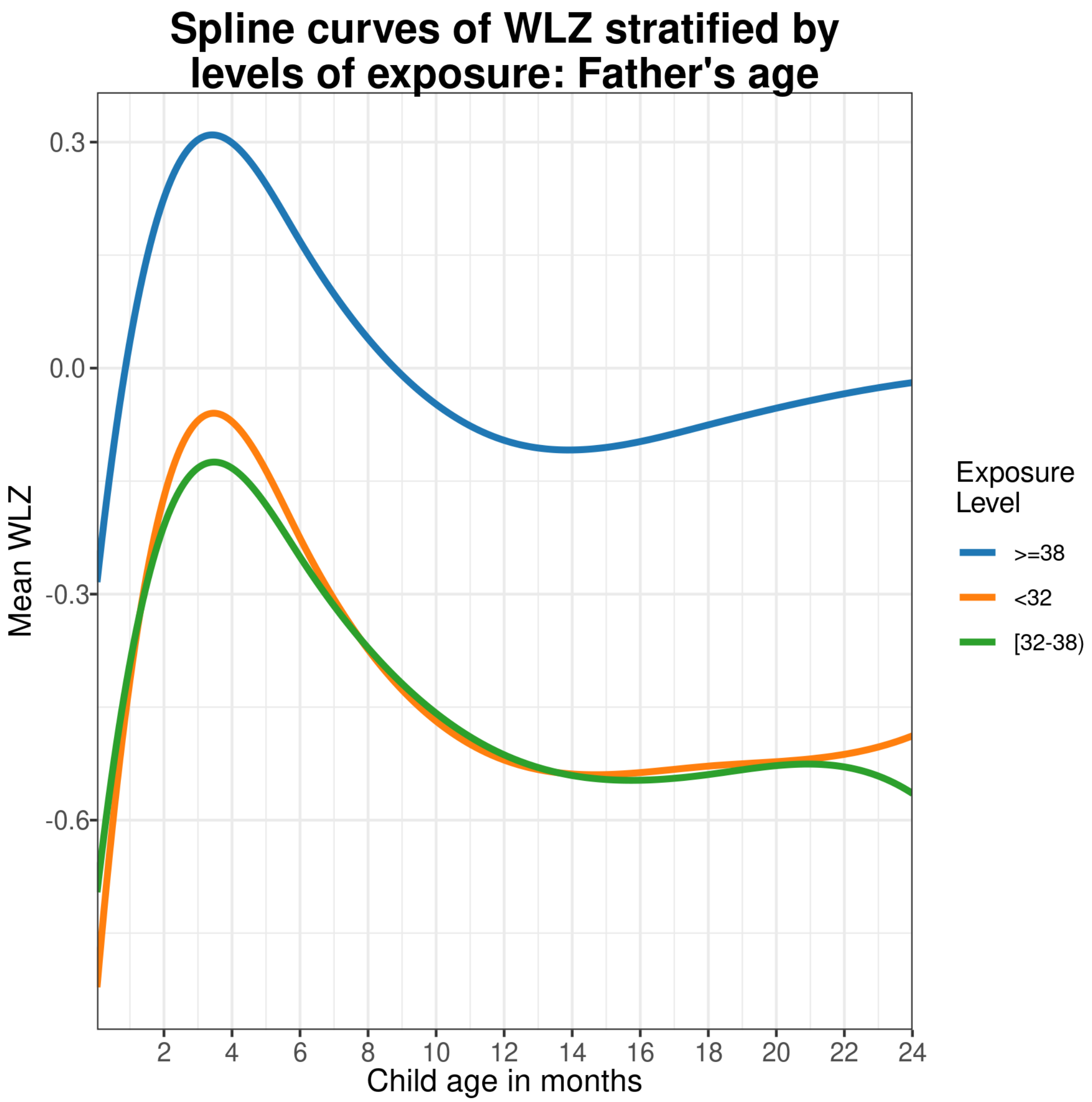

Spline curves of WLZ stratified by
levels of exposure: Father's age
Mean WLZ
0.3
0.0
-0.3
-0.6
2
4
6
8
10
12
14
16
18
20
22
24
Child age in months
Exposure
Level
>=38
<32
[32-38)

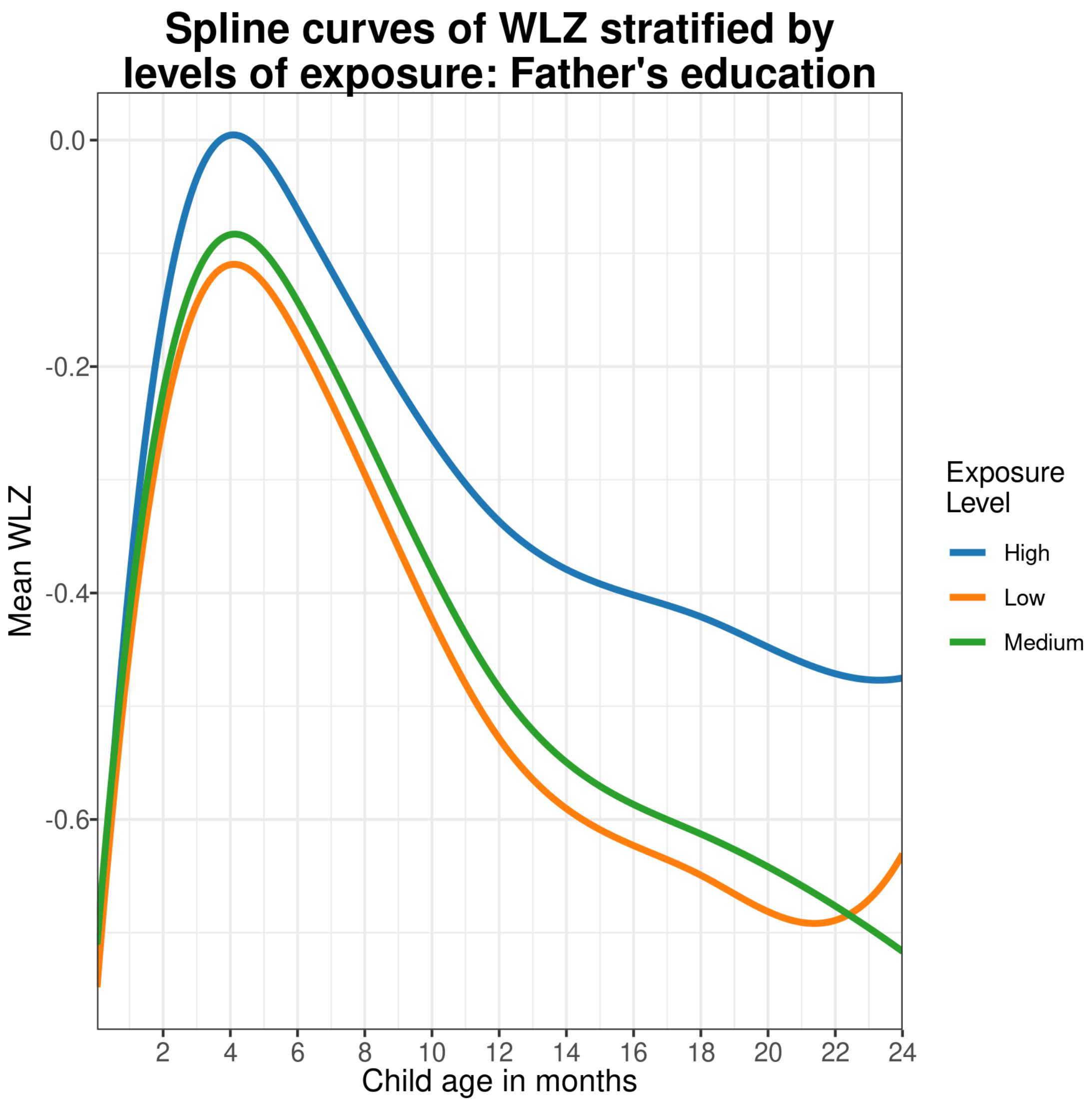
Spline curves of WLZ stratified by
levels of exposure: Father's education
0.0
-0.2
-0.4
-0.6
Mean WLZ
2
4
6
8
10
12
14
16
18
20
22
24
Child age in months
Exposure
Level
High
Low
Medium

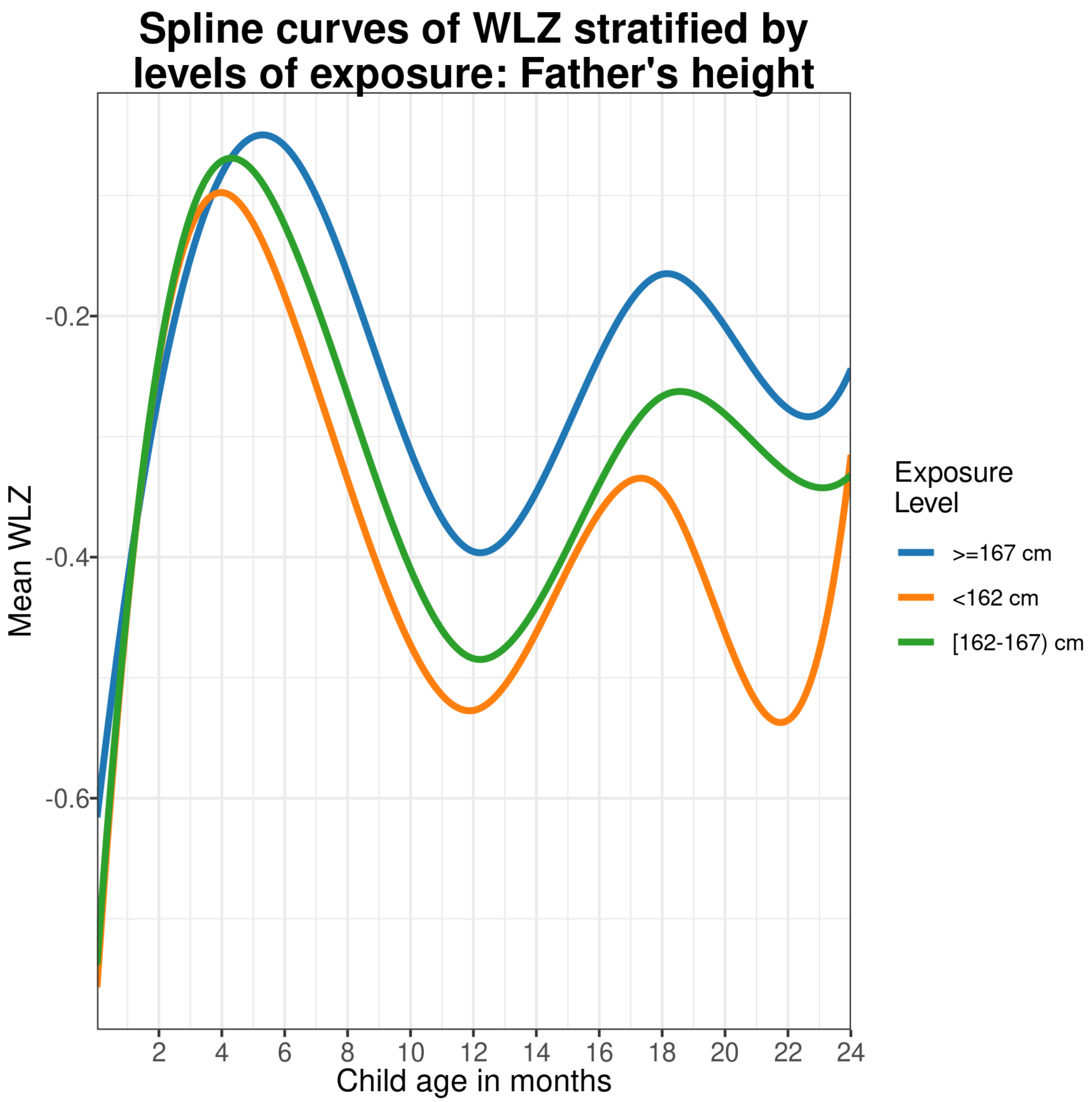
Spline curves of WLZ stratified by
levels of exposure: Father's height
Mean WLZ
-0.2
-0.4
-0.6
2
4
6
8
10
12
14
16
18
20
22
24
Child age in months
Exposure
Level
>=167 cm
<162 cm
[162-167) cm

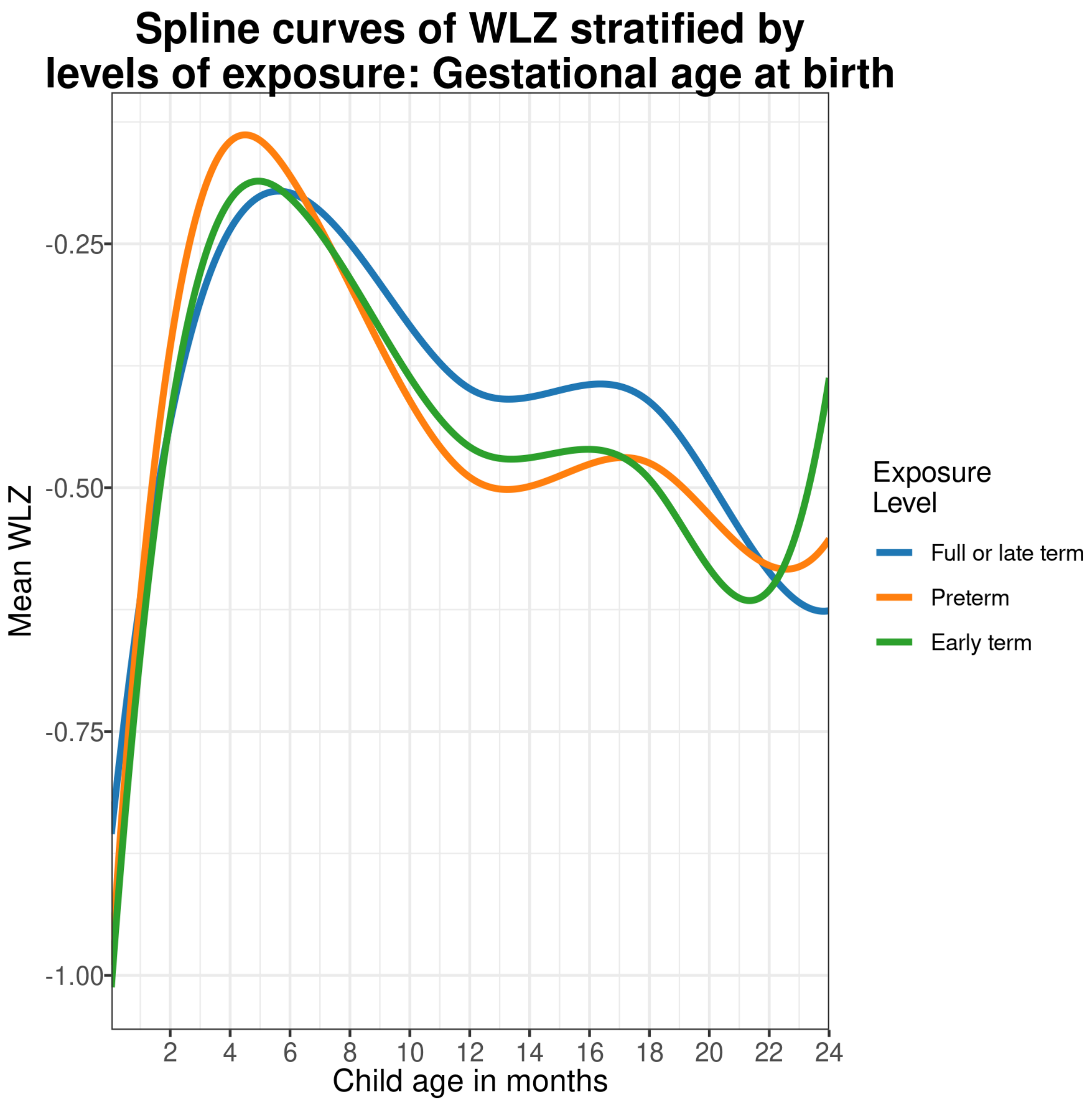
Spline curves of WLZ stratified by
levels of exposure: Gestational age at birth
Mean WLZ
-0.25
-0.50
-0.75
-1.00
2
4
6
8
10
12
14
16
18
20
22
24
Child age in months
Exposure
Level
Full or late term
Preterm
Early term

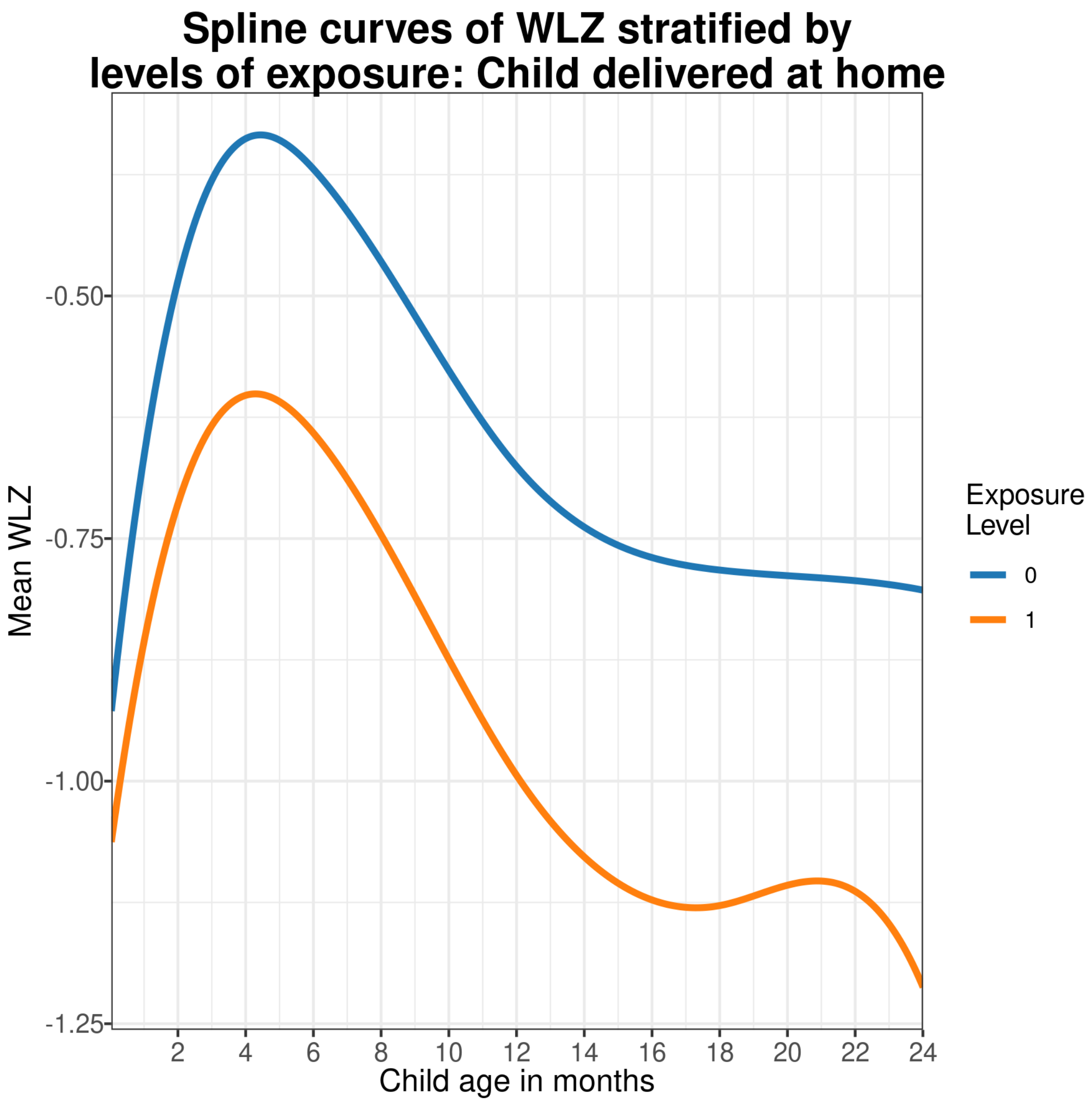
Spline curves of WLZ stratified by
levels of exposure: Child delivered at home
Mean WLZ
-0.50
-0.75
-1.00
-1.25
2
4
6
8
10
12
14
16
18
20
22
24
Child age in months
Exposure
Level
0
1

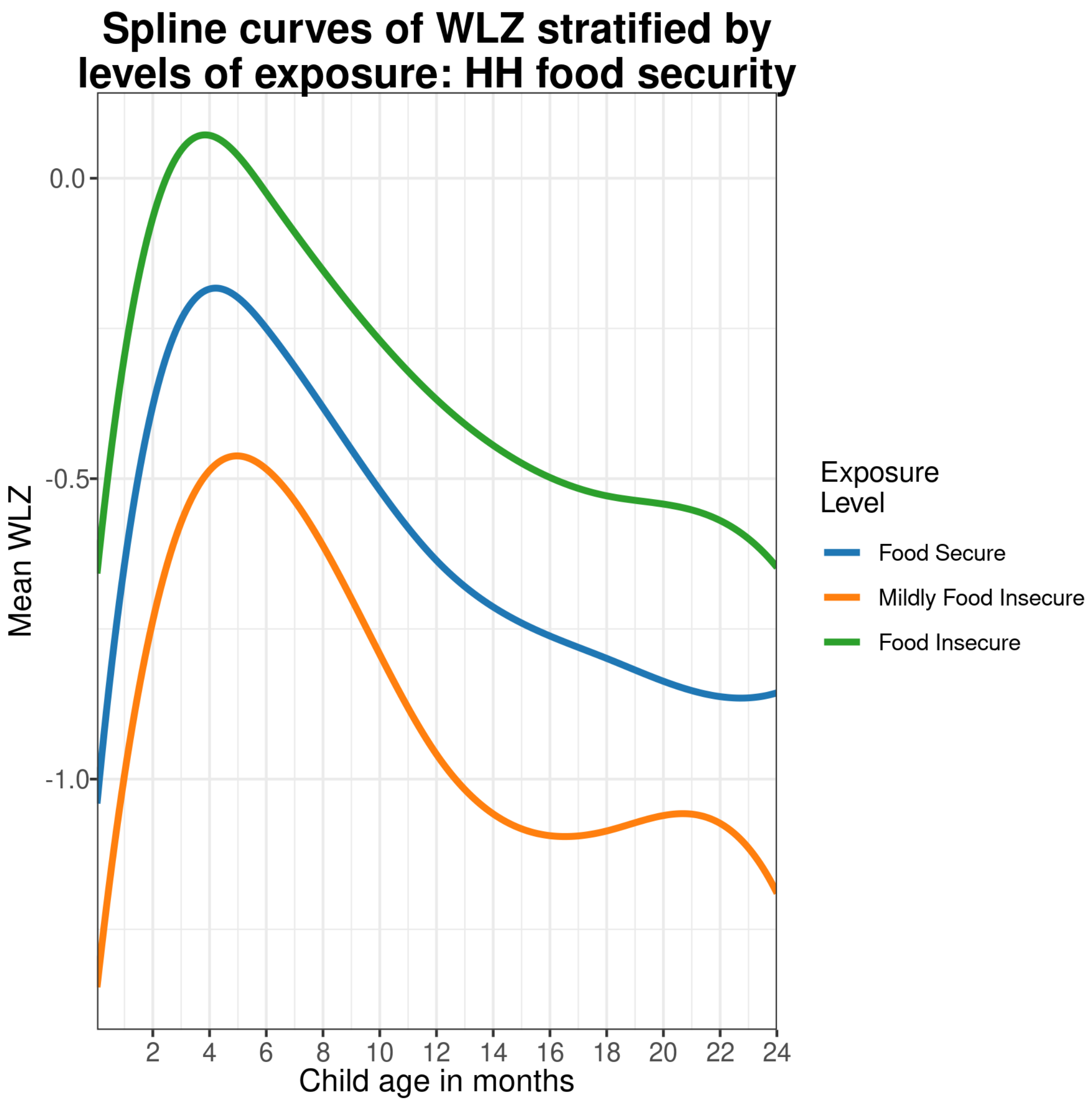
Spline curves of WLZ stratified by levels of exposure: HH food security
Mean WLZ
0.0
-0.5
-1.0
2
4
6
8
10
12
14
16
18
20
22
24
Child age in months
Exposure Level
Food Secure
Mildly Food Insecure
Food Insecure

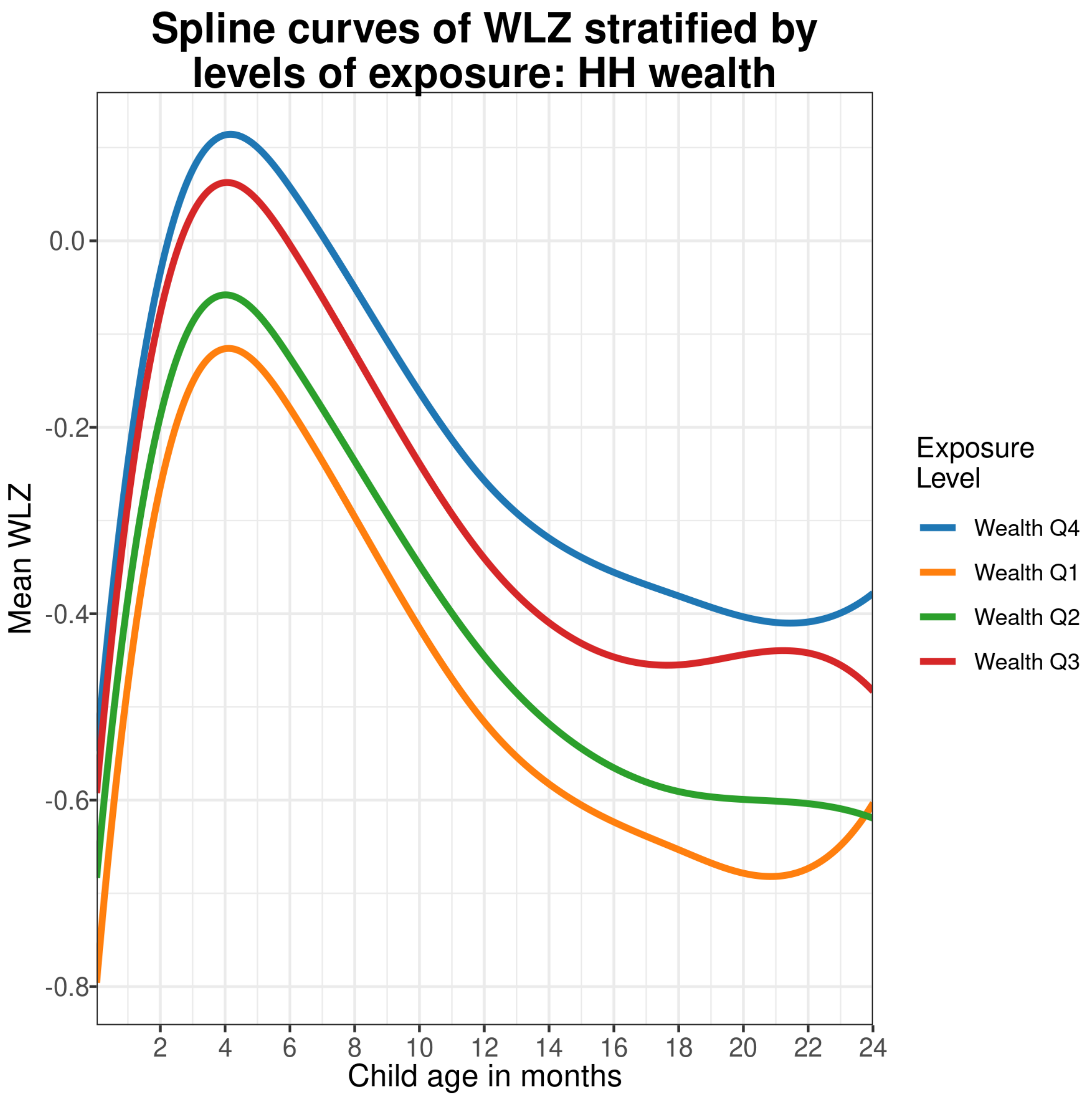

Spline curves of WLZ stratified by
levels of exposure: HH wealth
Mean WLZ
0.0
-0.2
-0.4
-0.6
-0.8
2
4
6
8
10
12
14
16
18
20
22
24
Child age in months
Exposure
Level
Wealth Q4
Wealth Q1
Wealth Q2
Wealth Q3

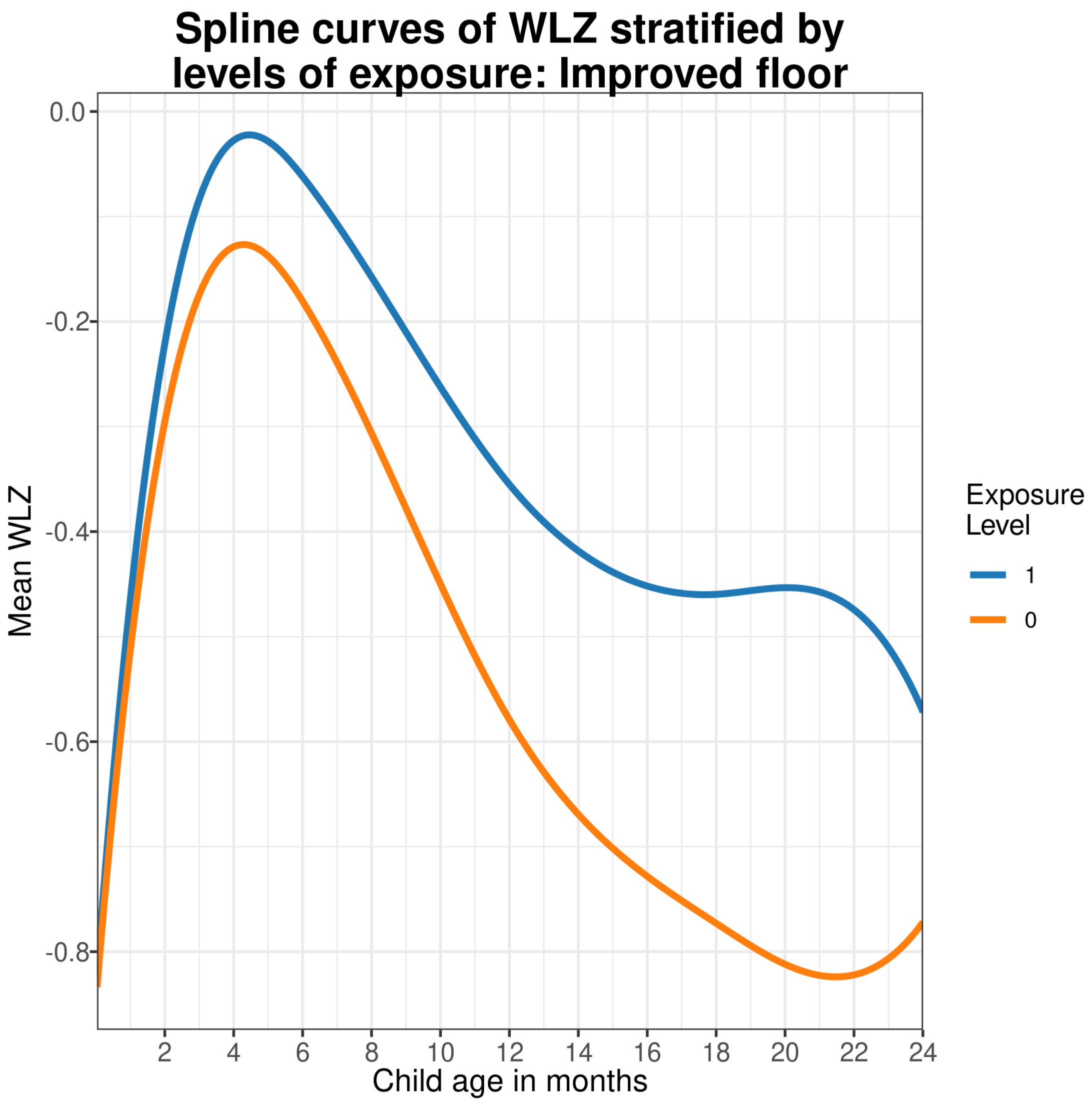
Spline curves of WLZ stratified by
levels of exposure: Improved floor
0.0
-0.2
-0.4
-0.6
-0.8
Mean WLZ
2
4
6
8
10
12
14
16
18
20
22
24
Child age in months
Exposure
Level
1
0

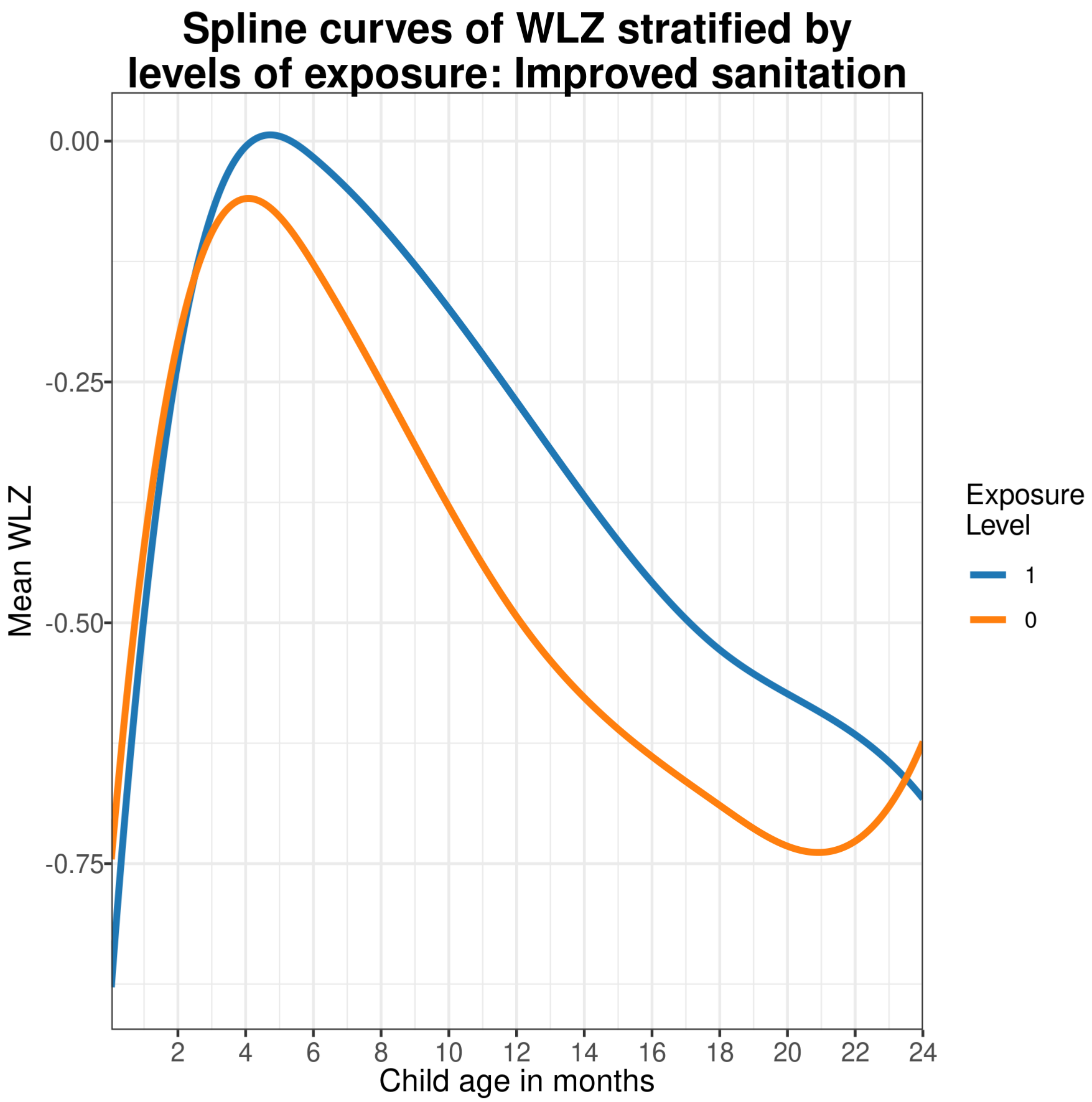

Spline curves of WLZ stratified by
levels of exposure: Improved sanitation
0.00
-0.25
-0.50
-0.75
Mean WLZ
2
4
6
8
10
12
14
16
18
20
22
24
Child age in months
Exposure
Level
1
0

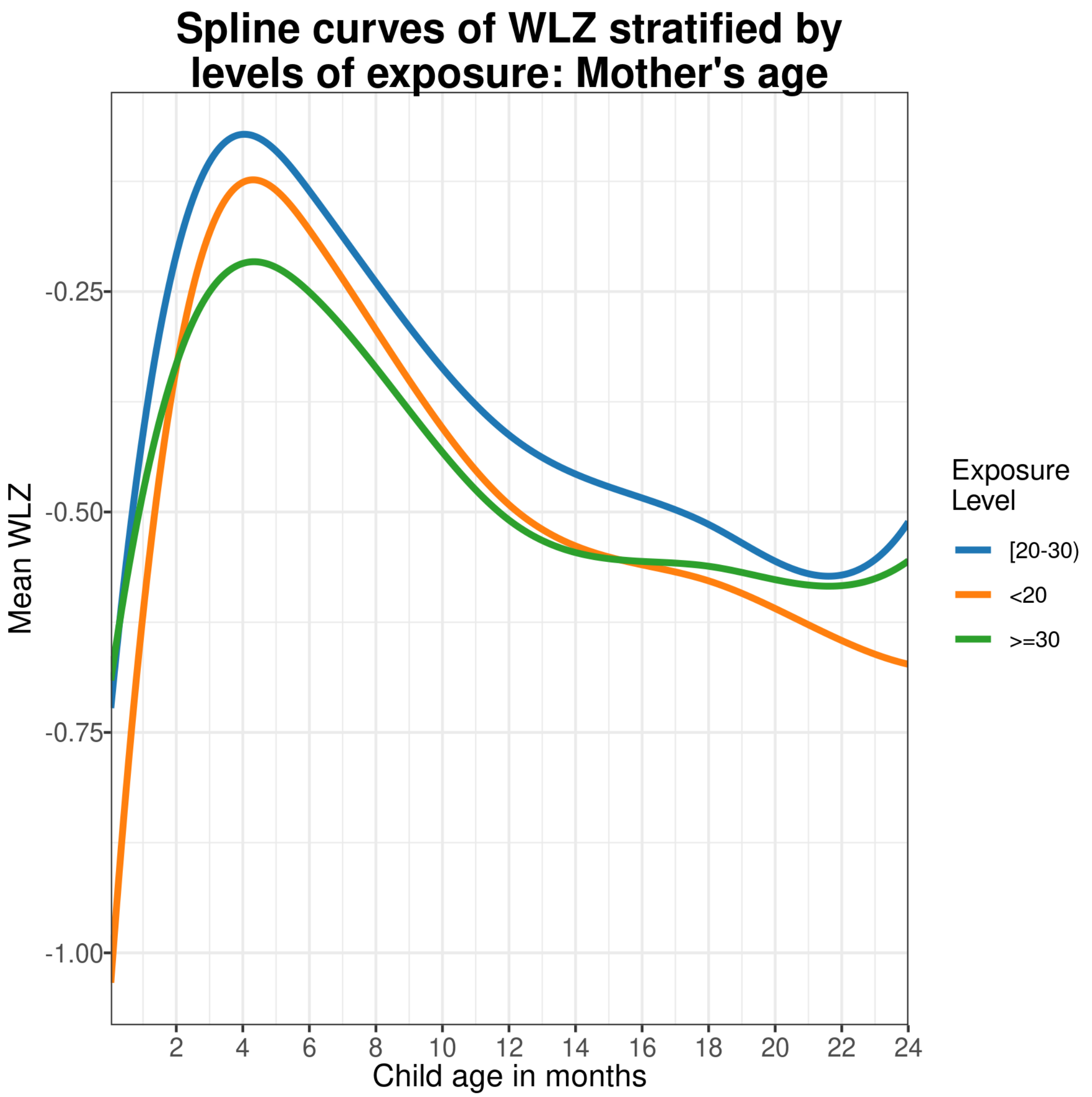

Spline curves of WLZ stratified by levels of exposure: Mother's age
Mean WLZ
-0.25
-0.50
-0.75
-1.00
2
4
6
8
10
12
14
16
18
20
22
24
Child age in months
Exposure Level
[20-30)
<20
>=30

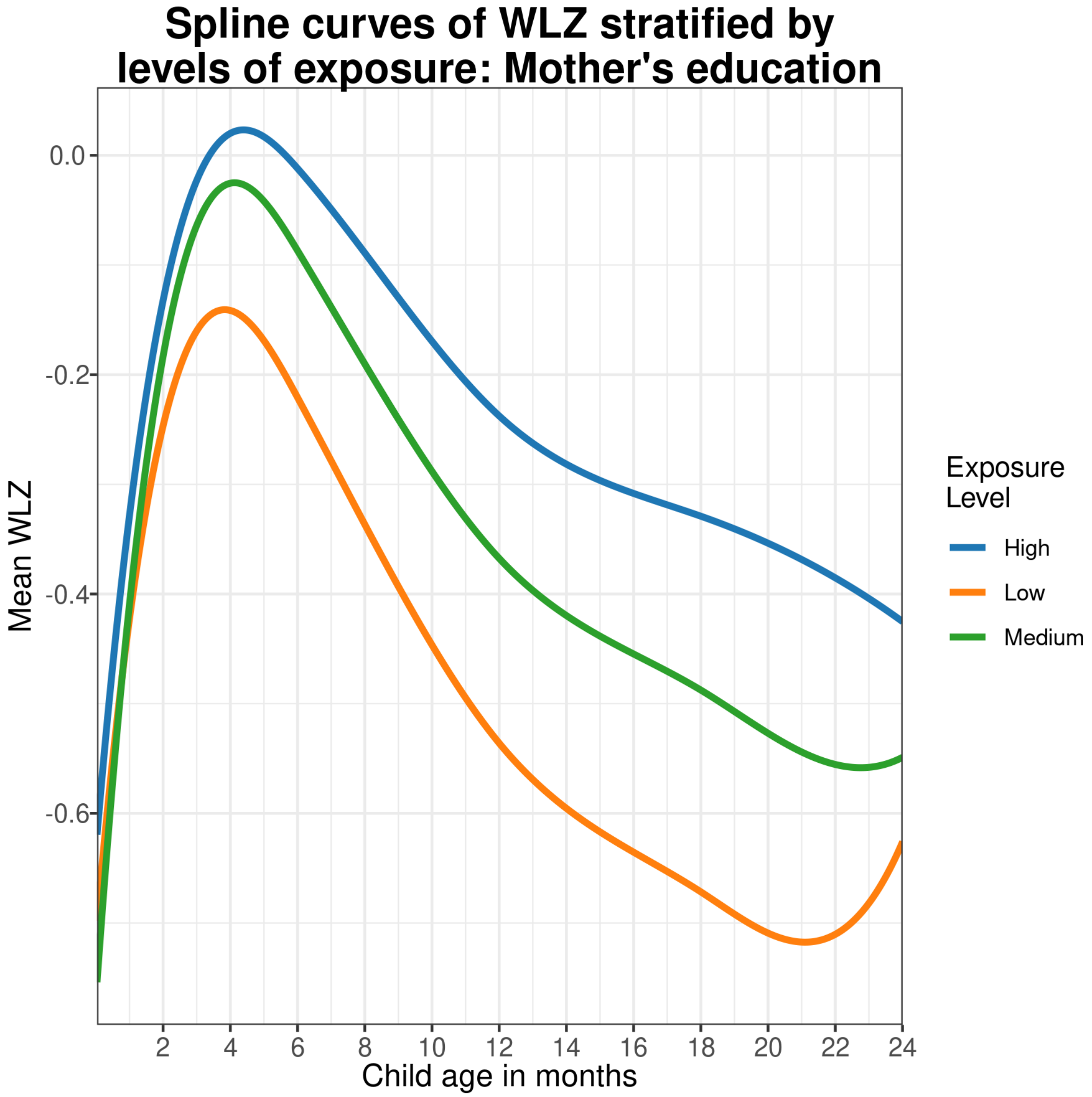

Spline curves of WLZ stratified by
levels of exposure: Mother's education
Mean WLZ
0.0
-0.2
-0.4
-0.6
2
4
6
8
10
12
14
16
18
20
22
24
Child age in months
Exposure
Level
High
Low
Medium

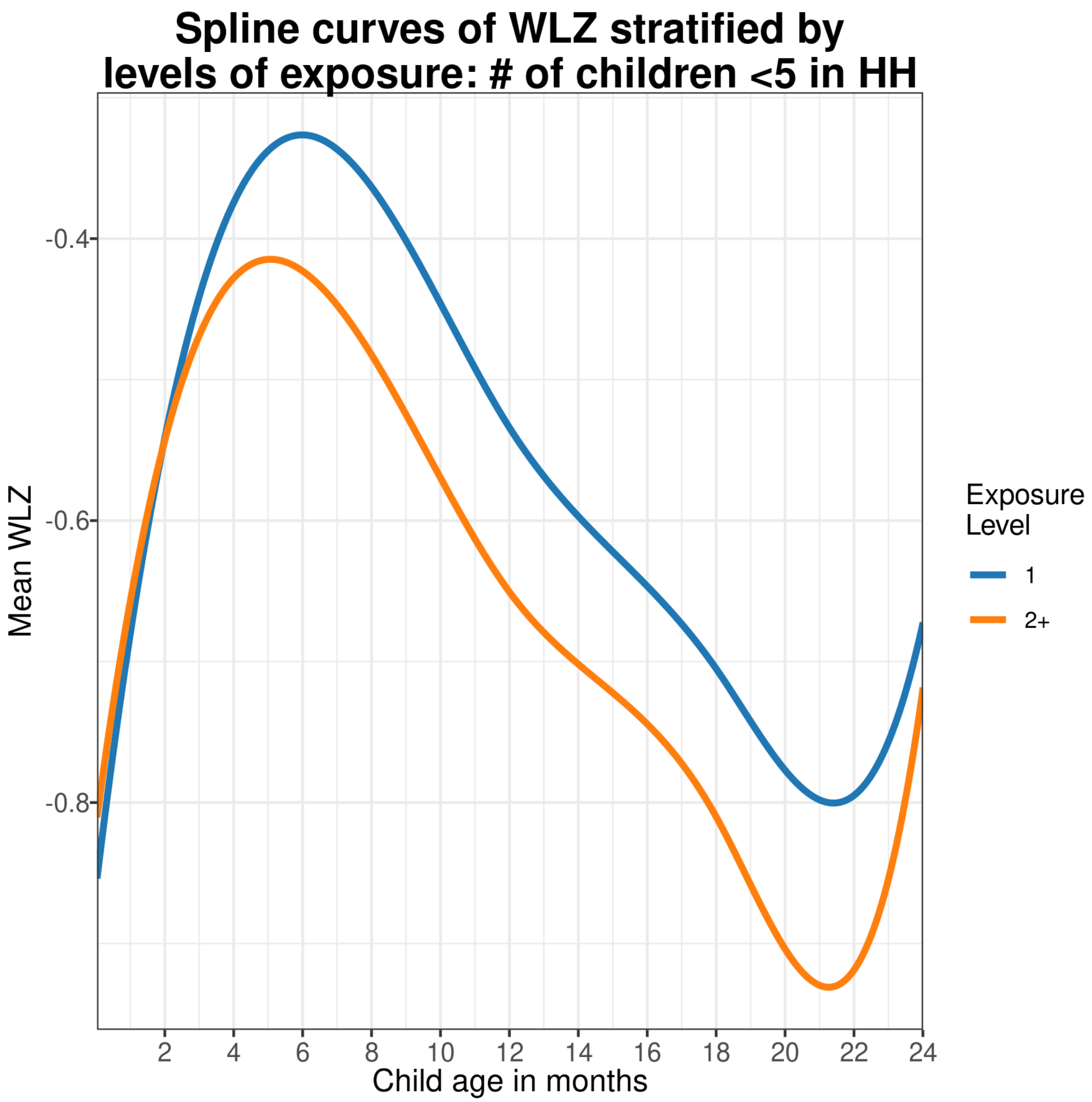
Spline curves of WLZ stratified by
levels of exposure: # of children <5 in HH
-0.4
-0.6
-0.8
Mean WLZ
2
4
6
8
10
12
14
16
18
20
22
24
Child age in months
Exposure
Level
1
2+

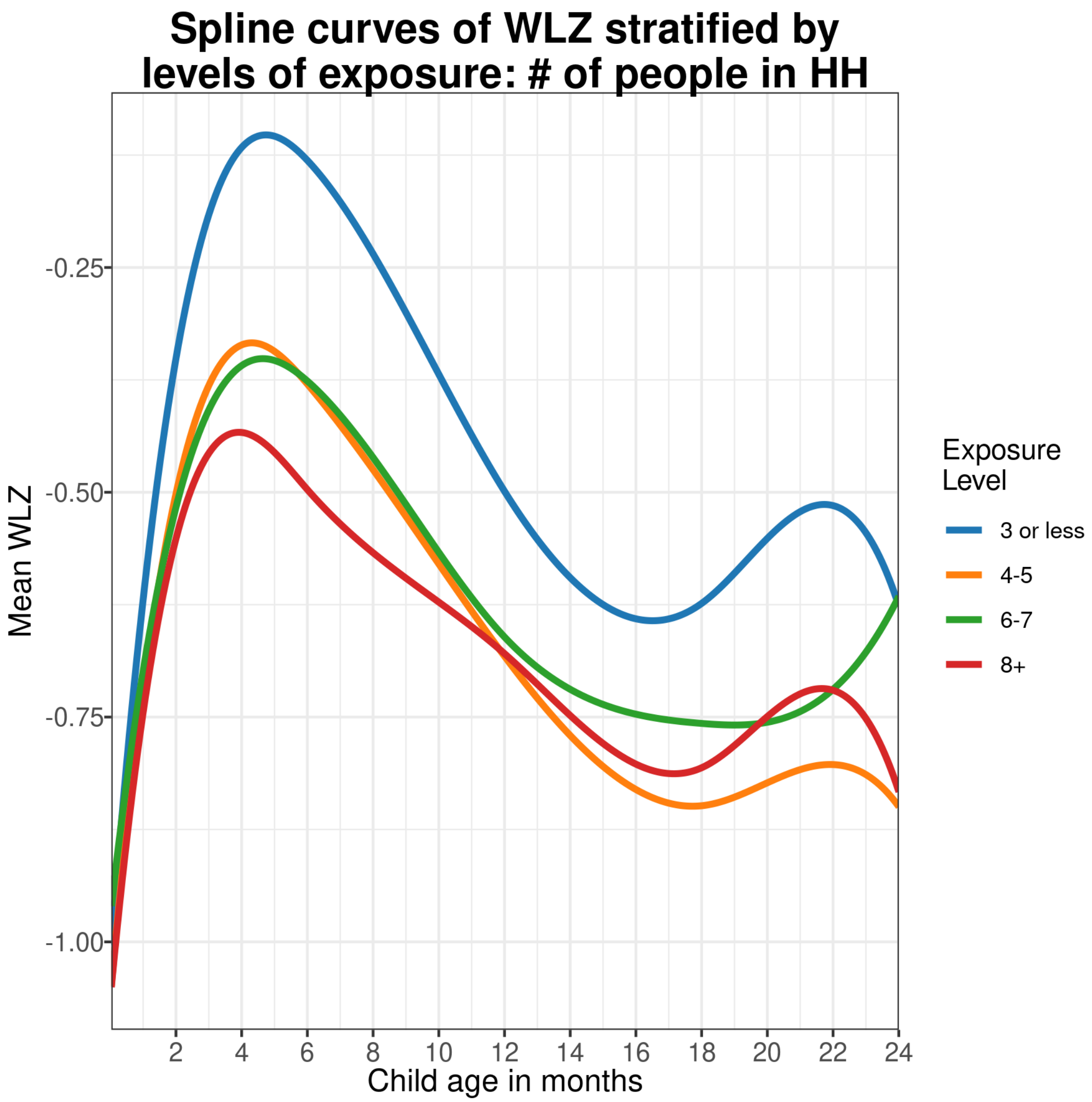
Spline curves of WLZ stratified by levels of exposure: # of people in HH
Mean WLZ
-0.25
-0.50
-0.75
-1.00
2
4
6
8
10
12
14
16
18
20
22
24
Child age in months
Exposure Level
3 or less
4-5
6-7
8+

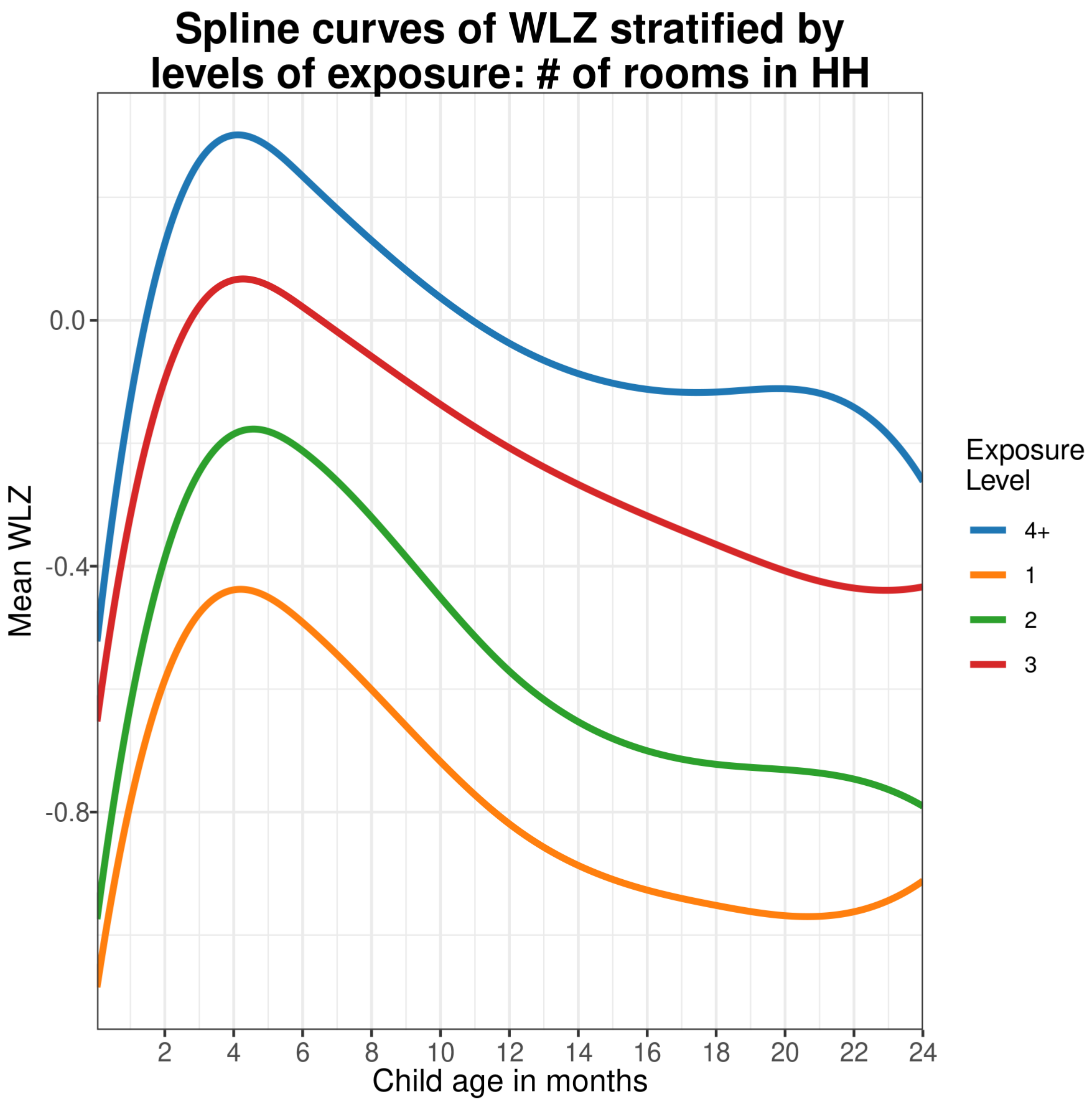
Spline curves of WLZ stratified by
levels of exposure: # of rooms in HH
Mean WLZ
0.0
-0.4
-0.8
2
4
6
8
10
12
14
16
18
20
22
24
Child age in months
Exposure
Level
4+
1
2
3

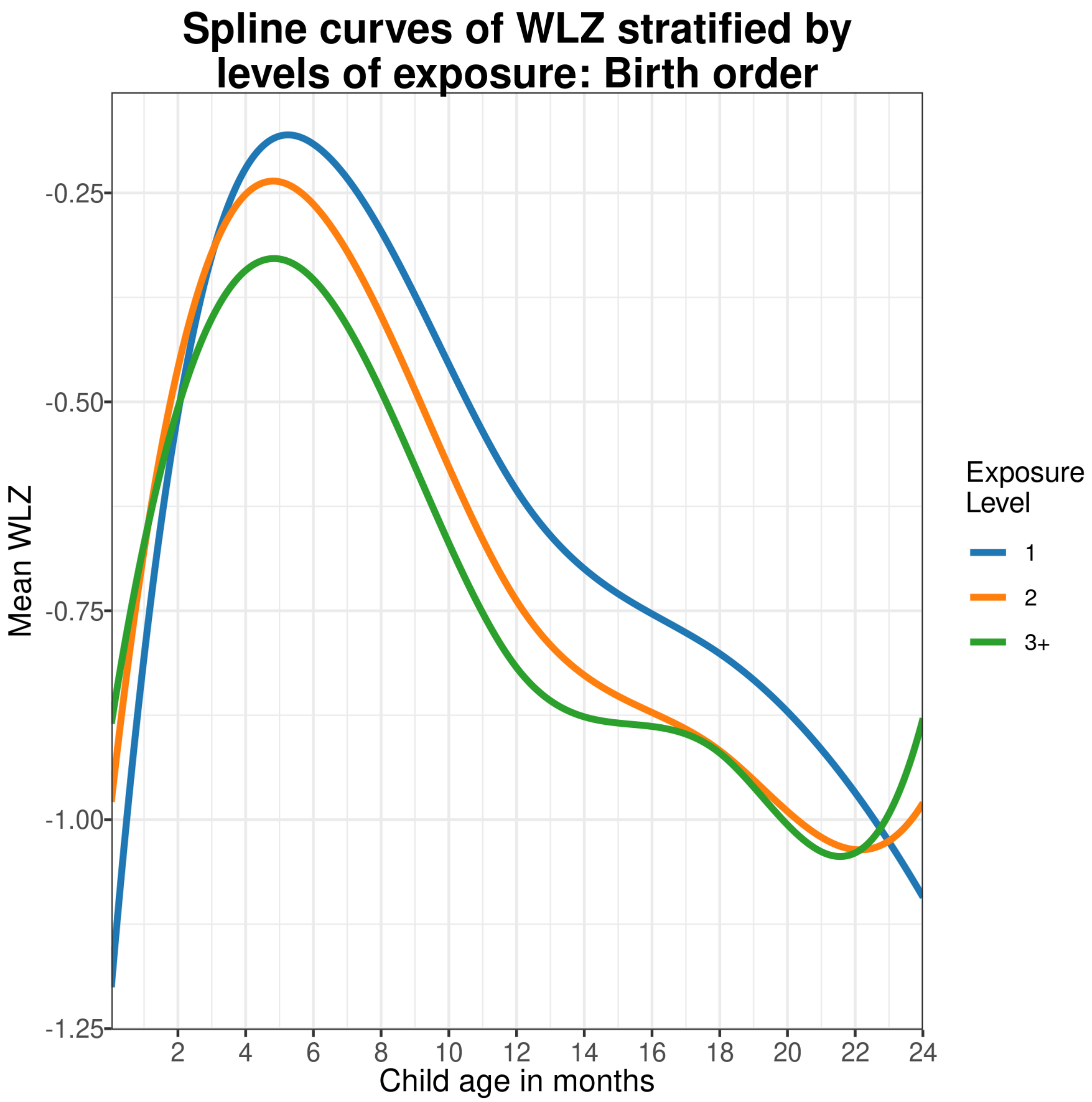
Spline curves of WLZ stratified by
levels of exposure: Birth order
Mean WLZ
-0.25
-0.50
-0.75
-1.00
-1.25
2
4
6
8
10
12
14
16
18
20
22
24
Child age in months
Exposure
Level
1
2
3+

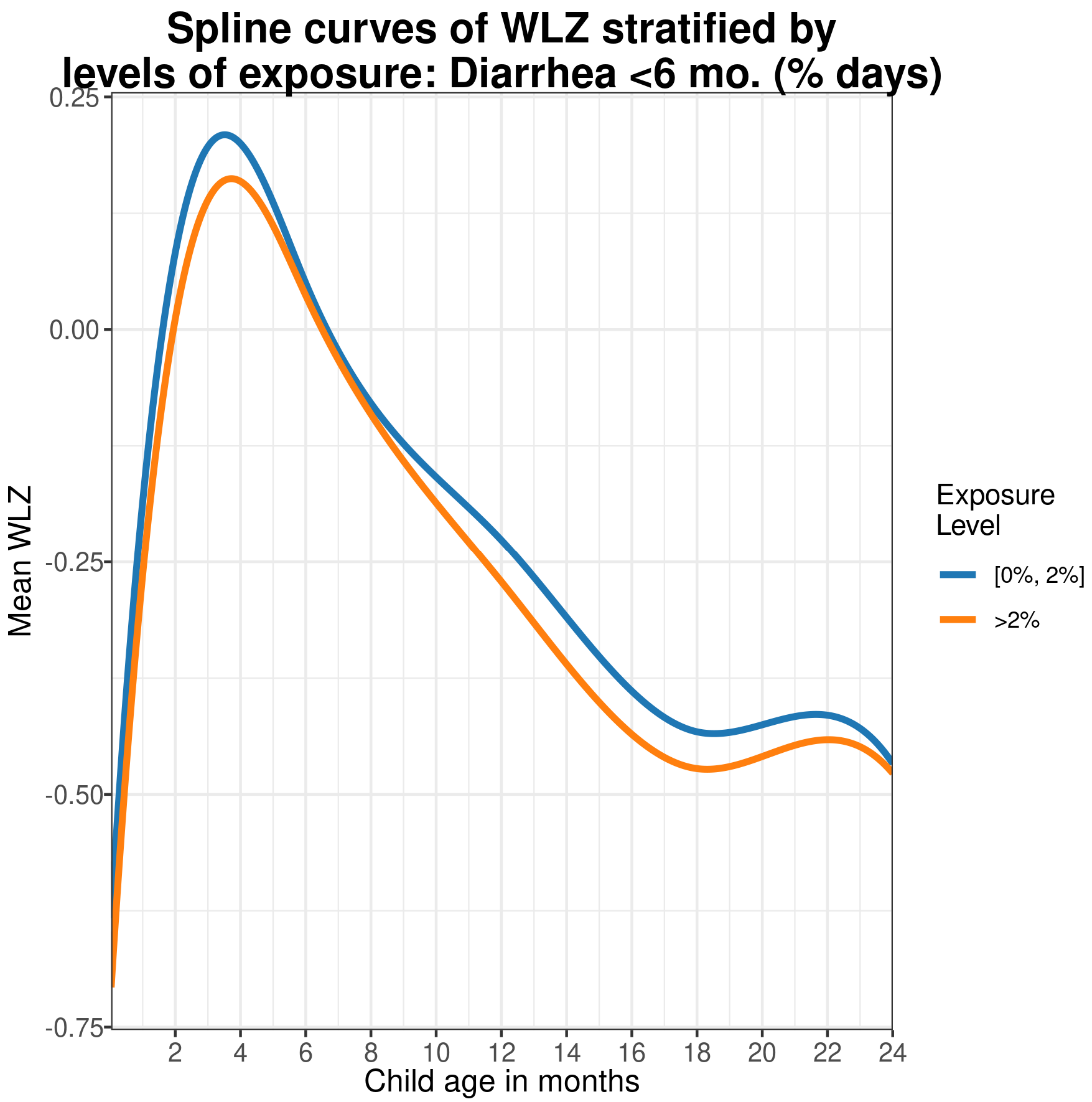

Spline curves of WLZ stratified by
levels of exposure: Diarrhea <6 mo. (% days)
0.25
0.00
-0.25
-0.50
-0.75
Mean WLZ
2
4
6
8
10
12
14
16
18
20
22
24
Child age in months
Exposure
Level
[0%, 2%]
>2%

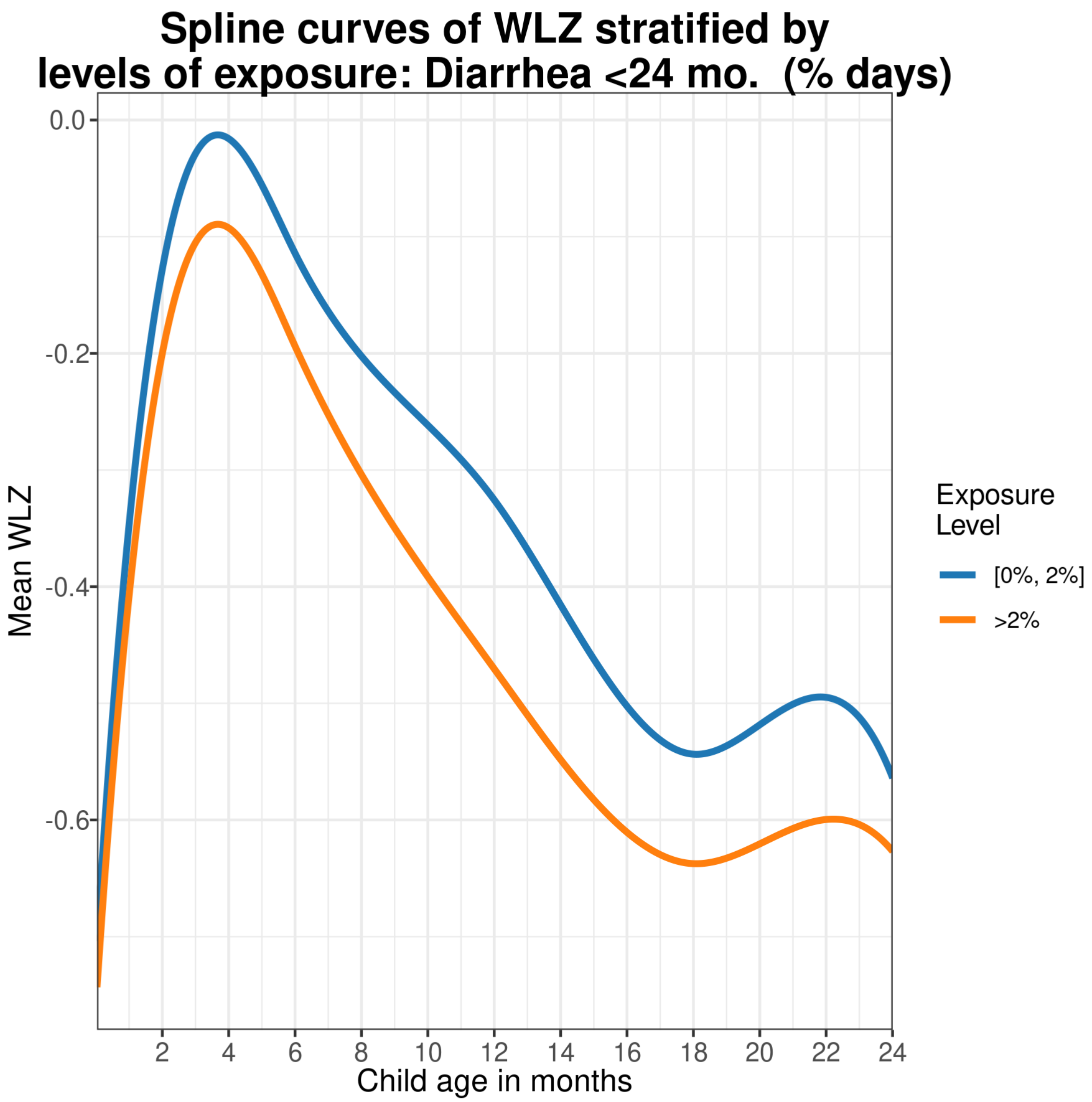
Spline curves of WLZ stratified by
levels of exposure: Diarrhea <24 mo. (% days)
0.0
-0.2
-0.4
-0.6
Mean WLZ
2
4
6
8
10
12
14
16
18
20
22
24
Child age in months
Exposure Level
[0%, 2%]
>2%

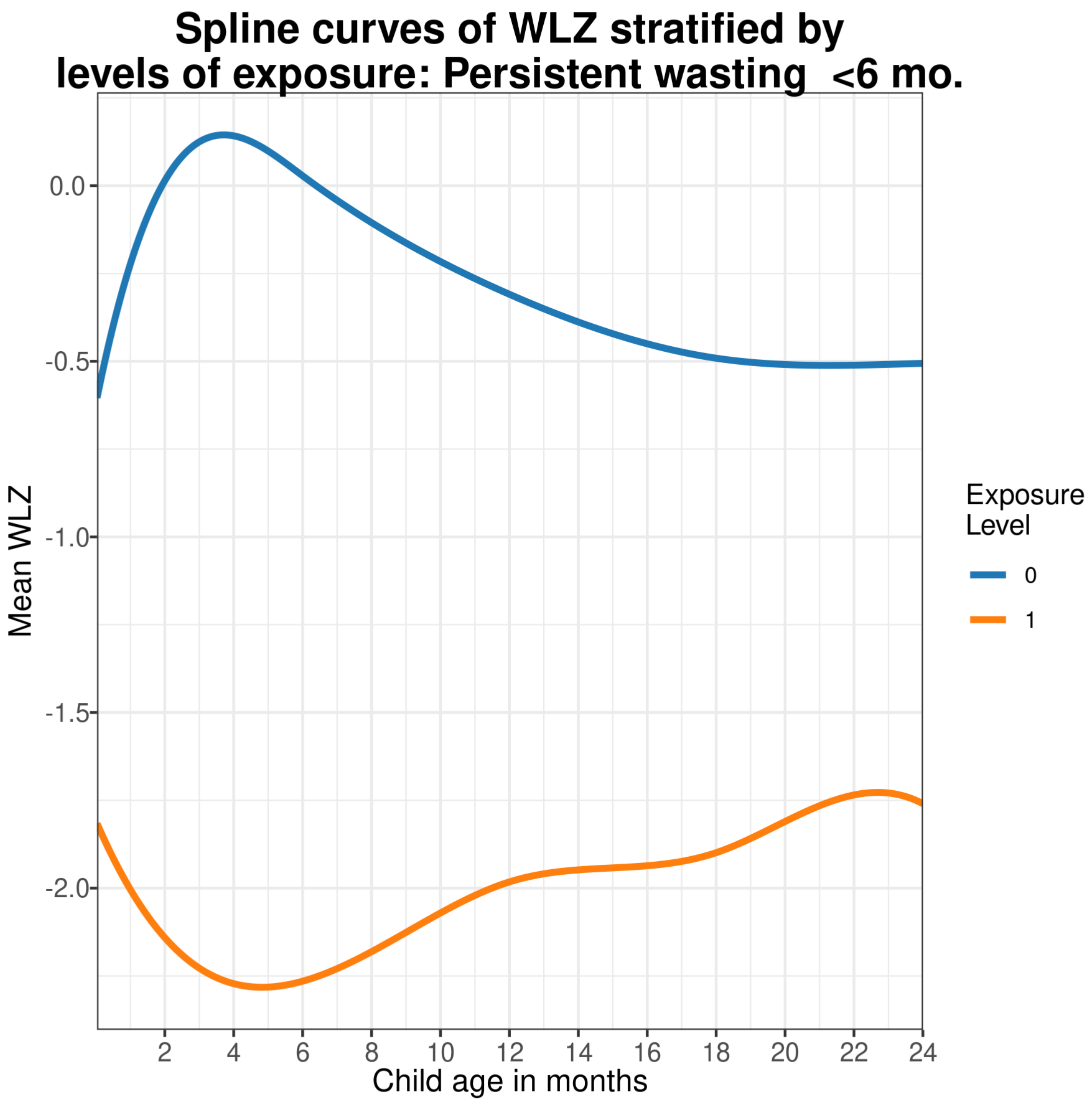
Spline curves of WLZ stratified by
levels of exposure: Persistent wasting <6 mo.
Mean WLZ
0.0
-0.5
-1.0
-1.5
-2.0
Child age in months
2
4
6
8
10
12
14
16
18
20
22
24
Exposure
Level
0
1

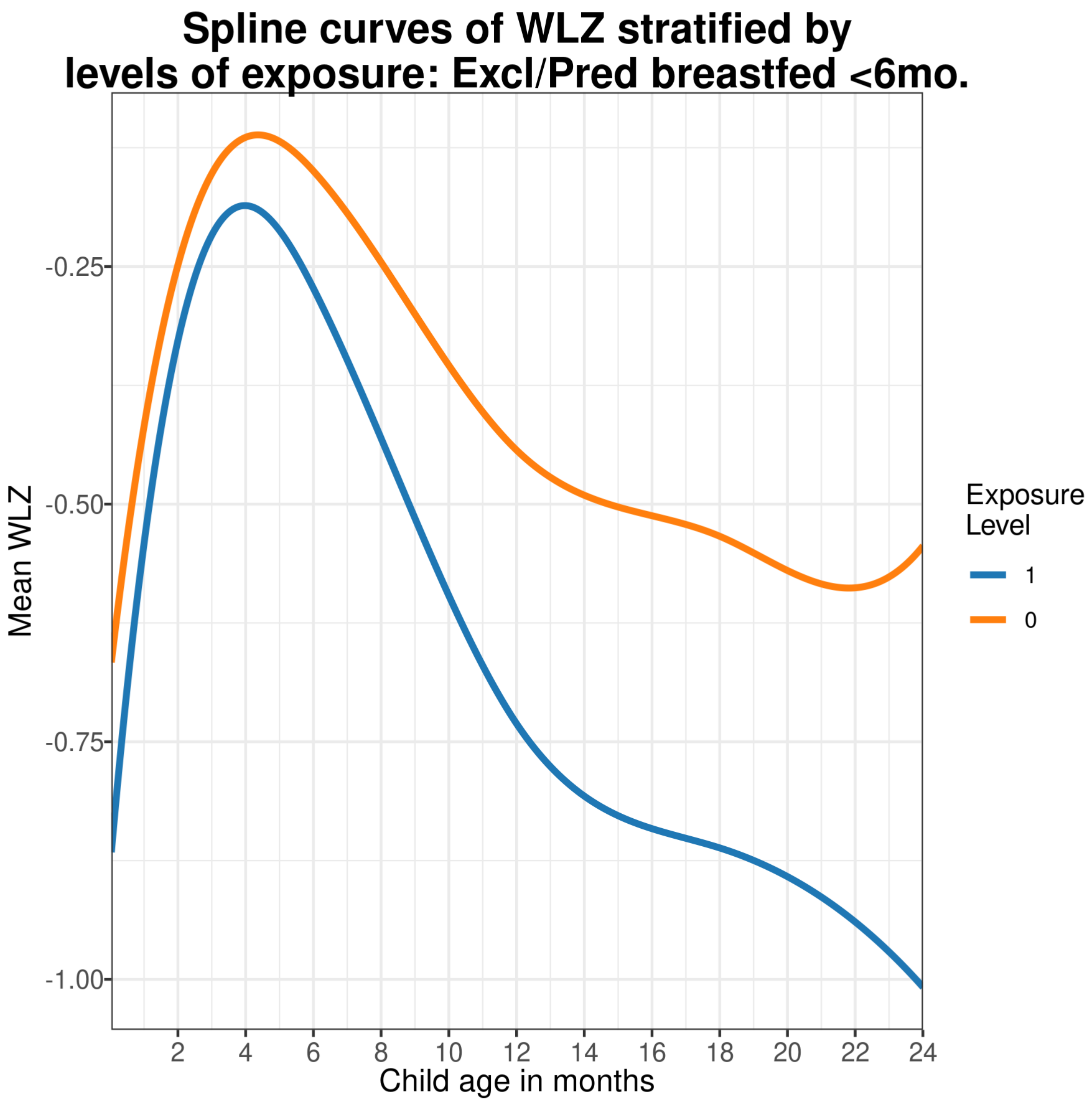
Spline curves of WLZ stratified by
levels of exposure: Excl/Pred breastfed <6mo.
Mean WLZ
-0.25
-0.50
-0.75
-1.00
2
4
6
8
10
12
14
16
18
20
22
24
Child age in months
Exposure
Level
1
0

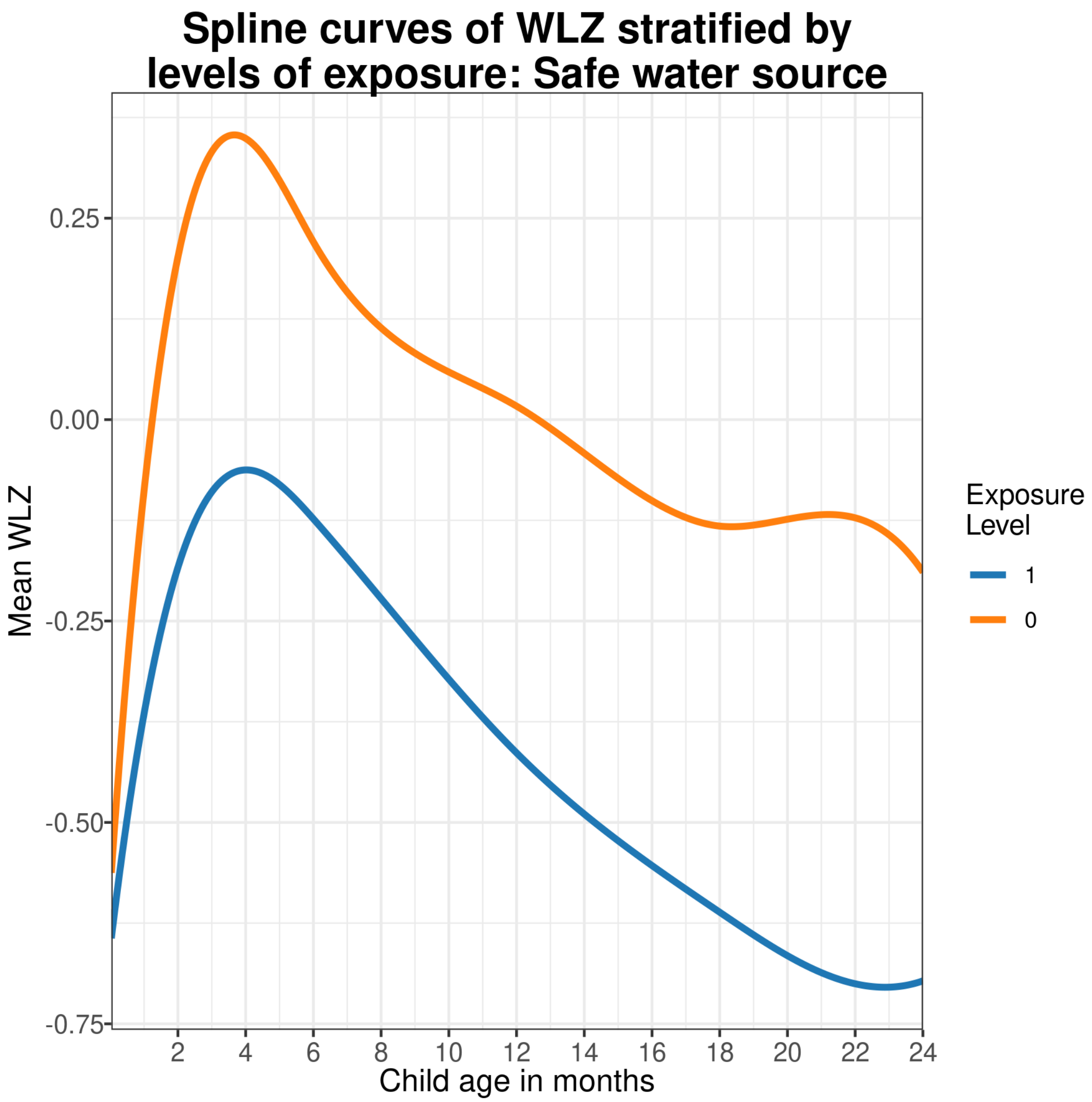
Spline curves of WLZ stratified by
levels of exposure: Safe water source
Mean WLZ
0.25
0.00
-0.25
-0.50
-0.75
2
4
6
8
10
12
14
16
18
20
22
24
Child age in months
Exposure
Level
1
0

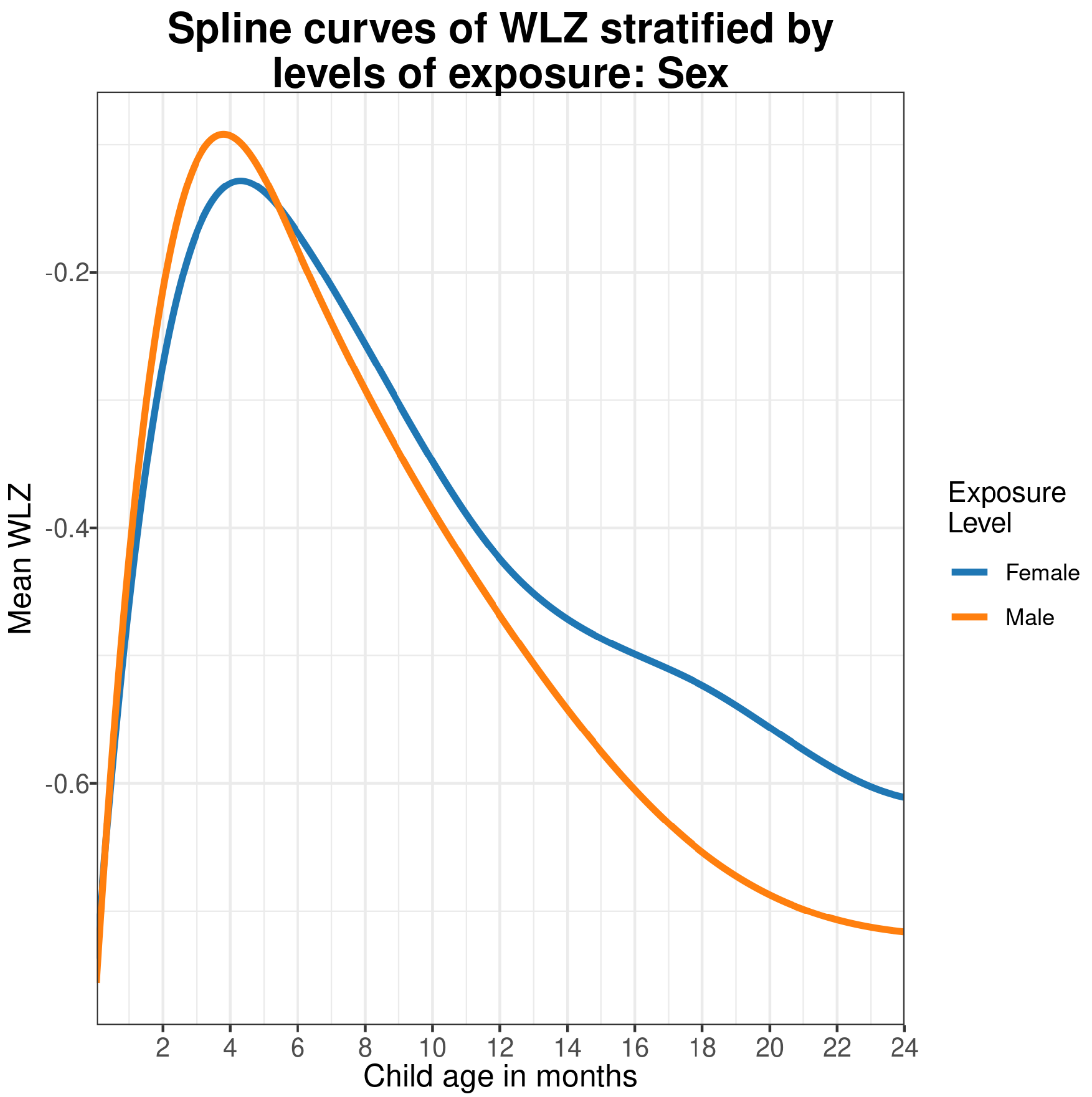

Spline curves of WLZ stratified by
levels of exposure: Sex
Mean WLZ
-0.2
-0.4
-0.6
2
4
6
8
10
12
14
16
18
20
22
24
Child age in months
Exposure
Level
Female
Male

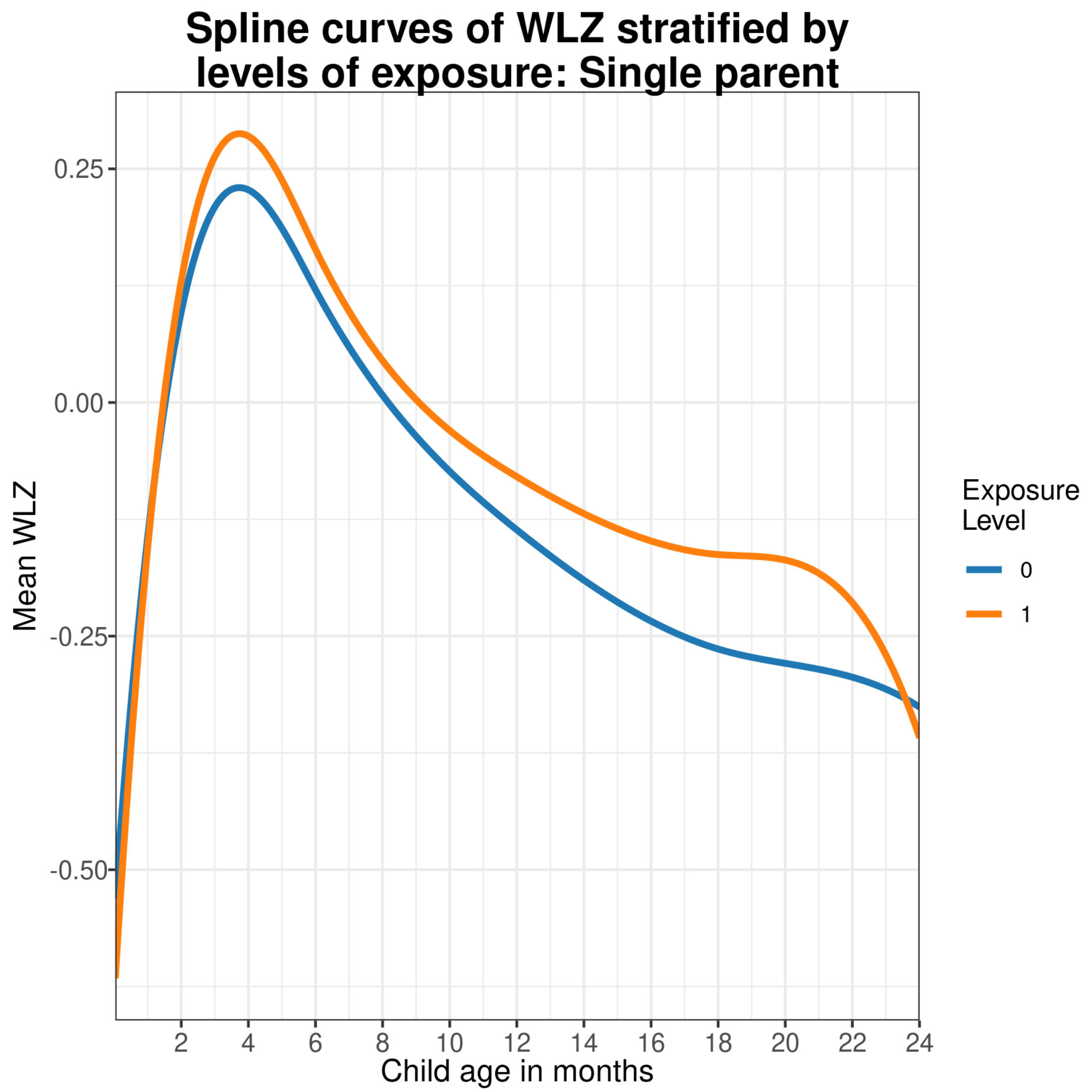
Spline curves of WLZ stratified by
levels of exposure: Single parent
Mean WLZ
0.25
0.00
-0.25
-0.50
2
4
6
8
10
12
14
16
18
20
22
24
Child age in months
Exposure
Level
0
1

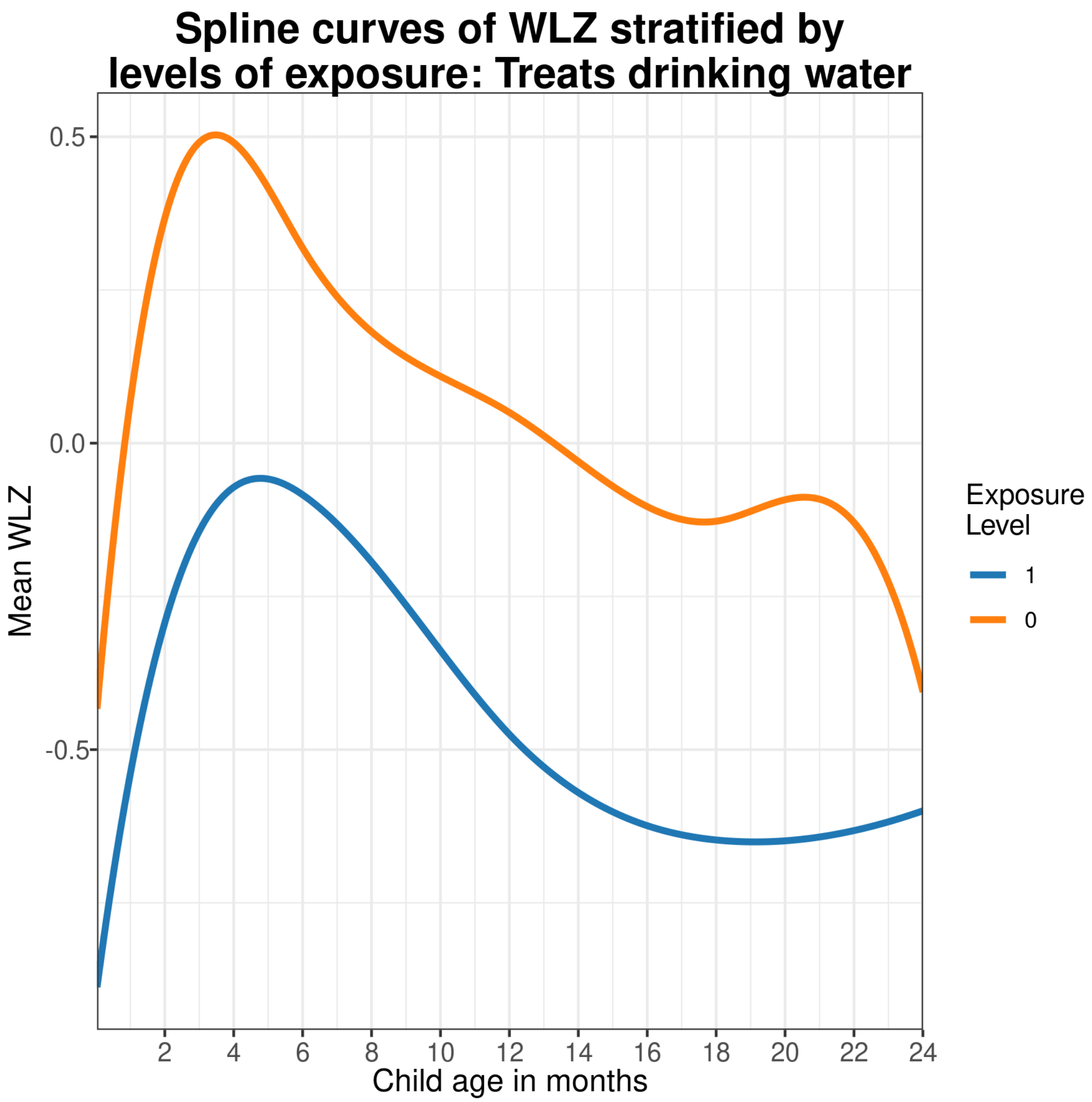
Spline curves of WLZ stratified by
levels of exposure: Treats drinking water
0.5
0.0
-0.5
Mean WLZ
2
4
6
8
10
12
14
16
18
20
22
24
Child age in months
Exposure
Level
1
0

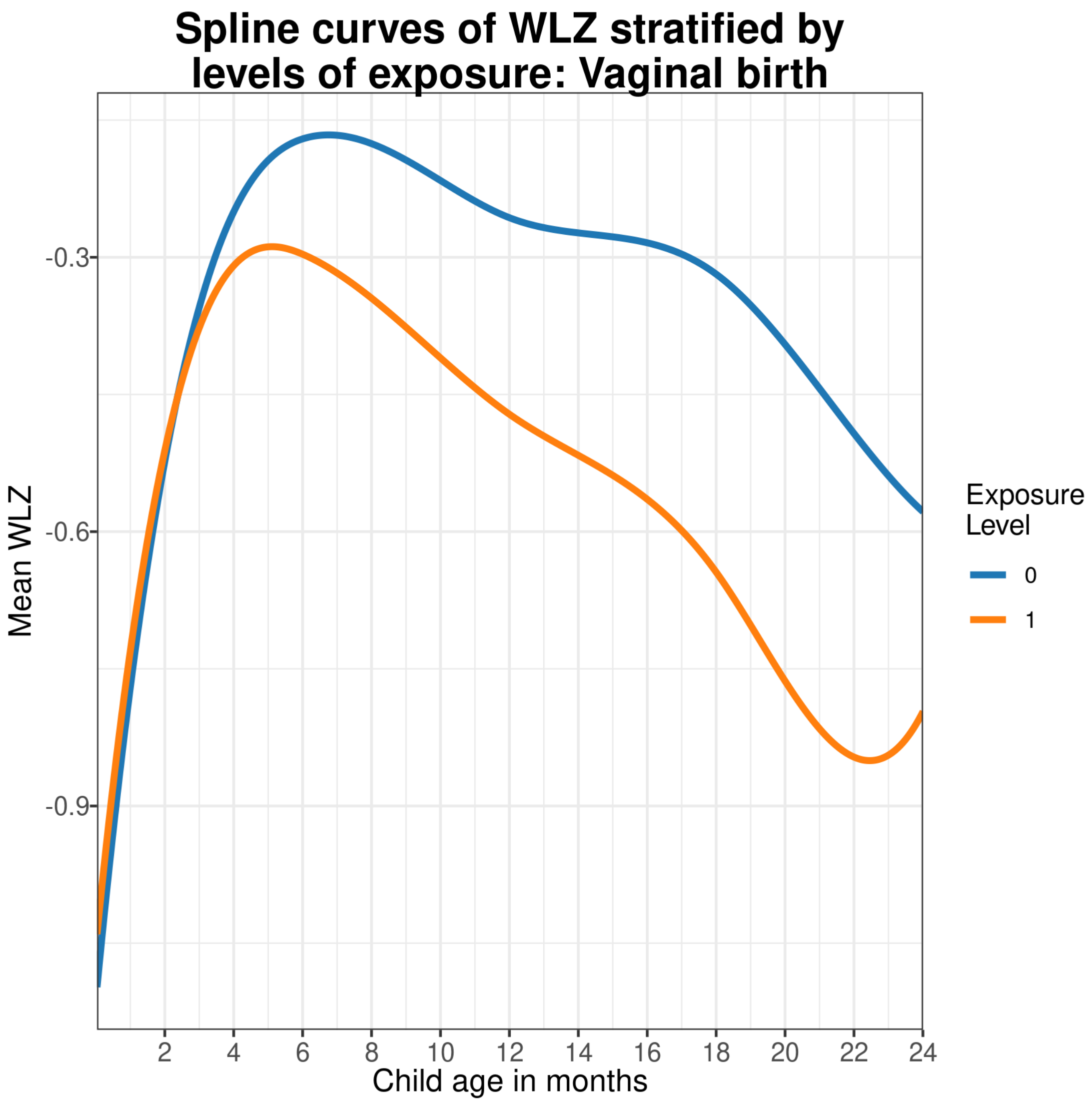


# Supplementary Note 6. Sensitivity to dropping PROBIT trial

## 6.1 Overview

The PROBIT trial, conducted in Belarus, was the only study included conducted in Europe, as opposed to Latin America, South Asia, or sub-Saharan Africa. It was also one of the largest studies, so may have an influential effect on the pooled estimates. Because of differences in income, environmental conditions, and population anthropometry, we reconducted the primary analyses without the PROBIT trial and present the results here. We found that the exclusion of the PROBIT trail did not affect the main inferences presented in the manuscript.

## 6.2 Comparison of attributable differences estimated with and without the PROBIT trial

Because PROBIT was the only European study, we also conducted a sensitivity analysis as to the effect of removing PROBIT on attributable differences at 24 months (within the exposures measures during the PROBIT trial). Other than for the estimated associations between father's height and child Z-scores at 24 months (which was measured in few other studies), PROBIT is not highly influential.

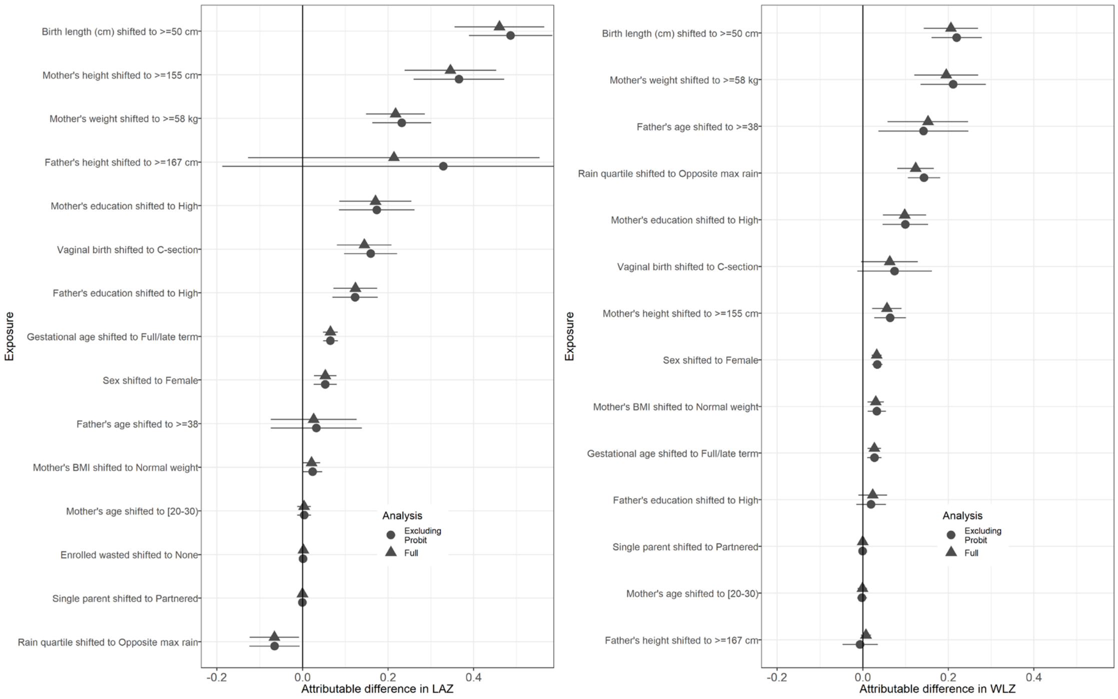


# Supplementary Note 7 Average treatment effect and relative risk plots

The primary parameter presented in the "Causes and Consequences of Child Growth Failure in Low- and Middle-income Countries" publications is the population intervention effect (PIE), the change in mean population Z-score if a exposure is shifted to the lowest-risk level, which combines the increase of risk associated with an exposure with the prevalence of the exposure in the population. This page of the supporting information presents plots for pooled relative risks (RR) and average treatment effects (ATE) for each level of each exposure (all the estimates shown in "Extended Data Figure 2. Heatmap of significance and direction across exposure-outcome combinations", but presented here with point estimates and confidence intervals). Relative risk are reported for binary CGF outcomes (stunting, wasting, underweight, severe stunting, severe wasting, and severe underweight) and ATEs (mean differences) are reported for continuous Z-score outcomes.

The primary purpose of this page is to present the pooled point estimates and confidence intervals for all of the results summarized in the Extended Data Figure 2 heatmap, and to allow the reader to compare the levels of risk across categorical exposures. The population intervention effects and attributable
fraction presented in the primary manuscript show the result when shifting the exposure distribution to

the low-risk reference levels, but the results presented here allow for comparing other exposure levels, like food secure versus moderately food insecure.

Plots are grouped and titled by exposure and growth outcome, and each panel of grouped plots visualizes the relative risk between levels of the exposure and different age-specific growth failure outcomes. Reference levels are printed in the titles after “ref:”.

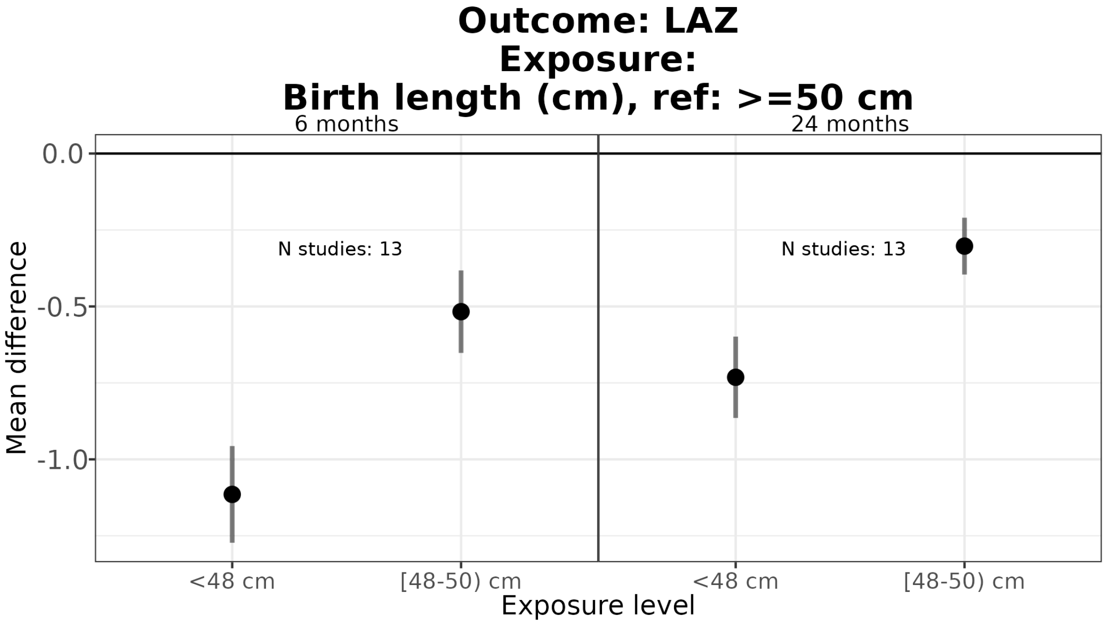


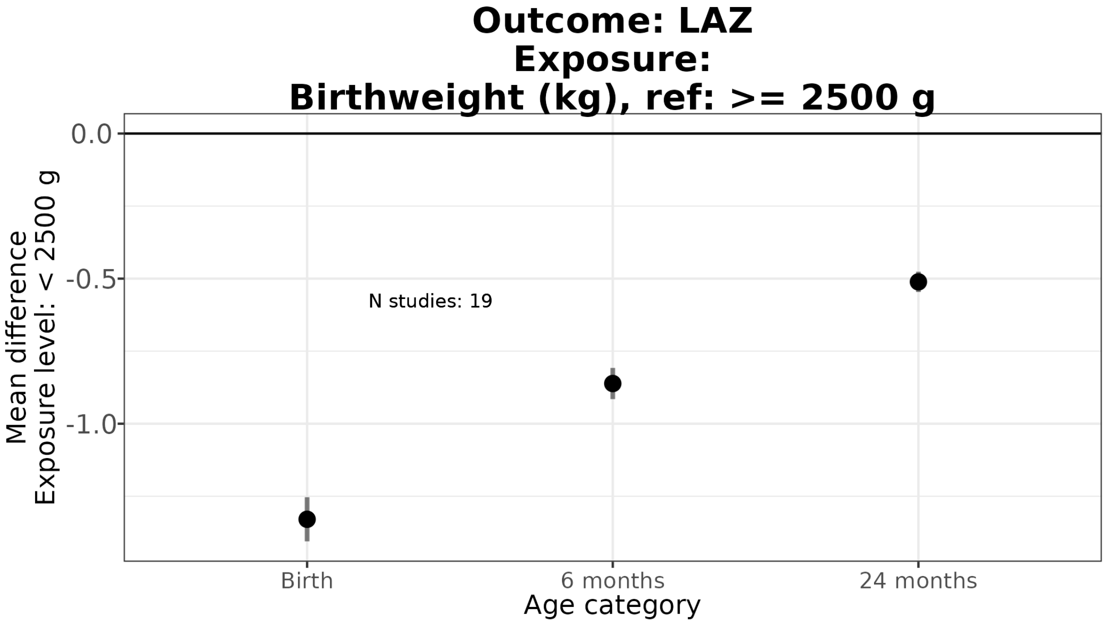

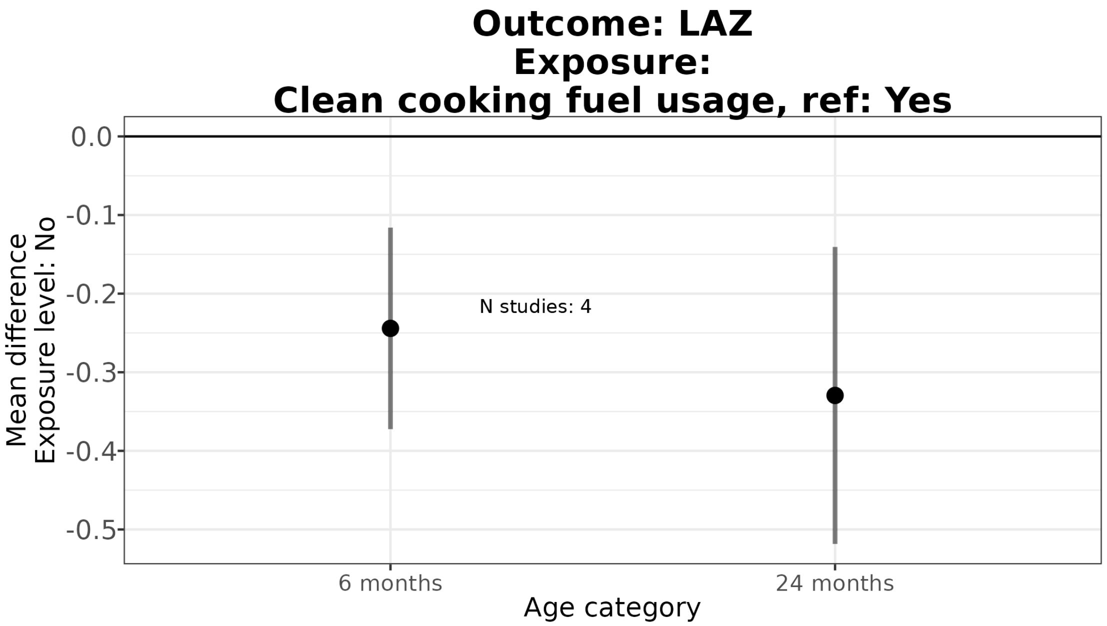

Outcome: LAZ
Exposure:
Clean cooking fuel usage, ref: Yes
Mean difference
Exposure level: No
0.0
-0.1
-0.2
-0.3
-0.4
-0.5
N studies: 4
6 months
24 months
Age category


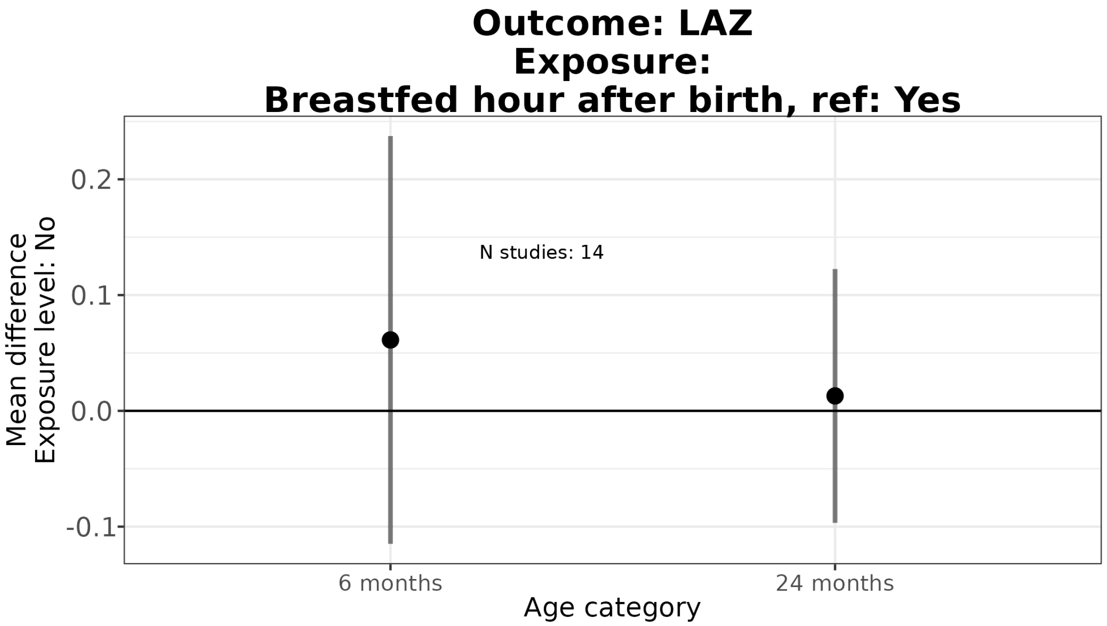

Outcome: LAZ
Exposure:
Breastfed hour after birth, ref: Yes
Mean difference
Exposure level: No
0.2
0.1
0.0
-0.1
N studies: 14
6 months
24 months
Age category

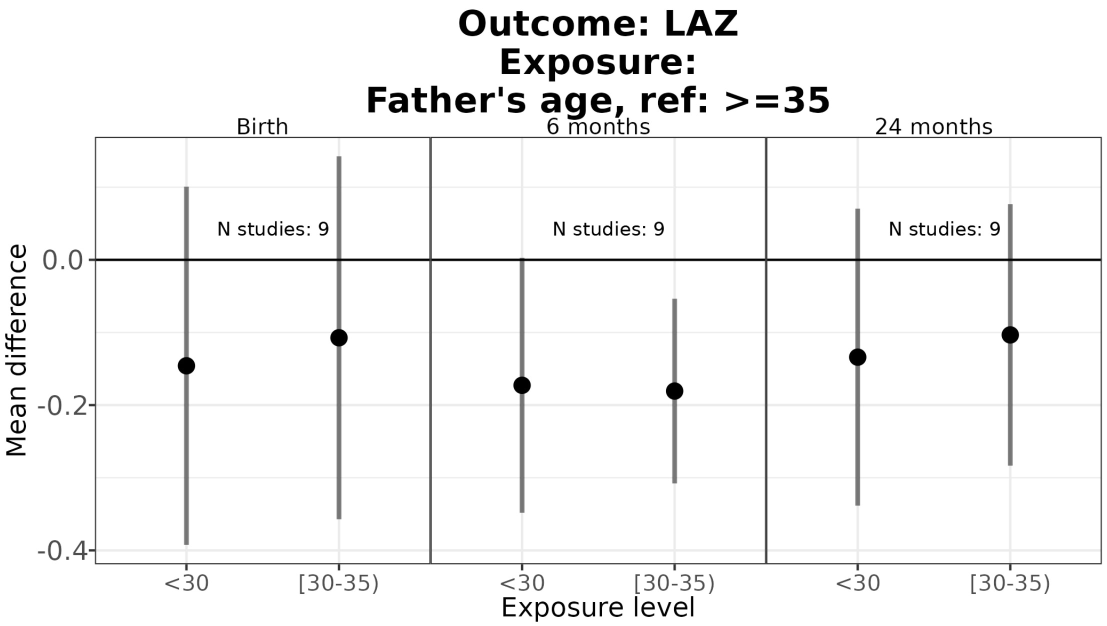
Outcome: LAZ
Exposure:
Father's age, ref: >=35
Birth
6 months
24 months
N studies: 9
N studies: 9
N studies: 9
Mean difference
0.0
-0.2
-0.4
<30
[30-35)
<30
[30-35)
<30
[30-35)
Exposure level

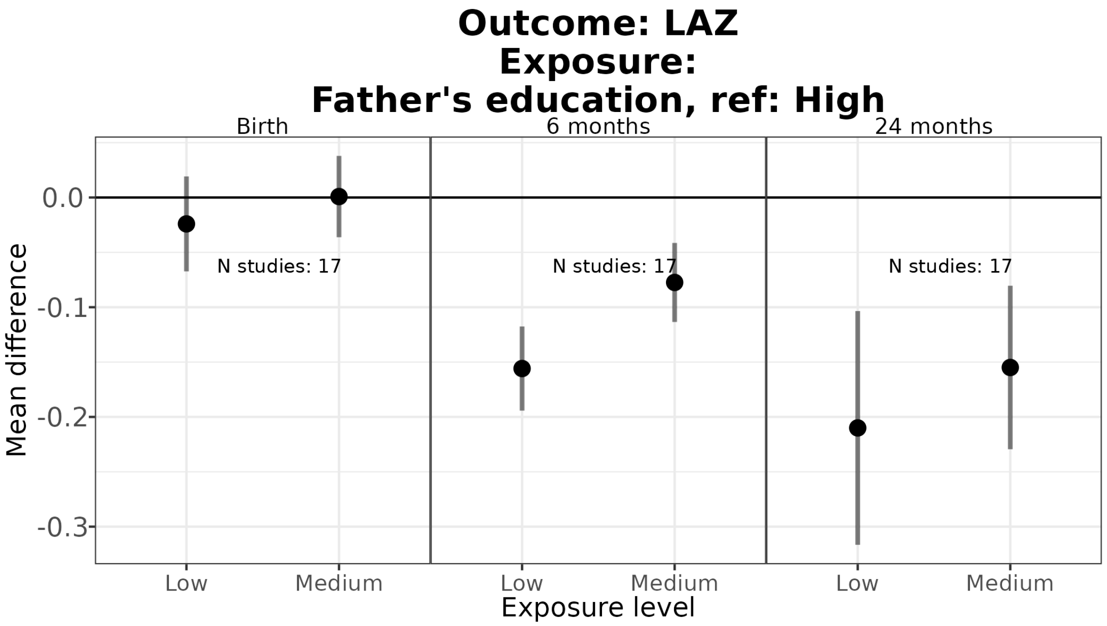
Outcome: LAZ
Exposure:
Father's education, ref: High
Birth
6 months
24 months
N studies: 17
N studies: 17
N studies: 17
Mean difference
0.0
-0.1
-0.2
-0.3
Low
Medium
Low
Medium
Low
Medium
Exposure level

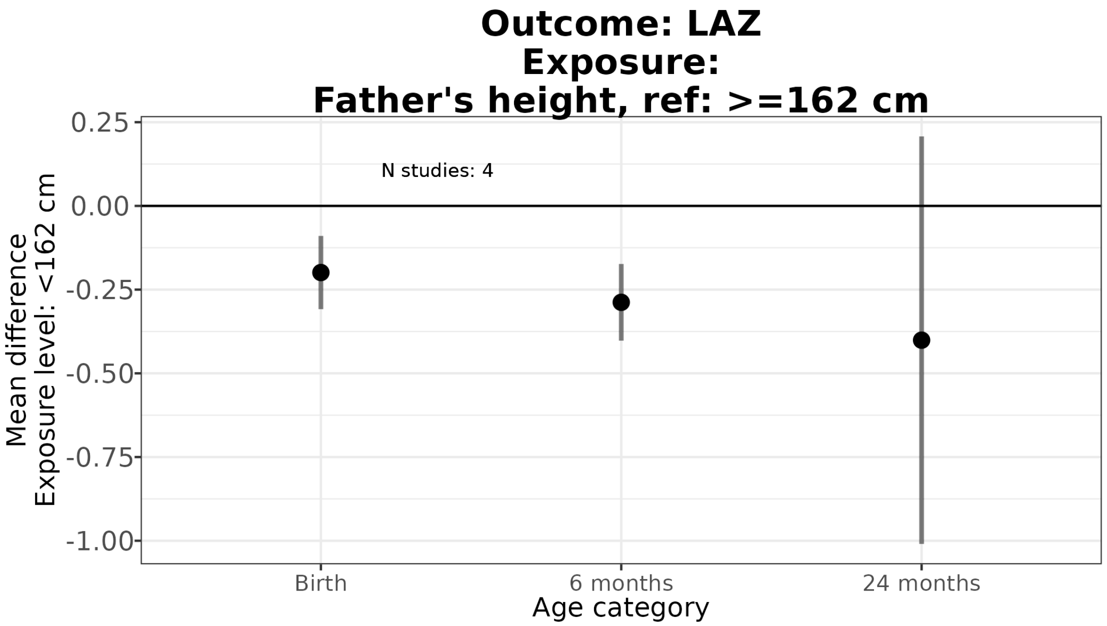

Outcome: LAZ
Exposure:
Father's height, ref: >=162 cm
N studies: 4
Mean difference
Exposure level: <162 cm
0.25
0.00
-0.25
-0.50
-0.75
-1.00
Birth
6 months
24 months
Age category


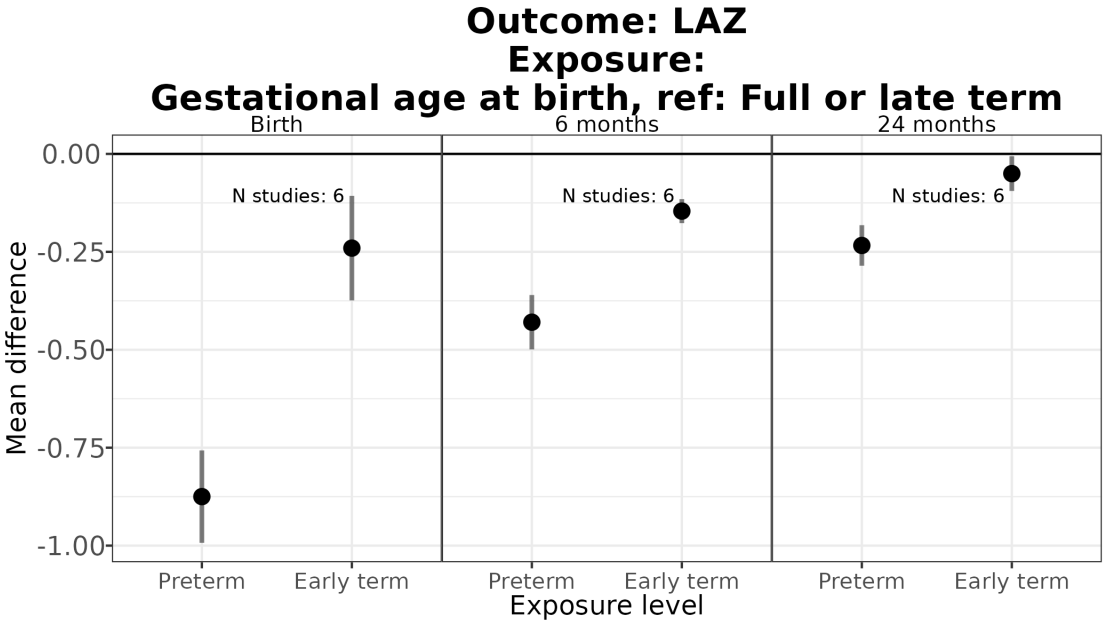

Outcome: LAZ
Exposure:
Gestational age at birth, ref: Full or late term
Birth
6 months
24 months
N studies: 6
N studies: 6
N studies: 6
Mean difference
0.00
-0.25
-0.50
-0.75
-1.00
Preterm
Early term
Preterm
Early term
Preterm
Early term
Exposure level

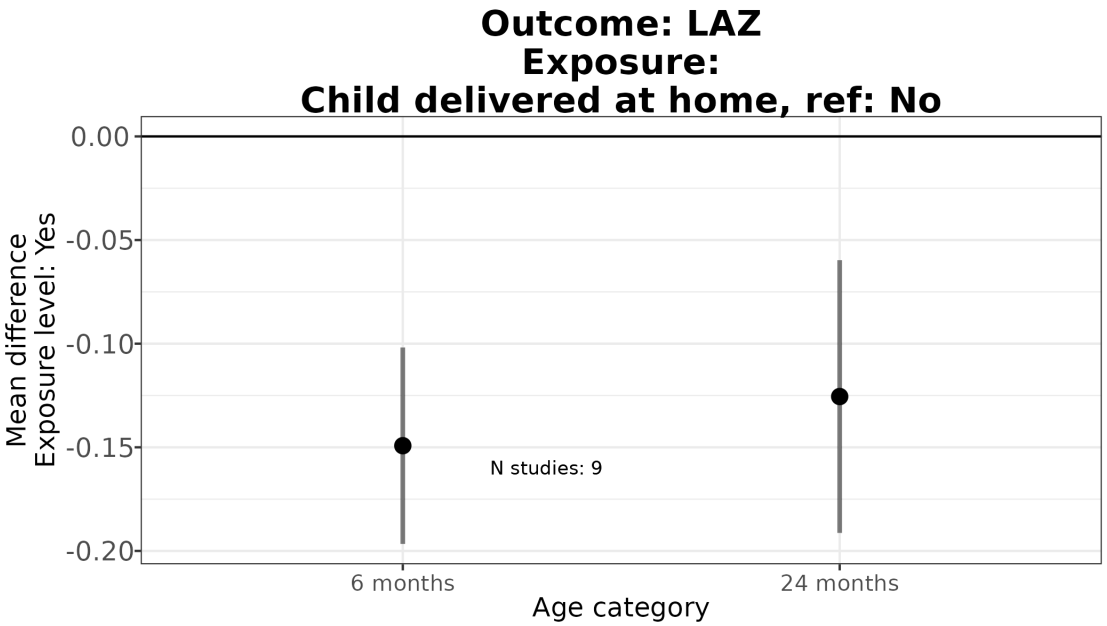

Outcome: LAZ
Exposure:
Child delivered at home, ref: No
Mean difference
Exposure level: Yes
0.00
-0.05
-0.10
-0.15
-0.20
N studies: 9
6 months
24 months
Age category


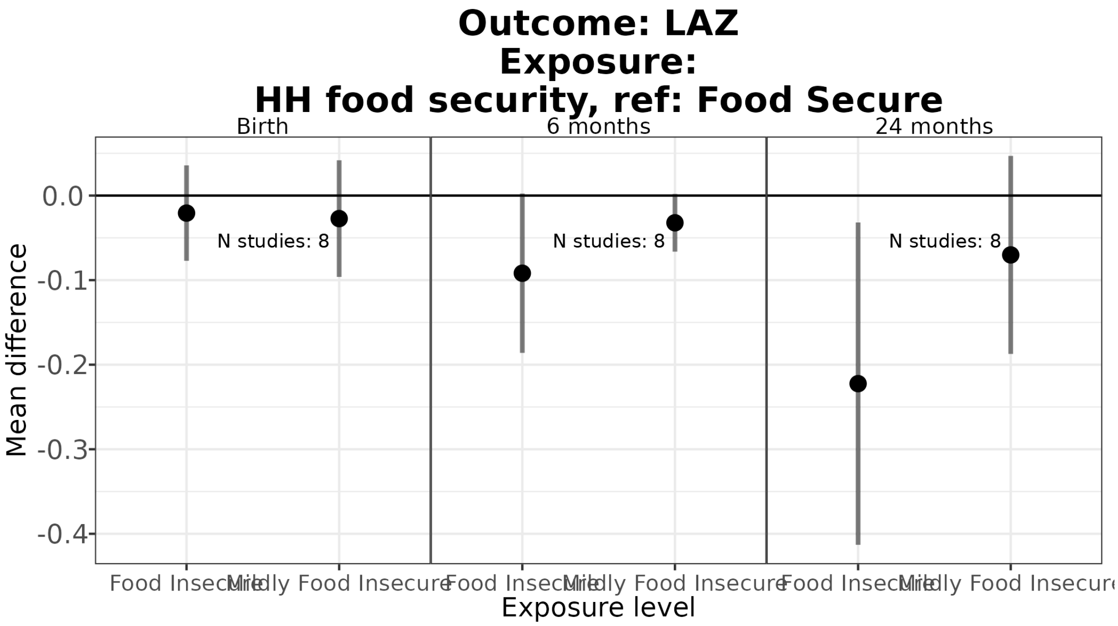

Outcome: LAZ
Exposure:
HH food security, ref: Food Secure
Birth
6 months
24 months
Mean difference
0.0
-0.1
-0.2
-0.3
-0.4
N studies: 8
N studies: 8
N studies: 8
Food Insecure
Mildly Food Insecure
Food Insecure
Mildly Food Insecure
Food Insecure
Mildly Food Insecure
Exposure level

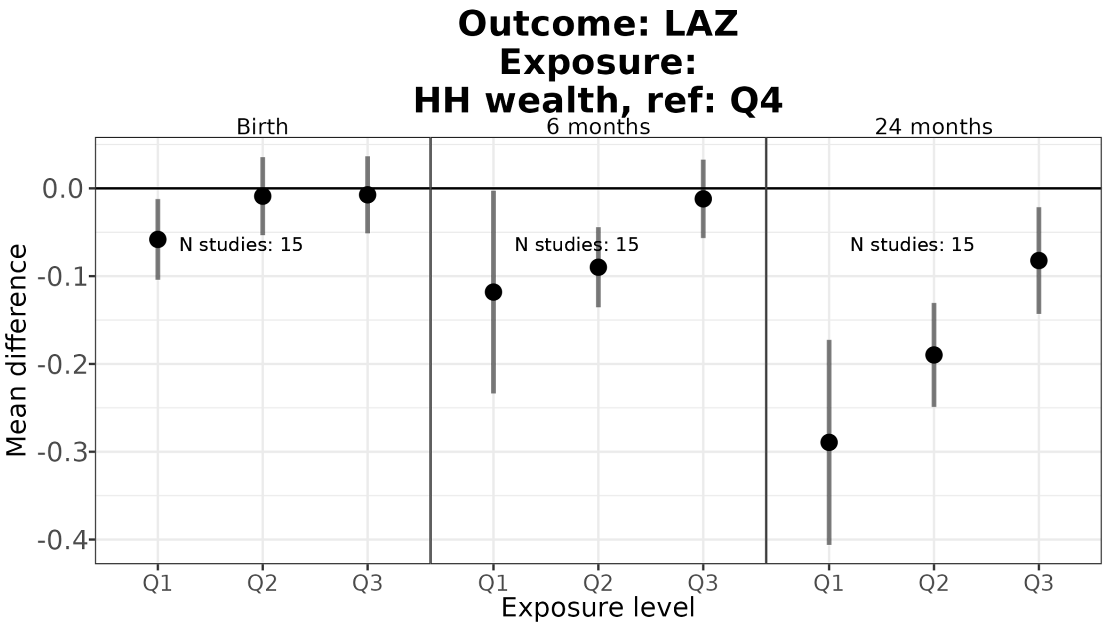
Outcome: LAZ
Exposure:
HH wealth, ref: Q4
Birth
6 months
24 months
N studies: 15
N studies: 15
N studies: 15
Mean difference
0.0
-0.1
-0.2
-0.3
-0.4
Q1
Q2
Q3
Q1
Q2
Q3
Q1
Q2
Q3
Exposure level

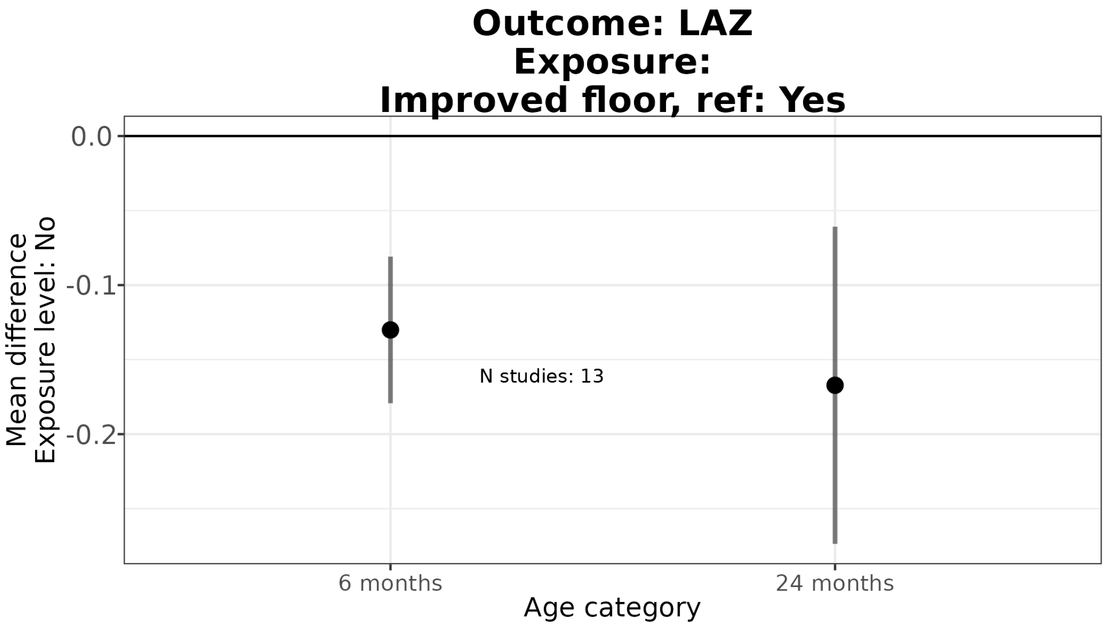
Outcome: LAZ
Exposure:
Improved floor, ref: Yes
Mean difference
Exposure level: No
0.0
-0.1
-0.2
N studies: 13
6 months
24 months
Age category

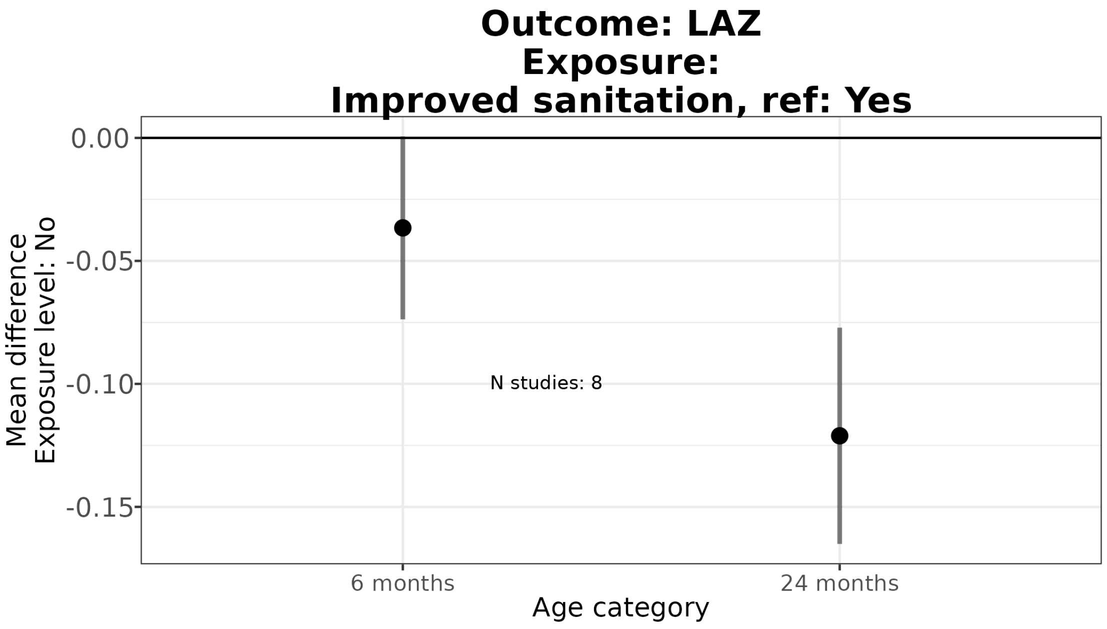
Outcome: LAZ
Exposure:
Improved sanitation, ref: Yes
Mean difference
Exposure level: No
0.00
-0.05
-0.10
-0.15
N studies: 8
6 months
24 months
Age category

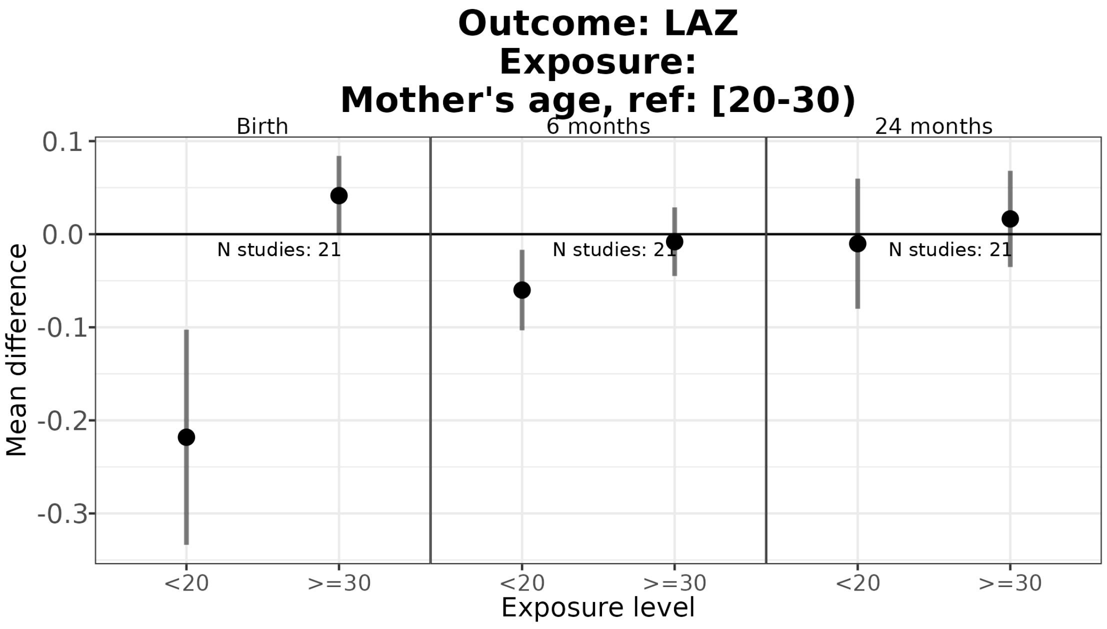
Outcome: LAZ
Exposure:
Mother's age, ref: [20-30)
Birth
6 months
24 months
Mean difference
0.1
0.0
-0.1
-0.2
-0.3
N studies: 21
N studies: 21
N studies: 21
<20
>=30
<20
>=30
<20
>=30
Exposure level

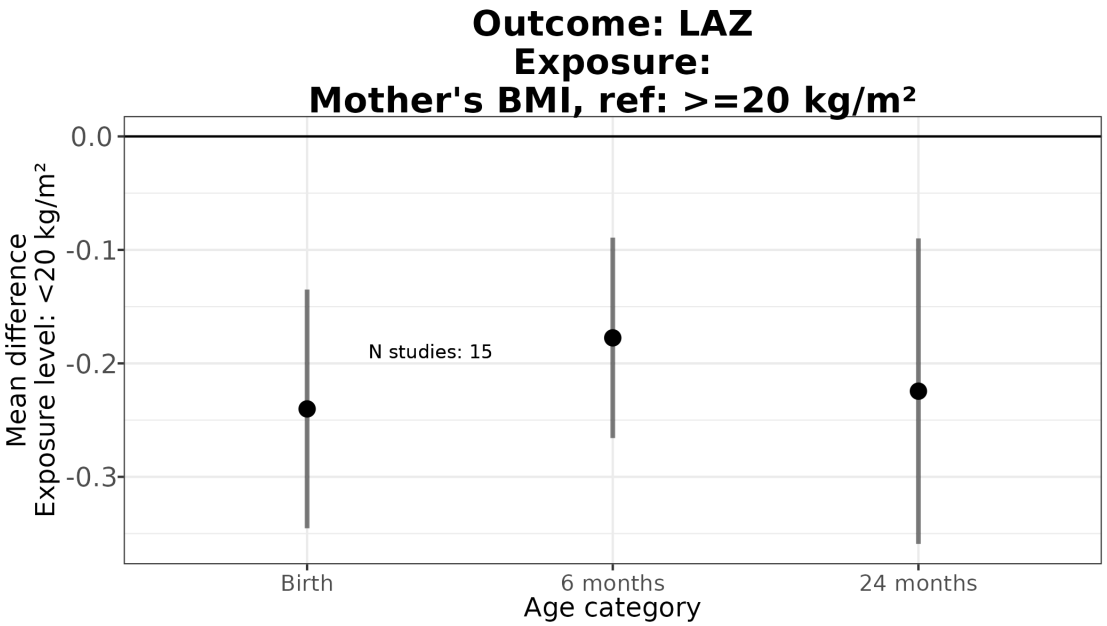
Outcome: LAZ
Exposure:
Mother's BMI, ref: >=20 kg/m²
Mean difference
Exposure level: <20 kg/m²
0.0
-0.1
-0.2
-0.3
N studies: 15
Birth
6 months
24 months
Age category

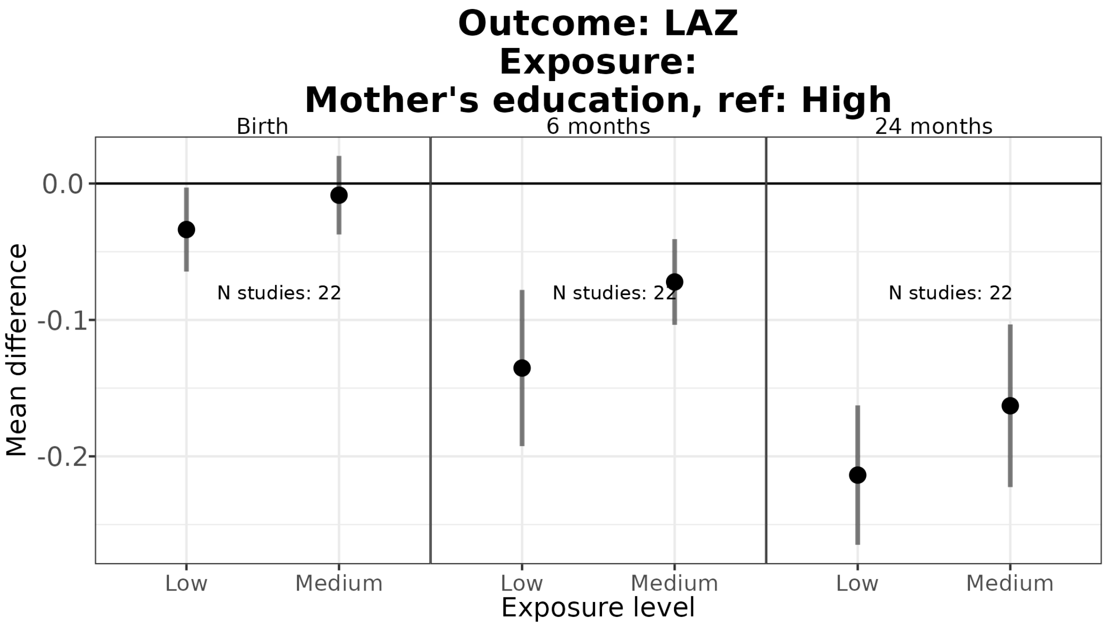
Outcome: LAZ
Exposure:
Mother's education, ref: High
Birth
6 months
24 months
Mean difference
0.0
-0.1
-0.2
N studies: 22
N studies: 22
N studies: 22
Low
Medium
Low
Medium
Low
Medium
Exposure level

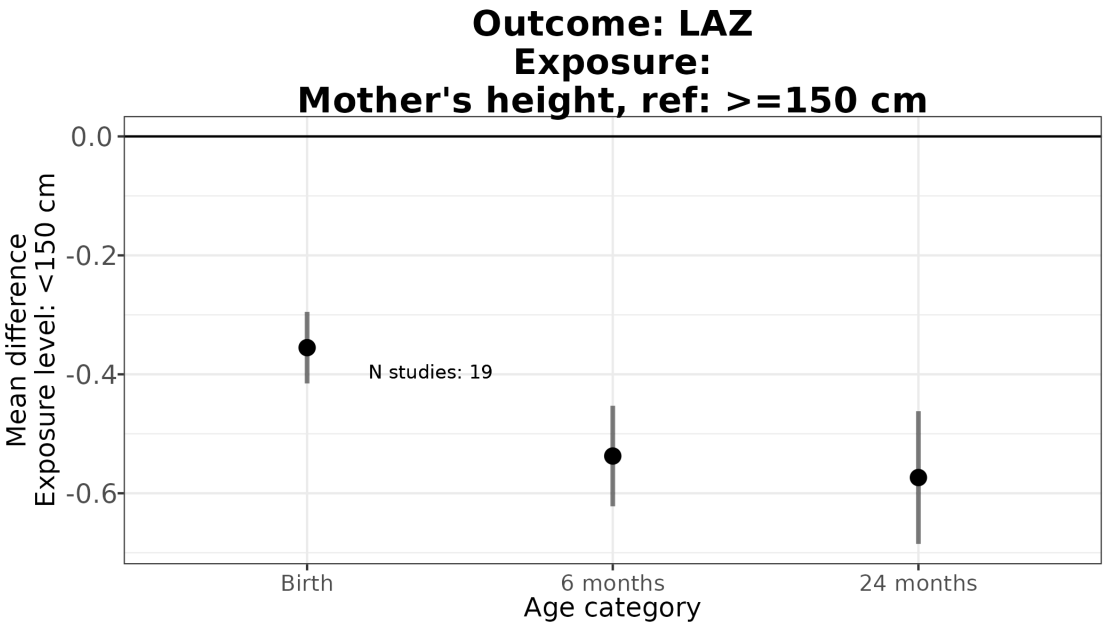
Outcome: LAZ
Exposure:
Mother's height, ref: >=150 cm
Mean difference
Exposure level: <150 cm
0.0
-0.2
-0.4
-0.6
N studies: 19
Birth
6 months
24 months
Age category

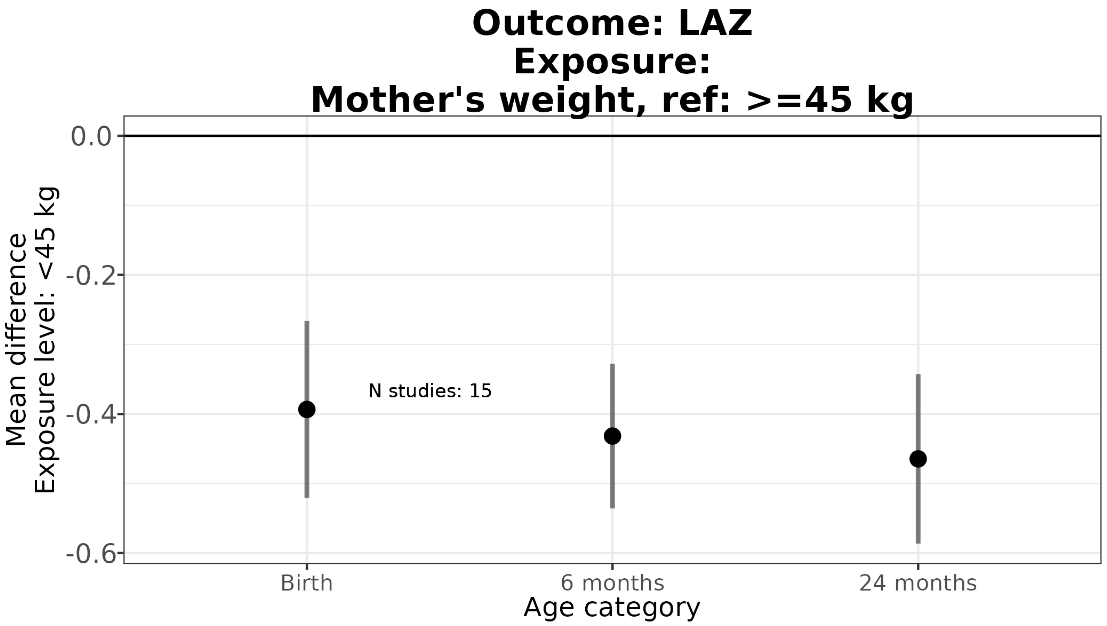
Outcome: LAZ
Exposure:
Mother's weight, ref: >=45 kg
Mean difference
Exposure level: <45 kg
0.0
-0.2
-0.4
-0.6
N studies: 15
Birth
6 months
24 months
Age category

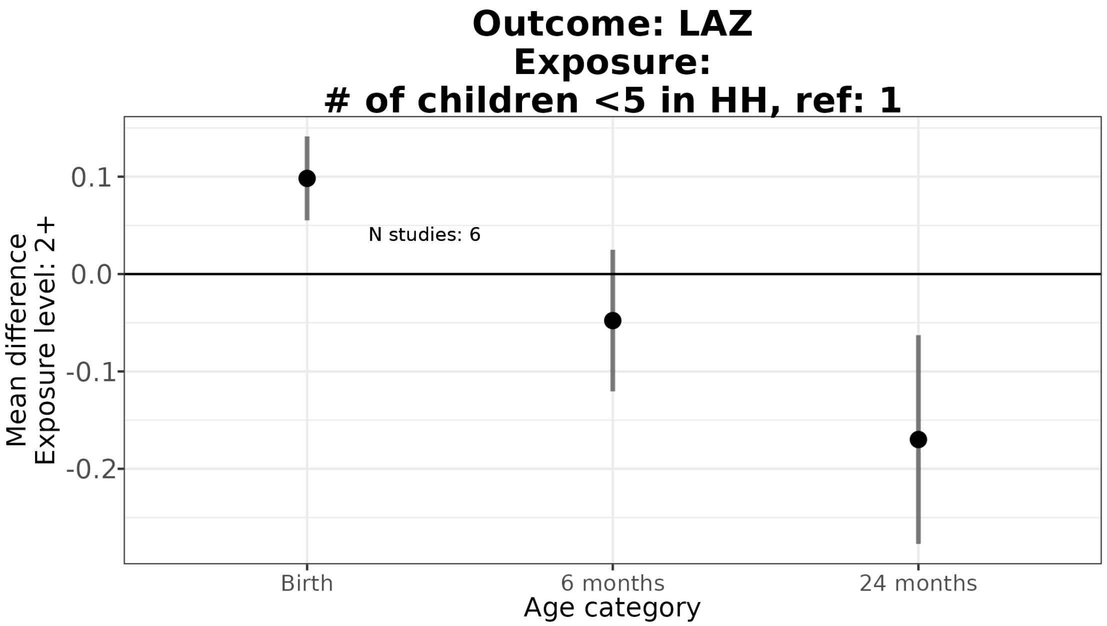

Outcome: LAZ
Exposure:
# of children <5 in HH, ref: 1
Mean difference
Exposure level: 2+
0.1
0.0
-0.1
-0.2
N studies: 6
Birth
6 months
24 months
Age category


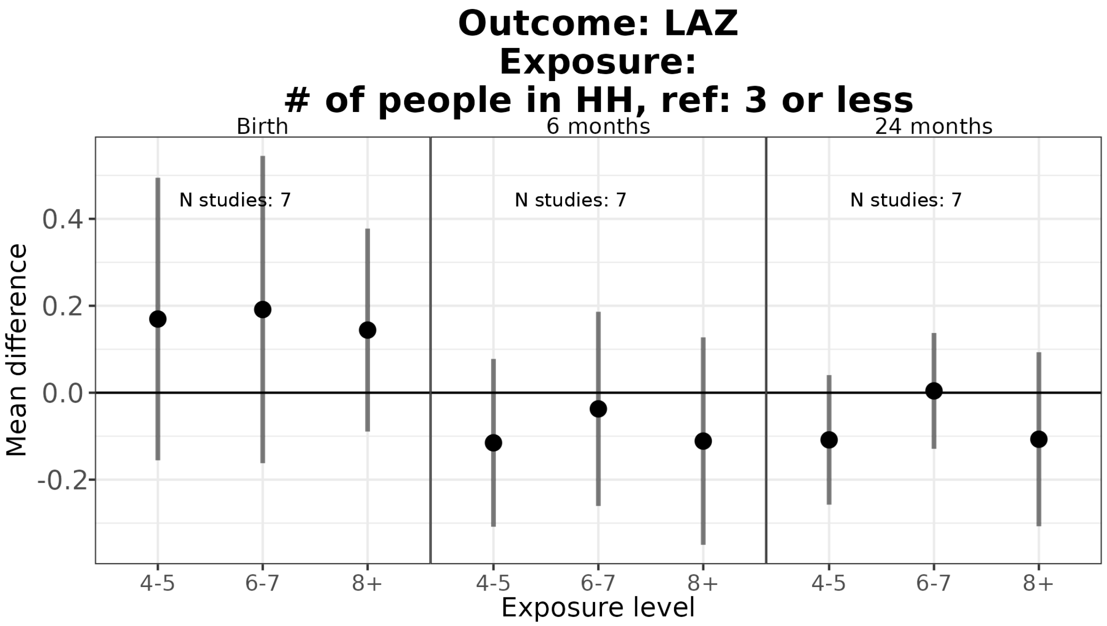

Outcome: LAZ
Exposure:
# of people in HH, ref: 3 or less
Birth
6 months
24 months
N studies: 7
N studies: 7
N studies: 7
Mean difference
0.4
0.2
0.0
-0.2
4-5
6-7
8+
4-5
6-7
8+
4-5
6-7
8+
Exposure level

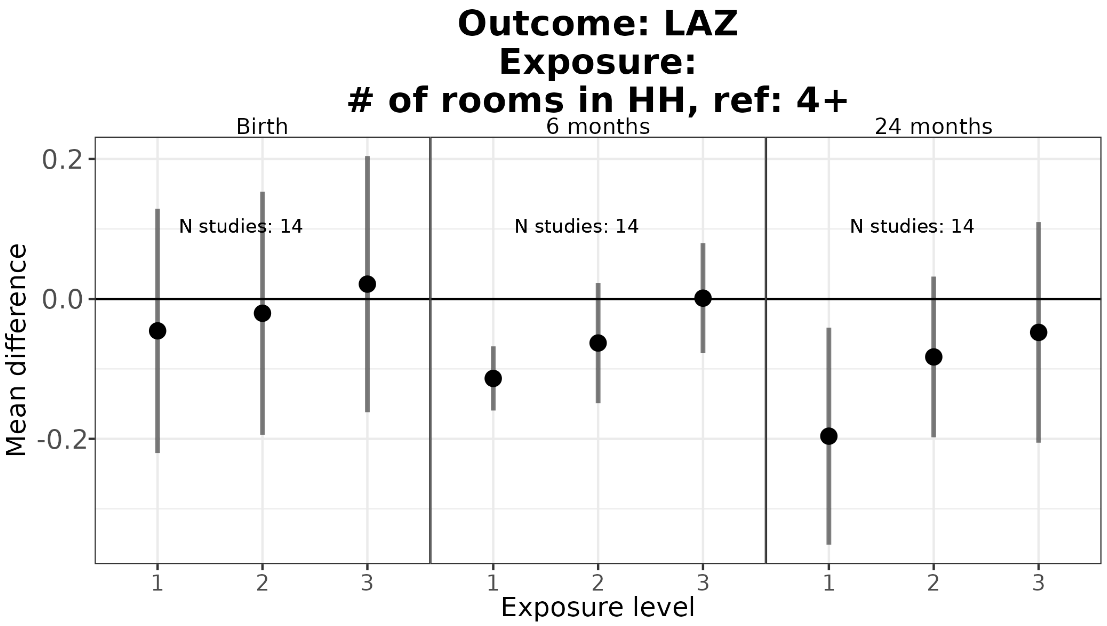
Outcome: LAZ
Exposure:
# of rooms in HH, ref: 4+
Birth
6 months
24 months
N studies: 14
N studies: 14
N studies: 14
0.2
0.0
-0.2
Mean difference
1
2
3
1
2
3
1
2
3
Exposure level

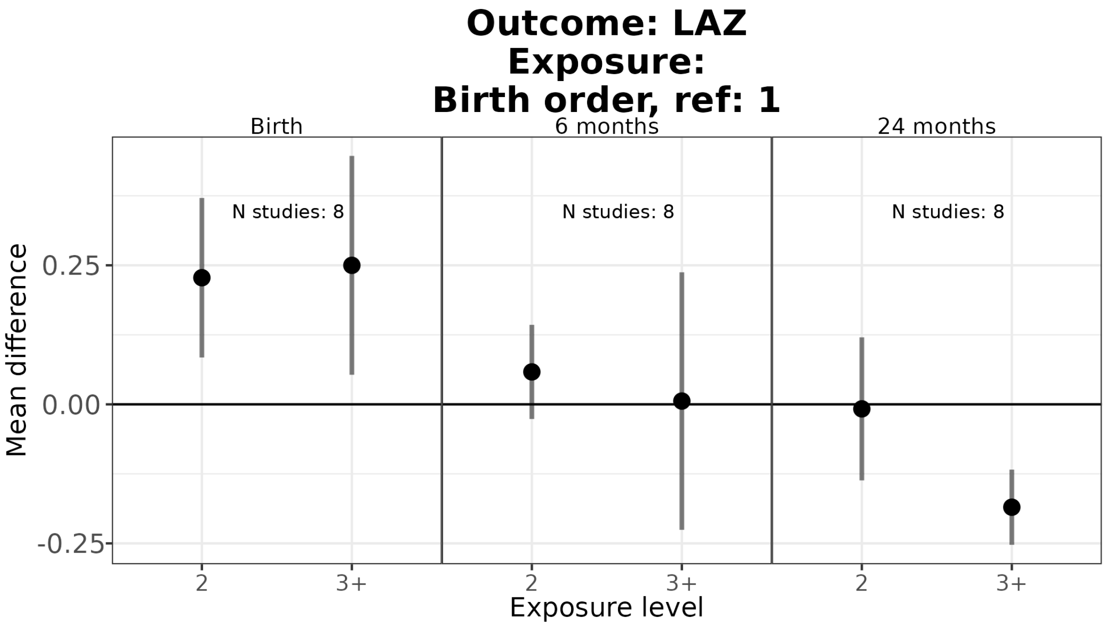
Outcome: LAZ
Exposure:
Birth order, ref: 1
Birth
6 months
24 months
N studies: 8
N studies: 8
N studies: 8
0.25
0.00
-0.25
Mean difference
2
3+
2
3+
2
3+
Exposure level

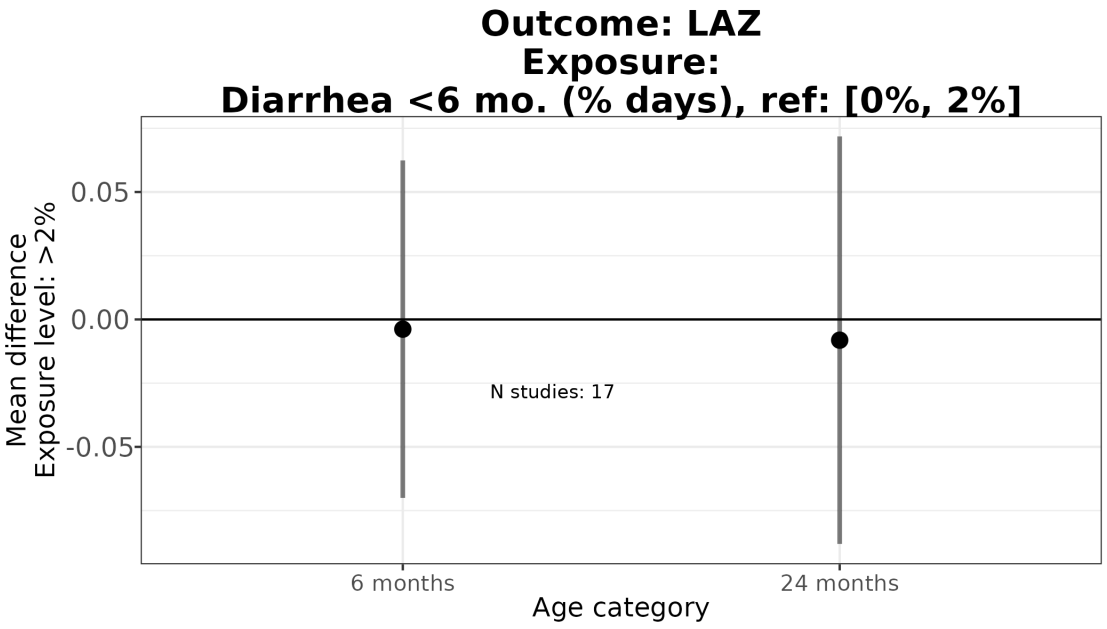

Outcome: LAZ
Exposure:
Diarrhea <6 mo. (% days), ref: [0%, 2%]
Mean difference
Exposure level: >2%
0.05
0.00
-0.05
N studies: 17
6 months
24 months
Age category

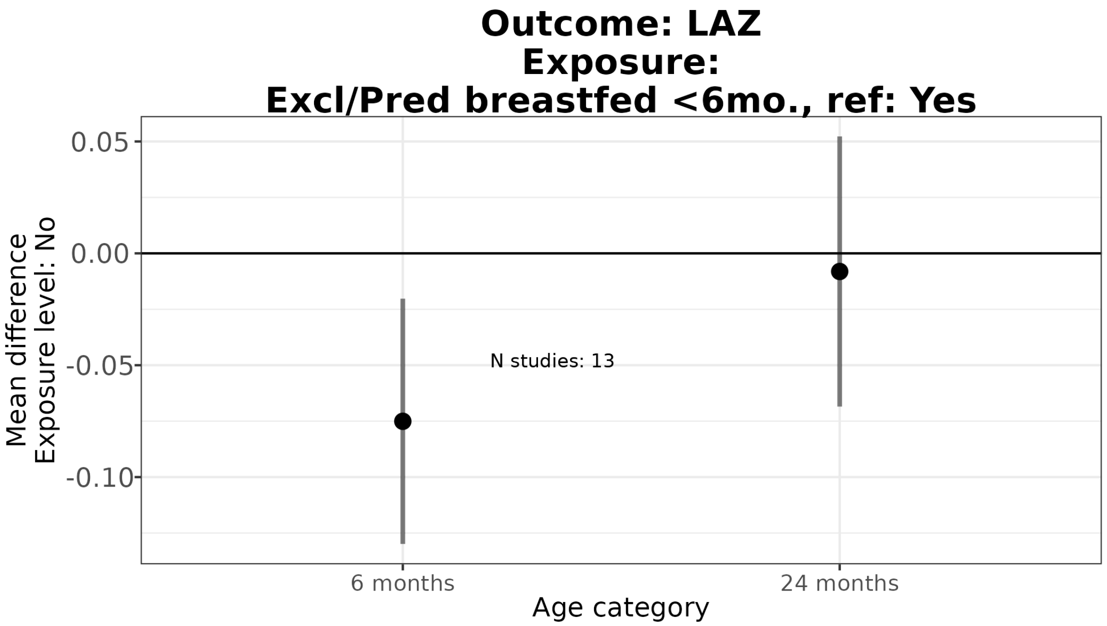
Outcome: LAZ
Exposure:
Excl/Pred breastfed <6mo., ref: Yes
Mean difference
Exposure level: No
0.05
0.00
-0.05
-0.10
N studies: 13
6 months
24 months
Age category

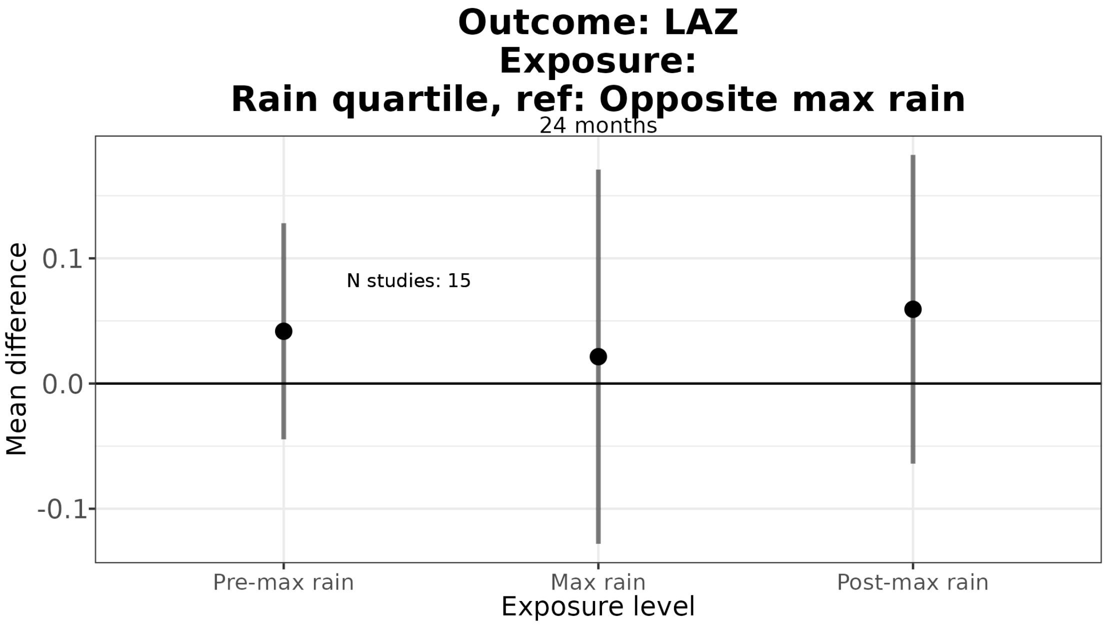
Outcome: LAZ
Exposure:
Rain quartile, ref: Opposite max rain
24 months
Mean difference
0.1
0.0
-0.1
N studies: 15
Pre-max rain
Max rain
Post-max rain
Exposure level

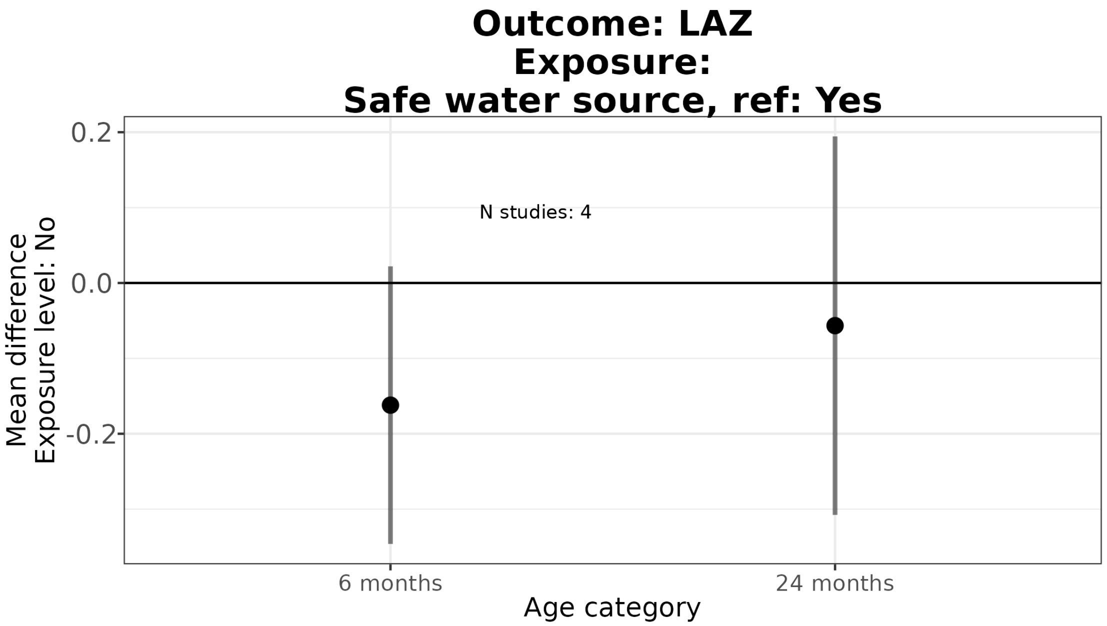
Outcome: LAZ
Exposure:
Safe water source, ref: Yes
Mean difference
Exposure level: No
0.2
0.0
-0.2
N studies: 4
6 months
24 months
Age category

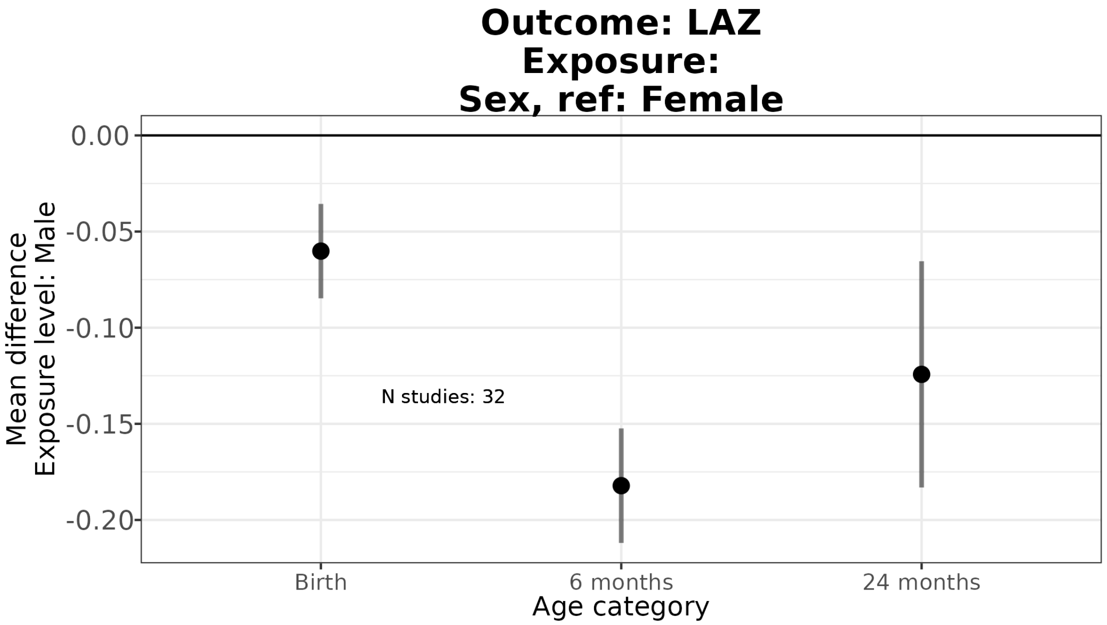
Outcome: LAZ
Exposure:
Sex, ref: Female
Mean difference
Exposure level: Male
0.00
-0.05
-0.10
-0.15
-0.20
N studies: 32
Birth
6 months
24 months
Age category

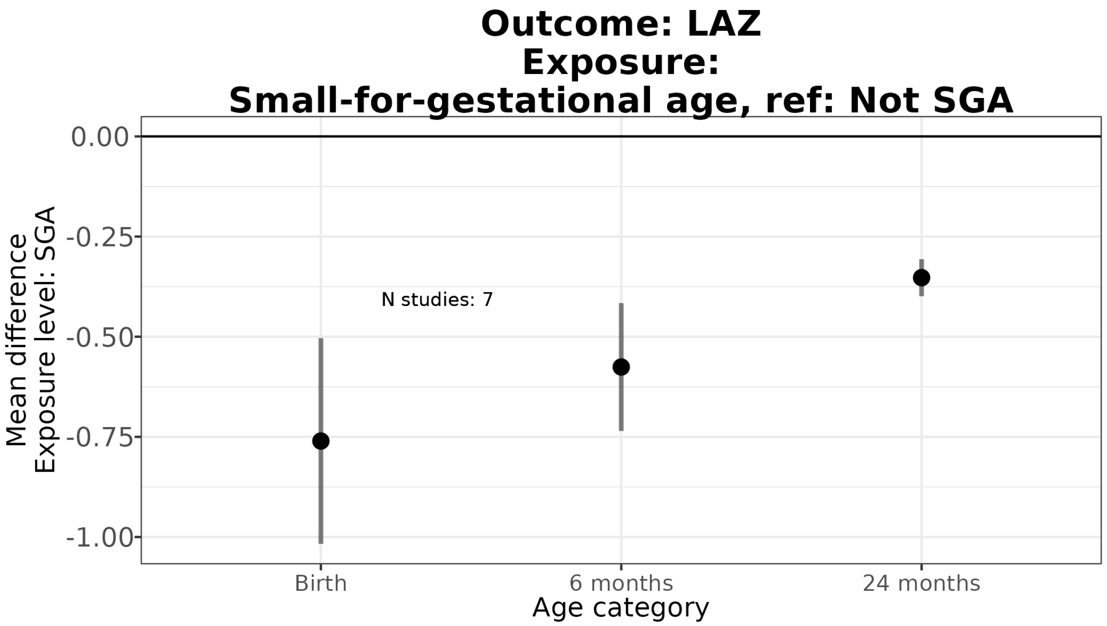
Outcome: LAZ
Exposure:
Small-for-gestational age, ref: Not SGA
Mean difference
Exposure level: SGA
0.00
-0.25
-0.50
-0.75
-1.00
N studies: 7
Birth
6 months
24 months
Age category

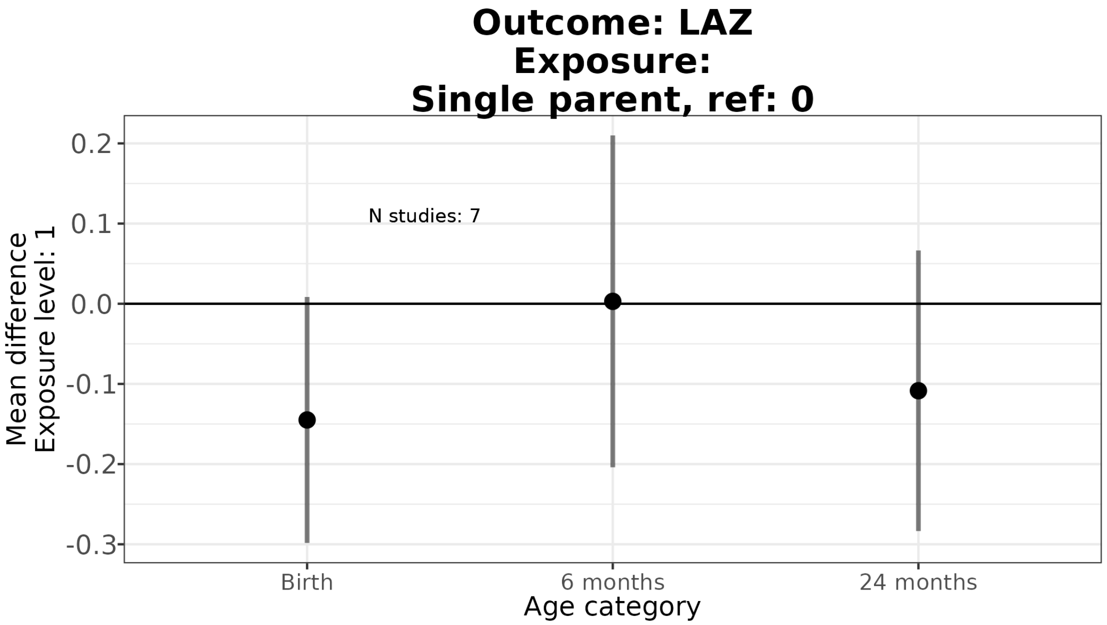
Outcome: LAZ
Exposure:
Single parent, ref: 0
Mean difference
Exposure level: 1
0.2
0.1
0.0
-0.1
-0.2
-0.3
N studies: 7
Birth
6 months
24 months
Age category

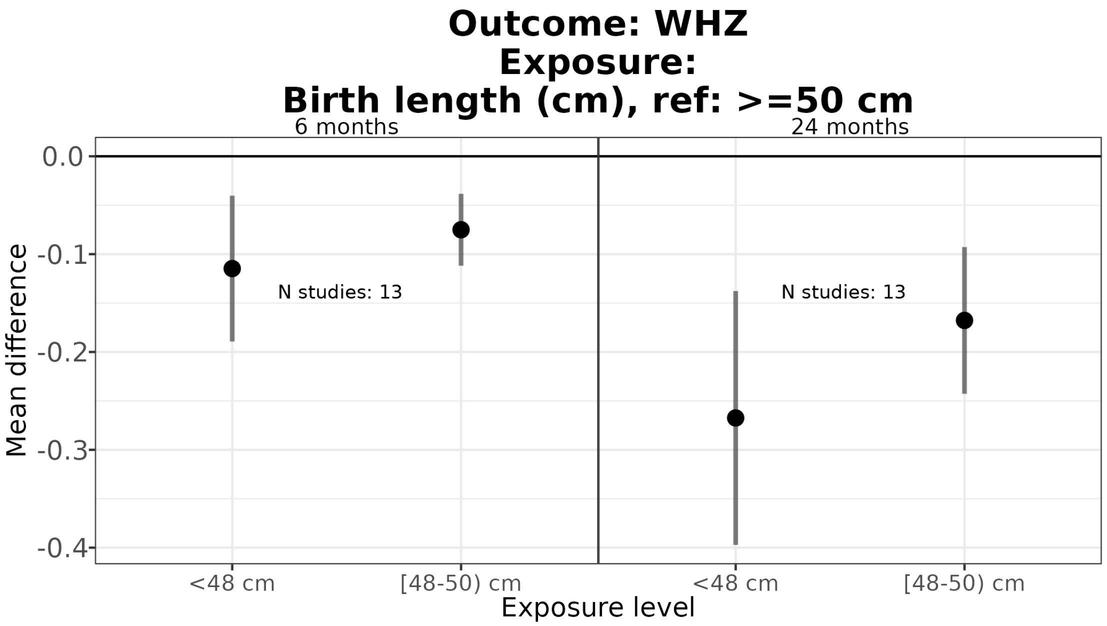

Outcome: WHZ
Exposure:
Birth length (cm), ref: >=50 cm
6 months
24 months
0.0
-0.1
-0.2
-0.3
-0.4
Mean difference
N studies: 13
N studies: 13
<48 cm
[48-50) cm
<48 cm
[48-50) cm
Exposure level


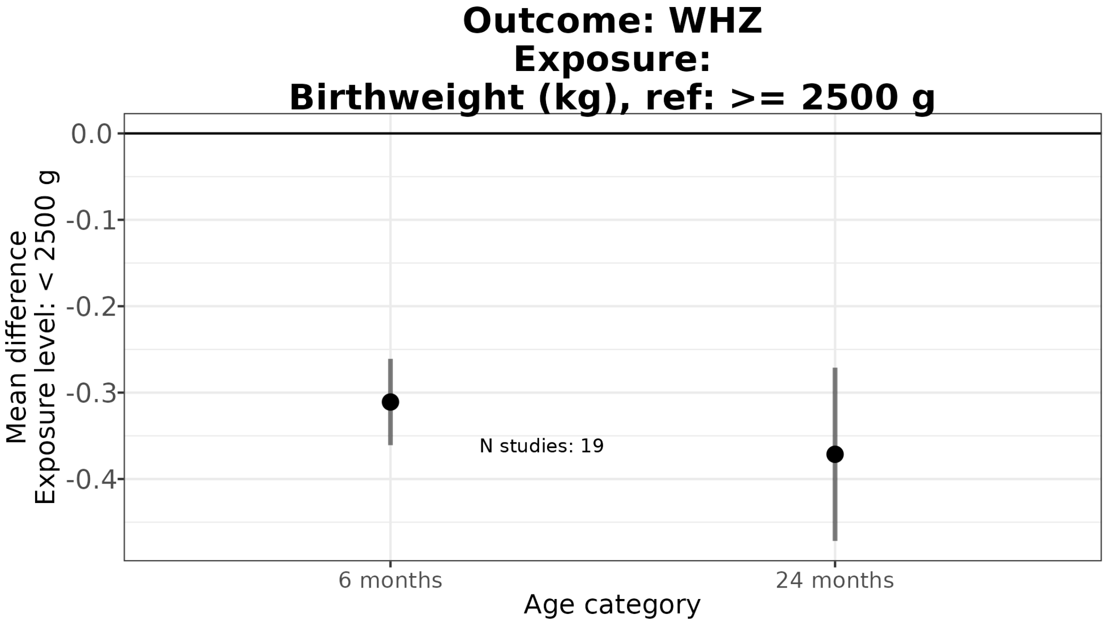

Outcome: WHZ
Exposure:
Birthweight (kg), ref: >= 2500 g
0.0
-0.1
-0.2
-0.3
-0.4
Mean difference
Exposure level: < 2500 g
N studies: 19
6 months
24 months
Age category

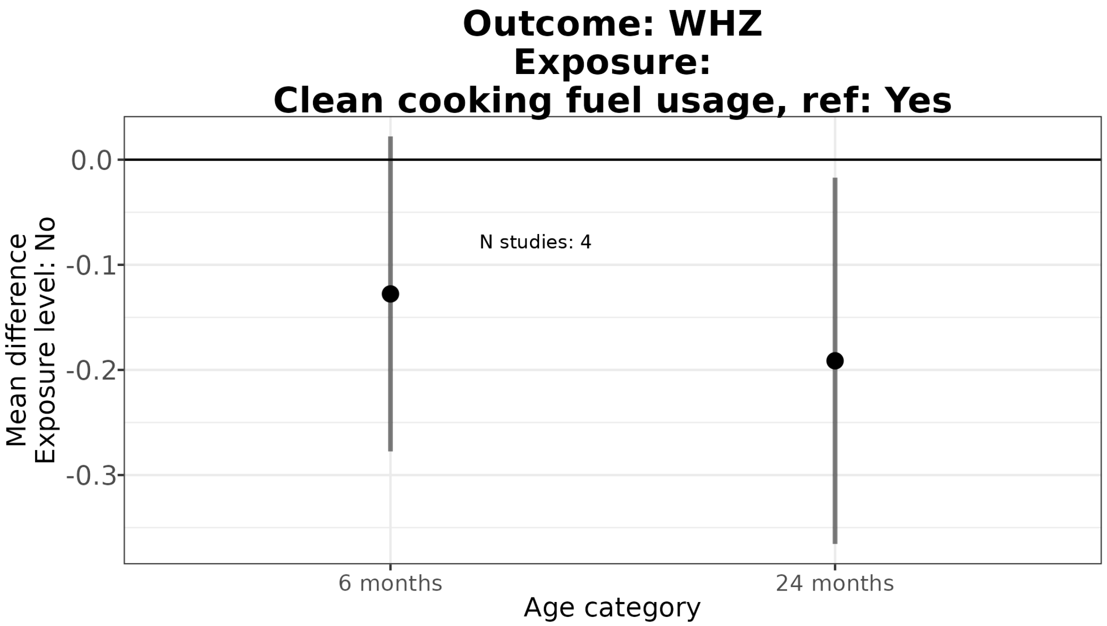
Outcome: WHZ
Exposure:
Clean cooking fuel usage, ref: Yes
Mean difference
Exposure level: No
0.0
-0.1
-0.2
-0.3
N studies: 4
6 months
24 months
Age category

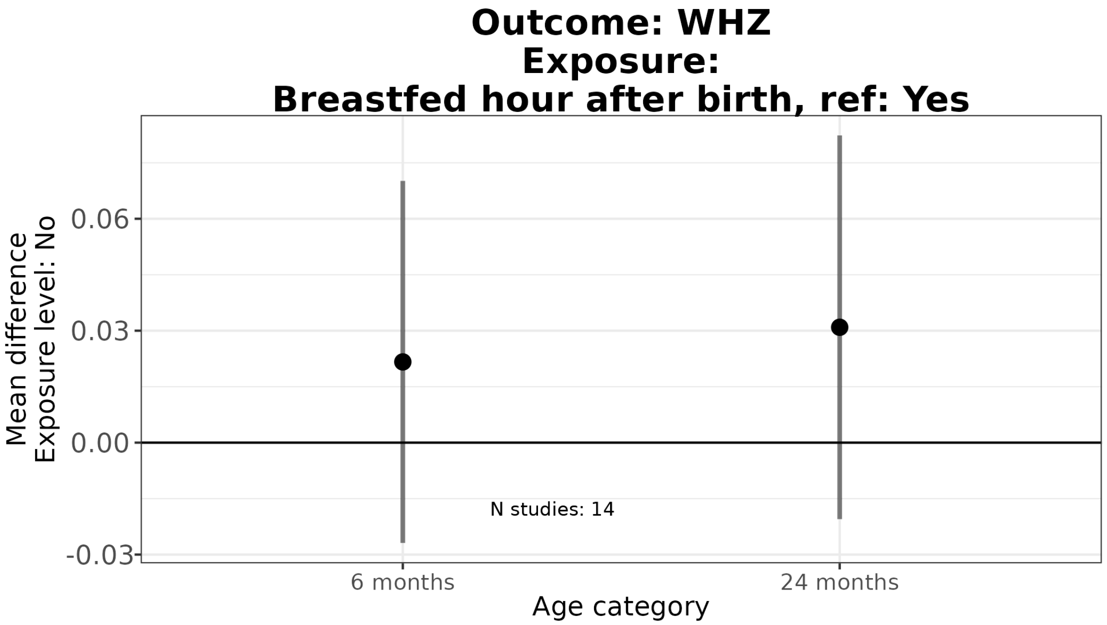
Outcome: WHZ
Exposure:
Breastfed hour after birth, ref: Yes
Mean difference
Exposure level: No
0.06
0.03
0.00
-0.03
N studies: 14
6 months
24 months
Age category

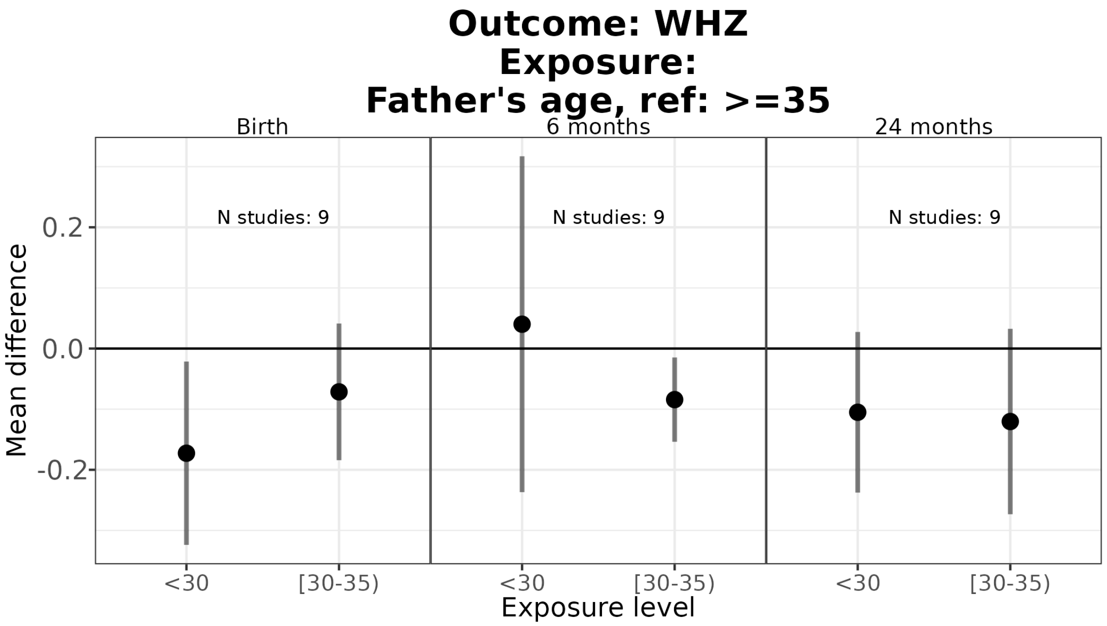
Outcome: WHZ
Exposure:
Father's age, ref: >=35
Birth
6 months
24 months
N studies: 9
N studies: 9
N studies: 9
0.2
0.0
-0.2
Mean difference
<30
[30-35)
<30
[30-35)
<30
[30-35)
Exposure level

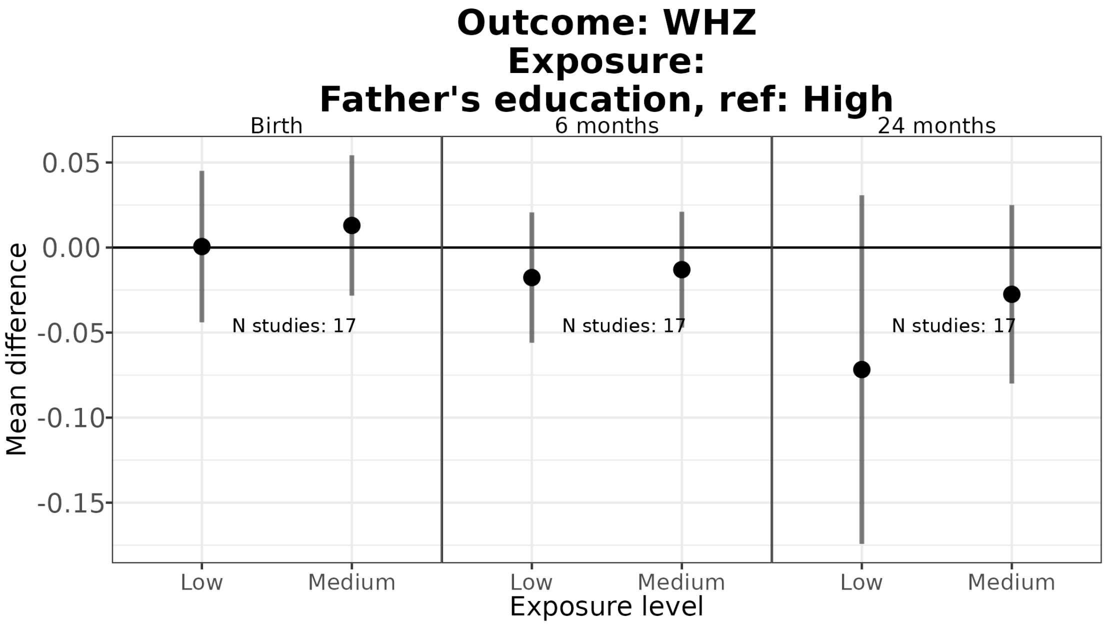
Outcome: WHZ
Exposure:
Father's education, ref: High
Birth
6 months
24 months
0.05
0.00
-0.05
-0.10
-0.15
Mean difference
N studies: 17
N studies: 17
N studies: 17
Low
Medium
Low
Medium
Low
Medium
Exposure level

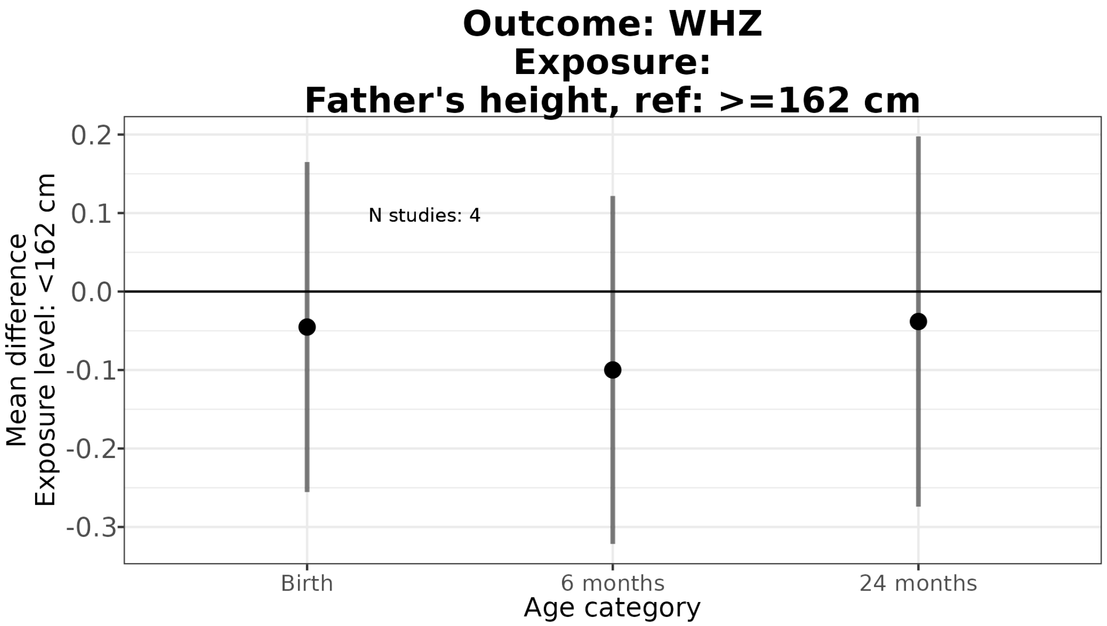
Outcome: WHZ
Exposure:
Father's height, ref: >=162 cm
Mean difference
Exposure level: <162 cm
N studies: 4
0.2
0.1
0.0
-0.1
-0.2
-0.3
Birth
6 months
24 months
Age category

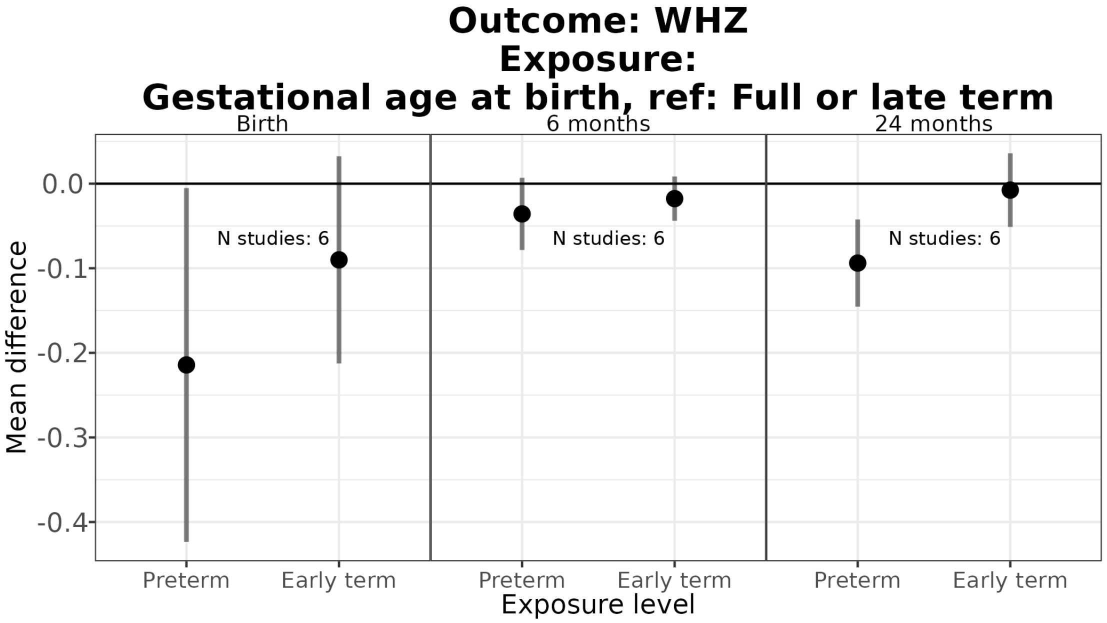
Outcome: WHZ
Exposure:
Gestational age at birth, ref: Full or late term
Birth
6 months
24 months
Mean difference
N studies: 6
N studies: 6
N studies: 6
0.0
-0.1
-0.2
-0.3
-0.4
Preterm
Early term
Preterm
Early term
Preterm
Early term
Exposure level

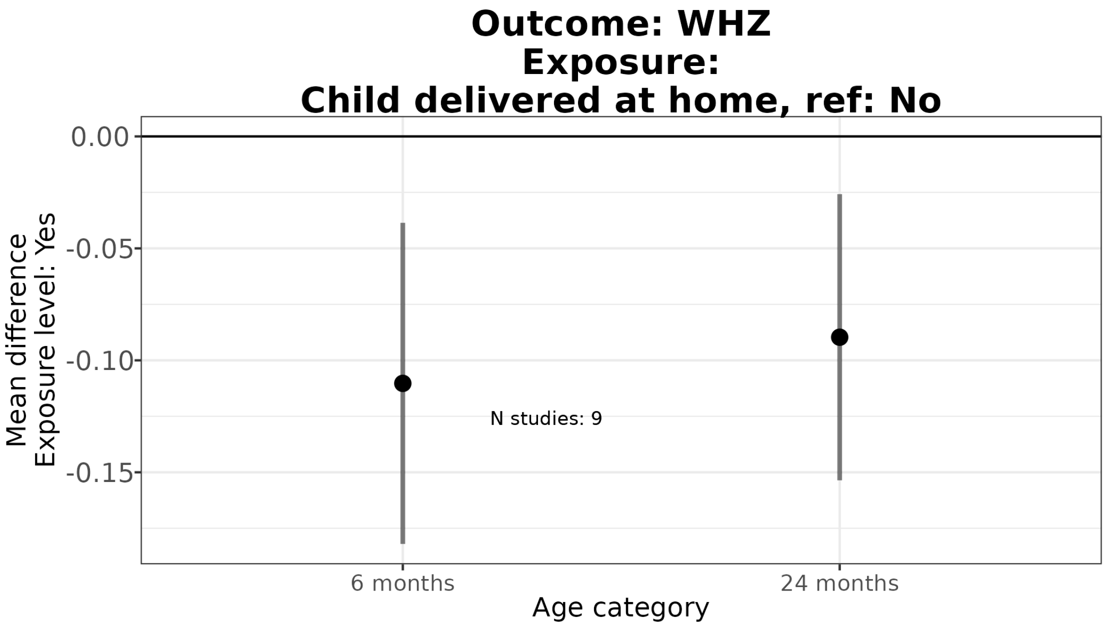
Outcome: WHZ
Exposure:
Child delivered at home, ref: No
Mean difference
Exposure level: Yes
0.00
-0.05
-0.10
-0.15
N studies: 9
6 months
24 months
Age category

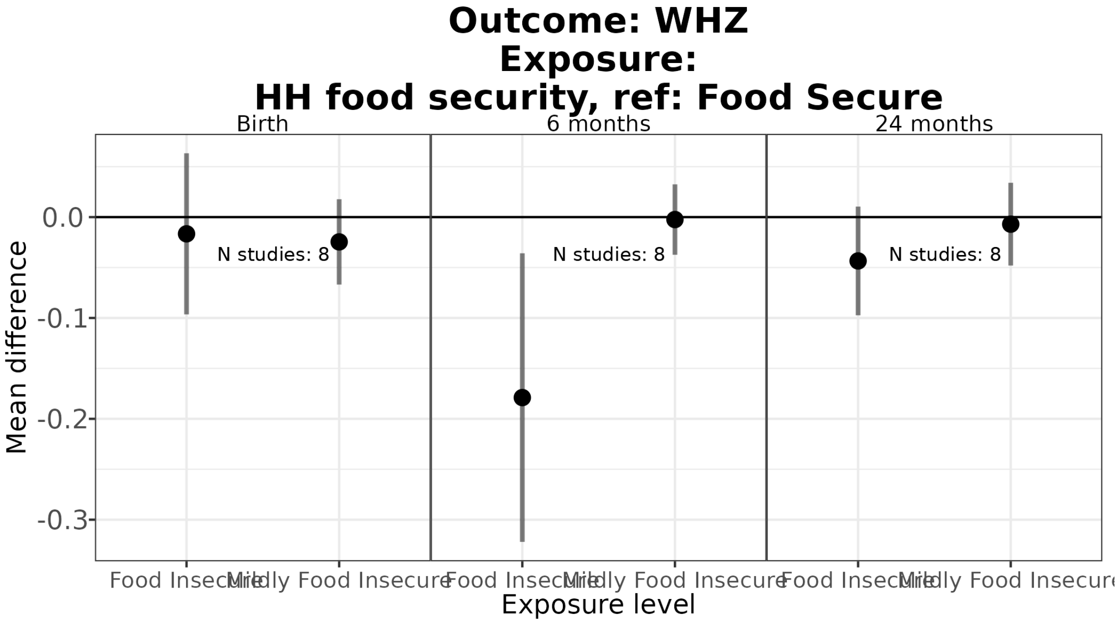
Outcome: WHZ
Exposure:
HH food security, ref: Food Secure
Birth
6 months
24 months
Mean difference
0.0
-0.1
-0.2
-0.3
N studies: 8
N studies: 8
N studies: 8
Food Insecure
Mildly Food Insecure
Exposure level

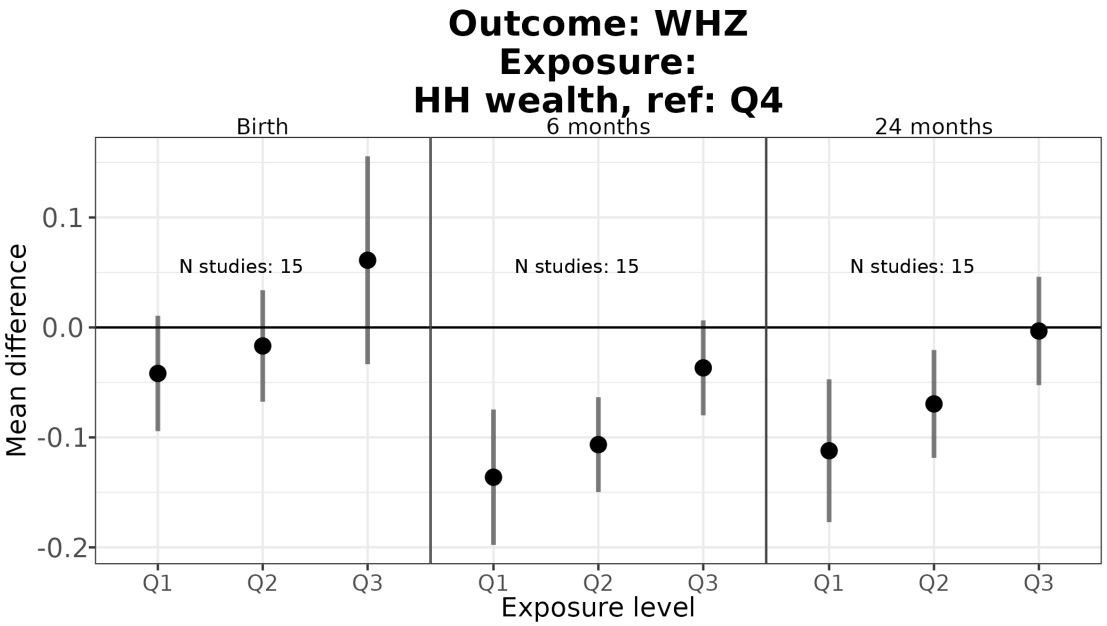
Outcome: WHZ
Exposure:
HH wealth, ref: Q4
Birth
6 months
24 months
N studies: 15
N studies: 15
N studies: 15
Mean difference
0.1
0.0
-0.1
-0.2
Q1
Q2
Q3
Q1
Q2
Q3
Q1
Q2
Q3
Exposure level

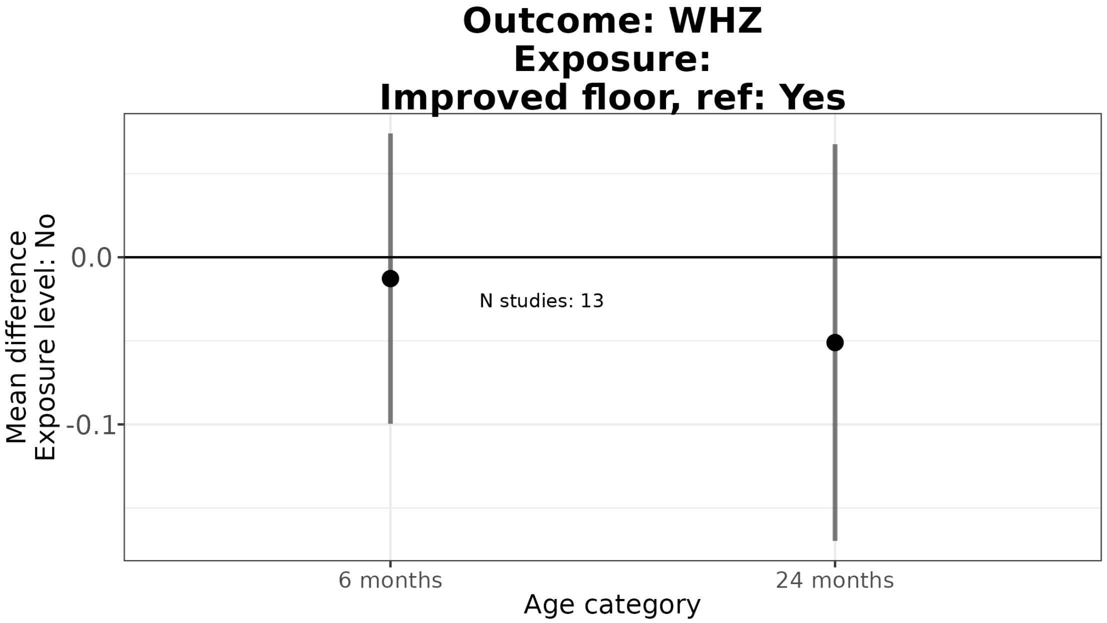
Outcome: WHZ
Exposure:
Improved floor, ref: Yes
Mean difference
Exposure level: No
0.0
-0.1
N studies: 13
6 months
24 months
Age category

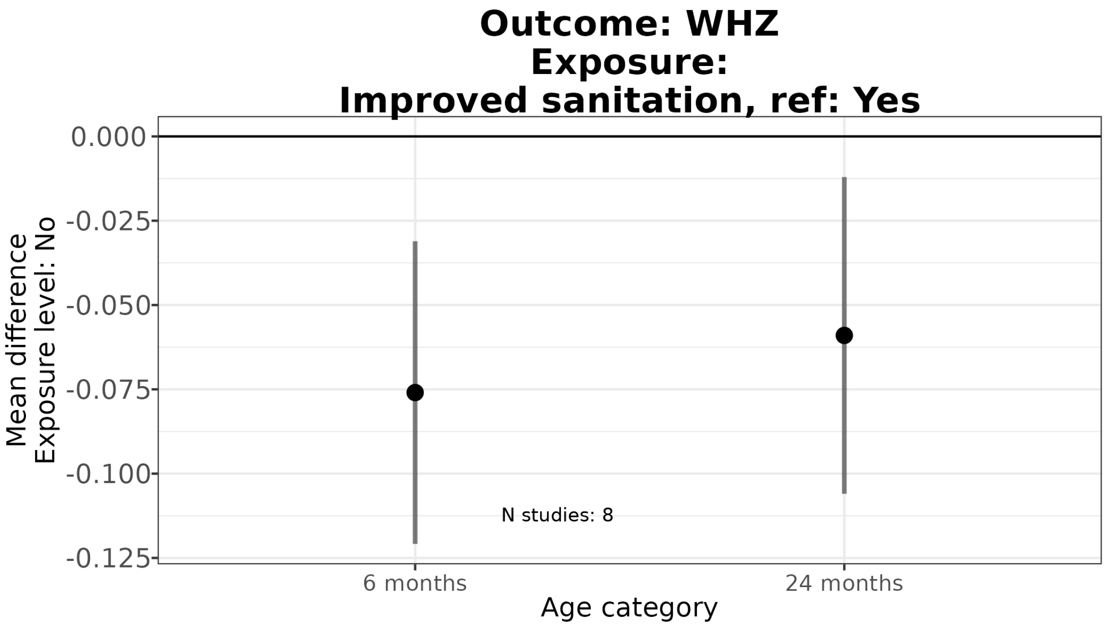

Outcome: WHZ
Exposure:
Improved sanitation, ref: Yes
Mean difference
Exposure level: No
0.000
-0.025
-0.050
-0.075
-0.100
-0.125
N studies: 8
6 months
24 months
Age category


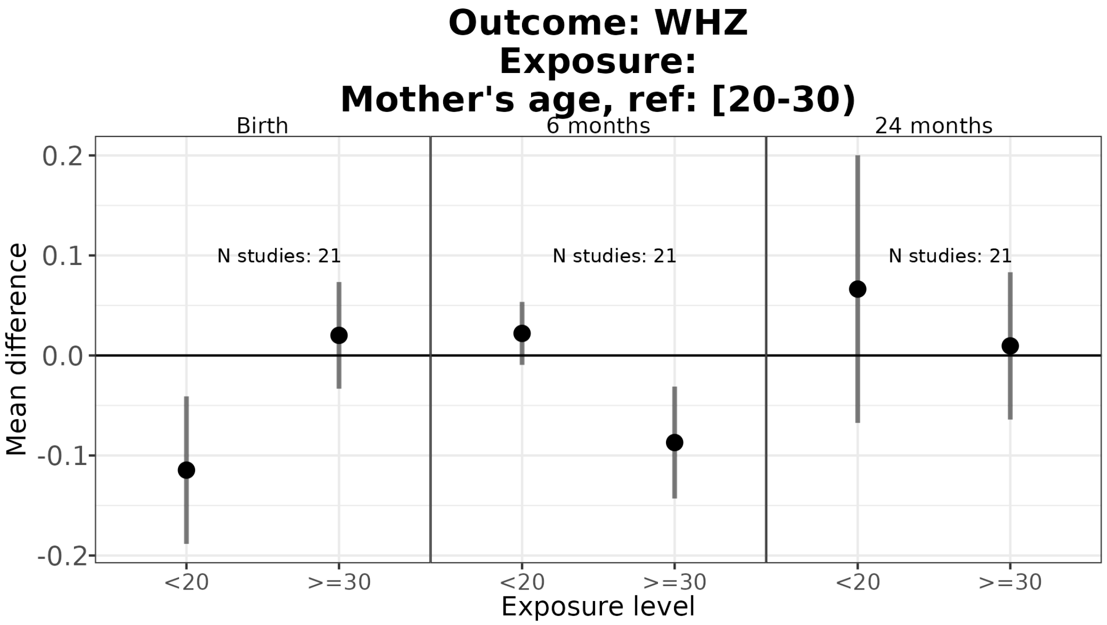

Outcome: WHZ
Exposure:
Mother's age, ref: [20-30)
Birth
6 months
24 months
Mean difference
0.2
0.1
0.0
-0.1
-0.2
N studies: 21
N studies: 21
N studies: 21
<20
>=30
<20
>=30
<20
>=30
Exposure level

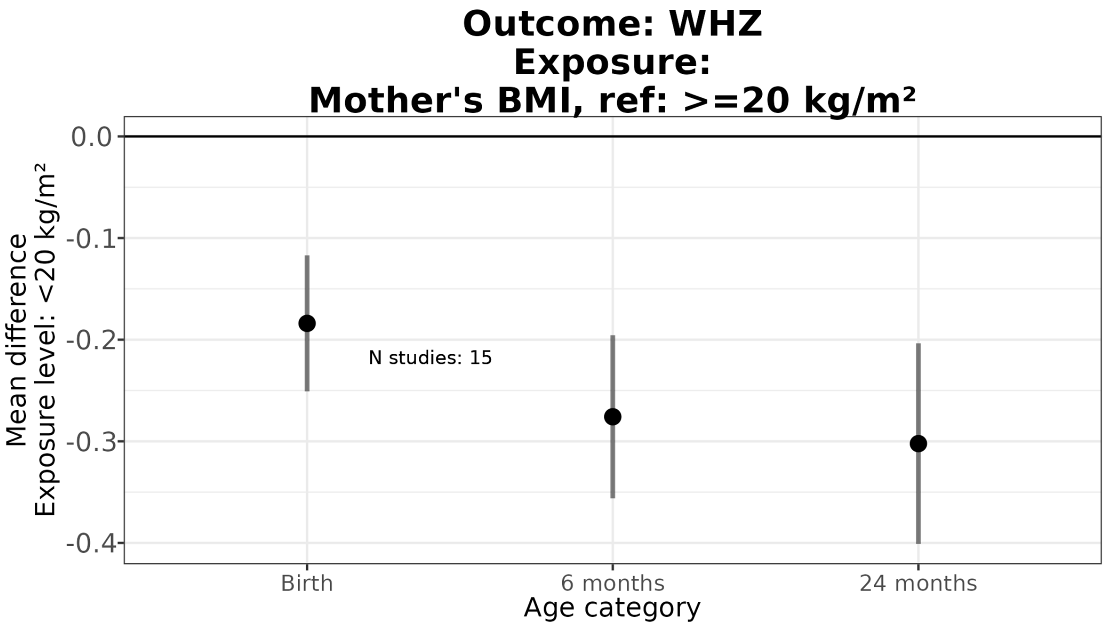
Outcome: WHZ
Exposure:
Mother's BMI, ref: >=20 kg/m²
Mean difference
Exposure level: <20 kg/m²
0.0
-0.1
-0.2
-0.3
-0.4
N studies: 15
Birth
6 months
24 months
Age category

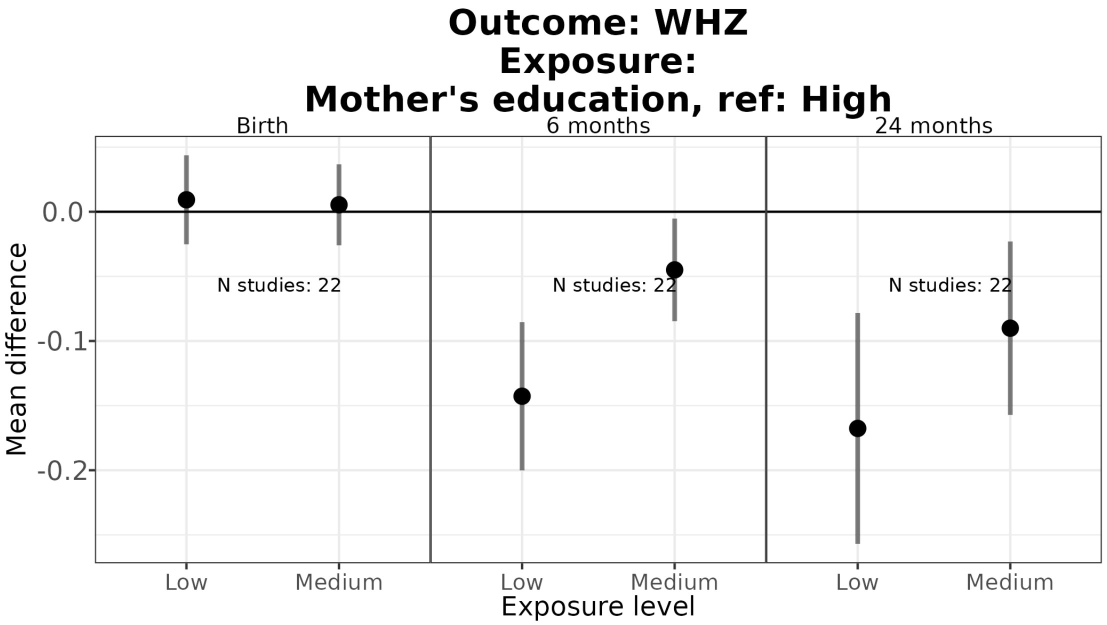
Outcome: WHZ
Exposure:
Mother's education, ref: High
Birth
6 months
24 months
Mean difference
0.0
-0.1
-0.2
N studies: 22
N studies: 22
N studies: 22
Low
Medium
Low
Medium
Low
Medium
Exposure level

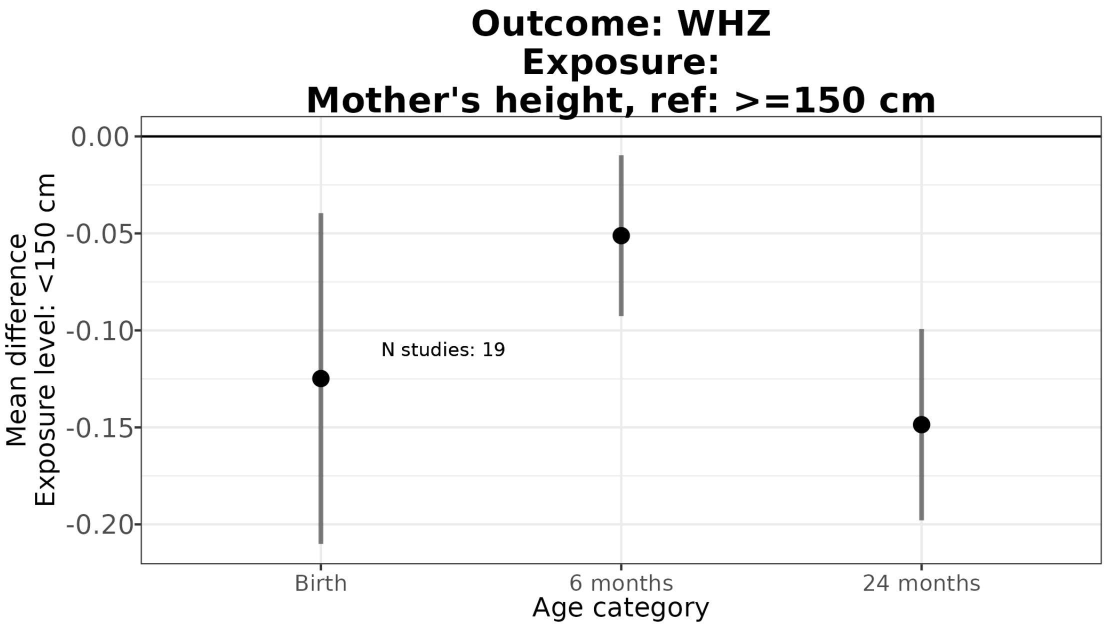
Outcome: WHZ
Exposure:
Mother's height, ref: >=150 cm
0.00
-0.05
-0.10
-0.15
-0.20
Mean difference
Exposure level: <150 cm
N studies: 19
Birth
6 months
24 months
Age category

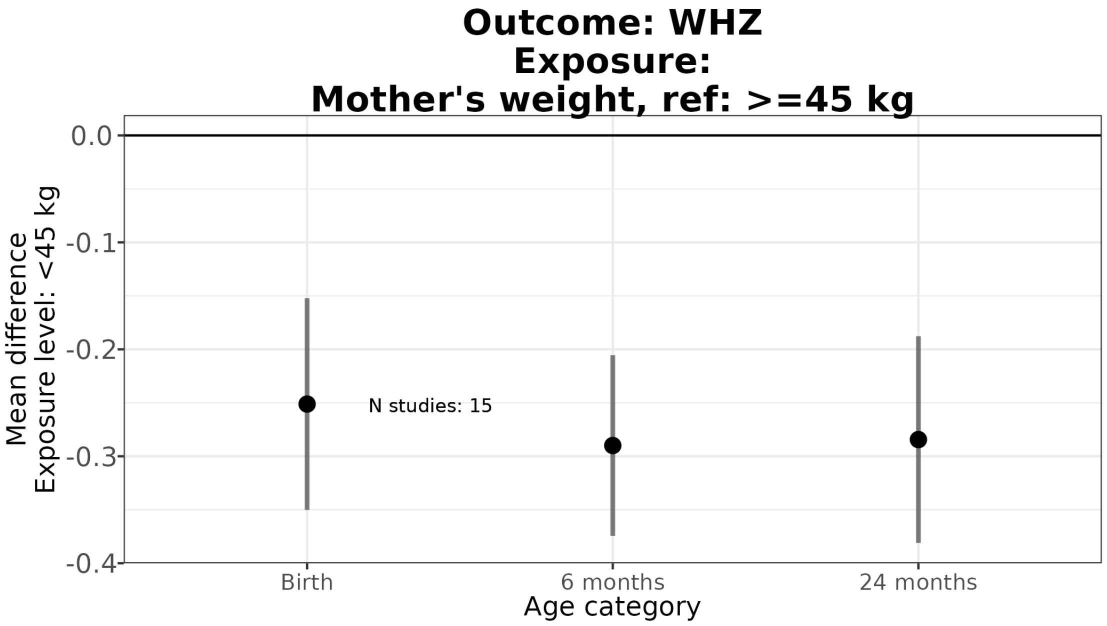
Outcome: WHZ
Exposure:
Mother's weight, ref: >=45 kg
0.0
-0.1
-0.2
-0.3
-0.4
Mean difference
Exposure level: <45 kg
N studies: 15
Birth
6 months
24 months
Age category

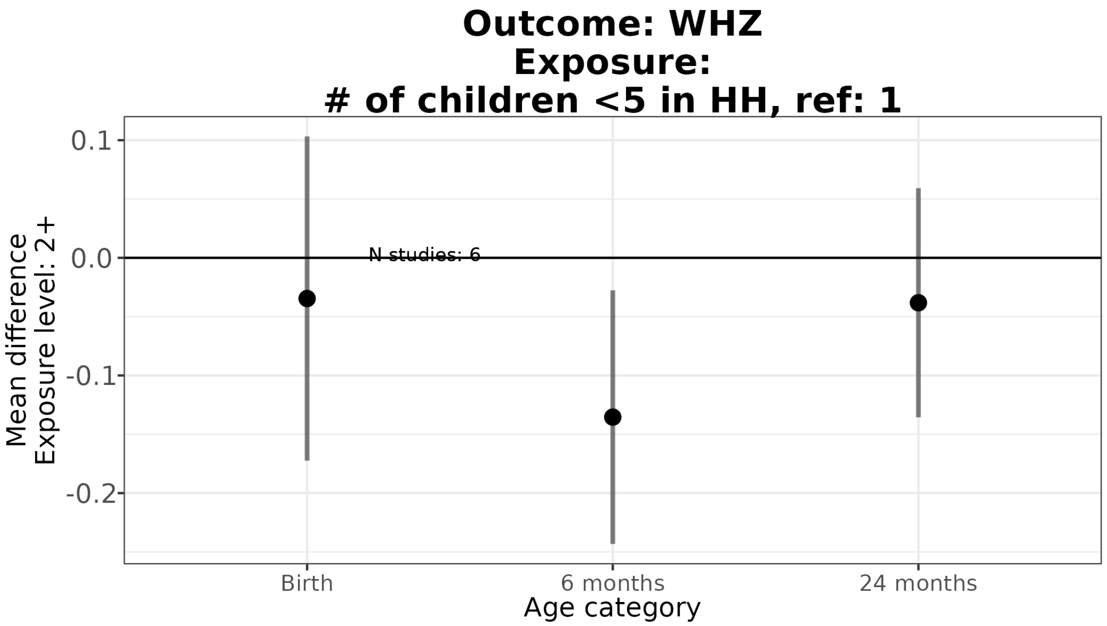
Outcome: WHZ
Exposure:
# of children <5 in HH, ref: 1
Mean difference
Exposure level: 2+
N studies: 6
0.1
0.0
-0.1
-0.2
Birth
6 months
24 months
Age category

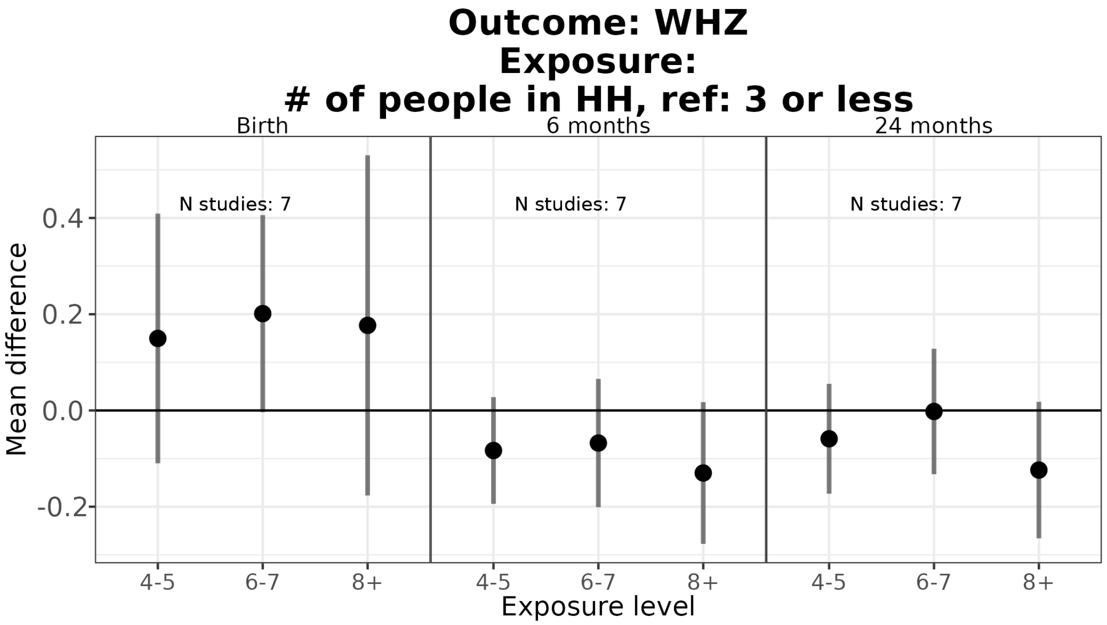
Outcome: WHZ
Exposure:
# of people in HH, ref: 3 or less
Birth
6 months
24 months
N studies: 7
N studies: 7
N studies: 7
Mean difference
0.4
0.2
0.0
-0.2
4-5
6-7
8+
4-5
6-7
8+
4-5
6-7
8+
Exposure level

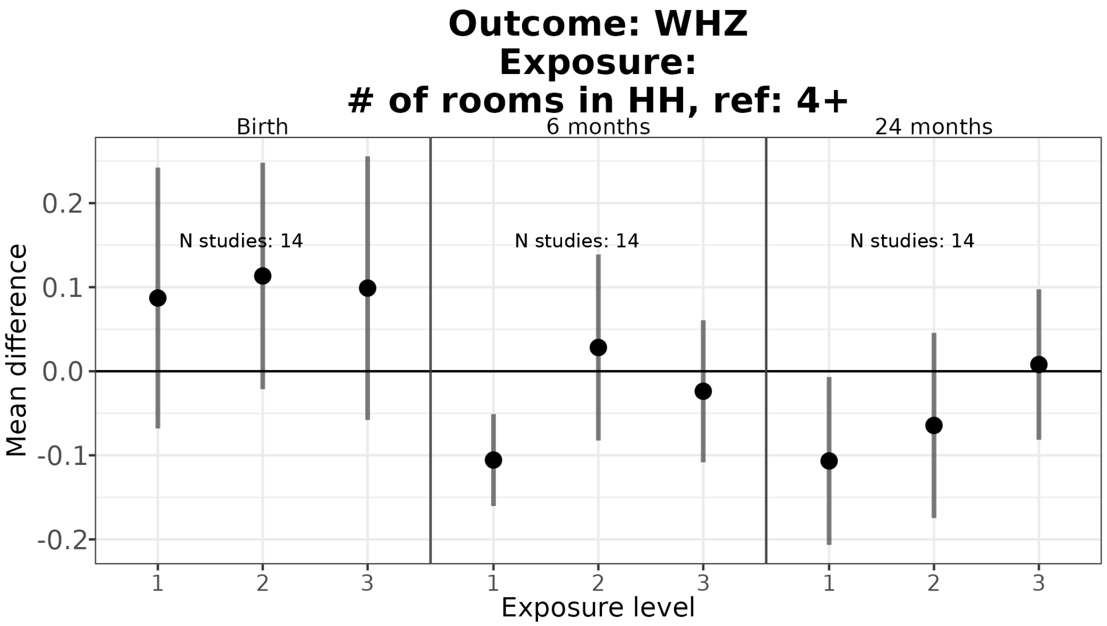

Outcome: WHZ
Exposure:
# of rooms in HH, ref: 4+
Birth
6 months
24 months
N studies: 14
N studies: 14
N studies: 14
Mean difference
0.2
0.1
0.0
-0.1
-0.2
1
2
3
Exposure level


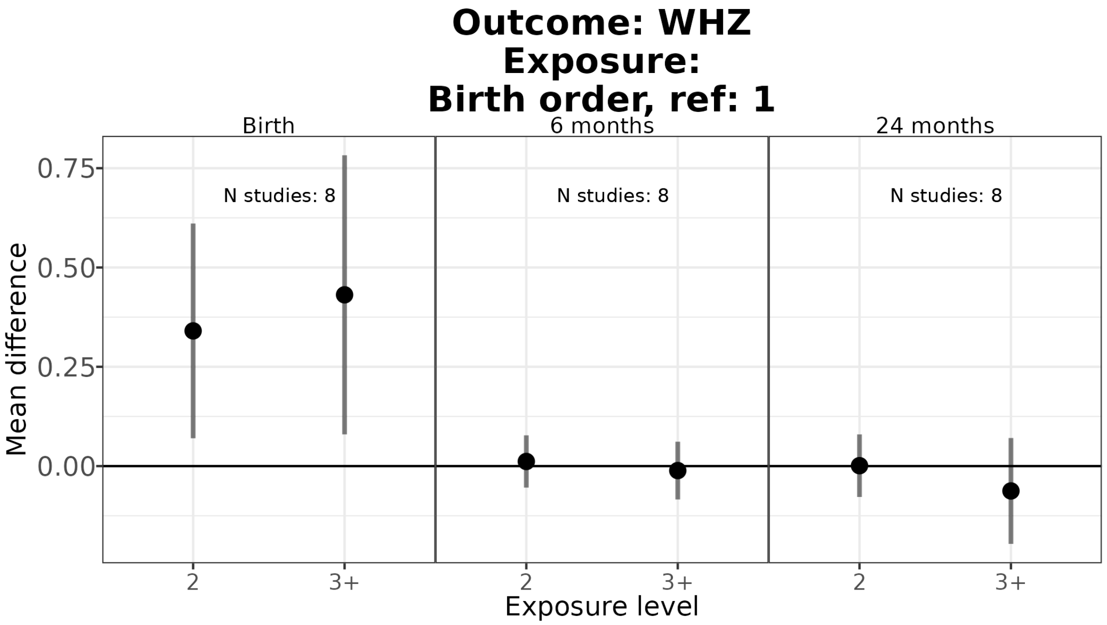

Outcome: WHZ
Exposure:
Birth order, ref: 1
Birth
6 months
24 months
N studies: 8
N studies: 8
N studies: 8
Mean difference
0.75
0.50
0.25
0.00
2
3+
Exposure level

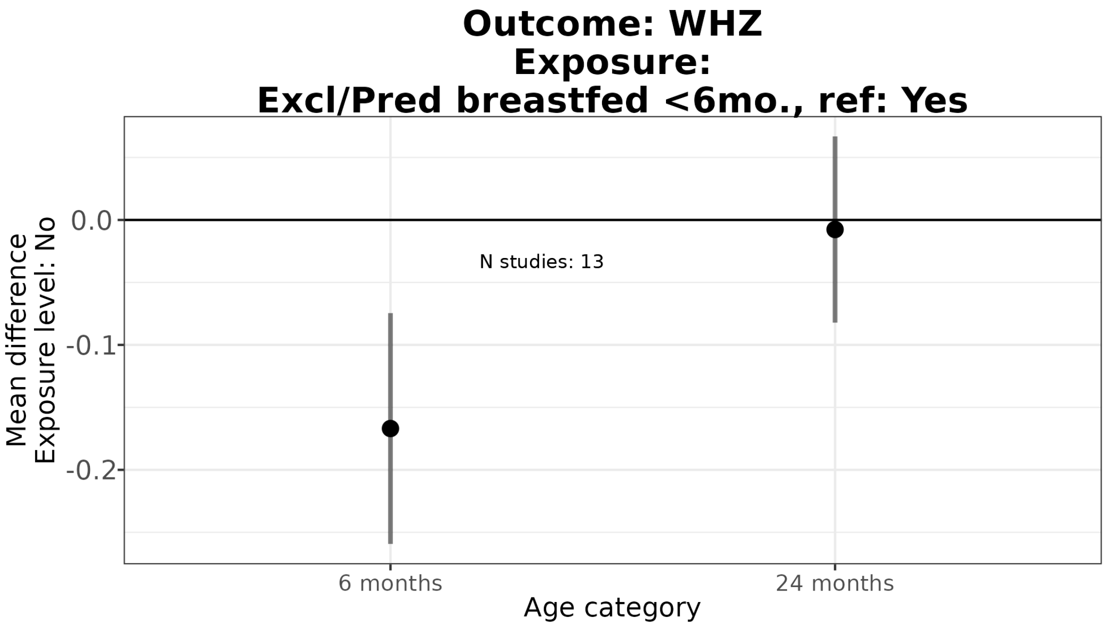
Outcome: WHZ
Exposure:
Excl/Pred breastfed <6mo., ref: Yes
Mean difference
Exposure level: No
0.0
-0.1
-0.2
N studies: 13
6 months
24 months
Age category

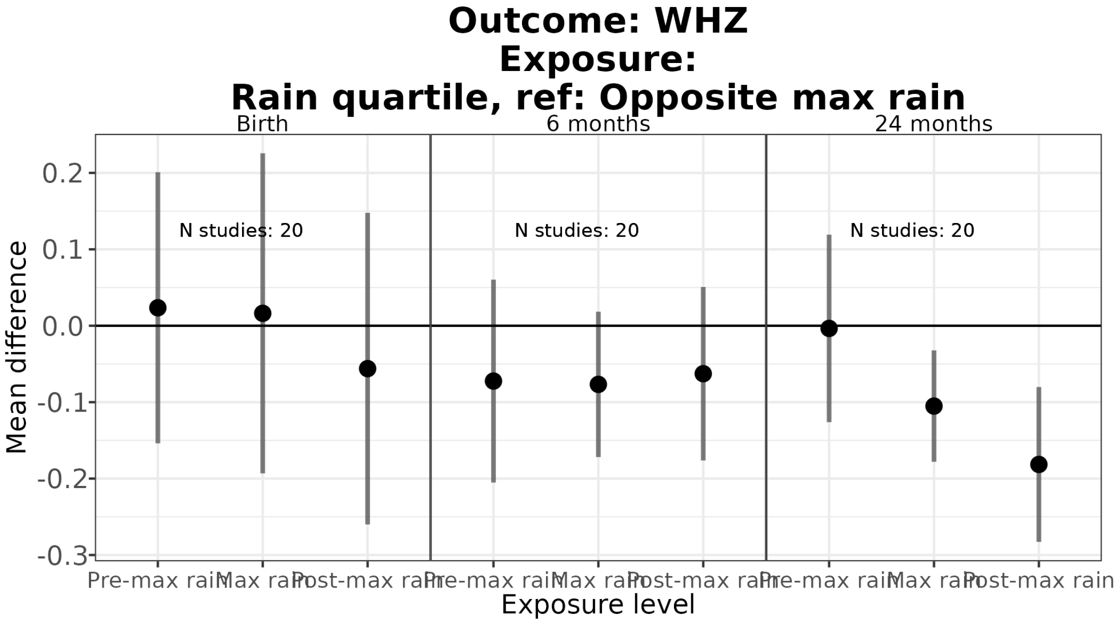
Outcome: WHZ
Exposure:
Rain quartile, ref: Opposite max rain
Birth
6 months
24 months
Mean difference
0.2
0.1
0.0
-0.1
-0.2
-0.3
N studies: 20
N studies: 20
N studies: 20
Pre-max rain
Max rain
Post-max rain
Exposure level

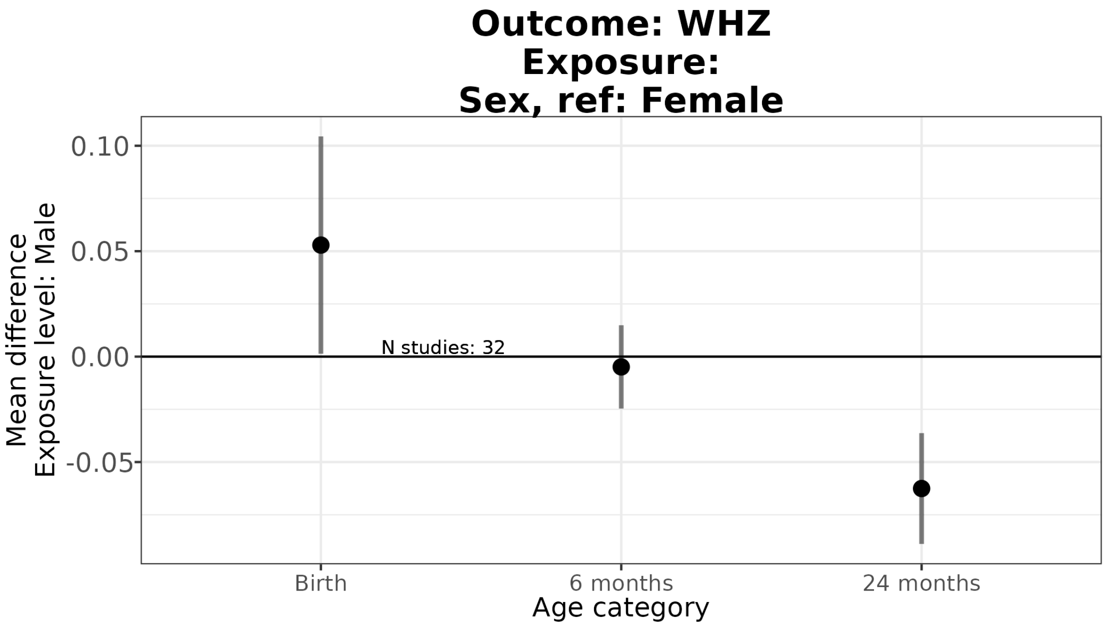
Outcome: WHZ
Exposure:
Sex, ref: Female
Mean difference
Exposure level: Male
0.10
0.05
0.00
-0.05
N studies: 32
Birth
6 months
24 months
Age category

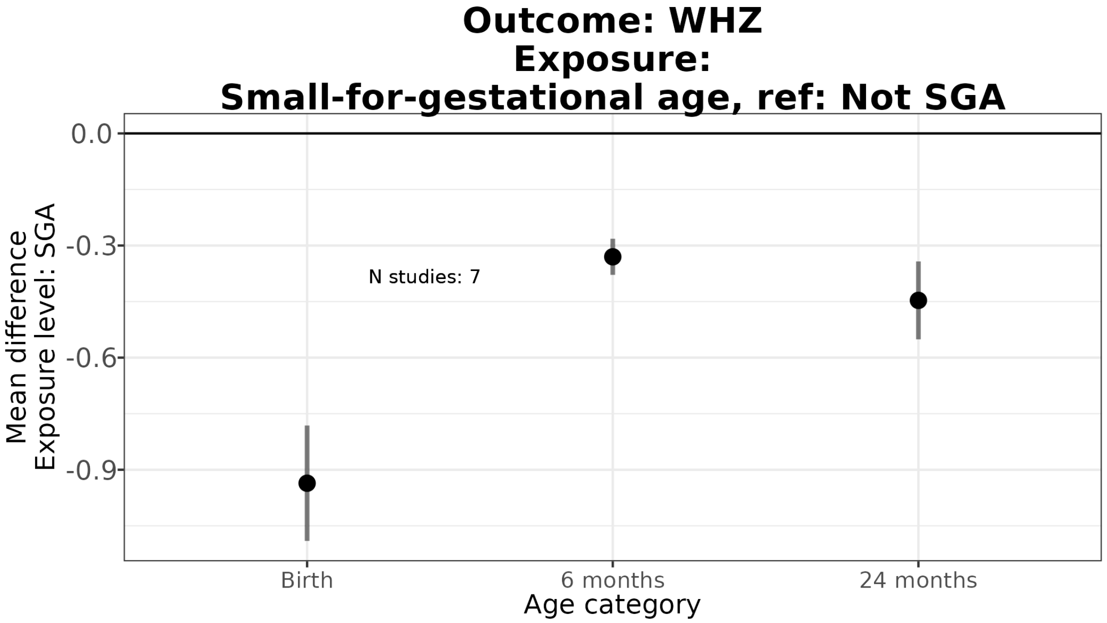
Outcome: WHZ
Exposure:
Small-for-gestational age, ref: Not SGA
Mean difference
Exposure level: SGA
0.0
-0.3
-0.6
-0.9
N studies: 7
Birth
6 months
24 months
Age category

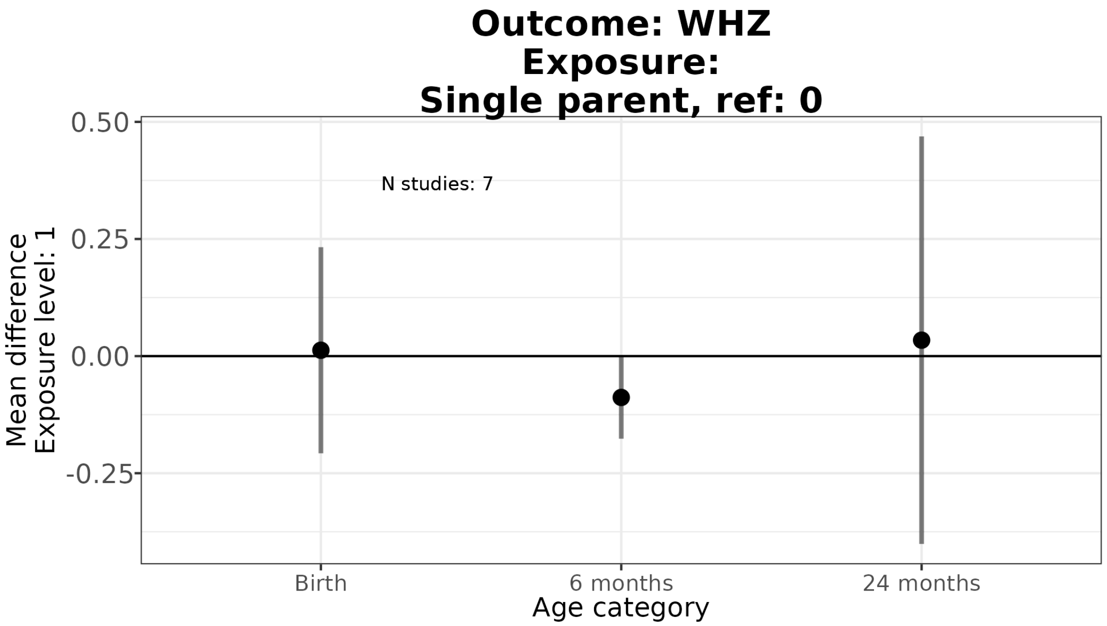

Outcome: WHZ
Exposure:
Single parent, ref: 0
N studies: 7
Mean difference
Exposure level: 1
0.50
0.25
0.00
-0.25
Birth
6 months
24 months
Age category


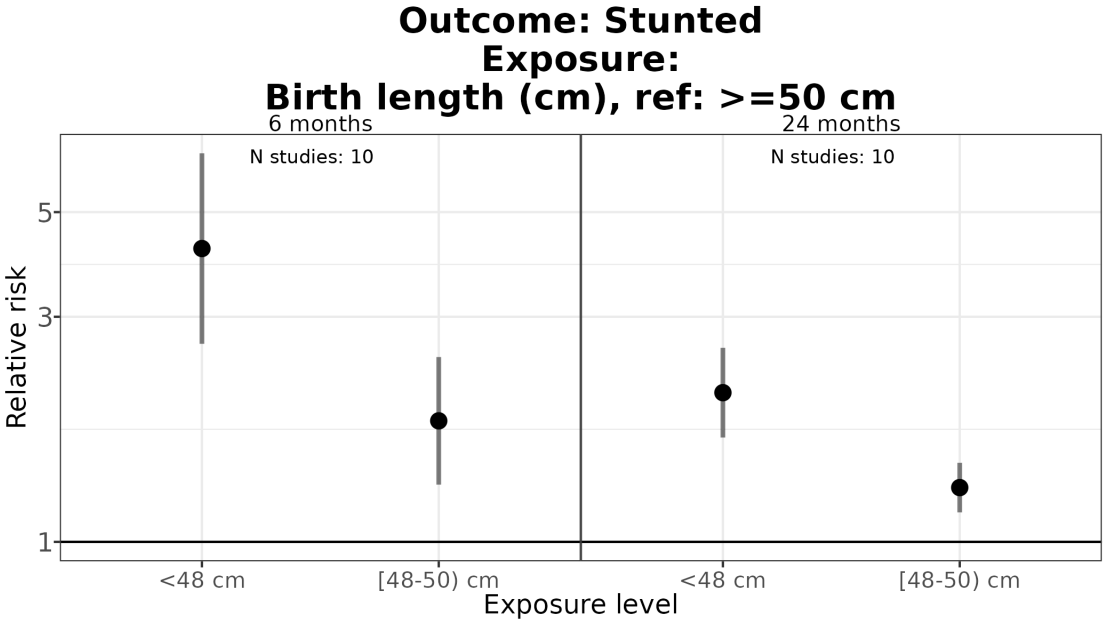

Outcome: Stunted
Exposure:
Birth length (cm), ref: >=50 cm
6 months
24 months
N studies: 10
N studies: 10
Relative risk
5
3
1
<48 cm
[48-50) cm
<48 cm
[48-50) cm
Exposure level

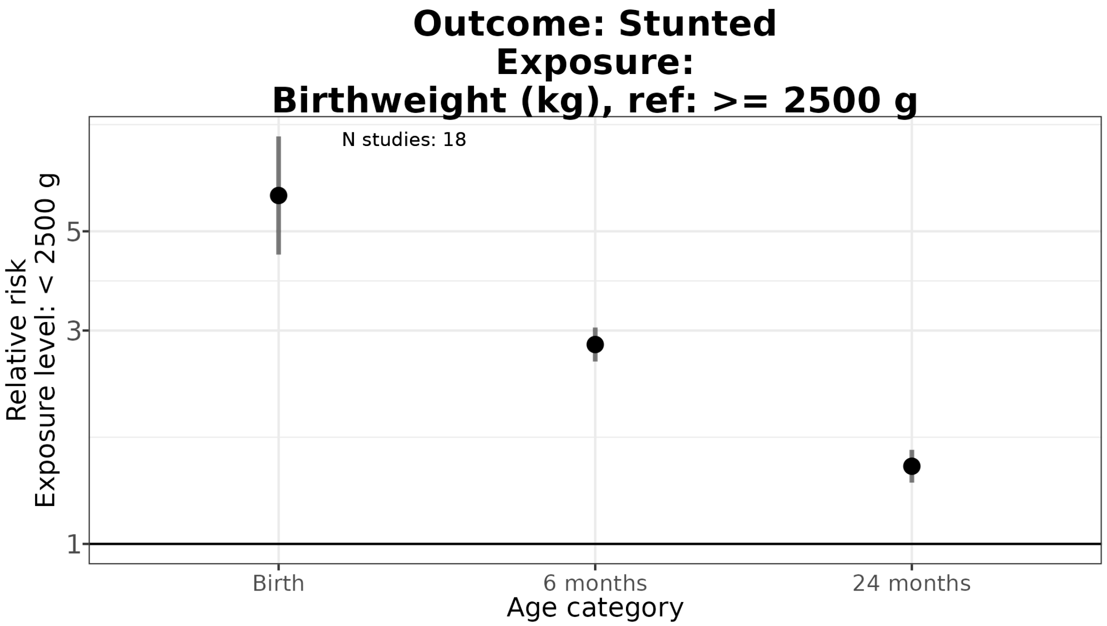
Outcome: Stunted
Exposure:
Birthweight (kg), ref: >= 2500 g
N studies: 18
Relative risk
Exposure level: < 2500 g
5
3
1
Birth
6 months
24 months
Age category

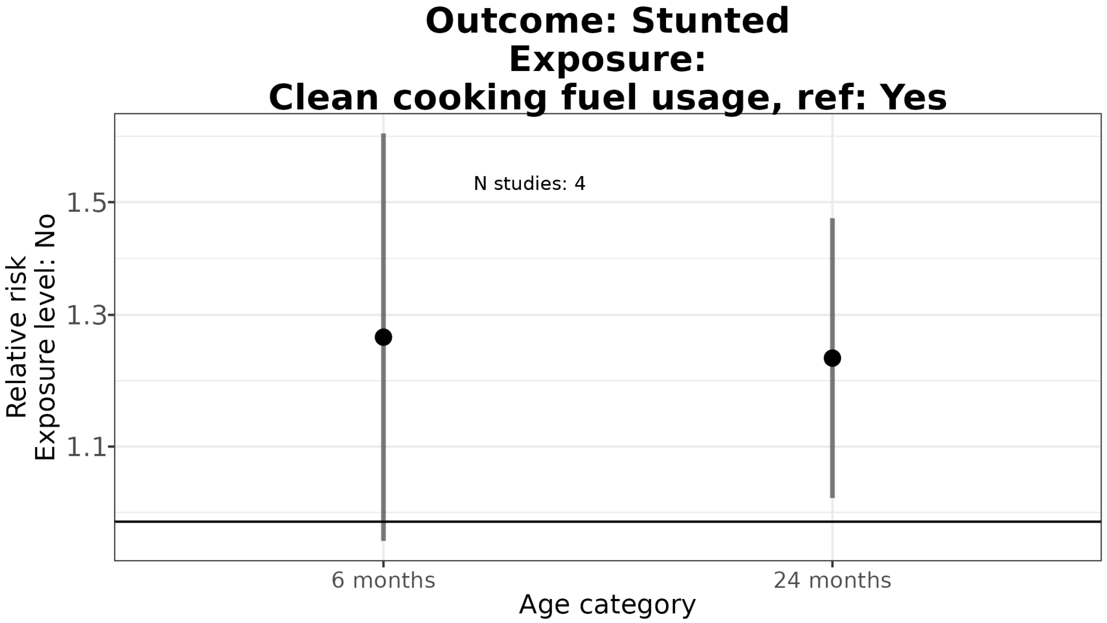
Outcome: Stunted
Exposure:
Clean cooking fuel usage, ref: Yes
N studies: 4
Relative risk
Exposure level: No
1.5
1.3
1.1
6 months
24 months
Age category

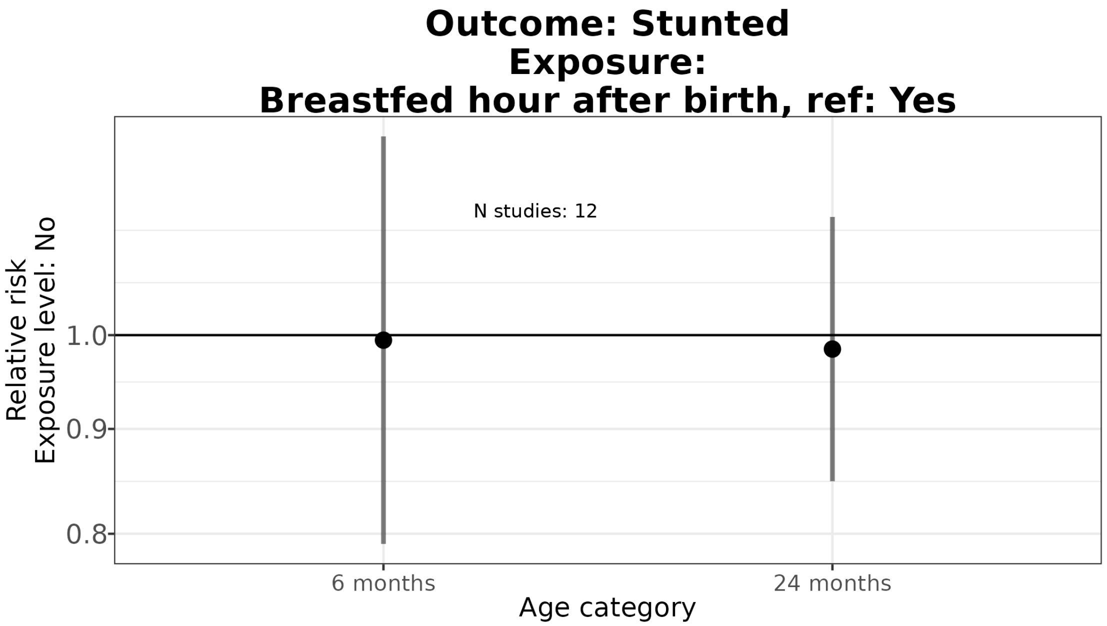

Outcome: Stunted
Exposure:
Breastfed hour after birth, ref: Yes
N studies: 12
Relative risk
Exposure level: No
1.0
0.9
0.8
6 months
24 months
Age category


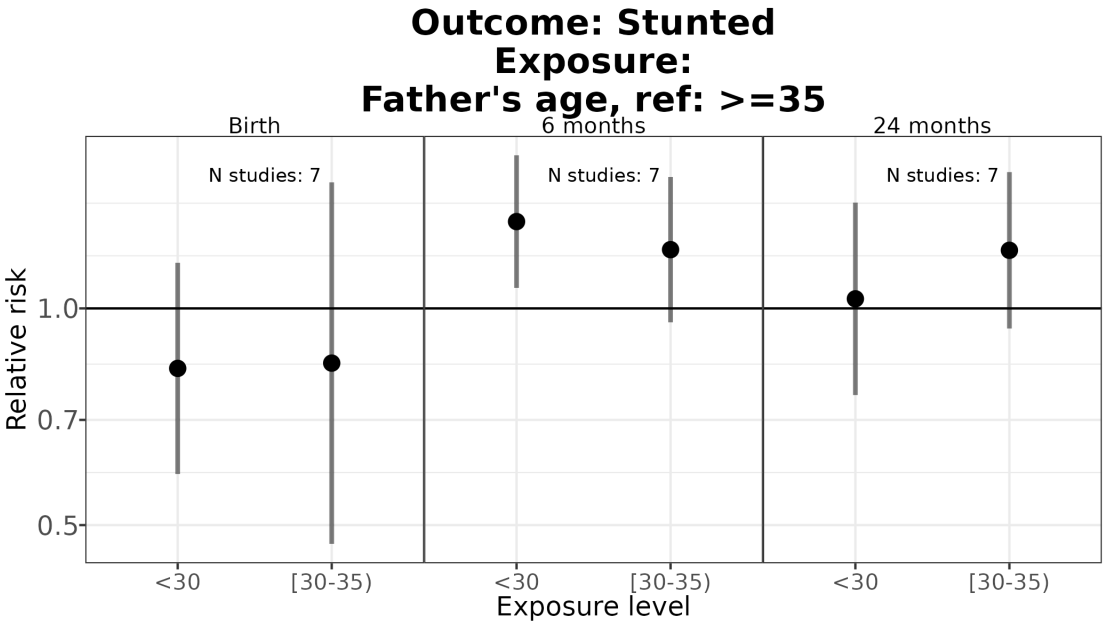

Outcome: Stunted
Exposure:
Father's age, ref: >=35
Birth
6 months
24 months
N studies: 7
N studies: 7
N studies: 7
Relative risk
1.0
0.7
0.5
<30
[30-35)
<30
[30-35)
<30
[30-35)
Exposure level

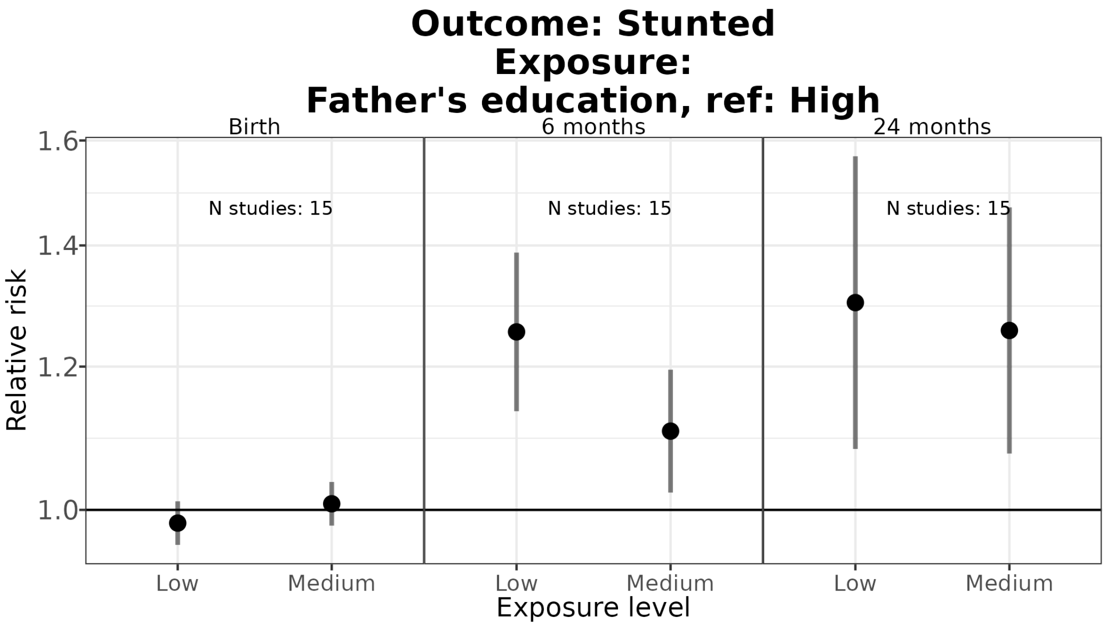
Outcome: Stunted
Exposure:
Father's education, ref: High
Birth
6 months
24 months
N studies: 15
N studies: 15
N studies: 15
1.6
1.4
1.2
1.0
Relative risk
Low
Medium
Low
Medium
Low
Medium
Exposure level

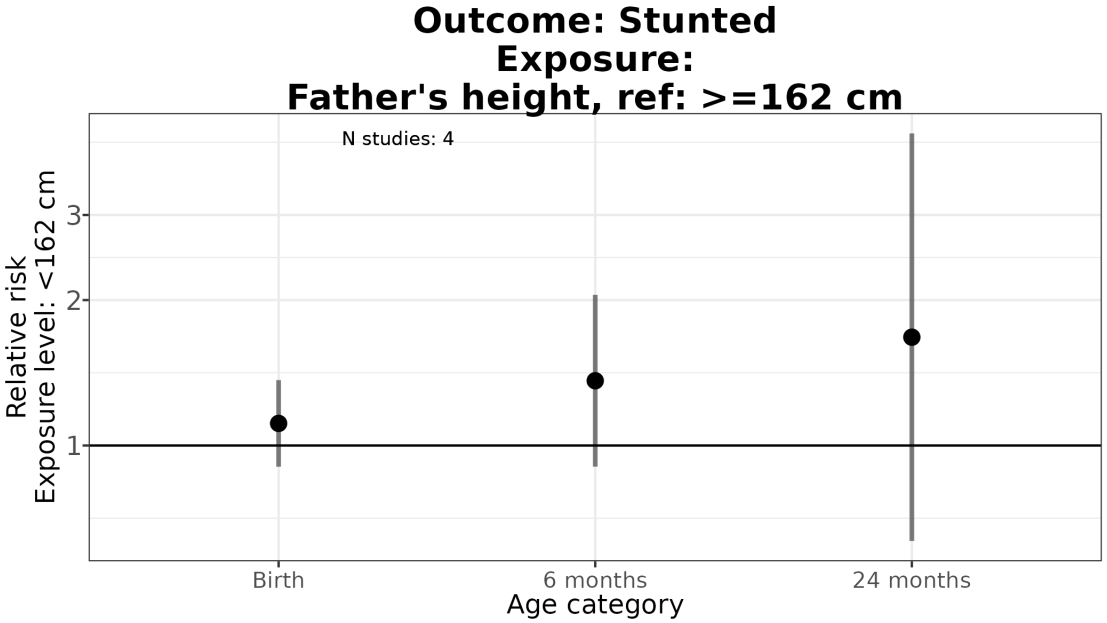
Outcome: Stunted
Exposure:
Father's height, ref: >=162 cm
N studies: 4
3
2
1
Relative risk
Exposure level: <162 cm
Birth
6 months
24 months
Age category

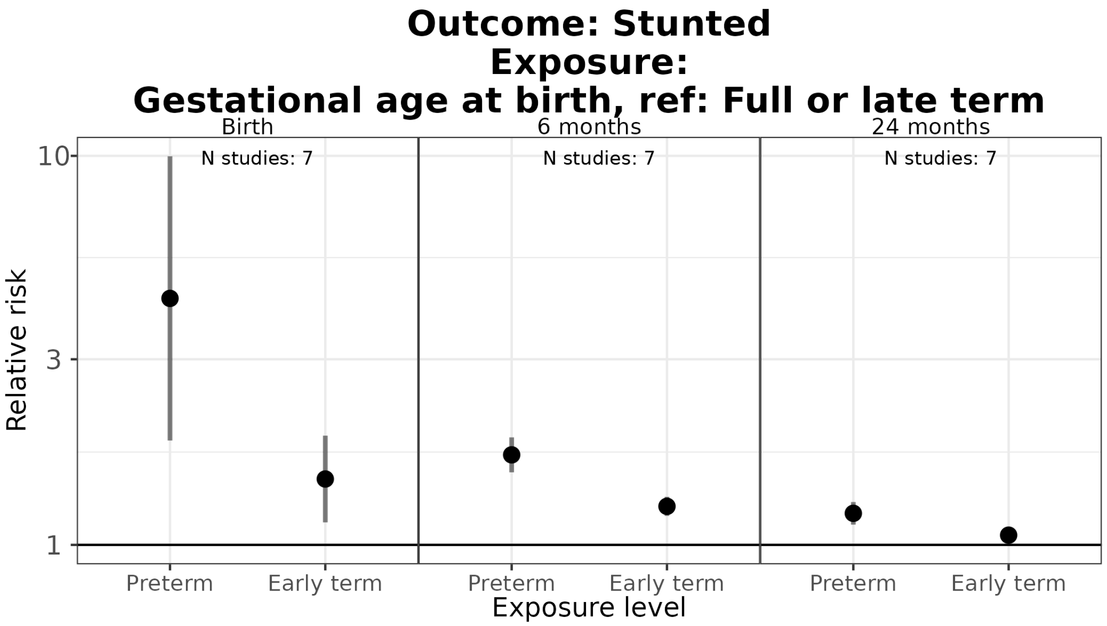
Outcome: Stunted
Exposure:
Gestational age at birth, ref: Full or late term
Birth
6 months
24 months
N studies: 7
N studies: 7
N studies: 7
10
3
1
Relative risk
Preterm
Early term
Preterm
Early term
Preterm
Early term
Exposure level

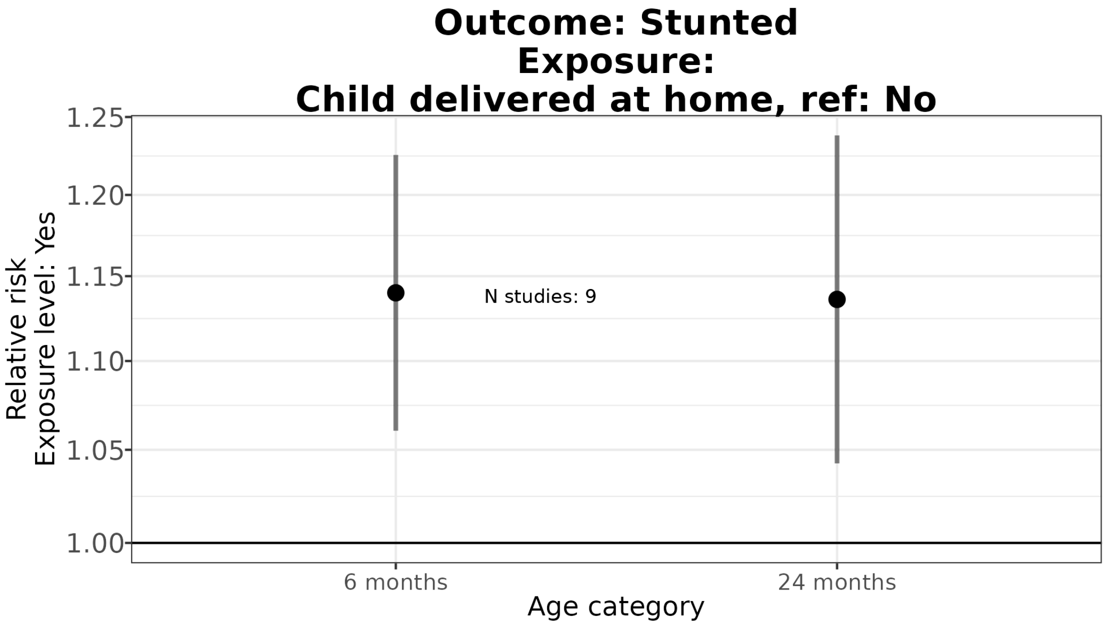
Outcome: Stunted
Exposure:
Child delivered at home, ref: No
1.25
1.20
1.15
1.10
1.05
1.00
Relative risk
Exposure level: Yes
N studies: 9
6 months
24 months
Age category

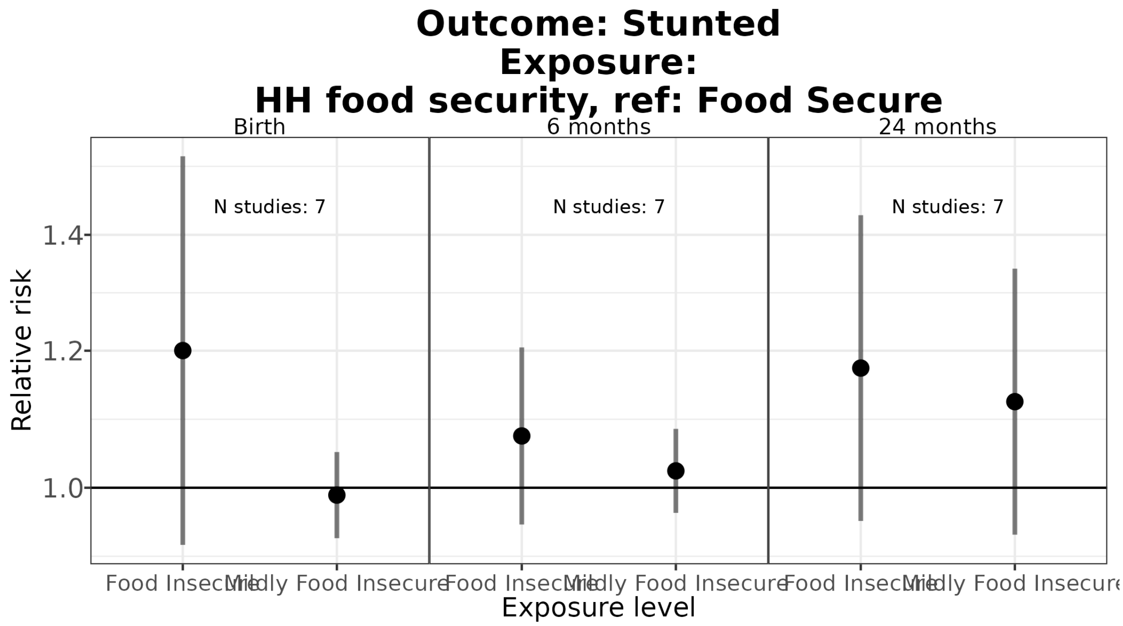
Outcome: Stunted
Exposure:
HH food security, ref: Food Secure
Birth
6 months
24 months
N studies: 7
N studies: 7
N studies: 7
Relative risk
1.4
1.2
1.0
Food Insecure
Mildly Food Insecure
Food Insecure
Mildly Food Insecure
Food Insecure
Mildly Food Insecure
Exposure level

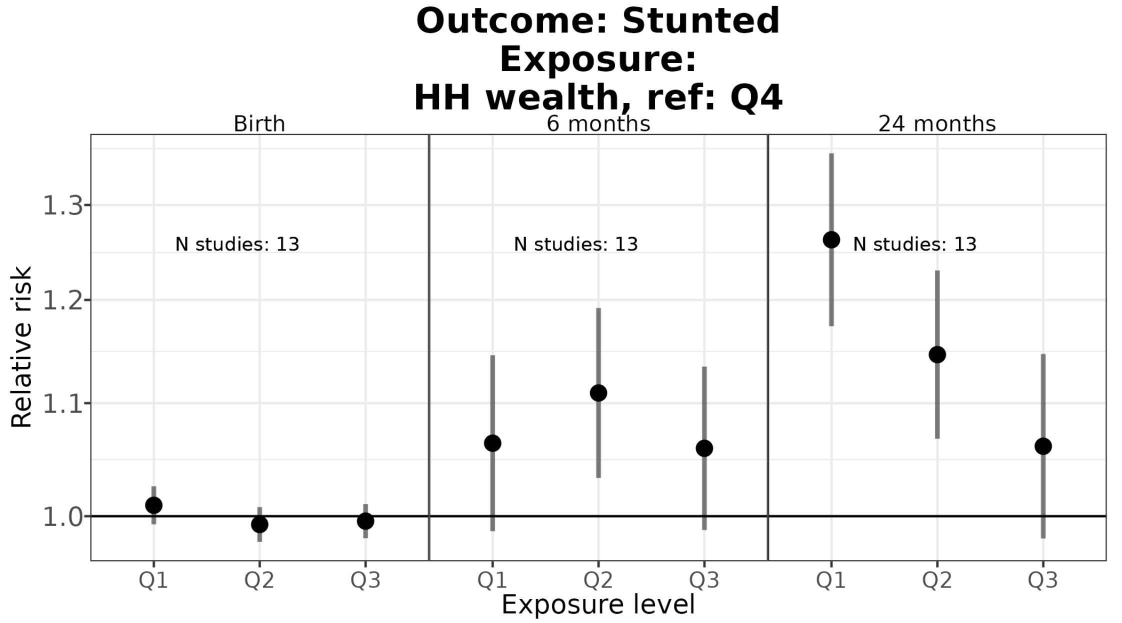
Outcome: Stunted
Exposure:
HH wealth, ref: Q4
Birth
6 months
24 months
N studies: 13
N studies: 13
N studies: 13
Relative risk
1.3
1.2
1.1
1.0
Q1
Q2
Q3
Q1
Q2
Q3
Q1
Q2
Q3
Exposure level

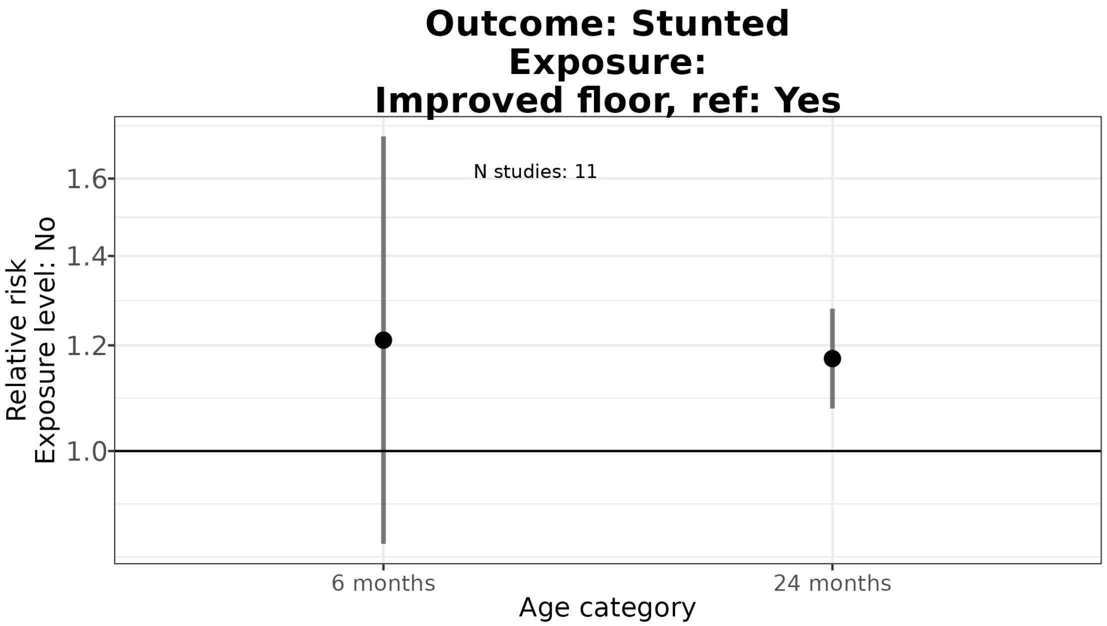
Outcome: Stunted
Exposure:
Improved floor, ref: Yes
N studies: 11
Relative risk
Exposure level: No
1.6
1.4
1.2
1.0
6 months
24 months
Age category

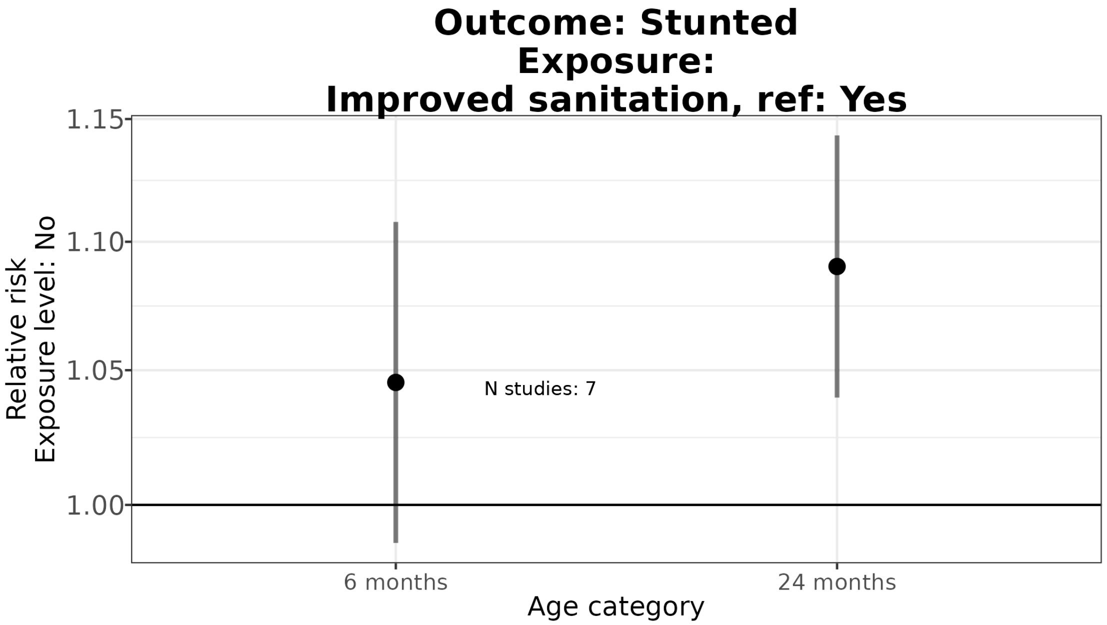
Outcome: Stunted
Exposure:
Improved sanitation, ref: Yes
N studies: 7
Relative risk
Exposure level: No
1.15
1.10
1.05
1.00
6 months
24 months
Age category

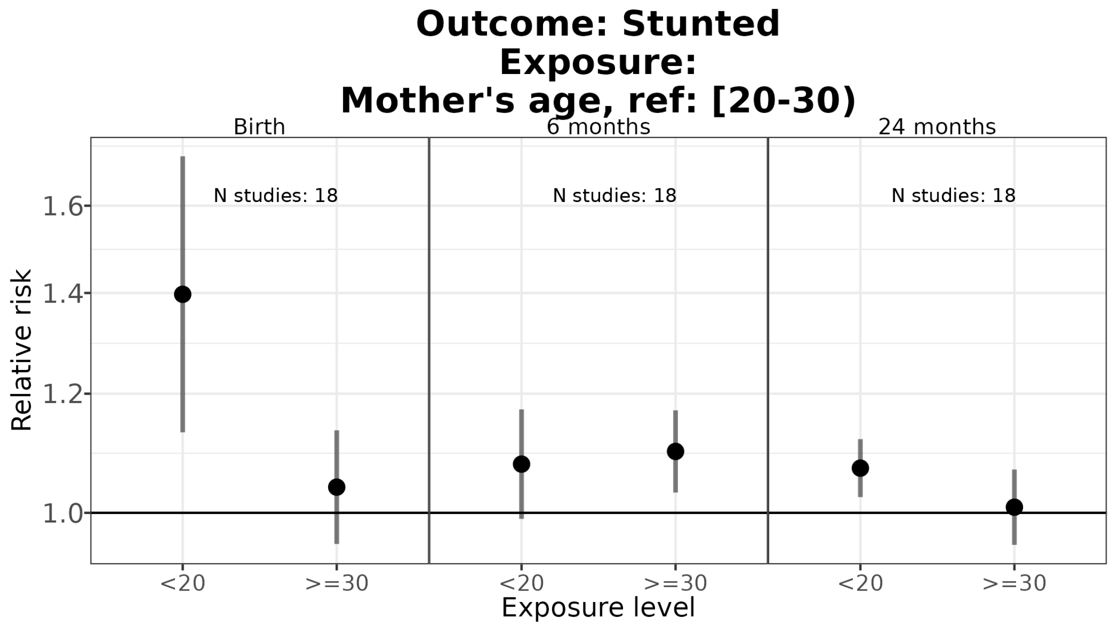

Outcome: Stunted
Exposure:
Mother's age, ref: [20-30)
Birth
6 months
24 months
N studies: 18
N studies: 18
N studies: 18
1.6
1.4
1.2
1.0
Relative risk
<20
>=30
<20
>=30
<20
>=30
Exposure level


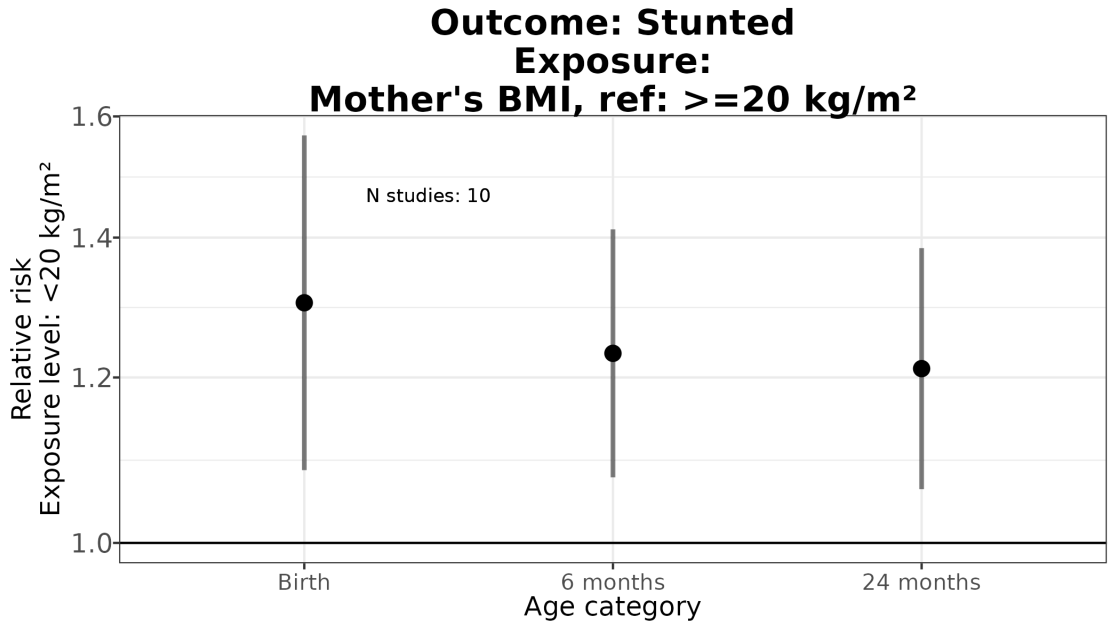

Outcome: Stunted
Exposure:
Mother's BMI, ref: >=20 kg/m²
N studies: 10
1.6
1.4
1.2
1.0
Relative risk
Exposure level: <20 kg/m²
Birth
6 months
24 months
Age category

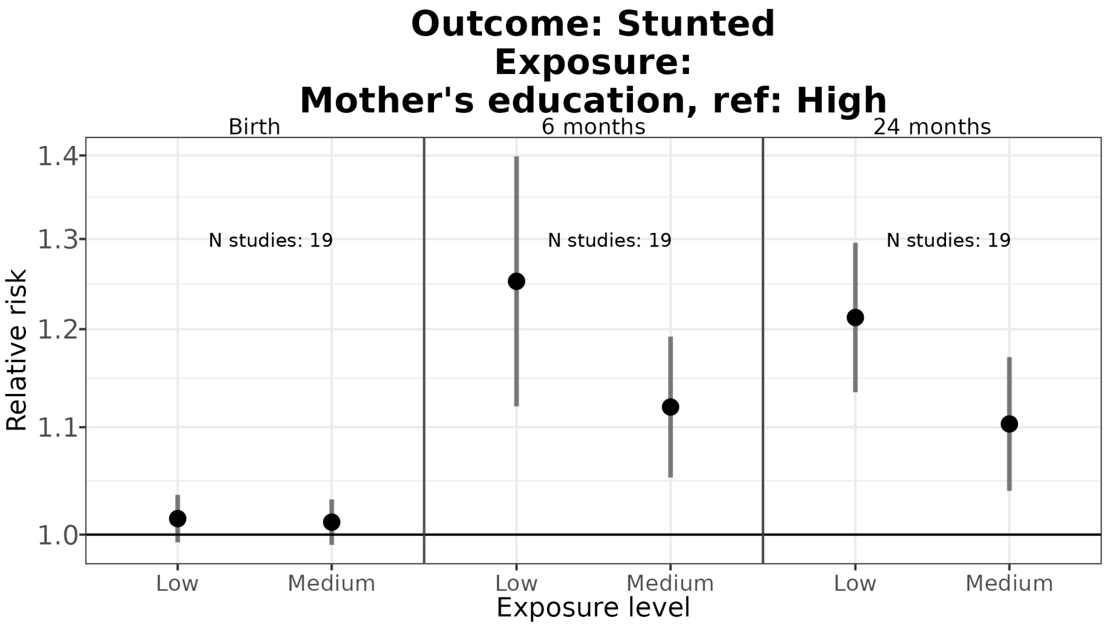
Outcome: Stunted
Exposure:
Mother's education, ref: High
Birth
6 months
24 months
1.4
1.3
1.2
1.1
1.0
Relative risk
N studies: 19
N studies: 19
N studies: 19
Low
Medium
Low
Medium
Low
Medium
Exposure level

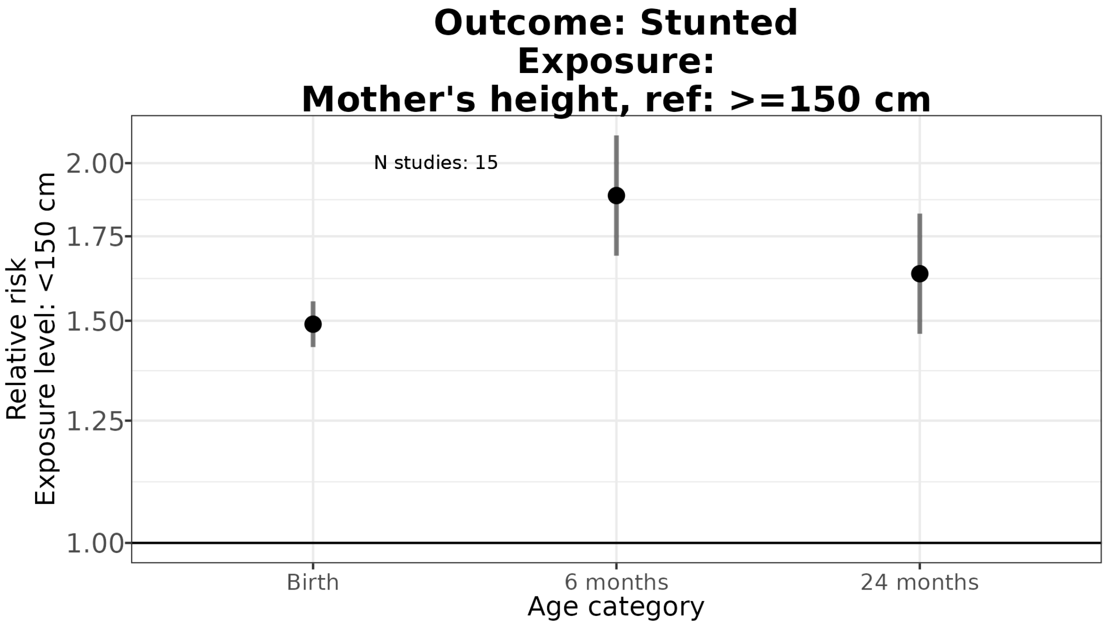
Outcome: Stunted
Exposure:
Mother's height, ref: >=150 cm
2.00
1.75
1.50
1.25
1.00
Relative risk
Exposure level: <150 cm
N studies: 15
Birth
6 months
24 months
Age category

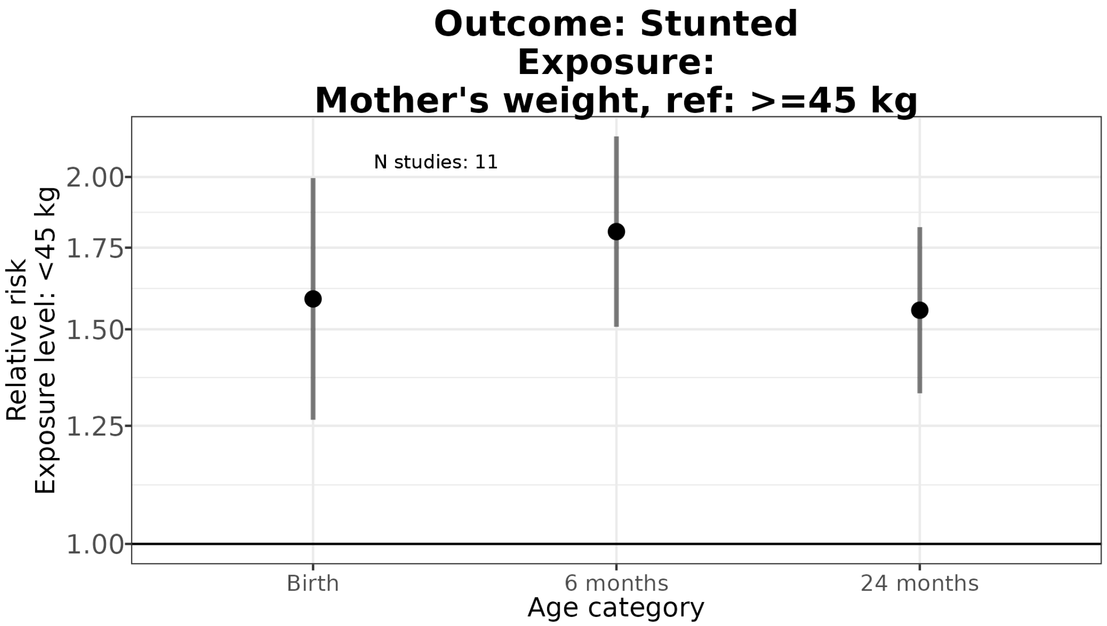
Outcome: Stunted
Exposure:
Mother's weight, ref: >=45 kg
N studies: 11
Relative risk
Exposure level: <45 kg
2.00
1.75
1.50
1.25
1.00
Birth
6 months
24 months
Age category

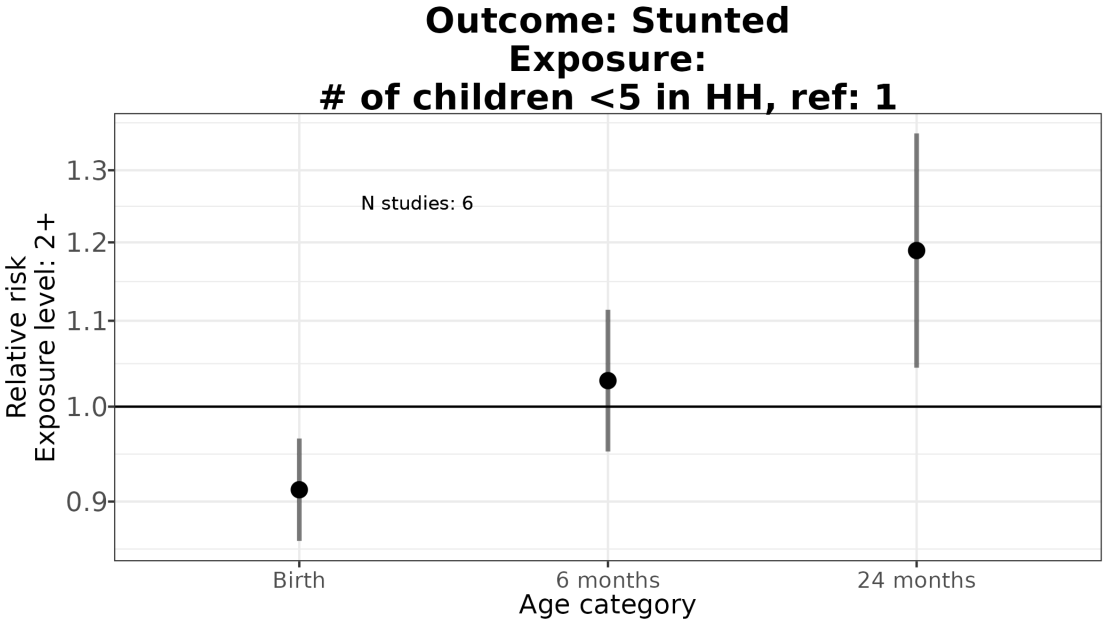
Outcome: Stunted
Exposure:
# of children <5 in HH, ref: 1
N studies: 6
Relative risk
Exposure level: 2+
1.3
1.2
1.1
1.0
0.9
Birth
6 months
24 months
Age category

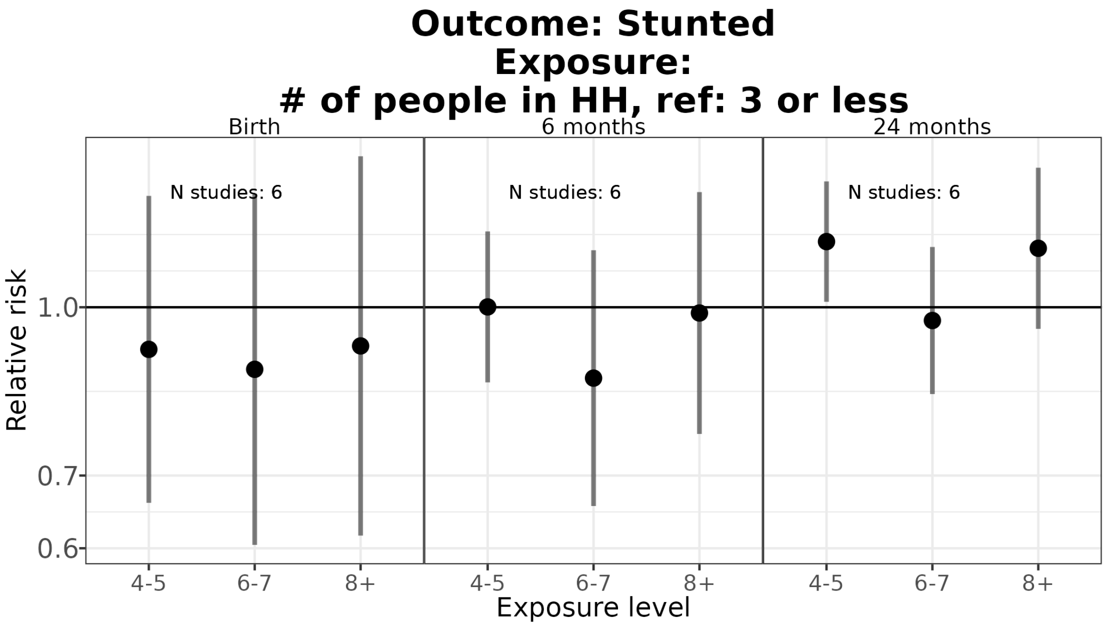
Outcome: Stunted
Exposure:
# of people in HH, ref: 3 or less
Birth
6 months
24 months
N studies: 6
N studies: 6
N studies: 6
Relative risk
1.0
0.7
0.6
4-5
6-7
8+
4-5
6-7
8+
4-5
6-7
8+
Exposure level

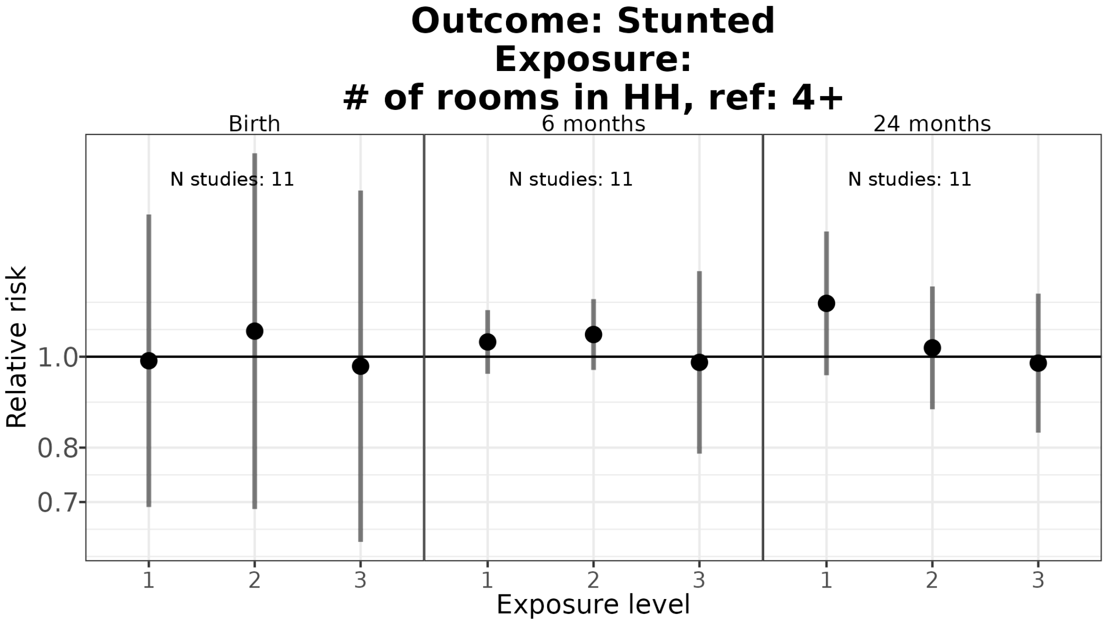
Outcome: Stunted
Exposure:
# of rooms in HH, ref: 4+
Birth
6 months
24 months
N studies: 11
N studies: 11
N studies: 11
Relative risk
1.0
0.8
0.7
1
2
3
1
2
3
1
2
3
Exposure level

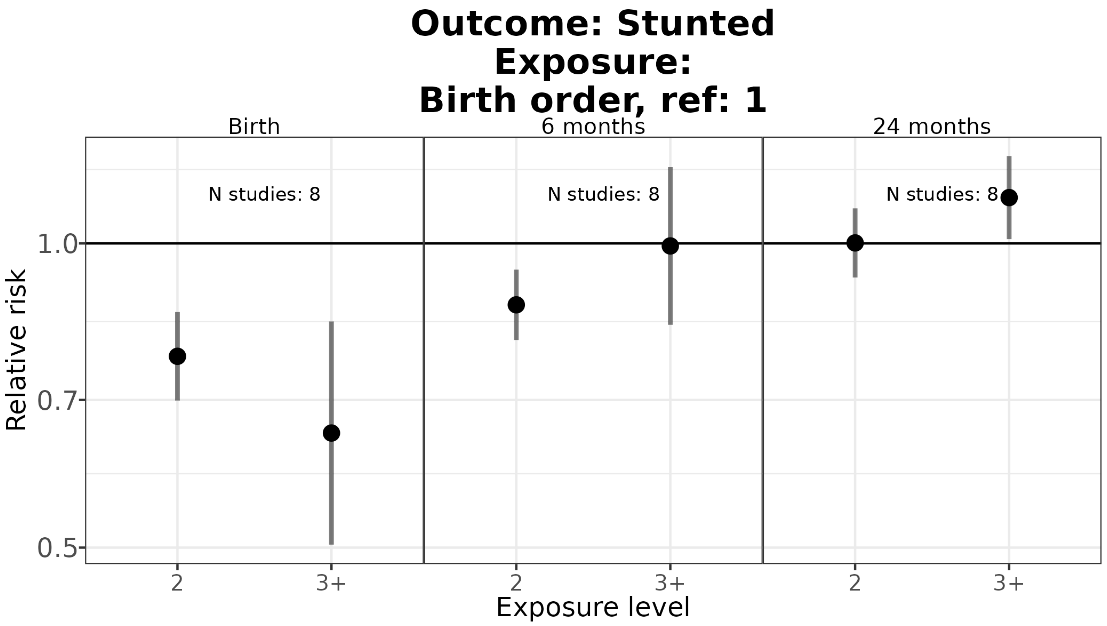
Outcome: Stunted
Exposure:
Birth order, ref: 1
Birth
6 months
24 months
N studies: 8
N studies: 8
N studies: 8
Relative risk
1.0
0.7
0.5
2
3+
2
3+
2
3+
Exposure level

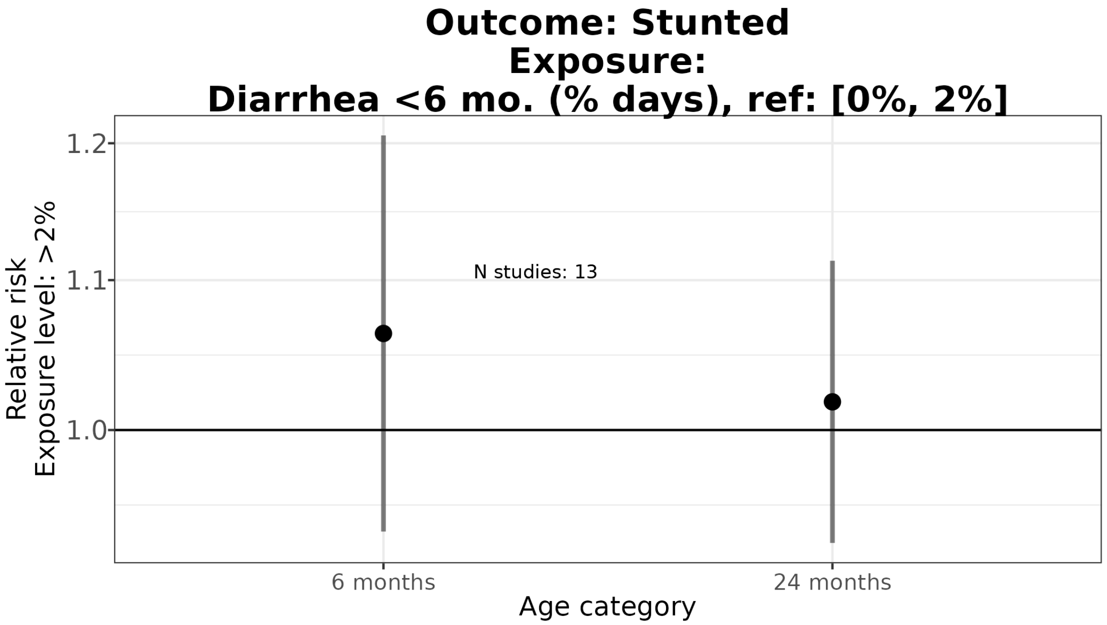
Outcome: Stunted
Exposure:
Diarrhea <6 mo. (% days), ref: [0%, 2%]
N studies: 13
Relative risk
Exposure level: >2%
1.2
1.1
1.0
6 months
24 months
Age category

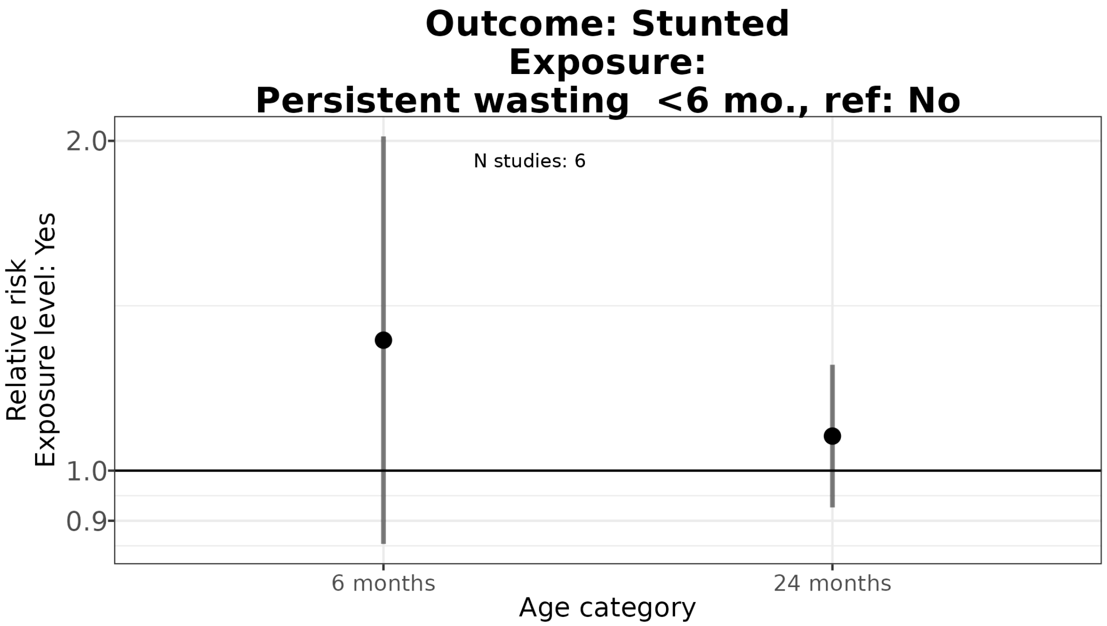
Outcome: Stunted
Exposure:
Persistent wasting <6 mo., ref: No
N studies: 6
Relative risk
Exposure level: Yes
2.0
1.0
0.9
6 months
24 months
Age category

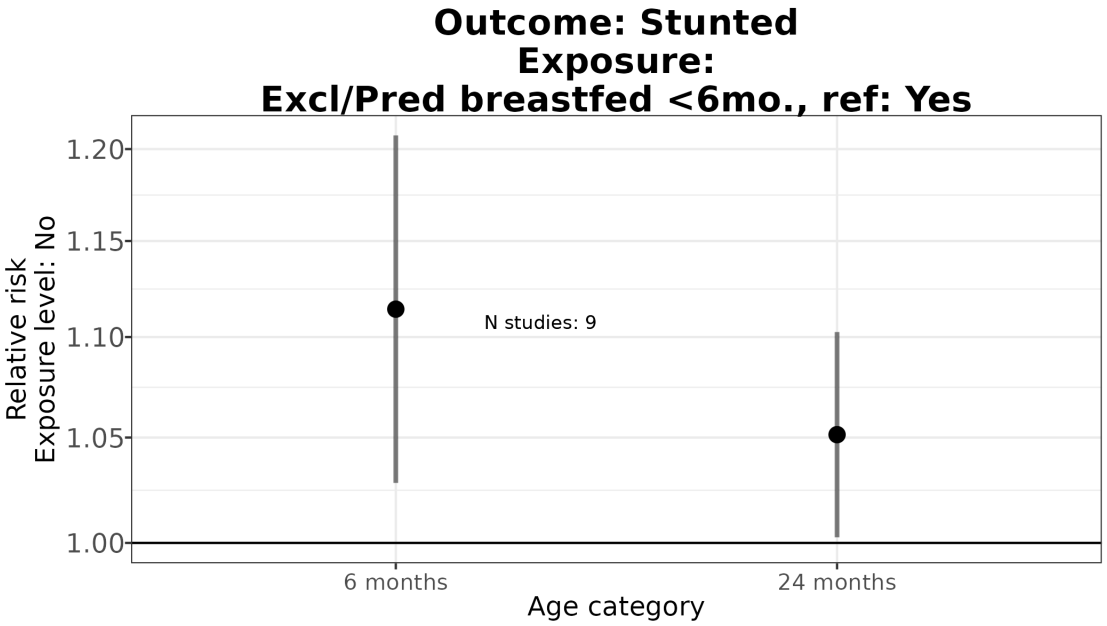
Outcome: Stunted
Exposure:
Excl/Pred breastfed <6mo., ref: Yes
N studies: 9
Relative risk
Exposure level: No
1.20
1.15
1.10
1.05
1.00
6 months
24 months
Age category

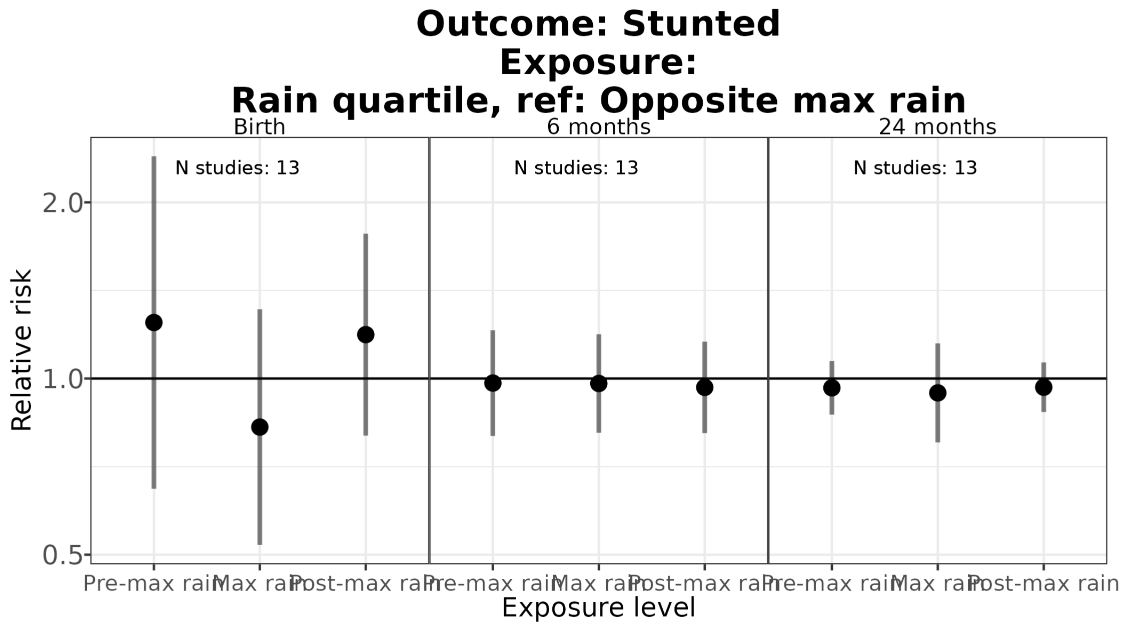
Outcome: Stunted
Exposure:
Rain quartile, ref: Opposite max rain
Birth
6 months
24 months
N studies: 13
N studies: 13
N studies: 13
2.0
1.0
0.5
Relative risk
Pre-max rain
Max rain
Post-max rain
Pre-max rain
Max rain
Post-max rain
Pre-max rain
Max rain
Post-max rain
Exposure level

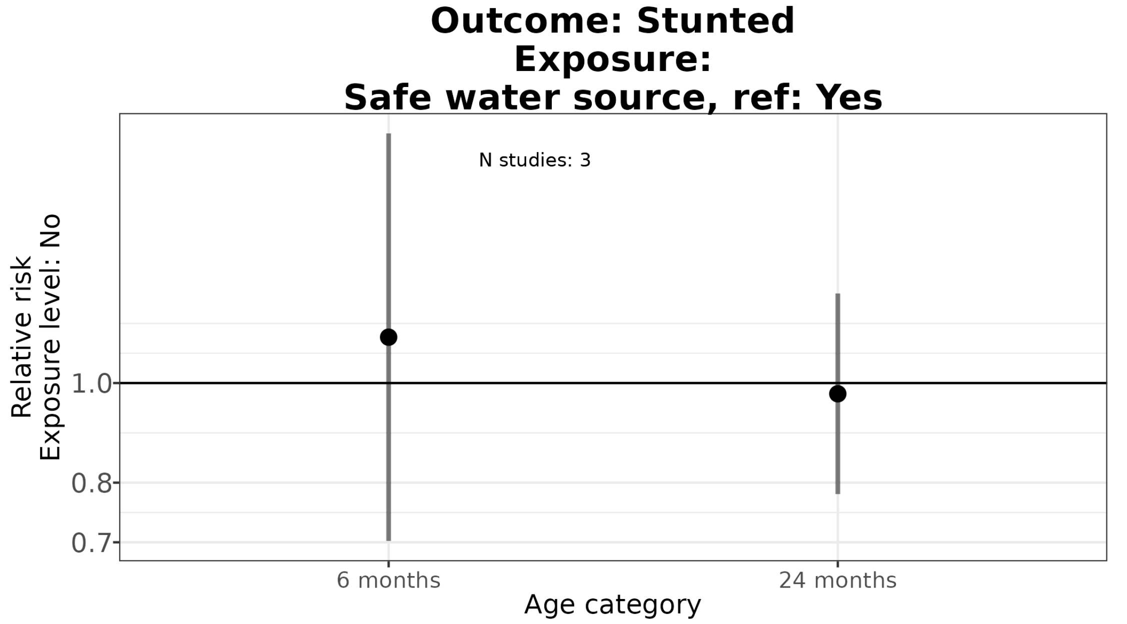
Outcome: Stunted
Exposure:
Safe water source, ref: Yes
N studies: 3
Relative risk
Exposure level: No
1.0
0.8
0.7
6 months
24 months
Age category

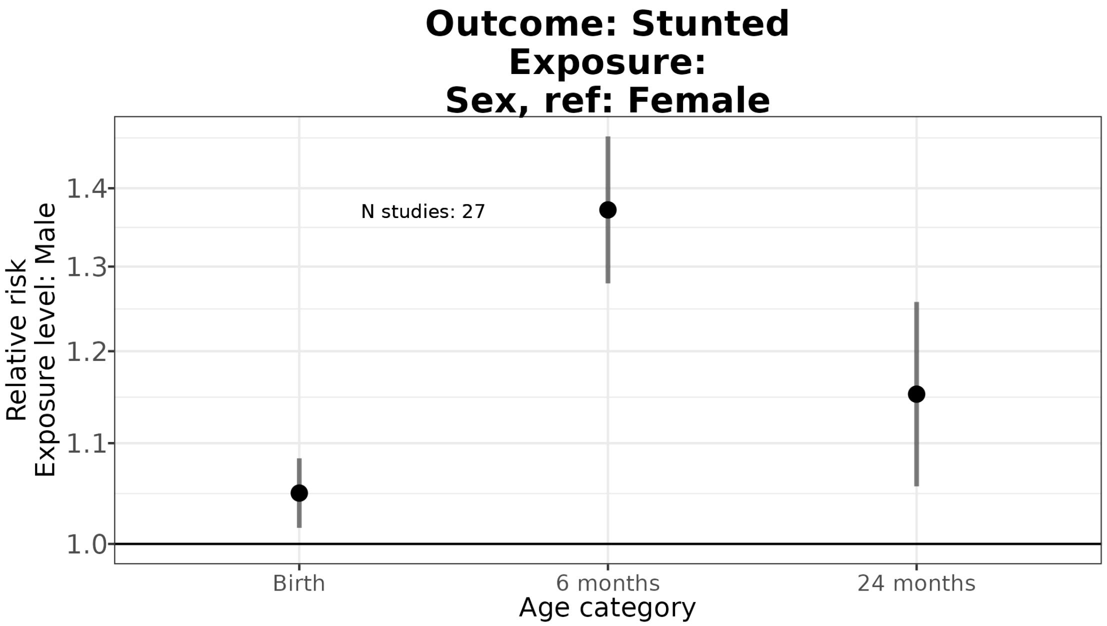
Outcome: Stunted
Exposure:
Sex, ref: Female
N studies: 27
Relative risk
Exposure level: Male
1.4
1.3
1.2
1.1
1.0
Birth
6 months
24 months
Age category

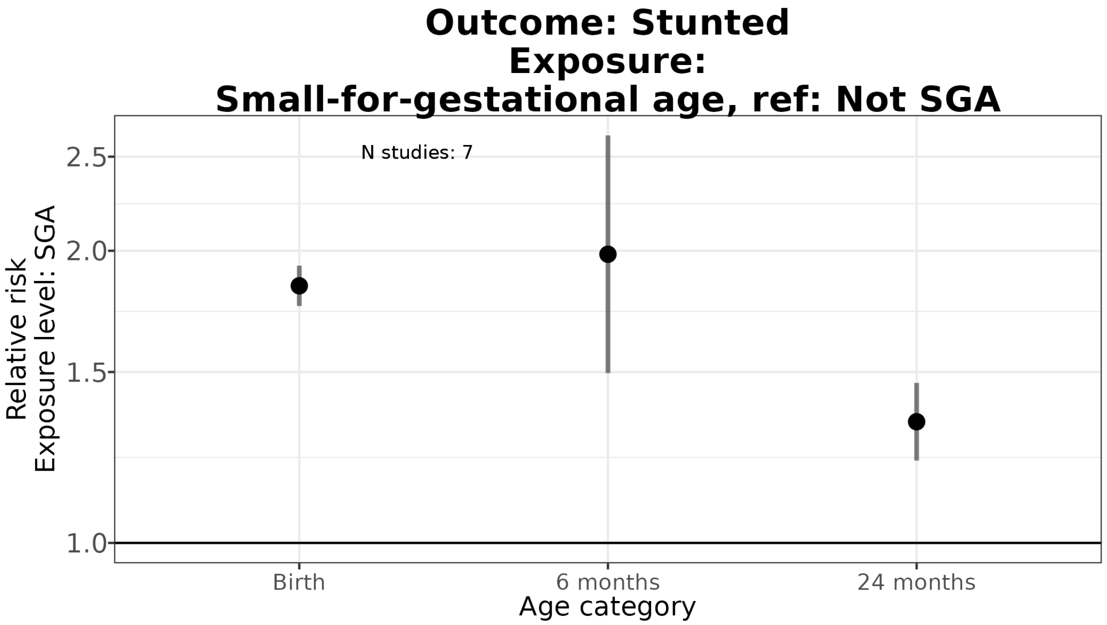
Outcome: Stunted
Exposure:
Small-for-gestational age, ref: Not SGA
N studies: 7
Relative risk
Exposure level: SGA
2.5
2.0
1.5
1.0
Birth
6 months
24 months
Age category

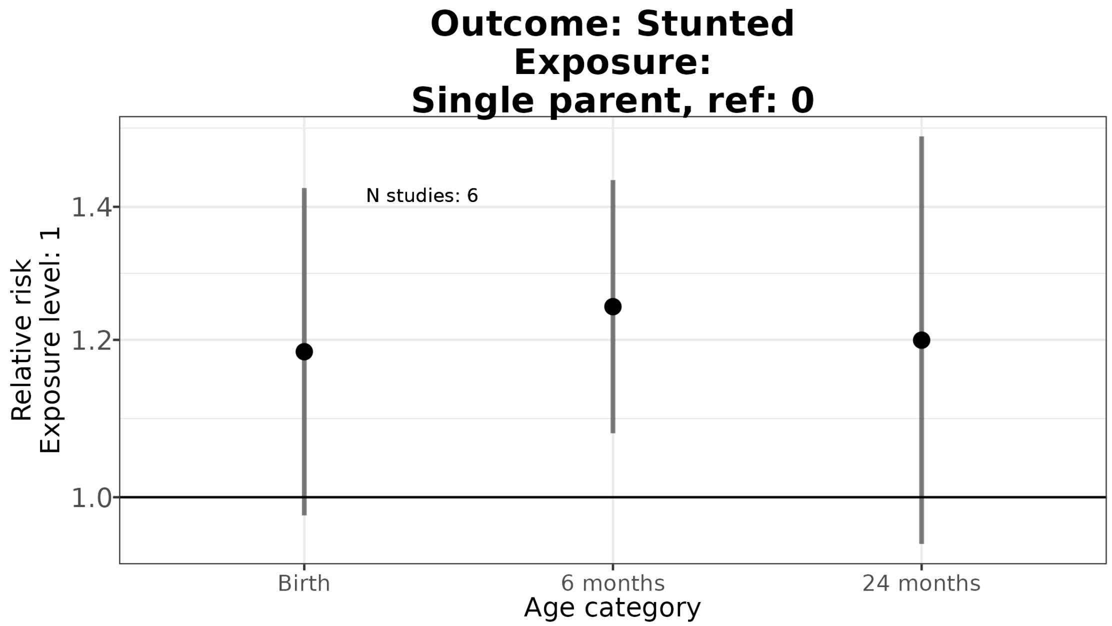
Outcome: Stunted
Exposure:
Single parent, ref: 0
N studies: 6
Relative risk
Exposure level: 1
1.4
1.2
1.0
Birth
6 months
24 months
Age category

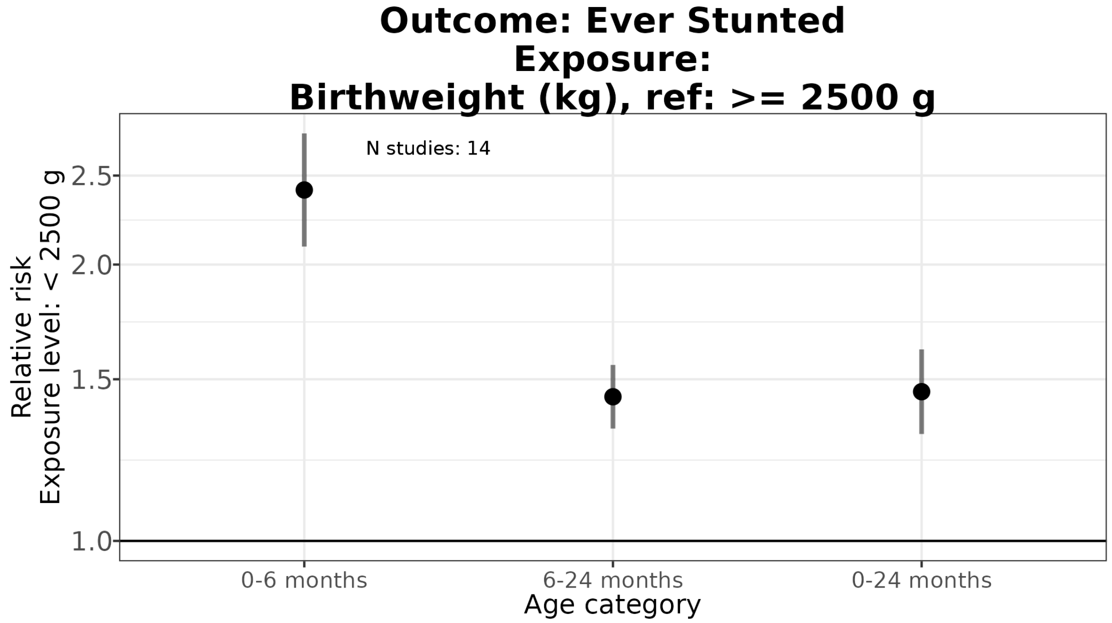
Outcome: Ever Stunted
Exposure:
Birthweight (kg), ref: >= 2500 g
N studies: 14
Relative risk
Exposure level: < 2500 g
2.5
2.0
1.5
1.0
0-6 months
6-24 months
0-24 months
Age category

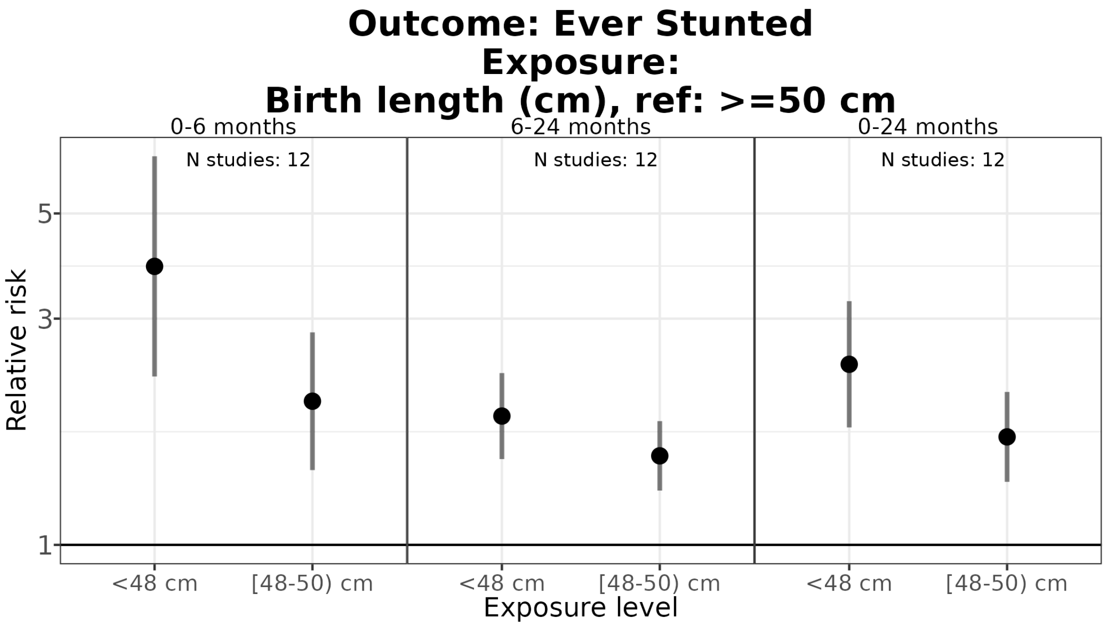
Outcome: Ever Stunted
Exposure:
Birth length (cm), ref: >=50 cm
0-6 months
6-24 months
0-24 months
N studies: 12
N studies: 12
N studies: 12
Relative risk
5
3
1
<48 cm
[48-50) cm
<48 cm
[48-50) cm
<48 cm
[48-50) cm
Exposure level

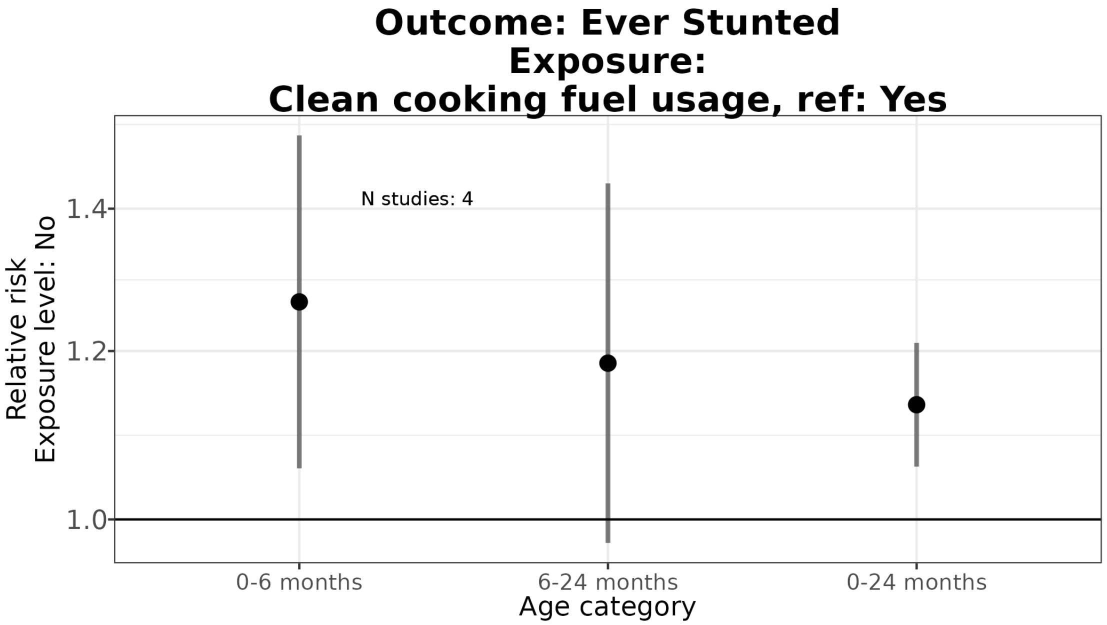
Outcome: Ever Stunted
Exposure:
Clean cooking fuel usage, ref: Yes
N studies: 4
Relative risk
Exposure level: No
1.4
1.2
1.0
0-6 months
6-24 months
0-24 months
Age category

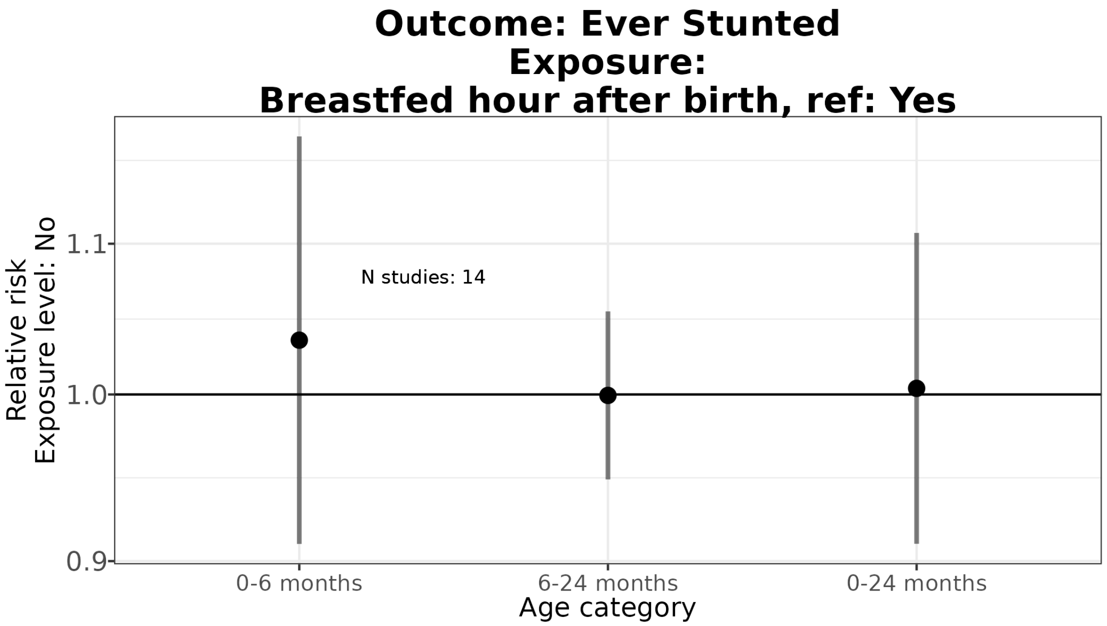
Outcome: Ever Stunted
Exposure:
Breastfed hour after birth, ref: Yes
N studies: 14
Relative risk
Exposure level: No
1.1
1.0
0.9
0-6 months
6-24 months
0-24 months
Age category

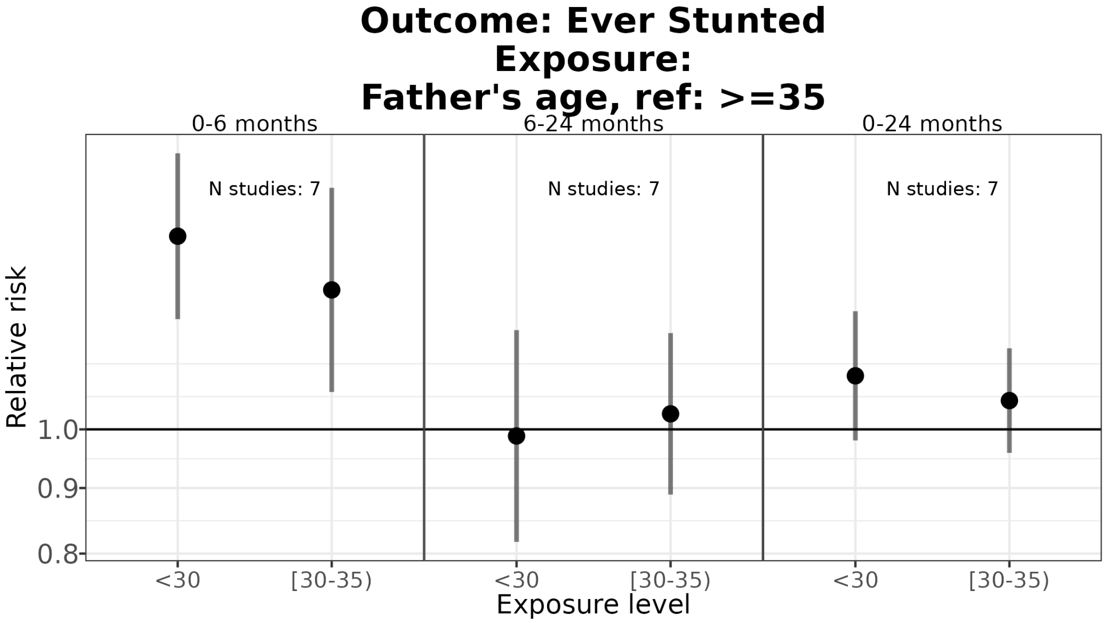
Outcome: Ever Stunted
Exposure:
Father's age, ref: >=35
0-6 months
6-24 months
0-24 months
N studies: 7
N studies: 7
N studies: 7
Relative risk
1.0
0.9
0.8
<30
[30-35)
<30
[30-35)
<30
[30-35)
Exposure level

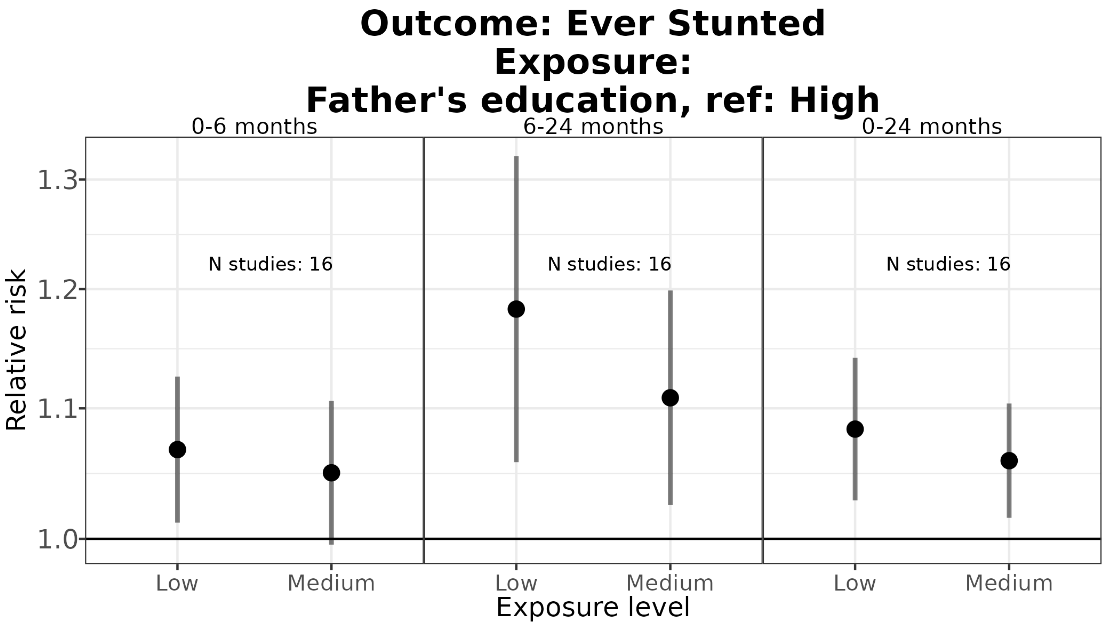

Outcome: Ever Stunted
Exposure:
Father's education, ref: High
0-6 months
6-24 months
0-24 months
Relative risk
1.3
1.2
1.1
1.0
N studies: 16
N studies: 16
N studies: 16
Low
Medium
Low
Medium
Low
Medium
Exposure level


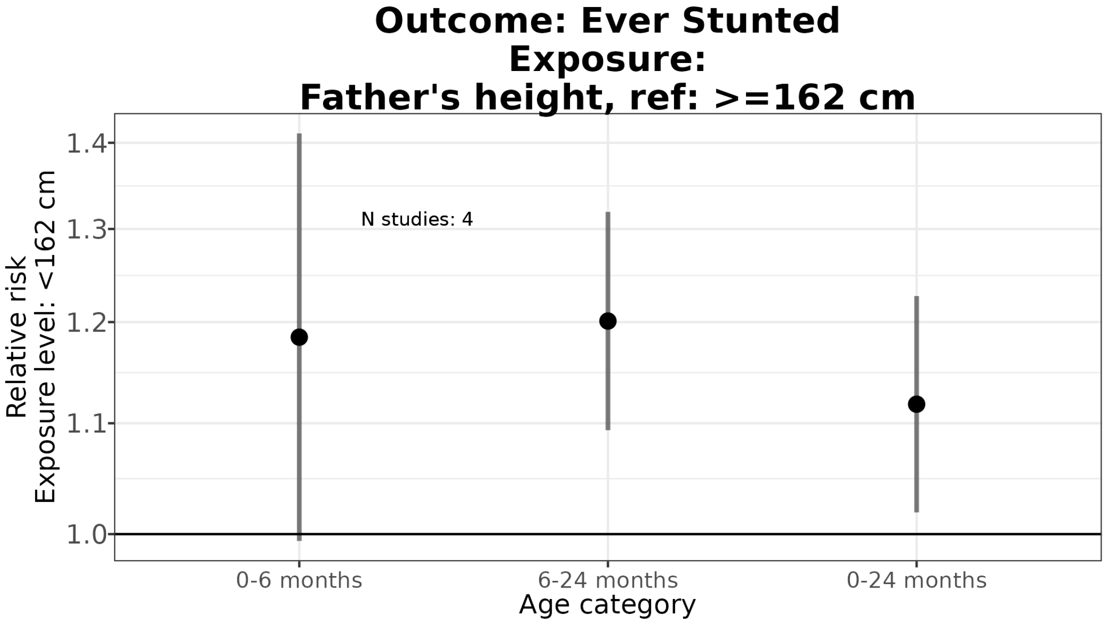

Outcome: Ever Stunted
Exposure:
Father's height, ref: >=162 cm
Relative risk
Exposure level: <162 cm
1.4
1.3
1.2
1.1
1.0
N studies: 4
0-6 months
6-24 months
0-24 months
Age category

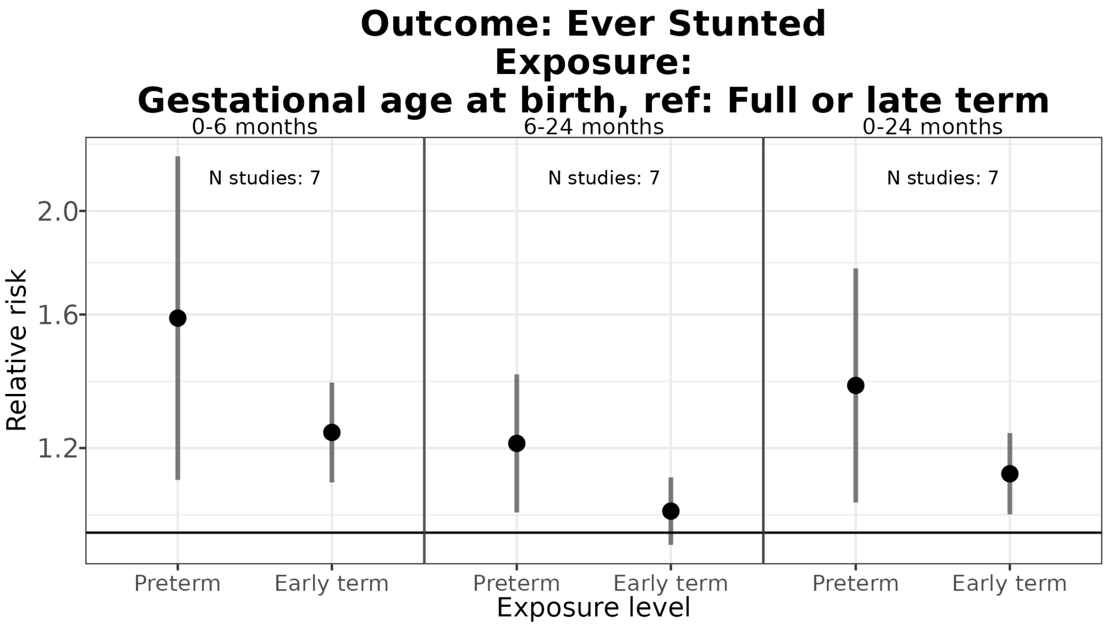
Outcome: Ever Stunted
Exposure:
Gestational age at birth, ref: Full or late term
0-6 months
6-24 months
0-24 months
N studies: 7
N studies: 7
N studies: 7
Relative risk
2.0
1.6
1.2
Preterm
Early term
Preterm
Early term
Preterm
Early term
Exposure level

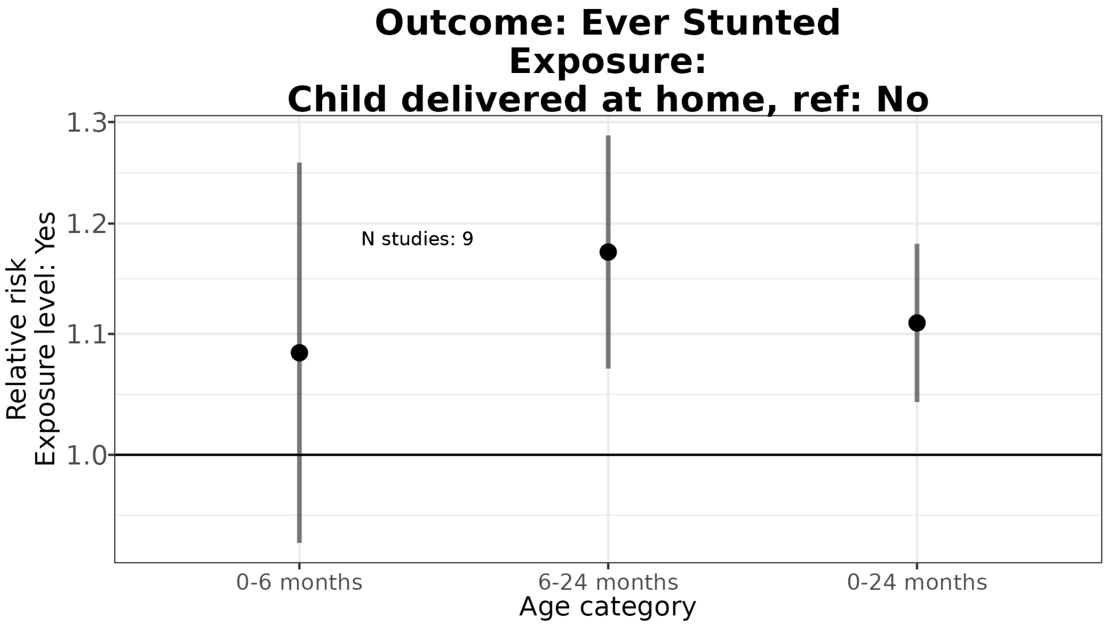
Outcome: Ever Stunted
Exposure:
Child delivered at home, ref: No
Relative risk
Exposure level: Yes
1.3
1.2
1.1
1.0
N studies: 9
0-6 months
6-24 months
0-24 months
Age category

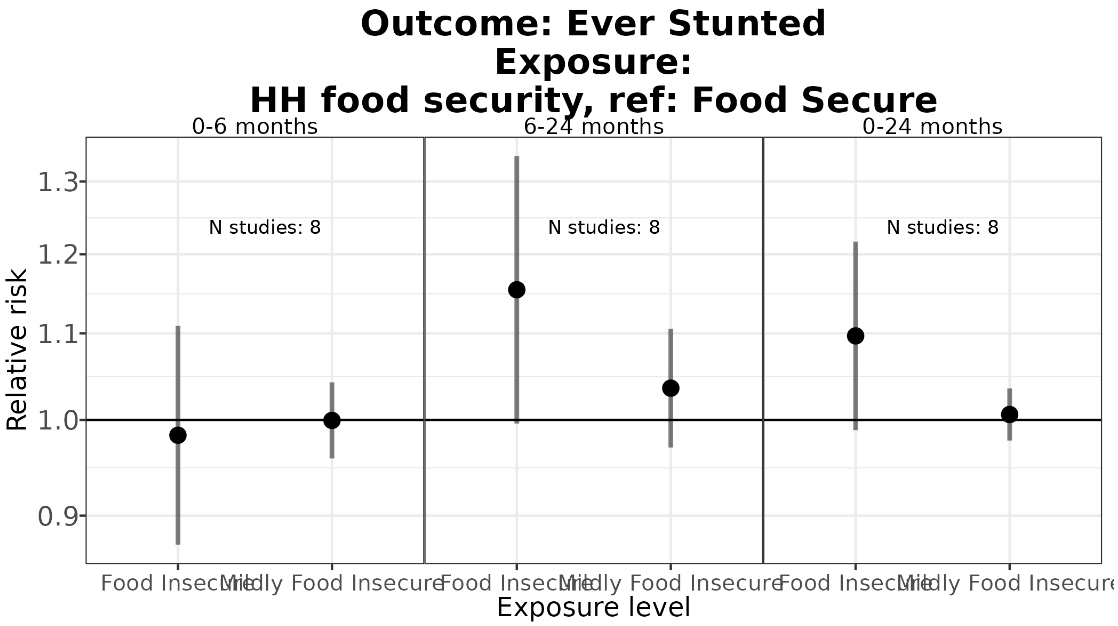
Outcome: Ever Stunted
Exposure:
HH food security, ref: Food Secure
0-6 months
6-24 months
0-24 months
N studies: 8
N studies: 8
N studies: 8
Relative risk
1.3
1.2
1.1
1.0
0.9
Food Insecure
Mildly Food Insecure
Exposure level

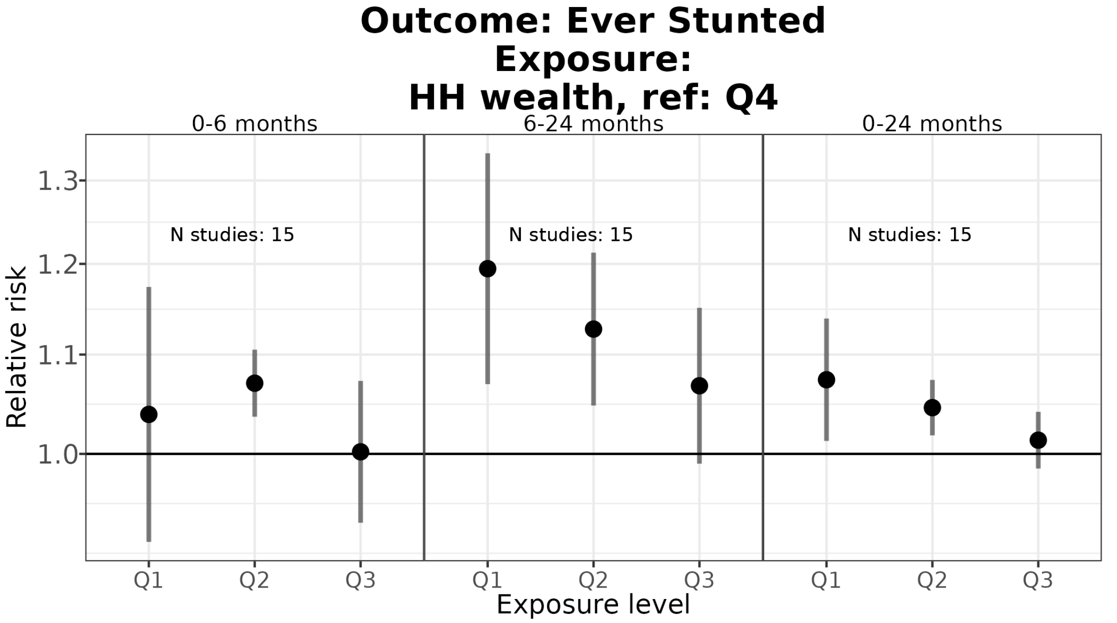
Outcome: Ever Stunted
Exposure:
HH wealth, ref: Q4
0-6 months
6-24 months
0-24 months
N studies: 15
N studies: 15
N studies: 15
Relative risk
1.3
1.2
1.1
1.0
Q1
Q2
Q3
Exposure level

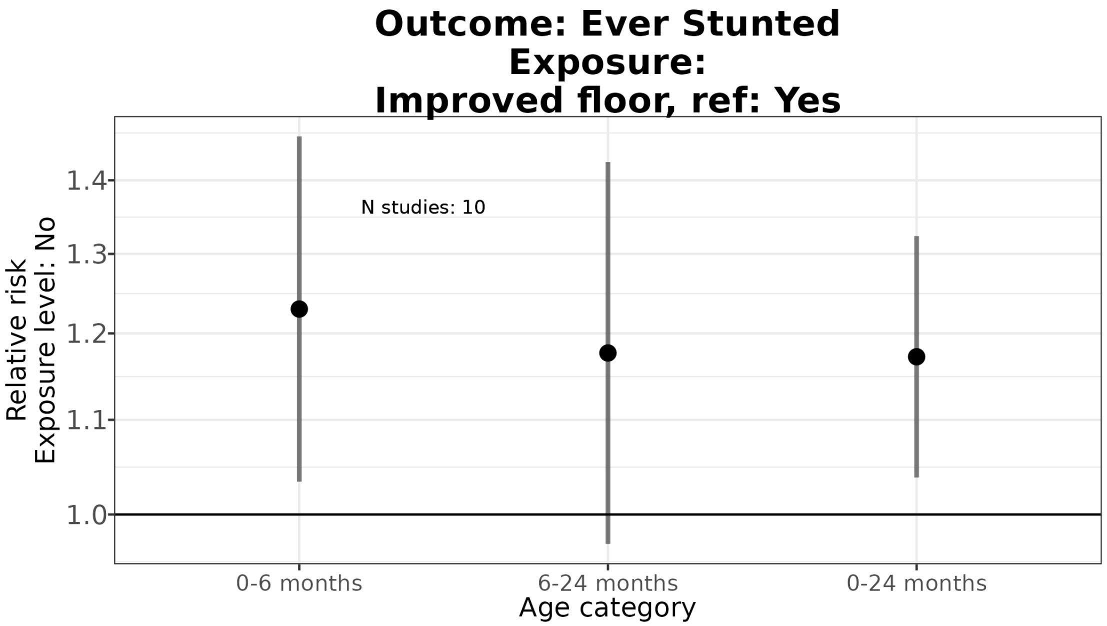

Outcome: Ever Stunted
Exposure:
Improved floor, ref: Yes
Relative risk
Exposure level: No
1.4
1.3
1.2
1.1
1.0
N studies: 10
0-6 months
6-24 months
0-24 months
Age category


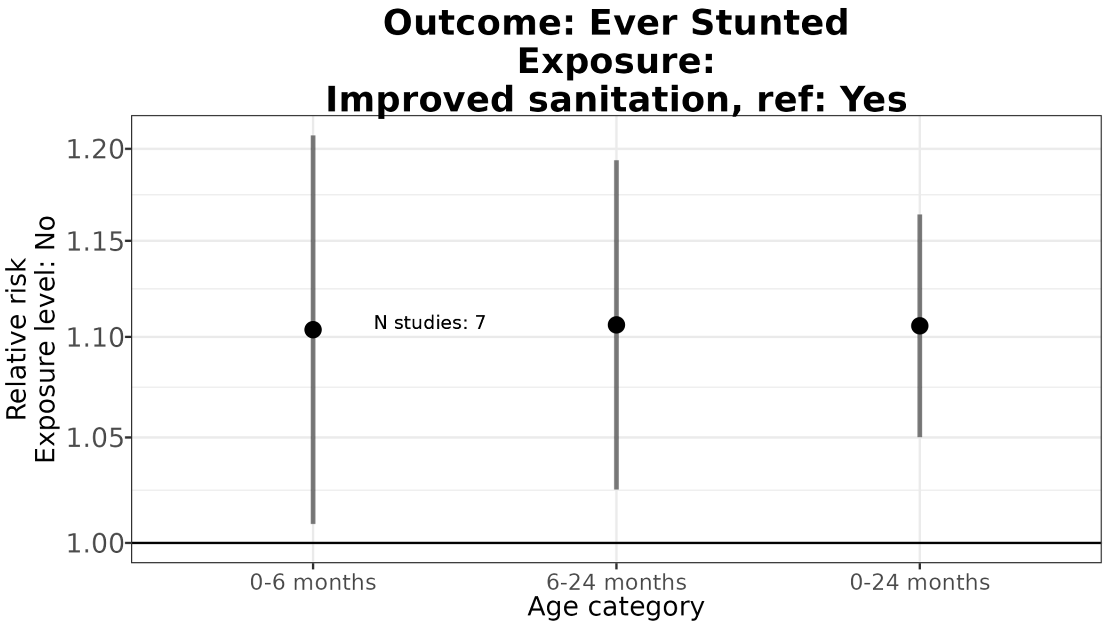

Outcome: Ever Stunted
Exposure:
Improved sanitation, ref: Yes
Relative risk
Exposure level: No
1.20
1.15
1.10
1.05
1.00
N studies: 7
0-6 months
6-24 months
0-24 months
Age category

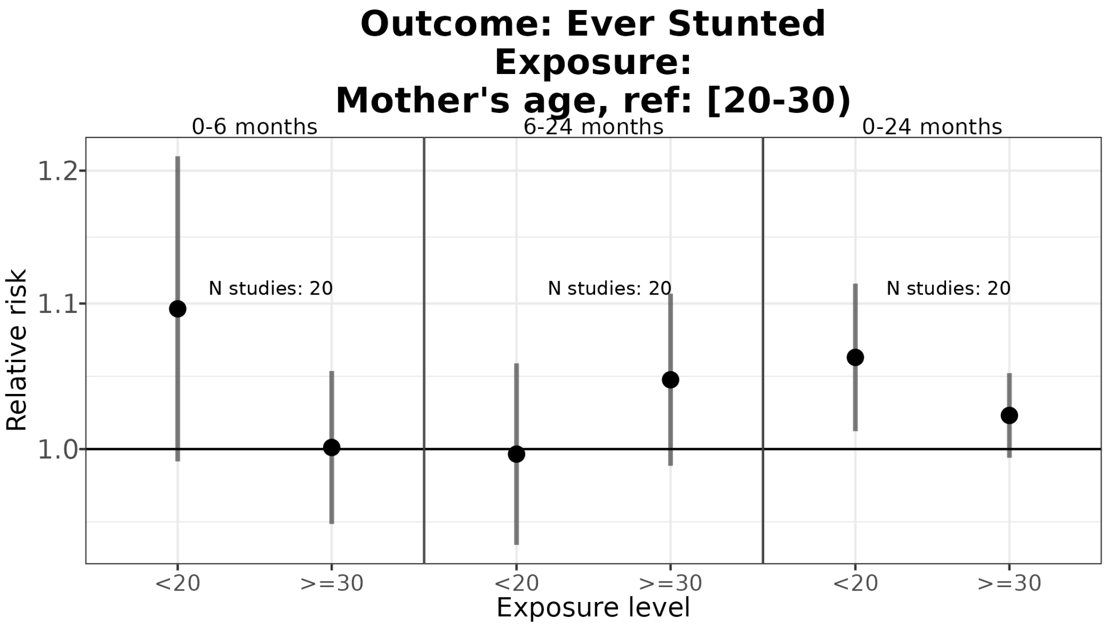
Outcome: Ever Stunted
Exposure:
Mother's age, ref: [20-30)
0-6 months
6-24 months
0-24 months
N studies: 20
N studies: 20
N studies: 20
Relative risk
1.2
1.1
1.0
<20
>=30
<20
>=30
<20
>=30
Exposure level

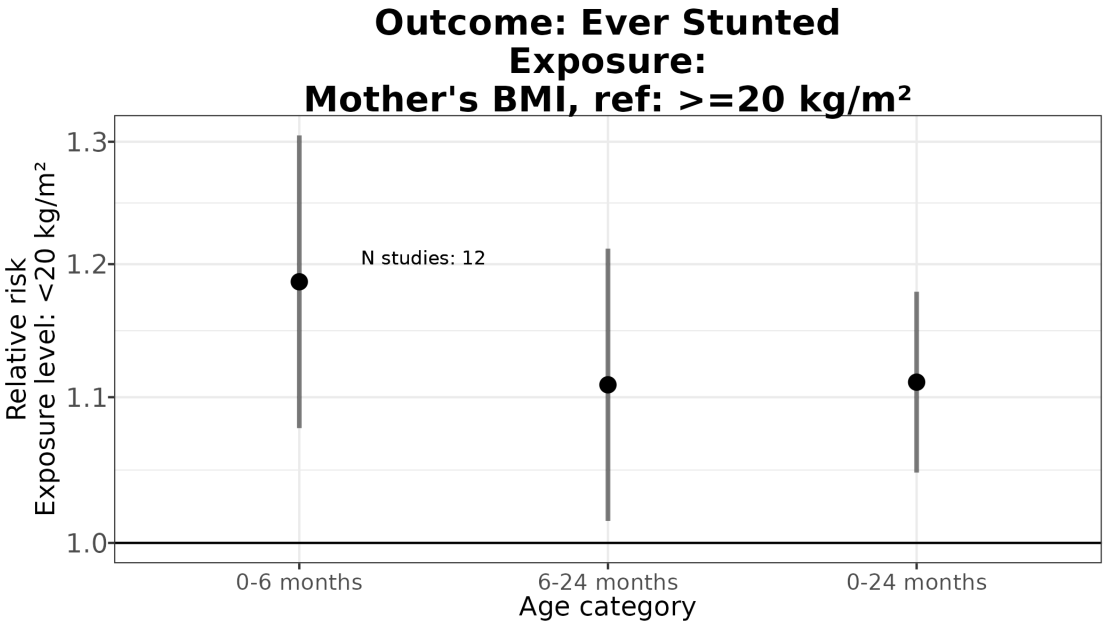
Outcome: Ever Stunted
Exposure:
Mother's BMI, ref: >=20 kg/m²
N studies: 12
Relative risk
Exposure level: <20 kg/m²
1.3
1.2
1.1
1.0
0-6 months
6-24 months
0-24 months
Age category

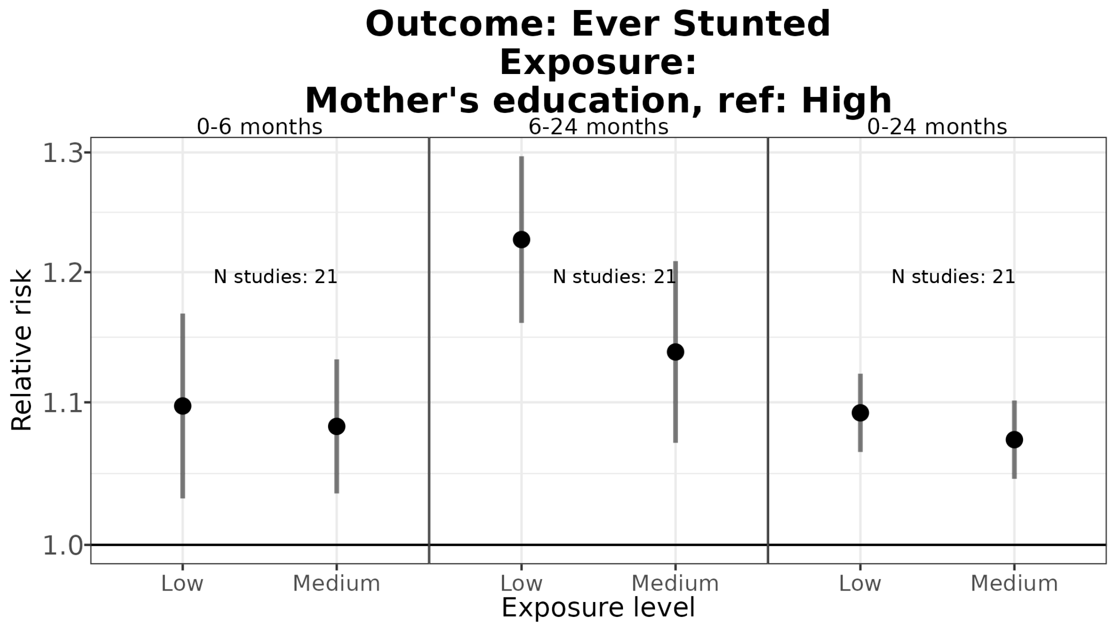
Outcome: Ever Stunted
Exposure:
Mother's education, ref: High
0-6 months
6-24 months
0-24 months
N studies: 21
N studies: 21
N studies: 21
Relative risk
1.3
1.2
1.1
1.0
Low
Medium
Low
Medium
Low
Medium
Exposure level

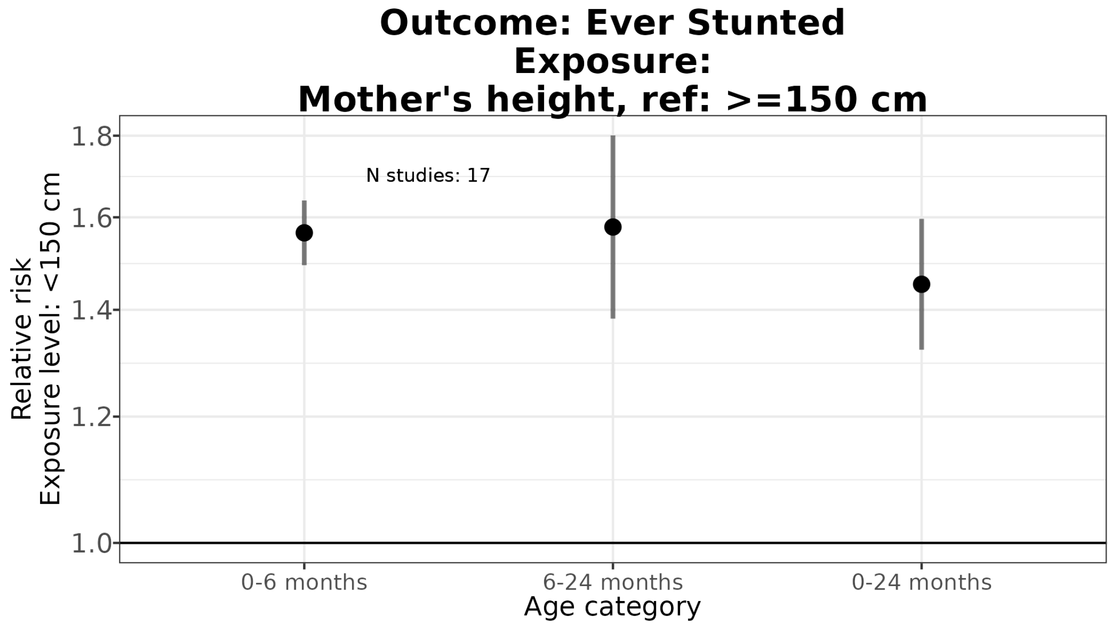
Outcome: Ever Stunted
Exposure:
Mother's height, ref: >=150 cm
N studies: 17
Relative risk
Exposure level: <150 cm
1.8
1.6
1.4
1.2
1.0
0-6 months
6-24 months
0-24 months
Age category

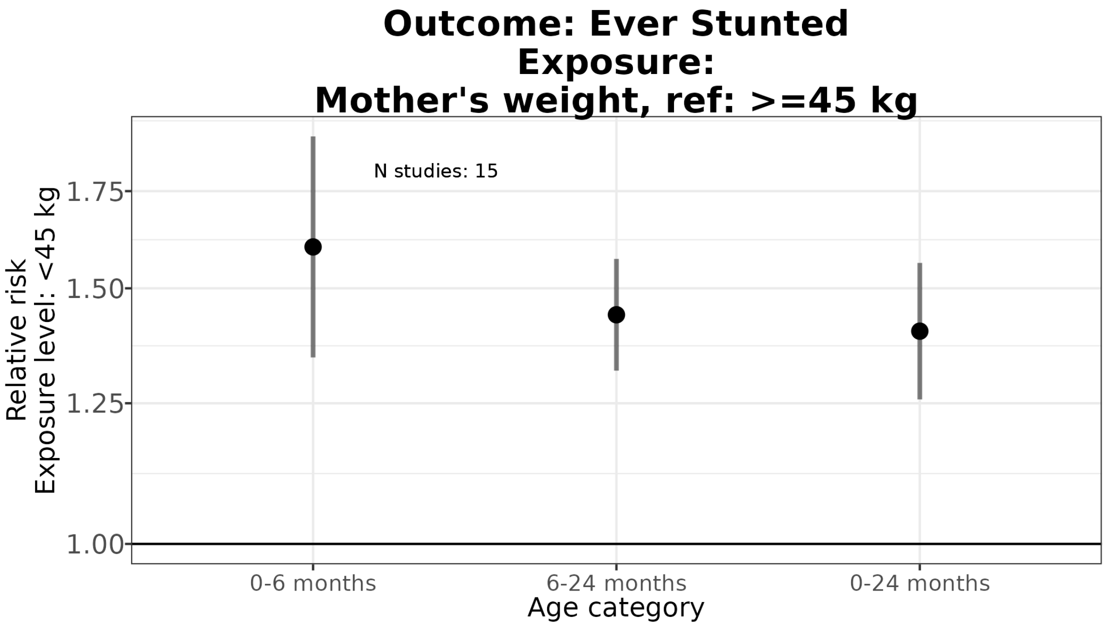
Outcome: Ever Stunted
Exposure:
Mother's weight, ref: >=45 kg
N studies: 15
Relative risk
Exposure level: <45 kg
1.75
1.50
1.25
1.00
0-6 months
6-24 months
0-24 months
Age category

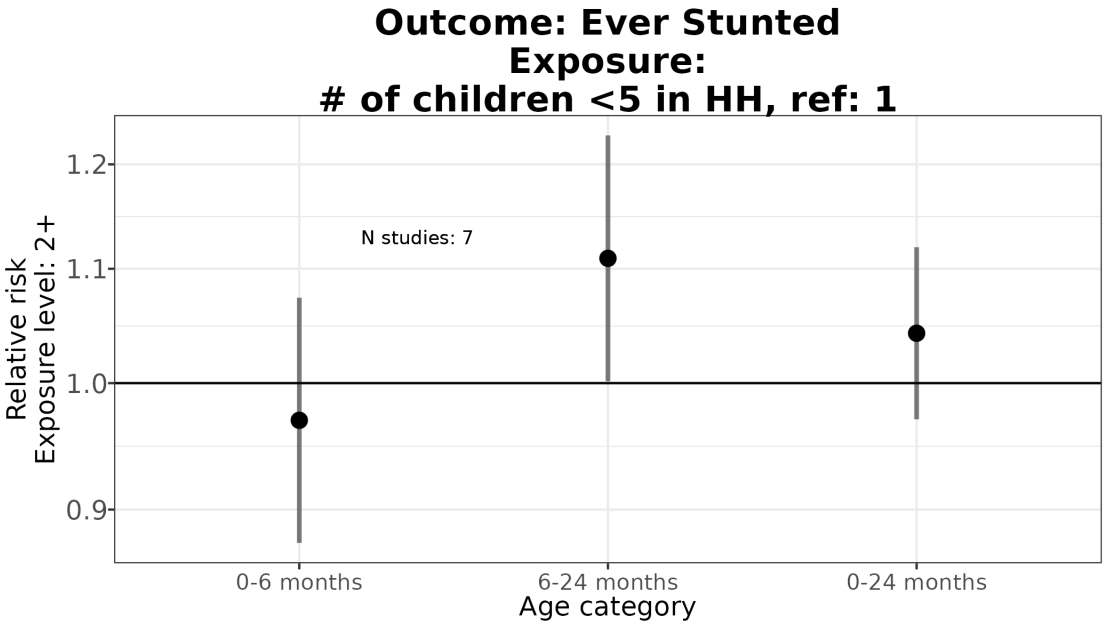
Outcome: Ever Stunted
Exposure:
# of children <5 in HH, ref: 1
N studies: 7
Relative risk
Exposure level: 2+
1.2
1.1
1.0
0.9
0-6 months
6-24 months
0-24 months
Age category

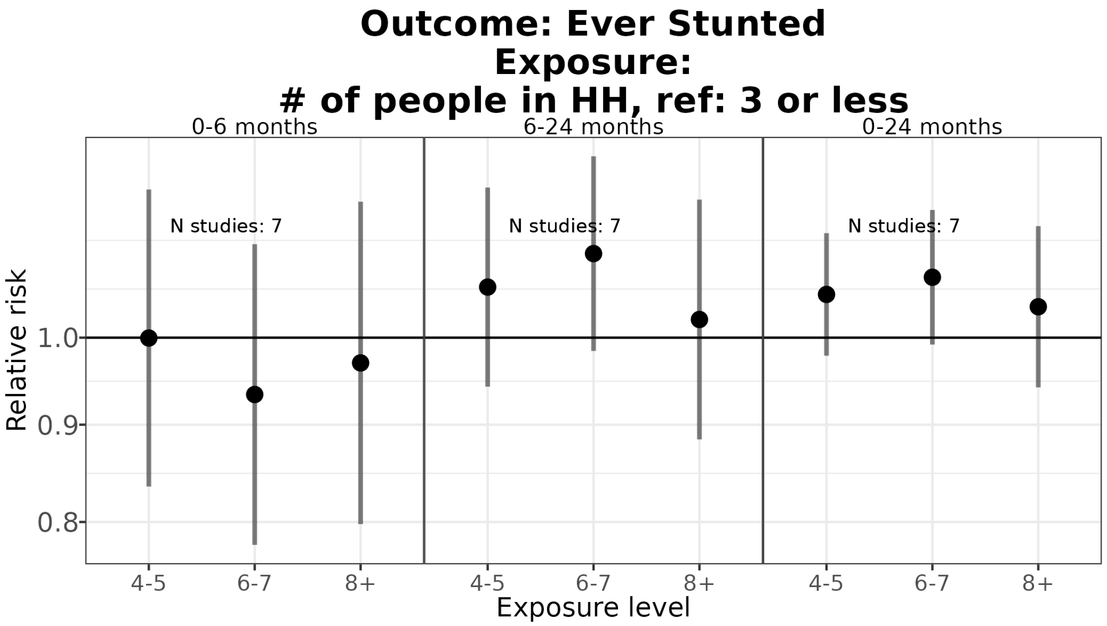
Outcome: Ever Stunted
Exposure:
# of people in HH, ref: 3 or less
0-6 months
6-24 months
0-24 months
N studies: 7
N studies: 7
N studies: 7
Relative risk
1.0
0.9
0.8
4-5
6-7
8+
4-5
6-7
8+
4-5
6-7
8+
Exposure level

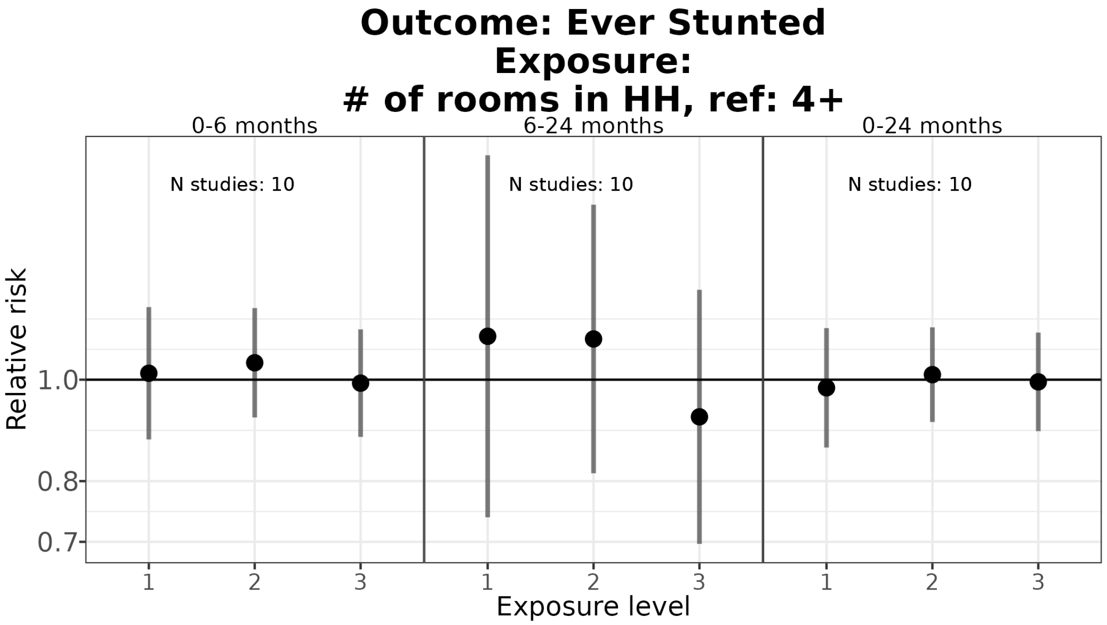
Outcome: Ever Stunted
Exposure:
# of rooms in HH, ref: 4+
0-6 months
6-24 months
0-24 months
N studies: 10
N studies: 10
N studies: 10
Relative risk
1.0
0.8
0.7
1
2
3
1
2
3
1
2
3
Exposure level

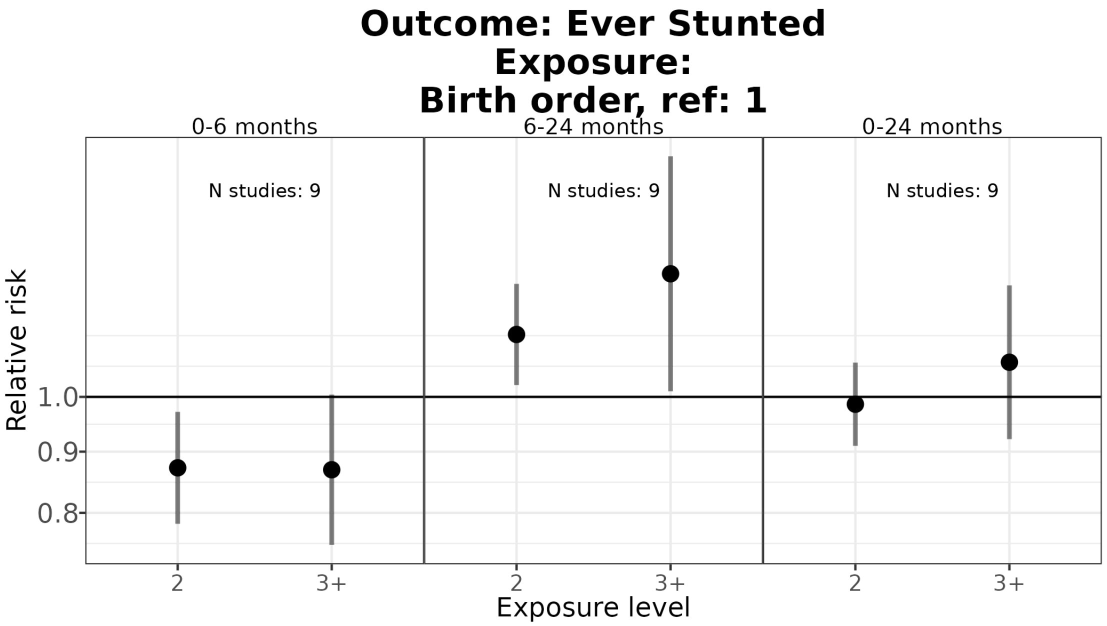
Outcome: Ever Stunted
Exposure:
Birth order, ref: 1
0-6 months
6-24 months
0-24 months
N studies: 9
N studies: 9
N studies: 9
Relative risk
1.0
0.9
0.8
2
3+
2
3+
2
3+
Exposure level

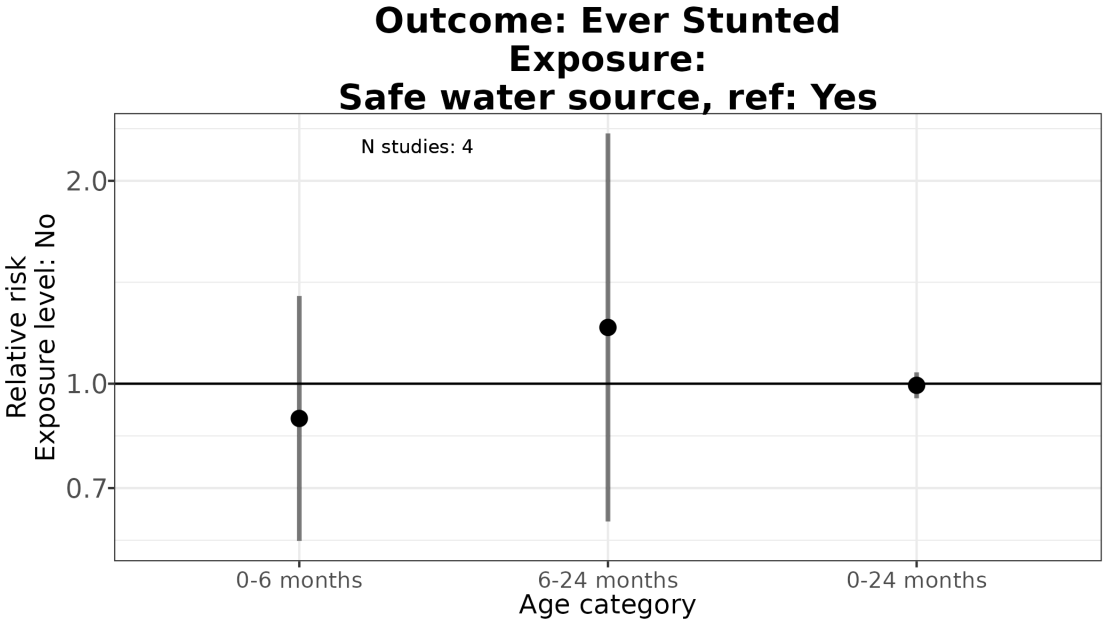
Outcome: Ever Stunted
Exposure:
Safe water source, ref: Yes
N studies: 4
2.0
1.0
0.7
Relative risk
Exposure level: No
0-6 months
6-24 months
0-24 months
Age category

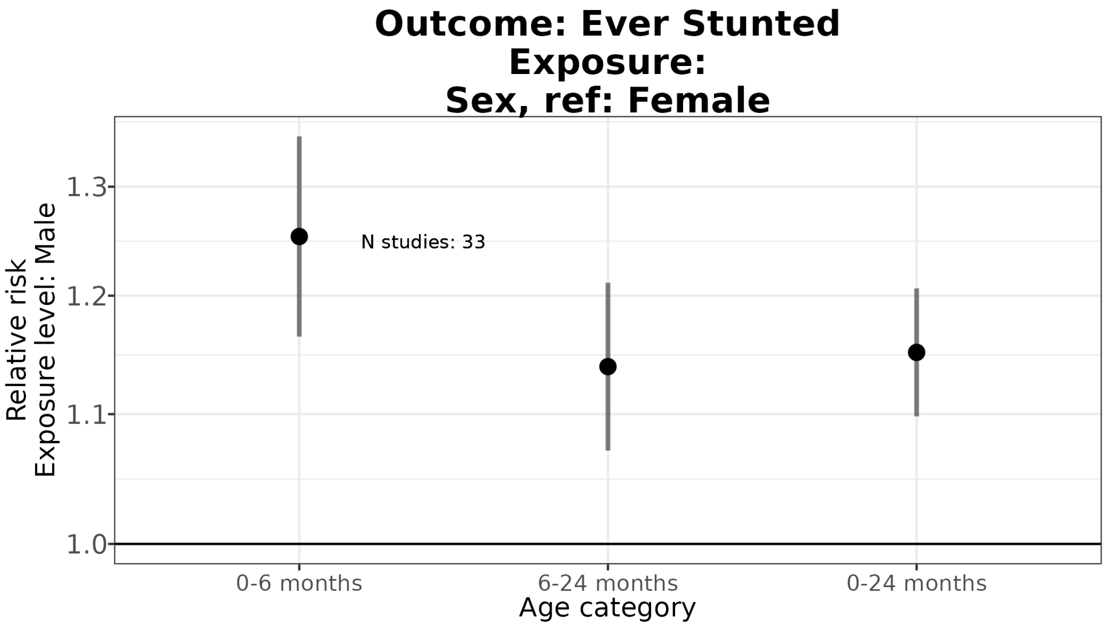
Outcome: Ever Stunted
Exposure:
Sex, ref: Female
Relative risk
Exposure level: Male
1.0
1.1
1.2
1.3
N studies: 33
0-6 months
6-24 months
0-24 months
Age category

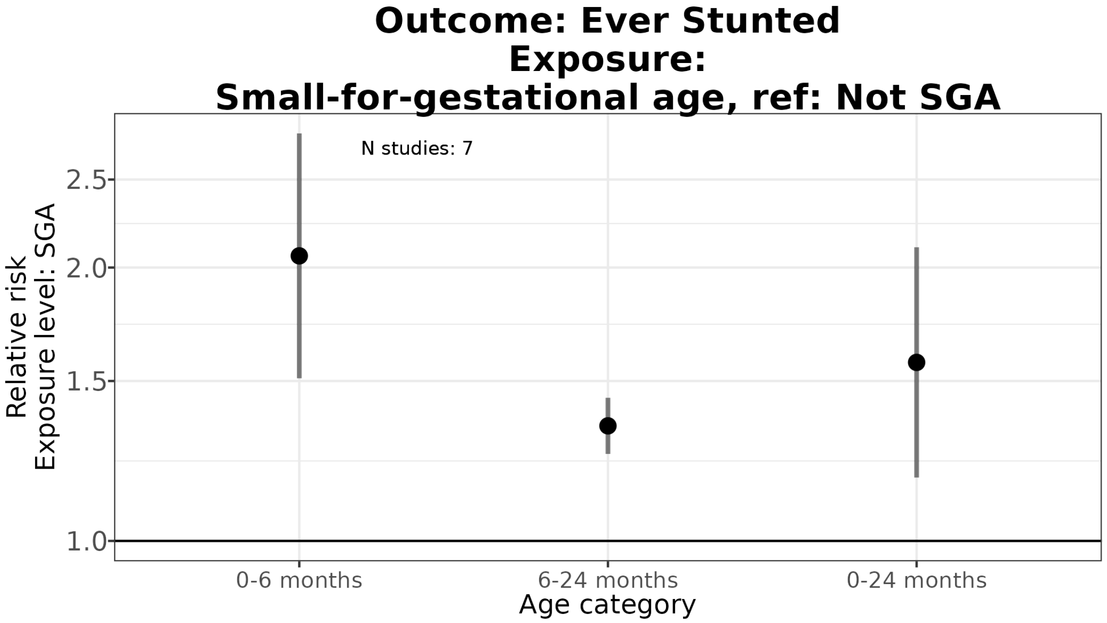
Outcome: Ever Stunted
Exposure:
Small-for-gestational age, ref: Not SGA
Relative risk
Exposure level: SGA
1.0
1.5
2.0
2.5
N studies: 7
0-6 months
6-24 months
0-24 months
Age category

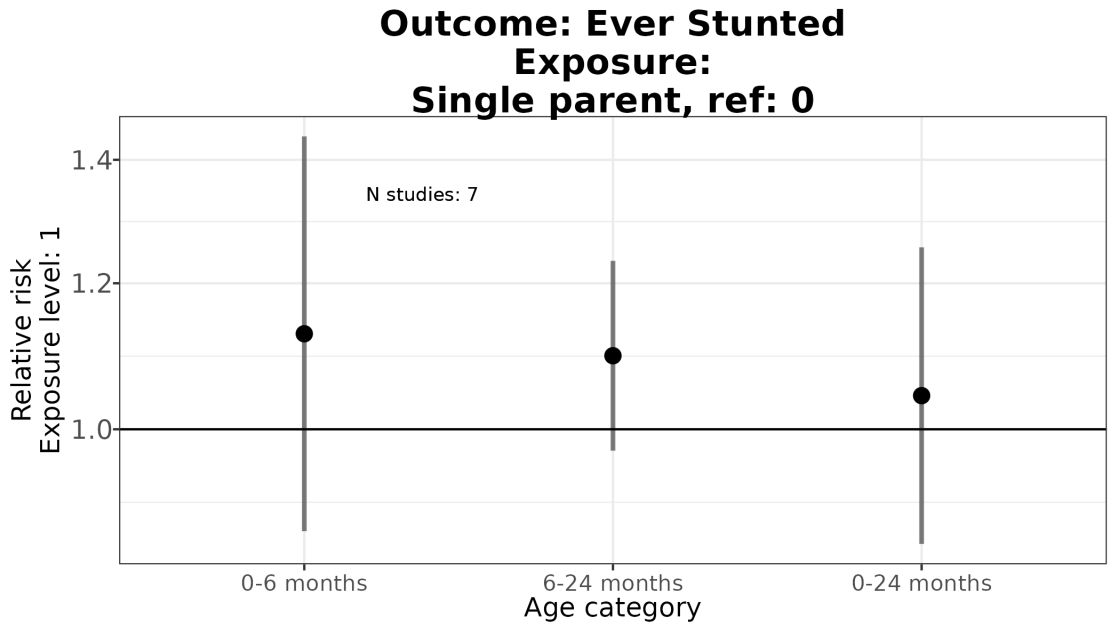
Outcome: Ever Stunted
Exposure:
Single parent, ref: 0
N studies: 7
Relative risk
Exposure level: 1
1.4
1.2
1.0
0-6 months
6-24 months
0-24 months
Age category

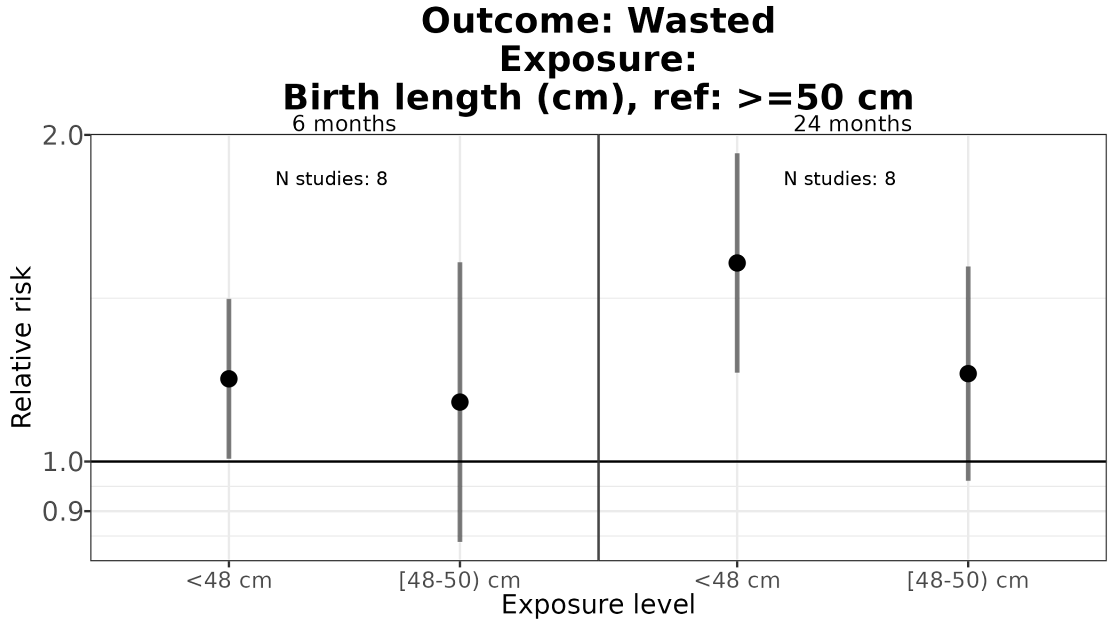
Outcome: Wasted
Exposure:
Birth length (cm), ref: >=50 cm
6 months
24 months
N studies: 8
N studies: 8
Relative risk
2.0
1.0
0.9
<48 cm
[48-50) cm
<48 cm
[48-50) cm
Exposure level

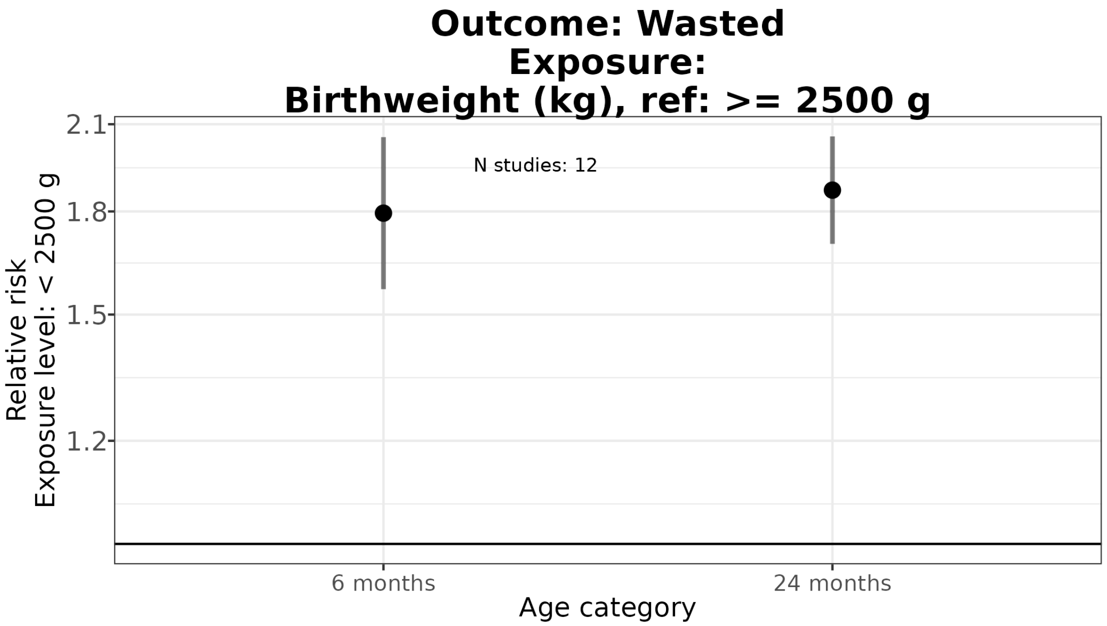
Outcome: Wasted
Exposure:
Birthweight (kg), ref: >= 2500 g
Relative risk
Exposure level: < 2500 g
2.1
1.8
1.5
1.2
N studies: 12
6 months
24 months
Age category

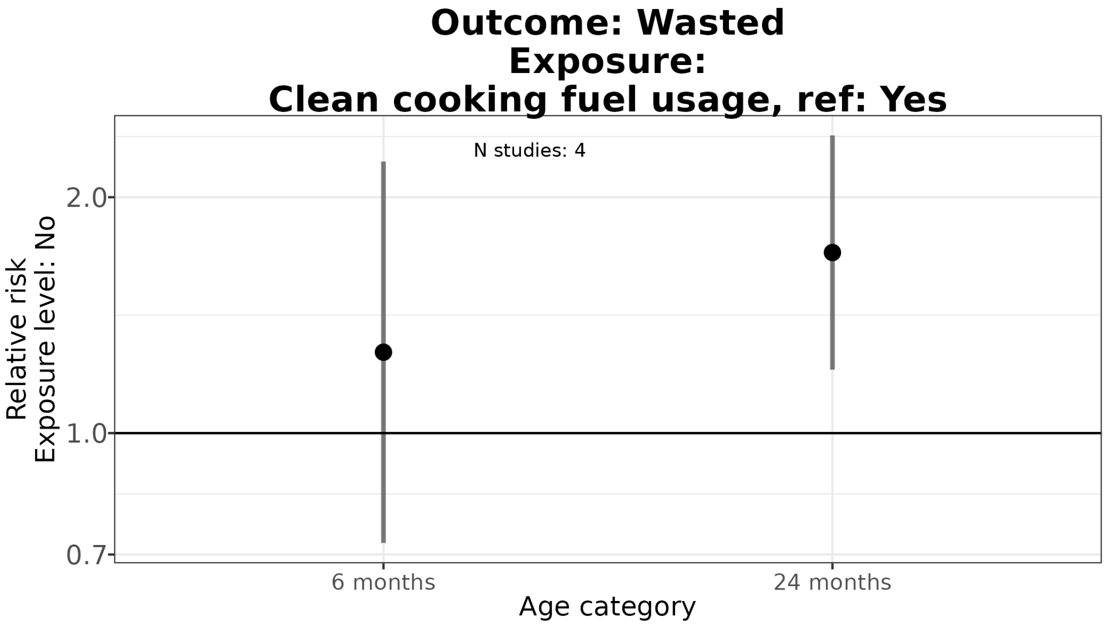
Outcome: Wasted
Exposure:
Clean cooking fuel usage, ref: Yes
Relative risk
Exposure level: No
2.0
1.0
0.7
N studies: 4
6 months
24 months
Age category

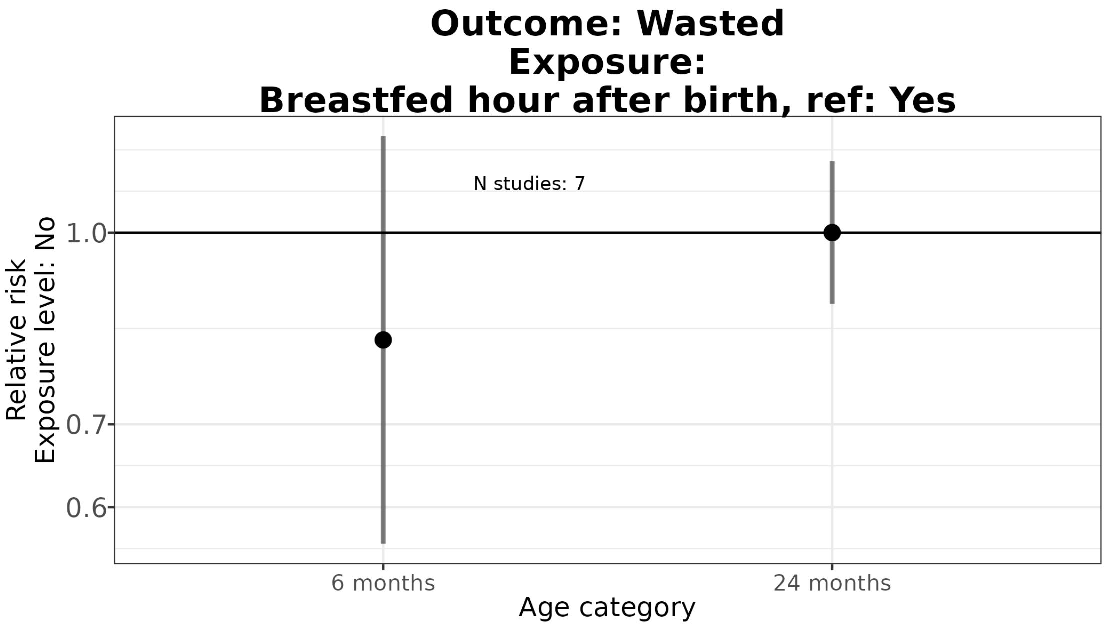
Outcome: Wasted
Exposure:
Breastfed hour after birth, ref: Yes
N studies: 7
Relative risk
Exposure level: No
1.0
0.7
0.6
6 months
24 months
Age category

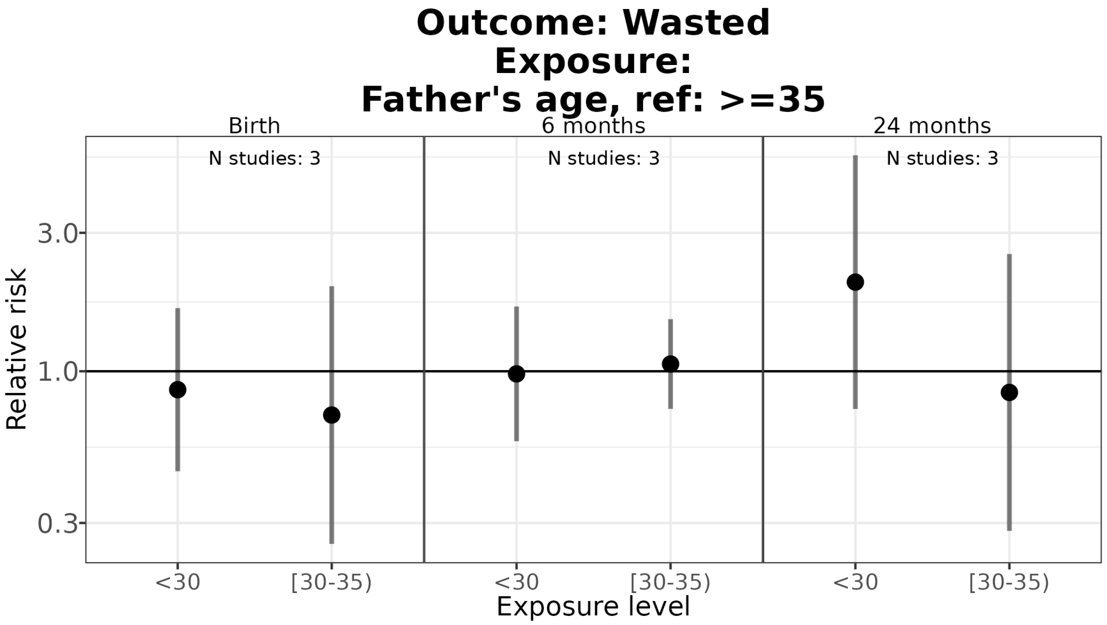
Outcome: Wasted
Exposure:
Father's age, ref: >=35
Birth
6 months
24 months
N studies: 3
N studies: 3
N studies: 3
Relative risk
3.0
1.0
0.3
<30
[30-35)
<30
[30-35)
<30
[30-35)
Exposure level

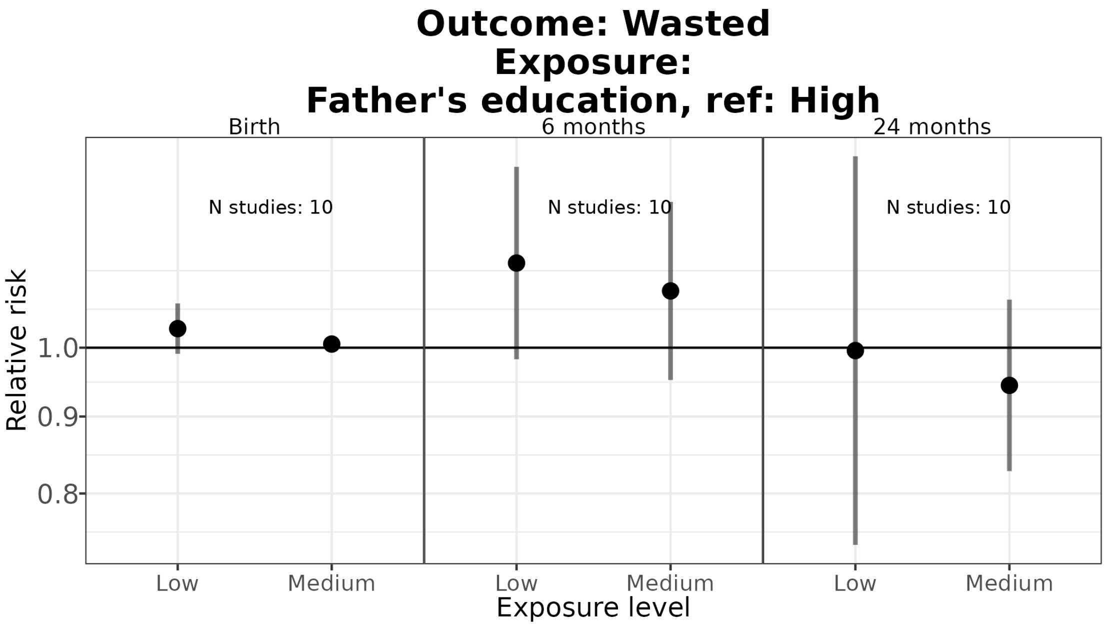
Outcome: Wasted
Exposure:
Father's education, ref: High
Birth
6 months
24 months
N studies: 10
N studies: 10
N studies: 10
Relative risk
1.0
0.9
0.8
Low
Medium
Low
Medium
Low
Medium
Exposure level

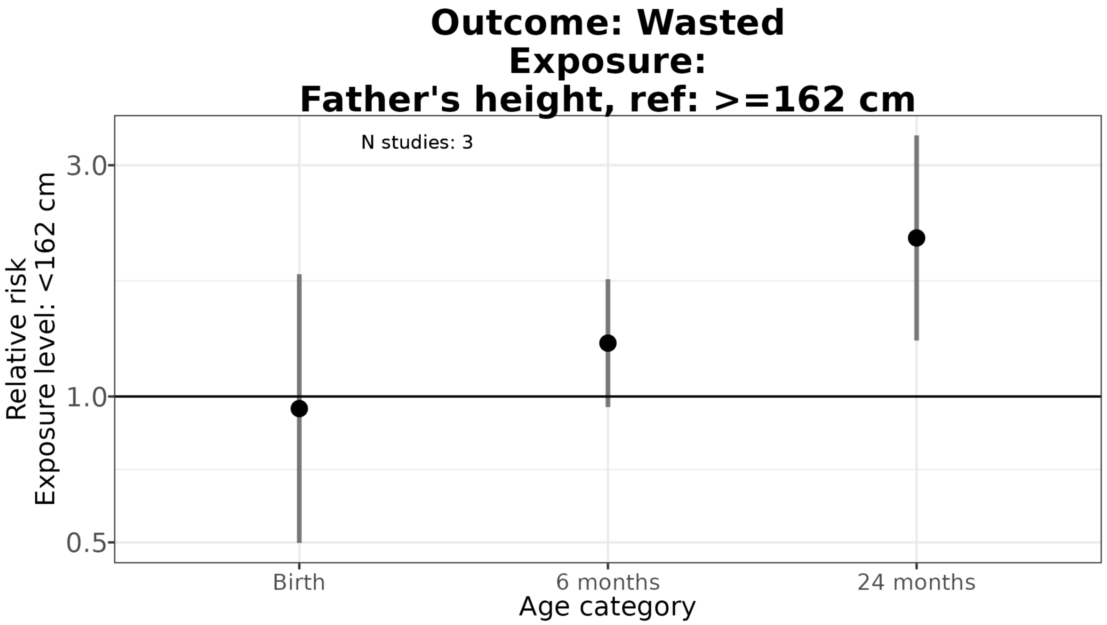
Outcome: Wasted
Exposure:
Father's height, ref: >=162 cm
N studies: 3
Relative risk
Exposure level: <162 cm
3.0
1.0
0.5
Birth
6 months
24 months
Age category

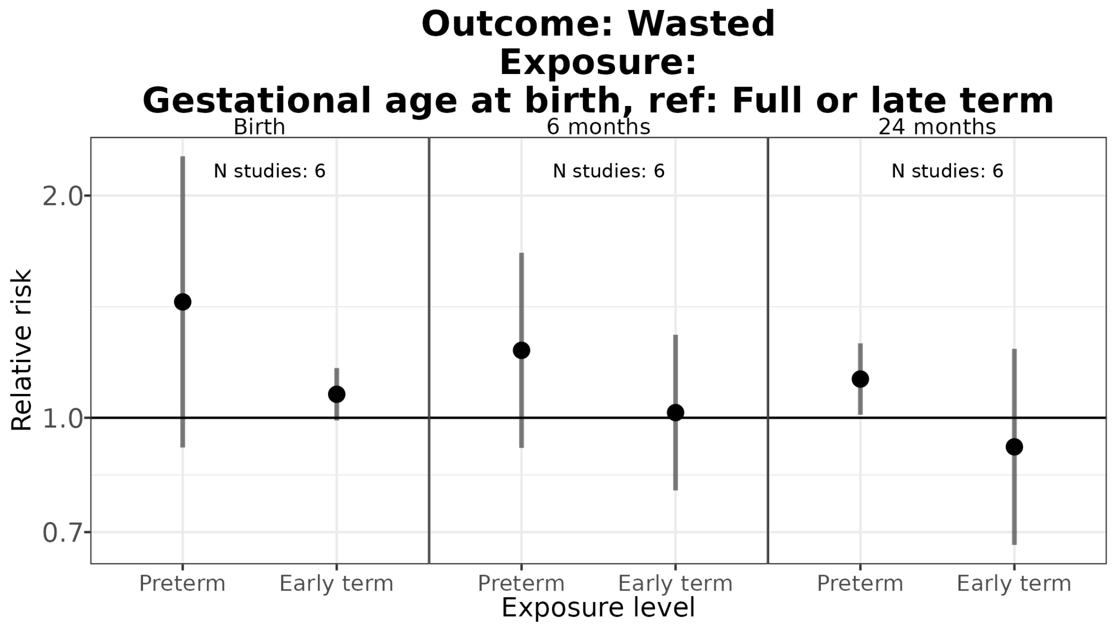
Outcome: Wasted
Exposure:
Gestational age at birth, ref: Full or late term
Birth
6 months
24 months
N studies: 6
N studies: 6
N studies: 6
2.0
1.0
0.7
Relative risk
Preterm
Early term
Preterm
Early term
Preterm
Early term
Exposure level

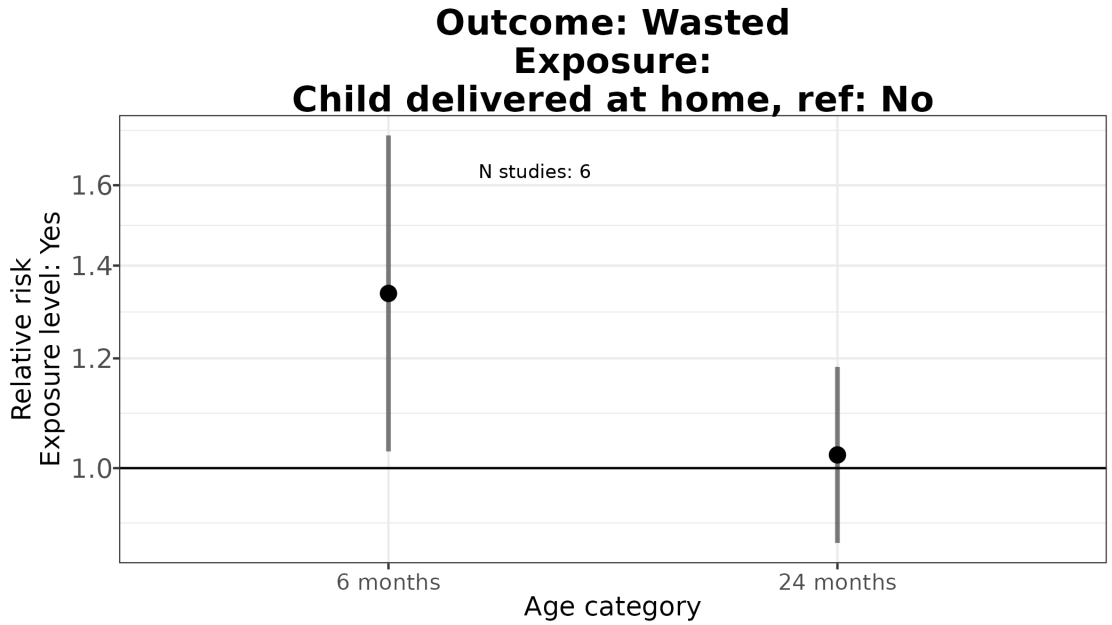
Outcome: Wasted
Exposure:
Child delivered at home, ref: No
N studies: 6
1.6
1.4
1.2
1.0
Relative risk
Exposure level: Yes
6 months
24 months
Age category

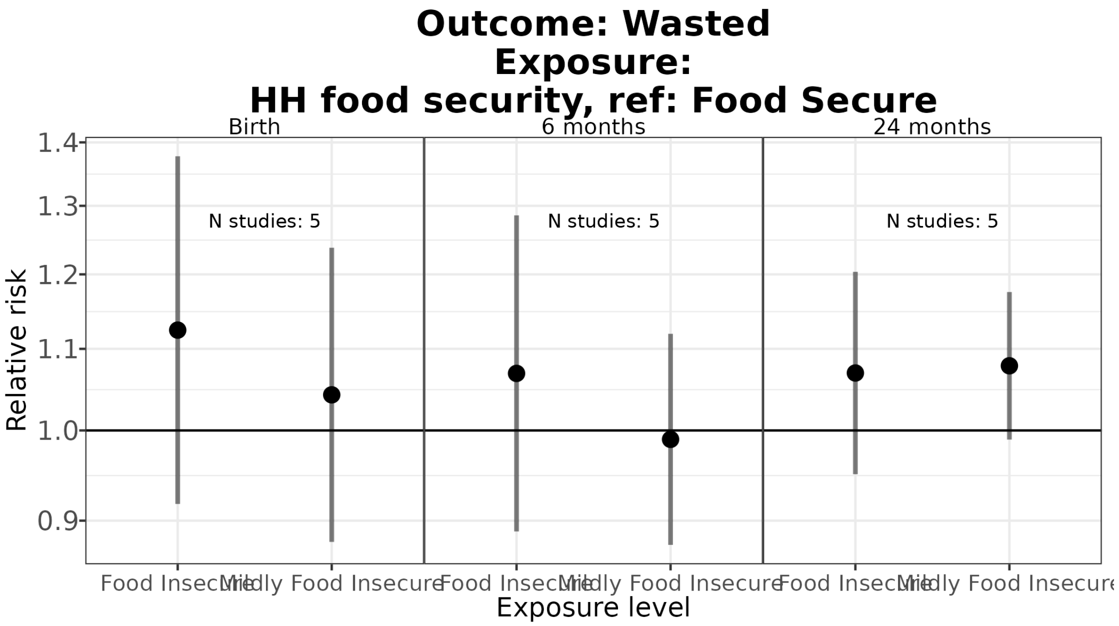
Outcome: Wasted
Exposure:
HH food security, ref: Food Secure
Birth
6 months
24 months
N studies: 5
N studies: 5
N studies: 5
Relative risk
1.4
1.3
1.2
1.1
1.0
0.9
Food Insecure
Mildly Food Insecure
Exposure level

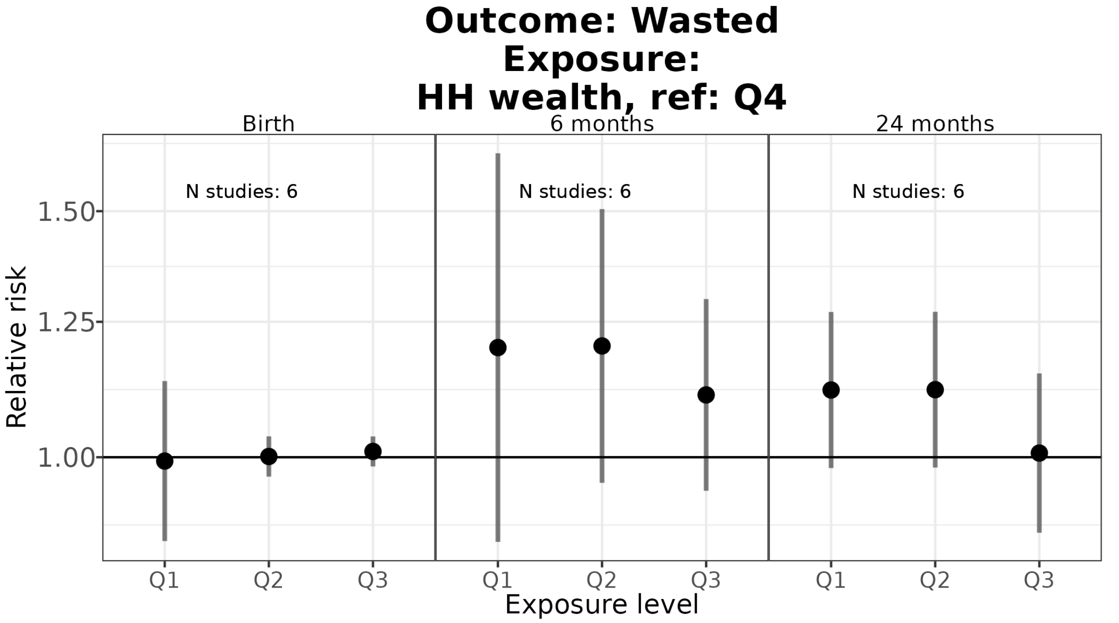
Outcome: Wasted
Exposure:
HH wealth, ref: Q4
Birth
6 months
24 months
N studies: 6
N studies: 6
N studies: 6
Relative risk
1.50
1.25
1.00
Q1
Q2
Q3
Exposure level

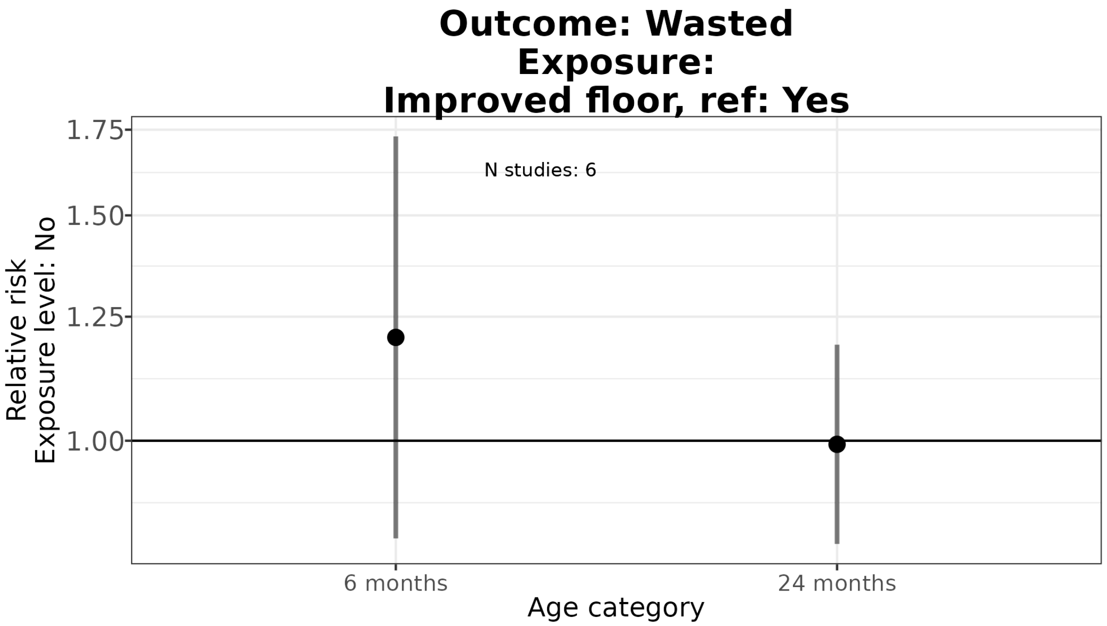
Outcome: Wasted
Exposure:
Improved floor, ref: Yes
Relative risk
Exposure level: No
1.75
1.50
1.25
1.00
N studies: 6
6 months
24 months
Age category

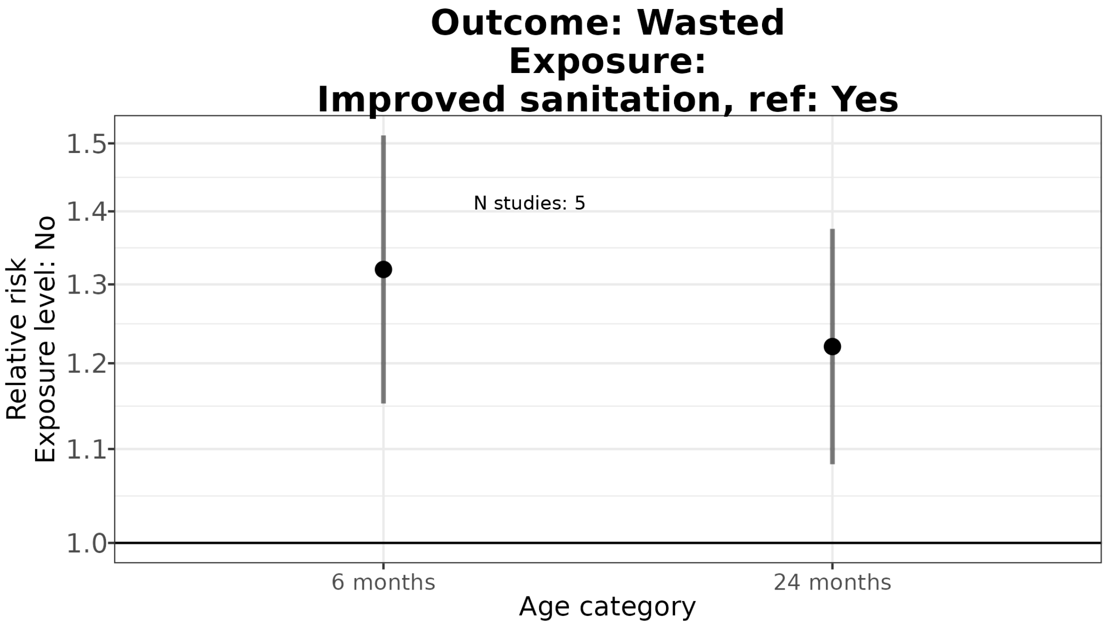
Outcome: Wasted
Exposure:
Improved sanitation, ref: Yes
Relative risk
Exposure level: No
1.5
1.4
1.3
1.2
1.1
1.0
N studies: 5
6 months
24 months
Age category

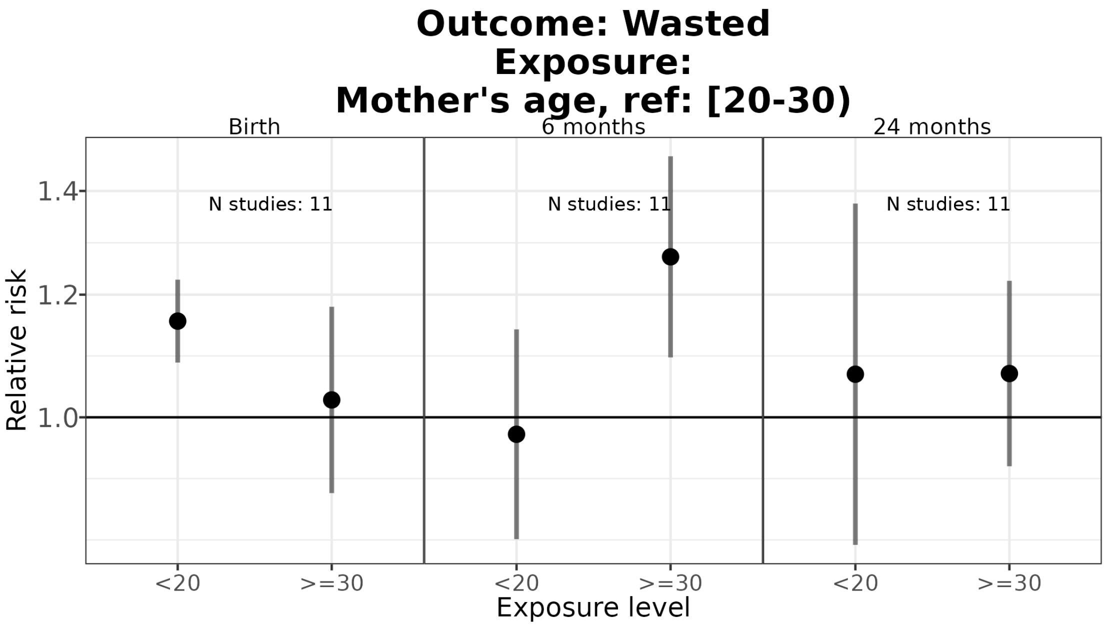
Outcome: Wasted
Exposure:
Mother's age, ref: [20-30)
Birth
6 months
24 months
N studies: 11
N studies: 11
N studies: 11
1.4
1.2
1.0
Relative risk
<20
>=30
<20
>=30
<20
>=30
Exposure level

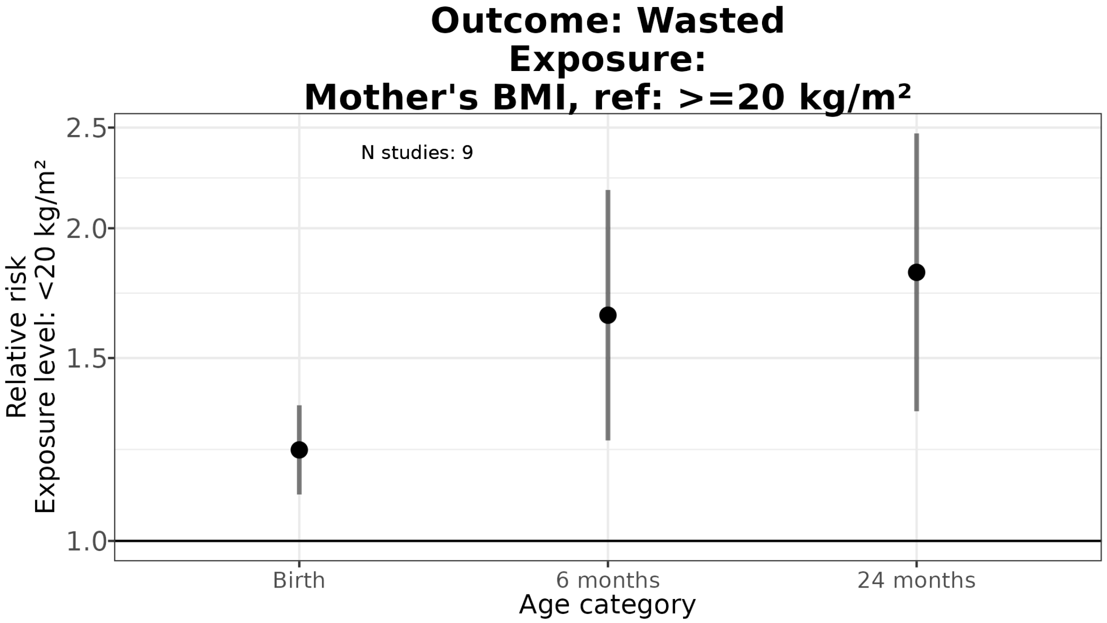
Outcome: Wasted
Exposure:
Mother's BMI, ref: >=20 kg/m²
N studies: 9
2.5
2.0
1.5
1.0
Relative risk
Exposure level: <20 kg/m²
Birth
6 months
24 months
Age category

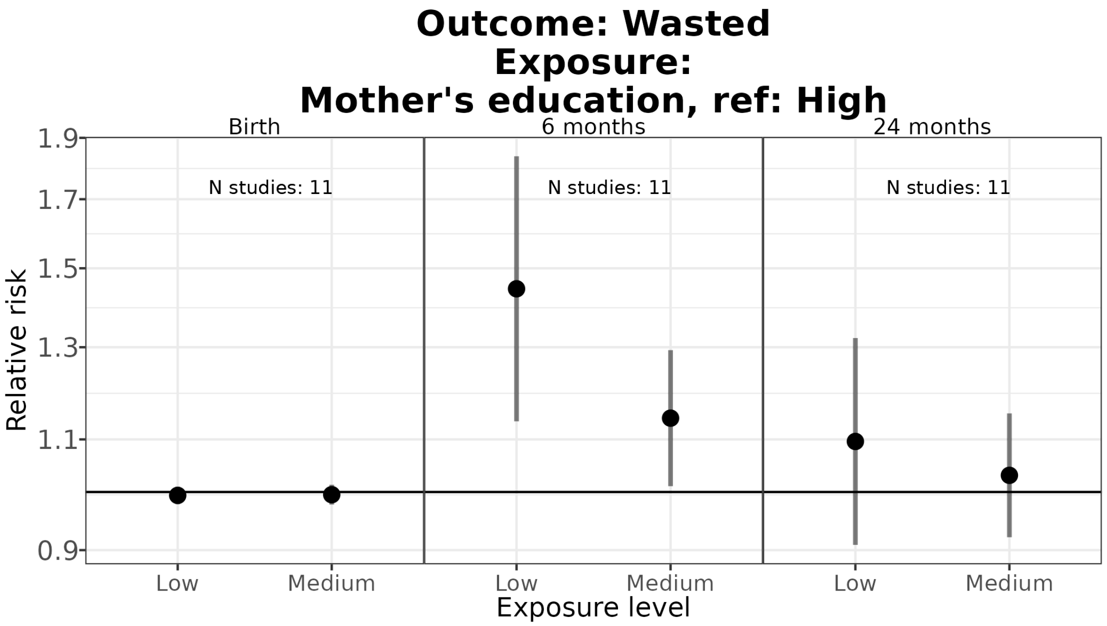

Outcome: Wasted
Exposure:
Mother's education, ref: High
Birth
6 months
24 months
N studies: 11
N studies: 11
N studies: 11
Relative risk
1.9
1.7
1.5
1.3
1.1
0.9
Low
Medium
Low
Medium
Low
Medium
Exposure level


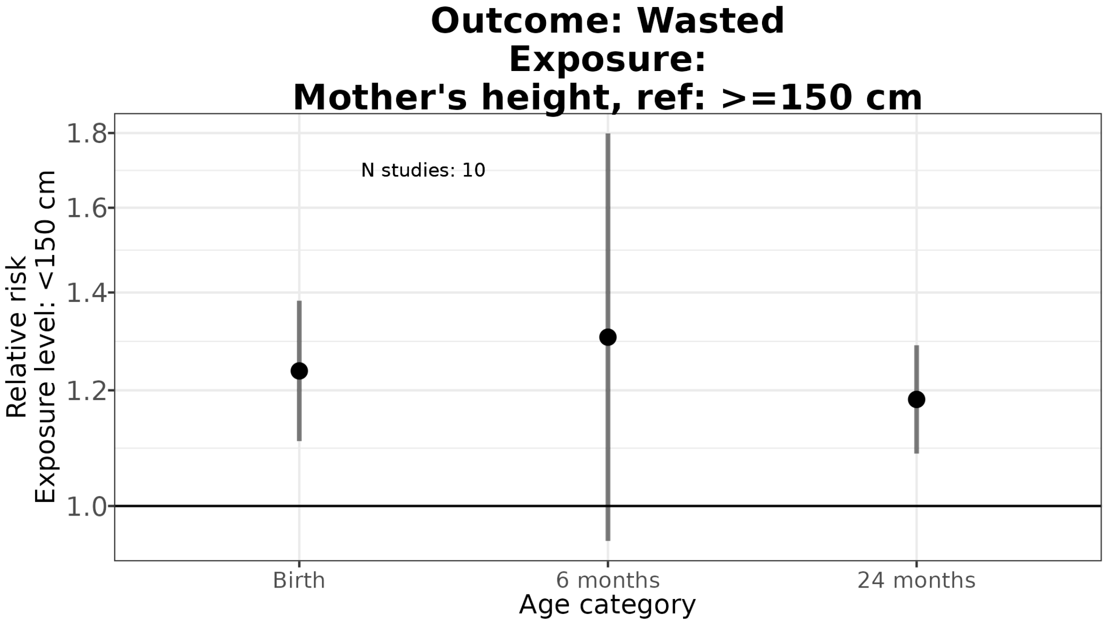

Outcome: Wasted
Exposure:
Mother's height, ref: >=150 cm
N studies: 10
Relative risk
Exposure level: <150 cm
1.8
1.6
1.4
1.2
1.0
Birth
6 months
24 months
Age category

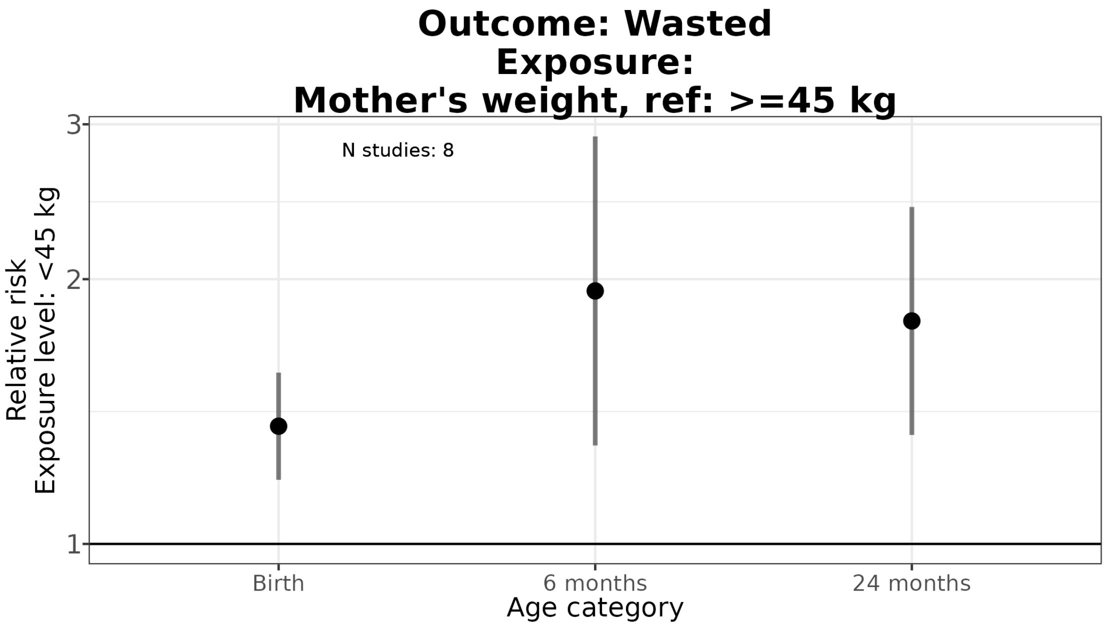
Outcome: Wasted
Exposure:
Mother's weight, ref: >=45 kg
N studies: 8
Relative risk
Exposure level: <45 kg
3
2
1
Birth
6 months
24 months
Age category

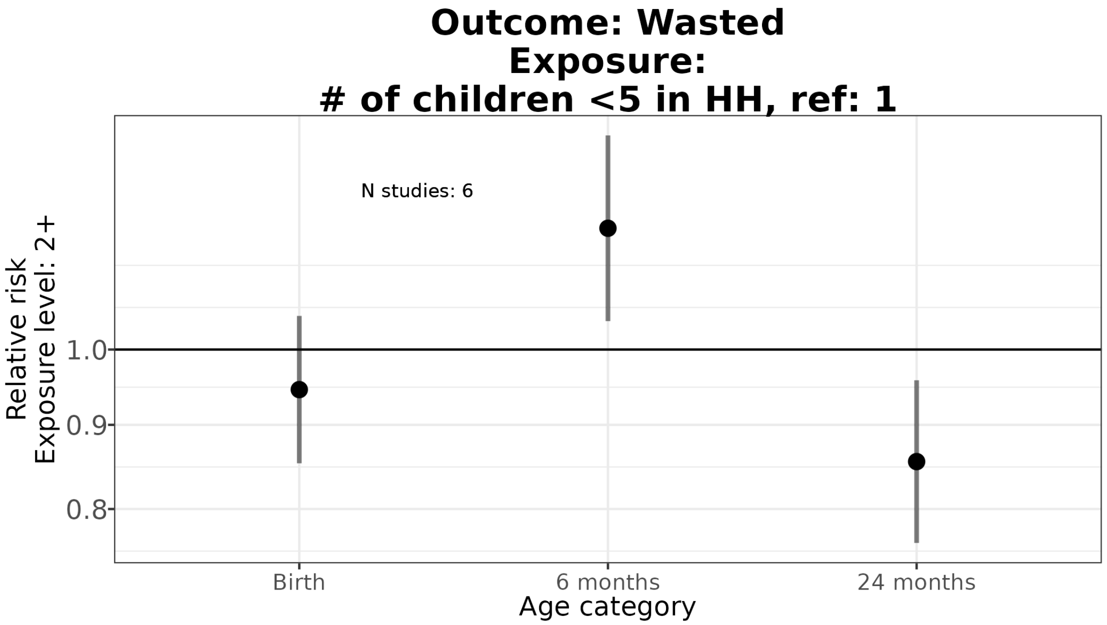
Outcome: Wasted
Exposure:
# of children <5 in HH, ref: 1
N studies: 6
Relative risk
Exposure level: 2+
1.0
0.9
0.8
Birth
6 months
24 months
Age category

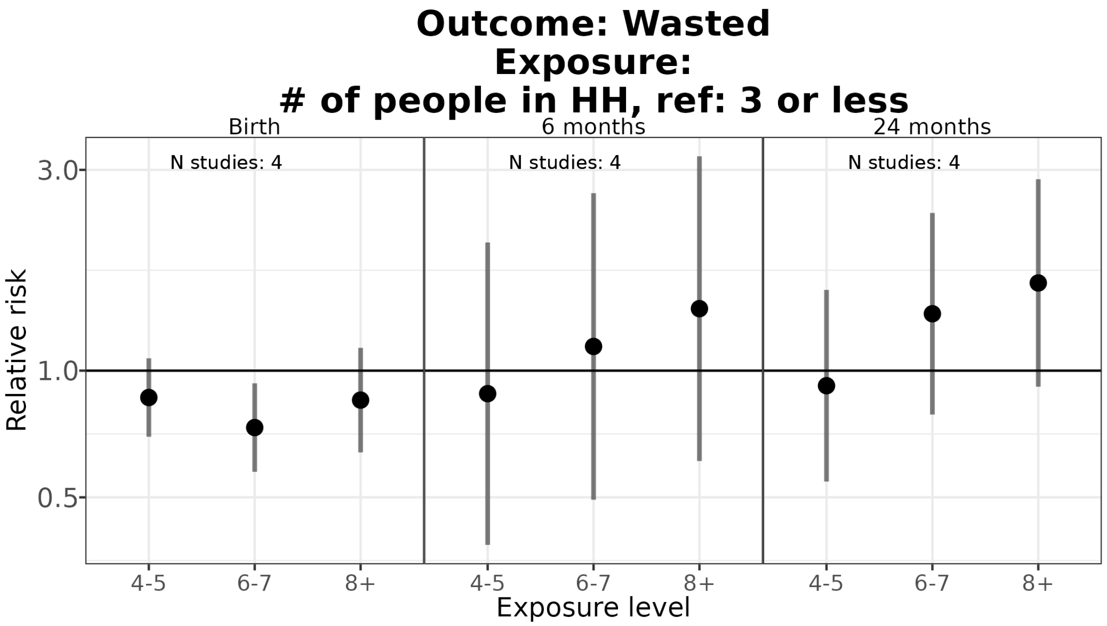
Outcome: Wasted
Exposure:
# of people in HH, ref: 3 or less
Birth
6 months
24 months
N studies: 4
N studies: 4
N studies: 4
3.0
1.0
0.5
Relative risk
4-5
6-7
8+
4-5
6-7
8+
4-5
6-7
8+
Exposure level

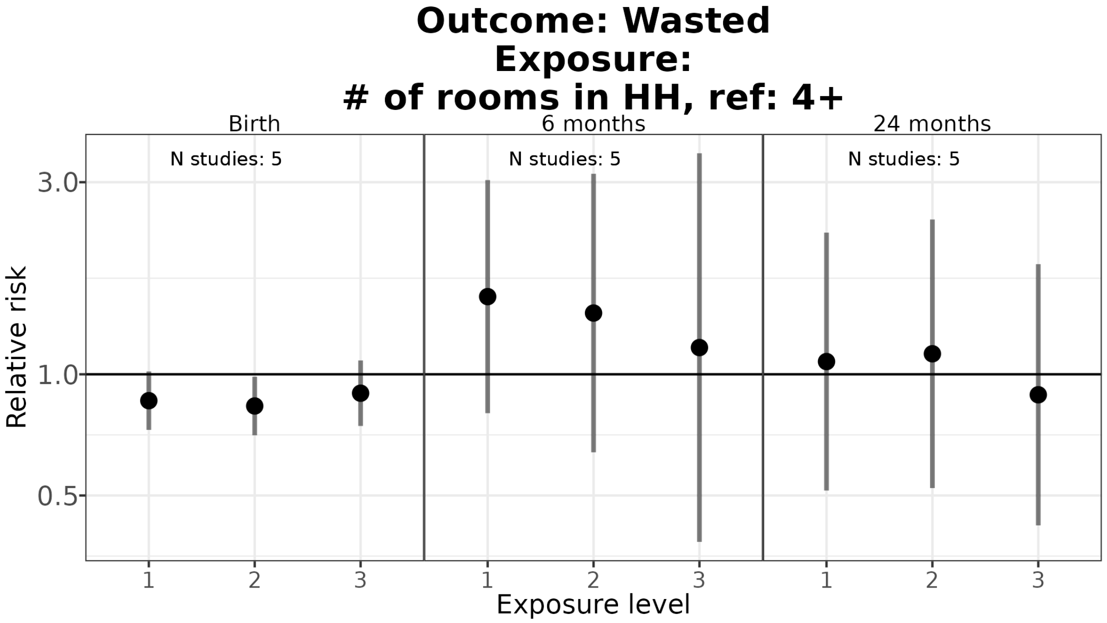
Outcome: Wasted
Exposure:
# of rooms in HH, ref: 4+
Birth
6 months
24 months
N studies: 5
N studies: 5
N studies: 5
3.0
1.0
0.5
Relative risk
1
2
3
1
2
3
1
2
3
Exposure level

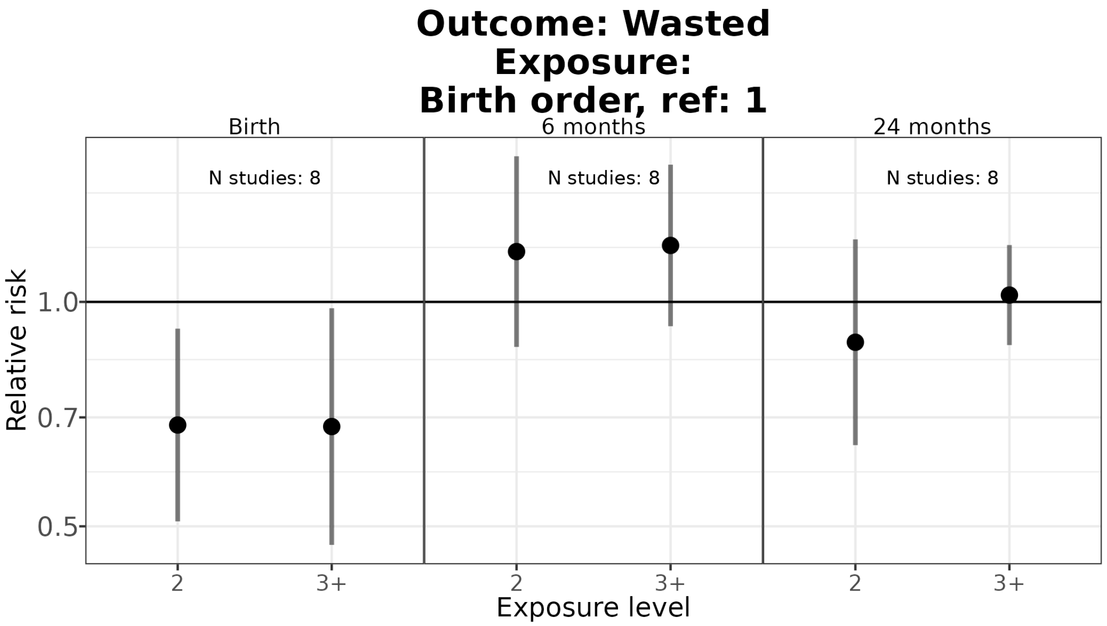
Outcome: Wasted
Exposure:
Birth order, ref: 1
Birth
6 months
24 months
N studies: 8
N studies: 8
N studies: 8
Relative risk
1.0
0.7
0.5
2
3+
2
3+
2
3+
Exposure level

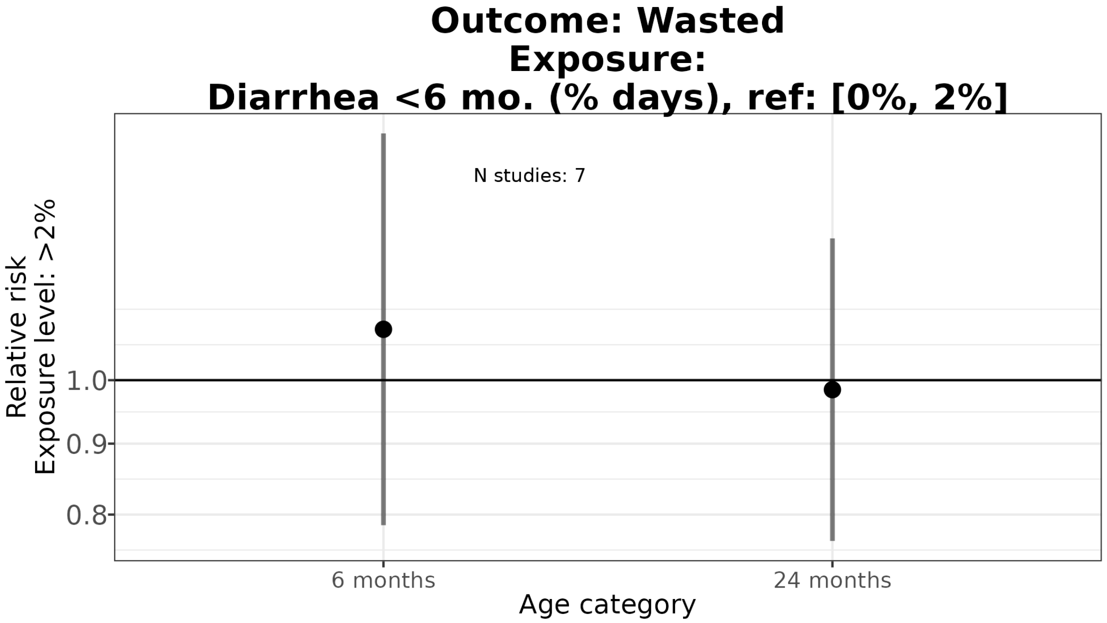
Outcome: Wasted
Exposure:
Diarrhea <6 mo. (% days), ref: [0%, 2%]
N studies: 7
Relative risk
Exposure level: >2%
1.0
0.9
0.8
6 months
24 months
Age category

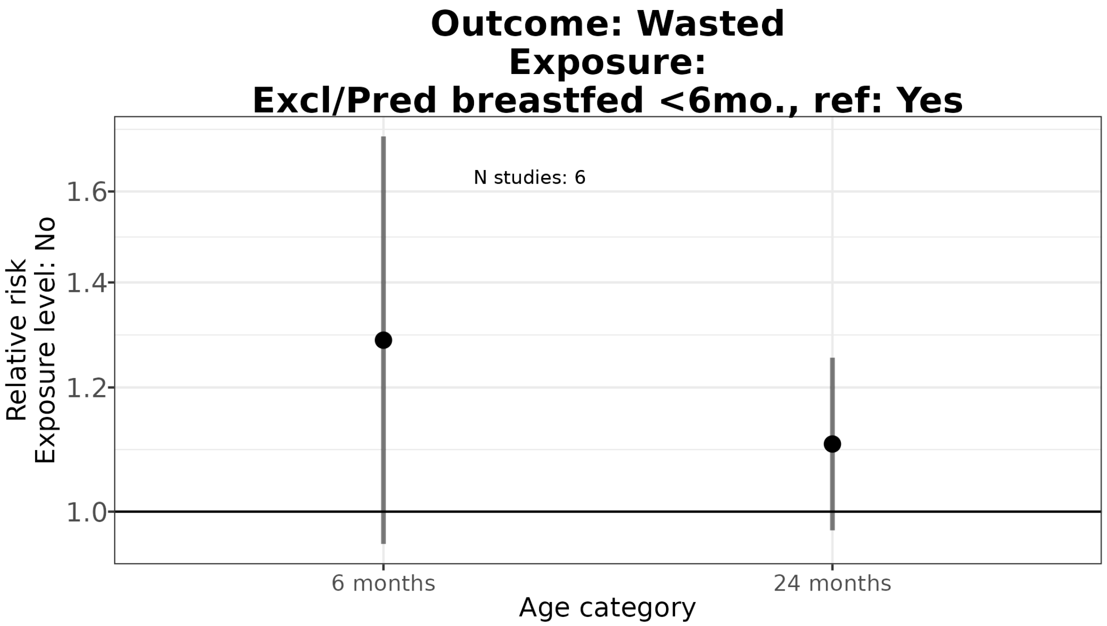

Outcome: Wasted
Exposure:
Excl/Pred breastfed <6mo., ref: Yes
N studies: 6
Relative risk
Exposure level: No
1.6
1.4
1.2
1.0
6 months
24 months
Age category


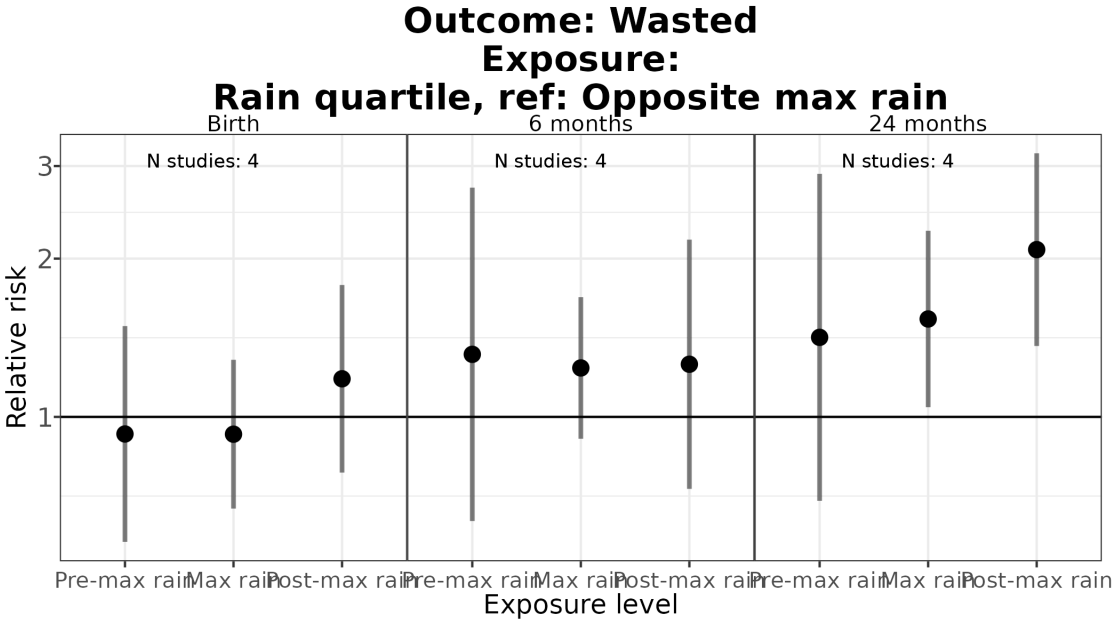

Outcome: Wasted
Exposure:
Rain quartile, ref: Opposite max rain
Birth
6 months
24 months
N studies: 4
N studies: 4
N studies: 4
Relative risk
3
2
1
Pre-max rain
Max rain
Post-max rain
Pre-max rain
Max rain
Post-max rain
Pre-max rain
Max rain
Post-max rain
Exposure level

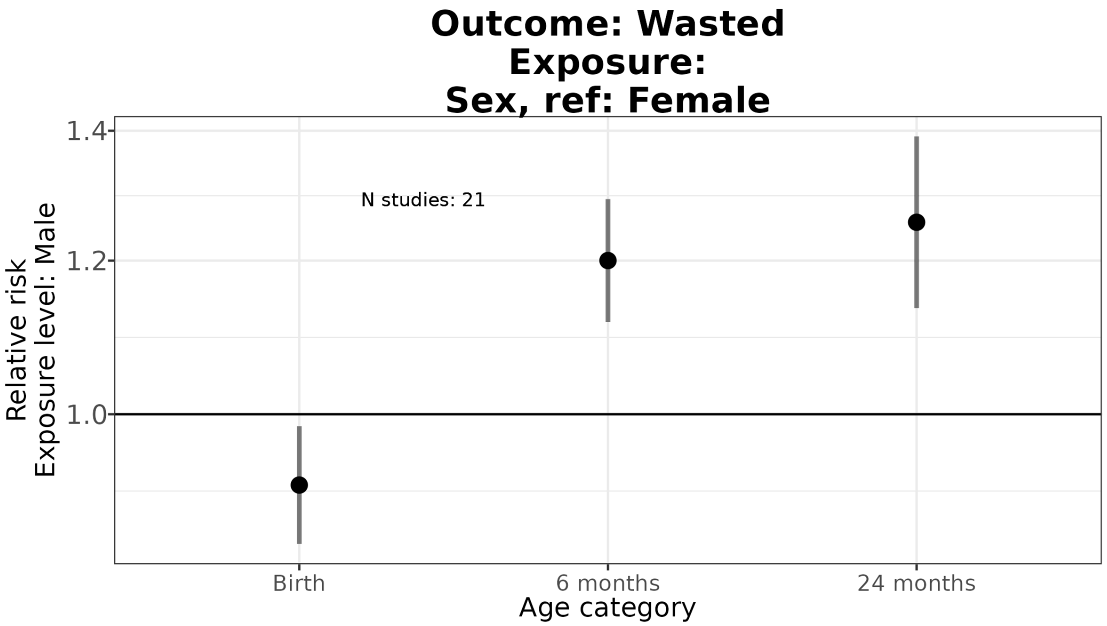
Outcome: Wasted
Exposure:
Sex, ref: Female
N studies: 21
Relative risk
Exposure level: Male
1.4
1.2
1.0
Birth
6 months
24 months
Age category

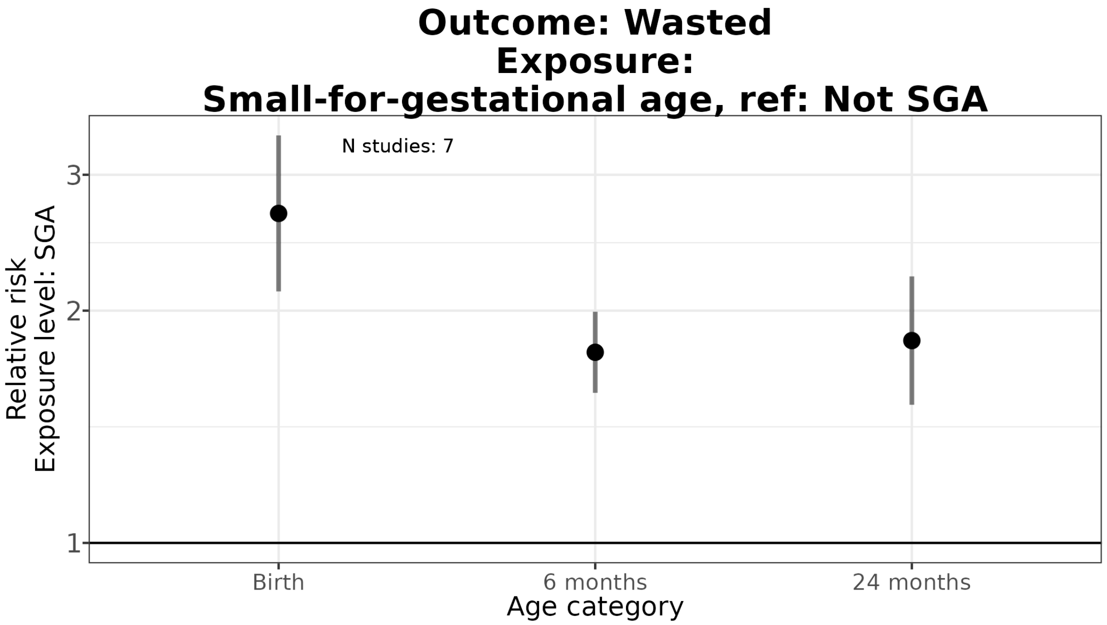
Outcome: Wasted
Exposure:
Small-for-gestational age, ref: Not SGA
N studies: 7
Relative risk
Exposure level: SGA
3
2
1
Birth
6 months
24 months
Age category

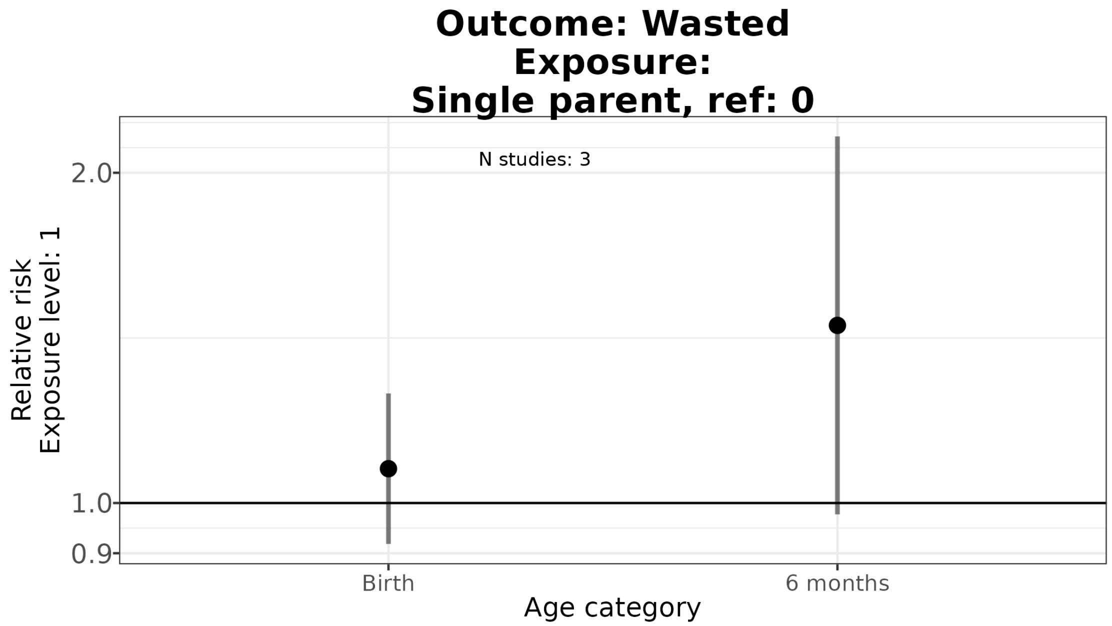
Outcome: Wasted
Exposure:
Single parent, ref: 0
N studies: 3
Relative risk
Exposure level: 1
2.0
1.0
0.9
Birth
6 months
Age category

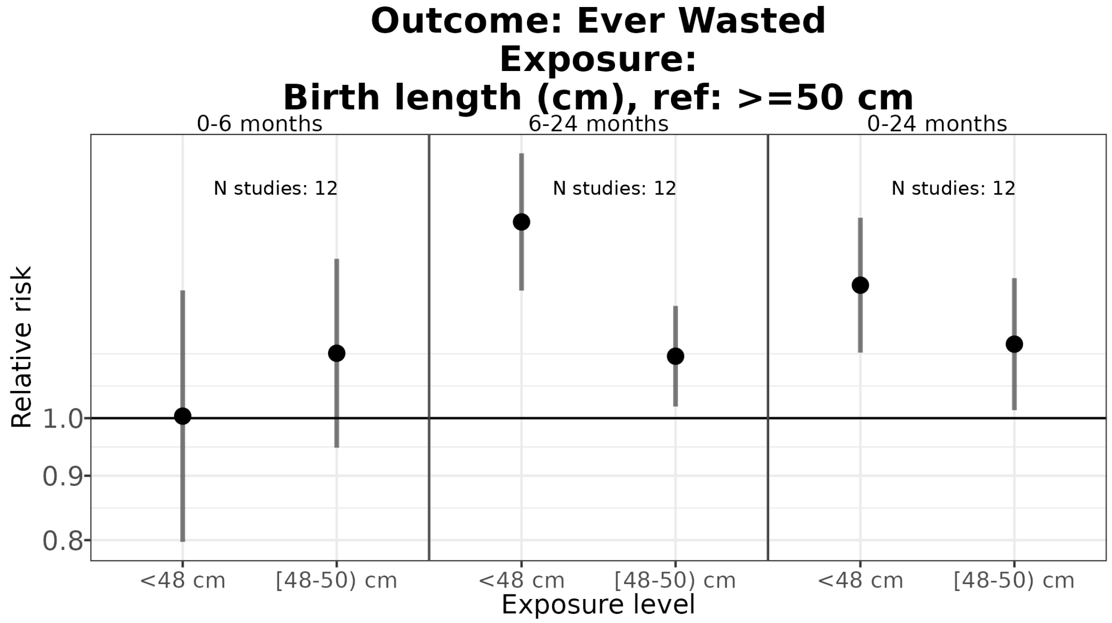
Outcome: Ever Wasted
Exposure:
Birth length (cm), ref: >=50 cm
0-6 months
6-24 months
0-24 months
N studies: 12
N studies: 12
N studies: 12
Relative risk
1.0
0.9
0.8
<48 cm
[48-50) cm
<48 cm
[48-50) cm
<48 cm
[48-50) cm
Exposure level

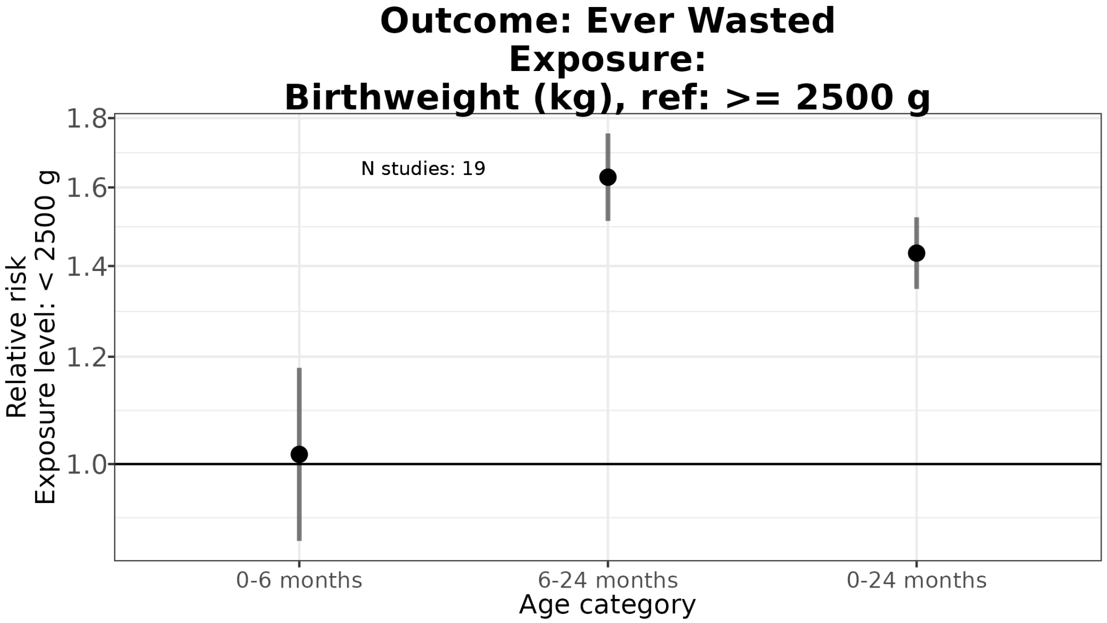
Outcome: Ever Wasted
Exposure:
Birthweight (kg), ref: >= 2500 g
Relative risk
Exposure level: < 2500 g
N studies: 19
1.8
1.6
1.4
1.2
1.0
0-6 months
6-24 months
0-24 months
Age category

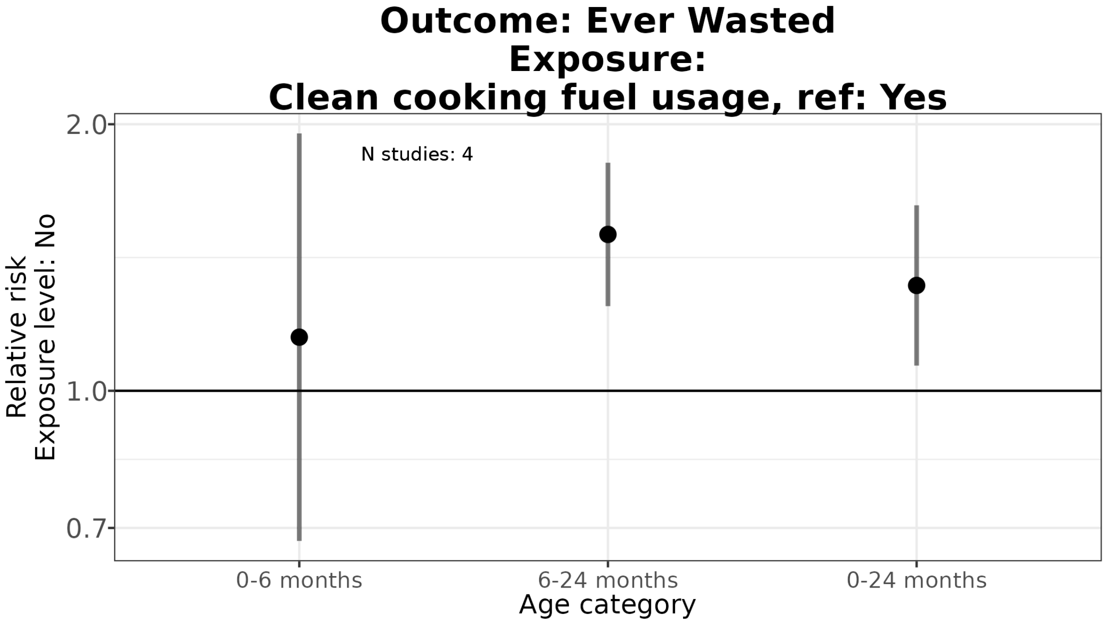
Outcome: Ever Wasted
Exposure:
Clean cooking fuel usage, ref: Yes
Relative risk
Exposure level: No
N studies: 4
2.0
1.0
0.7
0-6 months
6-24 months
0-24 months
Age category

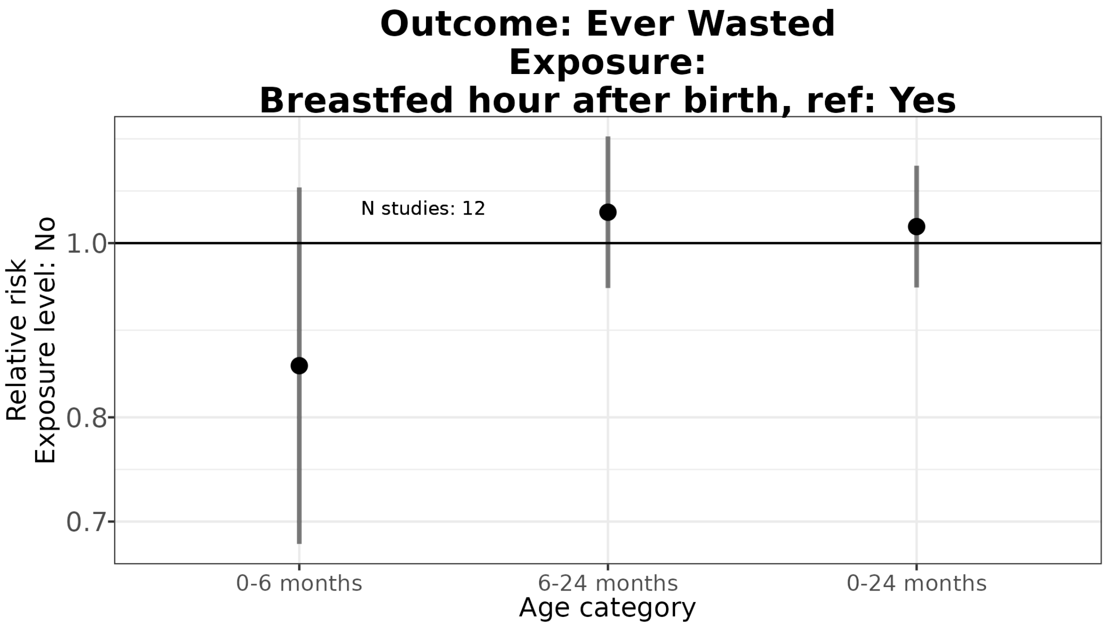
Outcome: Ever Wasted
Exposure:
Breastfed hour after birth, ref: Yes
Relative risk
Exposure level: No
N studies: 12
1.0
0.8
0.7
0-6 months
6-24 months
0-24 months
Age category

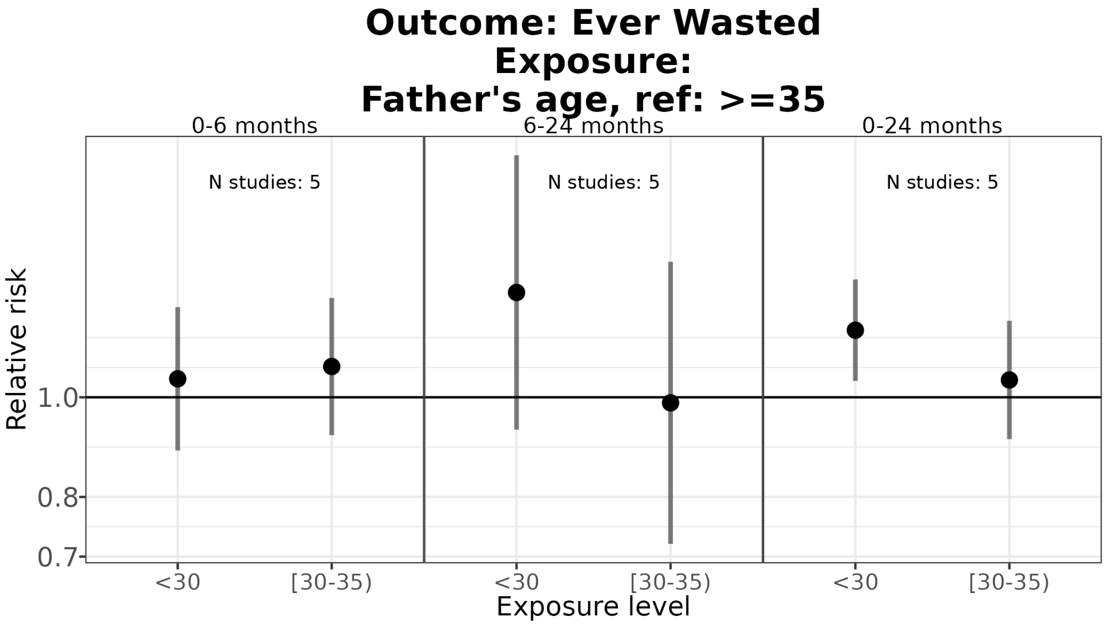
Outcome: Ever Wasted
Exposure:
Father's age, ref: >=35
0-6 months
6-24 months
0-24 months
N studies: 5
N studies: 5
N studies: 5
Relative risk
1.0
0.8
0.7
<30
[30-35)
<30
[30-35)
<30
[30-35)
Exposure level

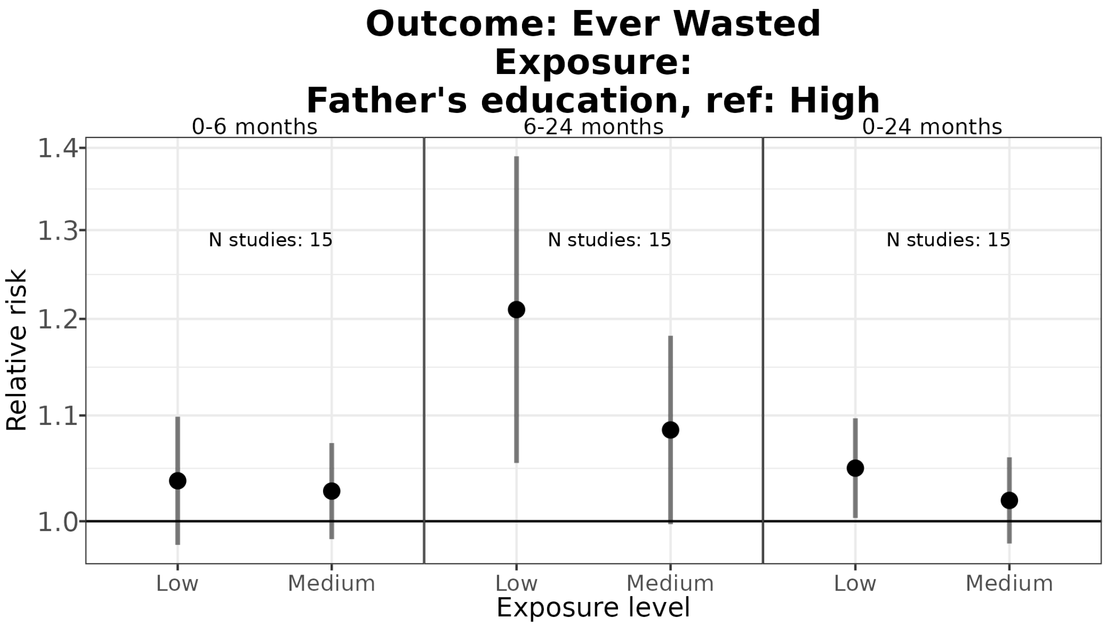
Outcome: Ever Wasted
Exposure:
Father's education, ref: High
0-6 months
6-24 months
0-24 months
N studies: 15
N studies: 15
N studies: 15
Relative risk
1.4
1.3
1.2
1.1
1.0
Low
Medium
Low
Medium
Low
Medium
Exposure level

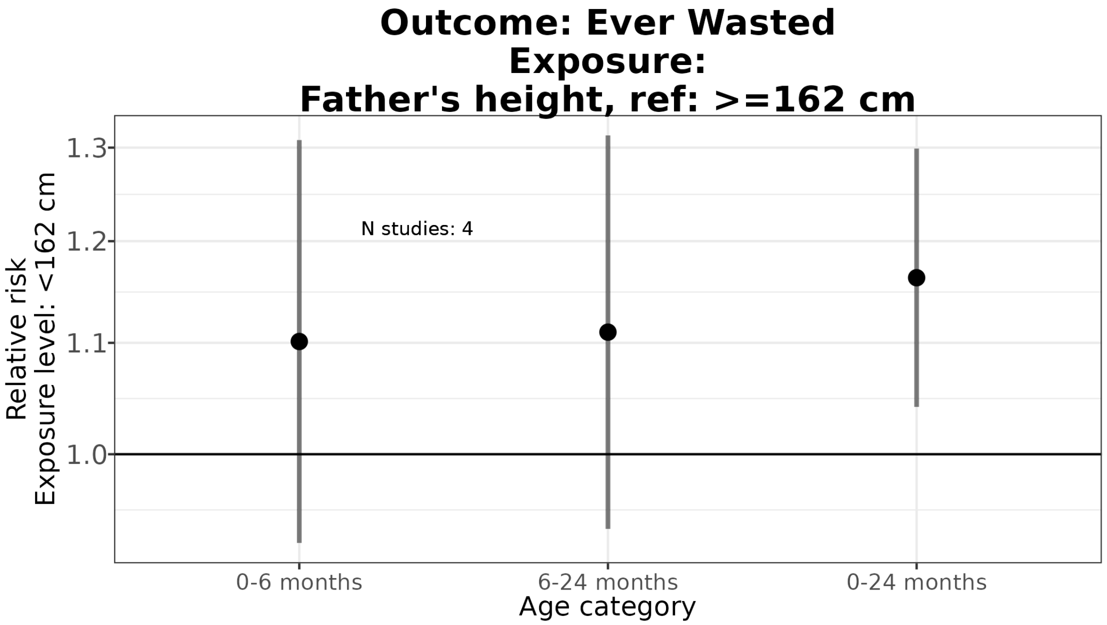
Outcome: Ever Wasted
Exposure:
Father's height, ref: >=162 cm
N studies: 4
Relative risk
Exposure level: <162 cm
1.3
1.2
1.1
1.0
0-6 months
6-24 months
0-24 months
Age category

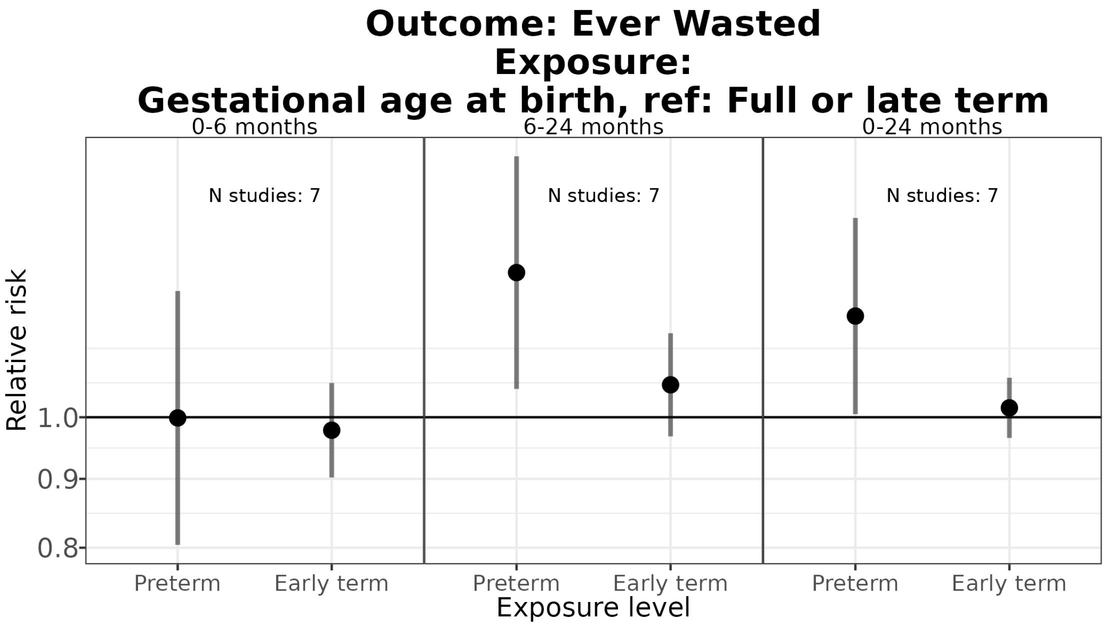

Outcome: Ever Wasted
Exposure:
Gestational age at birth, ref: Full or late term
0-6 months
6-24 months
0-24 months
N studies: 7
N studies: 7
N studies: 7
Relative risk
1.0
0.9
0.8
Preterm
Early term
Preterm
Early term
Preterm
Early term
Exposure level


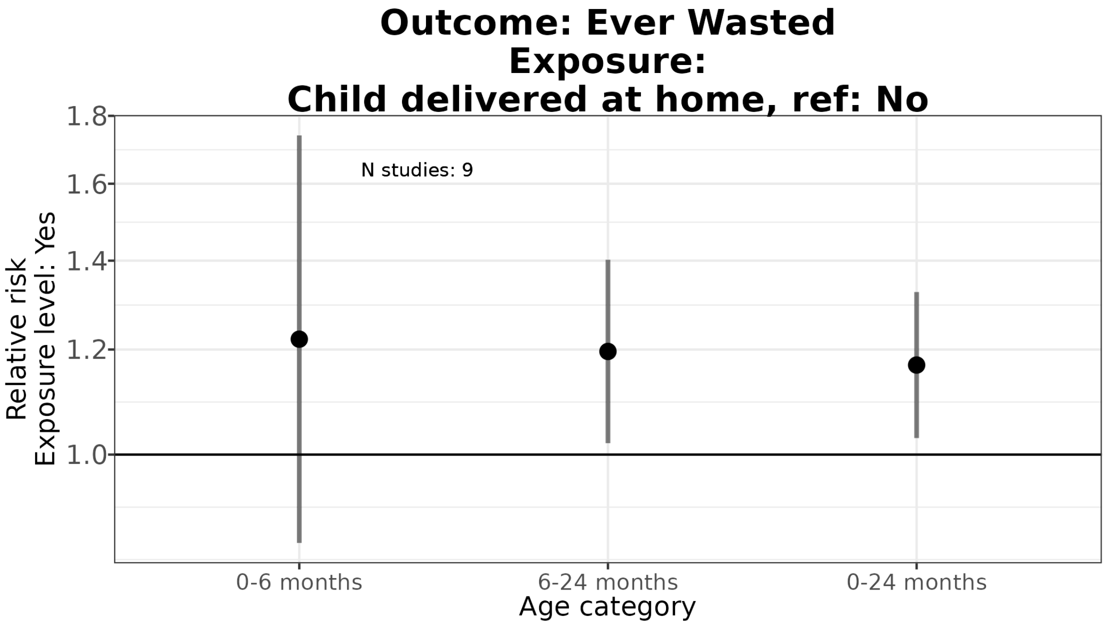

Outcome: Ever Wasted
Exposure:
Child delivered at home, ref: No
1.8
1.6
1.4
1.2
1.0
N studies: 9
Relative risk
Exposure level: Yes
0-6 months
6-24 months
0-24 months
Age category

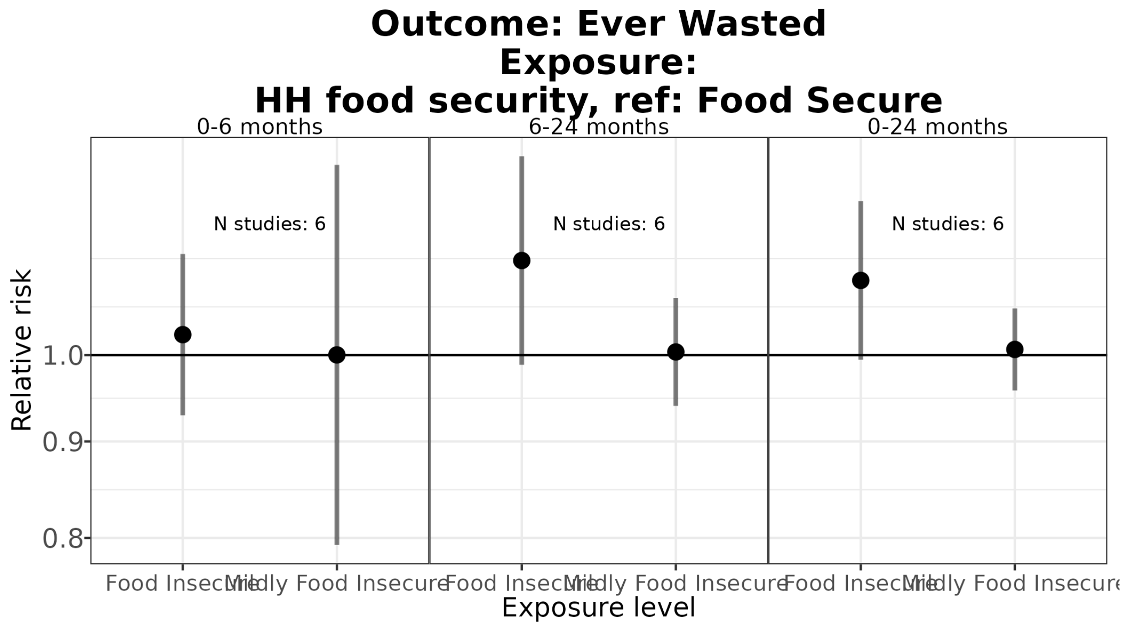
Outcome: Ever Wasted
Exposure:
HH food security, ref: Food Secure
0-6 months
6-24 months
0-24 months
N studies: 6
N studies: 6
N studies: 6
Relative risk
1.0
0.9
0.8
Food Insecure
Mildly Food Insecure
Food Insecure
Mildly Food Insecure
Food Insecure
Mildly Food Insecure
Exposure level

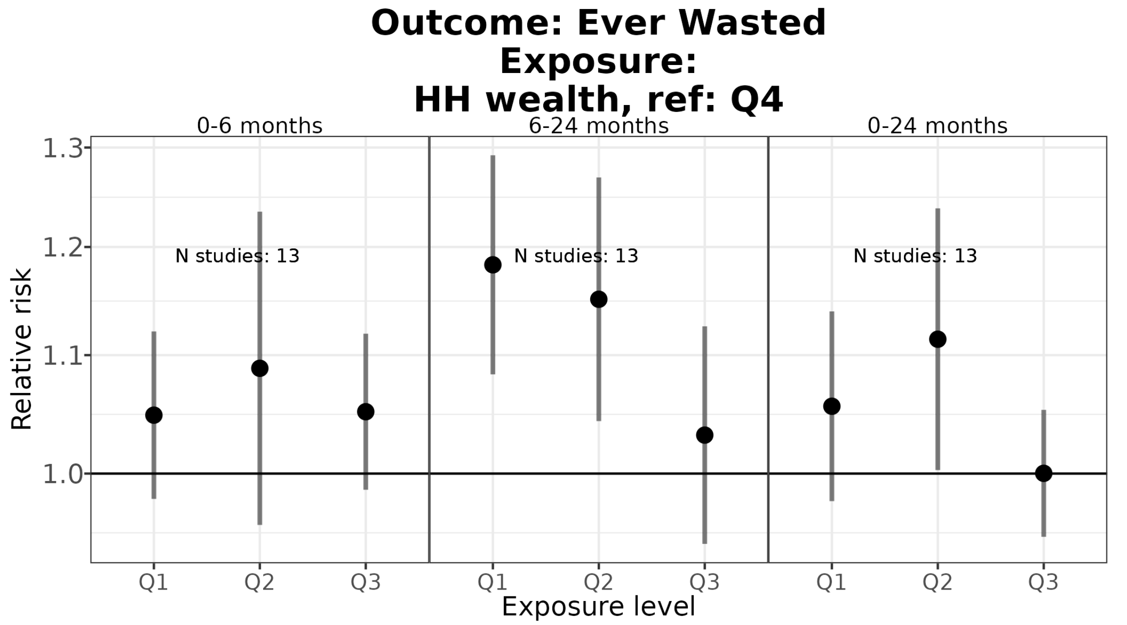
Outcome: Ever Wasted
Exposure:
HH wealth, ref: Q4
0-6 months
6-24 months
0-24 months
N studies: 13
N studies: 13
N studies: 13
Relative risk
1.3
1.2
1.1
1.0
Q1
Q2
Q3
Q1
Q2
Q3
Q1
Q2
Q3
Exposure level

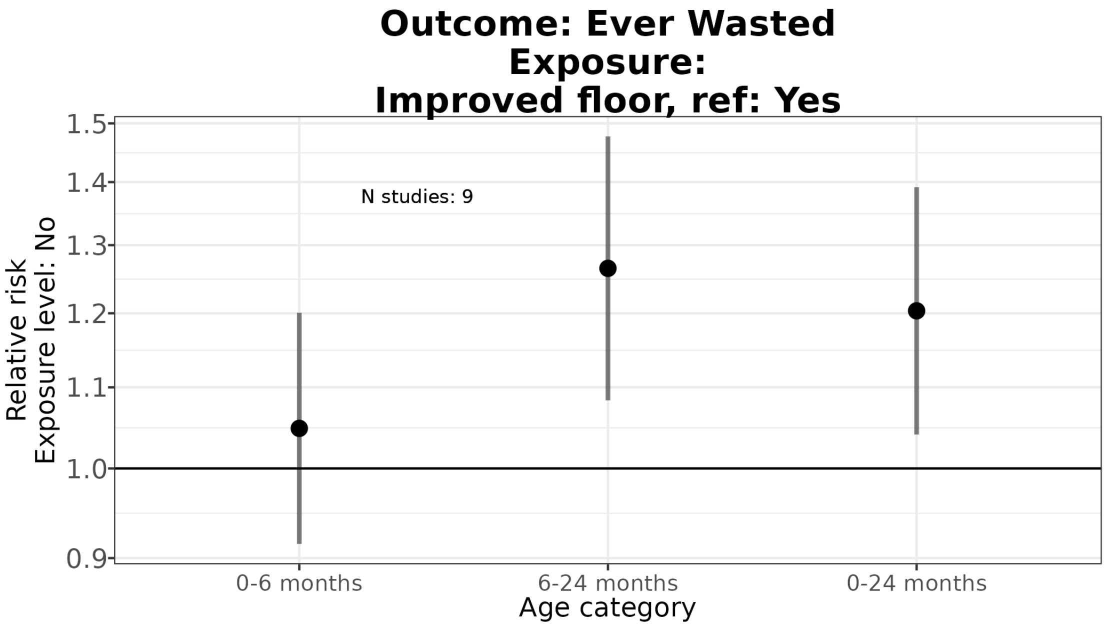

Outcome: Ever Wasted
Exposure:
Improved floor, ref: Yes
Relative risk
Exposure level: No
1.5
1.4
1.3
1.2
1.1
1.0
0.9
N studies: 9
0-6 months
6-24 months
0-24 months
Age category


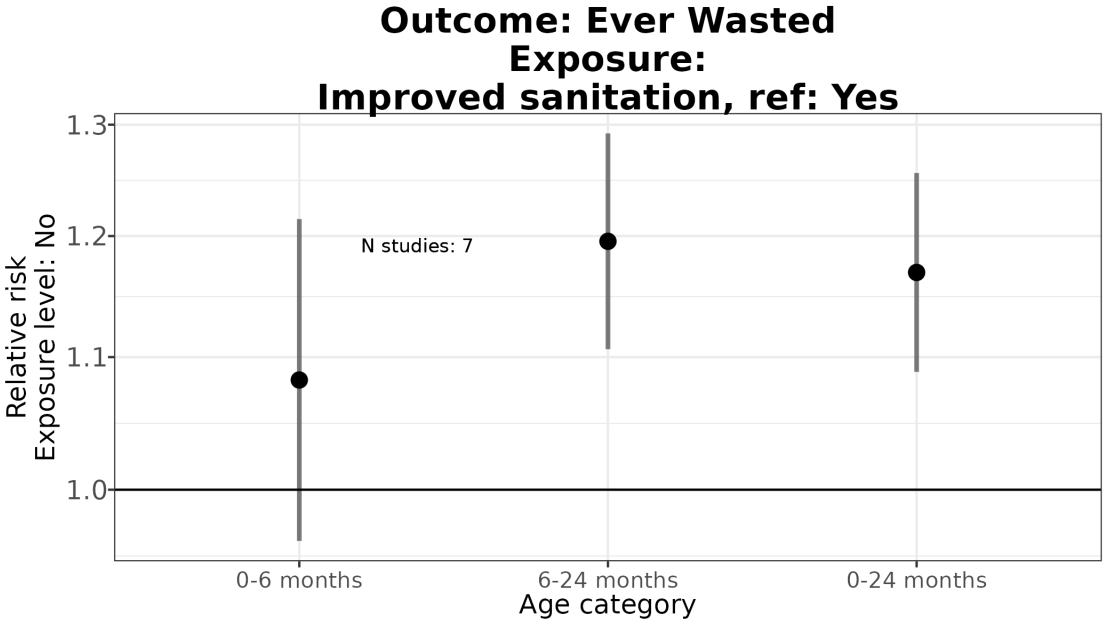

Outcome: Ever Wasted
Exposure:
Improved sanitation, ref: Yes
Relative risk
Exposure level: No
1.3
1.2
1.1
1.0
N studies: 7
0-6 months
6-24 months
0-24 months
Age category

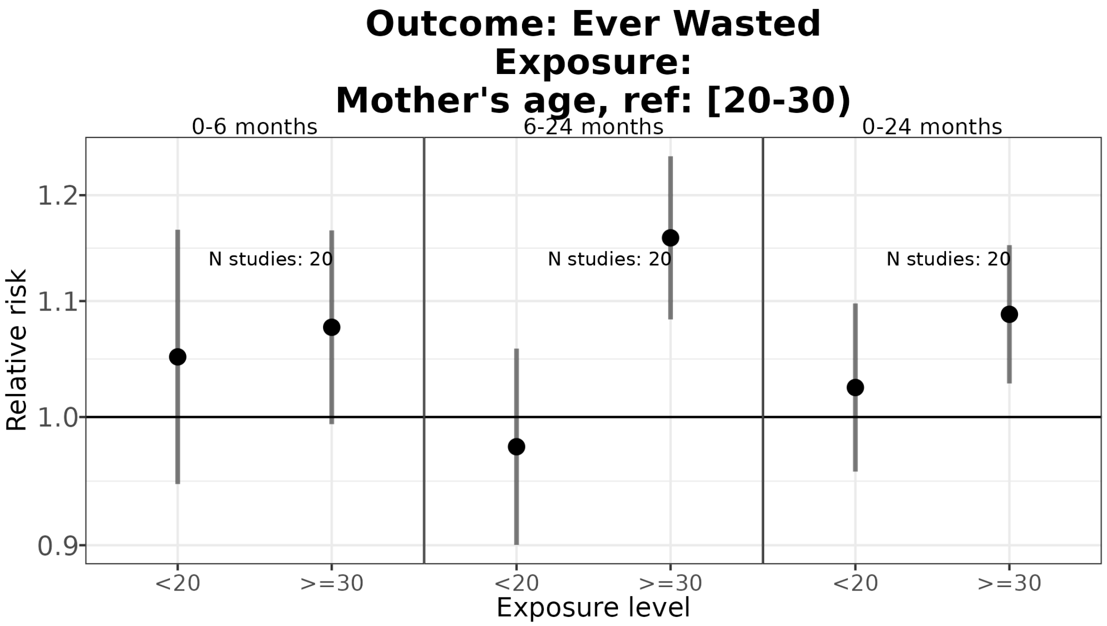
Outcome: Ever Wasted
Exposure:
Mother's age, ref: [20-30)
0-6 months
6-24 months
0-24 months
N studies: 20
N studies: 20
N studies: 20
Relative risk
1.2
1.1
1.0
0.9
<20
>=30
<20
>=30
<20
>=30
Exposure level

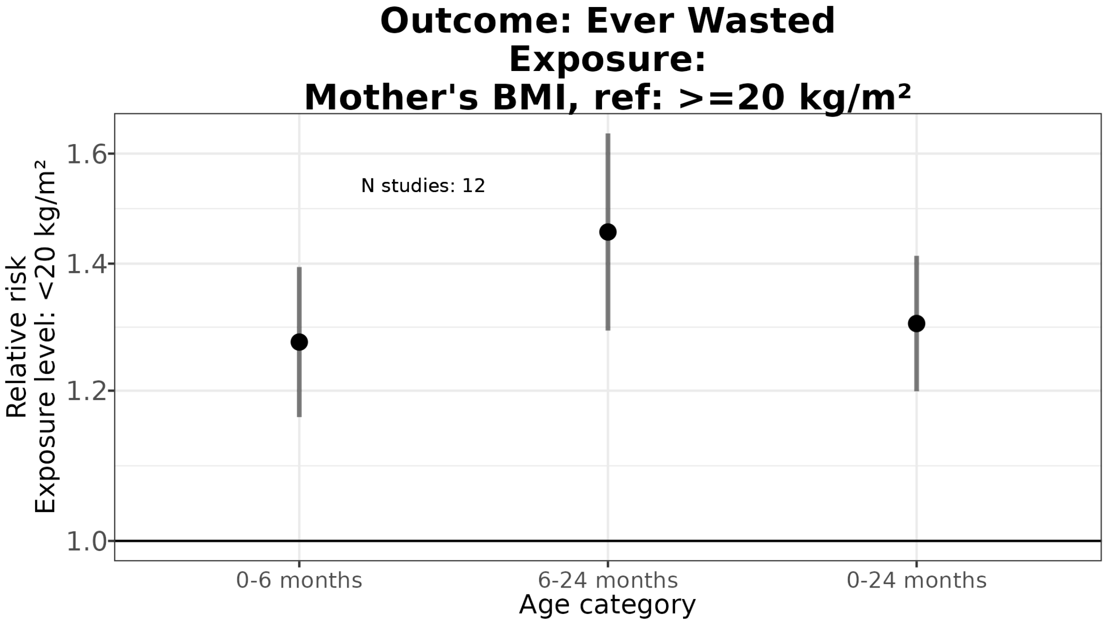
Outcome: Ever Wasted
Exposure:
Mother's BMI, ref: >=20 kg/m²
N studies: 12
Relative risk
Exposure level: <20 kg/m²
1.6
1.4
1.2
1.0
0-6 months
6-24 months
0-24 months
Age category

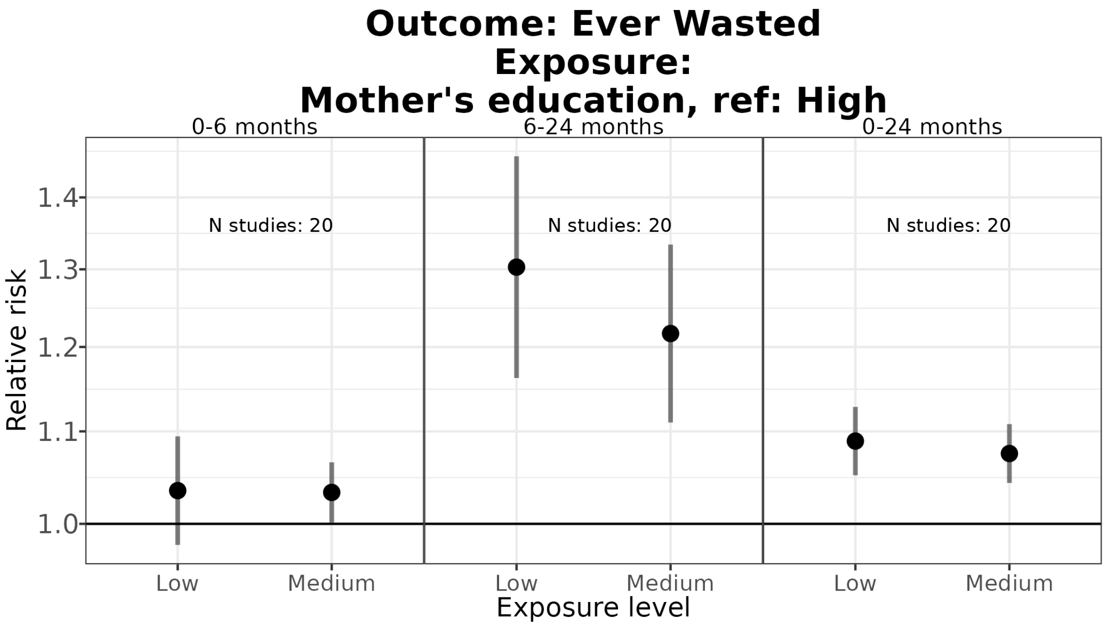
Outcome: Ever Wasted
Exposure:
Mother's education, ref: High
0-6 months
6-24 months
0-24 months
N studies: 20
N studies: 20
N studies: 20
Relative risk
1.4
1.3
1.2
1.1
1.0
Low
Medium
Low
Medium
Low
Medium
Exposure level

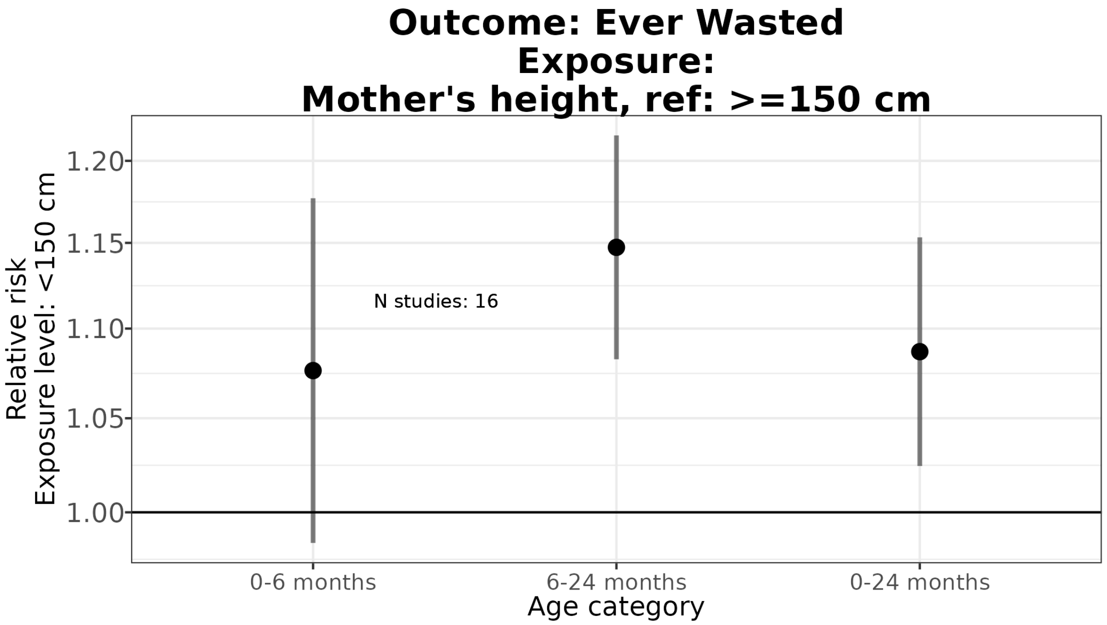
Outcome: Ever Wasted
Exposure:
Mother's height, ref: >=150 cm
Relative risk
Exposure level: <150 cm
1.20
1.15
1.10
1.05
1.00
N studies: 16
0-6 months
6-24 months
0-24 months
Age category

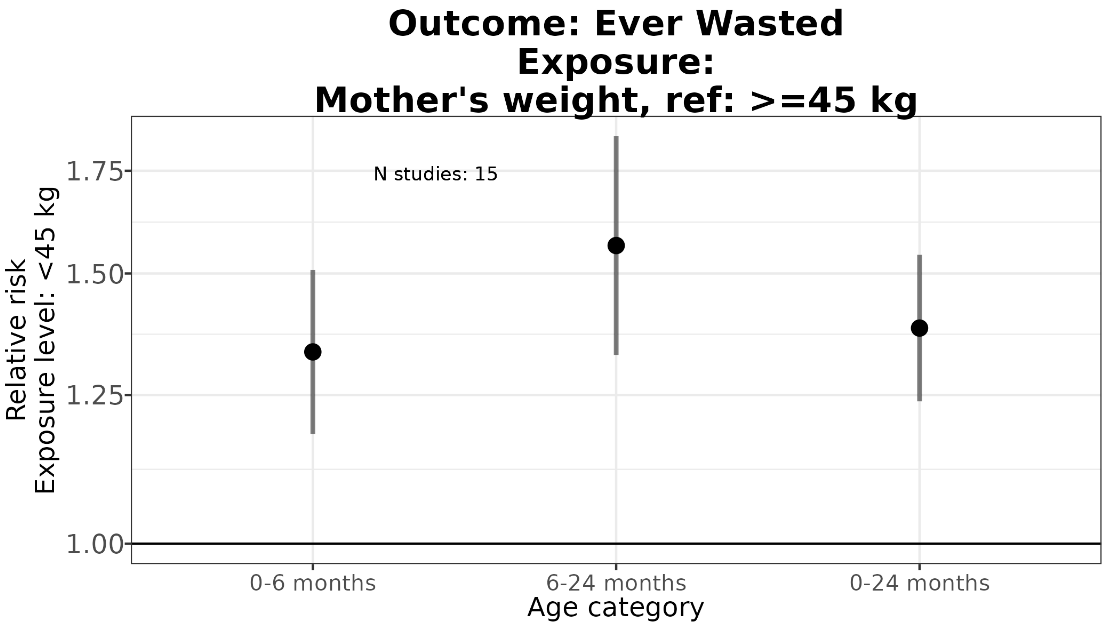
Outcome: Ever Wasted
Exposure:
Mother's weight, ref: >=45 kg
N studies: 15
Relative risk
Exposure level: <45 kg
1.75
1.50
1.25
1.00
0-6 months
6-24 months
0-24 months
Age category

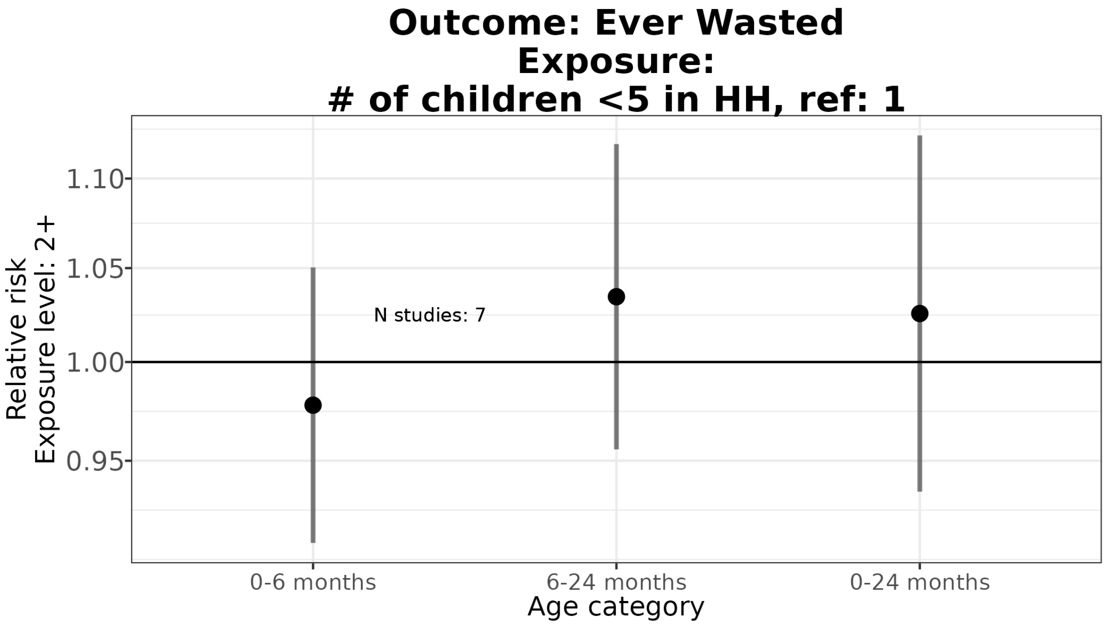
Outcome: Ever Wasted
Exposure:
# of children <5 in HH, ref: 1
N studies: 7
Relative risk
Exposure level: 2+
1.10
1.05
1.00
0.95
0-6 months
6-24 months
0-24 months
Age category

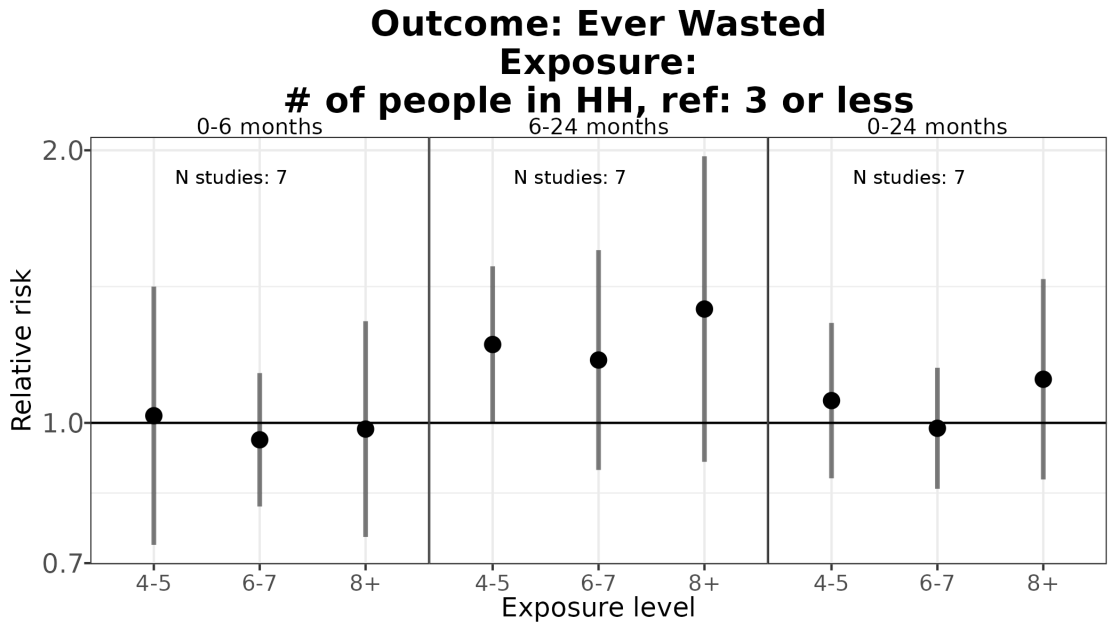
Outcome: Ever Wasted
Exposure:
# of people in HH, ref: 3 or less
0-6 months
6-24 months
0-24 months
N studies: 7
N studies: 7
N studies: 7
Relative risk
2.0
1.0
0.7
4-5
6-7
8+
Exposure level

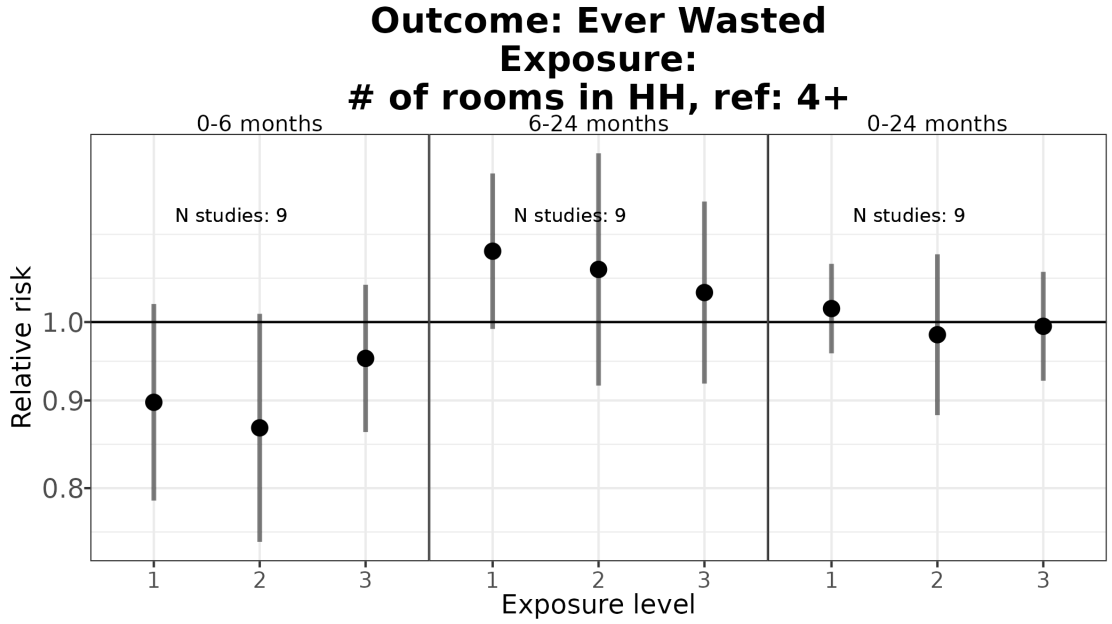
Outcome: Ever Wasted
Exposure:
# of rooms in HH, ref: 4+
0-6 months
6-24 months
0-24 months
N studies: 9
N studies: 9
N studies: 9
Relative risk
1.0
0.9
0.8
1
2
3
Exposure level

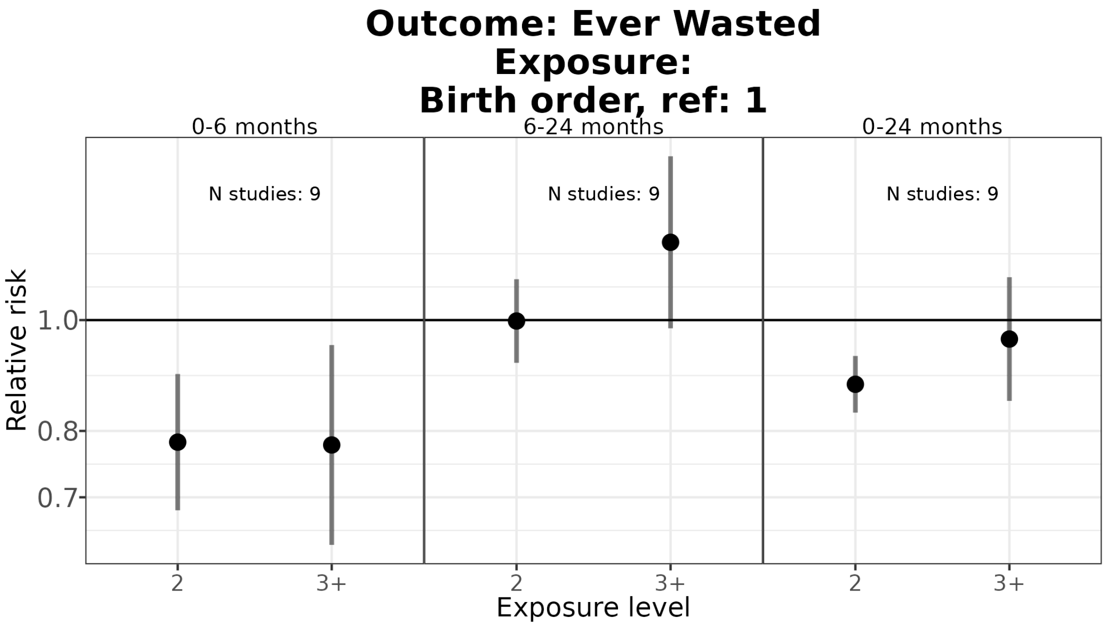
Outcome: Ever Wasted
Exposure:
Birth order, ref: 1
0-6 months
6-24 months
0-24 months
N studies: 9
N studies: 9
N studies: 9
Relative risk
1.0
0.8
0.7
2
3+
2
3+
2
3+
Exposure level

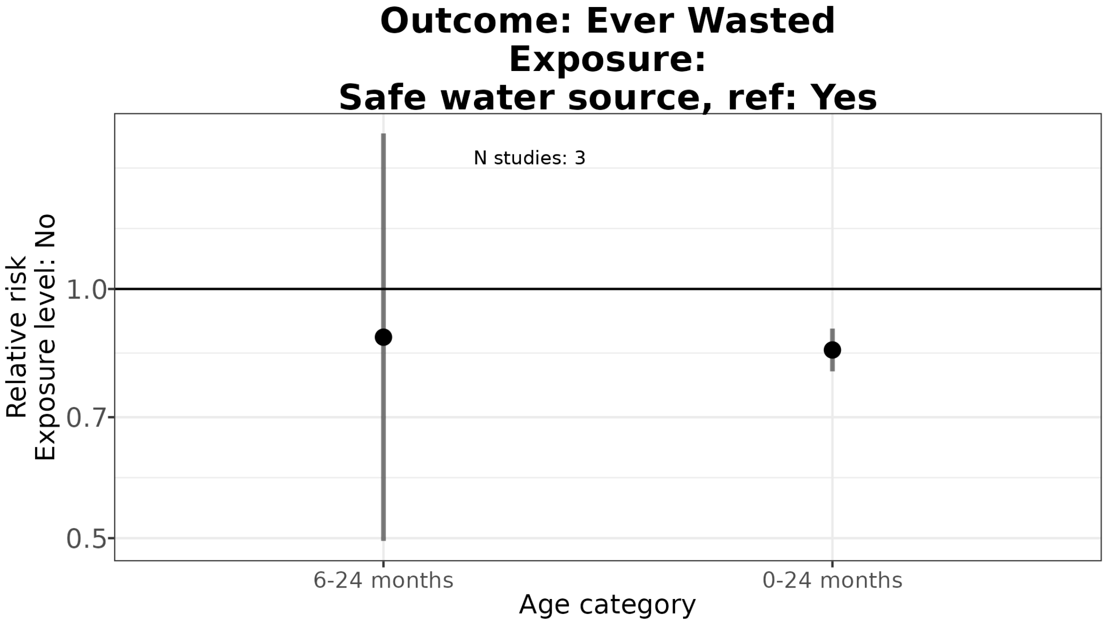
Outcome: Ever Wasted
Exposure:
Safe water source, ref: Yes
N studies: 3
Relative risk
Exposure level: No
1.0
0.7
0.5
6-24 months
0-24 months
Age category

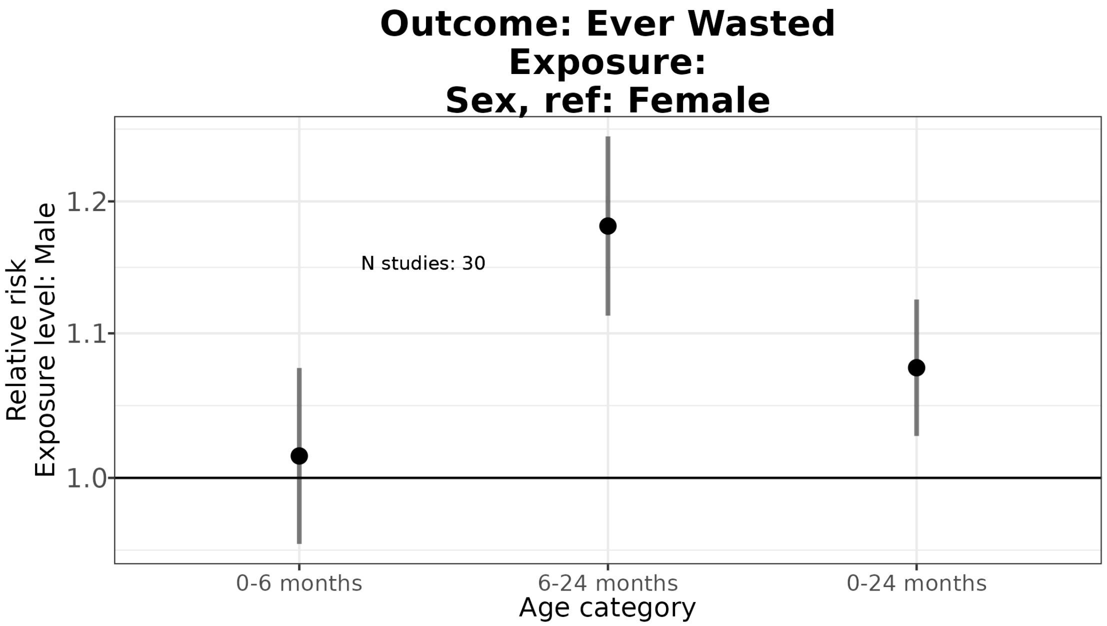
Outcome: Ever Wasted
Exposure:
Sex, ref: Female
Relative risk
Exposure level: Male
1.2
1.1
1.0
N studies: 30
0-6 months
6-24 months
0-24 months
Age category

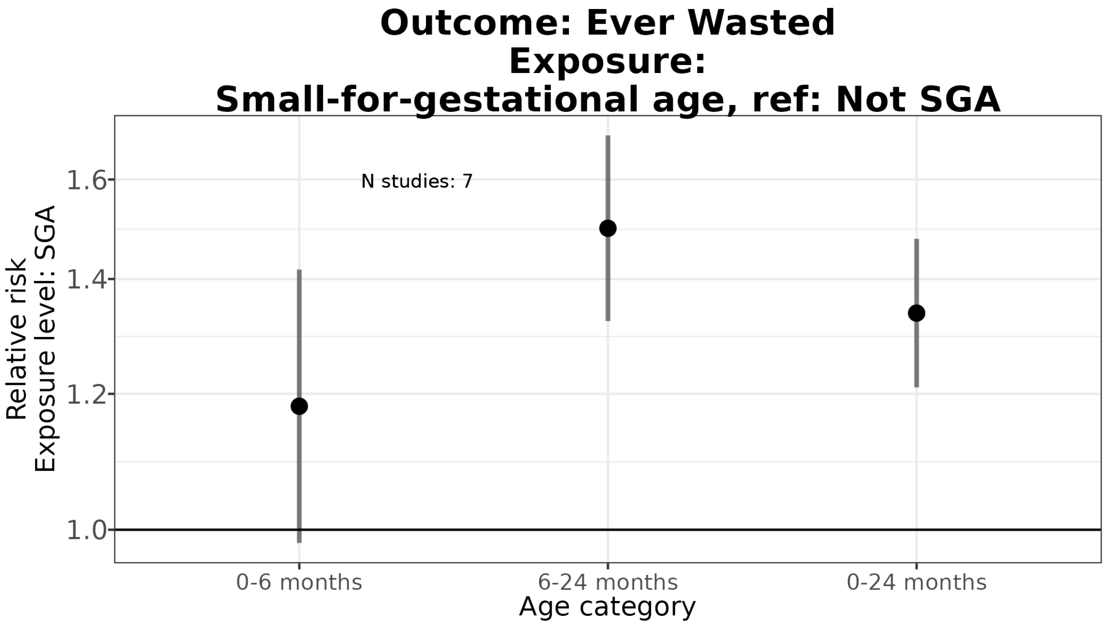
Outcome: Ever Wasted
Exposure:
Small-for-gestational age, ref: Not SGA
Relative risk
Exposure level: SGA
1.6
1.4
1.2
1.0
N studies: 7
0-6 months
6-24 months
0-24 months
Age category

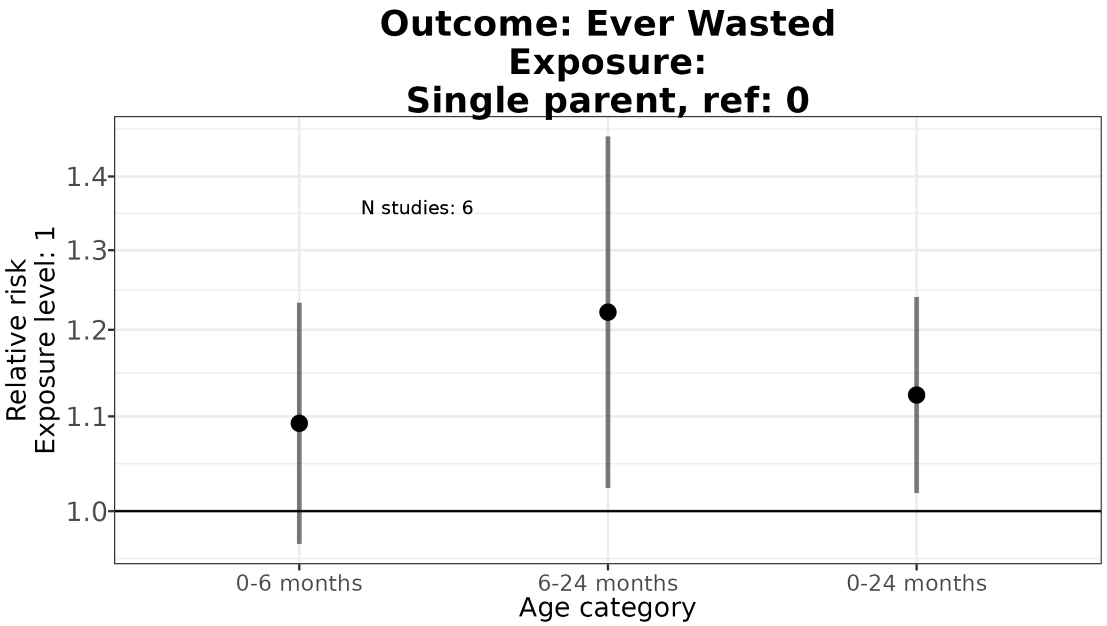
Outcome: Ever Wasted
Exposure:
Single parent, ref: 0
N studies: 6
Relative risk
Exposure level: 1
1.4
1.3
1.2
1.1
1.0
0-6 months
6-24 months
0-24 months
Age category

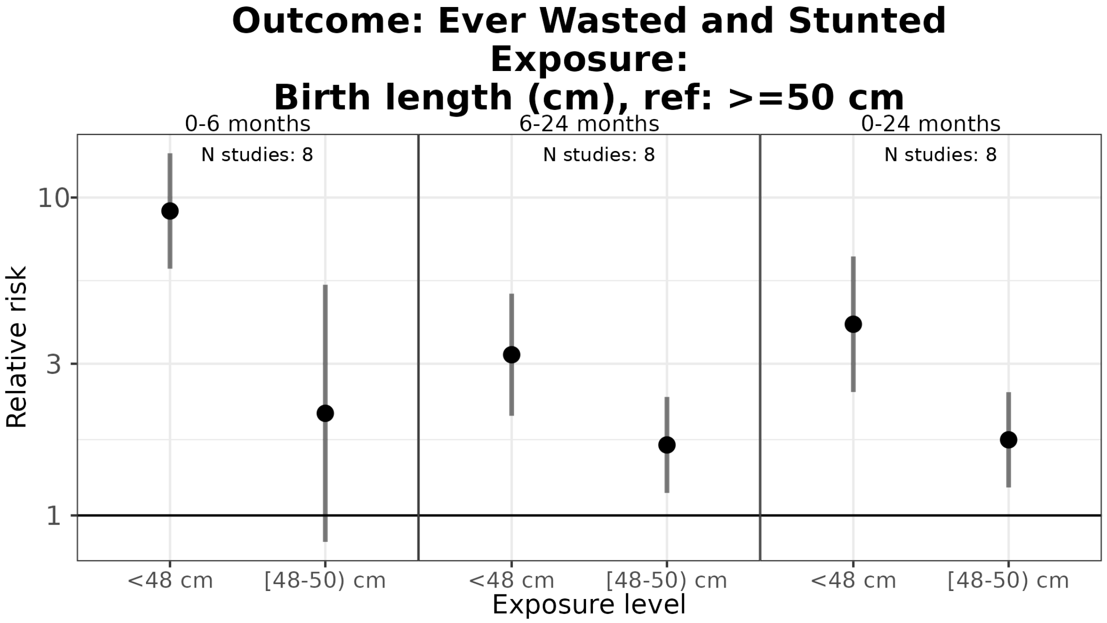
Outcome: Ever Wasted and Stunted
Exposure:
Birth length (cm), ref: >=50 cm
0-6 months
6-24 months
0-24 months
N studies: 8
N studies: 8
N studies: 8
Relative risk
10
3
1
<48 cm
[48-50) cm
<48 cm
[48-50) cm
<48 cm
[48-50) cm
Exposure level

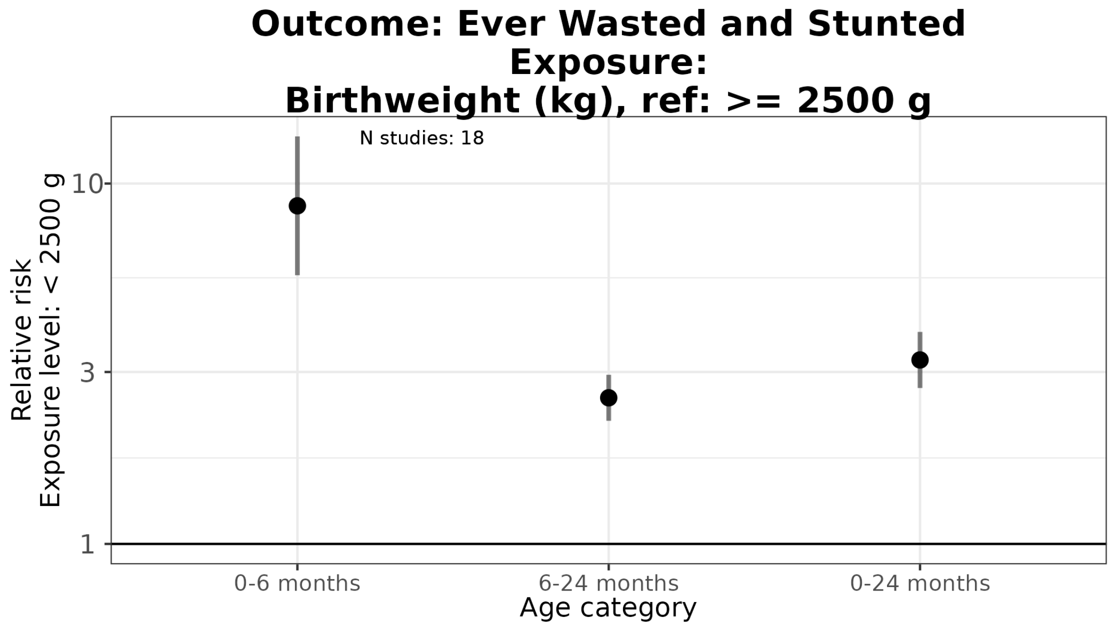
Outcome: Ever Wasted and Stunted
Exposure:
Birthweight (kg), ref: >= 2500 g
N studies: 18
Relative risk
Exposure level: < 2500 g
10
3
1
0-6 months
6-24 months
0-24 months
Age category

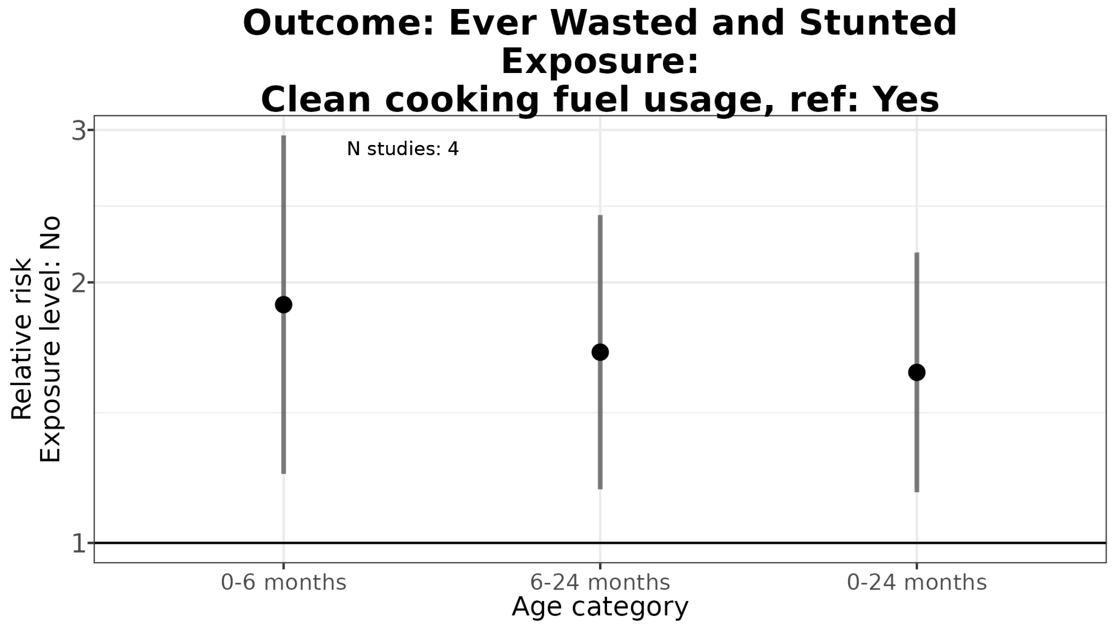
Outcome: Ever Wasted and Stunted
Exposure:
Clean cooking fuel usage, ref: Yes
N studies: 4
Relative risk
Exposure level: No
3
2
1
0-6 months
6-24 months
0-24 months
Age category

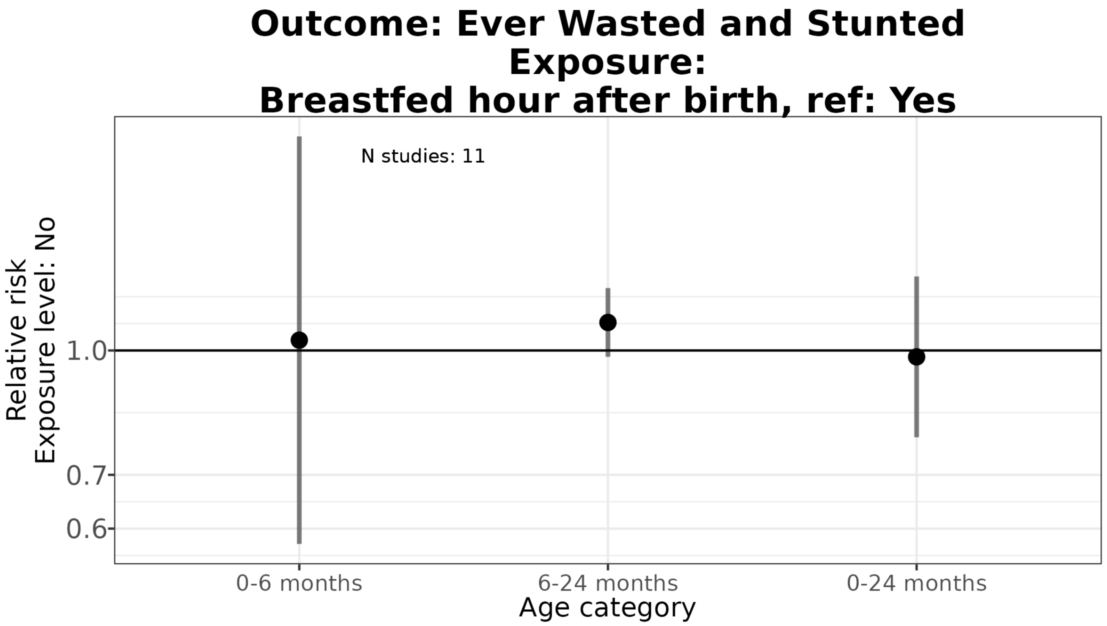
Outcome: Ever Wasted and Stunted
Exposure:
Breastfed hour after birth, ref: Yes
N studies: 11
Relative risk
Exposure level: No
1.0
0.7
0.6
0-6 months
6-24 months
0-24 months
Age category

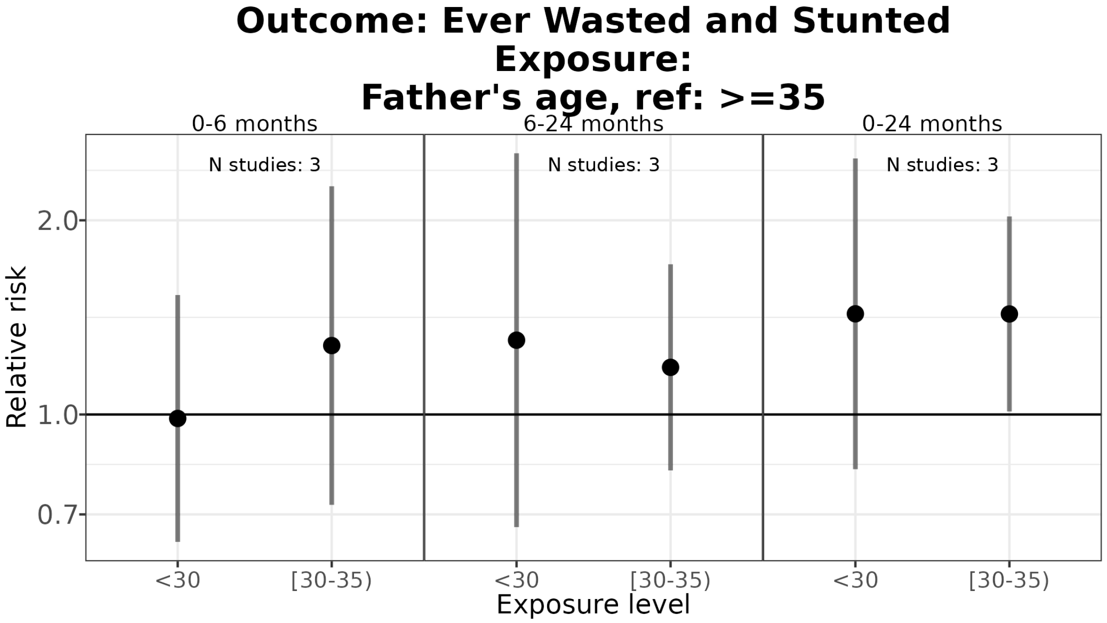
Outcome: Ever Wasted and Stunted
Exposure:
Father's age, ref: >=35
0-6 months
6-24 months
0-24 months
N studies: 3
N studies: 3
N studies: 3
Relative risk
2.0
1.0
0.7
<30
[30-35)
<30
[30-35)
<30
[30-35)
Exposure level

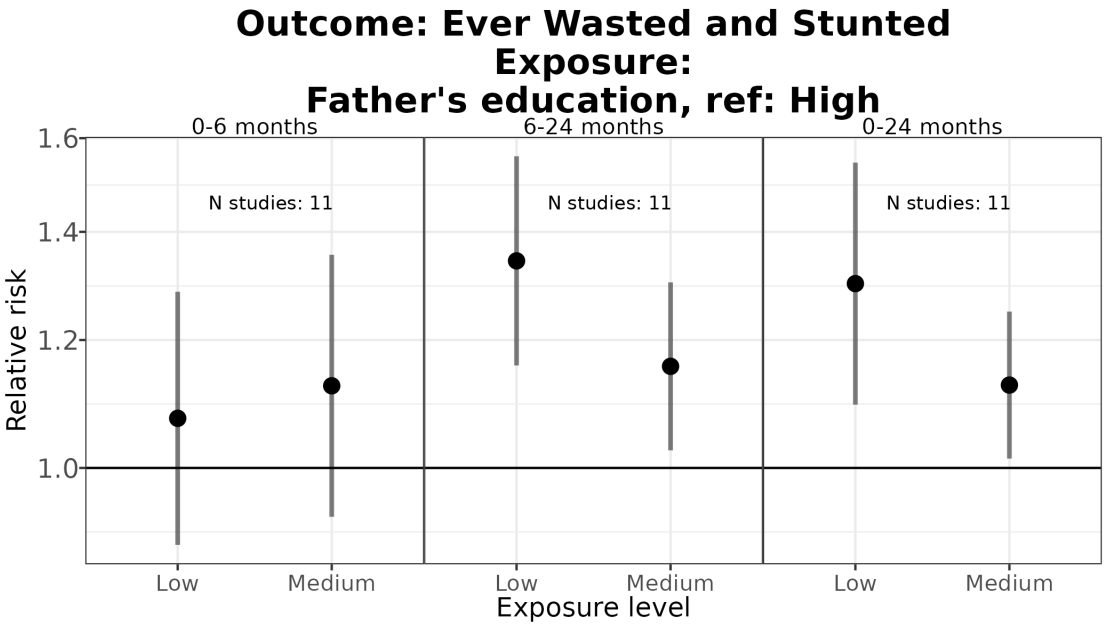

Outcome: Ever Wasted and Stunted
Exposure:
Father's education, ref: High
0-6 months
6-24 months
0-24 months
N studies: 11
N studies: 11
N studies: 11
Relative risk
1.6
1.4
1.2
1.0
Low
Medium
Low
Medium
Low
Medium
Exposure level


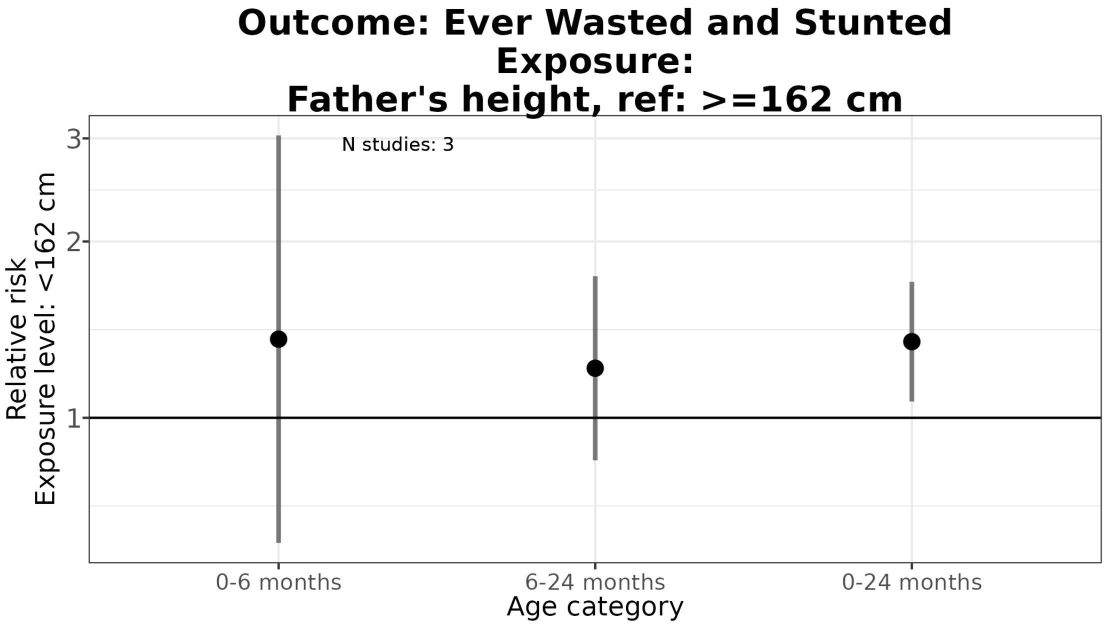

Outcome: Ever Wasted and Stunted
Exposure:
Father's height, ref: >=162 cm
N studies: 3
Relative risk
Exposure level: <162 cm
3
2
1
0-6 months
6-24 months
0-24 months
Age category

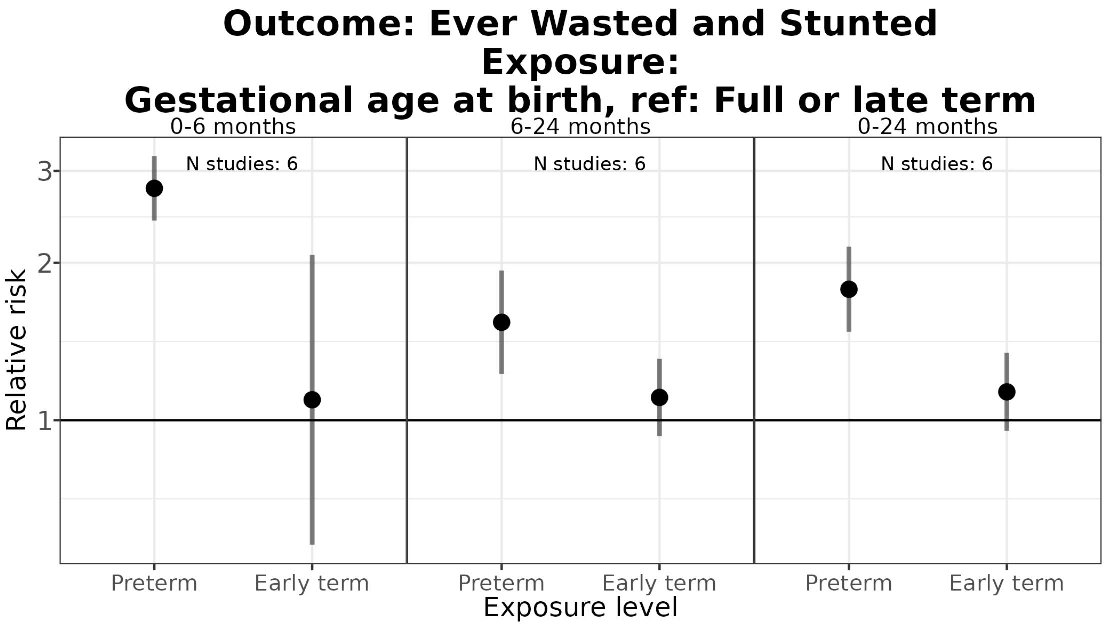
Outcome: Ever Wasted and Stunted
Exposure:
Gestational age at birth, ref: Full or late term
0-6 months
6-24 months
0-24 months
N studies: 6
N studies: 6
N studies: 6
Relative risk
3
2
1
Preterm
Early term
Preterm
Early term
Preterm
Early term
Exposure level

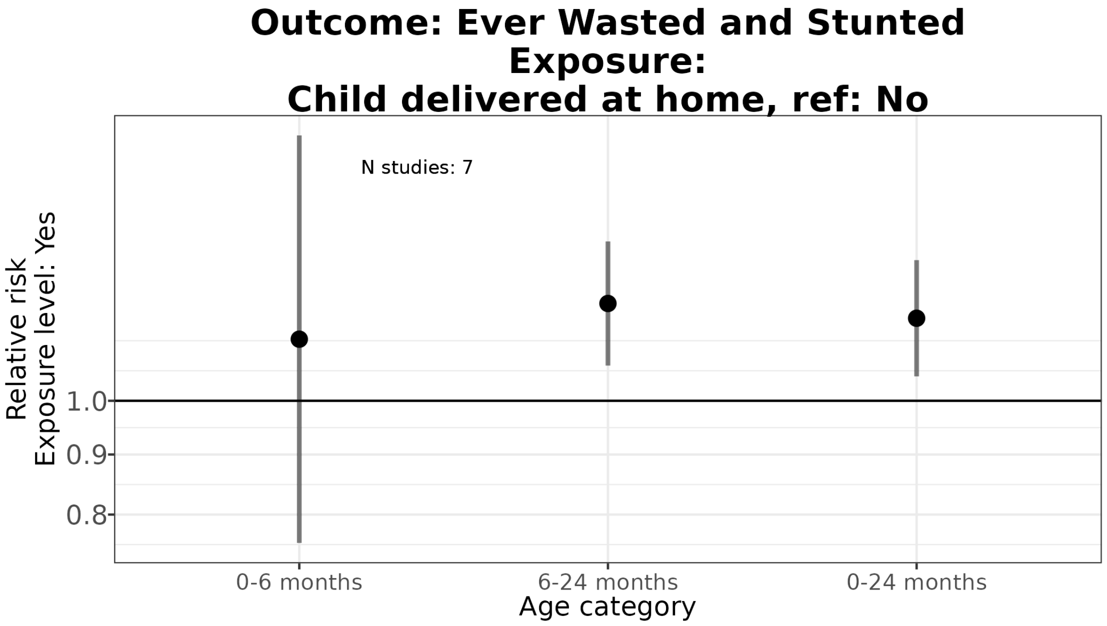
Outcome: Ever Wasted and Stunted
Exposure:
Child delivered at home, ref: No
N studies: 7
Relative risk
Exposure level: Yes
1.0
0.9
0.8
0-6 months
6-24 months
0-24 months
Age category

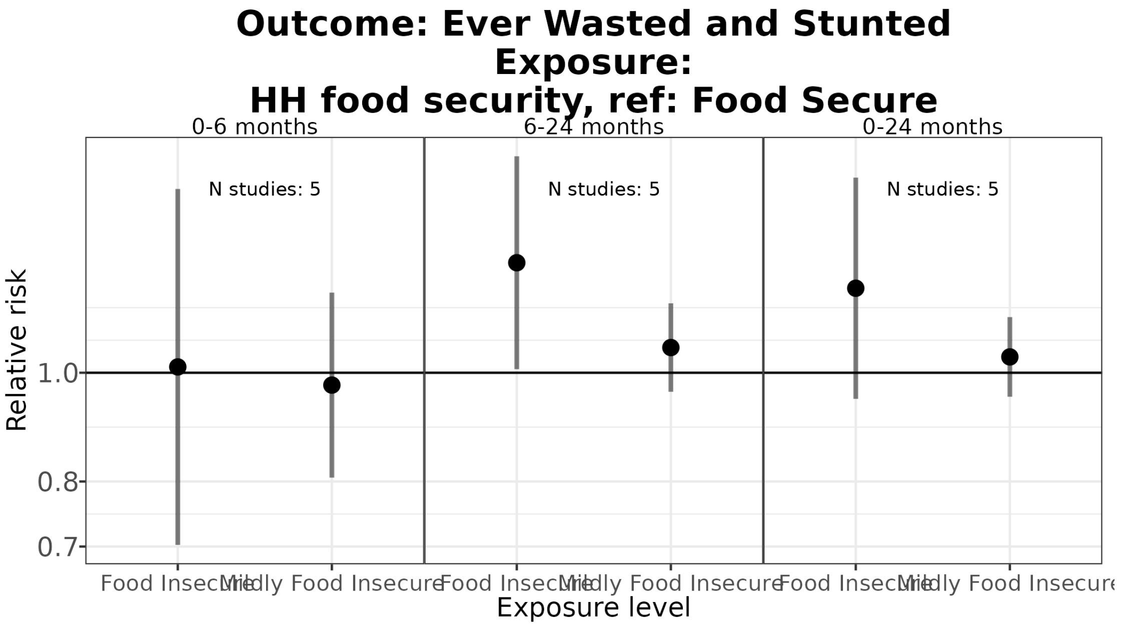
Outcome: Ever Wasted and Stunted
Exposure:
HH food security, ref: Food Secure
0-6 months
6-24 months
0-24 months
N studies: 5
N studies: 5
N studies: 5
Relative risk
1.0
0.8
0.7
Food Insecure
Mildly Food Insecure
Food Insecure
Mildly Food Insecure
Food Insecure
Mildly Food Insecure
Exposure level

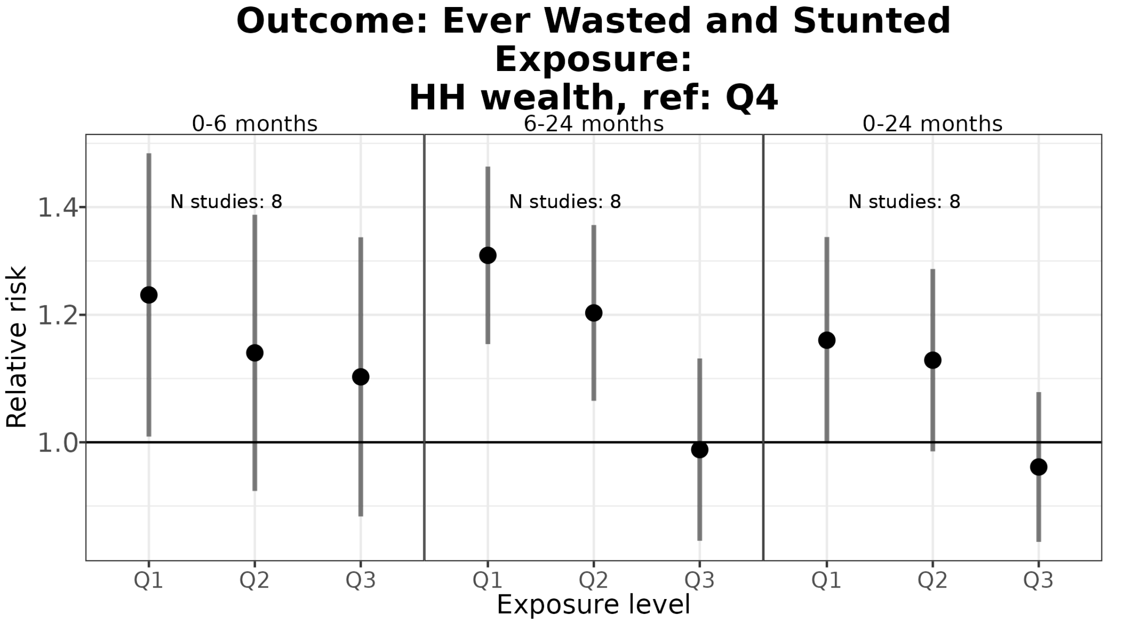
Outcome: Ever Wasted and Stunted
Exposure:
HH wealth, ref: Q4
0-6 months
6-24 months
0-24 months
N studies: 8
N studies: 8
N studies: 8
Relative risk
1.4
1.2
1.0
Q1
Q2
Q3
Q1
Q2
Q3
Q1
Q2
Q3
Exposure level

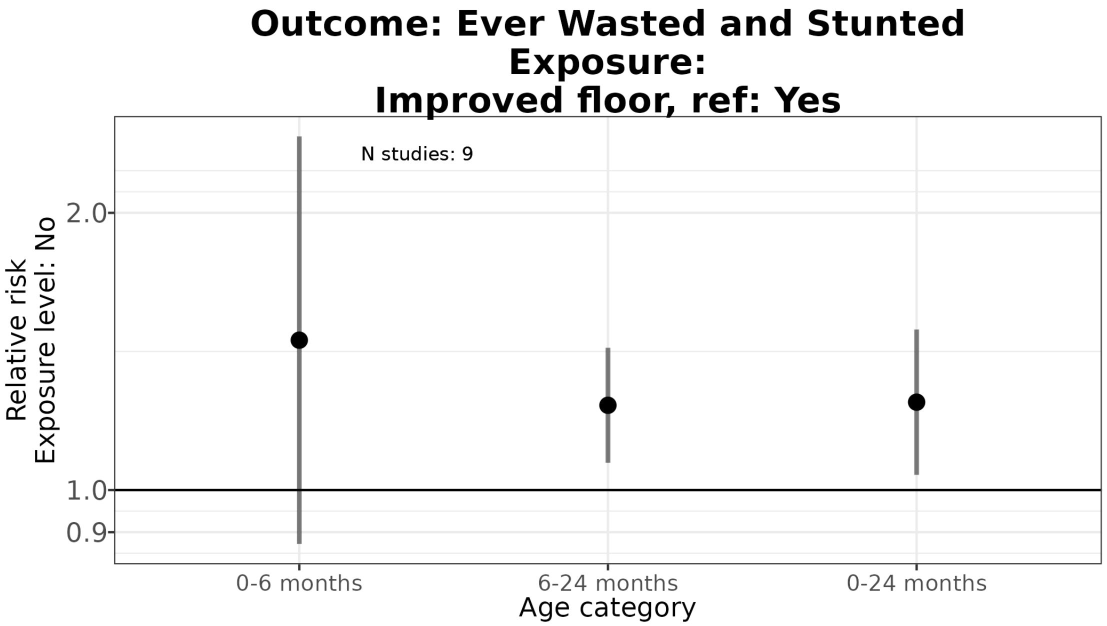

Outcome: Ever Wasted and Stunted
Exposure:
Improved floor, ref: Yes
N studies: 9
Relative risk
Exposure level: No
2.0
1.0
0.9
0-6 months
6-24 months
0-24 months
Age category


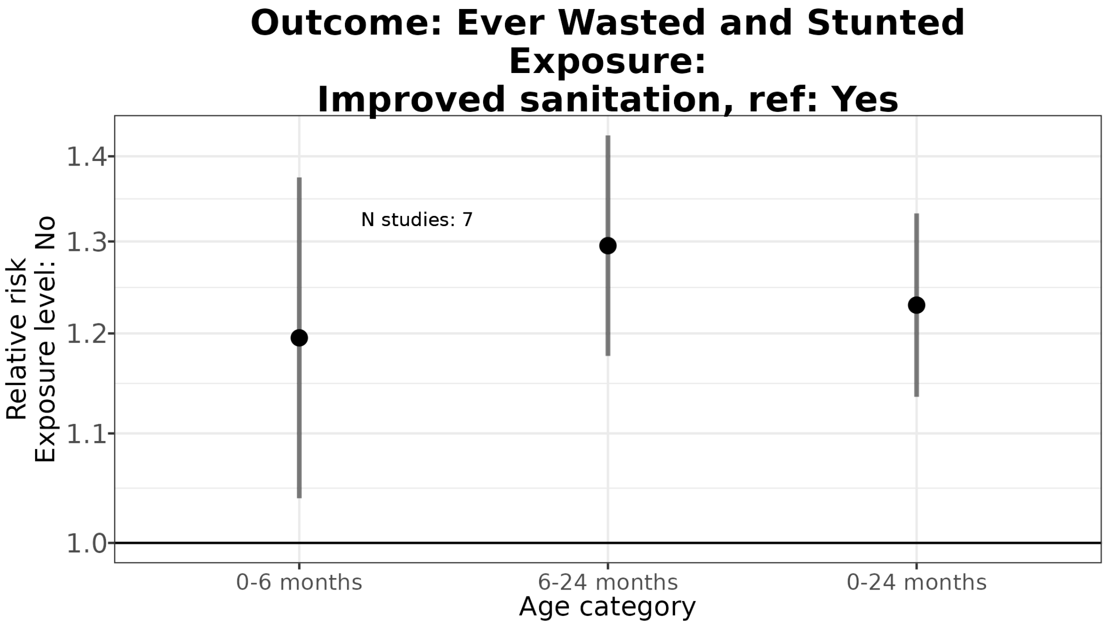

Outcome: Ever Wasted and Stunted
Exposure:
Improved sanitation, ref: Yes
N studies: 7
Relative risk
Exposure level: No
1.4
1.3
1.2
1.1
1.0
0-6 months
6-24 months
0-24 months
Age category

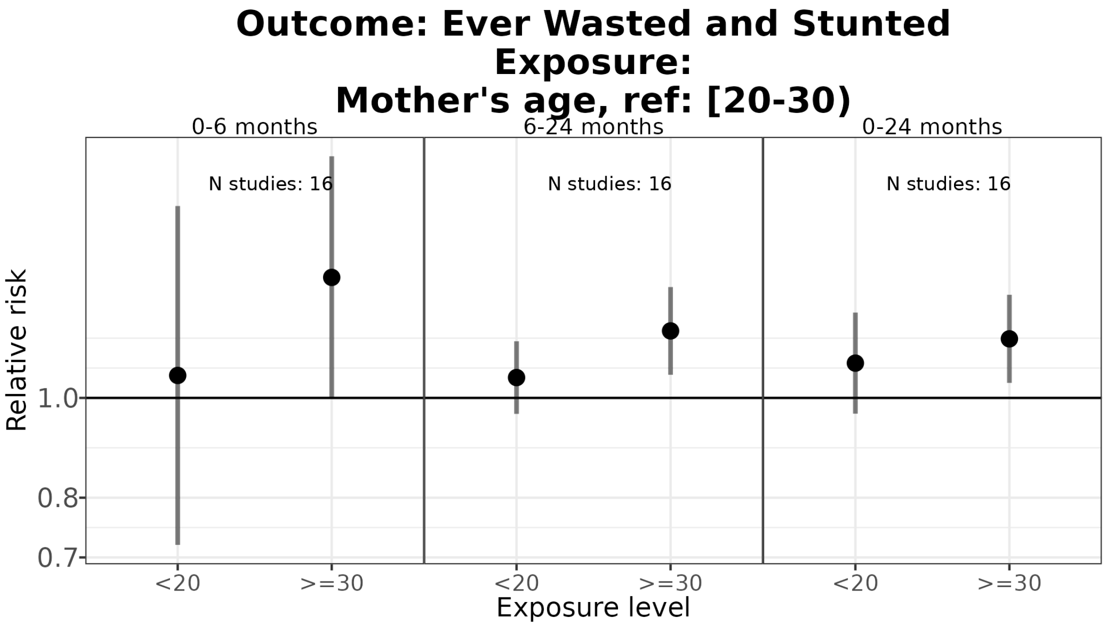
Outcome: Ever Wasted and Stunted
Exposure:
Mother's age, ref: [20-30)
0-6 months
6-24 months
0-24 months
N studies: 16
N studies: 16
N studies: 16
Relative risk
1.0
0.8
0.7
<20
>=30
<20
>=30
<20
>=30
Exposure level

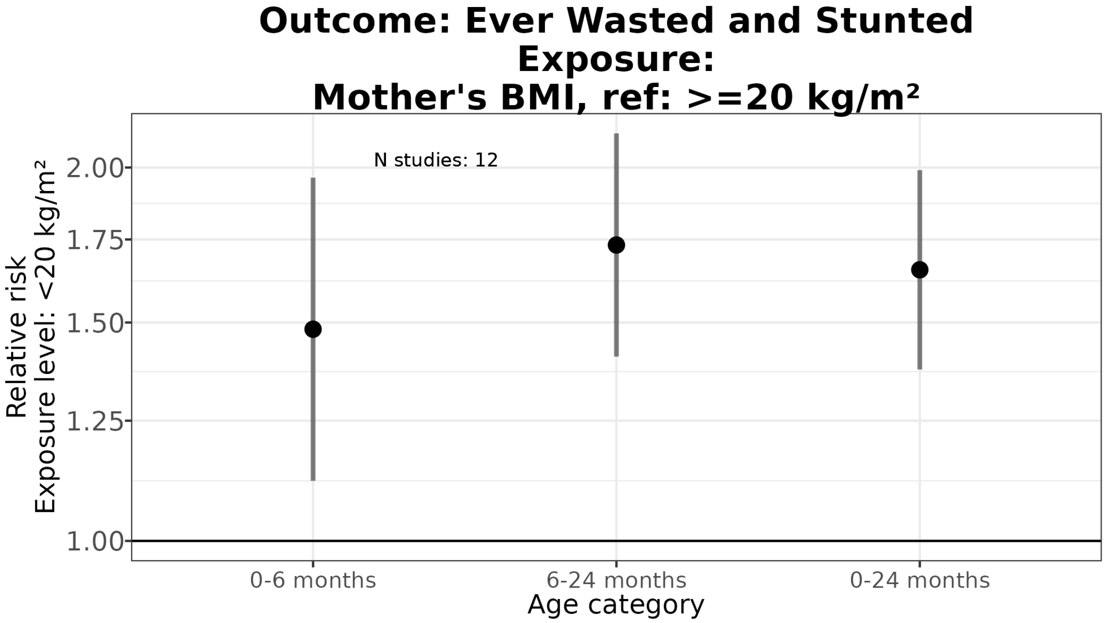
Outcome: Ever Wasted and Stunted
Exposure:
Mother's BMI, ref: >=20 kg/m²
N studies: 12
Relative risk
Exposure level: <20 kg/m²
2.00
1.75
1.50
1.25
1.00
0-6 months
6-24 months
0-24 months
Age category

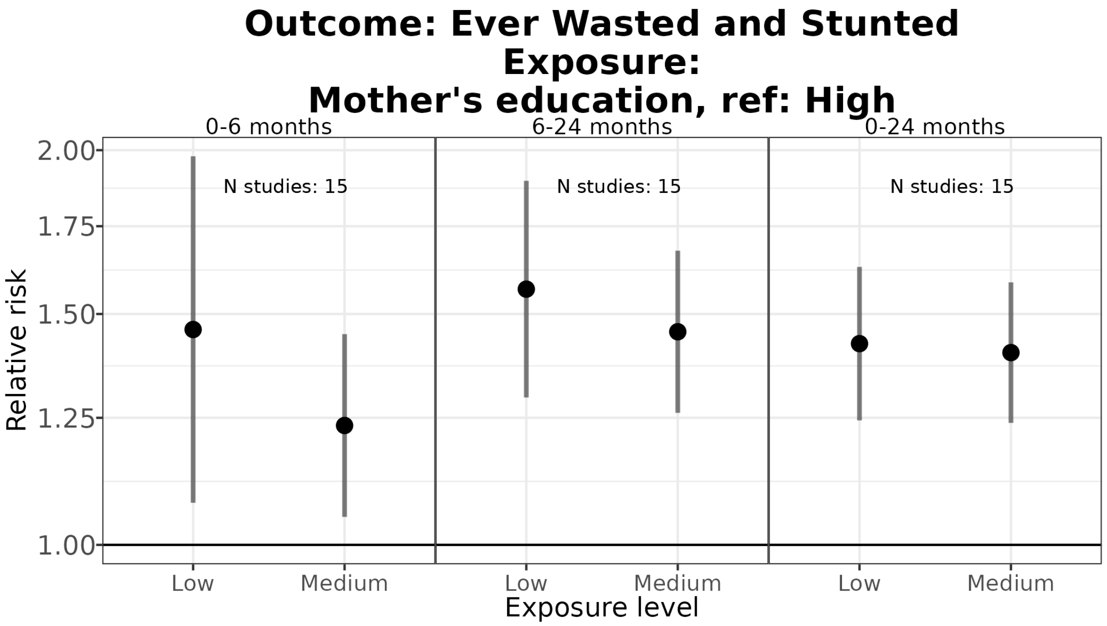
Outcome: Ever Wasted and Stunted
Exposure:
Mother's education, ref: High
0-6 months
6-24 months
0-24 months
N studies: 15
N studies: 15
N studies: 15
2.00
1.75
1.50
1.25
1.00
Relative risk
Low
Medium
Low
Medium
Low
Medium
Exposure level

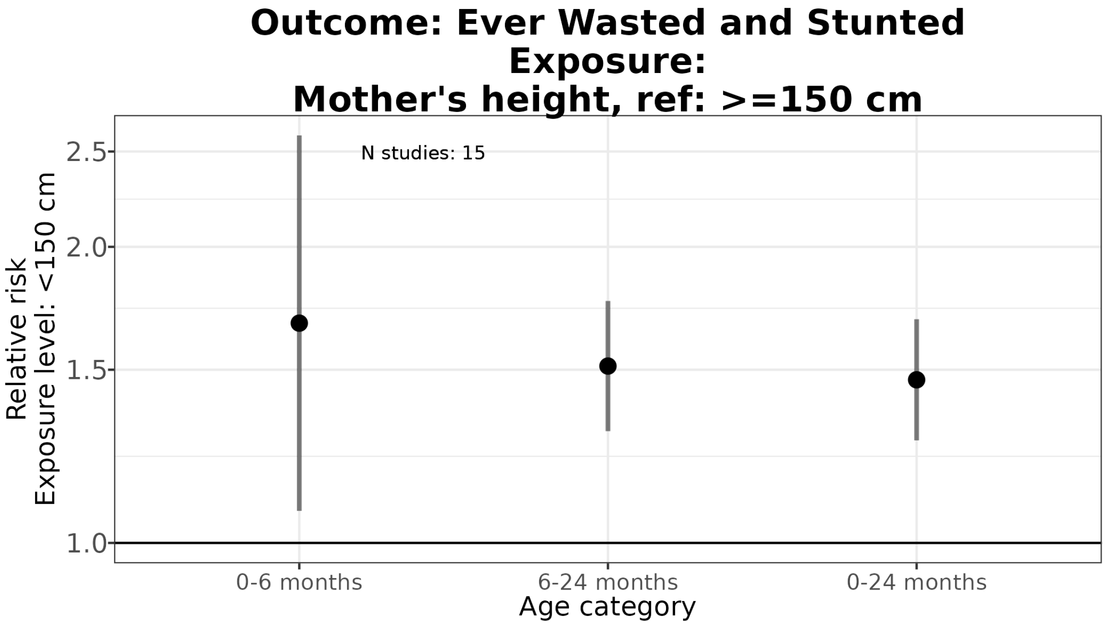
Outcome: Ever Wasted and Stunted
Exposure:
Mother's height, ref: >=150 cm
N studies: 15
2.5
2.0
1.5
1.0
Relative risk
Exposure level: <150 cm
0-6 months
6-24 months
0-24 months
Age category

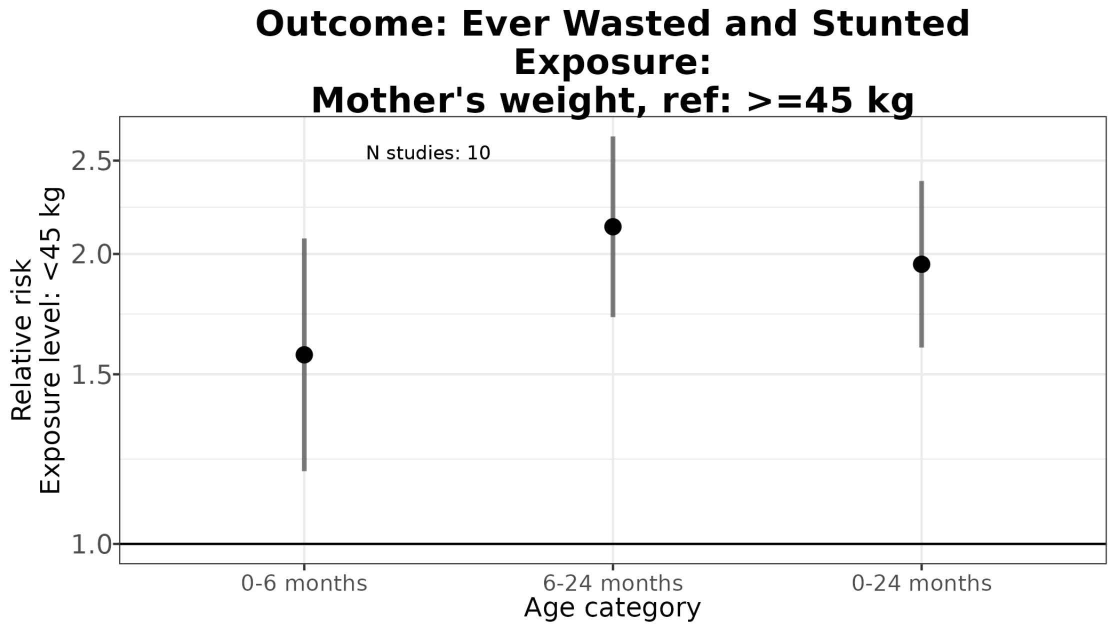
Outcome: Ever Wasted and Stunted
Exposure:
Mother's weight, ref: >=45 kg
N studies: 10
Relative risk
Exposure level: <45 kg
2.5
2.0
1.5
1.0
0-6 months
6-24 months
0-24 months
Age category

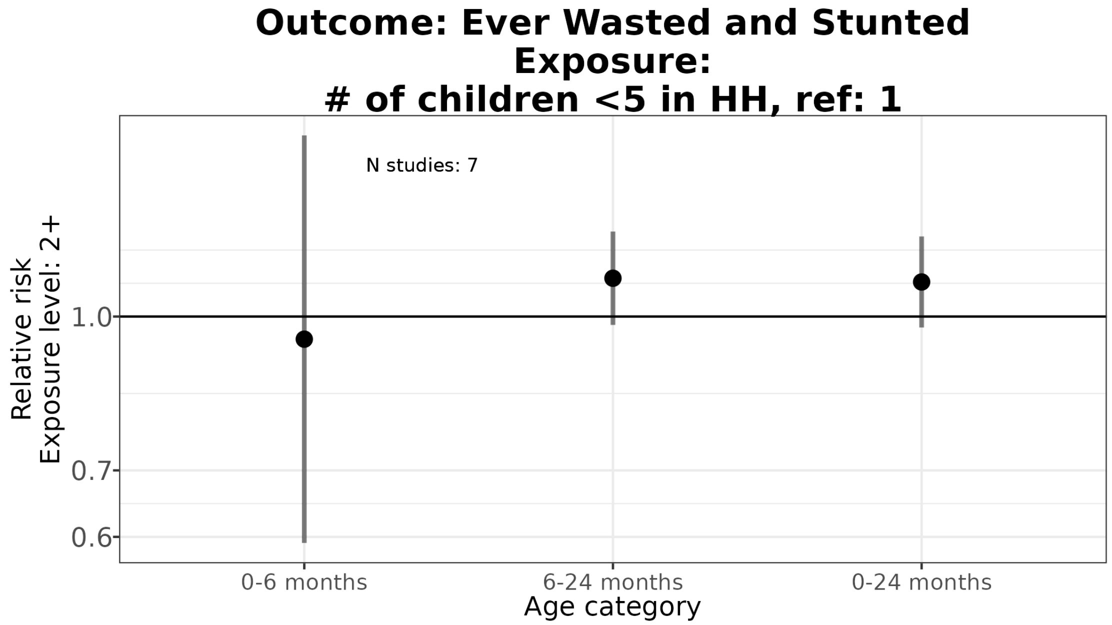
Outcome: Ever Wasted and Stunted
Exposure:
# of children <5 in HH, ref: 1
N studies: 7
Relative risk
Exposure level: 2+
1.0
0.7
0.6
0-6 months
6-24 months
0-24 months
Age category

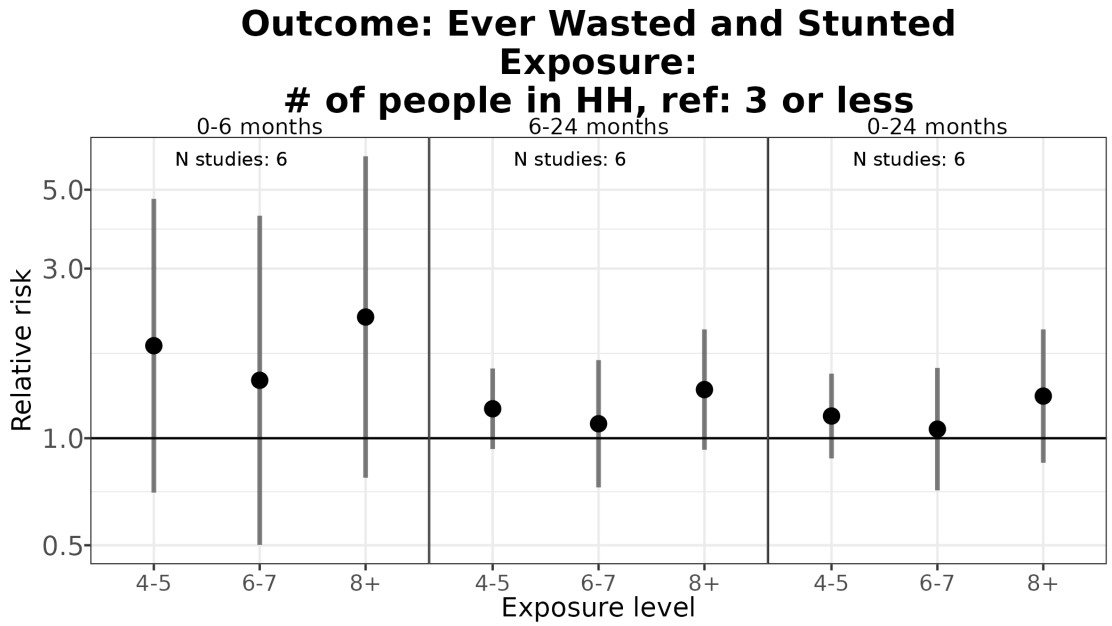
Outcome: Ever Wasted and Stunted
Exposure:
# of people in HH, ref: 3 or less
0-6 months
6-24 months
0-24 months
N studies: 6
N studies: 6
N studies: 6
5.0
3.0
1.0
0.5
Relative risk
4-5
6-7
8+
4-5
6-7
8+
4-5
6-7
8+
Exposure level

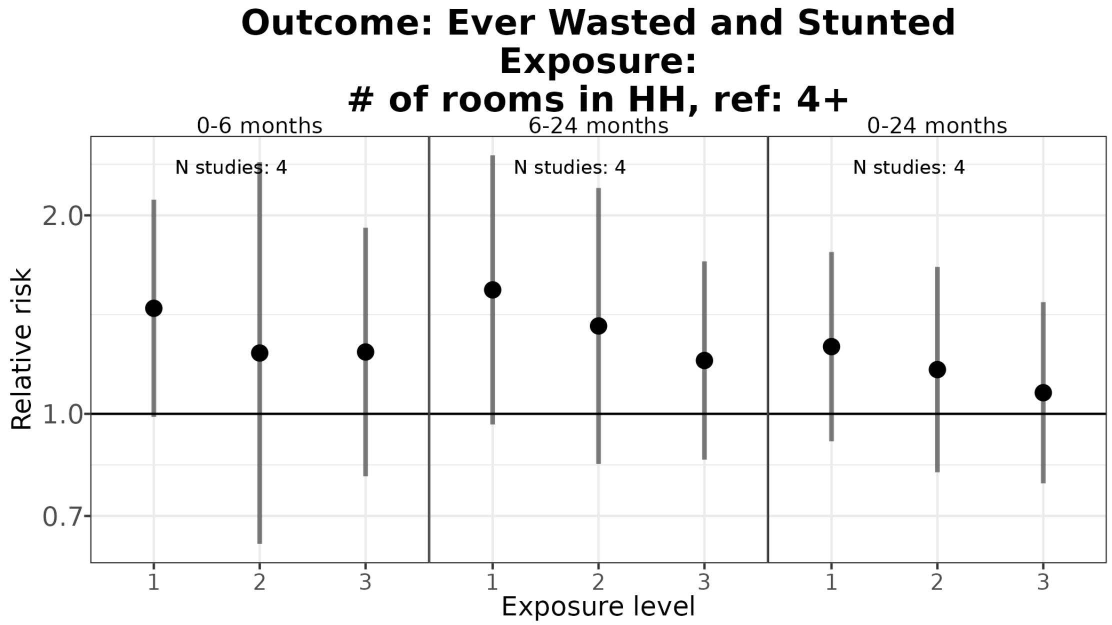
Outcome: Ever Wasted and Stunted
Exposure:
# of rooms in HH, ref: 4+
0-6 months
6-24 months
0-24 months
N studies: 4
N studies: 4
N studies: 4
2.0
1.0
0.7
Relative risk
1
2
3
1
2
3
1
2
3
Exposure level

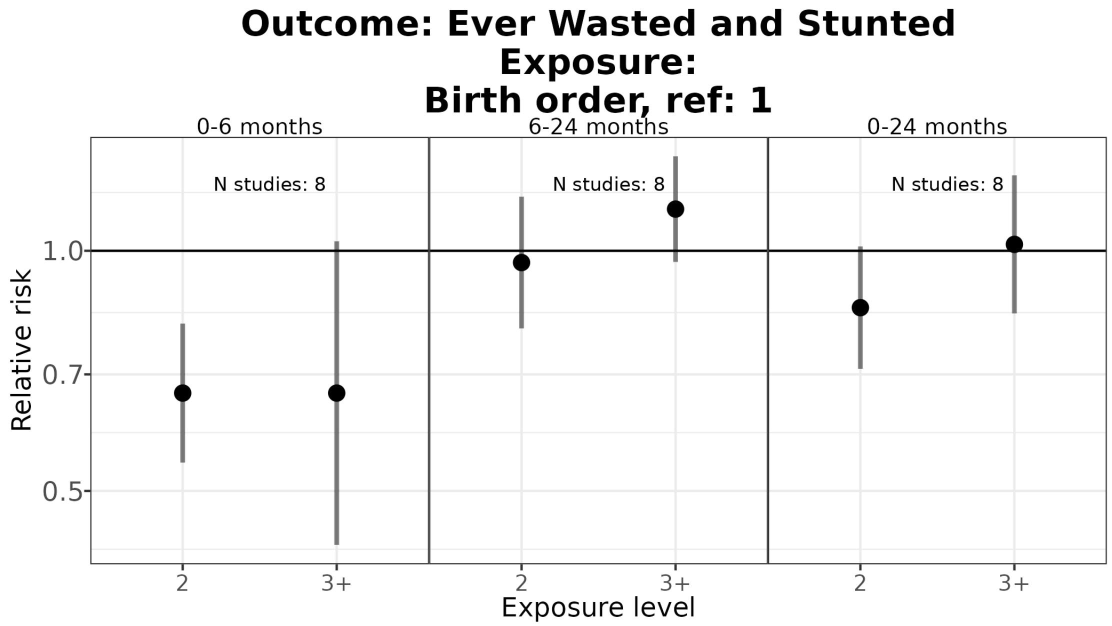
Outcome: Ever Wasted and Stunted
Exposure:
Birth order, ref: 1
0-6 months
6-24 months
0-24 months
N studies: 8
N studies: 8
N studies: 8
Relative risk
1.0
0.7
0.5
2
3+
2
3+
2
3+
Exposure level

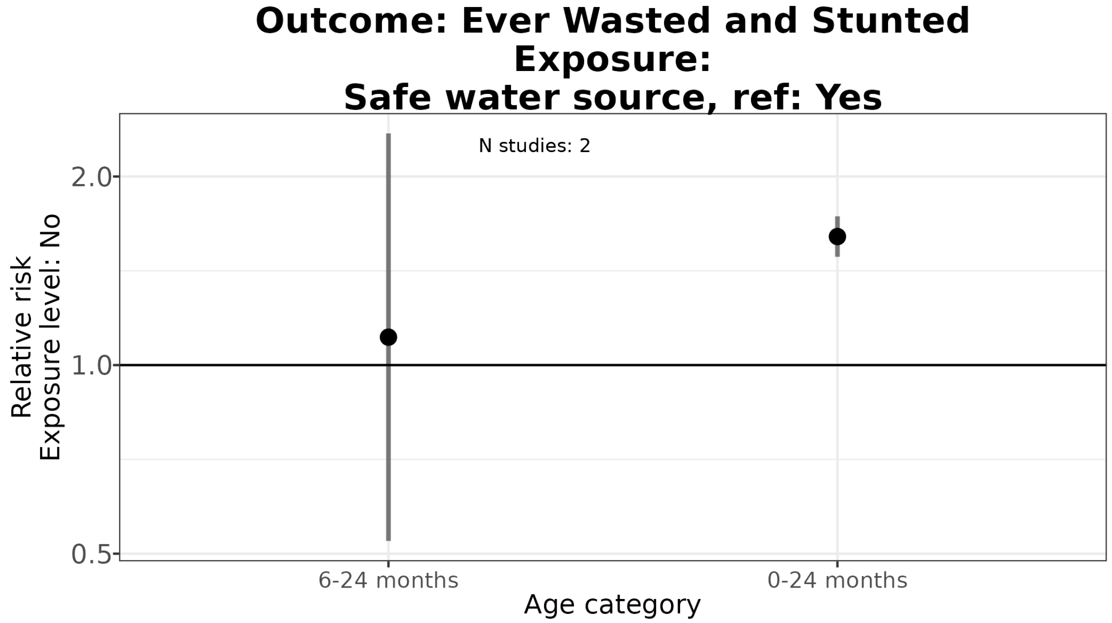
Outcome: Ever Wasted and Stunted
Exposure:
Safe water source, ref: Yes
N studies: 2
Relative risk
Exposure level: No
2.0
1.0
0.5
6-24 months
0-24 months
Age category

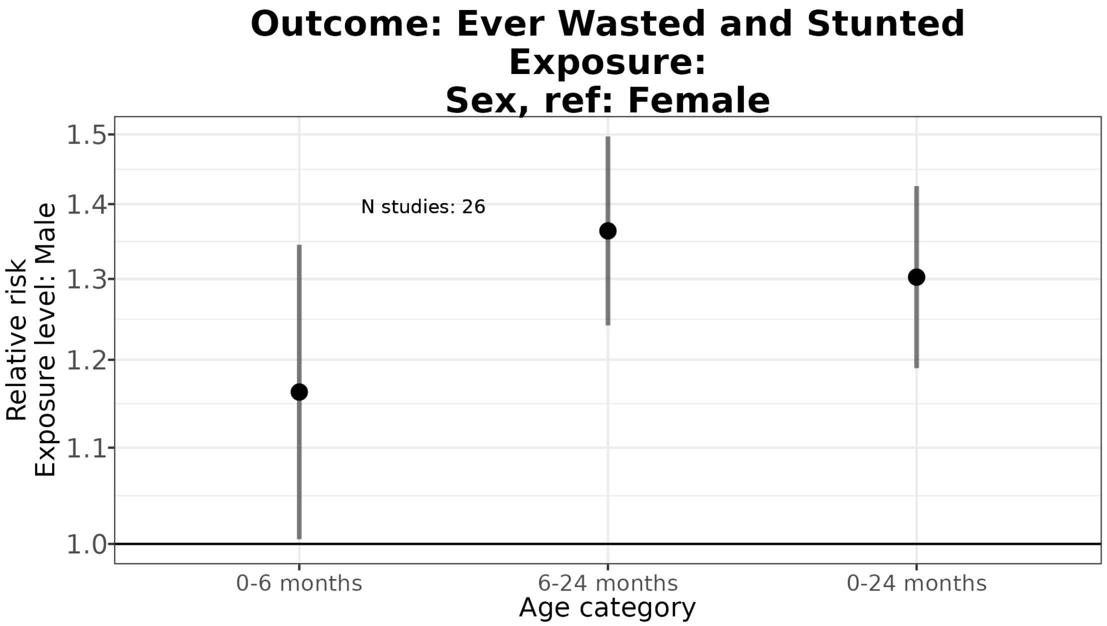
Outcome: Ever Wasted and Stunted
Exposure:
Sex, ref: Female
N studies: 26
Relative risk
Exposure level: Male
1.0
1.1
1.2
1.3
1.4
1.5
0-6 months
6-24 months
0-24 months
Age category

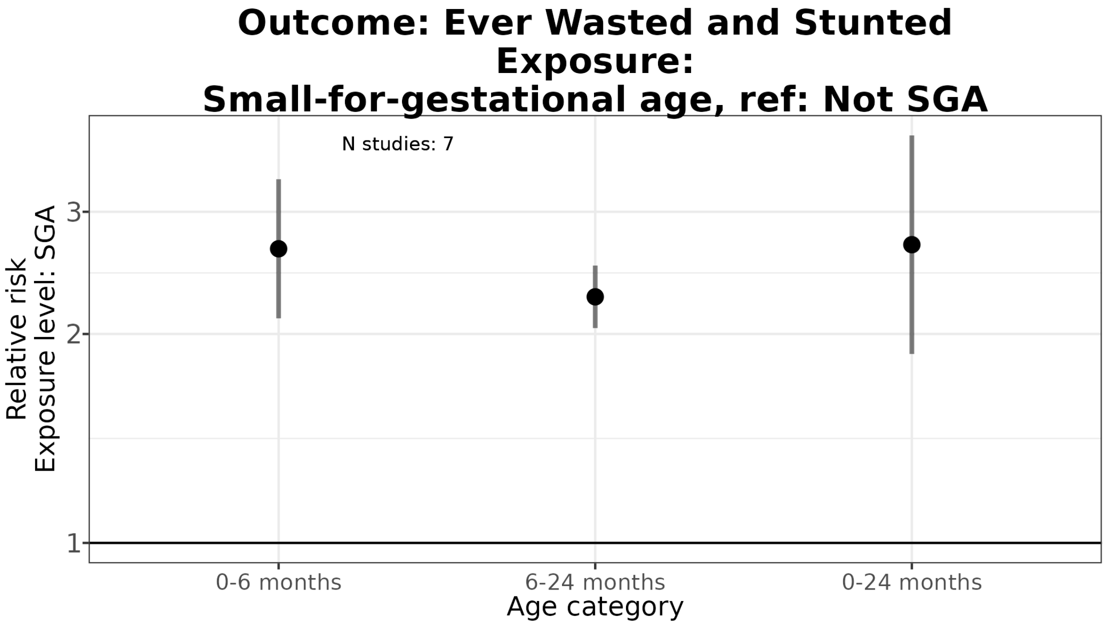
Outcome: Ever Wasted and Stunted
Exposure:
Small-for-gestational age, ref: Not SGA
N studies: 7
Relative risk
Exposure level: SGA
1
2
3
0-6 months
6-24 months
0-24 months
Age category

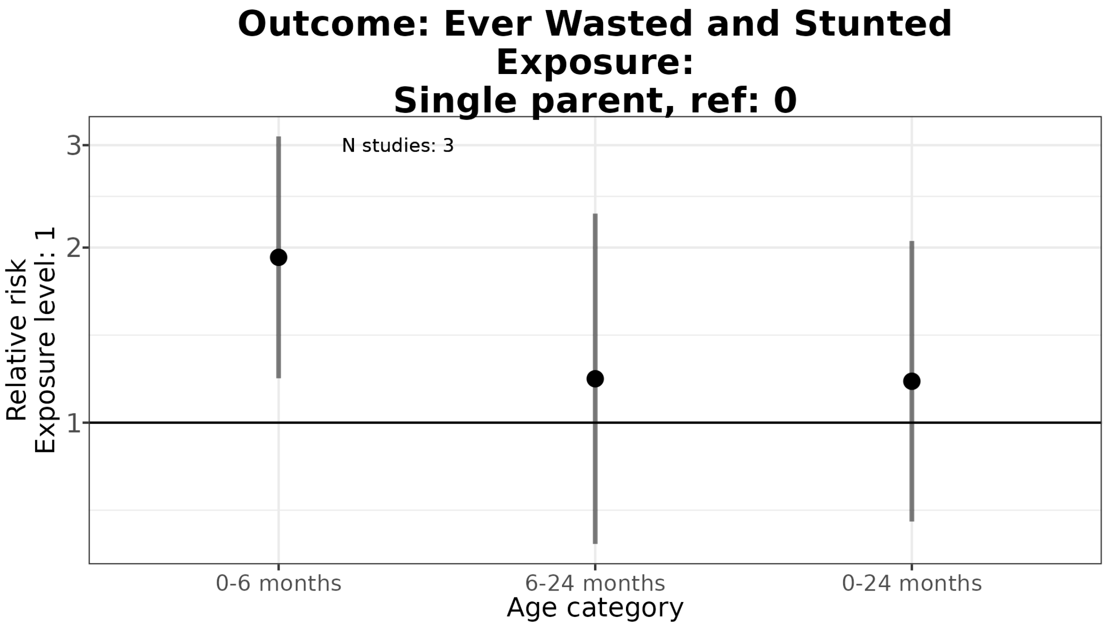

Outcome: Ever Wasted and Stunted
Exposure:
Single parent, ref: 0
N studies: 3
Relative risk
Exposure level: 1
3
2
1
0-6 months
6-24 months
0-24 months
Age category


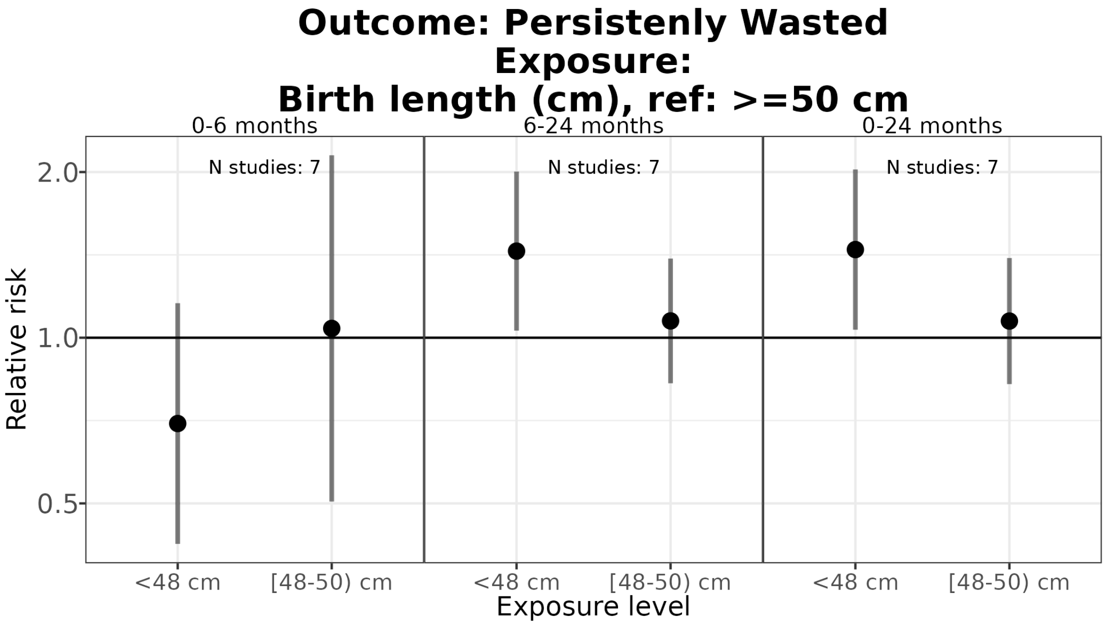

Outcome: Persistenly Wasted
Exposure:
Birth length (cm), ref: >=50 cm
0-6 months
6-24 months
0-24 months
N studies: 7
N studies: 7
N studies: 7
Relative risk
2.0
1.0
0.5
<48 cm
[48-50) cm
<48 cm
[48-50) cm
<48 cm
[48-50) cm
Exposure level

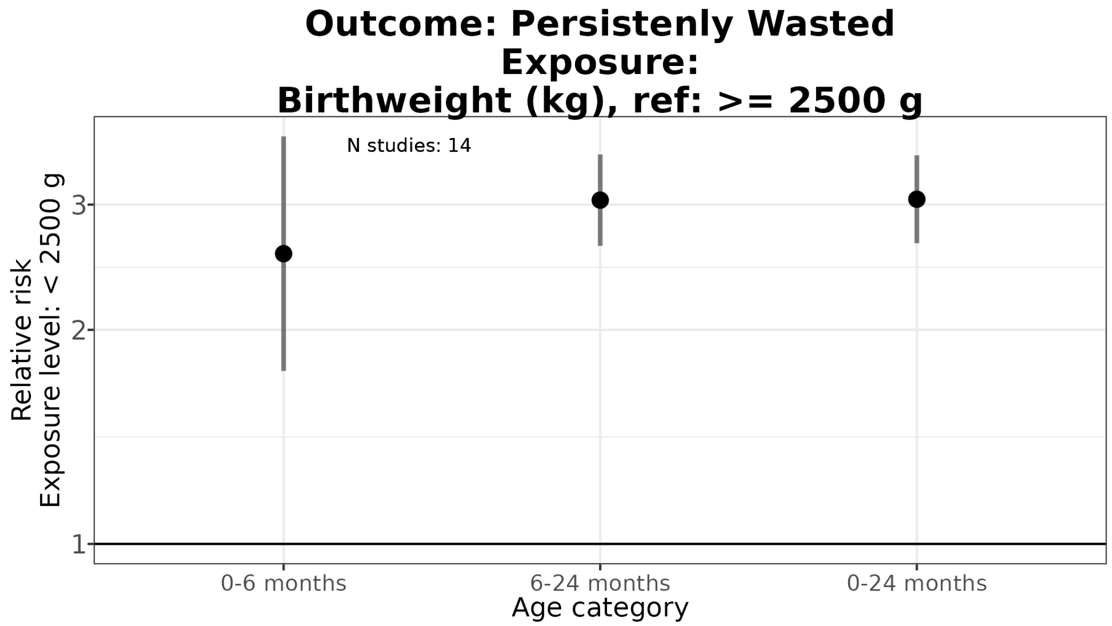
Outcome: Persistenly Wasted
Exposure:
Birthweight (kg), ref: >= 2500 g
N studies: 14
Relative risk
Exposure level: < 2500 g
3
2
1
0-6 months
6-24 months
0-24 months
Age category

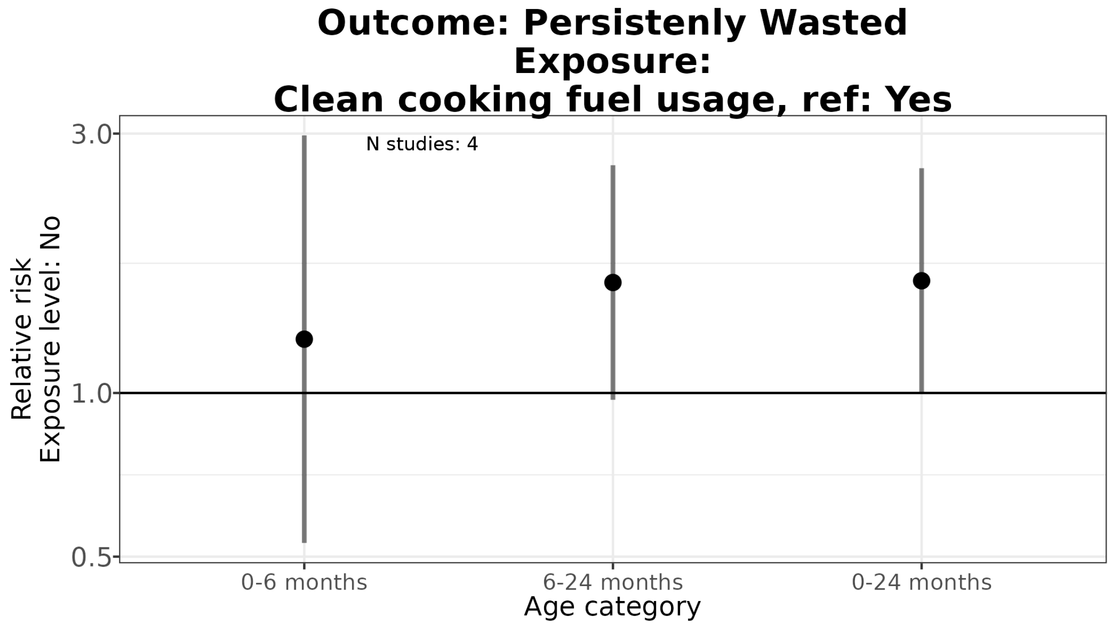
Outcome: Persistenly Wasted
Exposure:
Clean cooking fuel usage, ref: Yes
N studies: 4
Relative risk
Exposure level: No
3.0
1.0
0.5
0-6 months
6-24 months
0-24 months
Age category

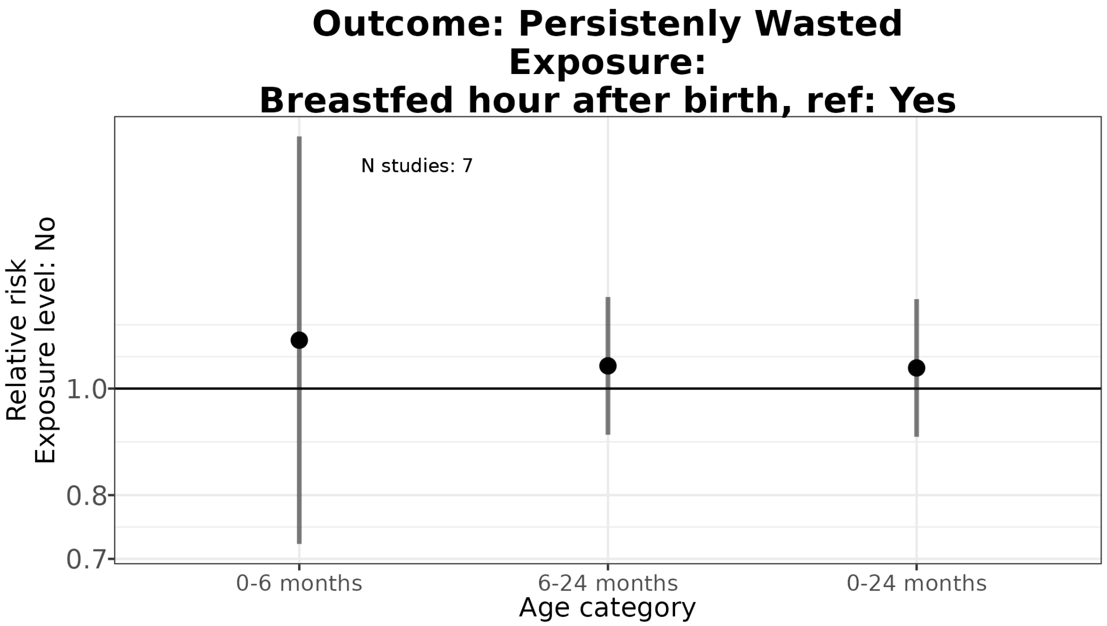
Outcome: Persistenly Wasted
Exposure:
Breastfed hour after birth, ref: Yes
N studies: 7
Relative risk
Exposure level: No
1.0
0.8
0.7
0-6 months
6-24 months
0-24 months
Age category

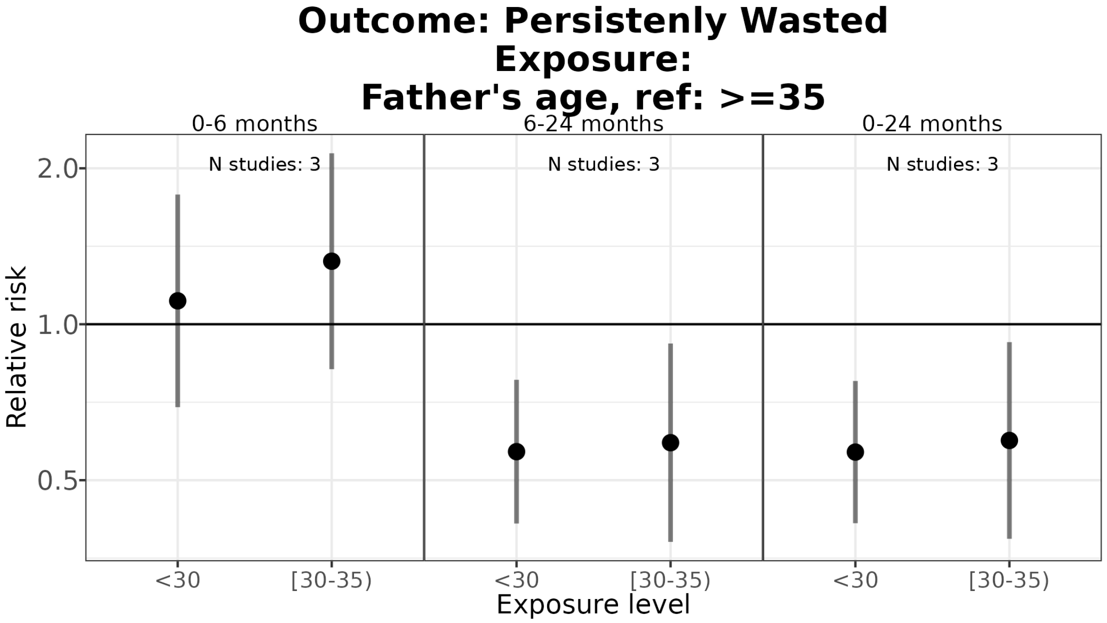
Outcome: Persistenly Wasted
Exposure:
Father's age, ref: >=35
0-6 months
6-24 months
0-24 months
N studies: 3
N studies: 3
N studies: 3
Relative risk
2.0
1.0
0.5
<30
[30-35)
<30
[30-35)
<30
[30-35)
Exposure level

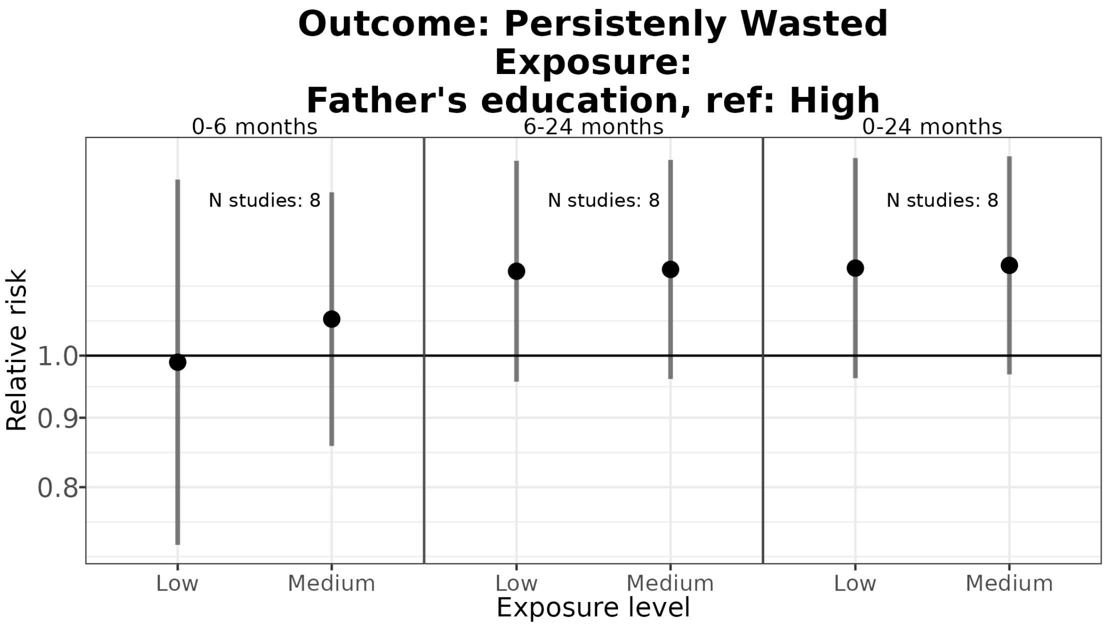
Outcome: Persistenly Wasted
Exposure:
Father's education, ref: High
0-6 months
6-24 months
0-24 months
N studies: 8
N studies: 8
N studies: 8
Relative risk
1.0
0.9
0.8
Low
Medium
Low
Medium
Low
Medium
Exposure level

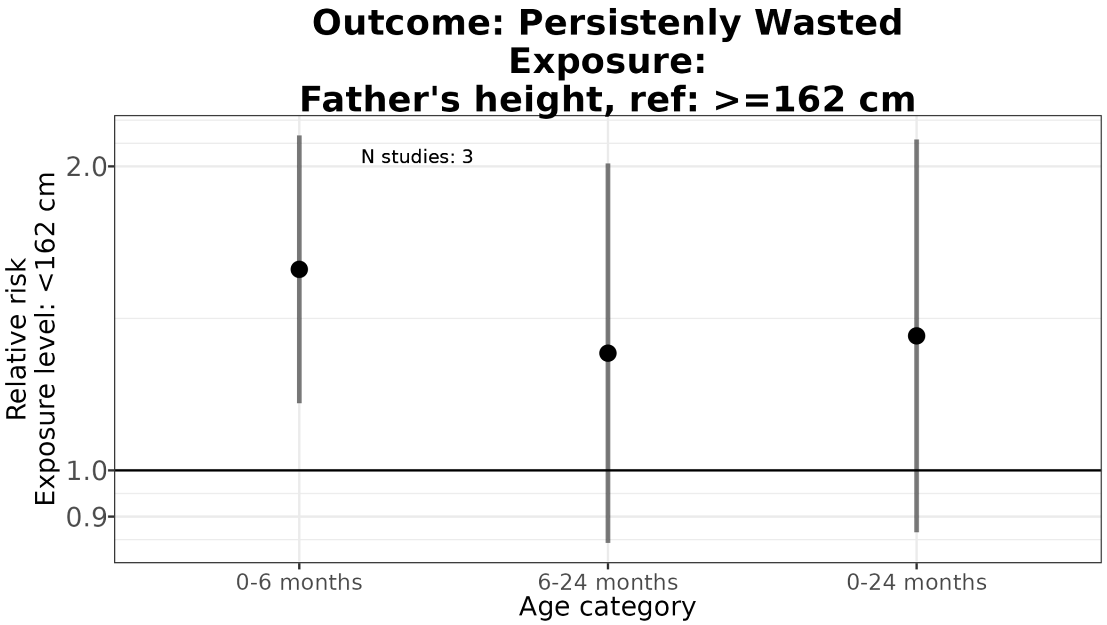
Outcome: Persistenly Wasted
Exposure:
Father's height, ref: >=162 cm
N studies: 3
Relative risk
Exposure level: <162 cm
2.0
1.0
0.9
0-6 months
6-24 months
0-24 months
Age category

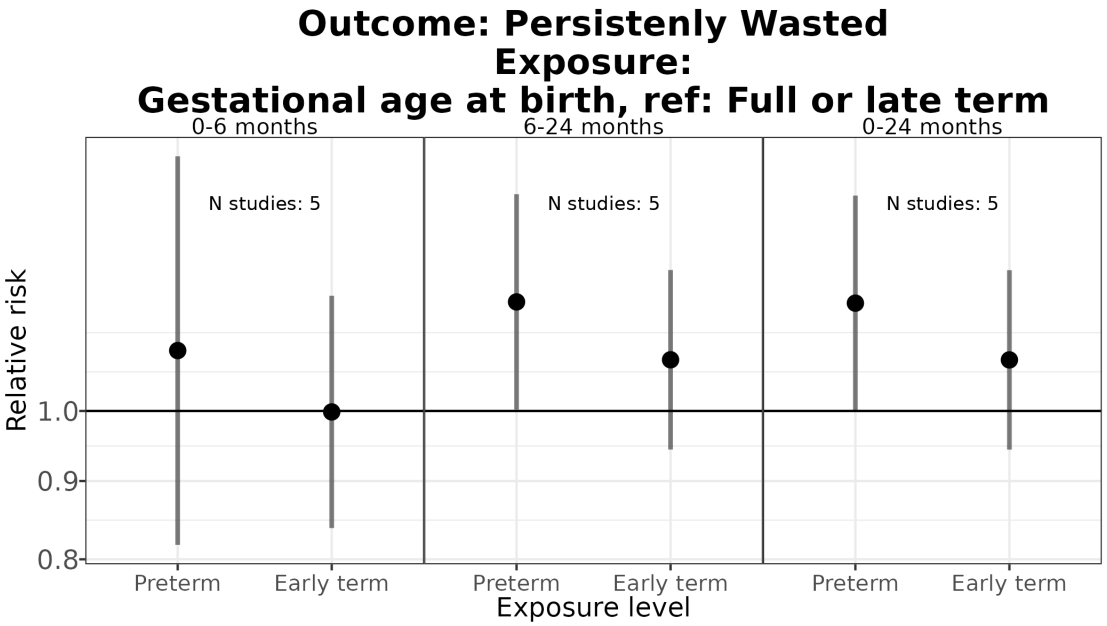
Outcome: Persistenly Wasted
Exposure:
Gestational age at birth, ref: Full or late term
0-6 months
6-24 months
0-24 months
N studies: 5
N studies: 5
N studies: 5
Relative risk
1.0
0.9
0.8
Preterm
Early term
Preterm
Early term
Preterm
Early term
Exposure level

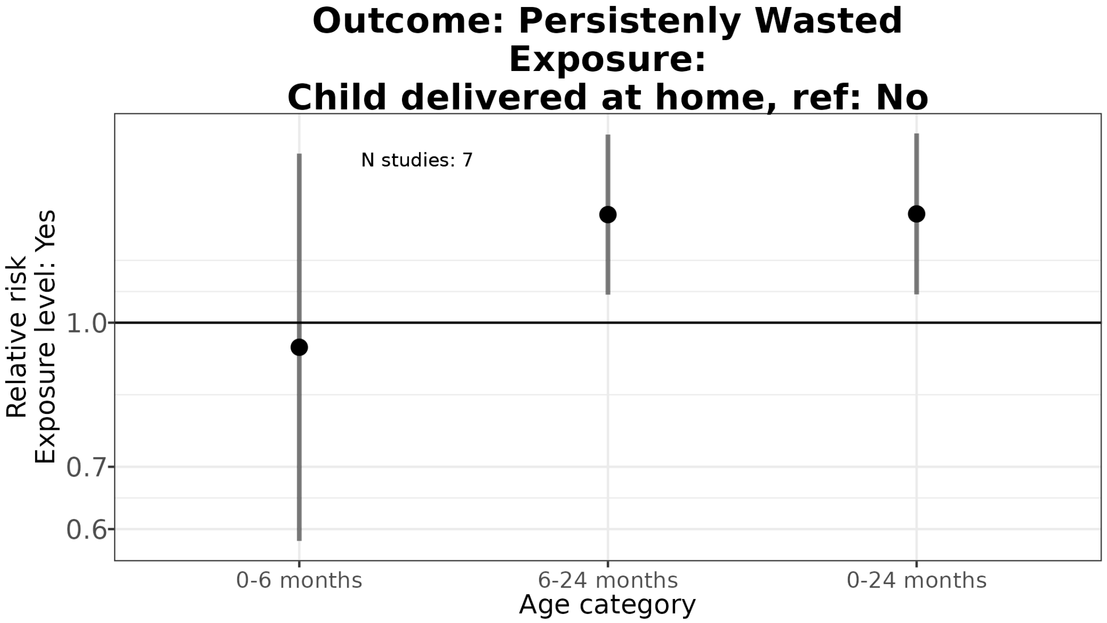
Outcome: Persistenly Wasted
Exposure:
Child delivered at home, ref: No
N studies: 7
Relative risk
Exposure level: Yes
1.0
0.7
0.6
0-6 months
6-24 months
0-24 months
Age category

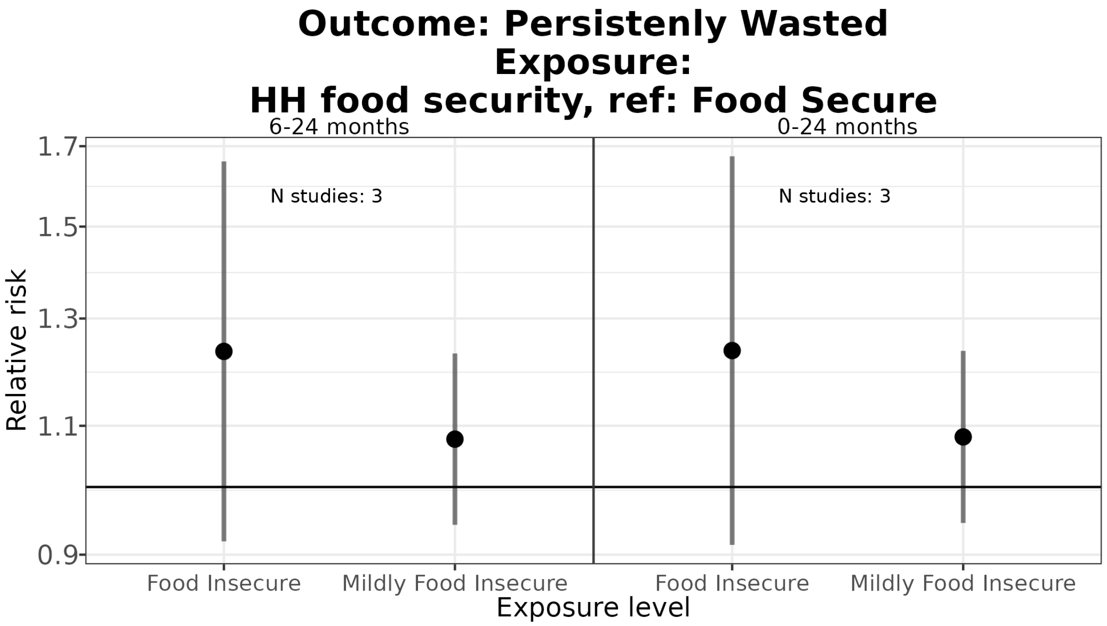
Outcome: Persistenly Wasted
Exposure:
HH food security, ref: Food Secure
6-24 months
0-24 months
N studies: 3
N studies: 3
Relative risk
1.7
1.5
1.3
1.1
0.9
Food Insecure
Mildly Food Insecure
Food Insecure
Mildly Food Insecure
Exposure level

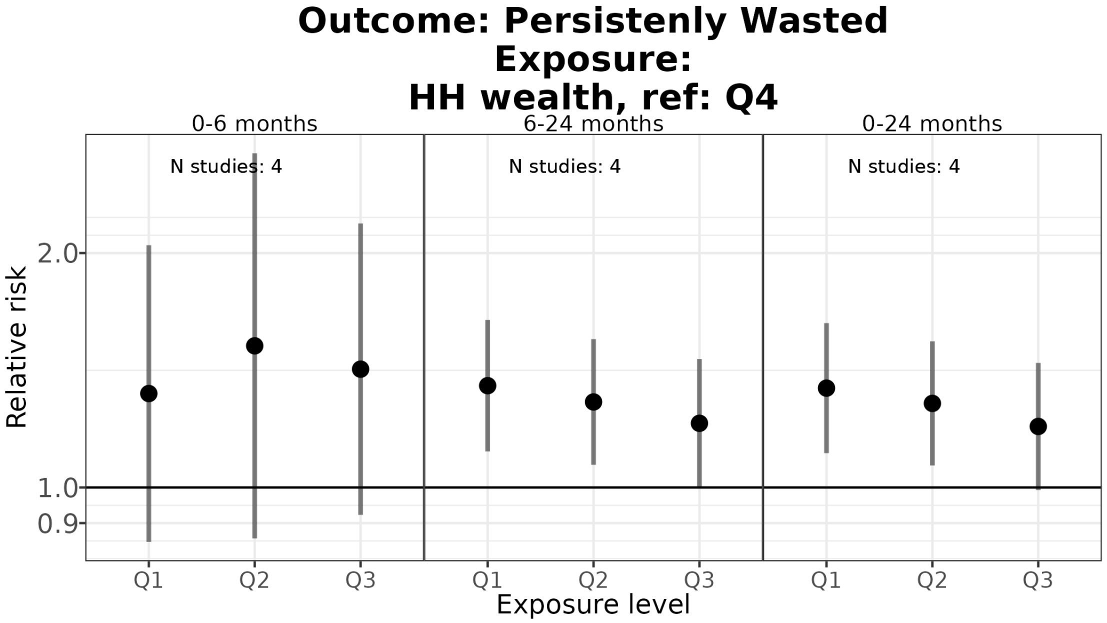
Outcome: Persistenly Wasted
Exposure:
HH wealth, ref: Q4
0-6 months
6-24 months
0-24 months
N studies: 4
N studies: 4
N studies: 4
Relative risk
2.0
1.0
0.9
Q1
Q2
Q3
Q1
Q2
Q3
Q1
Q2
Q3
Exposure level

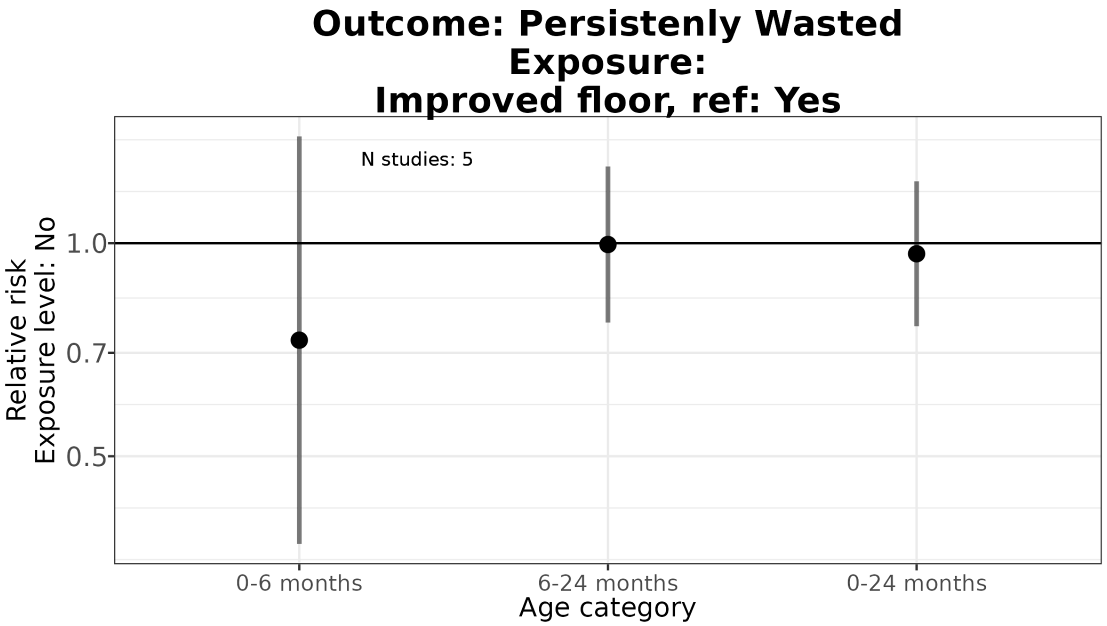
Outcome: Persistenly Wasted
Exposure:
Improved floor, ref: Yes
N studies: 5
1.0
0.7
0.5
Relative risk
Exposure level: No
0-6 months
6-24 months
0-24 months
Age category

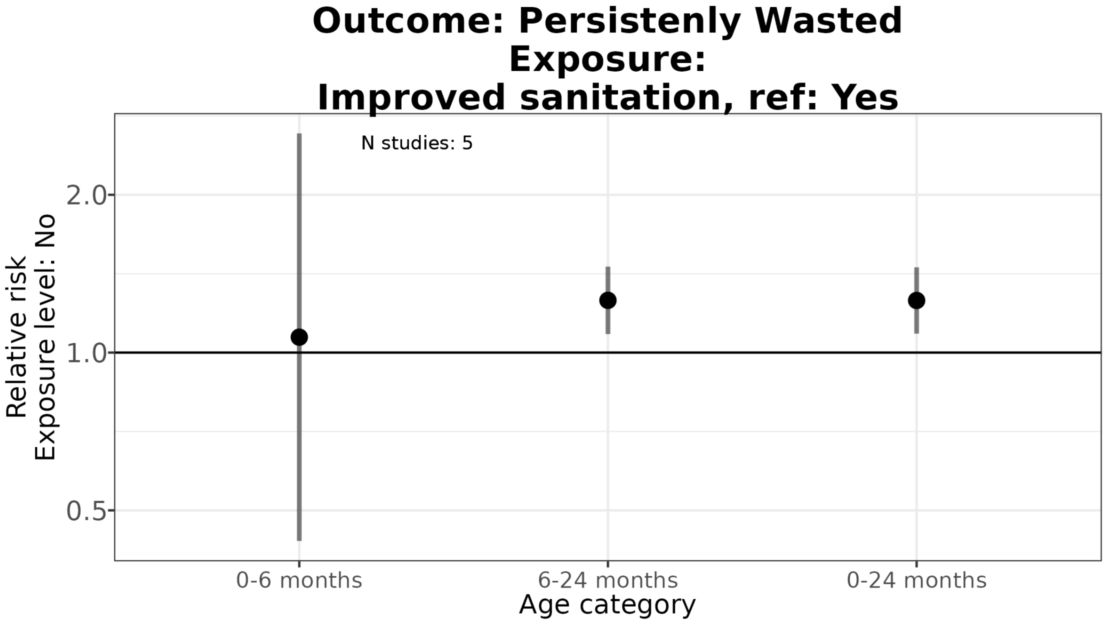
Outcome: Persistenly Wasted
Exposure:
Improved sanitation, ref: Yes
N studies: 5
2.0
1.0
0.5
Relative risk
Exposure level: No
0-6 months
6-24 months
0-24 months
Age category

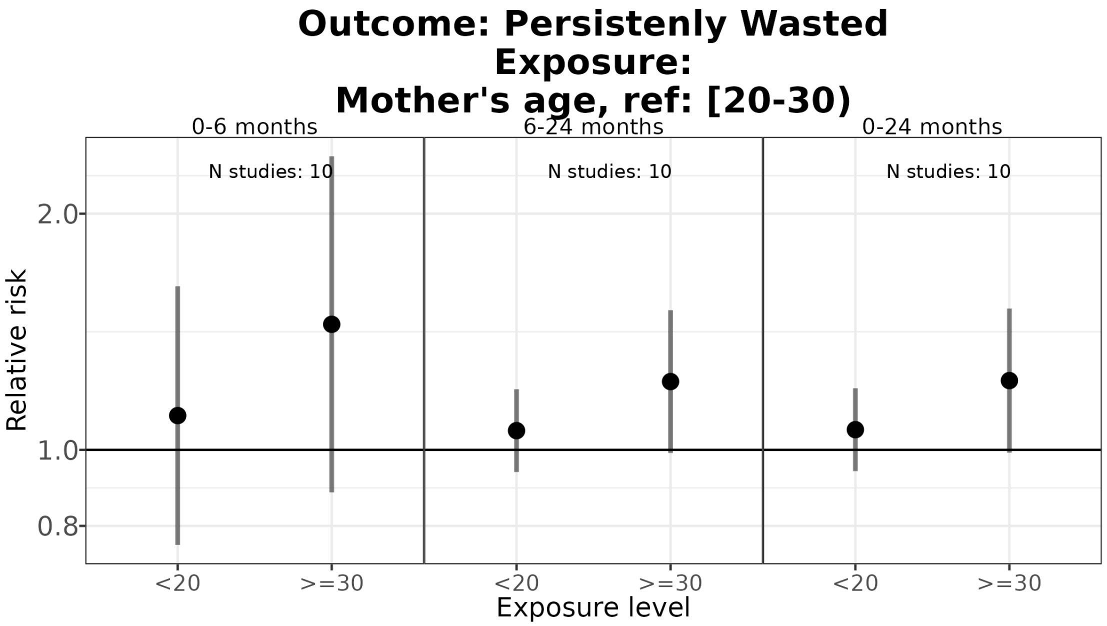
Outcome: Persistenly Wasted
Exposure:
Mother's age, ref: [20-30)
0-6 months
6-24 months
0-24 months
N studies: 10
N studies: 10
N studies: 10
Relative risk
2.0
1.0
0.8
<20
>=30
<20
>=30
<20
>=30
Exposure level

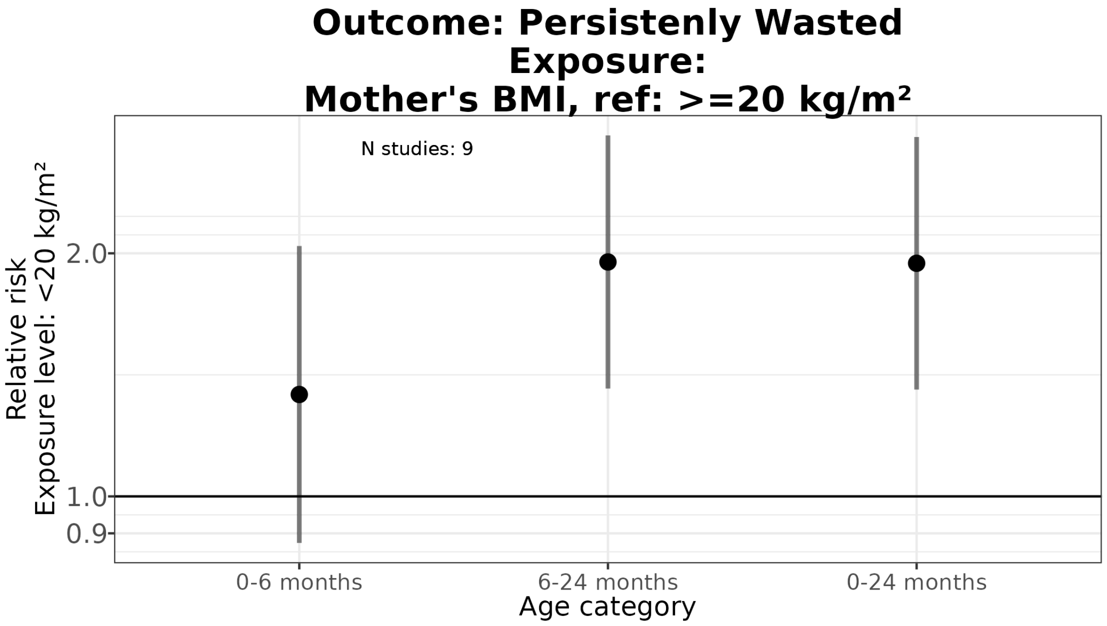
Outcome: Persistenly Wasted
Exposure:
Mother's BMI, ref: >=20 kg/m²
N studies: 9
Relative risk
Exposure level: <20 kg/m²
2.0
1.0
0.9
0-6 months
6-24 months
0-24 months
Age category

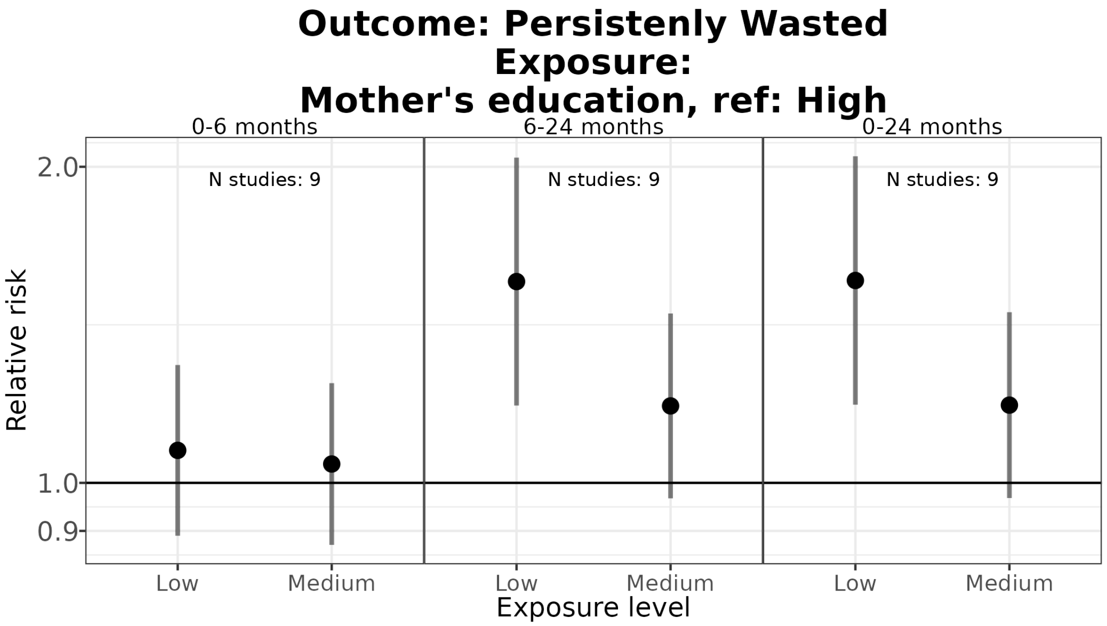

Outcome: Persistenly Wasted
Exposure:
Mother's education, ref: High
0-6 months
6-24 months
0-24 months
N studies: 9
N studies: 9
N studies: 9
Relative risk
2.0
1.0
0.9
Low
Medium
Low
Medium
Low
Medium
Exposure level


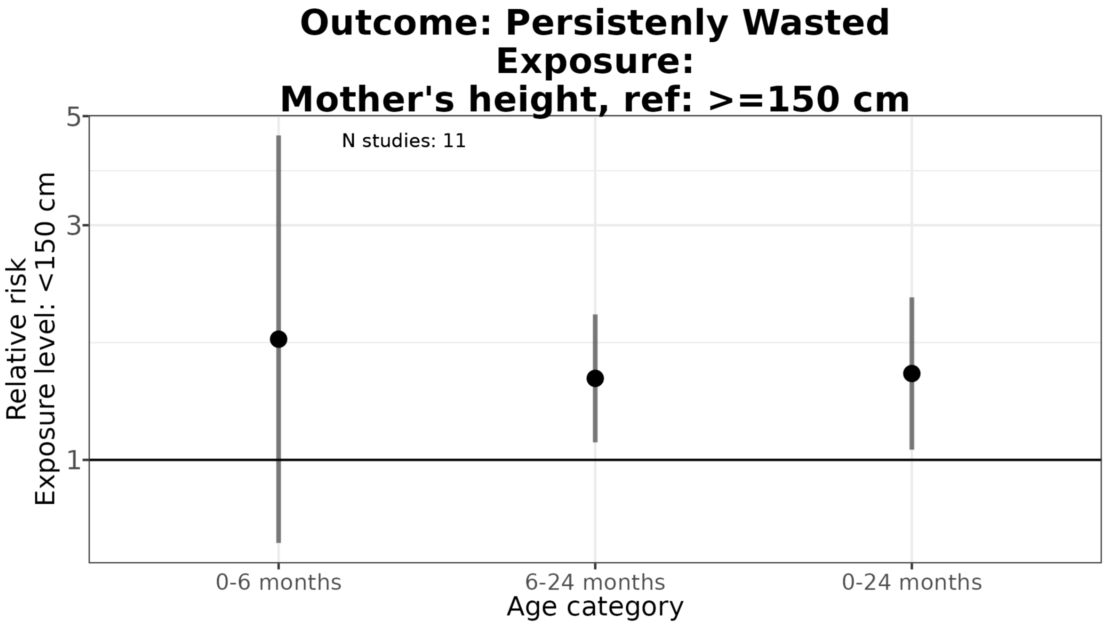

Outcome: Persistenly Wasted
Exposure:
Mother's height, ref: >=150 cm
N studies: 11
Relative risk
Exposure level: <150 cm
5
3
1
0-6 months
6-24 months
0-24 months
Age category

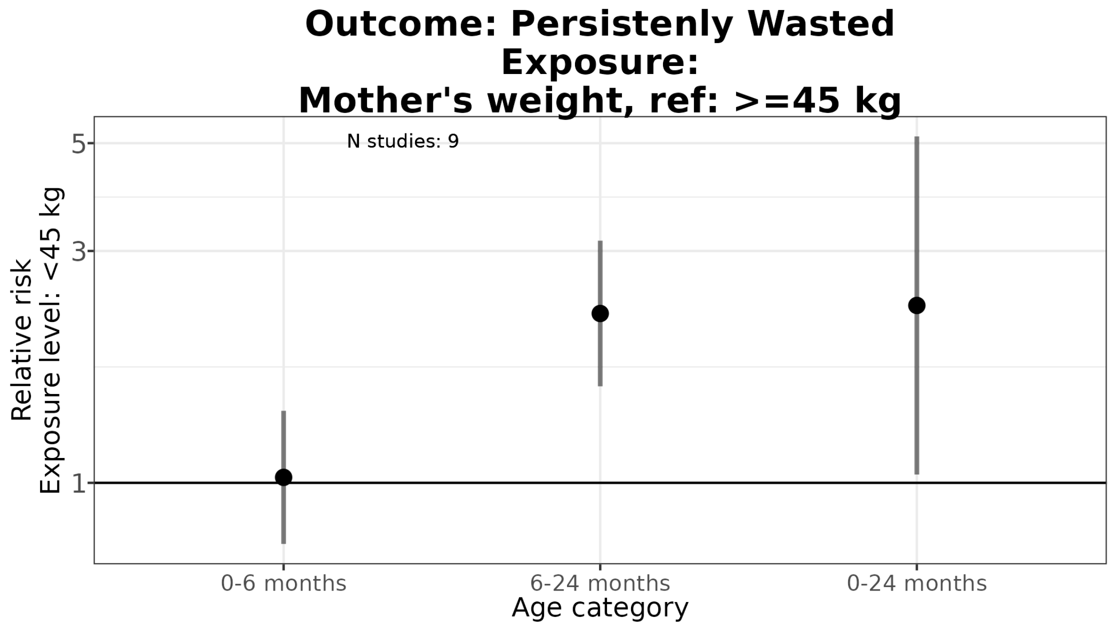
Outcome: Persistenly Wasted
Exposure:
Mother's weight, ref: >=45 kg
N studies: 9
Relative risk
Exposure level: <45 kg
5
3
1
0-6 months
6-24 months
0-24 months
Age category

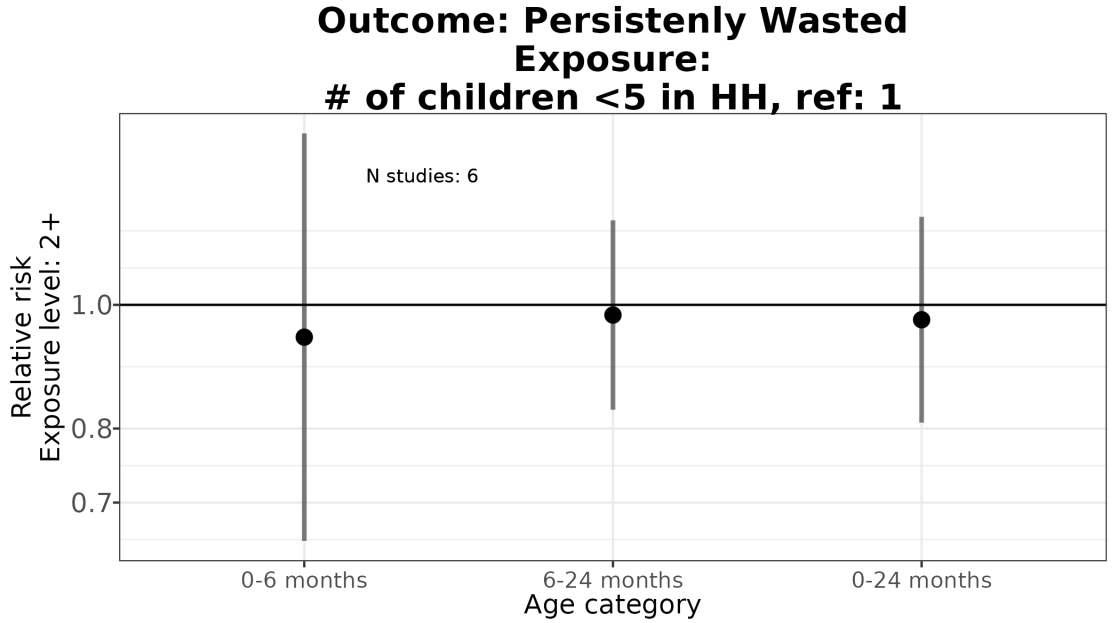
Outcome: Persistenly Wasted
Exposure:
# of children <5 in HH, ref: 1
N studies: 6
Relative risk
Exposure level: 2+
1.0
0.8
0.7
0-6 months
6-24 months
0-24 months
Age category

**Outcome: Persistenly Wasted**
**Exposure:**
**# of people in HH, ref: 3 or less**

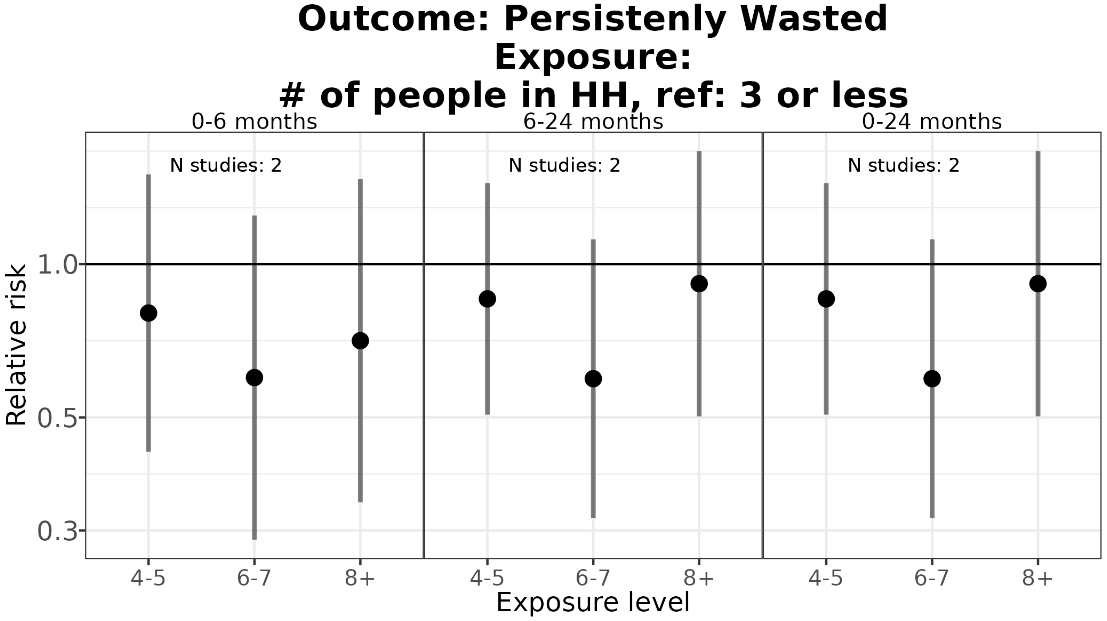


**Outcome: Persistenly Wasted**
**Exposure:**
**# of rooms in HH, ref: 4+**

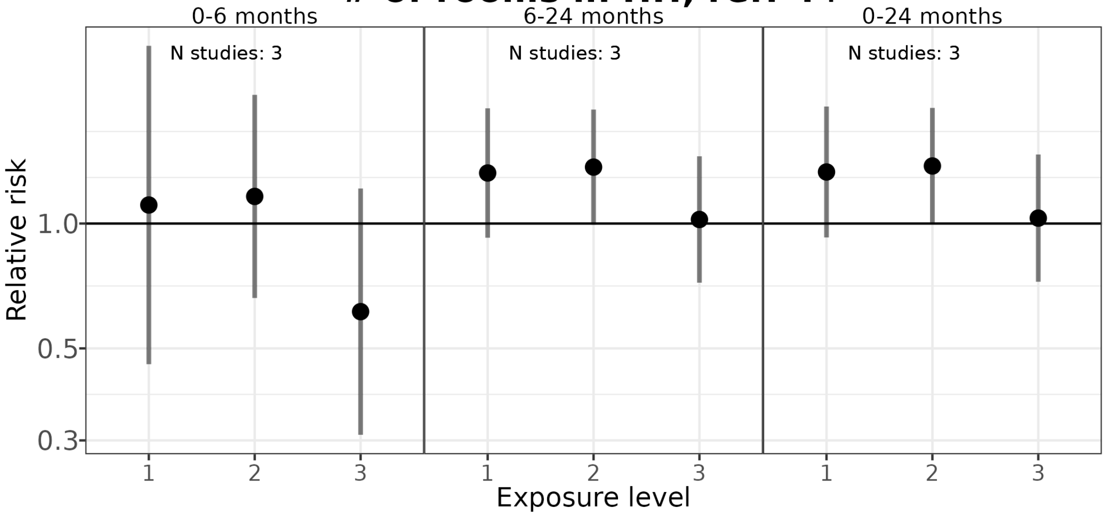

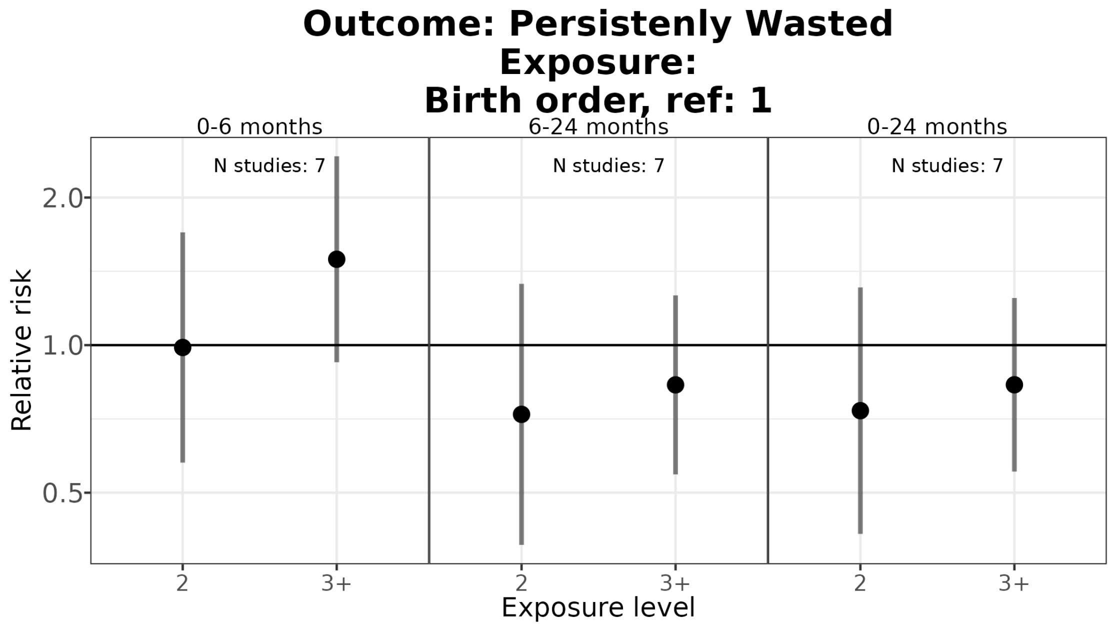
Outcome: Persistenly Wasted
Exposure:
Birth order, ref: 1
0-6 months
6-24 months
0-24 months
N studies: 7
N studies: 7
N studies: 7
Relative risk
2.0
1.0
0.5
2
3+
2
3+
2
3+
Exposure level

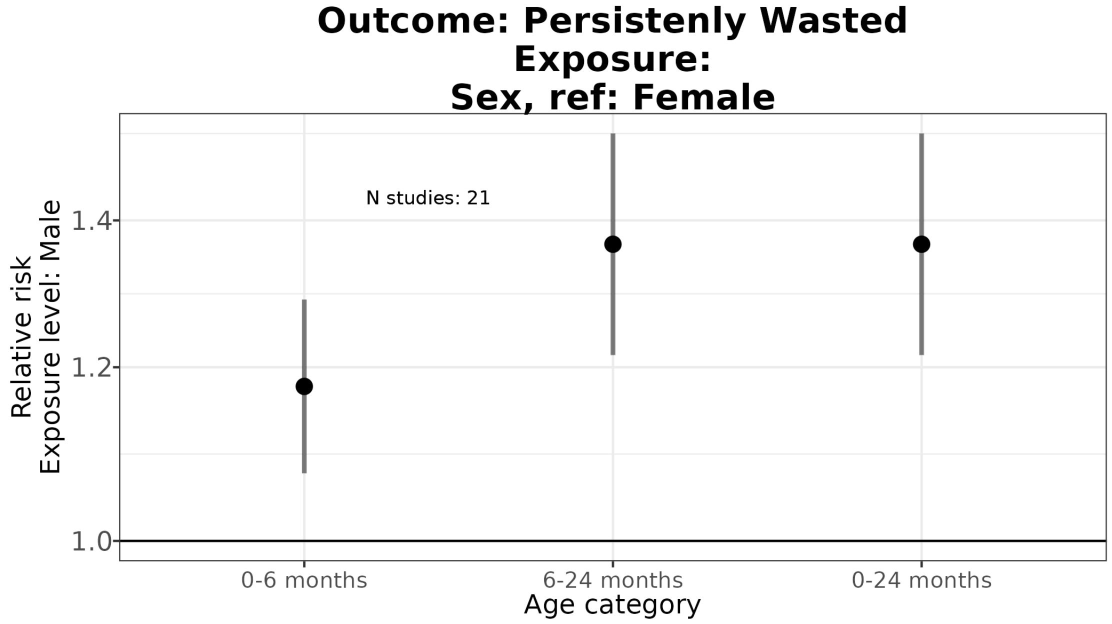
Outcome: Persistenly Wasted
Exposure:
Sex, ref: Female
N studies: 21
Relative risk
Exposure level: Male
1.4
1.2
1.0
0-6 months
6-24 months
0-24 months
Age category

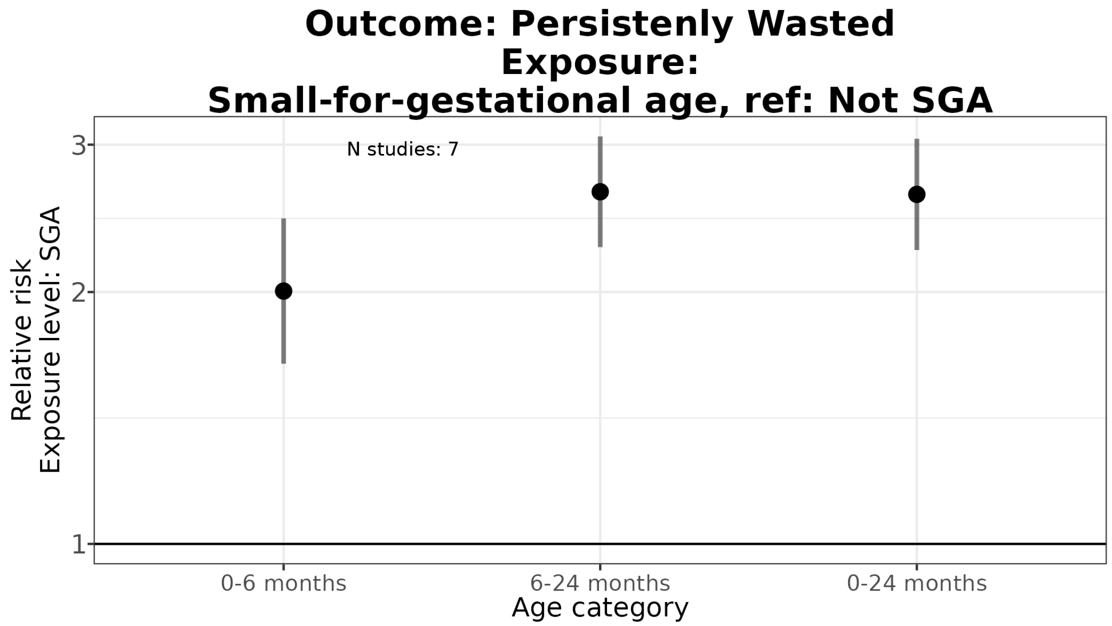
Outcome: Persistenly Wasted
Exposure:
Small-for-gestational age, ref: Not SGA
N studies: 7
Relative risk
Exposure level: SGA
3
2
1
0-6 months
6-24 months
0-24 months
Age category

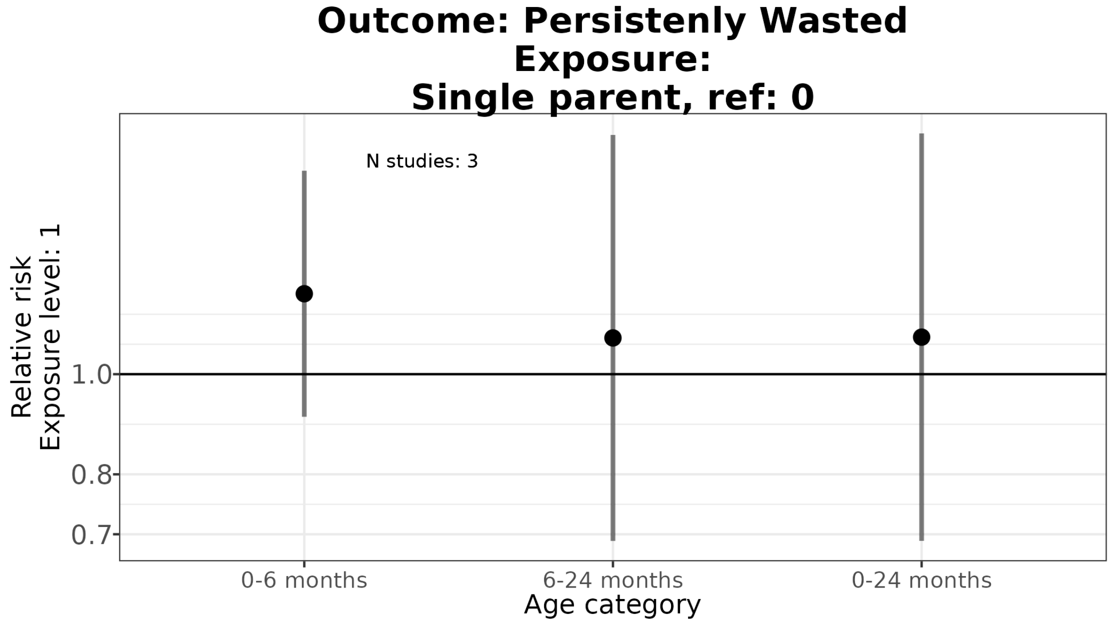
Outcome: Persistenly Wasted
Exposure:
Single parent, ref: 0
N studies: 3
Relative risk
Exposure level: 1
1.0
0.8
0.7
0-6 months
6-24 months
0-24 months
Age category

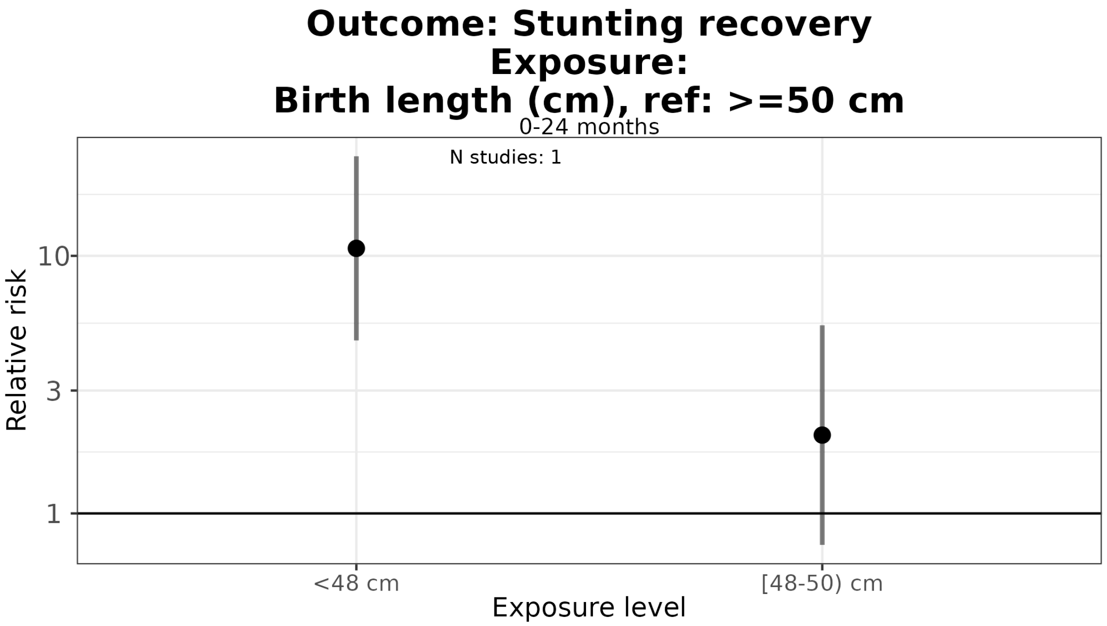

Outcome: Stunting recovery
Exposure:
Birth length (cm), ref: >=50 cm
0-24 months
N studies: 1
Relative risk
10
3
1
<48 cm
[48-50) cm
Exposure level


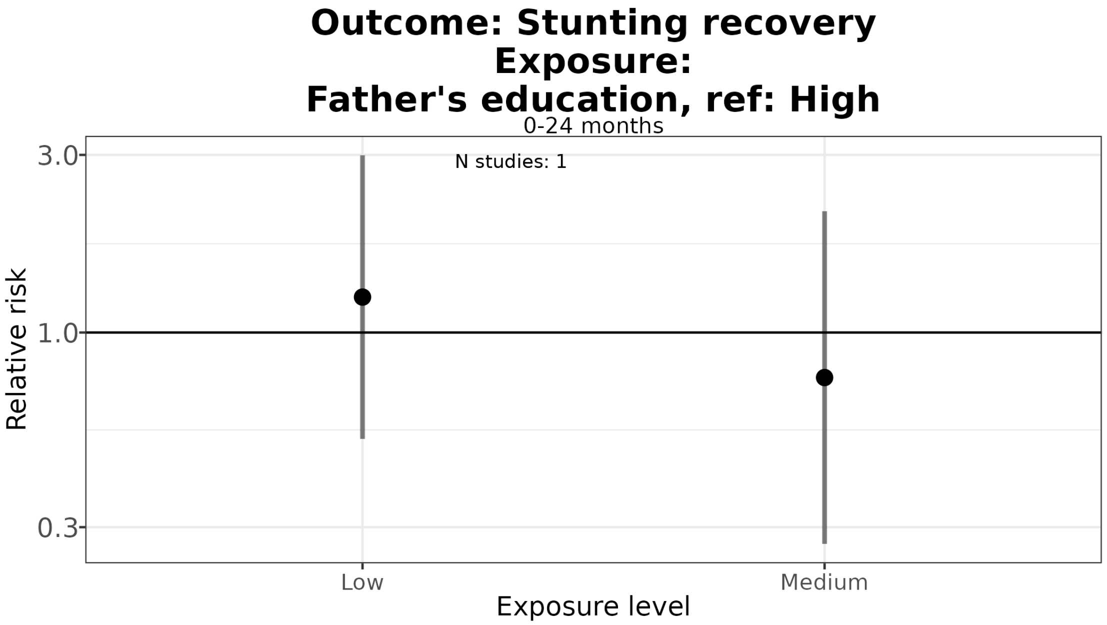

Outcome: Stunting recovery
Exposure:
Father's education, ref: High
0-24 months
N studies: 1
Relative risk
3.0
1.0
0.3
Low
Medium
Exposure level

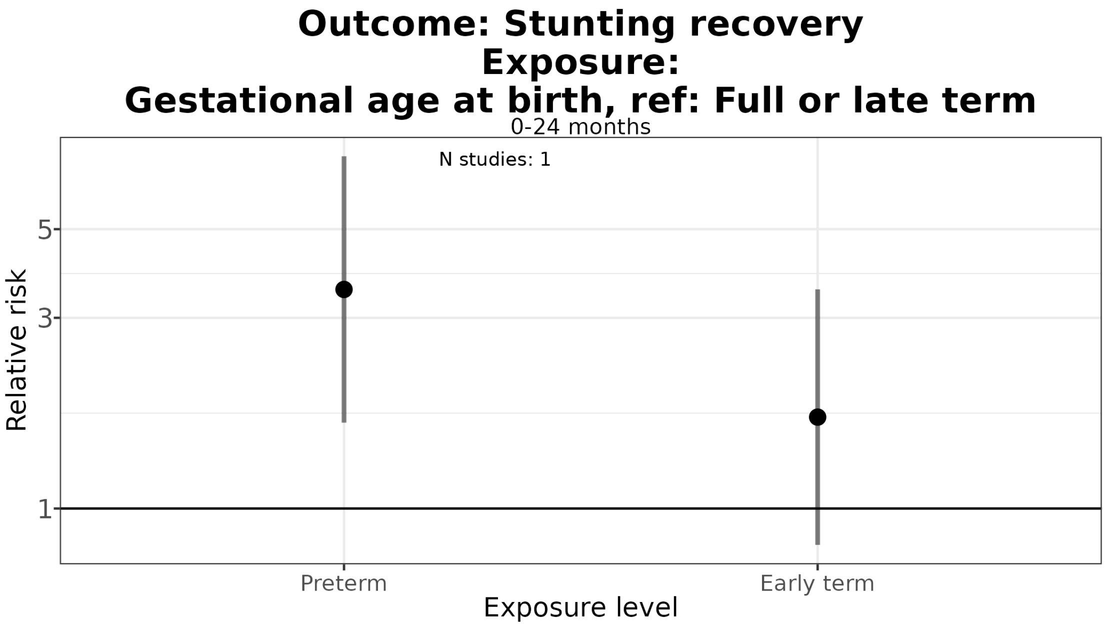
Outcome: Stunting recovery
Exposure:
Gestational age at birth, ref: Full or late term
0-24 months
N studies: 1
Relative risk
5
3
1
Preterm
Early term
Exposure level

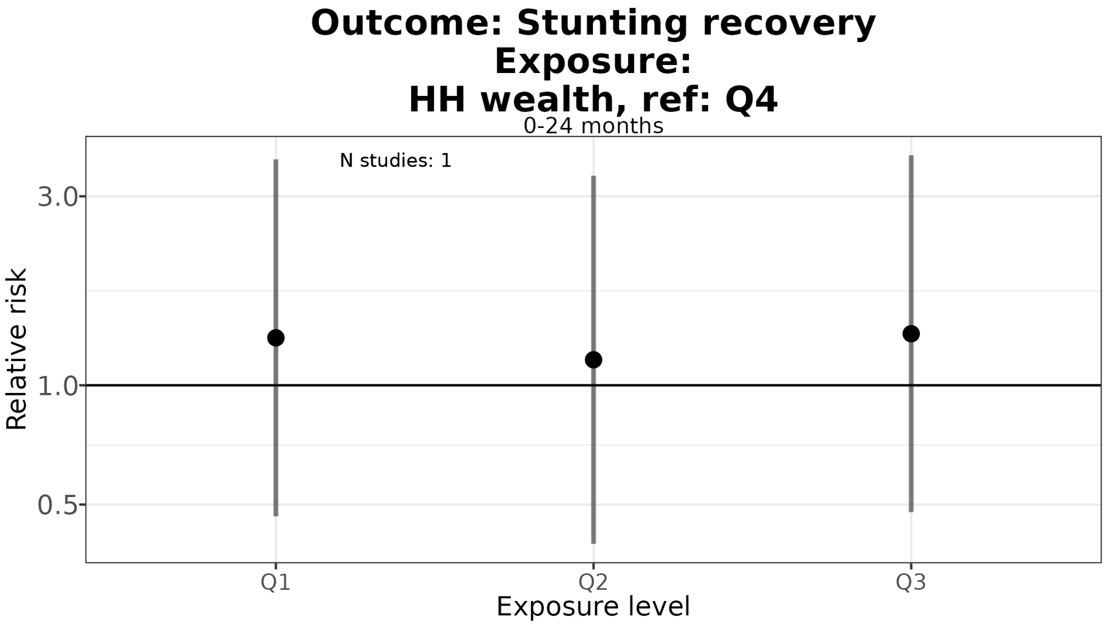
Outcome: Stunting recovery
Exposure:
HH wealth, ref: Q4
0-24 months
N studies: 1
Relative risk
3.0
1.0
0.5
Q1
Q2
Q3
Exposure level

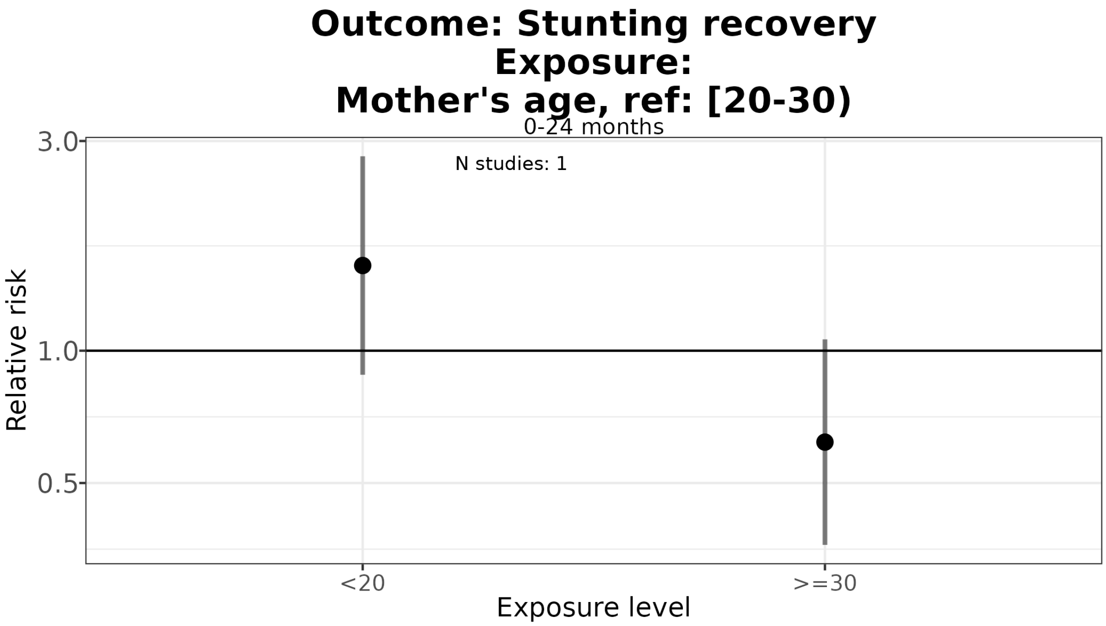
Outcome: Stunting recovery
Exposure:
Mother's age, ref: [20-30)
0-24 months
N studies: 1
3.0
1.0
0.5
Relative risk
<20
>=30
Exposure level

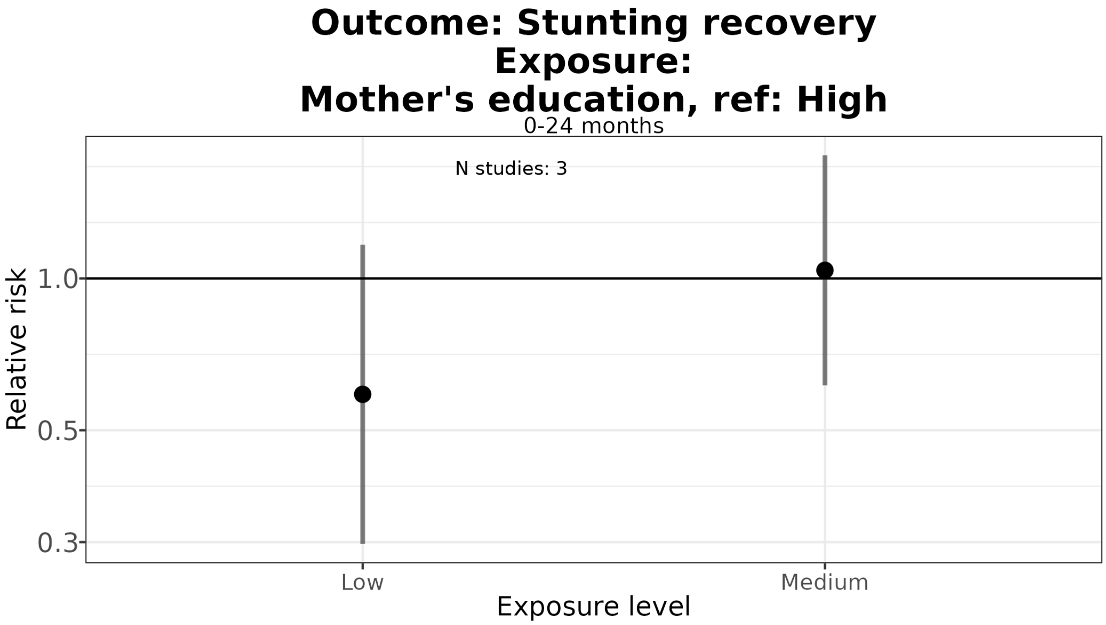
Outcome: Stunting recovery
Exposure:
Mother's education, ref: High
0-24 months
N studies: 3
1.0
0.5
0.3
Relative risk
Low
Medium
Exposure level

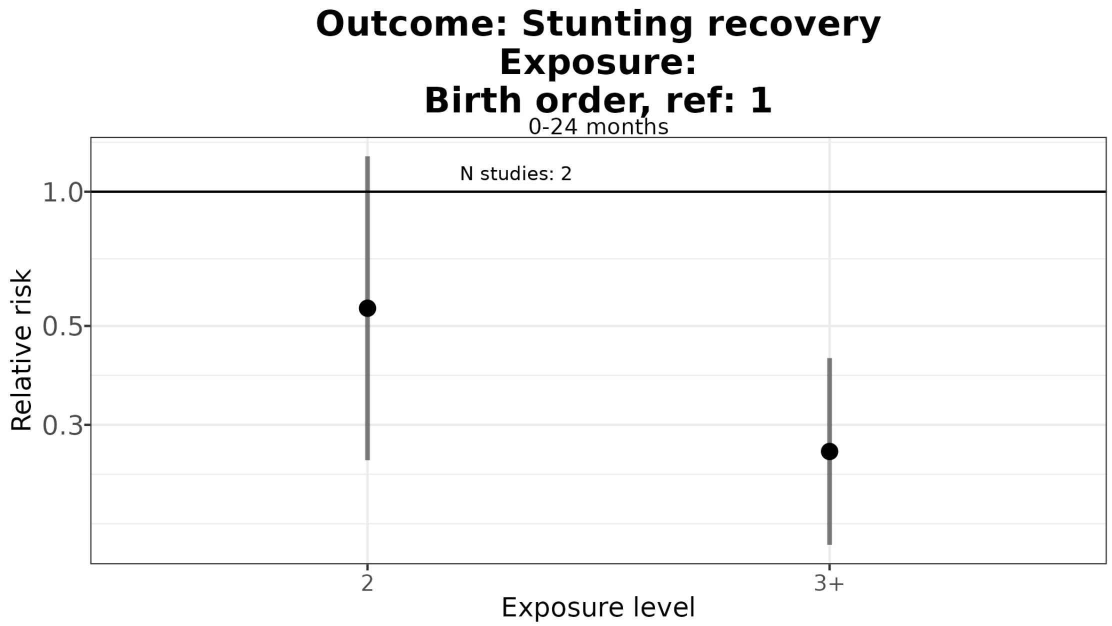

Outcome: Stunting recovery
Exposure:
Birth order, ref: 1
0-24 months
N studies: 2
1.0
0.5
0.3
Relative risk
2
3+
Exposure level


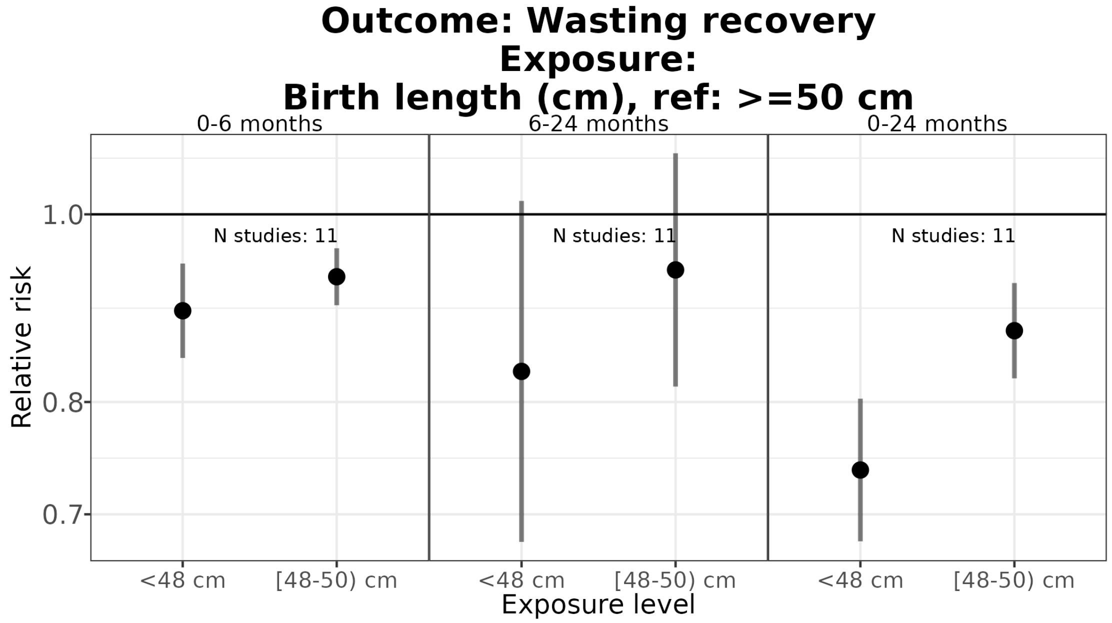

Outcome: Wasting recovery
Exposure:
Birth length (cm), ref: >=50 cm
0-6 months
6-24 months
0-24 months
N studies: 11
N studies: 11
N studies: 11
1.0
0.8
0.7
Relative risk
<48 cm
[48-50) cm
<48 cm
[48-50) cm
<48 cm
[48-50) cm
Exposure level

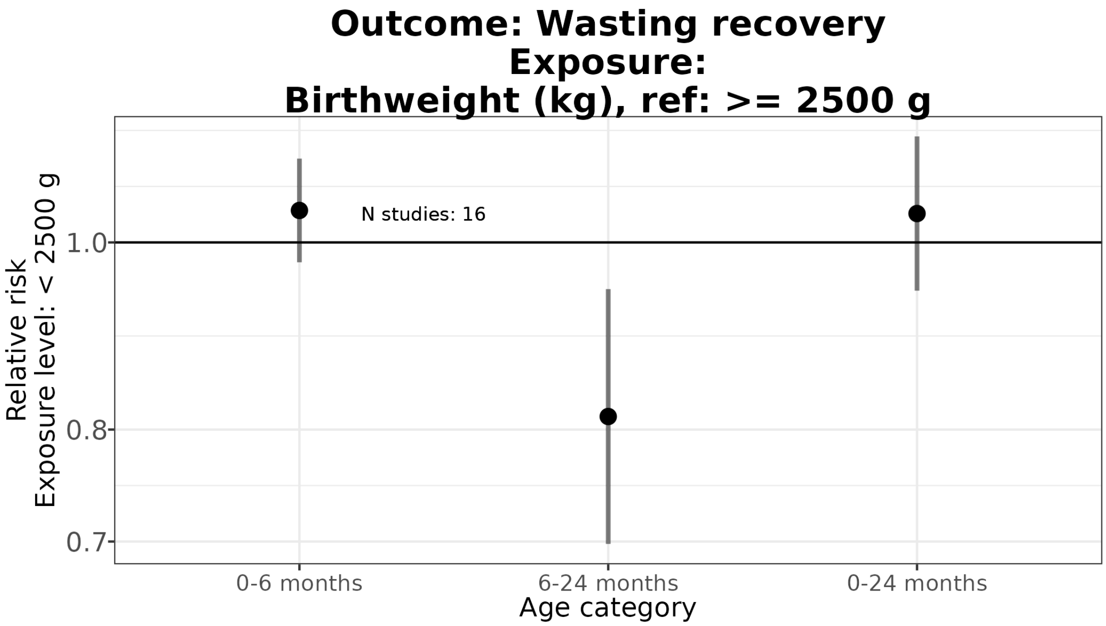

Outcome: Wasting recovery
Exposure:
Birthweight (kg), ref: >= 2500 g
Relative risk
Exposure level: < 2500 g
1.0
0.8
0.7
N studies: 16
0-6 months
6-24 months
0-24 months
Age category


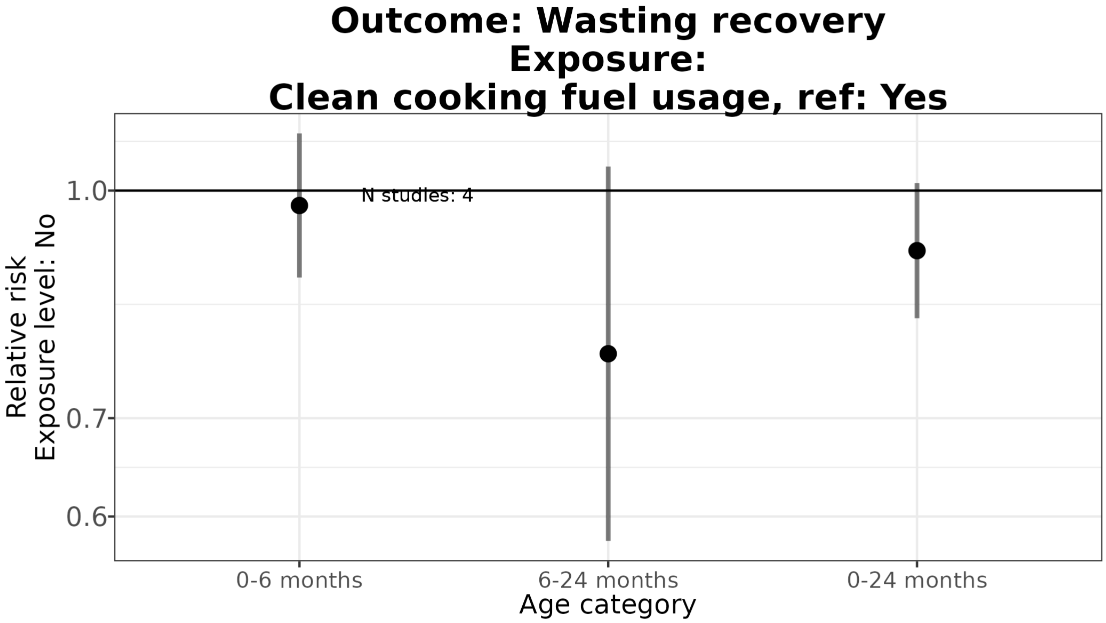

Outcome: Wasting recovery
Exposure:
Clean cooking fuel usage, ref: Yes
Relative risk
Exposure level: No
1.0
0.7
0.6
N studies: 4
0-6 months
6-24 months
0-24 months
Age category

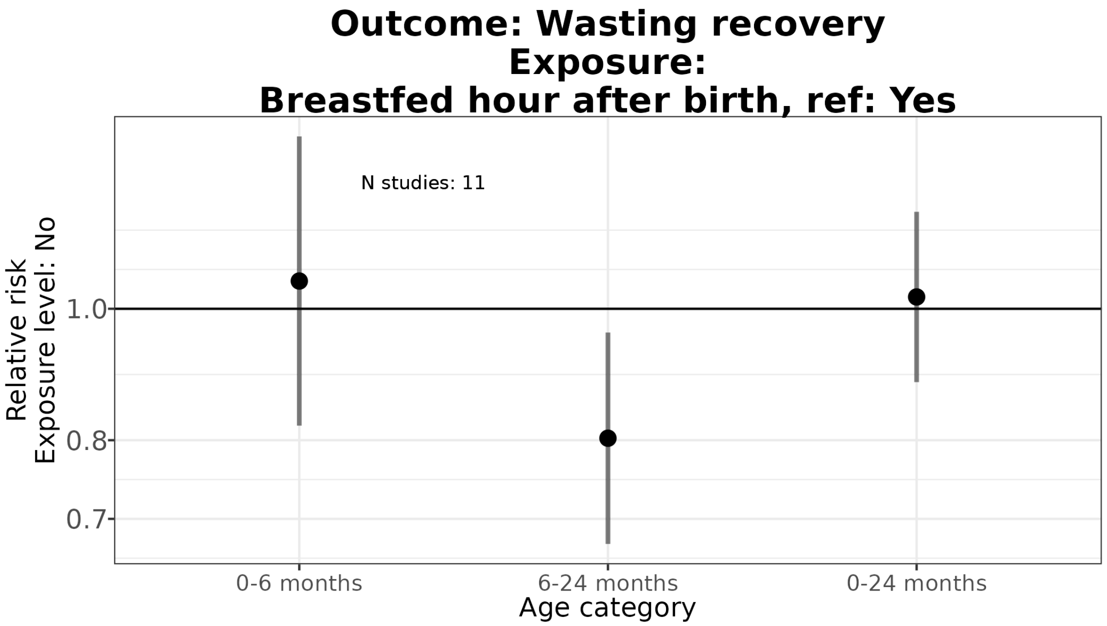
Outcome: Wasting recovery
Exposure:
Breastfed hour after birth, ref: Yes
N studies: 11
Relative risk
Exposure level: No
1.0
0.8
0.7
0-6 months
6-24 months
0-24 months
Age category

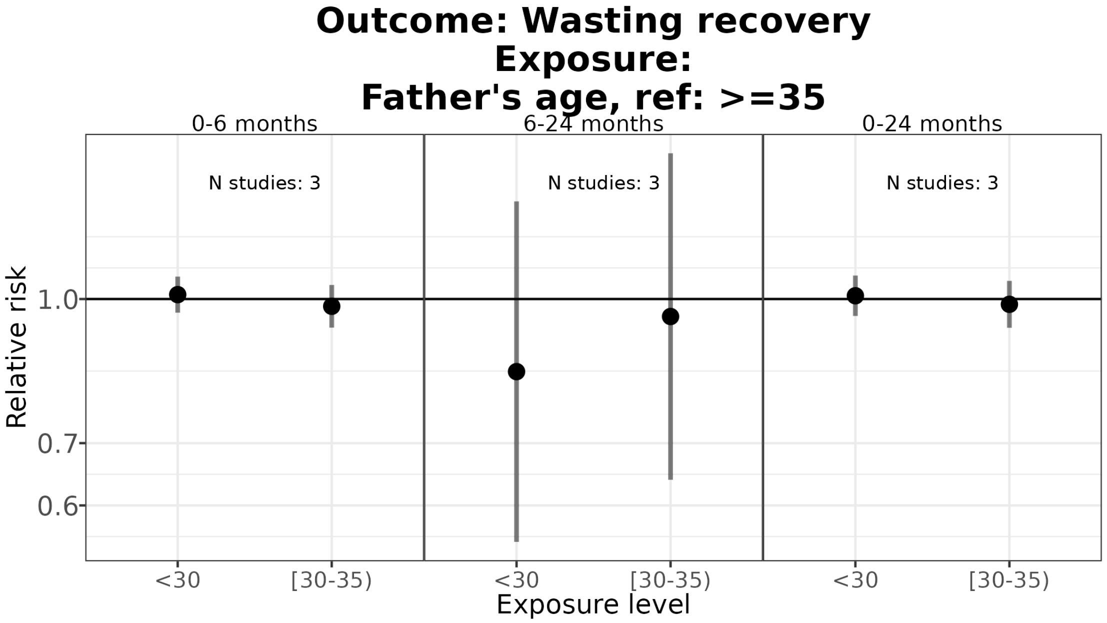
Outcome: Wasting recovery
Exposure:
Father's age, ref: >=35
0-6 months
6-24 months
0-24 months
N studies: 3
N studies: 3
N studies: 3
Relative risk
1.0
0.7
0.6
<30
[30-35)
<30
[30-35)
<30
[30-35)
Exposure level

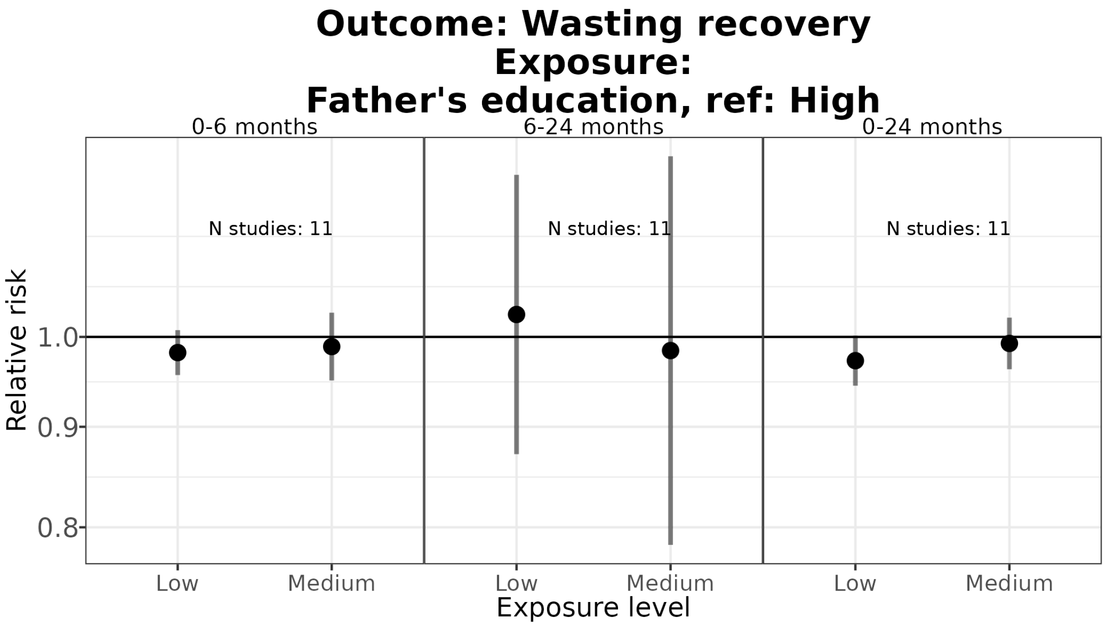
Outcome: Wasting recovery
Exposure:
Father's education, ref: High
0-6 months
6-24 months
0-24 months
N studies: 11
N studies: 11
N studies: 11
Relative risk
1.0
0.9
0.8
Low
Medium
Low
Medium
Low
Medium
Exposure level

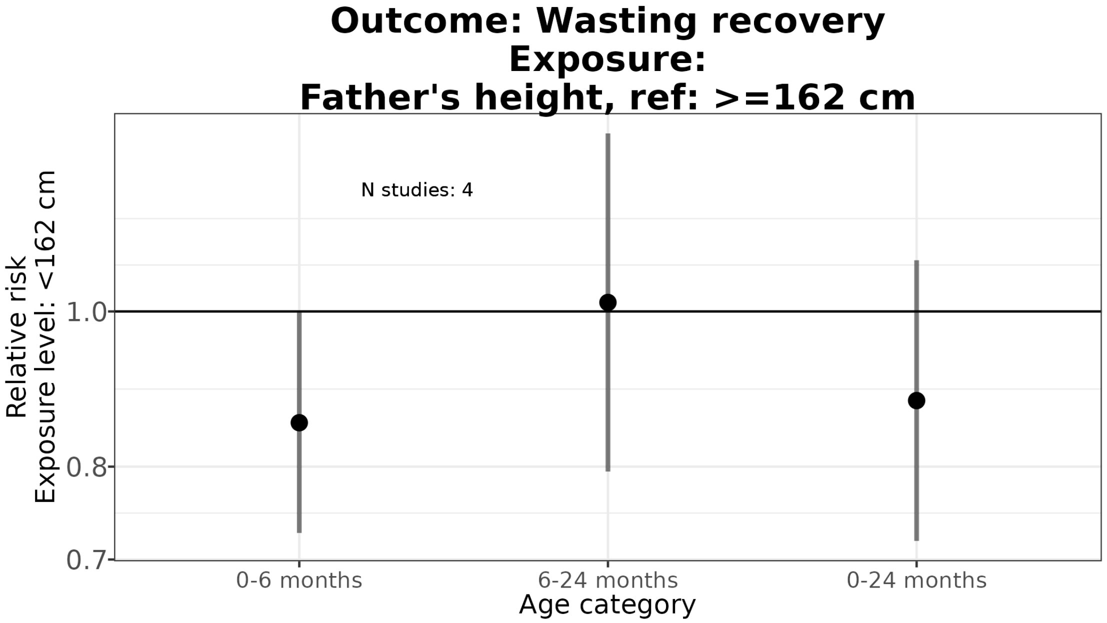
Outcome: Wasting recovery
Exposure:
Father's height, ref: >=162 cm
N studies: 4
Relative risk
Exposure level: <162 cm
1.0
0.8
0.7
0-6 months
6-24 months
0-24 months
Age category

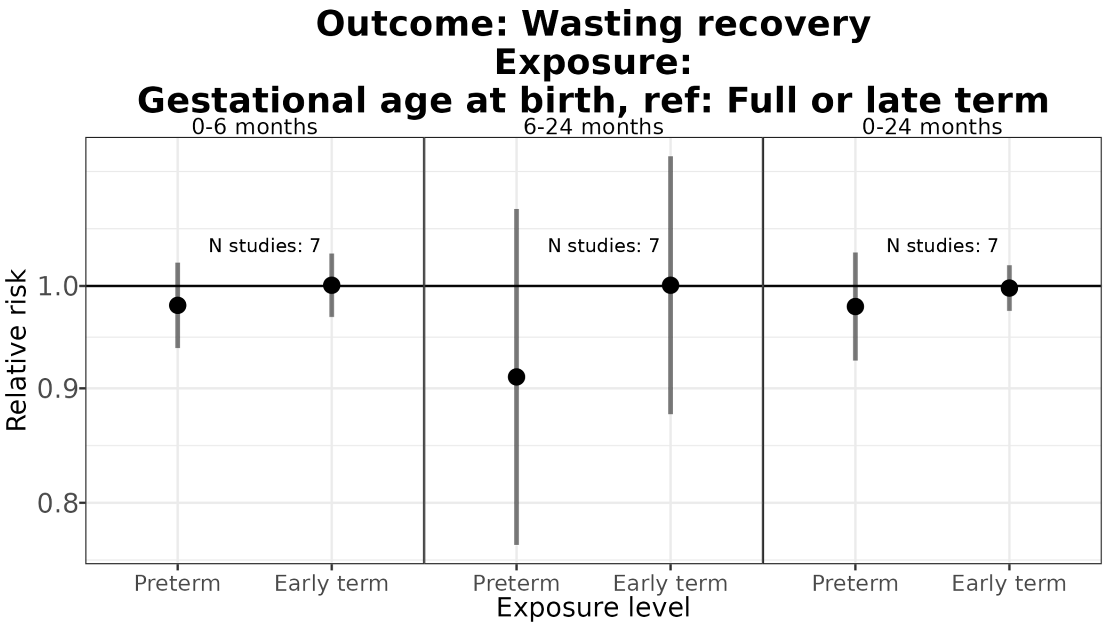
Outcome: Wasting recovery
Exposure:
Gestational age at birth, ref: Full or late term
0-6 months
6-24 months
0-24 months
N studies: 7
N studies: 7
N studies: 7
Relative risk
1.0
0.9
0.8
Preterm
Early term
Preterm
Early term
Preterm
Early term
Exposure level

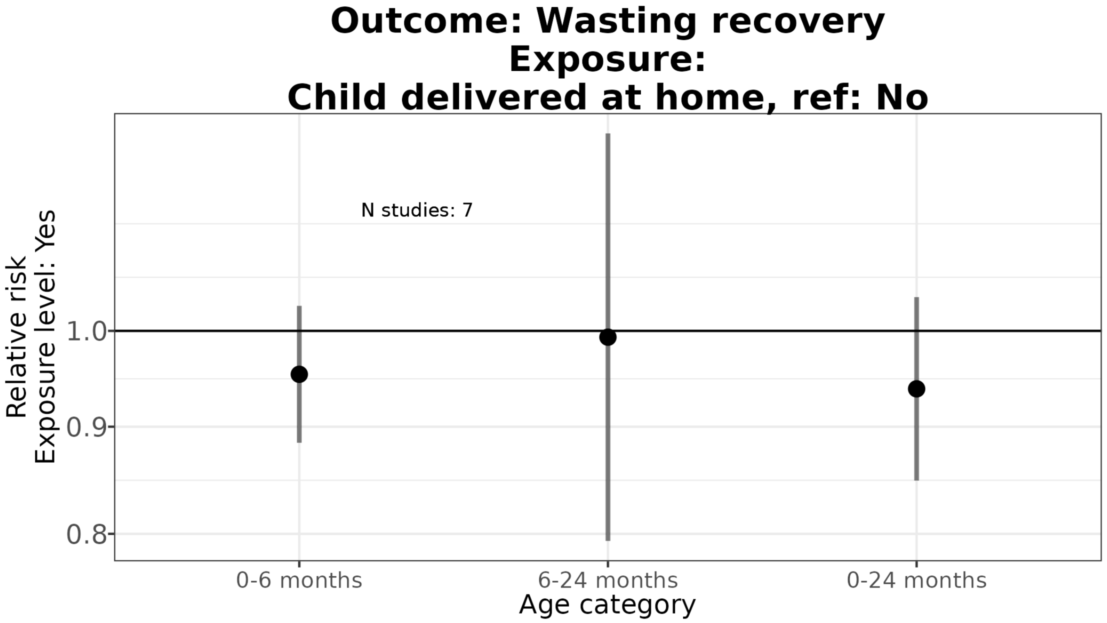
Outcome: Wasting recovery
Exposure:
Child delivered at home, ref: No
N studies: 7
Relative risk
Exposure level: Yes
1.0
0.9
0.8
0-6 months
6-24 months
0-24 months
Age category

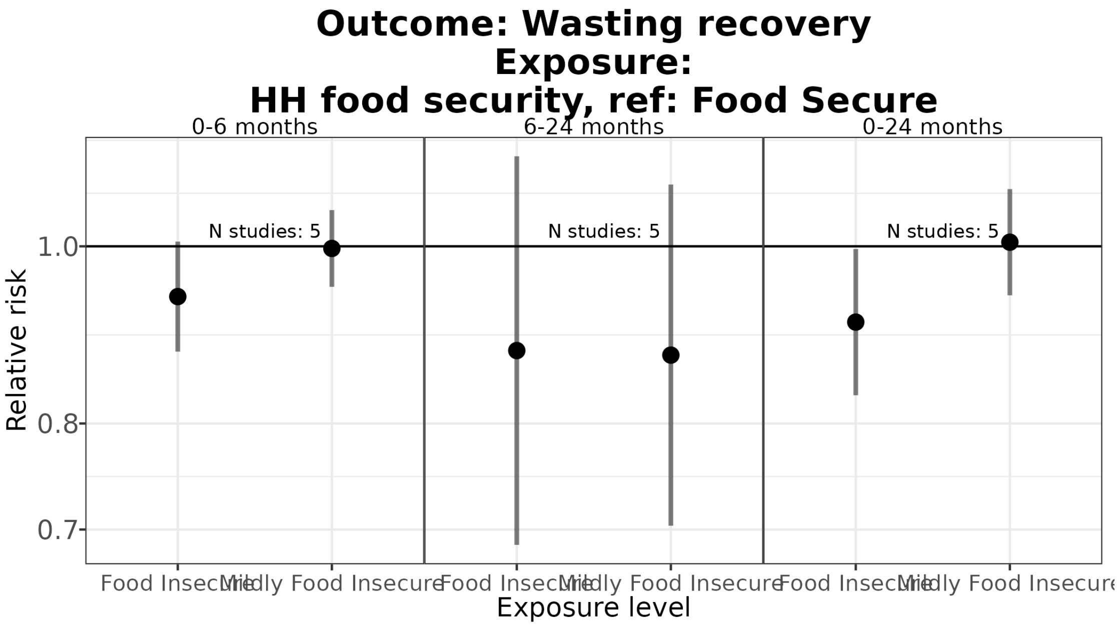
Outcome: Wasting recovery
Exposure:
HH food security, ref: Food Secure
0-6 months
6-24 months
0-24 months
N studies: 5
N studies: 5
N studies: 5
Relative risk
1.0
0.8
0.7
Food Insecure
Mildly Food Insecure
Exposure level

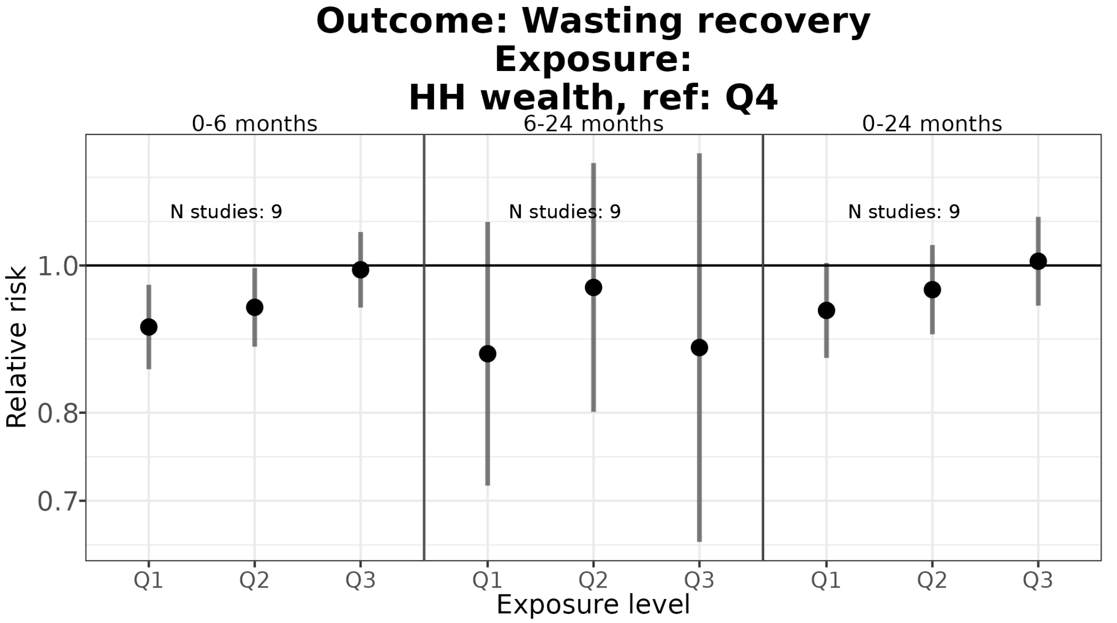
Outcome: Wasting recovery
Exposure:
HH wealth, ref: Q4
0-6 months
6-24 months
0-24 months
N studies: 9
N studies: 9
N studies: 9
Relative risk
1.0
0.8
0.7
Q1
Q2
Q3
Exposure level

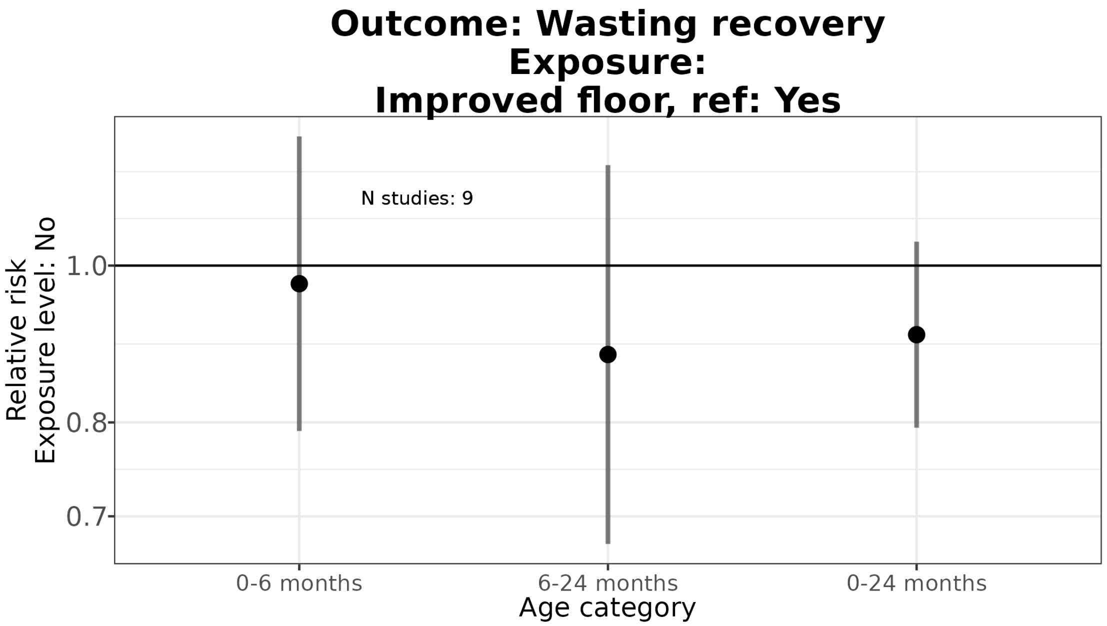
Outcome: Wasting recovery
Exposure:
Improved floor, ref: Yes
N studies: 9
Relative risk
Exposure level: No
1.0
0.8
0.7
0-6 months
6-24 months
0-24 months
Age category

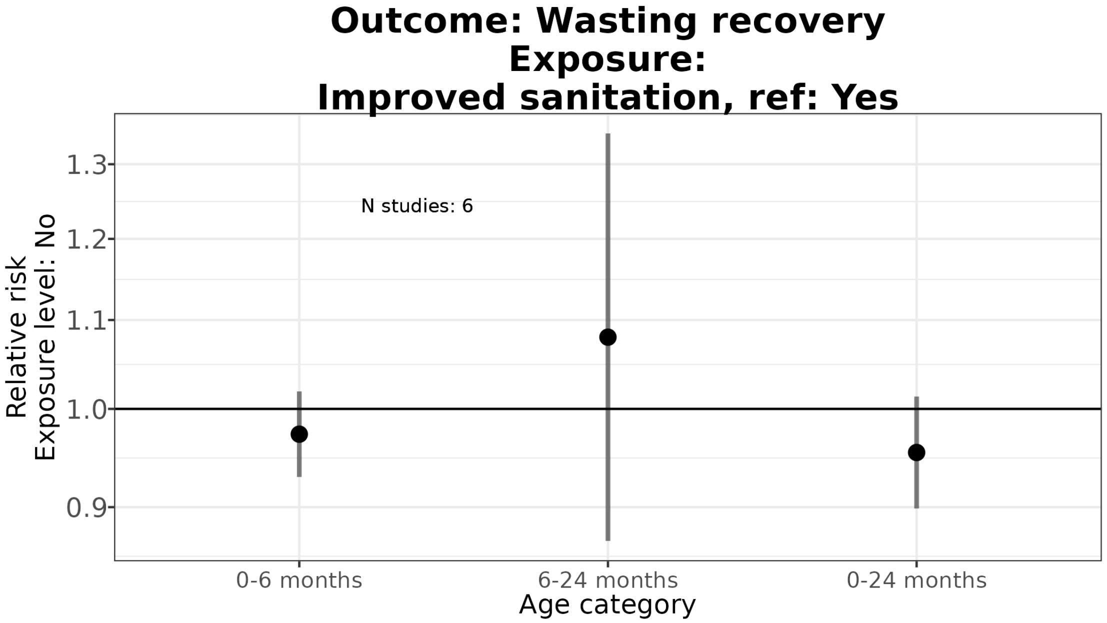
Outcome: Wasting recovery
Exposure:
Improved sanitation, ref: Yes
N studies: 6
Relative risk
Exposure level: No
1.3
1.2
1.1
1.0
0.9
0-6 months
6-24 months
0-24 months
Age category

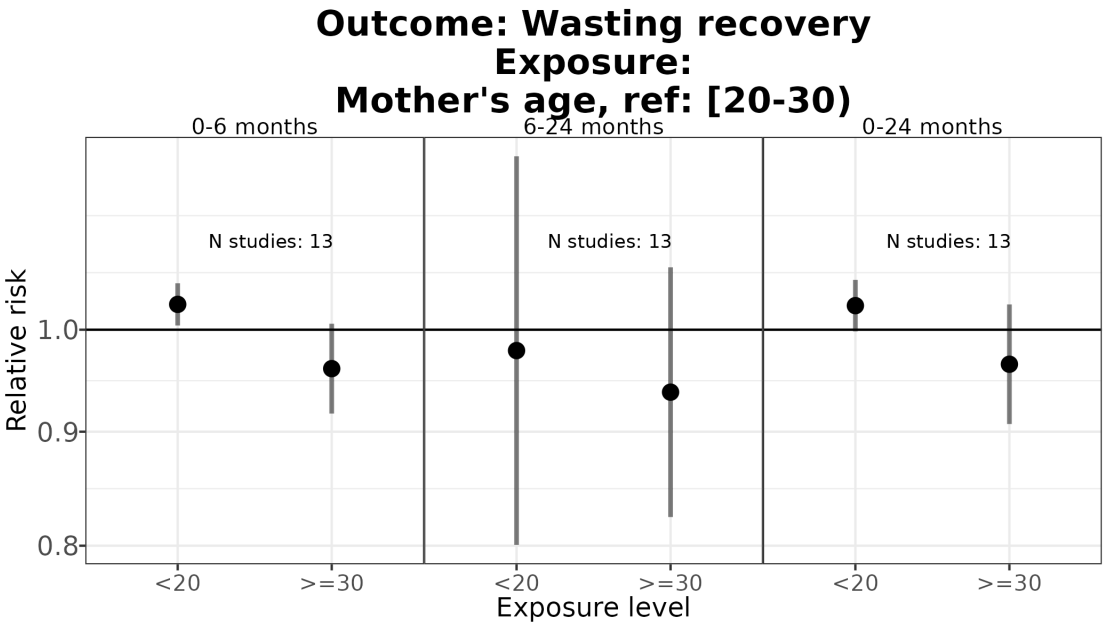
Outcome: Wasting recovery
Exposure:
Mother's age, ref: [20-30)
0-6 months
6-24 months
0-24 months
N studies: 13
N studies: 13
N studies: 13
Relative risk
1.0
0.9
0.8
<20
>=30
<20
>=30
<20
>=30
Exposure level

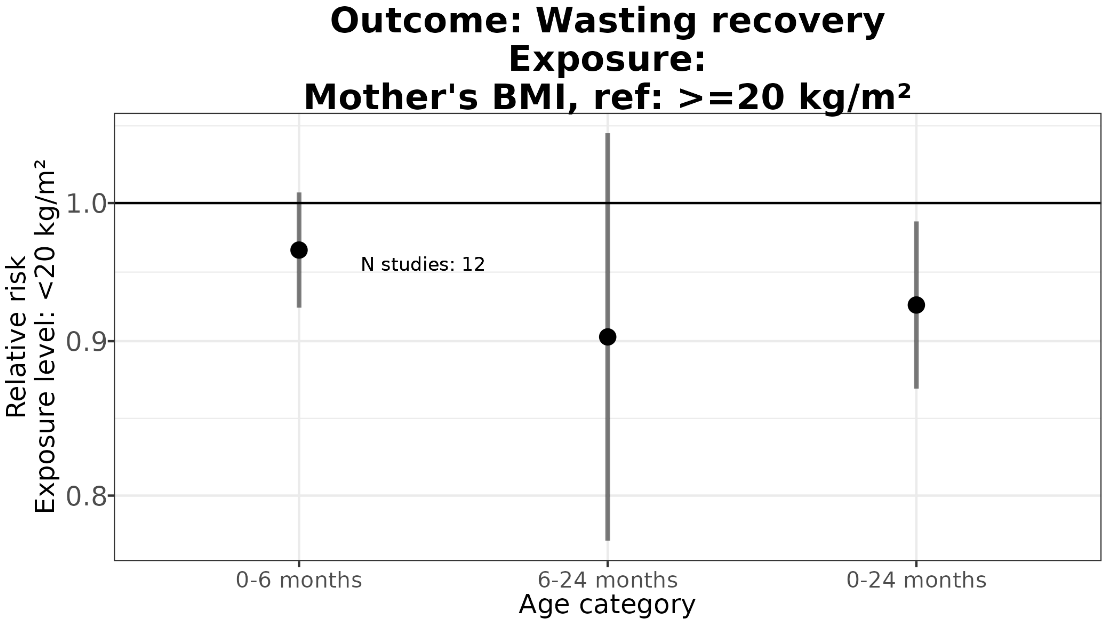
Outcome: Wasting recovery
Exposure:
Mother's BMI, ref: >=20 kg/m²
Relative risk
Exposure level: <20 kg/m²
1.0
0.9
0.8
N studies: 12
0-6 months
6-24 months
0-24 months
Age category

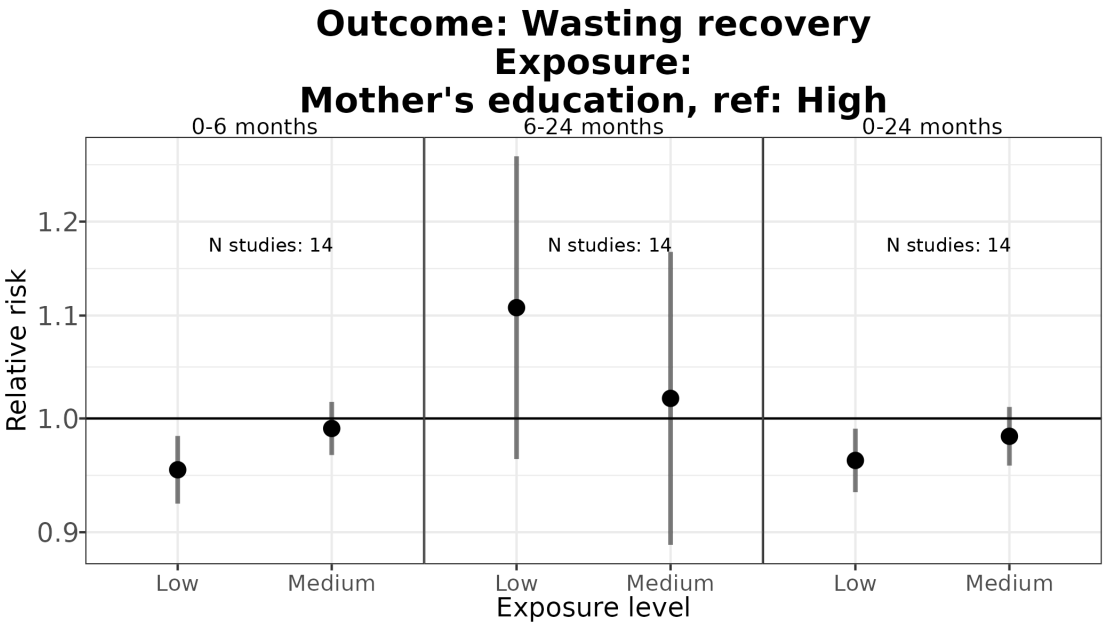
Outcome: Wasting recovery
Exposure:
Mother's education, ref: High
0-6 months
6-24 months
0-24 months
N studies: 14
N studies: 14
N studies: 14
Relative risk
1.2
1.1
1.0
0.9
Low
Medium
Low
Medium
Low
Medium
Exposure level

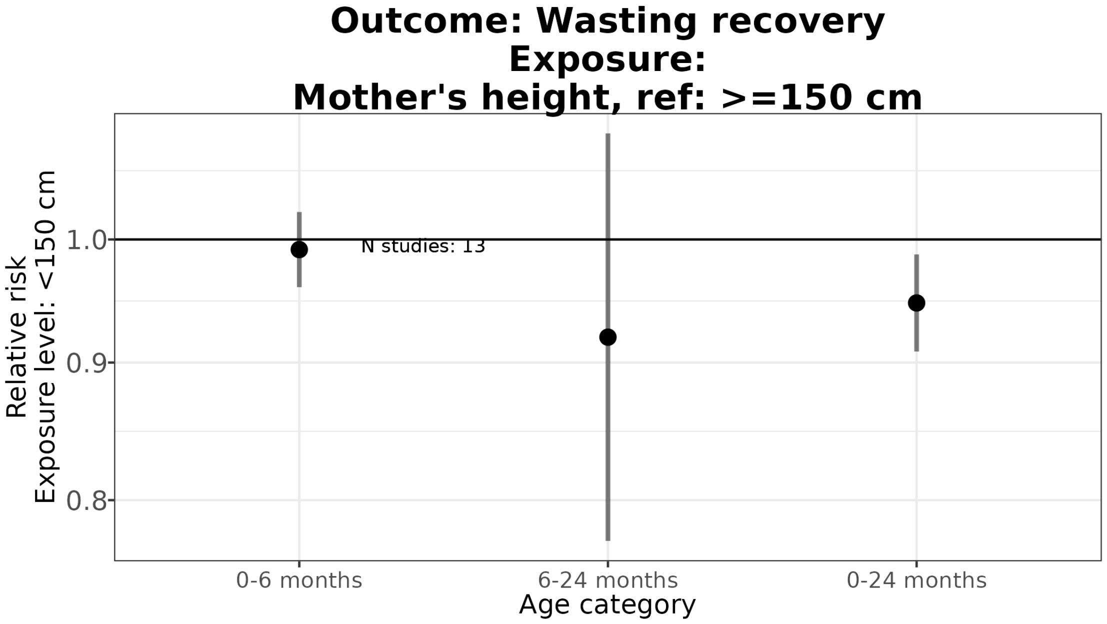
Outcome: Wasting recovery
Exposure:
Mother's height, ref: >=150 cm
N studies: 13
Relative risk
Exposure level: <150 cm
1.0
0.9
0.8
0-6 months
6-24 months
0-24 months
Age category

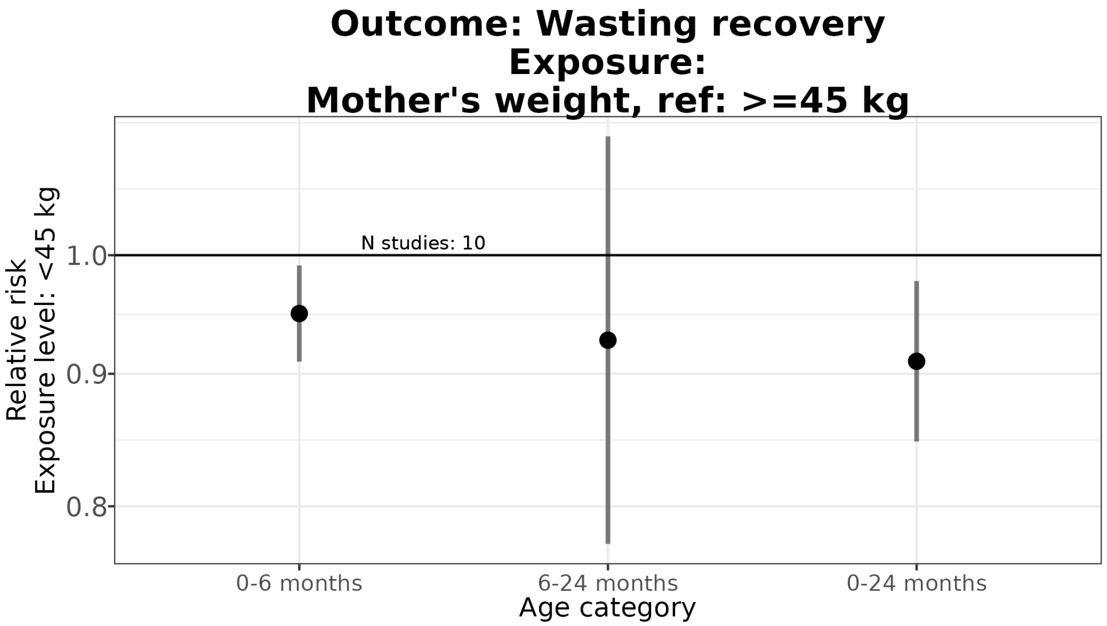
Outcome: Wasting recovery
Exposure:
Mother's weight, ref: >=45 kg
Relative risk
Exposure level: <45 kg
N studies: 10
1.0
0.9
0.8
0-6 months
6-24 months
0-24 months
Age category

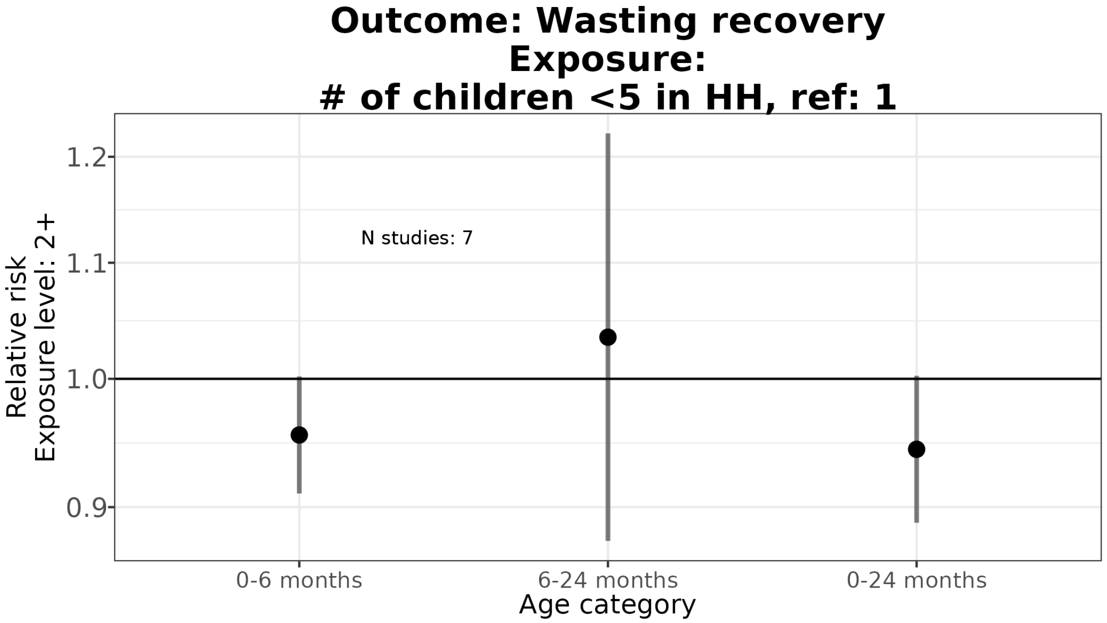
Outcome: Wasting recovery
Exposure:
# of children <5 in HH, ref: 1
Relative risk
Exposure level: 2+
N studies: 7
1.2
1.1
1.0
0.9
0-6 months
6-24 months
0-24 months
Age category

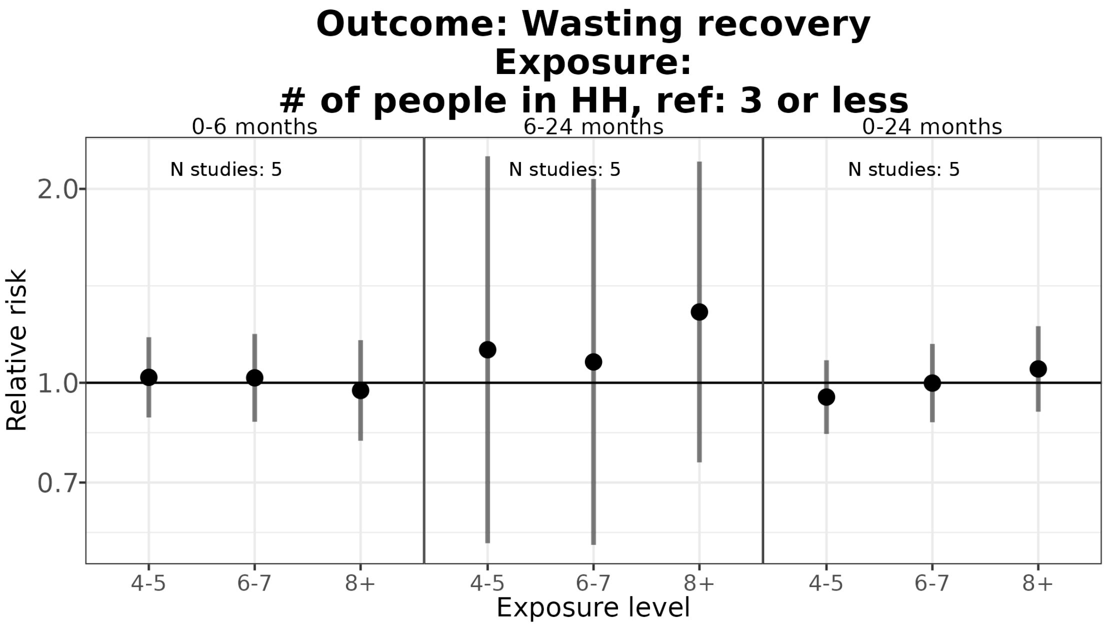
Outcome: Wasting recovery
Exposure:
# of people in HH, ref: 3 or less
0-6 months
6-24 months
0-24 months
N studies: 5
N studies: 5
N studies: 5
2.0
1.0
0.7
Relative risk
4-5
6-7
8+
4-5
6-7
8+
4-5
6-7
8+
Exposure level

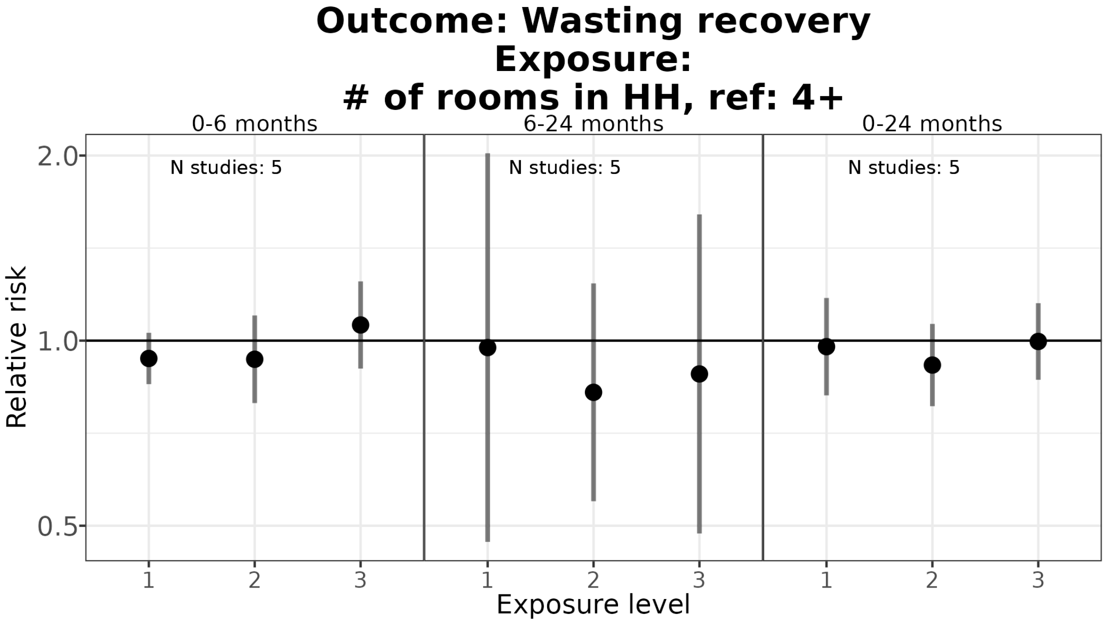
Outcome: Wasting recovery
Exposure:
# of rooms in HH, ref: 4+
0-6 months
6-24 months
0-24 months
N studies: 5
N studies: 5
N studies: 5
2.0
1.0
0.5
Relative risk
1
2
3
1
2
3
1
2
3
Exposure level

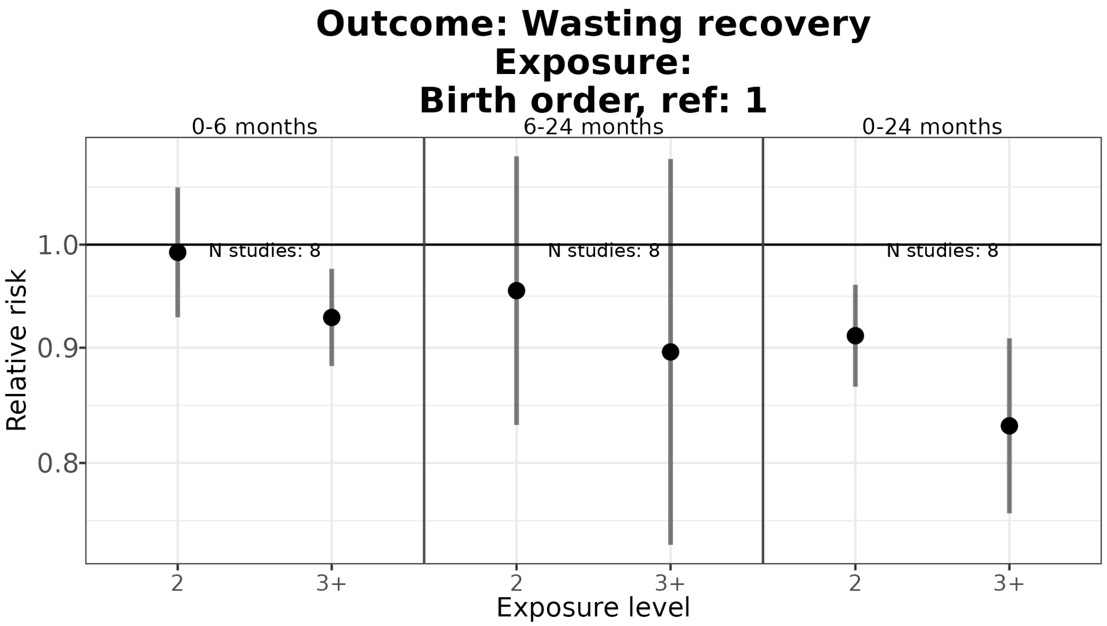

Outcome: Wasting recovery
Exposure:
Birth order, ref: 1
0-6 months
6-24 months
0-24 months
N studies: 8
N studies: 8
N studies: 8
1.0
0.9
0.8
Relative risk
2
3+
2
3+
2
3+
Exposure level


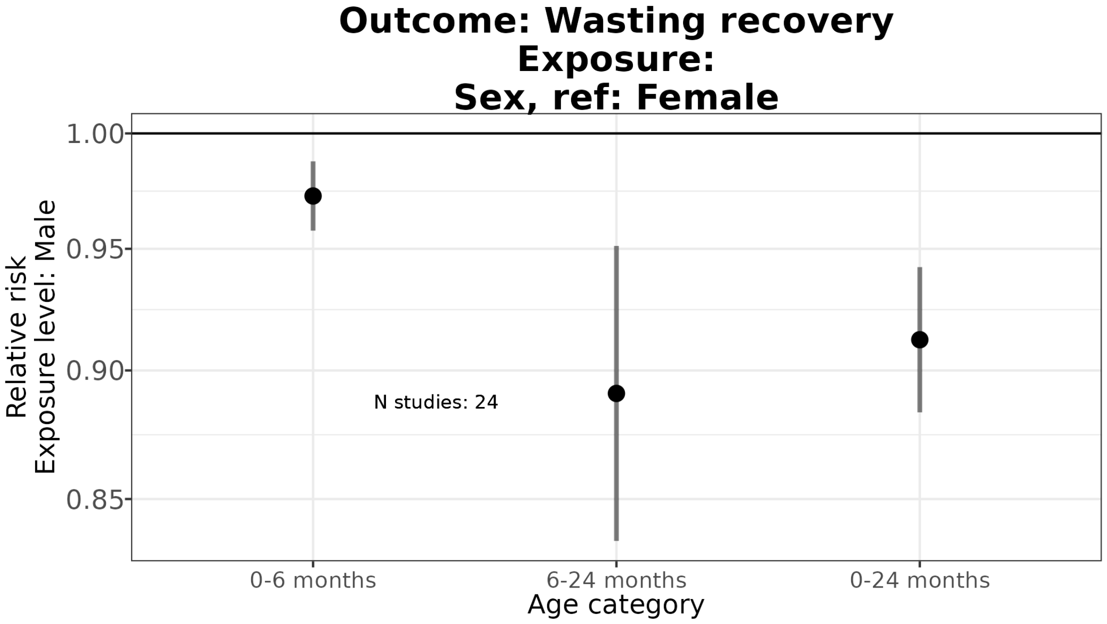

Outcome: Wasting recovery
Exposure:
Sex, ref: Female
1.00
0.95
0.90
0.85
Relative risk
Exposure level: Male
N studies: 24
0-6 months
6-24 months
0-24 months
Age category

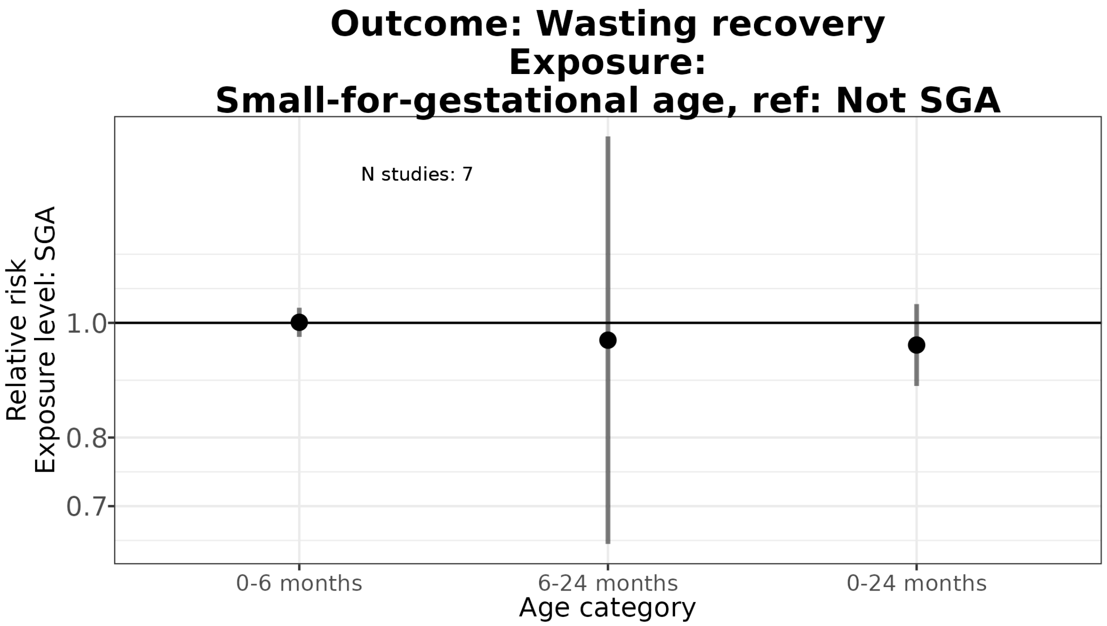
Outcome: Wasting recovery
Exposure:
Small-for-gestational age, ref: Not SGA
N studies: 7
Relative risk
Exposure level: SGA
1.0
0.8
0.7
0-6 months
6-24 months
0-24 months
Age category

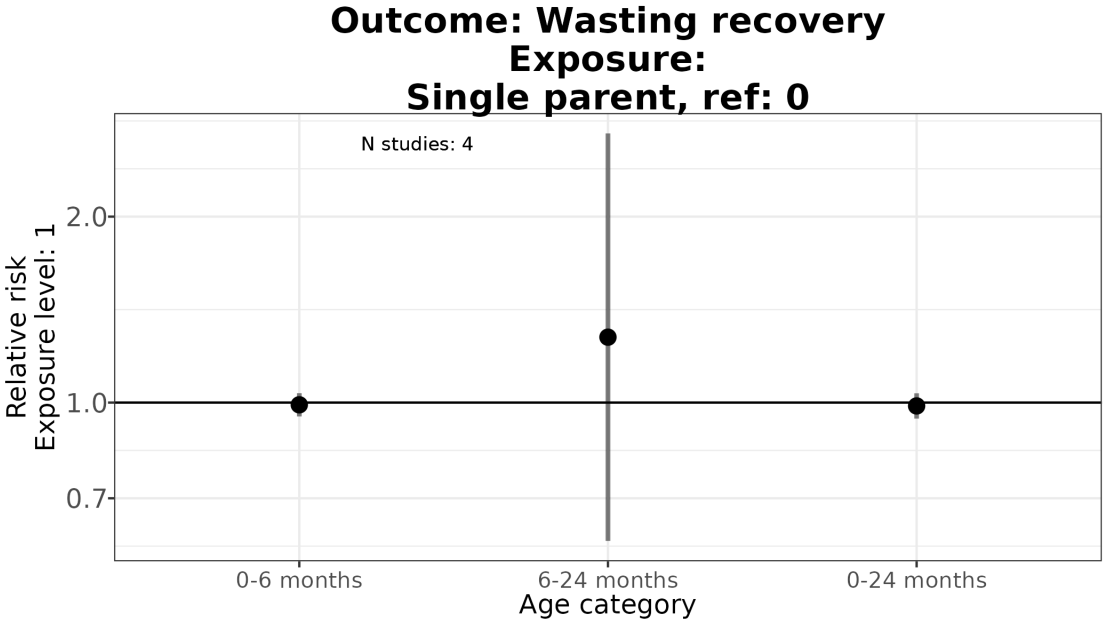
Outcome: Wasting recovery
Exposure:
Single parent, ref: 0
N studies: 4
Relative risk
Exposure level: 1
2.0
1.0
0.7
0-6 months
6-24 months
0-24 months
Age category
