# Supplementary material for: Causes and consequences of child growth faltering in low-resource settings
Source: Nature. 2023 Sep 13;621(7979):568–76. doi: 10.1038/s41586-023-06501-x (PMC10511328; doi:10.1038/s41586-023-06501-x)
Supplement: Supplementary file 2 — Reporting Summary [file 41586_2023_6501_MOESM2_ESM.pdf]

Corresponding author(s): Andrew Mertens, Benjamin F. Arnold

Last updated by author(s): Apr 6, 2023

Reporting Summary

Nature Portfolio wishes to improve the reproducibility of the work that we publish. This form provides structure for consistency and transparency in reporting. For further information on Nature Portfolio policies, see our Editorial Policies and the Editorial Policy Checklist.

Statistics

For all statistical analyses, confirm that the following items are present in the figure legend, table legend, main text, or Methods section.

n/a	Confirmed	
☐	☒	The exact sample size (*n*) for each experimental group/condition, given as a discrete number and unit of measurement
☐	☒	A statement on whether measurements were taken from distinct samples or whether the same sample was measured repeatedly
☒	☐	The statistical test(s) used AND whether they are one- or two-sided<br>*Only common tests should be described solely by name; describe more complex techniques in the Methods section.*
☐	☒	A description of all covariates tested
☐	☒	A description of any assumptions or corrections, such as tests of normality and adjustment for multiple comparisons
☐	☒	A full description of the statistical parameters including central tendency (e.g. means) or other basic estimates (e.g. regression coefficient) AND variation (e.g. standard deviation) or associated estimates of uncertainty (e.g. confidence intervals)
☐	☒	For null hypothesis testing, the test statistic (e.g. *F*, *t*, *r*) with confidence intervals, effect sizes, degrees of freedom and *P* value noted<br>*Give P values as exact values whenever suitable.*
☒	☐	For Bayesian analysis, information on the choice of priors and Markov chain Monte Carlo settings
☐	☒	For hierarchical and complex designs, identification of the appropriate level for tests and full reporting of outcomes
☐	☒	Estimates of effect sizes (e.g. Cohen's *d*, Pearson's *r*), indicating how they were calculated

Our web collection on statistics for biologists contains articles on many of the points above.

Software and code

Policy information about availability of computer code

Data collection	This manuscript is a secondary data analysis of existing study data from 33 cohorts and trials, so we were not involved in original data collection.
Data analysis	All analyses were conducted using R statistical software, and scripts that reproduce all analyses are available on Github here: https://github.com/child-growth/ki-longitudinal-growth

For manuscripts utilizing custom algorithms or software that are central to the research but not yet described in published literature, software must be made available to editors and reviewers. We strongly encourage code deposition in a community repository (e.g. GitHub). See the Nature Portfolio guidelines for submitting code & software for further information.

Data

Policy information about availability of data

All manuscripts must include a data availability statement. This statement should provide the following information, where applicable:

- Accession codes, unique identifiers, or web links for publicly available datasets
- A description of any restrictions on data availability
- For clinical datasets or third party data, please ensure that the statement adheres to our policy

Data are available upon agreement from the data contributors of individual studies, whose contact information is available from the Bill and Melinda Gates Foundation Knowledge Integration project (email [TO BE ADDED] upon reasonable request.

Research involving human participants, their data, or biological material

Policy information about studies with human participants or human data. See also policy information about sex, gender (identity/presentation), and sexual orientation and race, ethnicity and racism.

Reporting on sex and gender	We use the term sex throughout; data on gender was not collected in the original studies used. We include sex as a risk factor and examine sex as an effect modifier of mortality risk (supplementary material)
Reporting on race, ethnicity, or other socially relevant groupings	We did not have information on the race or ethnic groups of study participants.
Population characteristics	See below.
Recruitment	N/A: Secondary analysis of 33 completed studies.
Ethics oversight	N/A

Note that full information on the approval of the study protocol must also be provided in the manuscript.

Field-specific reporting

Please select the one below that is the best fit for your research. If you are not sure, read the appropriate sections before making your selection.

☐ Life sciences ☒ Behavioural & social sciences ☐ Ecological, evolutionary & environmental sciences

For a reference copy of the document with all sections, see nature.com/documents/nr-reporting-summary-flat.pdf

Behavioural & social sciences study design

All studies must disclose on these points even when the disclosure is negative.

Study description	This study performed a quantitative analysis of de-identified secondary, longitudinal data on child growth.
Research sample	The data analyzed in this study were amassed as part of the Knowledge Integration (ki) initiative of the Bill & Melinda Gates Foundation, which aggregated observations on millions of participants from a global collection of studies on child birth, growth and development. We selected longitudinal cohorts from the database that met five inclusion criteria: 1) conducted in LMICs; 2) enrolled children between birth and age 24 months and measured their length and weight repeatedly over time; 3) did not restrict enrollment to acutely ill children; 4) enrolled at least 200 children; and 5) collected anthropometry measurements at least every 3 months. Thirty-three cohorts from 15 countries met inclusion criteria, and 83,671 children and 592,030 total measurements were included in this analysis. Four additional cohorts were included in analyses of mortality, including 14,317 more children and 70,733 additional measurements.
Sampling strategy	Not applicable.
Data collection	Not applicable.
Timing	Included datasets were collected between 1990 and 2014.
Data exclusions	We dropped 1,190 (0.2%) unrealistic measurements of LAZ (>+6 or <–6 Z), 1,330 (0.2%) measurements of WAZ (> 5 or < –6 Z), and 1,670 (0.3%) measurements of WLZ (>+5 or –5 Z), consistent with WHO recommendations.
Non-participation	Not applicable.
Randomization	Participants were not randomly assigned.

Reporting for specific materials, systems and methods

We require information from authors about some types of materials, experimental systems and methods used in many studies. Here, indicate whether each material, system or method listed is relevant to your study. If you are not sure if a list item applies to your research, read the appropriate section before selecting a response.

Materials & experimental systems

n/a	Involved in the study
☒	☐ Antibodies
☒	☐ Eukaryotic cell lines
☒	☐ Palaeontology and archaeology
☒	☐ Animals and other organisms
☒	☐ Clinical data
☒	☐ Dual use research of concern
☒	☐ Plants

Methods

n/a	Involved in the study
☒	☐ ChIP-seq
☒	☐ Flow cytometry
☒	☐ MRI-based neuroimaging
